# Improved large-scale prediction of growth inhibition patterns using the NCI60 cancer cell line panel

**DOI:** 10.1093/bioinformatics/btv529

**Published:** 2015-09-08

**Authors:** Isidro Cortés-Ciriano, Gerard J. P. van Westen, Guillaume Bouvier, Michael Nilges, John P. Overington, Andreas Bender, Thérèse E. Malliavin

**Affiliations:** ^1^Unité de Bioinformatique Structurale, Institut Pasteur and CNRS UMR 3825, Structural Biology and Chemistry Department, 75 724 Paris, France,; ^2^Medicinal Chemistry, Leiden Academic Centre for Drug Research, Einsteinweg 55, 2333CC, Leiden,; ^3^European Molecular Biology Laboratory European Bioinformatics Institute, Wellcome Trust Genome Campus, CB10 1SD, Hinxton, Cambridge, UK and; ^4^Centre for Molecular Science Informatics, Department of Chemistry, University of Cambridge, CB2 1EW Cambridge, UK

## Abstract

**Motivation:** Recent large-scale omics initiatives have catalogued the somatic alterations of cancer cell line panels along with their pharmacological response to hundreds of compounds. In this study, we have explored these data to advance computational approaches that enable more effective and targeted use of current and future anticancer therapeutics.

**Results:** We modelled the 50% growth inhibition bioassay end-point (GI_50_) of 17 142 compounds screened against 59 cancer cell lines from the NCI60 panel (941 831 data-points, matrix 93.08% complete) by integrating the chemical and biological (cell line) information. We determine that the protein, gene transcript and miRNA abundance provide the highest predictive signal when modelling the GI_50_ endpoint, which significantly outperformed the DNA copy-number variation or exome sequencing data (Tukey’s Honestly Significant Difference, *P* <0.05). We demonstrate that, within the limits of the data, our approach exhibits the ability to both interpolate and extrapolate compound bioactivities to new cell lines and tissues and, although to a lesser extent, to dissimilar compounds. Moreover, our approach outperforms previous models generated on the GDSC dataset. Finally, we determine that in the cases investigated in more detail, the predicted drug-pathway associations and growth inhibition patterns are mostly consistent with the experimental data, which also suggests the possibility of identifying genomic markers of drug sensitivity for novel compounds on novel cell lines.

**Contact:** terez@pasteur.fr; ab454@ac.cam.uk

**Supplementary information**: Supplementary data are available at *Bioinformatics* online.

## 1 Introduction

Cultured cell lines have, despite their inherent limitations, served as versatile preclinical disease models for cancer drug discovery (Weinstein, 2012). Recent large-scale multi-omics initiatives have catalogued the somatic alterations of cancer cell line panels along with their pharmacological response to hundreds of compounds (Barretina *et al.*, 2012; Garnett *et al.*, 2012; Shoemaker, 2006), enabling us to now make links between the compounds’ action and the genetic makeup of a cell on a large scale. The US National Cancer Institute (NCI) pioneered these efforts by assembling the NCI60 tumour cell line panel, which, to date, has been assayed for its sensitivity to over 130 000 compounds and has been extensively profiled at the biological level (Shoemaker, 2006). Although these cell line collections have proved valuable to identify genomic markers of drug sensitivity (Barretina *et al.*, 2012; Garnett *et al.*, 2012) and even led to the development of new drugs (Adams and Kauffman, 2004), the question now arises of how these pharmacogenomic dataset can be meaningfully mined, both to discover cancer subtype-specific drugs, and ultimately to aid in the design of personalized cancer treatments.

Previous studies of the NCI60 panel include the identification of drug mechanism of action (MoA) (Weinstein *et al.*, 1992), visualization tools for drug sensitivity data (Paull *et al.*, 1989; Weinstein *et al.*, 1997) and drug sensitivity predictions based on the cell line profiling data (Kutalik *et al.*, 2008; Riddick *et al.*, 2011; Staunton *et al.*, 2001; Szakacs *et al.*, 2004). Beyond algorithmic differences, the conceptual limitation shared by these models was the unfeasibility of extrapolating the data to novel compounds and cell lines *simultaneously*, as the cell line profiling data and chemical information were *separately* used as predictive features and were not integrated into a single machine learning model. To overcome this limitation, two recent studies have pioneered the combination of drug and cell line information [gene expression, gene copy number variations (CNVs) and mutation profiles] on the data from the Genomics of Drug Sensitivity in Cancer project (GDSC) for drug sensitivity prediction (Garnett *et al.*, 2012). The first study (Menden *et al.*, 2013) modelled the sensitivity of 608 cell lines to 131 drugs with Neural Networks and Random Forests, thereby obtaining a coefficient of determination (*R*^2^) of 0.64 on an external set. The second study (Ammad-ud-din *et al.*, 2014) applied kernelized Bayesian matrix factorization to model the sensitivity of 650 cell lines to 116 drugs, and obtained an *R*^2^ value of 0.32 when predicting the activity for new drugs on a set of cell lines in the training set. The authors showed that the combination of chemical and cell line information indeed improved model performance, which permitted the interpolation of drug activities to complete the missing entries in the bioactivity matrix of 482 cell lines by 116 drugs.

**Fig. 1.**
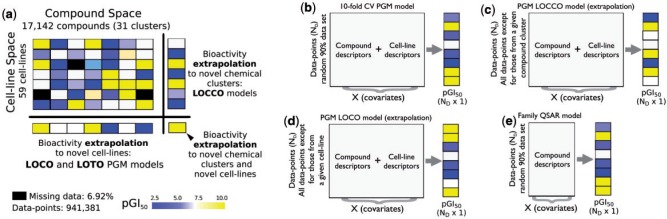
Pharmacogenomic modelling concept and illustration of the learning strategies explored. (**a**) The pGI_50_ values for 17 142 compounds on 59 cancer cell lines (941 831 data points) were modelled with RF and SVM models and conformal prediction. (**b**) Illustration of the training data used in the following learning strategies: (**b**) 10-fold CV PGM models (interpolation); (**c**) LOCCO; (**d**) LOCO; and (**e**) Family QSAR. As can be seen in (b–e), the training data used in each learning strategy differs with respect to (i) the subset of data-points from the whole dataset used for training and (ii) the type and combination of input descriptors, which can be only compound descriptors, only cell line descriptors, or the combination of both. In all models reported in this article, Morgan fingerprints were used as compound descriptors, whereas the dataset views indicated in Table 1 and four cell line kernels were used to encode the cell lines. Overall, this validation enabled us to assess the model’s performance in real-world settings, where the extrapolation to novel cell lines and compounds is often a necessary step

In this study, we now propose the *simultaneous* modelling of chemical and cell line information in a single machine learning model to predict the 50% growth inhibition bioassay end-point (GI_50_) of 17 142 compounds screened against 59 cancer cell lines from the NCI60 panel. Conformal prediction was implemented to predict the confidence intervals (CI) for individual predictions. The integration of these different, yet complementary, streams of information is often termed proteochemometrics or pharmacogemonic (PGM) modelling (Cortes-Ciriano *et al.*, 2015b;Wheeler *et al.*, 2013). In typical PGM models, each compound–cell line interaction is numerically encoded by the concatenation of the compound, Φ_c_(*c_d_*), and cell line, Φ_cl_(*cl_d_*), descriptors into a single vector, namely Φ_pair_(*c_d_*, *cl_d_*). Although this encoding approach is widely used, other approaches exist (Su *et al.*, 2010; Yamanishi *et al.*, 2010). For instance, in the case of kernel methods, each pair Φ_pair_(*c_d_*, *cl_d_*) is encoded by the tensor product of Φ_c_(*c_d_*) and Φ_cl_(*cl_d_*) (Jacob and Vert, 2008). Subsequently, these descriptors are related in a single machine-learning model to the specific biological readout of interest (Cortes-Ciriano *et al.*, 2015b; van Westen *et al.*, 2011). In practical terms, PGM thereby helps us to understand complex relationships between the compound structures and cell line features and enables the estimation of the bioactivity for (novel) compounds on (novel) cell lines (Fig. 1a).

We curated cell line profiling data consisting of 59 cell lines that were characterized using various genetic and proteomic profiling methods. From this, we assembled 14 profiling datasets, denoted here as dataset views. We benchmarked their predictive signal, and demonstrated that the simultaneous modelling of the compound and cell line information improves the prediction of compound potency across the NCI60 panel compared with models trained on only compound information. Our modelling approach permits us to interpolate and extrapolate compound bioactivities to novel cell lines and tissues in the NCI60 panel, and, to a lesser extent, to chemically dissimilar compounds. Finally, we demonstrate that the predicted bioactivities can be used to estimate growth inhibition patterns across the NCI60 panel and that the predicted drug-pathway associations are consistent with the experimental data published in the literature.

## 2 Methods

### 2.1 Datasets

The raw pGI_50_ values (−log_10_ GI_50_, M) for 17 142 compounds were downloaded from CellMiner (database version 1.4) (Reinhold *et al.*, 2012). The mean pGI_50_ values were calculated when several measurements were available for the same compound–cell line combination and the standard deviation of the replicates was considered as the experimental uncertainty of the measurements. The representation of the chemical structures (with respect to the aromaticity or stereochemistry) was normalized with the *StandardiseMolecules* function of the R package *camb* using the default values (Murrell *et al.*, 2015). The final dataset consisted of 17 142 compounds versus 59 cell lines (the NCI60 panel, except for ME.MDA_N) and a total of 941 831 data points, which corresponds to a matrix completeness of 93.08%. Table 1 summarizes the details of the profiling datasets used to describe the cell lines.

**Table 1.** Description of the dataset views benchmarked for the compound sensitivity prediction using the NCI60 panel

Original profiling dataset	Abbreviated data set view name	Details
Cell line fingerprints (Lorenzi *et al.*, 2009)	Cell Fingerprints	Number of short tandem repeats at 16 genomic loci
DNA copy-number variation (Varma *et al.*, 2014)	CNV	CNV for the 967 genes (Supplementary Table S1) exhibiting at least two mutations in the NCI60 panel. DNA gain (>3N, log_2_ = 0.58) was encoded as 1, DNA losses (<1N, log_2_ = −1) as −1, and the rest (2N) as 0
DNA copy-number variation (Varma *et al.*, 2014)	CNV Onc. & T. Suppre.	CNV for oncogenes and tumour suppressors (Supplementary Table S2)
mRNA (Reinhold *et al.*, 2010)	G.t.l ABC	Transcript levels (log_2_) of 47 ABC transporters
mRNA (Reinhold *et al.*, 2010)	G.t.l Onc. & T. Suppre.	Transcript levels (log_2_) of (i) oncogenes, and (ii) tumour suppressors
mRNA (Reinhold *et al.*, 2010)	G.t.l Kin.	Transcript levels (log_2_) of 402 human kinases (Supplementary Table S3)
mRNA (Reinhold *et al.*, 2010)	G.t.l 1000 genes	Transcript levels (log_2_) of the 1000 genes displaying the highest variability among the NCI60 panel (Supplementary Table S4)
mRNA (Reinhold *et al.*, 2010)	G.t.l 1000 pathways	Average transcript levels (log_2_) of the 1000 pathways displaying the highest variance among the NCI60 panel (Supplementary Table S5)
mRNA (Reinhold *et al.*, 2010)	G.t.l 1000 genes & Kin. & Onco. & T. Suppre.	Transcript levels (log_2_) of (i) the 1000 genes displaying the highest variance among the NCI60 panel, (ii) the human kinome, (iii) oncogenes, and (iv) tumour suppressors
mRNA (Reinhold *et al.*, 2010)	G.t.l Kin. & Onco. & T. Suppre.	Transcript levels (log_2_) of (i) the human kinome, (ii) oncogenes, and (iii) tumour suppressors
miRNA (Reinhold *et al.*, 2010)	miRNA	Expression (log_2_) of 627 miRNAs
Reverse-phase lysate arrays (Nishizuka *et al.*, 2003)	RPLA	Normalized protein abundance levels (log2) for 89 proteins (Supplementary Table S6)
Whole exome sequencing (Abaan *et al.*, 2013)	Exome	Mutation status (1: mutated, 0: non mutated) of 112 Type II variants (Supplementary Table S7) predicted to be deleterious (polyphen score > 0.85)
Whole exome sequencing & DNA copy-number variation	Exome & CNV	Concatenation of dataset views Exome seq. and CNV

The abbreviated names used in Fig. 3 are indicated in the second column. Prior biological knowledge, such as pathway information, was included in some dataset views, whereas the gene transcript levels and mutational status for genes implicated in cancer, kinases and ABC transporters were gathered independently and combined in the dataset views to assess the redundancy of their predictive signal

### 2.2 Compound descriptors

The compounds were described with circular Morgan fingerprints in count format, which encode the compound structures *via* radial atom neighbourhoods (Bender *et al.*, 2004). The fingerprints were calculated with the R package *camb* (Murrell *et al.*, 2015). The size of the fingerprints was set to 256 bits, and the maximum radius of the substructures considered was set to 2. We used the circular fingerprints because they have provided high retrieval rates in comparable studies (Bender *et al.*, 2009; Koutsoukas *et al.*, 2013).

### 2.3 Compound clustering

The compounds were clustered using periodic two-dimensional Self-Organizing Maps (SOMs) (Bouvier *et al.*, 2014). Two dimensions, here 50 × 50, determine the map size in a periodic manner, whereas the third dimension contained the compound fingerprints. Each vector along the third dimension is called a neuron, *v.* The same fingerprints used to train the PGM models served as input vectors to the SOMs. The SOM values were initialized from a uniform distribution spanning the values present in the input vectors. At each training step, the most similar neuron to the input vector considered was updated. The conventional Unified distance matrix (U-matrix) was calculated to delineate the clusters. The U-matrix value associated with a given neuron, ${\text{U}}_{\text{height}(v)}$, is defined as the average Euclidean distance between that neuron and its eight closest neighbours: ${\text{U}}_{\text{height}(v)}=\;\frac{1}{8}\;\underset{\mu \in N(v)}{\sum }{\text{E}}_{d}(v,\mu )$, where N(v) is the set of neighbours and ${\text{E}}_{d}$ is the Euclidean distance between neurons. A distance threshold value was then applied to the U-matrix to define the contours of the compound clusters (Cortes-Ciriano *et al.*, 2015c).

### 2.4 Model training

Random Forest (RF) models (Breiman, 2001) were trained with the *ensemble.RandomForestRegressor* module of the python library scikit-learn (Pedregosa *et al.*, 2011) using the following parameters: (i) number of trees in the forest: 100 (Sheridan, 2013); (ii) criterion to assess the quality of a split: mean squared error; (iii) minimum number of data points to split a node: 1; (iv) minimum number of data points in a leaf to keep a given node split: 1; (v) maximum number of randomly selected descriptors considered when splitting a node (*mtry*): dimensionality of the input space. Radial-kernel Support Vector Machine (SVM) models were trained using the *svm* module of the python library scikit-learn (Pedregosa *et al.*, 2011). The parameter values were optimized with a 10-fold cross-validation (CV) and a grid search. The parameter grid was composed of the following values: (i) *C*: {2^−^^8^, 2^−^^6^,…, 2^2^, 10^1^, 10^2^, 10^3^} and (ii) *σ*: {2^−^^8^, 2^−^^6^,…, 2^2^}. All calculations were performed on 16 Intel Xeon E5-2670 processors and a total memory of 256 GB. The training times on the complete dataset ranged between 6 and 8 h.

### 2.5 Learning strategies

#### 2.5.1. PGM models

In the following, we define ‘PGM models’ as those models simultaneously trained on (i) compound descriptors and on (ii) one cell line profiling data set view.

- Assessment of the interpolation power with a 10-fold CV (10-fold CV PGM) model: a given dataset was randomly divided into (i) a training set comprising 90% of the data points and (ii) a test set comprising the remaining 10% of the data (Fig. 1b). This process was repeated 10 times, each time leaving out a random subset of the data, which enabled the generation of predicted values for all data points. Thus, although the test set is comprised of compounds and cell lines that are also present in the training data, the training and test sets are comprised of different compound–cell line *pairs.* The interpolation power of these 10 models was evaluated on a *per cell line* and on a *per compound cluster basis.* To this end, the RMSE and *R*^2^_0_ values were calculated on subsets of the test set grouped by cell line (cell line-averaged performance) or by the compound cluster (compound cluster-averaged performance).
- Leave-One-Compound-Cluster-Out (LOCCO) models (Fig. 1c): all data points annotated on a given chemical cluster were left out as a test set; a PGM model was trained on the training set, and the values for the test set were predicted. These steps were repeated for each compound cluster. This scenario reflects the practical situation where a model predicts pGI_50_ values for chemically novel compounds, and thus permits the assessment of the extrapolation capabilities of a PGM model in chemical space.
- Leave-One-Cell-Line-Out (LOCO) models (Fig. 1d): this validation scenario is similar to LOCCO, except that the data left out for the test set are now all of the data obtained for a particular cell line. This scenario intends to mimic the situation where the PGM model is employed to extrapolate the data to novel cell lines.
- Leave-One-Tissue-Out (LOTO) models: The performance of the PGM models was further evaluated by extrapolating to cell lines whose tissue of origin was not present in the training set. This scheme is similar to LOCO, except for the fact that all of the cancer cell lines originating from the same tissue were left out from the training set each time. It was employed because cell lines from the same tissue, despite their genetic differences, often still share commonalities with regard to compound activity.

To quantify the improvement of the PGM models over models exclusively trained on compound fingerprints (compound-only models), the following one-space learning strategies were explored (Brown *et al.*, 2014).

#### 2.5.2 Compound-only (QSAR) models

- Family Quantitative Structure-Activity Relationship (QSAR F): the models were exclusively trained using the compound fingerprints as the input features (Fig. 1e). A QSAR F model serves to assess whether the explicit inclusion of the cell line information improves the prediction of compound activity; if the PGM model displays a performance very similar to the QSAR F model, the inclusion of cell line information would add little benefit to the modelling of compound activity.
- Individual QSAR models *per* cell line: one QSAR model per cell line was exclusively trained on compound descriptors, which is, therefore, not able to include information from the biological side or measurements against other cell lines. In this case, the comparison was made with the compound cluster-averaged interpolation power of the PGM models to evaluate whether the integration of the compound and cell line information leads to higher predictive ability with respect to the *per cell line* QSAR models.

#### 2.5.3 Cell line kernels

To assess whether the explicit inclusion of the cell line profiling data as input variables provides a higher predictive signal than the cell line kernels (Jacob et al., 2008), which encode cell lines with a vector quantifying the similarity among cell lines in a given space (e.g. gene expression), we trained models with two sources of information, namely: (i) compound descriptors and (ii) one of the following cell line kernels:

- *Dirac* kernel (Jacob *et al.*, 2008) or cell line identity fingerprints (CLIFP) (Brown *et al.*, 2014). CLIFP are binary descriptors of length equal to the number of different cell lines considered, where each bit position corresponds to one cell line. Formally, CLIFP are defined as $\text{CLIFP}\left(i,\;j\right)=\;\delta (i-j)(i,\;j\;\in \;1,...,\;N_{\text{cells}})$, where *δ* is the Kronecker delta function and *N*_cells_ is the number of distinct cell lines. These descriptors are simply cell line indicator variables, and, therefore, no information on compound activity across different cell lines is shared during model training (Jacob *et al.*, 2008).
- *Multitask* (*MLT*) kernel (Jacob *et al.*, 2008). This kernel encodes the cell lines in the following form: $\text{MLT}(i,\;j)=\left(1+\delta (i-j)(i,\;j\;\in \;1,...,\;N_{\text{cells}})\right)/2$. When coupled to SVM, this kernel decomposes the model as a sum of two linear functions. The first function, common to all cell lines, learns the shared patterns of compound activity against all cell lines (e.g. substructures enriched for compound activity). The second function describes the aspects of compounds specific to each cell line. The cell lines are located at an equal distance, and information across cell lines can be shared during the learning phase (though their biological similarities and differences are not explicitly taken into account).
- *Cor. Transcriptome* kernel. This kernel encodes cell lines by defining a *N*_cells_ × *N*_cells_ matrix where each *i, j* entry corresponds to the Spearman’s Rank Correlation coefficient (*r*_s_) of the transcript levels (log_2_) of 19 965 genes (Reinhold *et al.*, 2010) between cell lines *i* and *j.*
- *Cor. Proteome* kernel. This case is similar to the previous kernel, with the difference that it considers the correlation of the protein levels of 8113 distinct proteins (Gholami *et al.*, 2014) for all cell line pairs.

### 2.6 Model validation

The predictive power of the models was assessed on the test set according to the RMSE_test_ and *R*^2^_0 test_ values (Golbraikh and Tropsha, 2002):

R0 test2=1−∑i=1N(yi−y^i r0)2∑i=1N(yi−y¯)2,RMSE=(y−y^)2N,

where *N* represents the size of the test set; $y_{i}$ the observed, ${\hat{y}}_{i}\;$the predicted, and $\bar{y}$ the average pGI_50_ values of those data points included in the test set; and ${\hat{y}}_{i\;}^{r0}=s\hat{y}$, with $s=\sum \;y_{i}{\hat{y}}_{i}/\sum {\hat{y}}_{i}^{2}$.

### 2.7 Assessment of the maximum and minimum achievable model performance

To assess the maximum and the minimum achievable RMSE_test_ and *R*^2^_0 test_ values based on the experimental uncertainty of the pGI_50_ values, we generated the simulated data as follows:

- *Maximum performance.* A sample, *A*, of the same size as the test set was randomly extracted from the vector containing the whole set of pGI_50_ values. Subsequently, the experimental uncertainty (multiplied by −1 in half of the cases) was added to each data point in *A*, thus defining the sample *B.* We define experimental uncertainty as the standard deviation of replicate pGI_50_ measurements. In cases where no experimental uncertainty was available for a data point, the mean of the available replicate-averaged experimental uncertainties, namely 0.27 pGI_50_ units, was used. Next, the RMSE_test_ and *R*^2^_0 test_ values were calculated for *A* with respect to *B.* These steps were repeated 1000 times, which led to the distributions of the maximum achievable RMSE_test_ and *R*^2^_0 test_ values.
- *Minimum performance.* The procedure was the same as in the previous case, except that sample *A* was randomized before calculating the RMSE_test_ and *R*^2^_0 test_ values.

### 2.8 Conformal prediction

Conformal prediction builds upon past experience, i.e. the data points in the training set, to define the confidence intervals (CI) for individual predictions, $\hat{y}$, with an error of 1 − *ε*, where *ε* is the tolerated error. The non-conformity score, *α*_new_, for a new data point, *x*_new_, quantifies how distant it is with respect to the data points from the training set, $X={\left\{x_{i}\right\}}_{i=i}^{n}$. Here, the non-conformity scores were calculated as: $\alpha_{\text{new}}=\frac{\left|y_{\text{new}}-\;{\hat{y}}_{\text{new}}\right|}{{\hat{\rho }}_{y_{\text{new}}}}$, where ${\hat{\rho }}_{y_{\text{new}}}$ is the predicted error for *x*_new_ with an error model. The main advantage of conformal prediction is that the CI are always valid, i.e. a confidence of 0.8 means that the predicted confidence regions will contain the observed value in at least 80% of the cases (Norinder *et al.*, 2014).

To calculate and validate the predicted CI, the following protocol (Norinder *et al.*, 2014) was implemented. First, the entire dataset was randomly divided into an external set (20% of the data) and a training set (80%). The latter was subsequently split into a calibration set (30%) and a proper training set (70%). Thus, these three sets include data from the entire sets of cell lines and compounds. Two models were trained on the proper training set, the first of which predicted the pGI_50_ values (point prediction model), whereas the second predicted the errors in the prediction (error model), $\hat{\rho }$. Both models were trained with the compound fingerprints and the ‘G.t.l 1000 genes’ dataset view as input features using the *complete* dataset. The point prediction model was generated by training an RF model on the proper training set with a 10-fold CV and with pGI_50_ values as the dependent variable. The generated CV predictions served to calculate the residuals (i.e. prediction errors) for the data points in the proper training set. Subsequently, the error model was generated by training an RF model on the proper training set using these residuals as the dependent variable. The pGI_50_ values and prediction errors were subsequently predicted for the calibration set and employed to calculate the vector of non-conformity scores for this set, which, after being sorted in increasing order, led to: $\alpha_{\text{calib}}={\left\{\alpha_{{\text{calib}}_{j}}\right\}}_{j=1}^{N_{\text{calib}}}$, where $N_{\text{calib}}$ corresponds to the number of data points in the calibration set. The *α* value for a given confidence level, namely $\alpha_{1-\varepsilon }$, was calculated as follows: $\alpha_{1-\varepsilon }=\alpha_{{\text{calib}}_{j}}\;\text{if }j\equiv {\lfloor N}_{\text{calib}}*\left(1-\varepsilon )\right\rfloor ,$ where ≡ indicates identity. This corresponds to traversing the set $\alpha_{calib\;}$at its $\alpha_{1-\varepsilon }$ element, namely 1 − *ε.* Finally, the prediction errors (${\hat{\rho }}_{y}$) and the pGI_50_ values ($\hat{y}$) were predicted for the test set. The CI was calculated as: $\hat{y}\pm \;\alpha_{1-\varepsilon }*{\hat{\rho }}_{y}$.

### 2.9 Pathway–drug associations

The average log_2_ gene transcript level for the genes in each pathway of the MSigSB C2 Canonical Pathways gene set (Liberzon *et al.*, 2011) was defined as the expression level of each pathway. To assess the association between the drug response (pGI_50_) and the expression of a given pathway, we fitted a linear model considering the tissue type, *T*, as a blocking factor (Haibe-Kains *et al.*, 2013), which was defined as: $p{\text{GI}}_{50}=\;\beta_{\text{p}}P_{i}+\;\beta_{\text{T}}T_{i}+\;\varepsilon ,$ where $P_{i}\;$corresponds to the average expression of pathway *i* in a given cell line, T_i_ to the tissue of origin of that cell line, and *ε* to the error term. The $\beta_{\text{p}}\;$values determined the strength of the associations, whereas their significance was estimated by the statistical significance of $\beta_{\text{p}}$ (two-sided *t*-test, *α* = 0.05).

## 3 Results

### 3.1 Summary of the cell line profiling dataset views

We collected seven profiling datasets for 59 cell lines from the NCI60 panel, excluding ME.MDA-N due to the lack of gene transcript microarrays (Table 1). These molecular/phenotype datasets were combined in a variety of ways, which we termed dataset views in analogy to database views (Costello *et al.*, 2014). The dataset views may consist of (i) a profiling dataset, (ii) a subset thereof, e.g. gene transcript levels of gene sets or (iii) a modification of the dataset to which prior knowledge is added, e.g. the calculation of pathway expression levels based on knowledge of the cell signalling networks. A total of 14 dataset views were defined, which are summarized in Table 1 and provided in the Supplementary Material.

In addition to the *complete* dataset, comprising all available data and including 17 142 distinct compounds (Supplementary Table S8) and 941 831 data points, we assembled three additional datasets: (i) the *uncorrelated bioactivities 0.5* dataset, comprising 3641 distinct compounds (199 940 data points) whose bioactivity distributions on the 59 cell lines exhibit standard deviations higher than 0.5 pGI_50_ units (Supplementary Table S9); (ii) the *uncorrelated bioactivities 1* dataset, comprising 165 distinct compounds (9376 data points) whose bioactivity distributions on the 59 cell lines exhibit standard deviations higher than 1 pGI_50_ unit (Supplementary Table S10); and (iii) the *high confidence* dataset, exclusively comprising data points averaged over at least two experiments (304 212 data points and 5302 distinct compounds). The *uncorrelated bioactivities 0.5 and 1* datasets served to assess the models’ performance on compounds displaying a dynamic range of bioactivities across the cell line panel, while the latter dataset served to evaluate whether the models’ performance improves when using replicate-averaged cell line sensitivity data (Supplementary Table S11 for details).

### 3.2 Chemical space characterization

To assess the chemical diversity in the data, we clustered the 17 142 compounds with SOMs (Supplementary Figure S1 and Section 2.3), which resulted in the definition of 31 distinct chemical clusters. Several clusters, e.g. 4 and 18, are highly homogeneous, as highlighted by the high inter-neuron similarity (blue areas in Supplementary Fig. S1). In contrast, other clusters are comprised of more diverse compounds (shown in red in Supplementary Fig. S1). For instance, cluster 2 (Supplementary Table S8) is composed of polycyclic aromatic compounds with diverse halogen substituents and topologies. The definition of structural clusters will be employed later to examine the extrapolation power of the PGM models to chemically dissimilar structures.

### 3.3 Comparison between RF and SVM

**Fig. 2.**
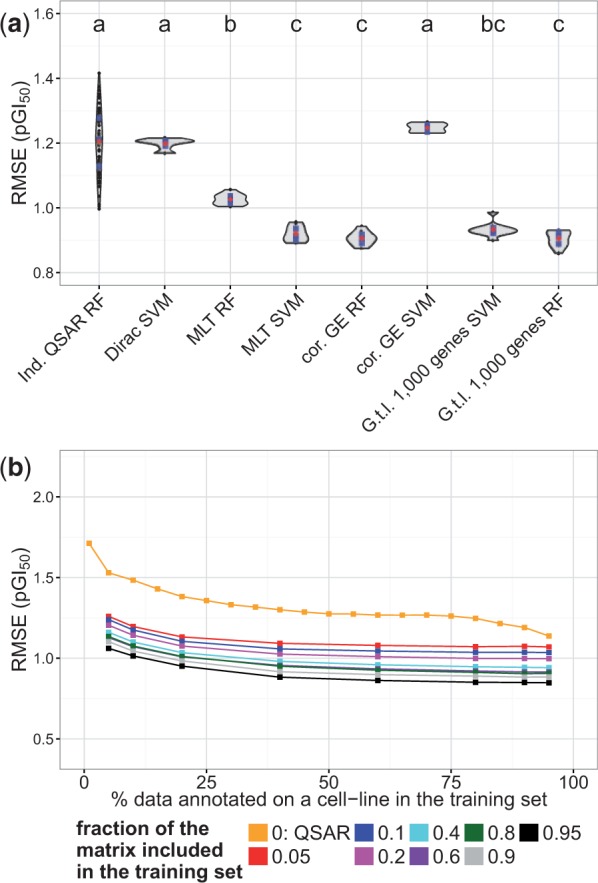
Comparison between (i) RF and SVM and (ii) the cell line kernel and PGM models. (**a**) The predictive power of the 10-fold CV RF and SVM models was compared on the *uncorrelated bioactivities 1* dataset across the cell line kernels explored and the data set view ‘G.t.l. 1000 genes’ (Table 1). The CV RMSE_test_ values on the left out sets in CV were used as a proxy to monitor the predictive power of the models. RF and SVM trained on the ‘G.t.l. 1000 genes’ dataset view displayed comparable predictive power, whereas RF and SVM exhibited diverse performance across the cell line kernels used in this study. Models sharing a letter label performed at the same level of statistical significance (Tukey’s HSD, *α* = 0.05). The blue points indicate the median and the interquartile range (25th–75th percentile), whereas the red points indicate the mean RMSE_test_ value. (**b**) Comparison between the individual QSAR and PGM models. The 10-fold CV Ind. QSAR models were trained on increasingly larger training sets and their performance was assessed on the left out data (orange). Thus, each point in the figure corresponds to the average 10-fold CV RMSE_test_ value across 59 models (one *per* cell line). The 10-fold CV PGM models were trained jointly on the compound and cell line descriptors (‘G.t.l. 1000 genes’ dataset view). For each model, the training set comprised a fraction of the data annotated on a given cell line (*x*-axis) and a percentage of the data annotated on the remaining cell lines (indicated in the legend). Overall, lower RMSE_test_ values are obtained when integrating information from several cell lines, indicating that the PGM models enable us to share information across cell lines and compounds, thereby outperforming the individual QSAR models

Although the statistical robustness of RF to model the chemical and biological data simultaneously has been empirically shown (Menden *et al.*, 2013), the mathematical notions by which information is shared across compounds and cell lines are poorly understood. Given that a comparison between the multi-task learning capabilities of RF and SVM in the field of pharmacogenomics is lacking, we first compared their performance in 10-fold CV when trained on the *uncorrelated bioactivities 1* dataset using (i) compound fingerprints and (ii) either gene expression data or a cell line kernels as the input features (Fig. 2a). We did not find statistically significant differences in the performance between the RF and SVM 10-fold CV PGM models trained on compound fingerprints and the dataset view ‘G.t.l. 1000 genes’ (*P* < 0.05). The performance of these models was comparable to that of the 10-fold CV PGM models encoding cell lines with the *cor. GE* and *MLT* kernels trained with RF and SVM, respectively (*P* < 0.05, labelled with ‘a’ in Fig. 2). Hence, in this dataset, encoding cell lines with kernels that are naïve with respect to the nature of the genomic makeup of the cell lines, in this case the *MTL* kernel, provides virtually the same predictive signal as the gene expression data, i.e. the ‘G.t.l. 1000 genes’ dataset view. Interestingly, the performance of SVM and RF across the cell line kernels explored was not constant, as the RF models outperformed SVM when using the *cor. Proteome* and *Dirac* kernels to encode cell lines, whereas the SVM models outperformed RF in the case of the *MLT* kernel, thus highlighting that information across compounds and cell lines is *not* shared in a similar way across RF and SVM. Based on the comparable predictive power of RF and SVM, we used RF to train all of the models presented in the following sections because RF (i) requires smaller training times than the kernel methods and (ii) are robust with respect to the value of their parameters (Supplementary Fig. S2 and Sheridan, 2013).

### 3.4 PGM model validation

The PGM models trained on the *complete* dataset with 10-fold CV using compound fingerprints and the dataset view ‘G.t.l 1000 genes’ as input features exhibited mean RMSE_test_ and *R*^2^_0 test_ values of 0.40 ± 0.00 pGI_50_ units and 0.83 ± 0.00 (*n* = 10), respectively. These values were consistent with the theoretical maximum and minimum achievable performance, which were 1.42 and 0.35 pGI_50_ units, respectively, for the RMSE_test_ and 0.96 and−0.96, respectively, for the *R*^2^_0 test_ (Supplementary Fig. S3). Moreover, the model performance did not stem from chance correlations, as *R*^2^_0 test_ values became negative when 75% of the pGI_50_ values were randomized (Clark and Fox, 2004) (Supplementary Fig. S4). Modelling the *high confidence* dataset led to a similar performance, with an RMSE_test_ of 0.45 pGI_50_ units and an *R*^2^_0 test_ value of 0.84. This indicates that the predictive power of RF does not decrease when we included the data points measured in only one experiment, which is in agreement with a recent benchmarking study on the noise sensitivity of machine learning algorithms in bioactivity modelling (Cortes-Ciriano *et al.*, 2015a). The 10-fold CV PGM models were further evaluated on the *uncorrelated bioactivities 0.5* dataset, where RMSE_test_ and *R*^2^_0 test_ values of 0.58 pGI_50_ units and 0.79, respectively, were observed. These RMSE_test_ and *R*^2^_0 test_ values were also in agreement with the maximum achievable performance estimated for the *uncorrelated bioactivities 0.5* dataset (Supplementary Fig. S5).

### 3.5 Comparisons between the PGM and QSAR models

To test whether the PGM models display higher predictive power than *per cell line* QSAR models, we trained (i) the individual QSAR 10-fold CV RF models with increasingly larger training sets, whose performance was assessed on the held-out data (orange in Fig. 2), on *the uncorrelated bioactivities 1* dataset and (ii) the 10-fold CV PGM RF models on the compound and cell line descriptors (‘G.t.l. 1000 genes’ dataset view). In the latter case, the training set comprised an increasingly larger percentage of the data annotated on a given cell line (*x*-axis in Fig. 2) and an increasingly larger percentage of the data annotated on the remaining cell lines (indicated in the legend of Fig. 2). We consistently obtained lower RMSE_test_ values with the PGM models than with the individual QSAR models, indicating that PGM modelling enables the sharing of information in the biological and chemical space. Likewise, the RMSE_test_ values systematically decreased as more data were added to the training set in the PGM models. The biggest RMSE_test_ difference between the individual QSAR and the PGM models were obtained when the least amount of data (5 or 10%) was assigned to the training set, whereas the lowest difference was found when almost all of the data available on a given cell line was used for training. This indicates that integrating data across different cell lines seems more beneficial when the data are relatively scarce.

On the *complete* dataset, the 10-fold CV PGM models slightly outperformed models trained exclusively on compound descriptors, namely QSAR F, with RMSE_test_ and *R*^2^_0 test_ values of 0.45 pGI_50_ units and 0.78, respectively. These results indicate (compared to an RMSE_test_ value of 0.40 pGI_50_ units and *R*^2^_0 test_ = 0.83 for the 10-fold CV PGM model) that the pGI_50_ values of the compounds are largely correlated across the cell line panel. However, the 10-fold CV PGM models increased the relative performance over the QSAR F models on the *uncorrelated bioactivities 0.5* dataset, with respective RMSE_test_ values of 0.58 versus 0.69 pGI_50_ units, respectively, highlighting the finding that the PGM models appear to be more suitable for modelling compounds exhibiting an uncorrelated pGI_50_ value on a cell line panel. Moreover, the 10-fold CV PGM models significantly outperformed individual cell line models, i.e.*,* QSAR (two-sided *t*-test, *α* = 0.05, *P* < 0.05), trained on the data points corresponding to a given cell line that exclusively used the compound descriptors as the input features. These individual models displayed an average RMSE_test_ value of 0.73 ± 0.05 pGI_50_ units (Supplementary Table S12). Therefore, integrating the biological information from different cell lines in a single PGM model improves the compound sensitivity prediction on the NCI60 panel over the QSAR models, and even more so in cases where activities across cell lines show less of a correlation.

### 3.6 Benchmarking the cell line profiling datasets

**Fig. 3.**
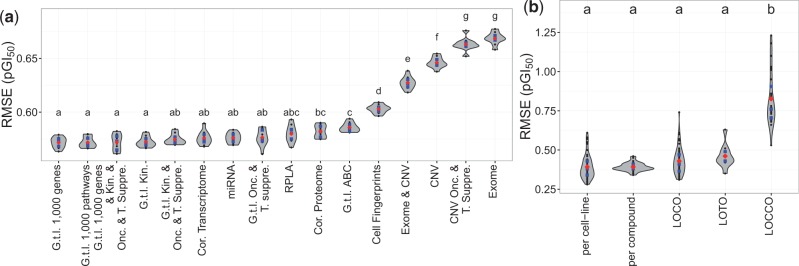
Benchmarking the cell line profiling dataset views for the cell line sensitivity prediction. (**a**) The predictive power of the 14 dataset views (Table 1) and two cell line kernels, namely *cor. Proteome* and *cor. Transcriptome*, was quantified by the RMSE values on the test set. For each dataset view, we trained the 10-fold CV PGM models on the *uncorrelated bioactivities 0.5* dataset. We found significant differences among the dataset views (ANOVA, *P* < 0.01). *Post-hoc* analyses (Tukey’s HSD, *α* = 0.05) were used to cluster the dataset views according to their predictive power. Dataset views sharing a letter label performed at the same level of statistical significance. We consistently found that the gene transcript levels and the abundance of proteins and miRNA led to the most predictive models (labelled with ‘a’). (**b**) The evaluation of both interpolation and extrapolation power was evaluated on the *complete* dataset. After finding significant differences across groups (ANOVA, *P* < 0.01), we found that the PGM models interpolate and extrapolate to new cell lines and tissues at the same level of statistical significance (Tukey’s HSD, *α* = 0.05). In contrast, we found statistically significant differences in the performance between extrapolation and interpolation to new *chemical* clusters. The blue points indicate the median and the interquartile range (25th–75th percentile), whereas the red points indicate the mean RMSE value

To benchmark the predictive signal across the 14 dataset views, we used the *uncorrelated bioactivities 0.5* dataset, as it contains the compounds displaying the least correlated pGI_50_ values on the cell line panel and thus is more challenging to model. For each dataset view, we trained a 10-fold CV PGM model using that dataset view and the compound fingerprints as the input features, which resulted in a total of 140 models (14 data set views × 10 CV folds). An analysis of variance (ANOVA) on the RMSE_test_ values (Fig. 3a) yielded significant differences between the models (*P* < 1 × 10^−^^17^). Similar to previous reports (Costello *et al.*, 2014; Jang *et al.*, 2014), we have also observed that the gene transcript levels led to the highest predictive power, with median RMSE_test_ values in the 0.56–0.58 pGI_50_ units range (Fig. 3a). However, the combination of transcript levels from different gene sets, e.g. kinases and ABC transporters (Fig. 3a), did not translate into increased performance, suggesting that these dataset views might contain redundant predictive signal. In addition, there were no significant differences in performance between the models trained on the gene transcript levels and those trained on the miRNA abundance or RPLA data [Tukey’s Honestly Significance Difference (HSD), *P* value < 0.05]. Interestingly, the performance of the models trained on the CNV or exome sequencing data was significantly worse (Fig. 3a), with RMSE_test_ values in the 0.63–0.68 pGI_50_ units range. This poorer performance could possibly be expected given the sparseness of these data, as the percentage of non-zero entries in the CNV and exome-seq matrices is 2.66 and 0.03%, respectively.

### 3.7 Bioactivity interpolation to the cell lines and compound clusters present in the training set

The interpolation power of the 10-fold CV PGM models was evaluated by calculating the RMSE_test_ values on subsets of the test set grouped by the cell line, cell line-averaged (Supplementary Fig. S6a), or compound cluster, compound cluster-averaged performance (Supplementary Fig. 6b). Thus, one average RMSE_test_ value across the 10-fold models was calculated for each cell line and compound cluster. If not otherwise indicated, the results presented in the following sections correspond to models trained on the *uncorrelated bioactivities 0.5* dataset with (i) compound fingerprints and (ii) the ‘G.t.l. 1000 genes’ dataset view as the input features. The cell line-averaged RMSE_test_ values ranged from 0.41 ± 0.01 (U251) to 0.86 ± 0.01 pGI_50_ units (HOP-92). We found significant differences for the tissue-averaged performance (Tukey’s HSD, *P* < 1 × 10^−^^16^), with RMSE_test_ values ranging from 0.48 ± 0.01 (prostate, displayed in cyan) to 0.70 ± 0.01 (leukaemia, displayed in green) pGI_50_ units (Supplementary Fig. 6a). In addition, the learning curves showed that the RMSE_test_ values of approximately twice the replicate-averaged experimental uncertainty (0.27 pGI_50_ units) and the maximum achievable performance (0.35 pGI_50_ units) can be obtained when <10% of the data is used as a training set (Supplementary Fig. S7). In contrast, an ANOVA analysis did not yield significant (*P* > 0.05) differences among the 31 chemical clusters (Supplementary Fig. S6b), with observed median RMSE_test_ values in the 0.48–0.65 pGI_50_ units range. Hence, overall, it has been found that the interpolation power is constant across the 31 chemical clusters, but not across tissues.

### 3.8 Extrapolation in the cell line and tissue space

We further evaluated the extrapolation power to novel cell lines and tissues with the LOCO and LOTO models using the *complete* dataset. The LOCO models exhibited mean RMSE_test_ values of 0.43 ± 0.08 pGI_50_ units (Fig. 3b and Supplementary Table S12), with the lowest and highest RMSE_test_ values of 0.31 and 0.61 observed for cell lines U251 and OVCAR-5, respectively. Notably, we found that the LOCO and cell line-averaged predictions are highly correlated (*r*_s_ = 0.92), indicating that interpolation and extrapolation to novel cell lines are correlated. The RMSE_test_ values for the LOTO models ranged between 0.35 (prostate) and 0.63 pGI_50_ units (leukaemia). Remarkably, the prediction errors were similar across the entire bioactivity range (Supplementary Fig. S8). Overall, we did not observe significant differences in performance among the LOCO, LOTO (Fig. 3b), cell line-averaged (Supplementary Fig. S6a), and compound cluster-averaged (Supplementary Fig. S6b) results (Tukey’s HSD, *P* < 0.05). We found that the RMSE values between the observed and predicted pGI_50_ values calculated using the LOCO and LOTO models for 47 out of 81 drugs, such as Imiquimod (NSC 369100) and Bendamustine (NSC 138783), were below 0.5 pGI_50_ units (Supplementary Fig. S9a and b). High RMSE values, between 1 and 1.5 pGI_50_ units, were observed for 11 drugs, such as the folate antimetabolite pemetrexed (NSC 698037) and Irinotecan (NSC 728073). Together, these data indicate that the PGM models extrapolate compound bioactivities to novel cell lines and tissues at the same level of statistical significance as for interpolation within a given cell line or tissue.

### 3.9 Extrapolation in the chemical space

A markedly different trend was observed for the ability of the PGM models to generalize across the chemical space, as assessed by the LOCCO models using the *complete* dataset. The LOCCO models exhibited mean RMSE_test_ values of 0.83 ± 0.17 pGI_50_ units (Fig. 3b and Supplementary Table S12), which differed significantly from the LOCO and LOTO results (Fig. 3b) (Tukey’s HSD, *P* < 0.01), and from the compound cluster-averaged interpolation performance (Supplementary Fig. S6b). Notably, the chemical diversity within the compound clusters was not correlated with model performance, as low RMSE_test_ values were consistently obtained for the heterogeneous and homogeneous clusters (Fig. S1 and Supplementary Table S13). The lowest RMSE_test_ value of 0.53 pGI_50_ units was obtained for cluster 24, which contains 485 compounds with polycyclic ring systems, generally with no more than three fused rings, as well as ring assemblies linked by sulphide, sulfinyl, secondary amine, carbonyl and alkyl groups (Supplementary Table S13). Cluster 16, which was modelled with the highest RMSE_test_ value of 1.23 pGI_50_ units, contains tri- and tetracyclic systems with hydroxybenzene, methoxybenzene and quinone functionalities. We obtained errors in prediction values below 0.5 pGI_50_ units for 15 out of 81 drugs in the *complete* dataset, and below 1 pGI_50_ units for 43 compounds. The worst predicted drugs were depsipeptide (NSC 630176) and the halichondrin B analogue NSC 707389, with respective errors in prediction of 4.29 and 4.35 pGI_50_ units (Supplementary Fig. S9c). Taken together, these results indicate that the range of errors is considerably large (>4 pGI_50_ units), and, hence, extrapolations in the chemical space remain a challenging task, and certainly on this particular dataset.

### 3.10 Conformal prediction provides informative CI

Conformal prediction was included in the modelling framework to provide CI for the individual predictions. The percentage of data points for which the predicted value lay within the calculated CI, which were calculated with an increasing confidence level, is highly correlated with the confidence level (Spearman’s *r*_s_ > 0.99, Supplementary Fig. S10). Therefore, the combination of the Random Forest and conformal prediction estimates the compound activity as a pGI_50_ region associated to a user-defined confidence level, which represents an easily interpretable estimate of the reliability of individual predictions.

### 3.11 Consistency of pathway–drug associations with predicted bioactivities

To investigate whether the bioactivities predicted by the PGM models make it possible to identify genomic markers of drug sensitivity, we evaluated the consistency between the pathway–drug associations inferred from the experimental and predicted bioactivities for the 37 FDA-approved drugs and the 19 compounds in the clinical trials (Supplementary Table S14) present in the *uncorrelated bioactivities 0.5* dataset. For each pathway, we fitted a linear model controlled by the tissue source, where the average pathway expression was considered as the predictor of drug sensitivity. Overall, no significant differences (Tukey’s HSD, *P* < 0.05) were observed between the pathway–drug associations calculated with the most predictive 10-fold CV PGM (Supplementary Fig. S11a) and LOCO models, as the median Spearman’s *r*_s_ values between the $\beta_{\text{p}}\;$coefficients estimated with (i) the experimental cell-line sensitivity data and (ii) the CV and LOCO predictions were in the 0.75–0.91 range (Supplementary Fig. S11b). These results indicate that the 10-fold CV and LOCO predictions reflect the association between gene expression summarized at the pathway level and the cell line sensitivity. However, significant differences were found among these groups and the LOCCO and LOTO models, for which the median Spearman’s *r*_s_ values were 0.63 and 0.03, respectively. We obtained similar results when considering only the pathways significantly associated to drug response [false discovery rate (FDR) < 20%] (Supplementary Fig. S11c and d).

Next, we analysed whether the pathway–drug associations are consistently predicted for drugs exhibiting diverse MoAs. Out of the 56 drugs considered, 26 exhibited median Spearman’s *r*_s_ values between the $\beta_{\text{p}}\;$ coefficients estimated with the experimental cell line sensitivity data and the 10-fold CV predictions in the 0.5–0.75 range, and 18 were above 0.75 (Supplementary Fig. S10b). High Spearman’s *r*_s_ values were obtained across the 22 distinct drug MoAs, indicating that no specific MoA is favoured. Notably, most Spearman’s *r*_s_ values increased (Supplementary Fig. S11d) when the calculation of the pathway–drug associations was restricted to the pathways significantly associated to drug response (FDR < 20%). Together, we can conclude that the identification of genomic markers of drug sensitivity is significantly dependent on the presence of cell lines originating from the same tissue and structurally similar compounds in the training set.

### 3.12 Prediction of the growth inhibition patterns using the NCI60 panel

The experimental and predicted growth inhibition patterns obtained by the most predictive models were highly correlated (Supplementary Fig. S11e), with median Spearman’s *r*_s_ values in the 0.53–0.58 range, and 32 out of the 56 drugs from the *uncorrelated bioactivities 0.5* dataset had values higher than 0.5 for (Supplementary Fig. S11f). Nevertheless, the LOCO, LOTO and LOCCO models (Tukey’s HSD, *P* value < 0.001) displayed a marked decrease in the *r*_s_ values (Supplementary Fig. S11e) with respect to the most predictive 10-fold CV PGM models.

**Fig. 4.**
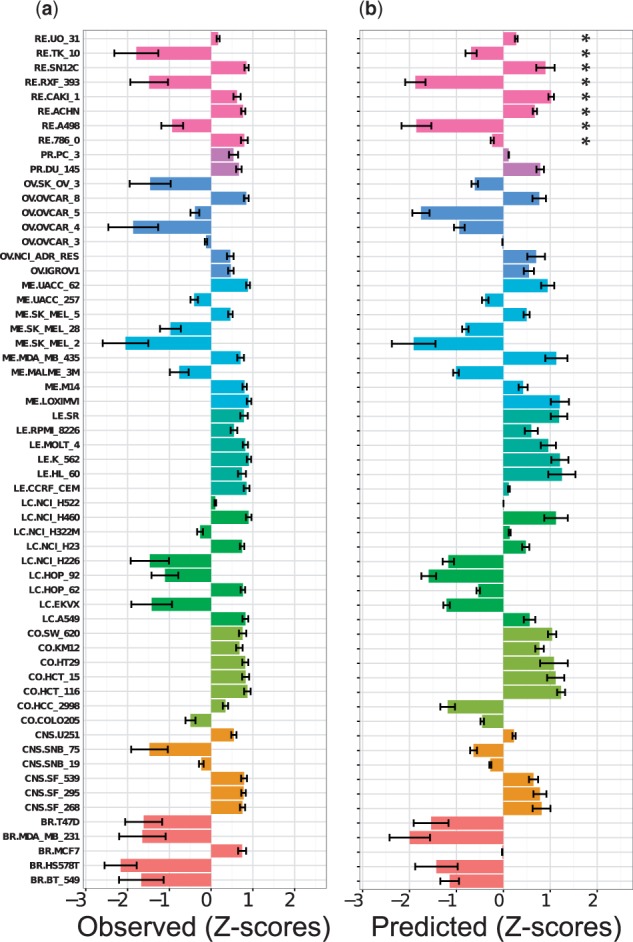
Evaluation of the predicted growth inhibition patterns for MTX on the NCI60 panel. (**a**) The relative growth inhibition pattern (*z*-scores) on the NCI60 panel was calculated from the experimental pGI_50_ values together with the experimental uncertainty of the measurements. (**b**) Predicted relative growth inhibition pattern of growth inhibition in the 10-fold CV model (i.e. interpolation) along with the 75% CI calculated using conformal prediction. Complex, and overall matching, inhibition patterns are reflected by the predictions. For instance, the TK-10, RXF-393 and A498 renal cell lines (marked with an asterisk) were predicted to be highly resistant to MTX, whereas the effect of MTX on sensitive cell lines, namely UO-31, SN12C, CAKI-1 and ACHN, was also correctly predicted. Cell lines originating from the same tissue are in the same colour (breast: red, central nervous system: orange, colon: olive green, lung cancer: dark green, leukaemia: turquoise, melanoma: blue, ovarian: blue, prostate: purple, renal: magenta)

The relative growth inhibition values using the NCI60 panel can be depicted in a bar plot with the pGI_50_ values transformed into *z*-scores. Figure 4 depicts the observed and predicted growth inhibition patterns for methotrexate (MTX) *via* the 10-fold CV PGM model. MTX was chosen for illustration, given its complex growth inhibition pattern. (Bar plots for the 56 drugs calculated with the predictions obtained with the 10-fold CV PGM, LOCO, LOTO and LOCCO models are provided in the Supplementary material.) The predictions accounted for 55 out of 59 cases for the relative sensitivity of the cell line. For instance, the six leukaemia cell lines (green turquoise) were predicted to be sensitive to MTX. Moreover, complex inhibition patterns for renal-derived cell lines (light magenta) were accounted for by the predictions, as the TK-10, RXF-393 and A498 cell lines were predicted to be highly resistant to MTX, whereas the effect of MTX on sensitive cell lines, namely UO-31, SN12C, CAKI-1 and ACHN, was also correctly predicted (Fig. 4b). Taken together, these data indicate that the drug sensitivity predictions were able to account for the complex patterns of cell line growth inhibition for this particular drug.

### 3.13 Comparisons to previous methods

We compared our results with previous studies (Ammad-ud-din *et al.*, 2014; Menden *et al.*, 2013) by applying our modelling procedure to the GDSC and CCLE datasets using the Morgan fingerprints as the compound descriptors, and the gene transcript levels for the 1000 genes displaying the highest variance across the cell line panel to describe the cell lines (Supplementary Text and Table S15). For the GDSC dataset, we obtained lower mean RMSE_test_ and higher *R*^2^_test_ values, namely 0.75 ± 0.01 and 0.74 ± 0.01 pGI_50_ units, respectively, with the 10-fold CV models compared with Menden *et al.* (2013), who obtained an RMSE_test_ of 0.83 and an *R*^2^_test_ of 0.72 pGI_50_ units, and Ammad-ud-din *et al.* (2014), who obtained an RMSE_test_ of 0.83 ± 1.00 and an *R*^2^_test_ of 0.32 ± 0.37 pGI_50_ units. The same trend was observed for the LOTO models, with mean RMSE_test_ and *R*^2^_test_ values of 0.81 ± 0.16 and 0.72 ± 0.08 pGI_50_ units, respectively, compared with Menden *et al.* (2013), with RMSE_test_ and *R*^2^_test_ values of 0.99 and 0.61 pGI_50_ units, respectively. The results obtained here, with RMSE_test_ values of 1.40 ± 0.80 pGI_50_ units, are comparable to those of Ammad-ud-din *et al.* (2014), with RMSE_test_ values of 0.85 ± 0.41 pGI_50_ units, when extrapolating to new compounds. However, it is important to mention that Ammad-ud-din *et al.* (2014) grouped compounds into sets at random and not based on chemical similarity, which likely made their extrapolation easier.

## 4 Discussion

The primary goal of this study was to capitalize on the increasing amount of *in vitro* cell line sensitivity and molecular profiling data of cancer cells to predict the growth inhibition patterns of compounds on the NCI60 panel. Although the principles of pharmacogenomic modelling are not new, this study represents novelty in the field, as, to our knowledge, it is the first effort to combine large-scale NCI anticancer screening data using the NCI60 panel with the available cell line profiling information, including error bars. Unlike previous modelling studies on the NCI60 panel (Abaan *et al.*, 2013; Paull *et al.*, 1989; Staunton *et al.*, 2001; Szakacs *et al.*, 2004; Wan and Pal, 2014), we simultaneously integrated the chemical information and cell line profiling data, which enables us to predict the growth inhibition patterns and to inter- and extrapolate on the chemical and cell line domains. In addition, coupling conformal prediction to the Random Forest enabled the definition of CI for the individual predictions.

We consistently found the highest predictive signals in the gene expression, miRNA and protein abundance data. The incorporation of prior biological knowledge by summarizing the gene expression data at the pathway level did not provide an additional predictive signal. Interestingly, high predictive power was attained with models trained on gene expression data from the genes displaying the most variable transcript levels across the cell line panel. Encoding cell lines with cell line kernels led, in some cases, to models with comparable predictive power to those trained explicitly on the cell line profiling data. Although encoding cell lines with cell line kernels may be sufficient to model the compound pGI_50_ values from the NCI60 panel, in our view this would not be the case on datasets comprising highly dissimilar cell lines. In addition, we note, in particular, that the *Dirac* and *MLT* kernels do not permit us to extrapolate in the cell line space, likely rendering them less useful in those cases. The sparseness of the CNV and exome sequencing data may be the reason for the poor model performance, and, therefore, it remains to be seen whether the modelling of cell line panels with more comprehensive mutational data leads to better predictions (Costello *et al.*, 2014).

A major challenge to the cell line sensitivity prediction is the extrapolation of compound bioactivities to novel cell lines and to structurally distinct compounds. We did not find significant differences in performance between the interpolation and the extrapolation to new cell lines (LOCO) and tissues (LOTO), with mean RMSE_test_ values in all cases smaller than twice the mean uncertainty value of the bioactivity measurements. This observation enables the prediction of compound activities on cancer cell lines for which little bioactivity data are currently available; however, extrapolation is still improved by the presence of cell lines from the same tissue/ontogeny in the training set. Given that similar compounds exhibit similar growth inhibition profiles (Shivakumar and Krauthammer, 2009), it was expected that the model performance would considerably decrease when predicting the activity of structurally dissimilar compounds. This was indeed the case because mean RMSE_test_ values of approximately three times the average experimental uncertainty were obtained when extrapolating in the chemical space (LOCCO). Although the error in prediction should ideally be close to the experimental uncertainty, this performance may still be useful for compound prioritization. Previous studies have shown that adding physicochemical descriptors or increasing the bit-string length of the Morgan fingerprints (set to 256 here) leads to higher predictive power when modelling a highly diverse set of molecules (De Bruyn *et al.*, 2013). Here, we did not obtain higher predictive power when increasing the bit-string length, when adding physicochemical descriptors to the compound fingerprints, or when using Morgan fingerprints in binary format (Cortes-Ciriano *et al.*, 2014). Our approach displays a higher predictive ability (10% decrease of CV RMSE) than the methods previously applied to the GDSC dataset (Supplementary Text and Supplementary Table S15).

Moreover, the application of our approach to the Cancer Cell line Encyclopedia (CCLE) (Barretina *et al.*, 2012) dataset led to statistically validated models, displaying *R*^2^_0 test_ values of∼0.74, which were comparable to those obtained on the GDSC and NCI60 datasets. Given that gene expression profiles for the 44 cell lines comprised in the CCLE and the NCI60 panel are highly correlated (Spearman’s *r*_s_ = 0.88) (Supplementary Fig. S12), we suggest that PGM models trained on the NCI60 panel could be applied to the CCLE cell line panel, which could identify new purposes for the 17 142 compounds considered here (Weinstein, 2012).

It is also important to consider how the cell line sensitivity is quantified for the application of the type of model presented here. Fallahi-Sichani *et al.* (2013) applied a multi-parametric analysis to a dataset comprising the activity of 64 anticancer drugs on 53 breast cancer cell lines. The results of this study indicate that the parameters of the dose–response curve vary systematically and depend on both the cell line as well as the drug class. For instance, the MoA of a drug has a strong influence on drug efficacy (*E*_max_), potency (IC_50_) and on the steepness of the drug response curve. Overall, this study indicates that parameters other than the potency of the drug response curve should be considered in studies comparing drug activity, as they are likely to provide crucial insights into the biology of the cell line’s response to drug treatment and into the drugs’ MoA.

An additional key aspect is the consistency of the *in vitro* cell line sensitivity data. A previous study (Haibe-Kains *et al.*, 2013) reported a rather low correlation of the cell line sensitivity data from the CCLE and GDSC datasets. We found low concordance between the NCI60 and the CCLE sensitivity data (Supplementary Fig. S13), and we did not obtain a high correlation between the predictions calculated with a model trained on the NCI60 data for eight drugs on 44 cell lines shared by the CCLE and the subset of the NCI60 cell lines considered here. Actually, the RMSE values for these predictions against the experimental data from the CCLE and, the pIC_50_ values from the CCLE and the pGI_50_ from the NCI60 dataset for these eight drugs and 44 cell-lines was comparable, namely 0.87 log_10_ units, indicating that high predictive power cannot be attained given the low concordance of the sensitivity data from these two datasets. These results come as no surprise, given the different surrogates of cytotoxicity exploited by the assays used to screen the CCLE and NCI60 panels, namely metabolic activity and protein abundance, respectively. Therefore, we conclude, in accord with Haibe-Kains *et al.* (2013), that models trained on the NCI60 data set are likely to fail on sensitivity data measured with a different experimental procedure, e.g. the CCLE or GDSC datasets.

Finally, although cultured cell lines and primary tumours differ genetically (Borrell, 2010) and 2D cell lines cultures do not recapitulate the complex tumour microenvironment, investigating the extent to which gene expression (and other cell line profiling) data can be used to model *in vitro* cell line sensitivity can help researchers to develop approaches for the prediction of clinical drug responses using the genomic data of tumour samples (Geeleher *et al.*, 2014).

## Supplementary Material

Supplementary Data
