# Supplementary material for: Improved large-scale prediction of growth inhibition patterns using the NCI60 cancer cell line panel
Source: Bioinformatics. 2015 Sep 8;32(1):85–95. doi: 10.1093/bioinformatics/btv529 (PMC4681992; doi:10.1093/bioinformatics/btv529)
Supplement: Supplementary Data [file supp_32_1_85__index.html]

Improved large-scale prediction of growth inhibition patterns using the NCI60 cancer cell line panel — Improved large-scale prediction of growth inhibition patterns using the NCI60 cancer cell line panel — Supplementary Data 

# Improved large-scale prediction of growth inhibition patterns using the NCI60 cancer cell line panel

## Supplementary Data

files

- Supplementary Data - pdf file
- Supplementary Data - pdf file
- Supplementary Data - xlsx file
