# Supplementary material for: Improved large-scale prediction of growth inhibition patterns using the NCI60 cancer cell line panel
Source: Bioinformatics. 2015 Sep 8;32(1):85–95. doi: 10.1093/bioinformatics/btv529 (PMC4681992; doi:10.1093/bioinformatics/btv529)

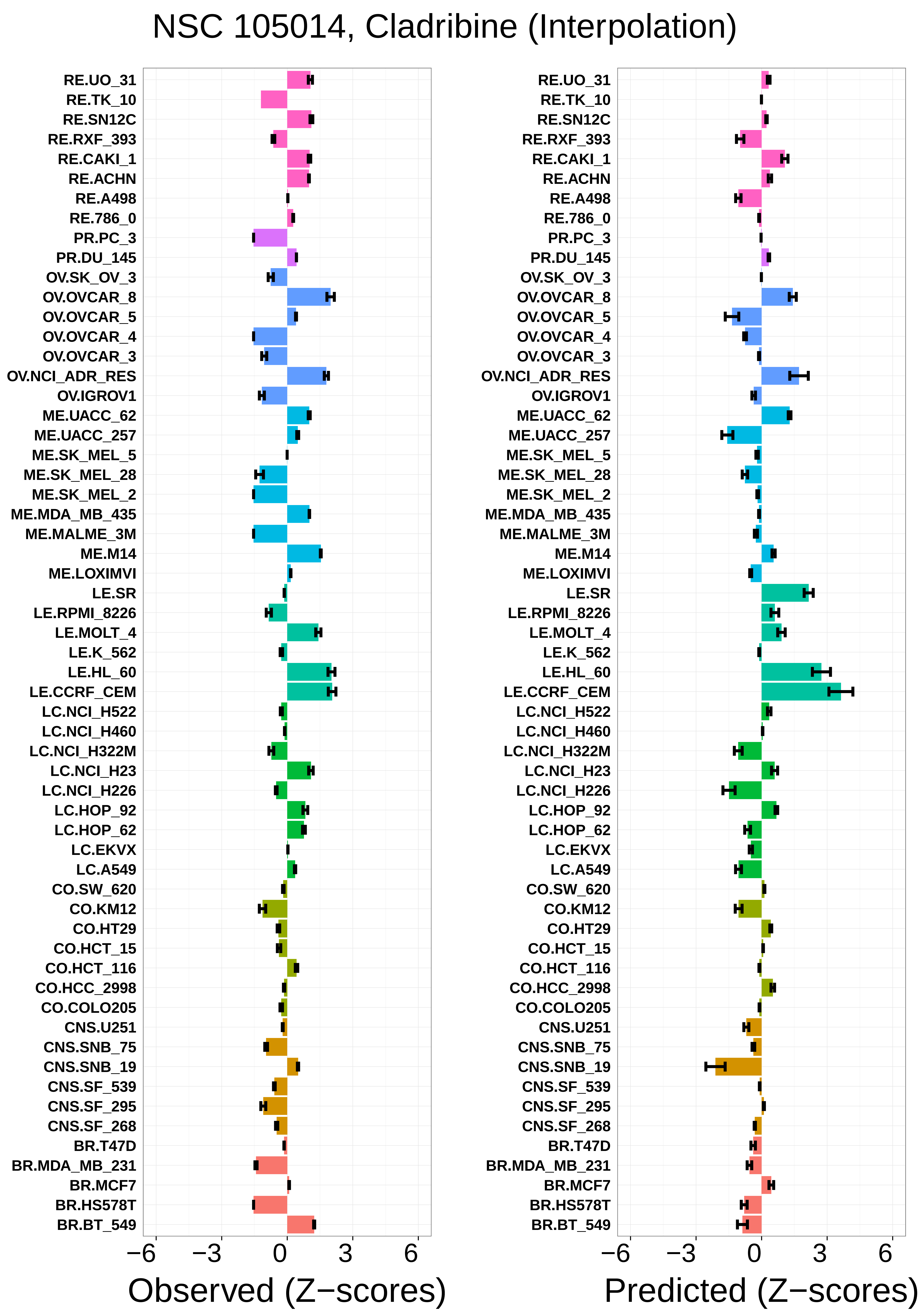
NSC 105014, Cladribine (Interpolation)
RE.UO_31
RE.TK_10
RE.SN12C
RE.RXF_393
RE.CAKI_1
RE.ACHN
RE.A498
RE.786_0
PR.PC_3
PR.DU_145
OV.SK_OV_3
OV.OVCAR_8
OV.OVCAR_5
OV.OVCAR_4
OV.OVCAR_3
OV.NCI_ADR_RES
OV.IGROV1
ME.UACC_62
ME.UACC_257
ME.SK_MEL_5
ME.SK_MEL_28
ME.SK_MEL_2
ME.MDA_MB_435
ME.MALME_3M
ME.M14
ME.LOXIMVI
LE.SR
LE.RPMI_8226
LE.MOLT_4
LE.K_562
LE.HL_60
LE.CCRF_CEM
LC.NCI_H522
LC.NCI_H460
LC.NCI_H322M
LC.NCI_H23
LC.NCI_H226
LC.HOP_92
LC.HOP_62
LC.EKVX
LC.A549
CO.SW_620
CO.KM12
CO.HT29
CO.HCT_15
CO.HCT_116
CO.HCC_2998
CO.COLO205
CNS.U251
CNS.SNB_75
CNS.SNB_19
CNS.SF_539
CNS.SF_295
CNS.SF_268
BR.T47D
BR.MDA_MB_231
BR.MCF7
BR.HS578T
BR.BT_549
−6
−3
0
3
6
Observed (Z−scores)
Predicted (Z−scores)

NSC 118218, Fludarabine (Interpolation)

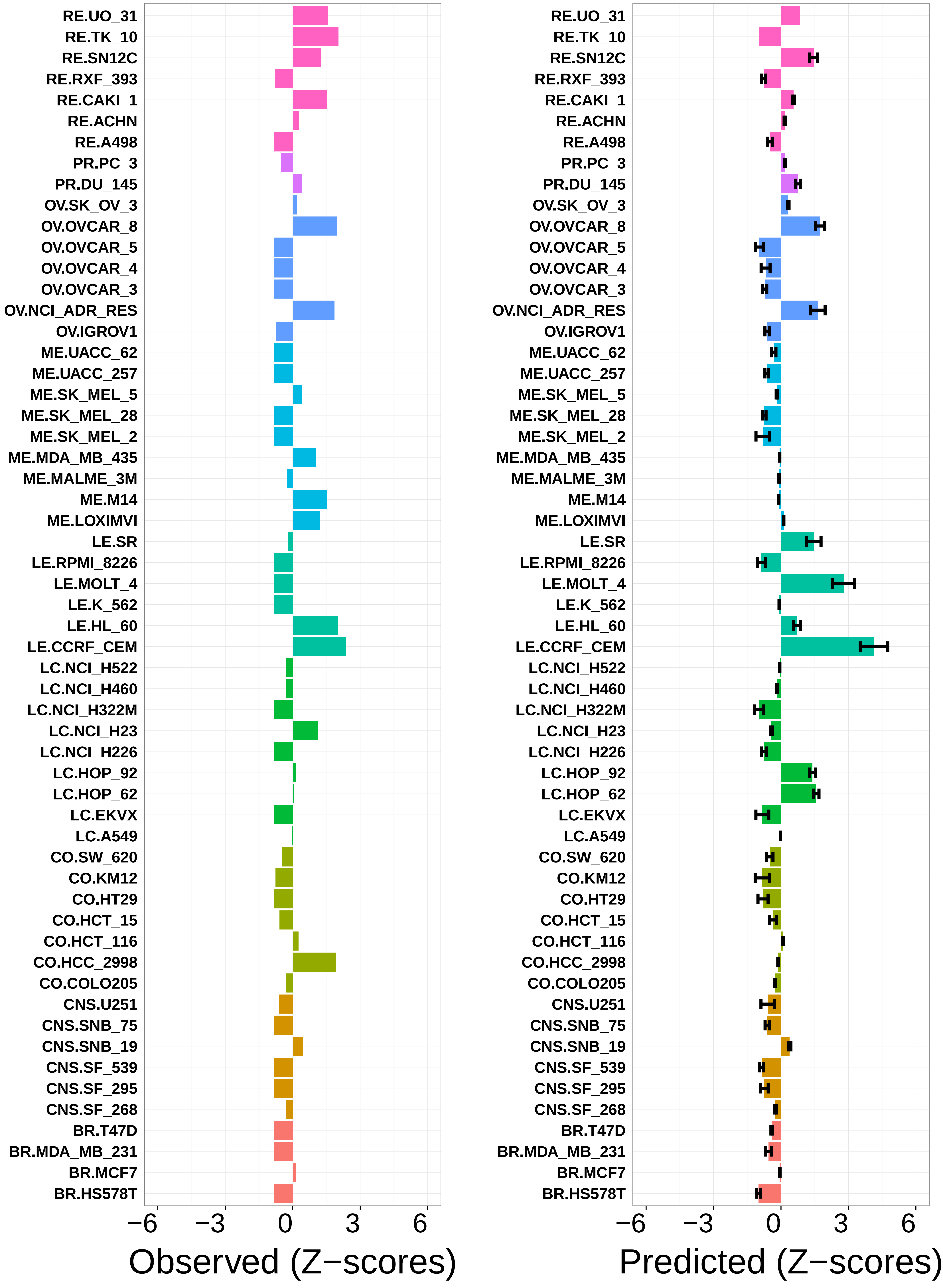

NSC 122819, Teniposide (Interpolation)

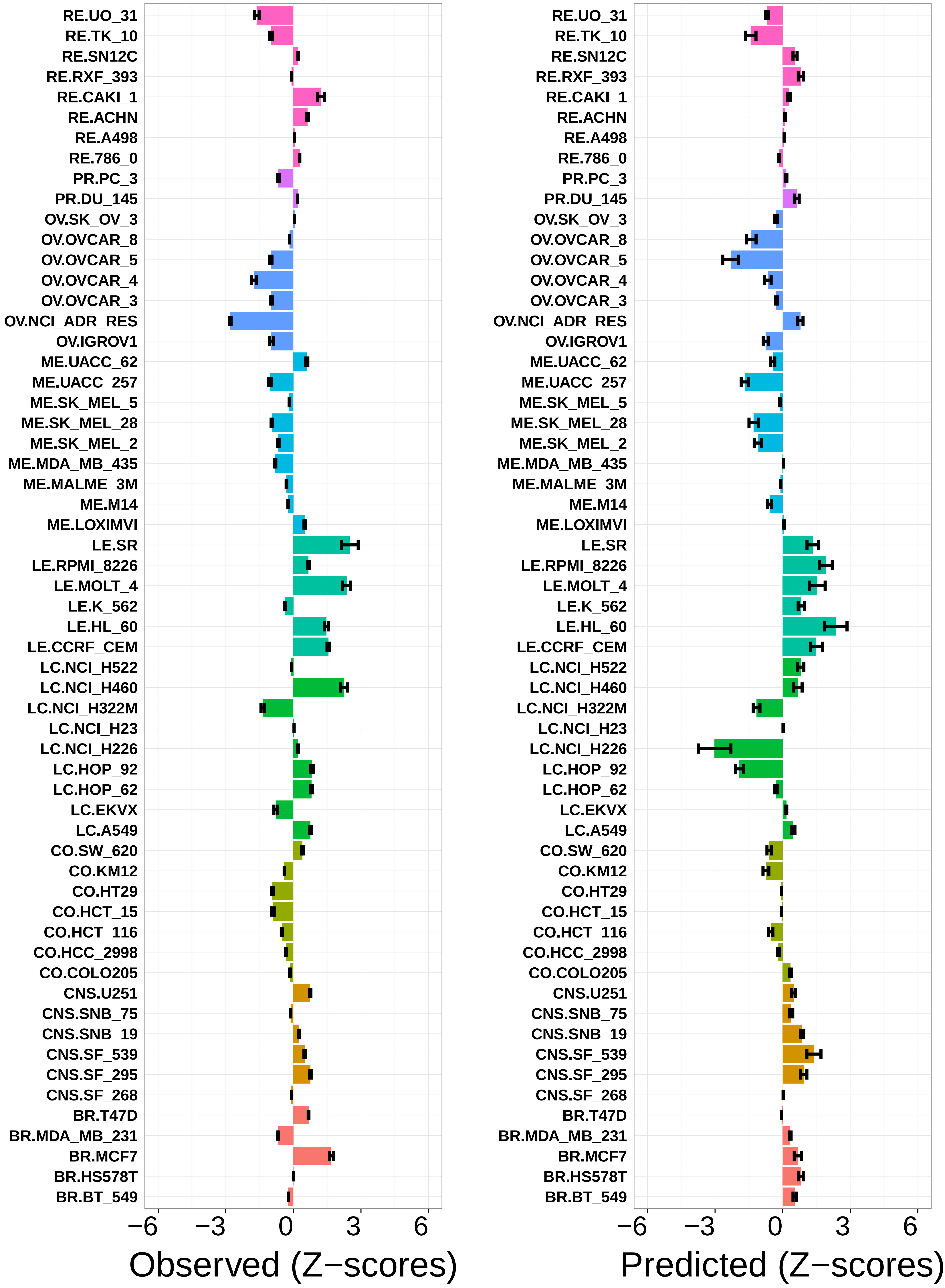

NSC 123127, Doxorubicin_Hydrochloride (Interpolation)

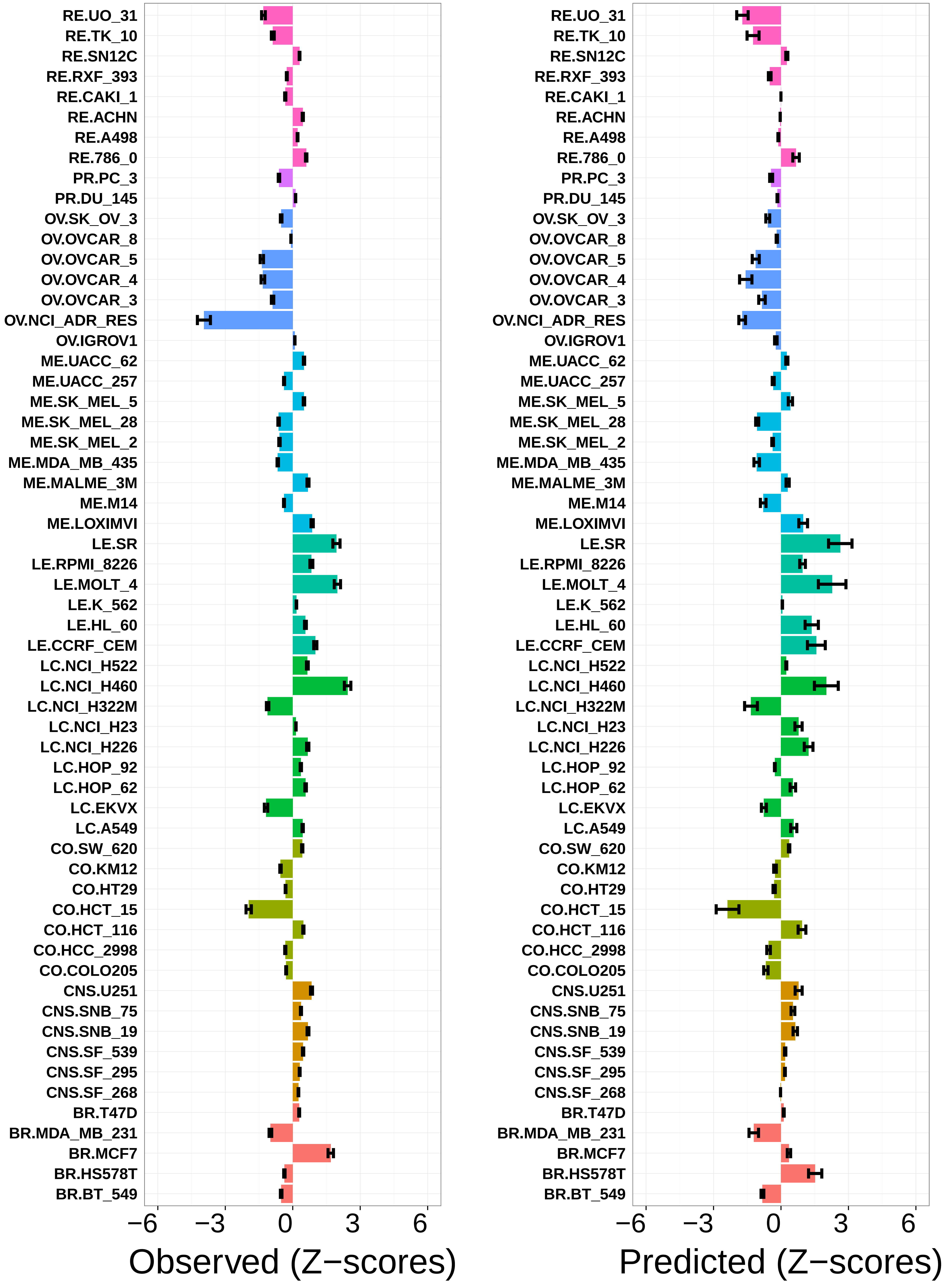

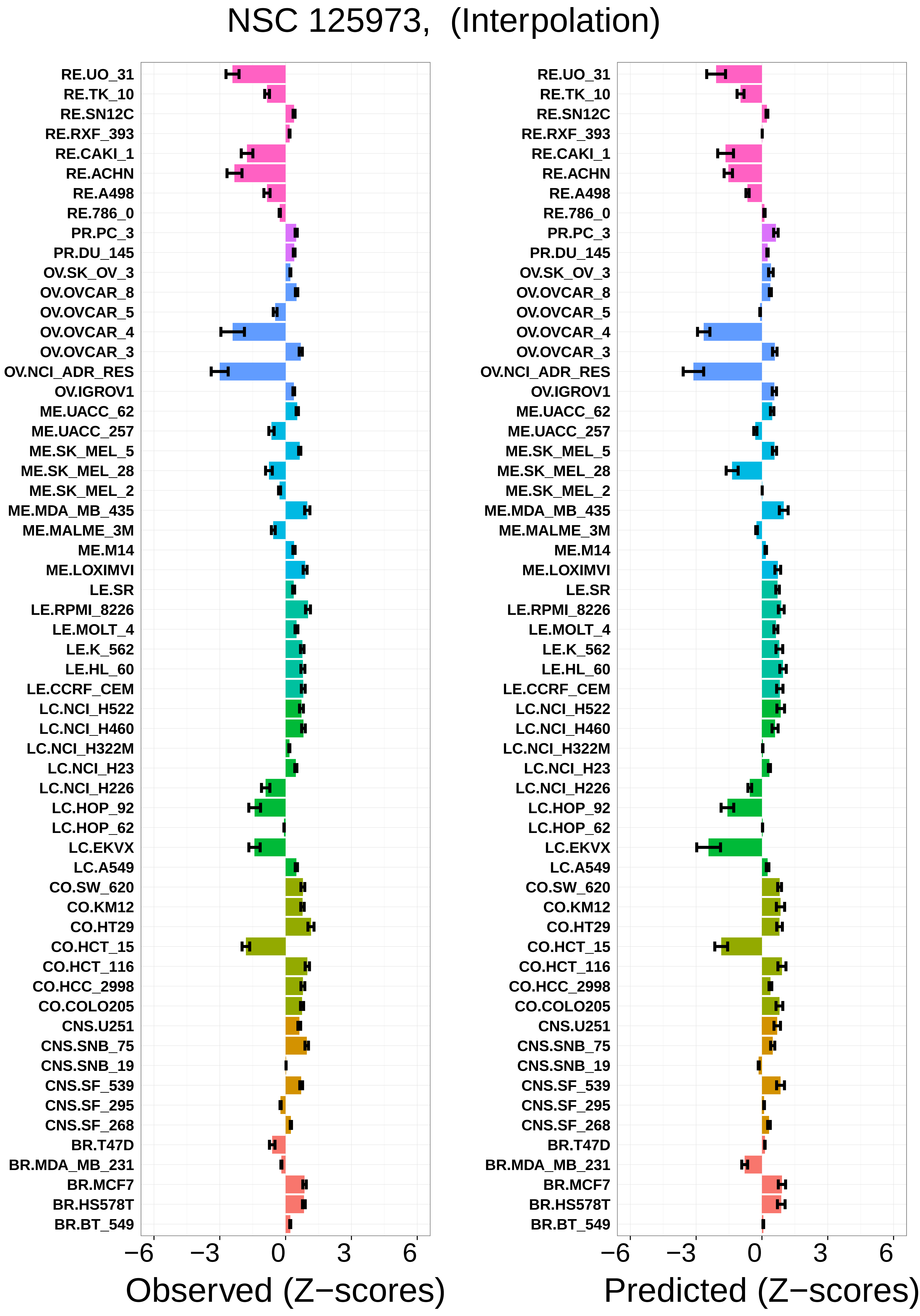
NSC 125973, (Interpolation)
RE.UO_31
RE.TK_10
RE.SN12C
RE.RXF_393
RE.CAKI_1
RE.ACHN
RE.A498
RE.786_0
PR.PC_3
PR.DU_145
OV.SK_OV_3
OV.OVCAR_8
OV.OVCAR_5
OV.OVCAR_4
OV.OVCAR_3
OV.NCI_ADR_RES
OV.IGROV1
ME.UACC_62
ME.UACC_257
ME.SK_MEL_5
ME.SK_MEL_28
ME.SK_MEL_2
ME.MDA_MB_435
ME.MALME_3M
ME.M14
ME.LOXIMVI
LE.SR
LE.RPMI_8226
LE.MOLT_4
LE.K_562
LE.HL_60
LE.CCRF_CEM
LC.NCI_H522
LC.NCI_H460
LC.NCI_H322M
LC.NCI_H23
LC.NCI_H226
LC.HOP_92
LC.HOP_62
LC.EKVX
LC.A549
CO.SW_620
CO.KM12
CO.HT29
CO.HCT_15
CO.HCT_116
CO.HCC_2998
CO.COLO205
CNS.U251
CNS.SNB_75
CNS.SNB_19
CNS.SF_539
CNS.SF_295
CNS.SF_268
BR.T47D
BR.MDA_MB_231
BR.MCF7
BR.HS578T
BR.BT_549
−6
−3
0
3
6
Observed (Z−scores)
Predicted (Z−scores)

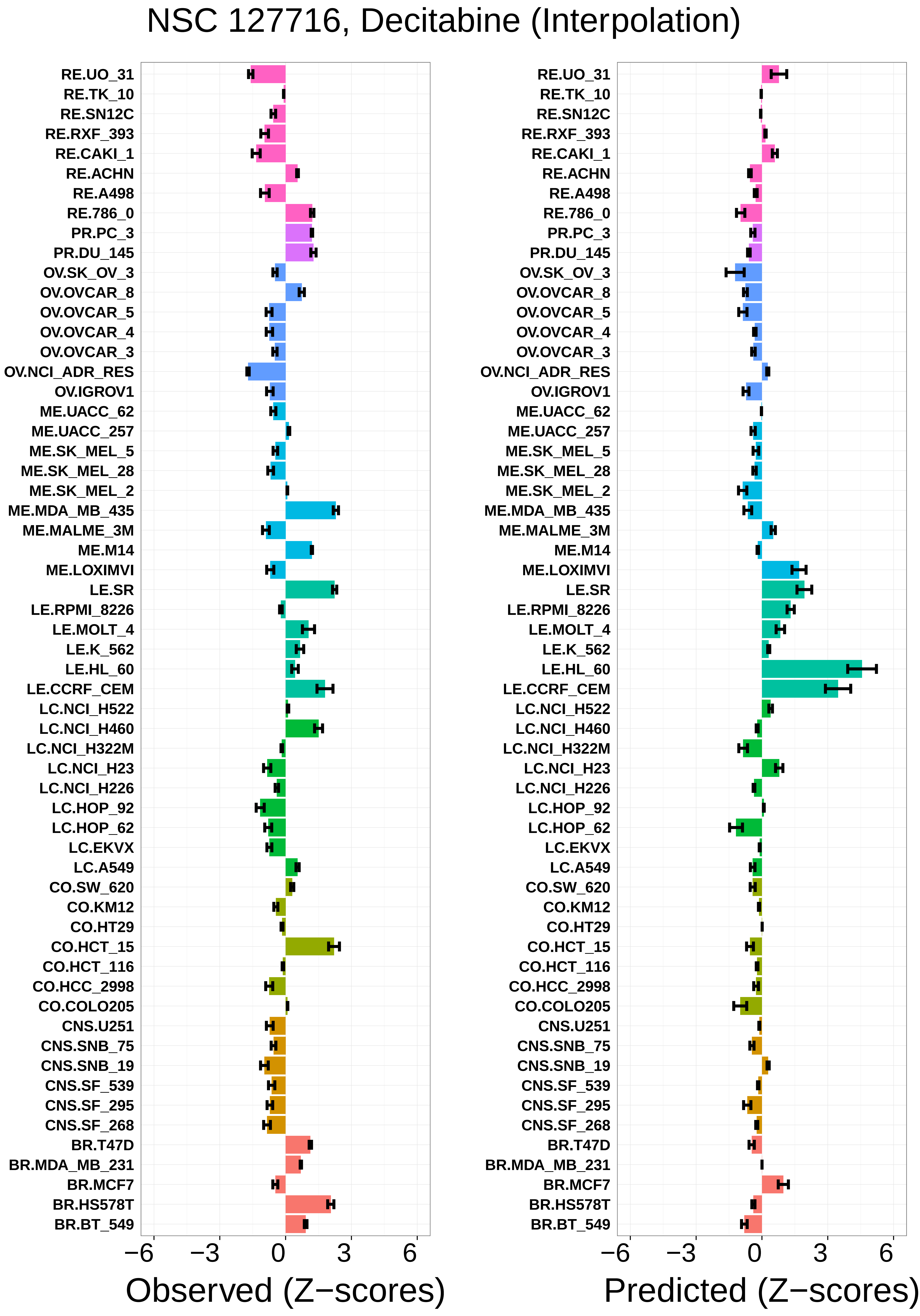
NSC 127716, Decitabine (Interpolation)
RE.UO_31
RE.TK_10
RE.SN12C
RE.RXF_393
RE.CAKI_1
RE.ACHN
RE.A498
RE.786_0
PR.PC_3
PR.DU_145
OV.SK_OV_3
OV.OVCAR_8
OV.OVCAR_5
OV.OVCAR_4
OV.OVCAR_3
OV.NCI_ADR_RES
OV.IGROV1
ME.UACC_62
ME.UACC_257
ME.SK_MEL_5
ME.SK_MEL_28
ME.SK_MEL_2
ME.MDA_MB_435
ME.MALME_3M
ME.M14
ME.LOXIMVI
LE.SR
LE.RPMI_8226
LE.MOLT_4
LE.K_562
LE.HL_60
LE.CCRF_CEM
LC.NCI_H522
LC.NCI_H460
LC.NCI_H322M
LC.NCI_H23
LC.NCI_H226
LC.HOP_92
LC.HOP_62
LC.EKVX
LC.A549
CO.SW_620
CO.KM12
CO.HT29
CO.HCT_15
CO.HCT_116
CO.HCC_2998
CO.COLO205
CNS.U251
CNS.SNB_75
CNS.SNB_19
CNS.SF_539
CNS.SF_295
CNS.SF_268
BR.T47D
BR.MDA_MB_231
BR.MCF7
BR.HS578T
BR.BT_549
−6 −3 0 3 6
Observed (Z−scores)
Predicted (Z−scores)

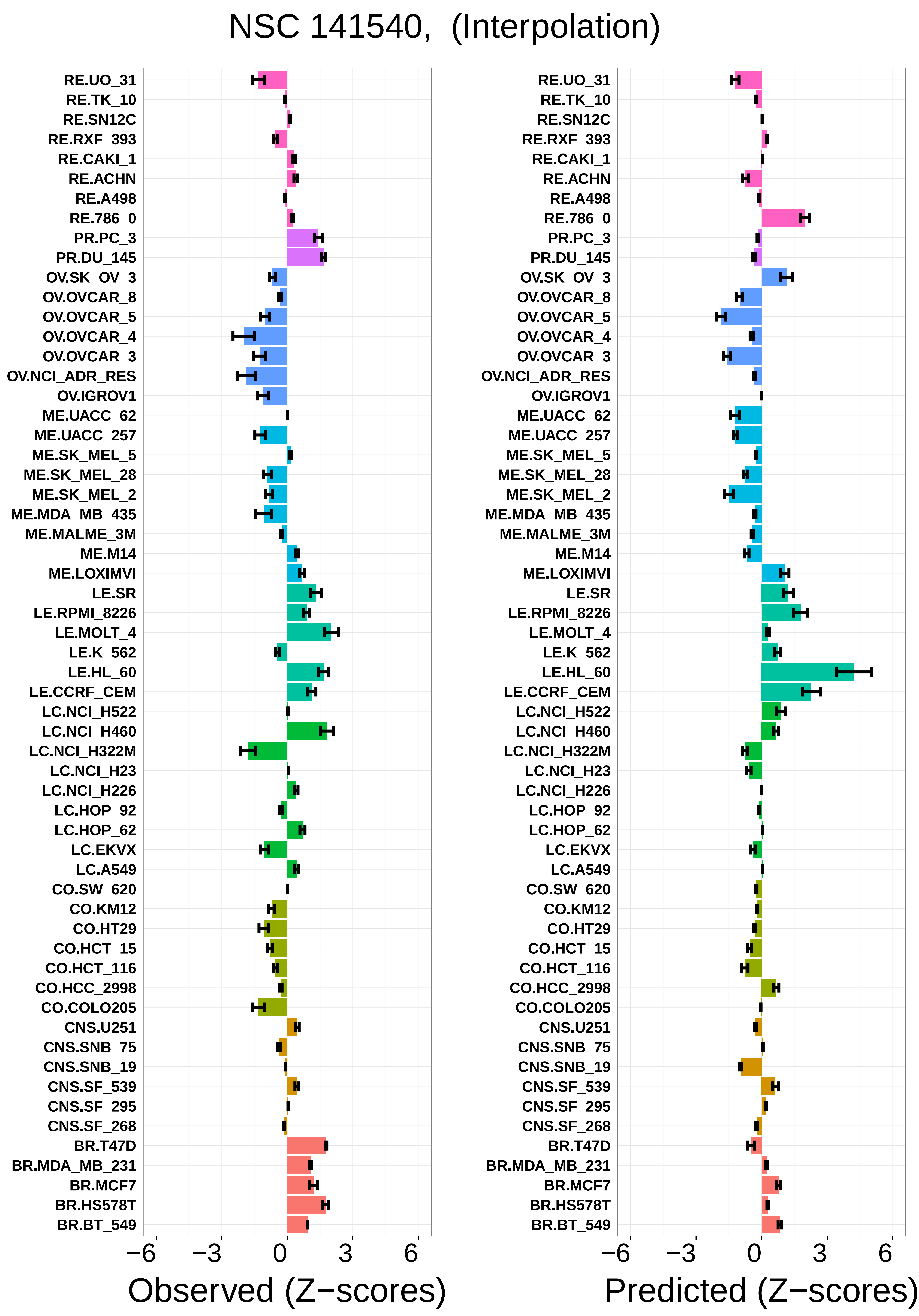
NSC 141540, (Interpolation)
RE.UO_31
RE.TK_10
RE.SN12C
RE.RXF_393
RE.CAKI_1
RE.ACHN
RE.A498
RE.786_0
PR.PC_3
PR.DU_145
OV.SK_OV_3
OV.OVCAR_8
OV.OVCAR_5
OV.OVCAR_4
OV.OVCAR_3
OV.NCI_ADR_RES
OV.IGROV1
ME.UACC_62
ME.UACC_257
ME.SK_MEL_5
ME.SK_MEL_28
ME.SK_MEL_2
ME.MDA_MB_435
ME.MALME_3M
ME.M14
ME.LOXIMVI
LE.SR
LE.RPMI_8226
LE.MOLT_4
LE.K_562
LE.HL_60
LE.CCRF_CEM
LC.NCI_H522
LC.NCI_H460
LC.NCI_H322M
LC.NCI_H23
LC.NCI_H226
LC.HOP_92
LC.HOP_62
LC.EKVX
LC.A549
CO.SW_620
CO.KM12
CO.HT29
CO.HCT_15
CO.HCT_116
CO.HCC_2998
CO.COLO205
CNS.U251
CNS.SNB_75
CNS.SNB_19
CNS.SF_539
CNS.SF_295
CNS.SF_268
BR.T47D
BR.MDA_MB_231
BR.MCF7
BR.HS578T
BR.BT_549
−6
−3
0
3
6
Observed (Z−scores)
Predicted (Z−scores)

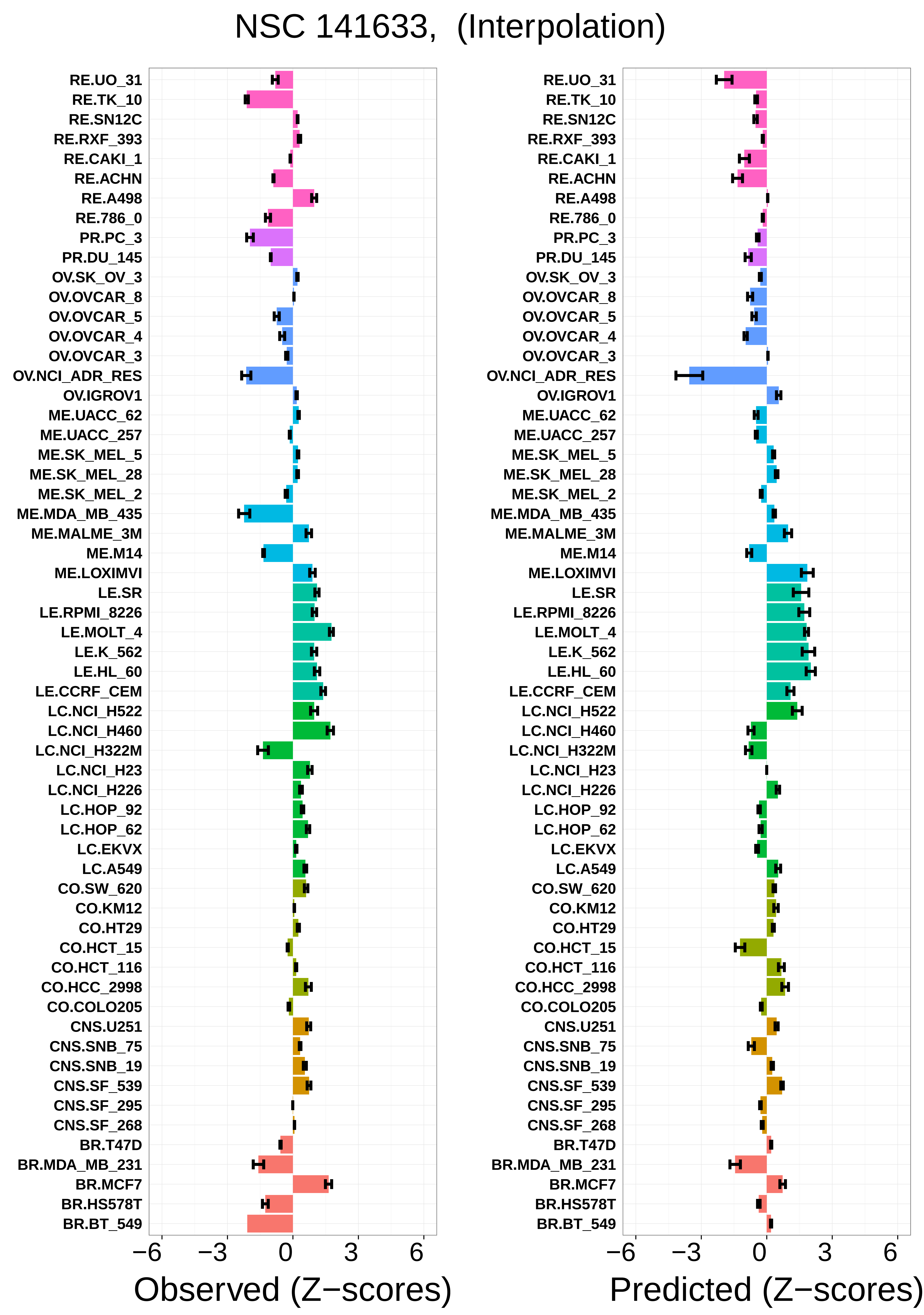
NSC 141633, (Interpolation)
RE.UO_31
RE.TK_10
RE.SN12C
RE.RXF_393
RE.CAKI_1
RE.ACHN
RE.A498
RE.786_0
PR.PC_3
PR.DU_145
OV.SK_OV_3
OV.OVCAR_8
OV.OVCAR_5
OV.OVCAR_4
OV.OVCAR_3
OV.NCI_ADR_RES
OV.IGROV1
ME.UACC_62
ME.UACC_257
ME.SK_MEL_5
ME.SK_MEL_28
ME.SK_MEL_2
ME.MDA_MB_435
ME.MALME_3M
ME.M14
ME.LOXIMVI
LE.SR
LE.RPMI_8226
LE.MOLT_4
LE.K_562
LE.HL_60
LE.CCRF_CEM
LC.NCI_H522
LC.NCI_H460
LC.NCI_H322M
LC.NCI_H23
LC.NCI_H226
LC.HOP_92
LC.HOP_62
LC.EKVX
LC.A549
CO.SW_620
CO.KM12
CO.HT29
CO.HCT_15
CO.HCT_116
CO.HCC_2998
CO.COLO205
CNS.U251
CNS.SNB_75
CNS.SNB_19
CNS.SF_539
CNS.SF_295
CNS.SF_268
BR.T47D
BR.MDA_MB_231
BR.MCF7
BR.HS578T
BR.BT_549
−6 −3 0 3 6
Observed (Z−scores)
Predicted (Z−scores)

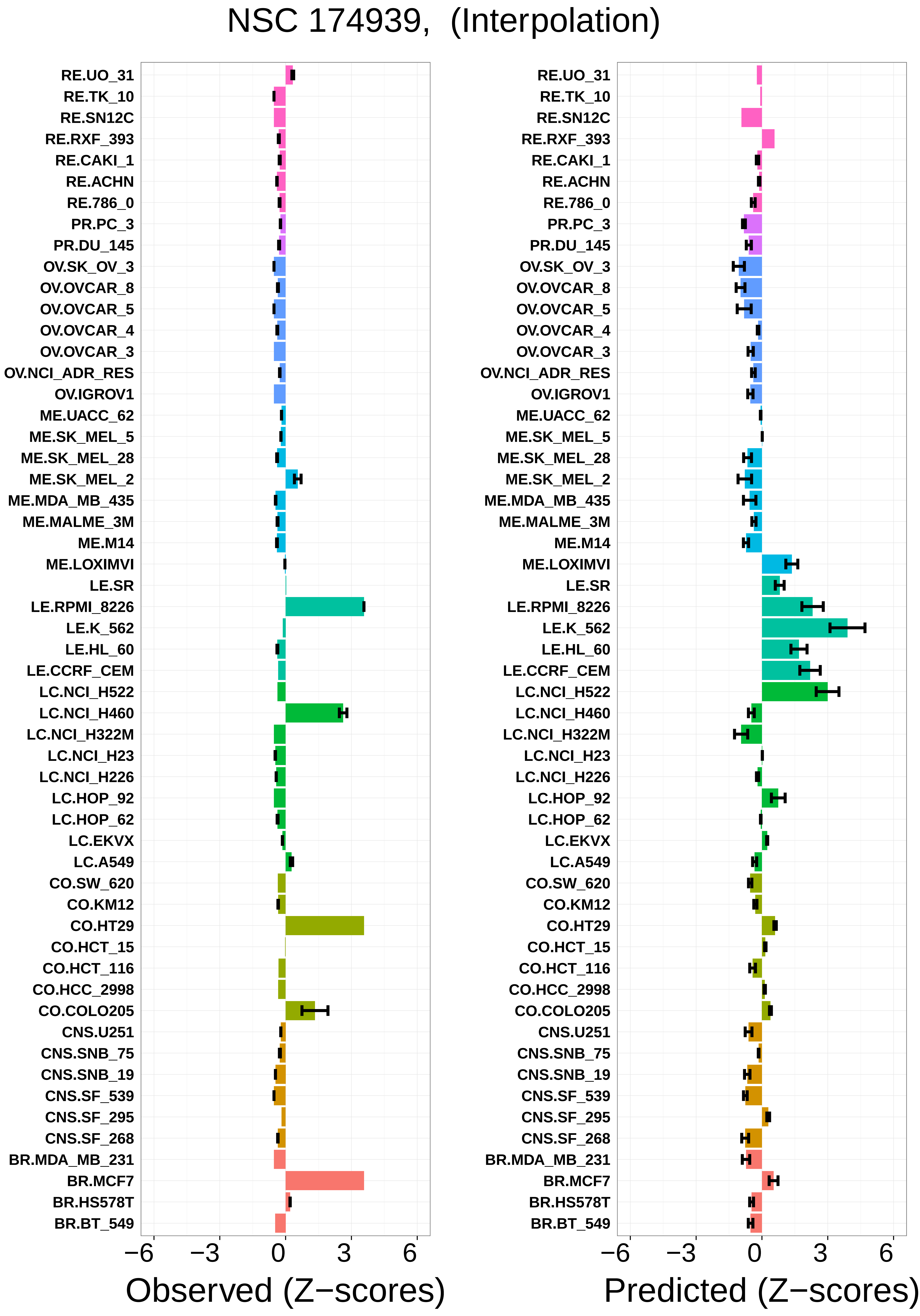
NSC 174939, (Interpolation)
RE.UO_31
RE.TK_10
RE.SN12C
RE.RXF_393
RE.CAKI_1
RE.ACHN
RE.786_0
PR.PC_3
PR.DU_145
OV.SK_OV_3
OV.OVCAR_8
OV.OVCAR_5
OV.OVCAR_4
OV.OVCAR_3
OV.NCI_ADR_RES
OV.IGROV1
ME.UACC_62
ME.SK_MEL_5
ME.SK_MEL_28
ME.SK_MEL_2
ME.MDA_MB_435
ME.MALME_3M
ME.M14
ME.LOXIMVI
LE.SR
LE.RPMI_8226
LE.K_562
LE.HL_60
LE.CCRF_CEM
LC.NCI_H522
LC.NCI_H460
LC.NCI_H322M
LC.NCI_H23
LC.NCI_H226
LC.HOP_92
LC.HOP_62
LC.EKVX
LC.A549
CO.SW_620
CO.KM12
CO.HT29
CO.HCT_15
CO.HCT_116
CO.HCC_2998
CO.COLO205
CNS.U251
CNS.SNB_75
CNS.SNB_19
CNS.SF_539
CNS.SF_295
CNS.SF_268
BR.MDA_MB_231
BR.MCF7
BR.HS578T
BR.BT_549
−6 −3 0 3 6
Observed (Z−scores)
Predicted (Z−scores)

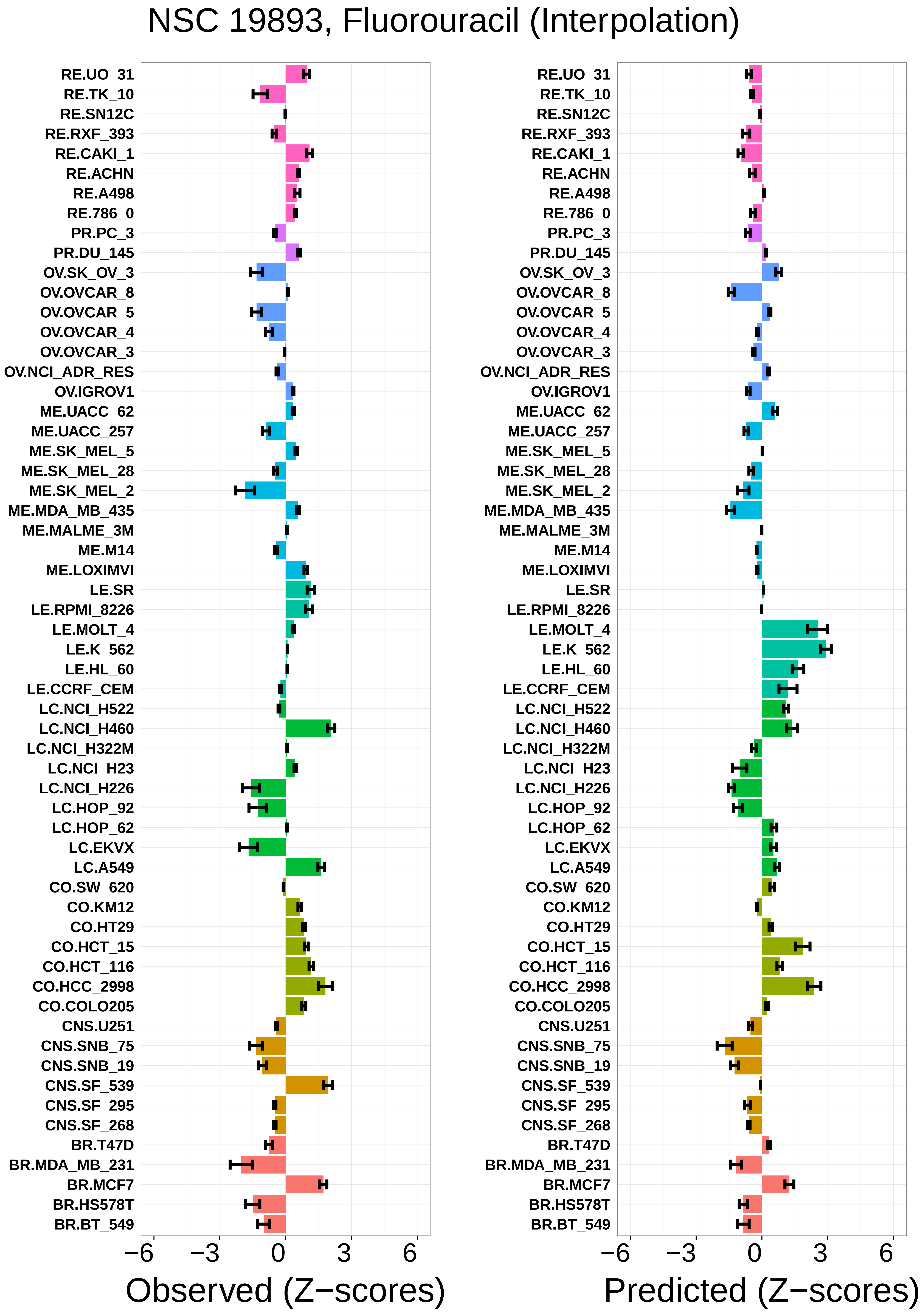

NSC 19893, Fluorouracil (Interpolation)
RE.UO_31
RE.TK_10
RE.SN12C
RE.RXF_393
RE.CAKI_1
RE.ACHN
RE.A498
RE.786_0
PR.PC_3
PR.DU_145
OV.SK_OV_3
OV.OVCAR_8
OV.OVCAR_5
OV.OVCAR_4
OV.OVCAR_3
OV.NCI_ADR_RES
OV.IGROV1
ME.UACC_62
ME.UACC_257
ME.SK_MEL_5
ME.SK_MEL_28
ME.SK_MEL_2
ME.MDA_MB_435
ME.MALME_3M
ME.M14
ME.LOXIMVI
LE.SR
LE.RPMI_8226
LE.MOLT_4
LE.K_562
LE.HL_60
LE.CCRF_CEM
LC.NCI_H522
LC.NCI_H460
LC.NCI_H322M
LC.NCI_H23
LC.NCI_H226
LC.HOP_92
LC.HOP_62
LC.EKVX
LC.A549
CO.SW_620
CO.KM12
CO.HT29
CO.HCT_15
CO.HCT_116
CO.HCC_2998
CO.COLO205
CNS.U251
CNS.SNB_75
CNS.SNB_19
CNS.SF_539
CNS.SF_295
CNS.SF_268
BR.T47D
BR.MDA_MB_231
BR.MCF7
BR.HS578T
BR.BT_549
−6
−3
0
3
6
Observed (Z−scores)
Predicted (Z−scores)

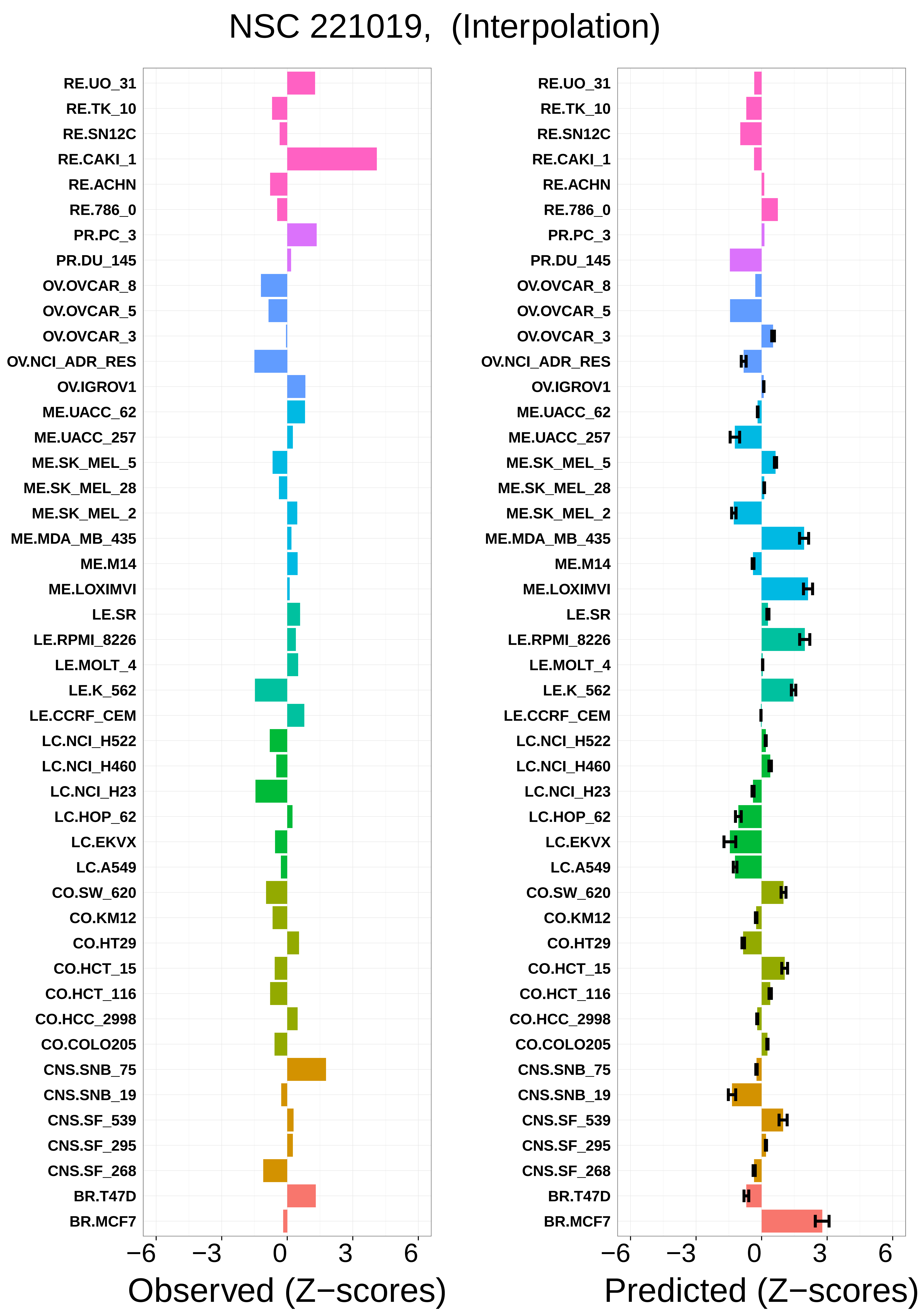
NSC 221019, (Interpolation)
RE.UO_31
RE.TK_10
RE.SN12C
RE.CAKI_1
RE.ACHN
RE.786_0
PR.PC_3
PR.DU_145
OV.OVCAR_8
OV.OVCAR_5
OV.OVCAR_3
OV.NCI_ADR_RES
OV.IGROV1
ME.UACC_62
ME.UACC_257
ME.SK_MEL_5
ME.SK_MEL_28
ME.SK_MEL_2
ME.MDA_MB_435
ME.M14
ME.LOXIMVI
LE.SR
LE.RPMI_8226
LE.MOLT_4
LE.K_562
LE.CCRF_CEM
LC.NCI_H522
LC.NCI_H460
LC.NCI_H23
LC.HOP_62
LC.EKVX
LC.A549
CO.SW_620
CO.KM12
CO.HT29
CO.HCT_15
CO.HCT_116
CO.HCC_2998
CO.COLO205
CNS.SNB_75
CNS.SNB_19
CNS.SF_539
CNS.SF_295
CNS.SF_268
BR.T47D
BR.MCF7
−6 −3 0 3 6
Observed (Z−scores)
Predicted (Z−scores)

NSC 246131, (Interpolation)

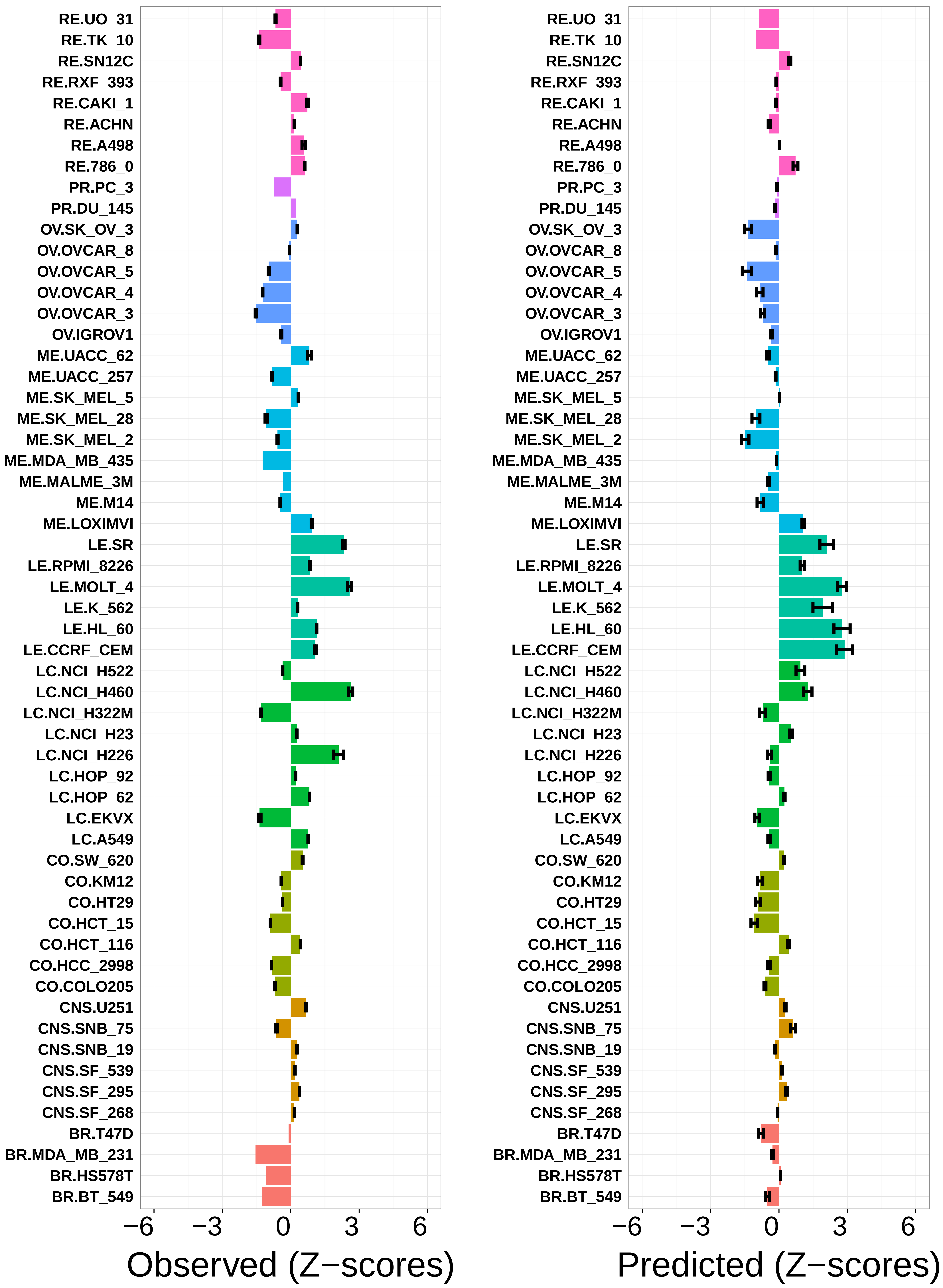

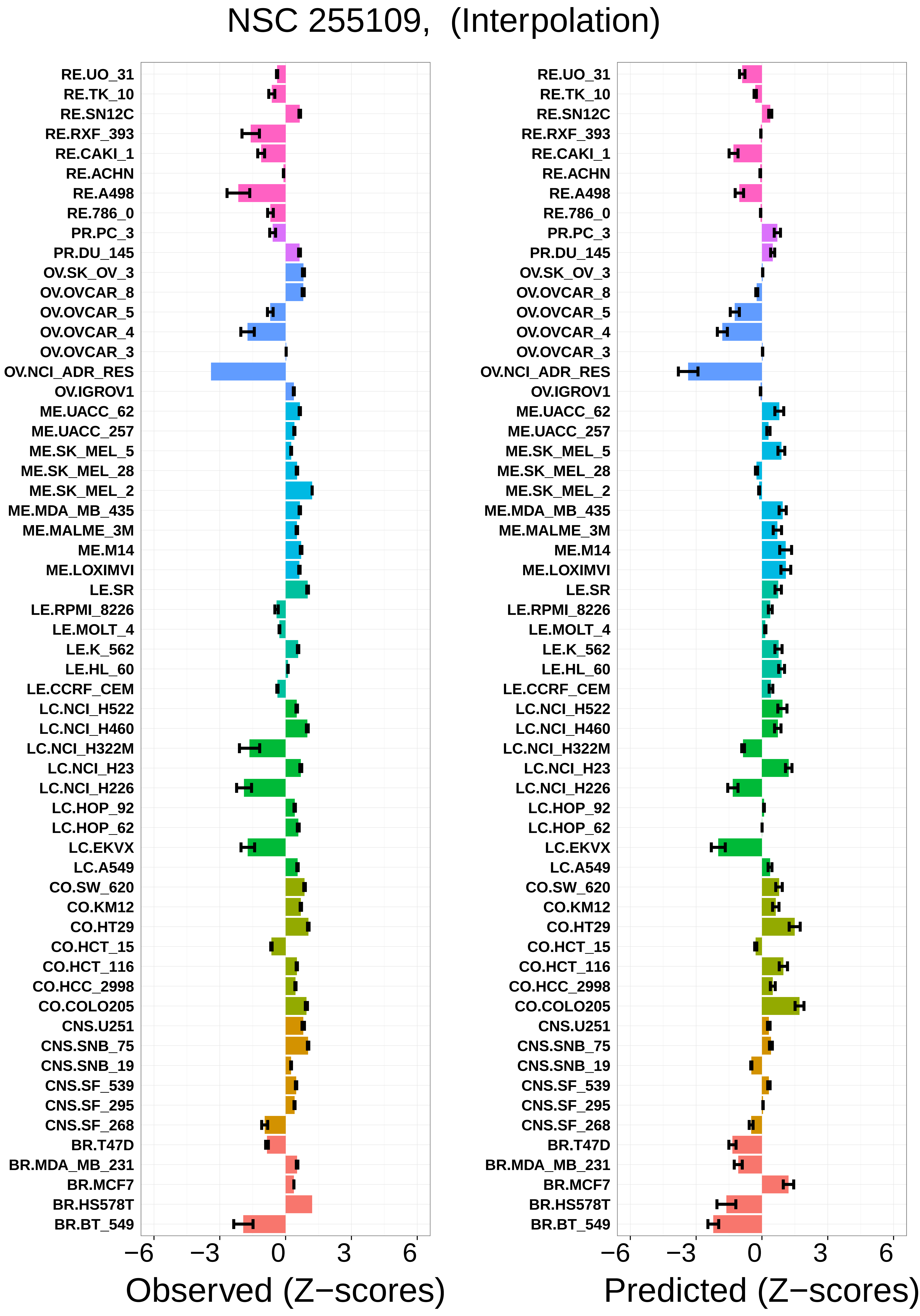
NSC 255109, (Interpolation)
RE.UO_31
RE.TK_10
RE.SN12C
RE.RXF_393
RE.CAKI_1
RE.ACHN
RE.A498
RE.786_0
PR.PC_3
PR.DU_145
OV.SK_OV_3
OV.OVCAR_8
OV.OVCAR_5
OV.OVCAR_4
OV.OVCAR_3
OV.NCI_ADR_RES
OV.IGROV1
ME.UACC_62
ME.UACC_257
ME.SK_MEL_5
ME.SK_MEL_28
ME.SK_MEL_2
ME.MDA_MB_435
ME.MALME_3M
ME.M14
ME.LOXIMVI
LE.SR
LE.RPMI_8226
LE.MOLT_4
LE.K_562
LE.HL_60
LE.CCRF_CEM
LC.NCI_H522
LC.NCI_H460
LC.NCI_H322M
LC.NCI_H23
LC.NCI_H226
LC.HOP_92
LC.HOP_62
LC.EKVX
LC.A549
CO.SW_620
CO.KM12
CO.HT29
CO.HCT_15
CO.HCT_116
CO.HCC_2998
CO.COLO205
CNS.U251
CNS.SNB_75
CNS.SNB_19
CNS.SF_539
CNS.SF_295
CNS.SF_268
BR.T47D
BR.MDA_MB_231
BR.MCF7
BR.HS578T
BR.BT_549
−6 −3 0 3 6
Observed (Z−scores)
Predicted (Z−scores)

NSC 256942, (Interpolation)

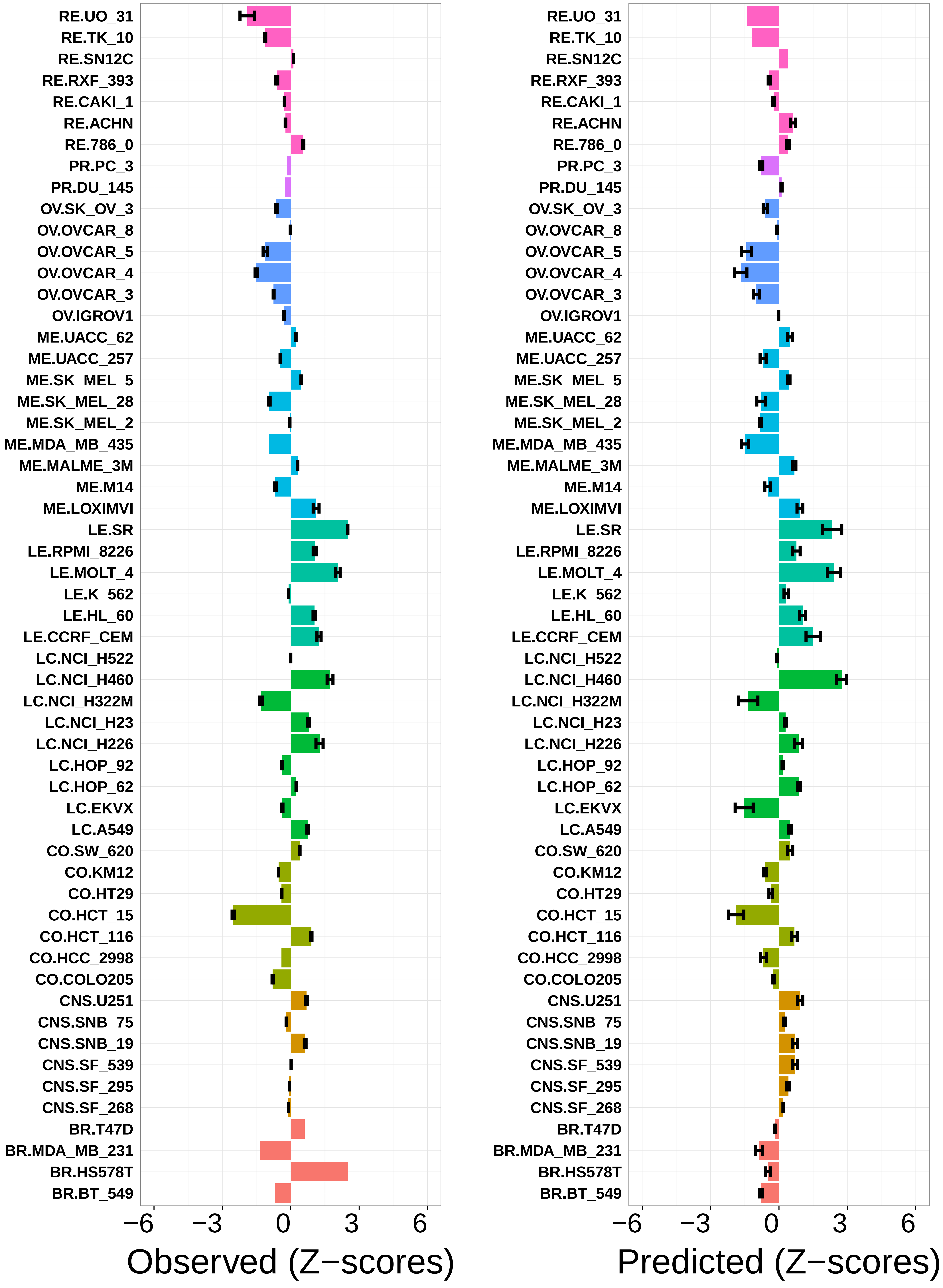

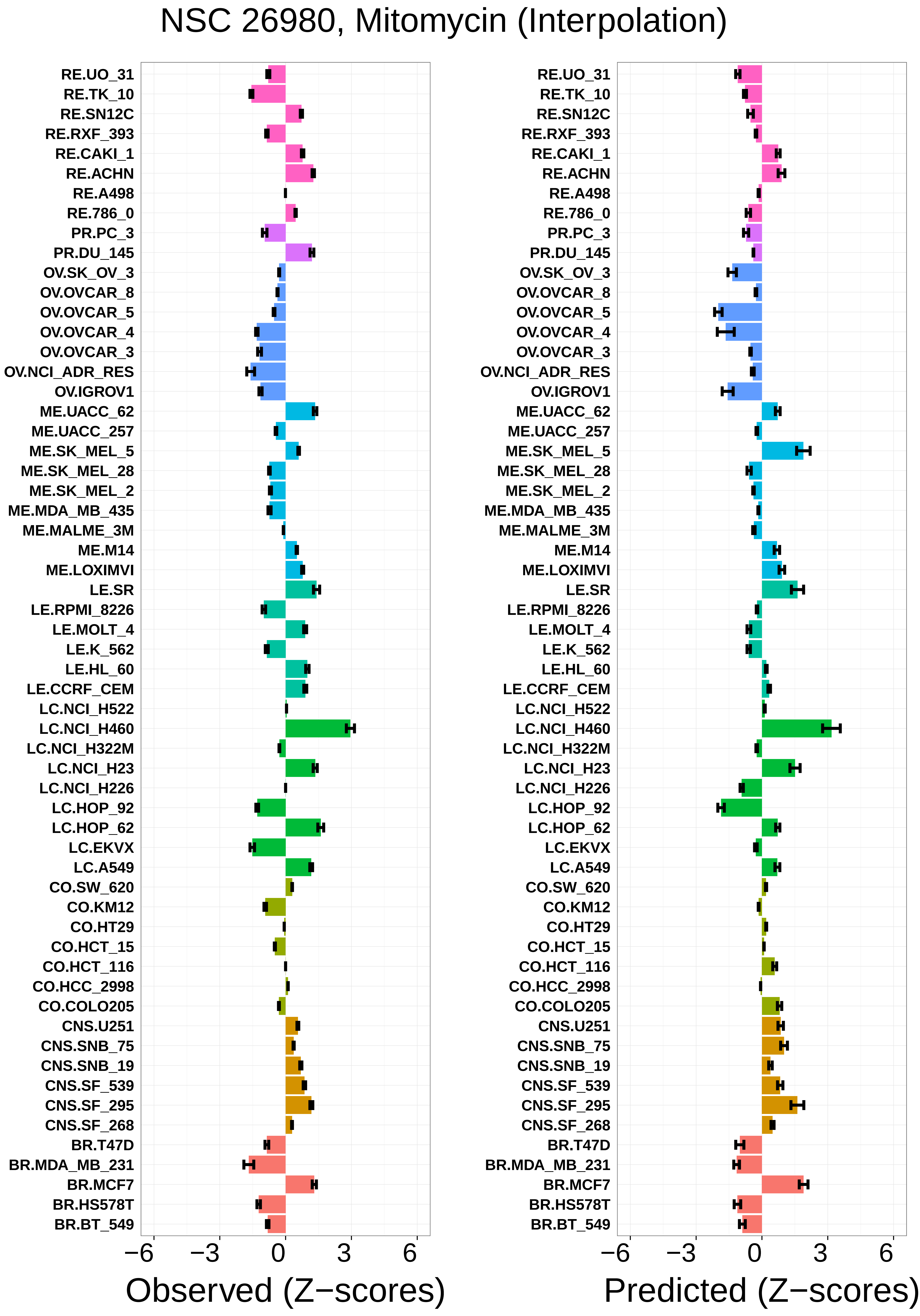
NSC 26980, Mitomycin (Interpolation)
RE.UO_31
RE.TK_10
RE.SN12C
RE.RXF_393
RE.CAKI_1
RE.ACHN
RE.A498
RE.786_0
PR.PC_3
PR.DU_145
OV.SK_OV_3
OV.OVCAR_8
OV.OVCAR_5
OV.OVCAR_4
OV.OVCAR_3
OV.NCI_ADR_RES
OV.IGROV1
ME.UACC_62
ME.UACC_257
ME.SK_MEL_5
ME.SK_MEL_28
ME.SK_MEL_2
ME.MDA_MB_435
ME.MALME_3M
ME.M14
ME.LOXIMVI
LE.SR
LE.RPMI_8226
LE.MOLT_4
LE.K_562
LE.HL_60
LE.CCRF_CEM
LC.NCI_H522
LC.NCI_H460
LC.NCI_H322M
LC.NCI_H23
LC.NCI_H226
LC.HOP_92
LC.HOP_62
LC.EKVX
LC.A549
CO.SW_620
CO.KM12
CO.HT29
CO.HCT_15
CO.HCT_116
CO.HCC_2998
CO.COLO205
CNS.U251
CNS.SNB_75
CNS.SNB_19
CNS.SF_539
CNS.SF_295
CNS.SF_268
BR.T47D
BR.MDA_MB_231
BR.MCF7
BR.HS578T
BR.BT_549
−6
−3
0
3
6
Observed (Z−scores)
Predicted (Z−scores)

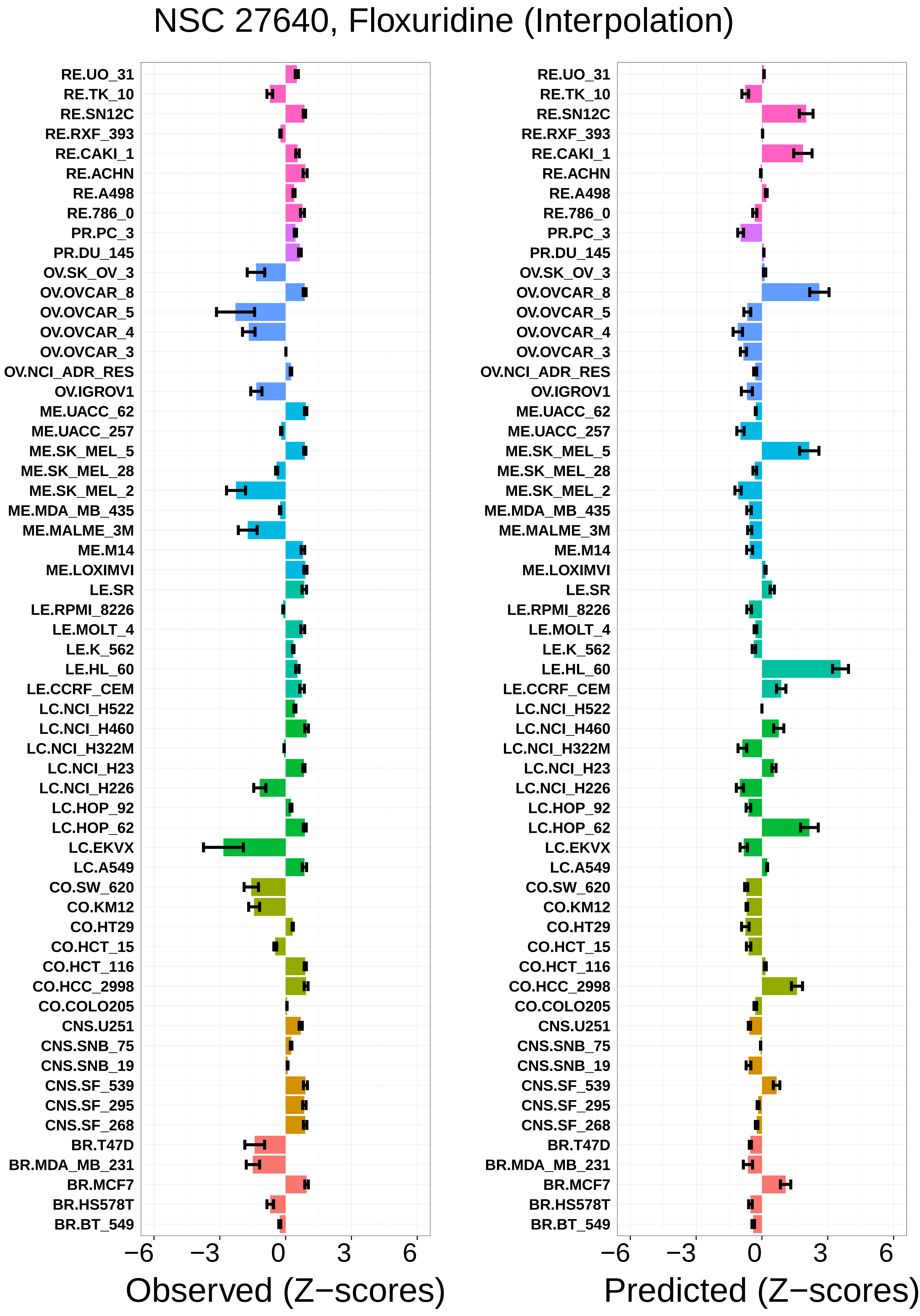
NSC 27640, Floxuridine (Interpolation)
RE.UO_31
RE.TK_10
RE.SN12C
RE.RXF_393
RE.CAKI_1
RE.ACHN
RE.A498
RE.786_0
PR.PC_3
PR.DU_145
OV.SK_OV_3
OV.OVCAR_8
OV.OVCAR_5
OV.OVCAR_4
OV.OVCAR_3
OV.NCI_ADR_RES
OV.IGROV1
ME.UACC_62
ME.UACC_257
ME.SK_MEL_5
ME.SK_MEL_28
ME.SK_MEL_2
ME.MDA_MB_435
ME.MALME_3M
ME.M14
ME.LOXIMVI
LE.SR
LE.RPMI_8226
LE.MOLT_4
LE.K_562
LE.HL_60
LE.CCRF_CEM
LC.NCI_H522
LC.NCI_H460
LC.NCI_H322M
LC.NCI_H23
LC.NCI_H226
LC.HOP_92
LC.HOP_62
LC.EKVX
LC.A549
CO.SW_620
CO.KM12
CO.HT29
CO.HCT_15
CO.HCT_116
CO.HCC_2998
CO.COLO205
CNS.U251
CNS.SNB_75
CNS.SNB_19
CNS.SF_539
CNS.SF_295
CNS.SF_268
BR.T47D
BR.MDA_MB_231
BR.MCF7
BR.HS578T
BR.BT_549
−6 −3 0 3 6
Observed (Z−scores)
−6 −3 0 3 6
Predicted (Z−scores)

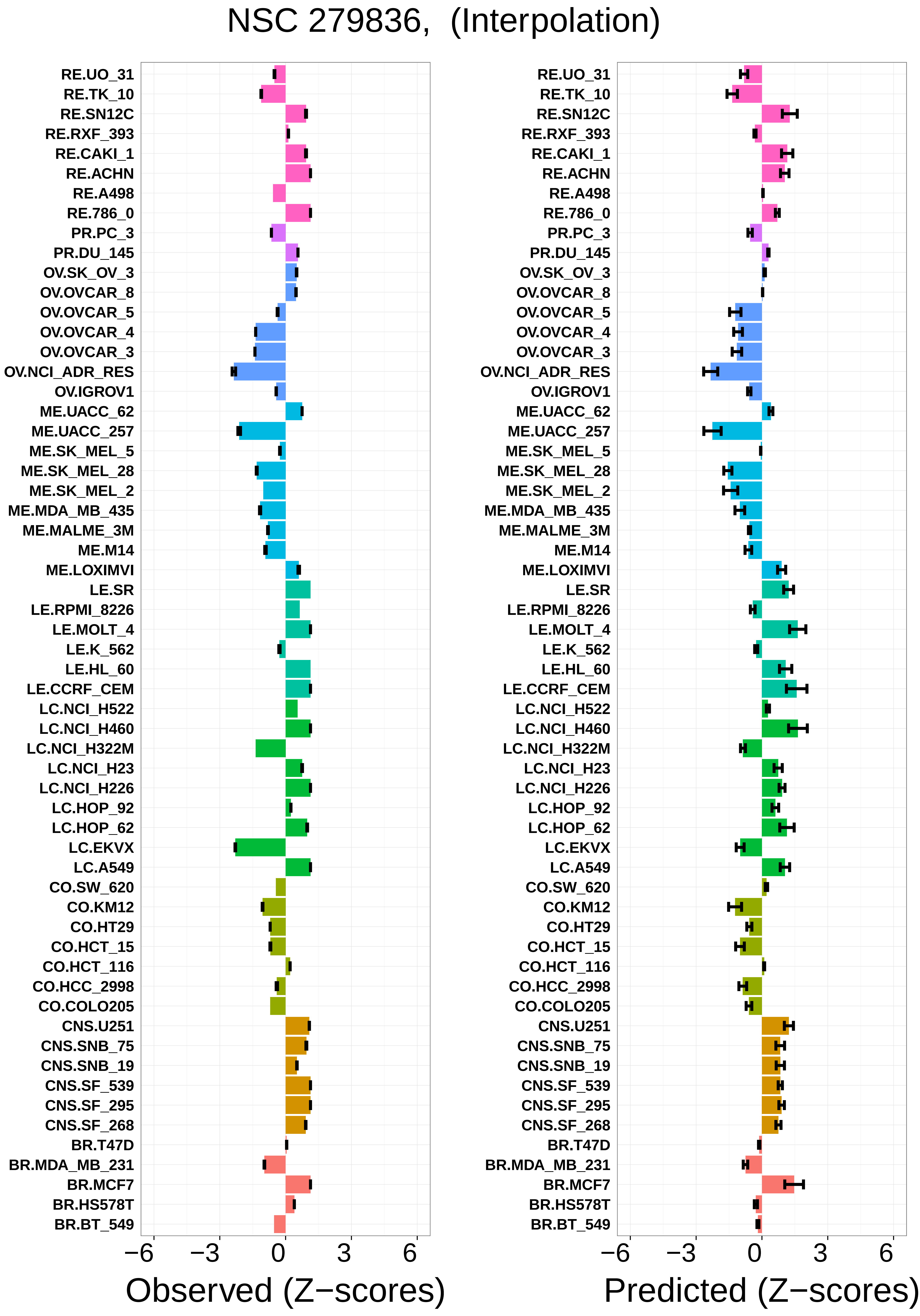

NSC 279836, (Interpolation)
RE.UO_31
RE.TK_10
RE.SN12C
RE.RXF_393
RE.CAKI_1
RE.ACHN
RE.A498
RE.786_0
PR.PC_3
PR.DU_145
OV.SK_OV_3
OV.OVCAR_8
OV.OVCAR_5
OV.OVCAR_4
OV.OVCAR_3
OV.NCI_ADR_RES
OV.IGROV1
ME.UACC_62
ME.UACC_257
ME.SK_MEL_5
ME.SK_MEL_28
ME.SK_MEL_2
ME.MDA_MB_435
ME.MALME_3M
ME.M14
ME.LOXIMVI
LE.SR
LE.RPMI_8226
LE.MOLT_4
LE.K_562
LE.HL_60
LE.CCRF_CEM
LC.NCI_H522
LC.NCI_H460
LC.NCI_H322M
LC.NCI_H23
LC.NCI_H226
LC.HOP_92
LC.HOP_62
LC.EKVX
LC.A549
CO.SW_620
CO.KM12
CO.HT29
CO.HCT_15
CO.HCT_116
CO.HCC_2998
CO.COLO205
CNS.U251
CNS.SNB_75
CNS.SNB_19
CNS.SF_539
CNS.SF_295
CNS.SF_268
BR.T47D
BR.MDA_MB_231
BR.MCF7
BR.HS578T
BR.BT_549
−6
−3
0
3
6
Observed (Z−scores)
Predicted (Z−scores)

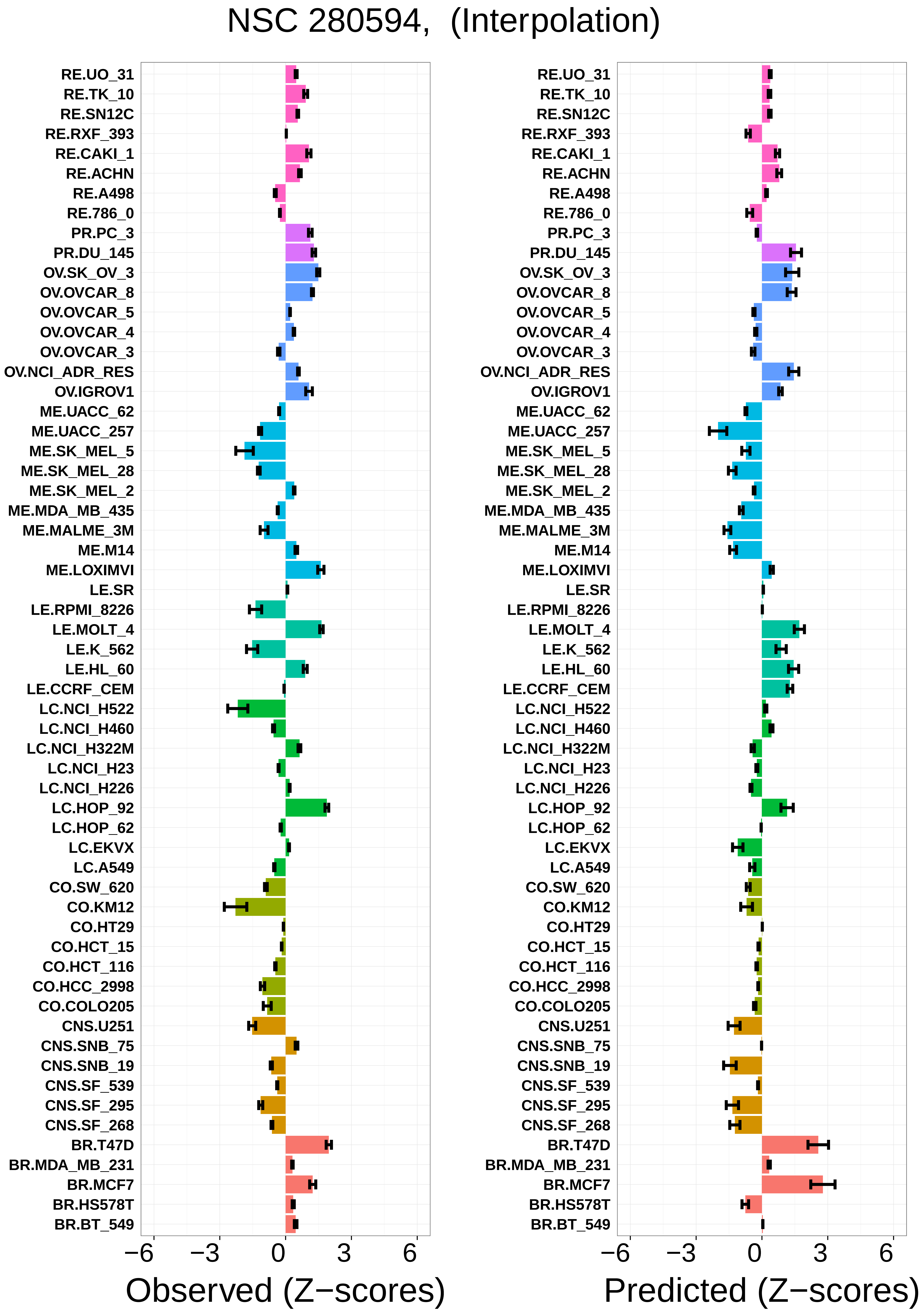
NSC 280594, (Interpolation)
RE.UO_31
RE.TK_10
RE.SN12C
RE.RXF_393
RE.CAKI_1
RE.ACHN
RE.A498
RE.786_0
PR.PC_3
PR.DU_145
OV.SK_OV_3
OV.OVCAR_8
OV.OVCAR_5
OV.OVCAR_4
OV.OVCAR_3
OV.NCI_ADR_RES
OV.IGROV1
ME.UACC_62
ME.UACC_257
ME.SK_MEL_5
ME.SK_MEL_28
ME.SK_MEL_2
ME.MDA_MB_435
ME.MALME_3M
ME.M14
ME.LOXIMVI
LE.SR
LE.RPMI_8226
LE.MOLT_4
LE.K_562
LE.HL_60
LE.CCRF_CEM
LC.NCI_H522
LC.NCI_H460
LC.NCI_H322M
LC.NCI_H23
LC.NCI_H226
LC.HOP_92
LC.HOP_62
LC.EKVX
LC.A549
CO.SW_620
CO.KM12
CO.HT29
CO.HCT_15
CO.HCT_116
CO.HCC_2998
CO.COLO205
CNS.U251
CNS.SNB_75
CNS.SNB_19
CNS.SF_539
CNS.SF_295
CNS.SF_268
BR.T47D
BR.MDA_MB_231
BR.MCF7
BR.HS578T
BR.BT_549
−6
−3
0
3
6
Observed (Z−scores)
Predicted (Z−scores)

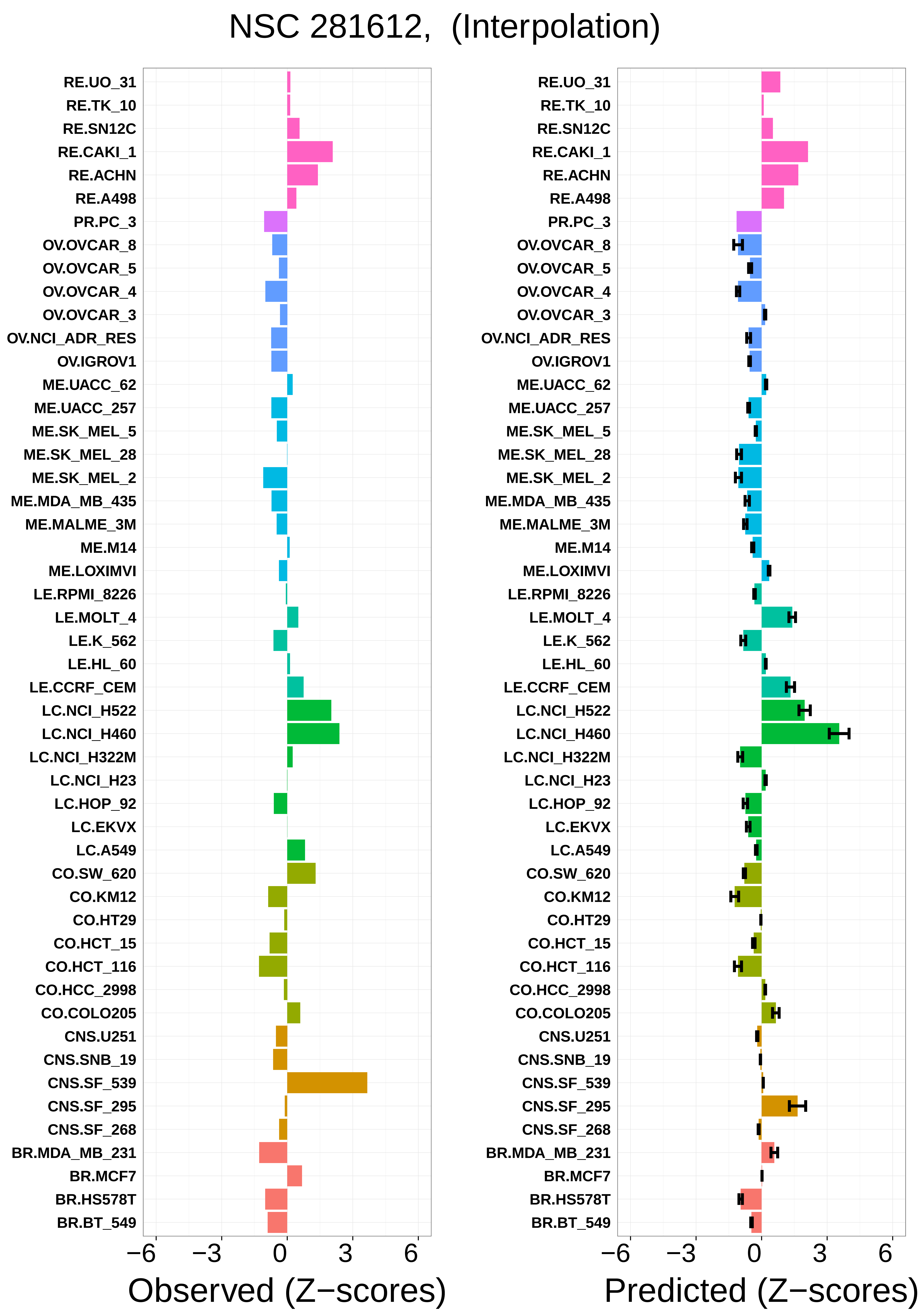
NSC 281612, (Interpolation)
RE.UO_31
RE.TK_10
RE.SN12C
RE.CAKI_1
RE.ACHN
RE.A498
PR.PC_3
OV.OVCAR_8
OV.OVCAR_5
OV.OVCAR_4
OV.OVCAR_3
OV.NCI_ADR_RES
OV.IGROV1
ME.UACC_62
ME.UACC_257
ME.SK_MEL_5
ME.SK_MEL_28
ME.SK_MEL_2
ME.MDA_MB_435
ME.MALME_3M
ME.M14
ME.LOXIMVI
LE.RPMI_8226
LE.MOLT_4
LE.K_562
LE.HL_60
LE.CCRF_CEM
LC.NCI_H522
LC.NCI_H460
LC.NCI_H322M
LC.NCI_H23
LC.HOP_92
LC.EKVX
LC.A549
CO.SW_620
CO.KM12
CO.HT29
CO.HCT_15
CO.HCT_116
CO.HCC_2998
CO.COLO205
CNS.U251
CNS.SNB_19
CNS.SF_539
CNS.SF_295
CNS.SF_268
BR.MDA_MB_231
BR.MCF7
BR.HS578T
BR.BT_549
−6 −3 0 3 6
Observed (Z−scores)
Predicted (Z−scores)

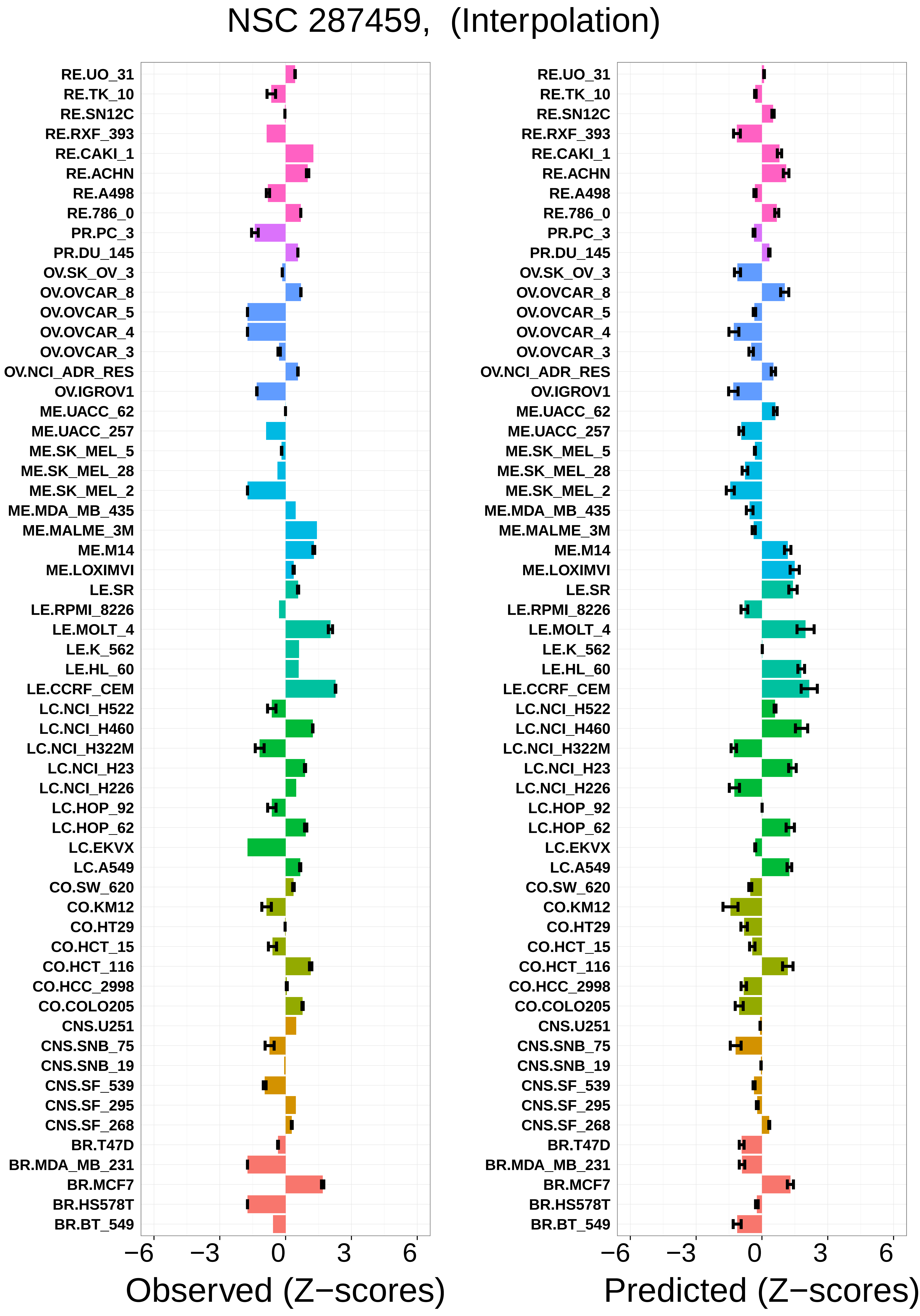
NSC 287459, (Interpolation)
RE.UO_31
RE.TK_10
RE.SN12C
RE.RXF_393
RE.CAKI_1
RE.ACHN
RE.A498
RE.786_0
PR.PC_3
PR.DU_145
OV.SK_OV_3
OV.OVCAR_8
OV.OVCAR_5
OV.OVCAR_4
OV.OVCAR_3
OV.NCI_ADR_RES
OV.IGROV1
ME.UACC_62
ME.UACC_257
ME.SK_MEL_5
ME.SK_MEL_28
ME.SK_MEL_2
ME.MDA_MB_435
ME.MALME_3M
ME.M14
ME.LOXIMVI
LE.SR
LE.RPMI_8226
LE.MOLT_4
LE.K_562
LE.HL_60
LE.CCRF_CEM
LC.NCI_H522
LC.NCI_H460
LC.NCI_H322M
LC.NCI_H23
LC.NCI_H226
LC.HOP_92
LC.HOP_62
LC.EKVX
LC.A549
CO.SW_620
CO.KM12
CO.HT29
CO.HCT_15
CO.HCT_116
CO.HCC_2998
CO.COLO205
CNS.U251
CNS.SNB_75
CNS.SNB_19
CNS.SF_539
CNS.SF_295
CNS.SF_268
BR.T47D
BR.MDA_MB_231
BR.MCF7
BR.HS578T
BR.BT_549
−6 −3 0 3 6
Observed (Z−scores)
Predicted (Z−scores)

NSC 301739, (Interpolation)

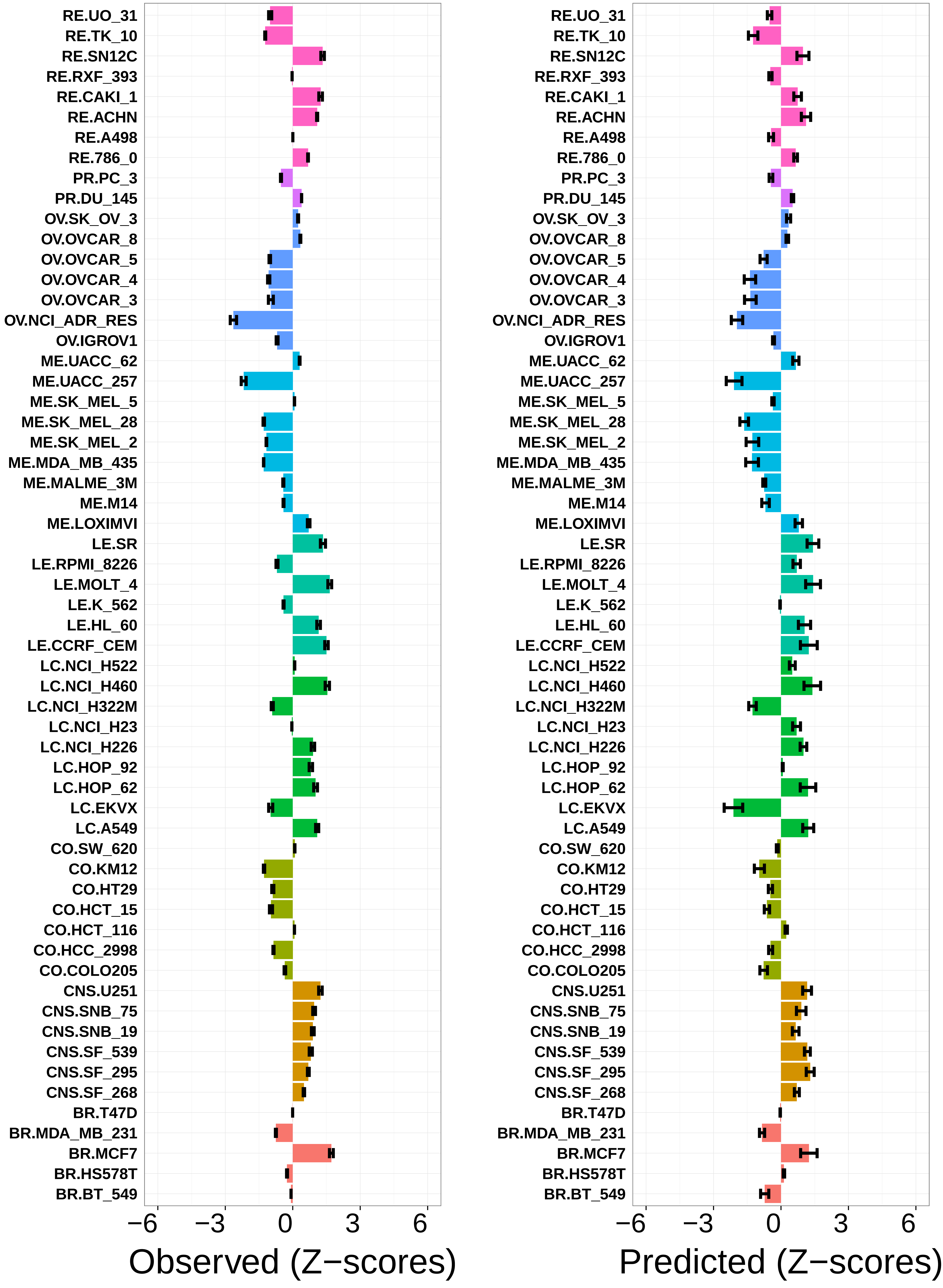

NSC 312887, Fludarabine_phosphate_(USAN) (Interpolation)

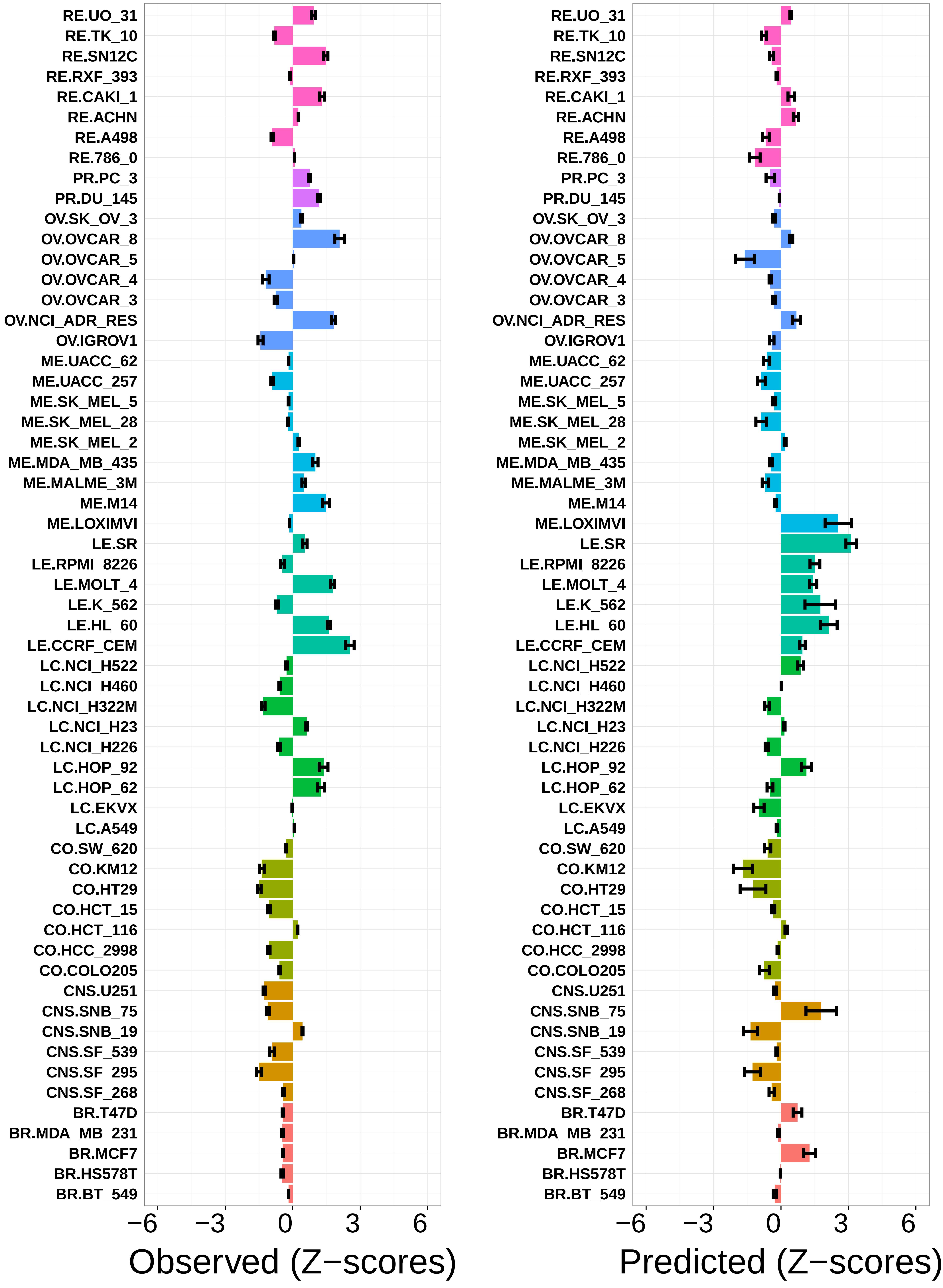

NSC 330507, Tanespimycin (Interpolation)

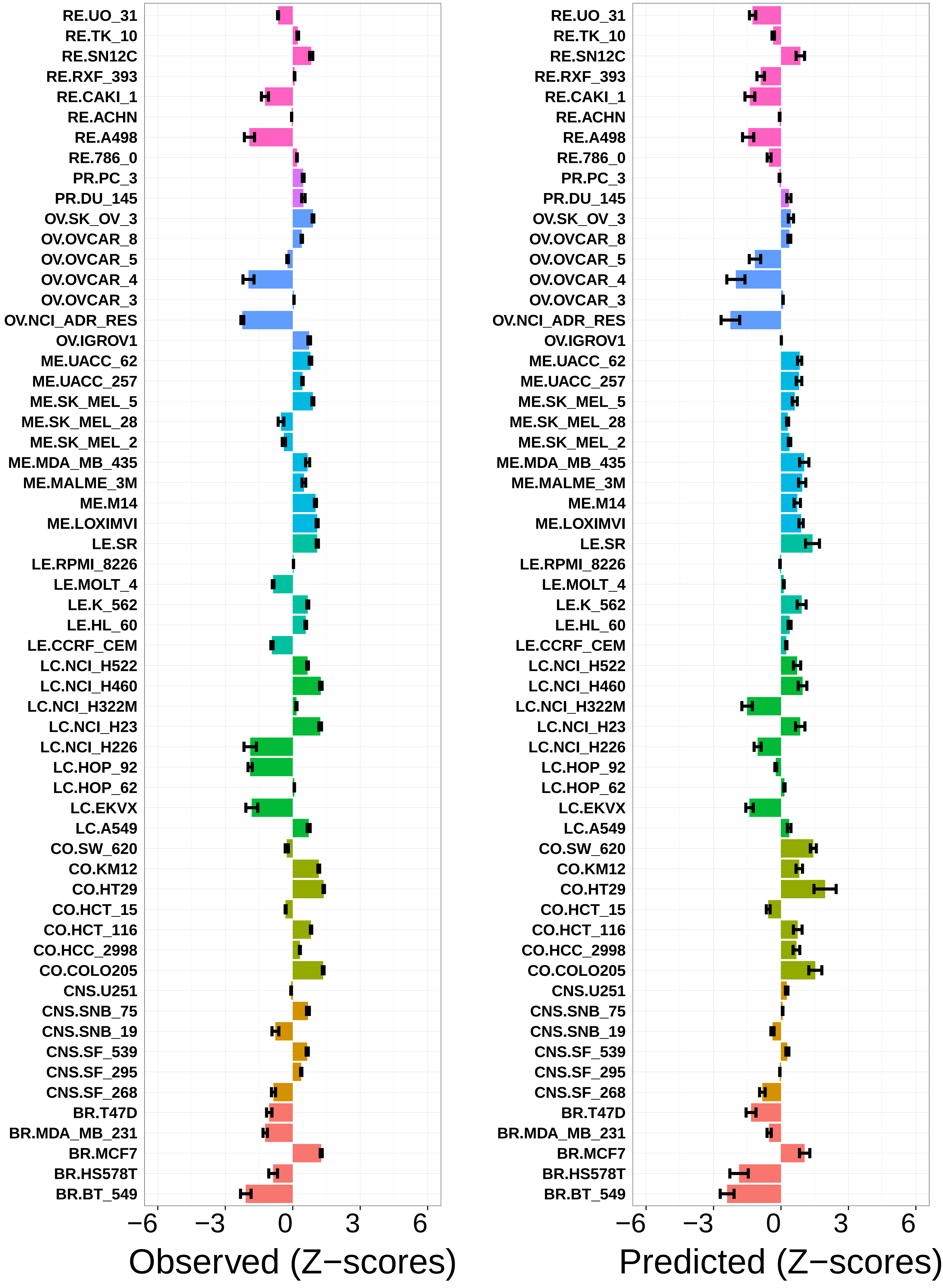

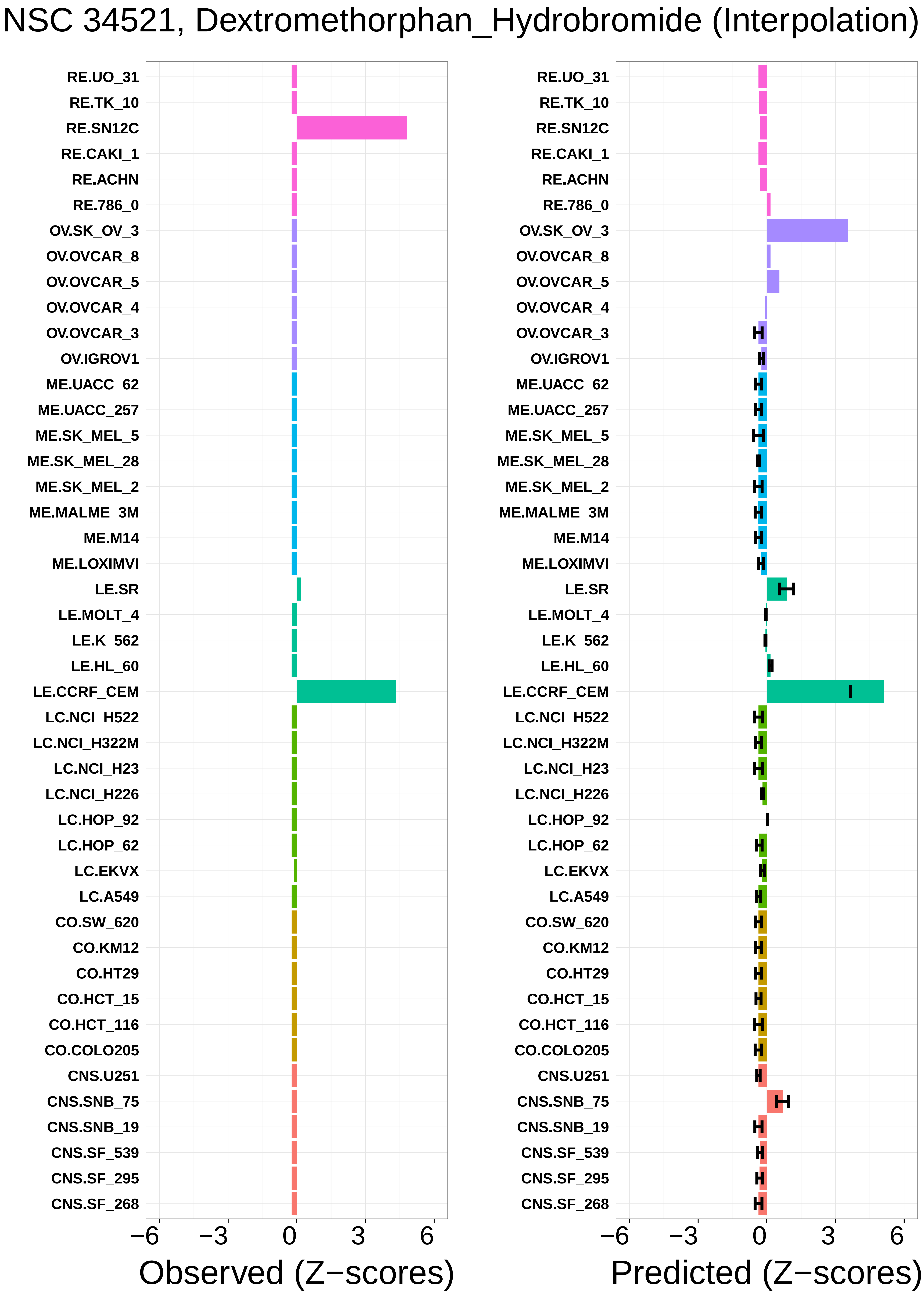

NSC 34521, Dextromethorphan_Hydrobromide (Interpolation)
RE.UO_31
RE.TK_10
RE.SN12C
RE.CAKI_1
RE.ACHN
RE.786_0
OV.SK_OV_3
OV.OVCAR_8
OV.OVCAR_5
OV.OVCAR_4
OV.OVCAR_3
OV.IGROV1
ME.UACC_62
ME.UACC_257
ME.SK_MEL_5
ME.SK_MEL_28
ME.SK_MEL_2
ME.MALME_3M
ME.M14
ME.LOXIMVI
LE.SR
LE.MOLT_4
LE.K_562
LE.HL_60
LE.CCRF_CEM
LC.NCI_H522
LC.NCI_H322M
LC.NCI_H23
LC.NCI_H226
LC.HOP_92
LC.HOP_62
LC.EKVX
LC.A549
CO.SW_620
CO.KM12
CO.HT29
CO.HCT_15
CO.HCT_116
CO.COLO205
CNS.U251
CNS.SNB_75
CNS.SNB_19
CNS.SF_539
CNS.SF_295
CNS.SF_268
−6
−3
0
3
6
Observed (Z−scores)
Predicted (Z−scores)

NSC 354258, 8−Chloro−Adenosine (Interpolation)

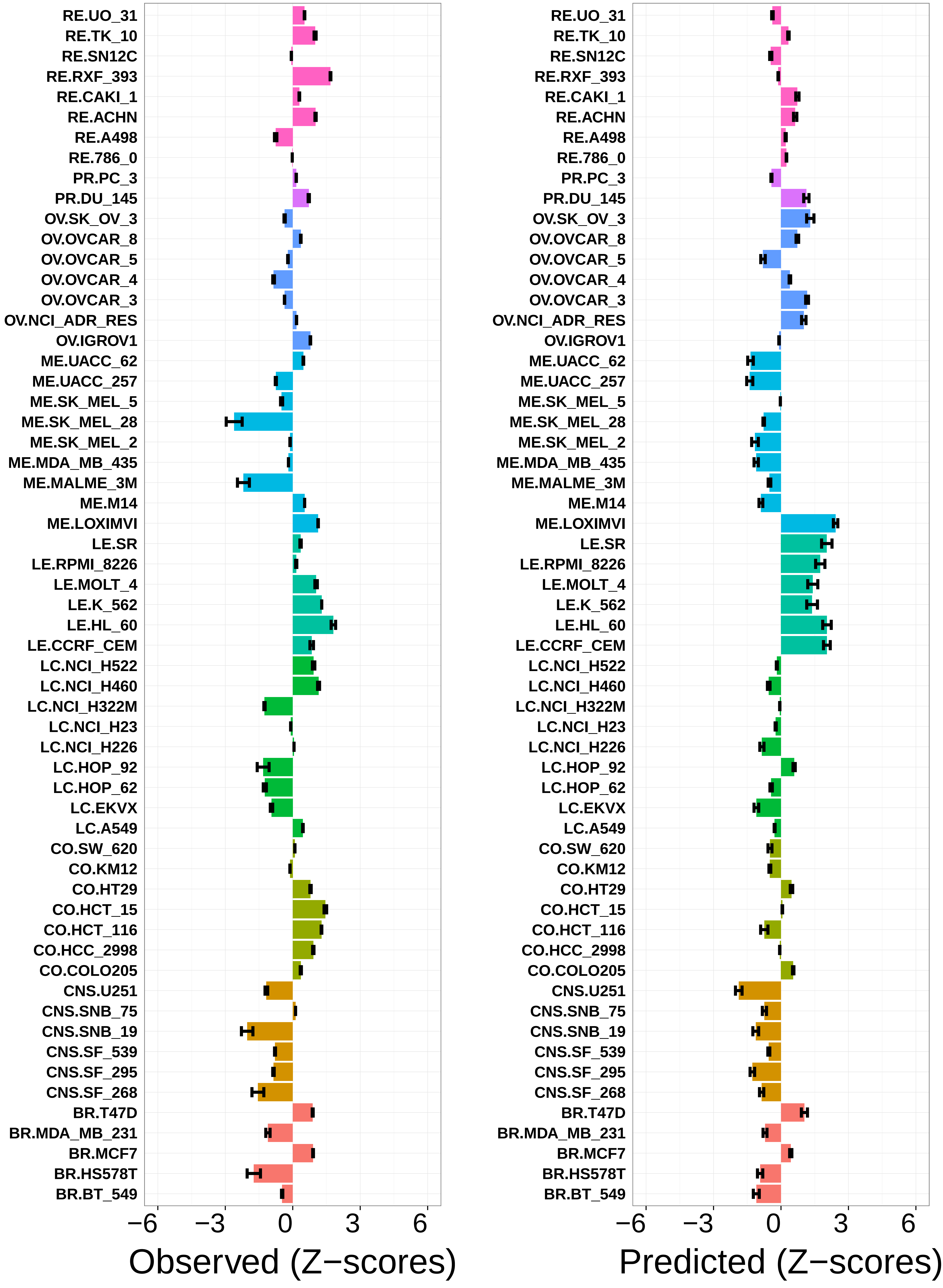

NSC 354462, Hypothemycin (Interpolation)

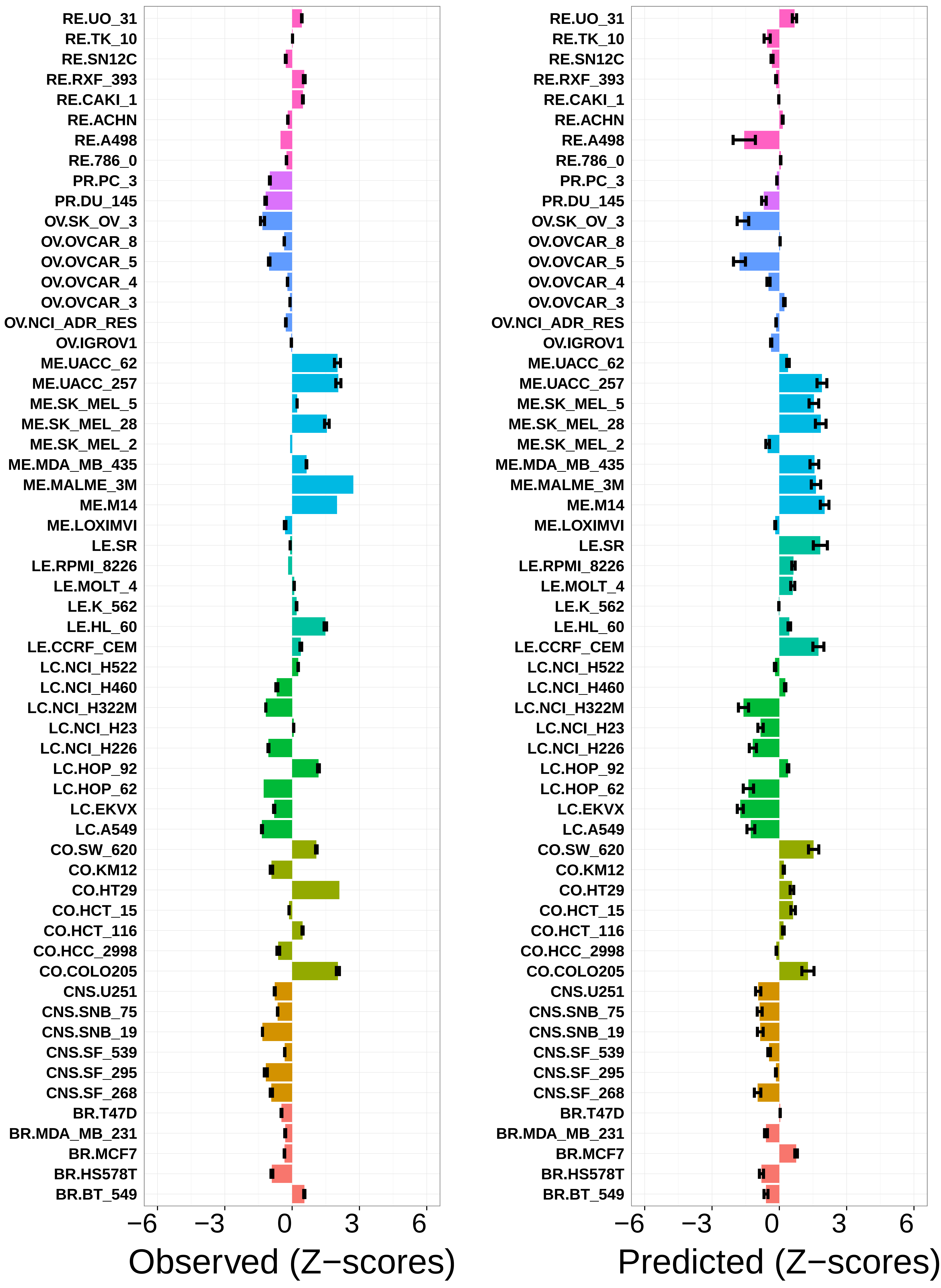

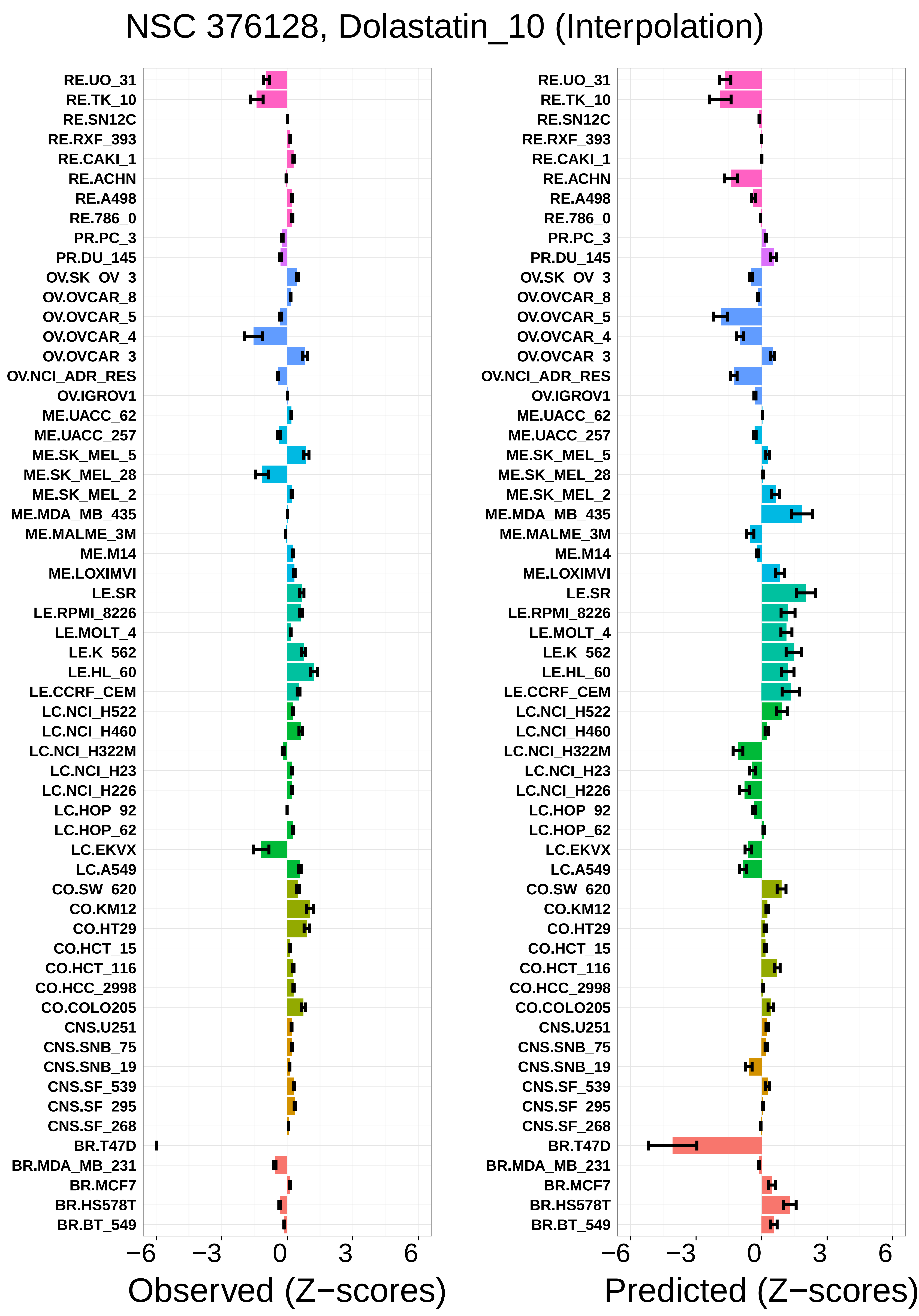
NSC 376128, Dolastatin_10 (Interpolation)
RE.UO_31
RE.TK_10
RE.SN12C
RE.RXF_393
RE.CAKI_1
RE.ACHN
RE.A498
RE.786_0
PR.PC_3
PR.DU_145
OV.SK_OV_3
OV.OVCAR_8
OV.OVCAR_5
OV.OVCAR_4
OV.OVCAR_3
OV.NCI_ADR_RES
OV.IGROV1
ME.UACC_62
ME.UACC_257
ME.SK_MEL_5
ME.SK_MEL_28
ME.SK_MEL_2
ME.MDA_MB_435
ME.MALME_3M
ME.M14
ME.LOXIMVI
LE.SR
LE.RPMI_8226
LE.MOLT_4
LE.K_562
LE.HL_60
LE.CCRF_CEM
LC.NCI_H522
LC.NCI_H460
LC.NCI_H322M
LC.NCI_H23
LC.NCI_H226
LC.HOP_92
LC.HOP_62
LC.EKVX
LC.A549
CO.SW_620
CO.KM12
CO.HT29
CO.HCT_15
CO.HCT_116
CO.HCC_2998
CO.COLO205
CNS.U251
CNS.SNB_75
CNS.SNB_19
CNS.SF_539
CNS.SF_295
CNS.SF_268
BR.T47D
BR.MDA_MB_231
BR.MCF7
BR.HS578T
BR.BT_549
−6 −3 0 3 6
Observed (Z−scores)
Predicted (Z−scores)

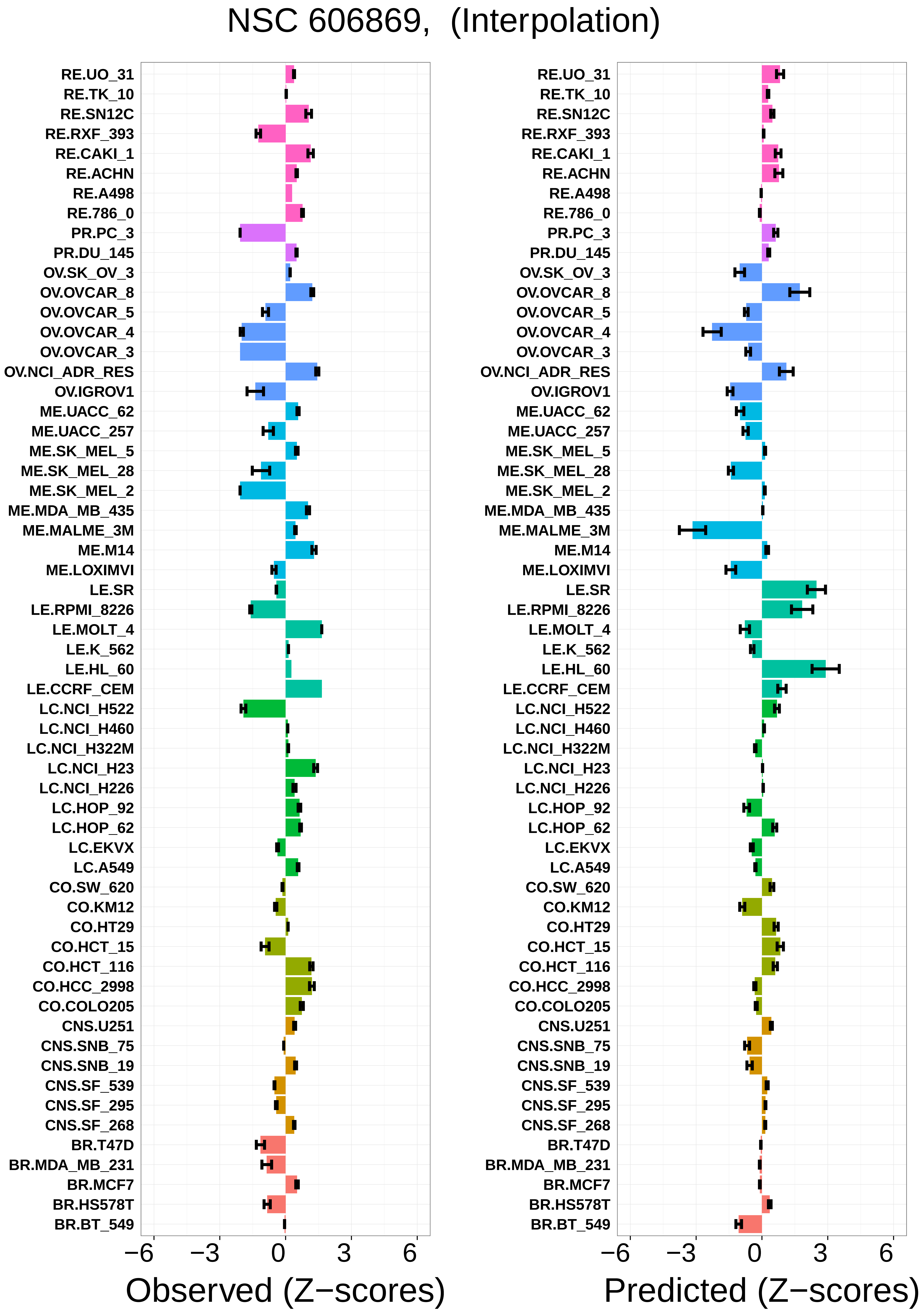
NSC 606869, (Interpolation)
RE.UO_31
RE.TK_10
RE.SN12C
RE.RXF_393
RE.CAKI_1
RE.ACHN
RE.A498
RE.786_0
PR.PC_3
PR.DU_145
OV.SK_OV_3
OV.OVCAR_8
OV.OVCAR_5
OV.OVCAR_4
OV.OVCAR_3
OV.NCI_ADR_RES
OV.IGROV1
ME.UACC_62
ME.UACC_257
ME.SK_MEL_5
ME.SK_MEL_28
ME.SK_MEL_2
ME.MDA_MB_435
ME.MALME_3M
ME.M14
ME.LOXIMVI
LE.SR
LE.RPMI_8226
LE.MOLT_4
LE.K_562
LE.HL_60
LE.CCRF_CEM
LC.NCI_H522
LC.NCI_H460
LC.NCI_H322M
LC.NCI_H23
LC.NCI_H226
LC.HOP_92
LC.HOP_62
LC.EKVX
LC.A549
CO.SW_620
CO.KM12
CO.HT29
CO.HCT_15
CO.HCT_116
CO.HCC_2998
CO.COLO205
CNS.U251
CNS.SNB_75
CNS.SNB_19
CNS.SF_539
CNS.SF_295
CNS.SF_268
BR.T47D
BR.MDA_MB_231
BR.MCF7
BR.HS578T
BR.BT_549
−6
−3
0
3
6
Observed (Z−scores)
Predicted (Z−scores)

NSC 609699, Topotecan_Hydrochloride (Interpolation)

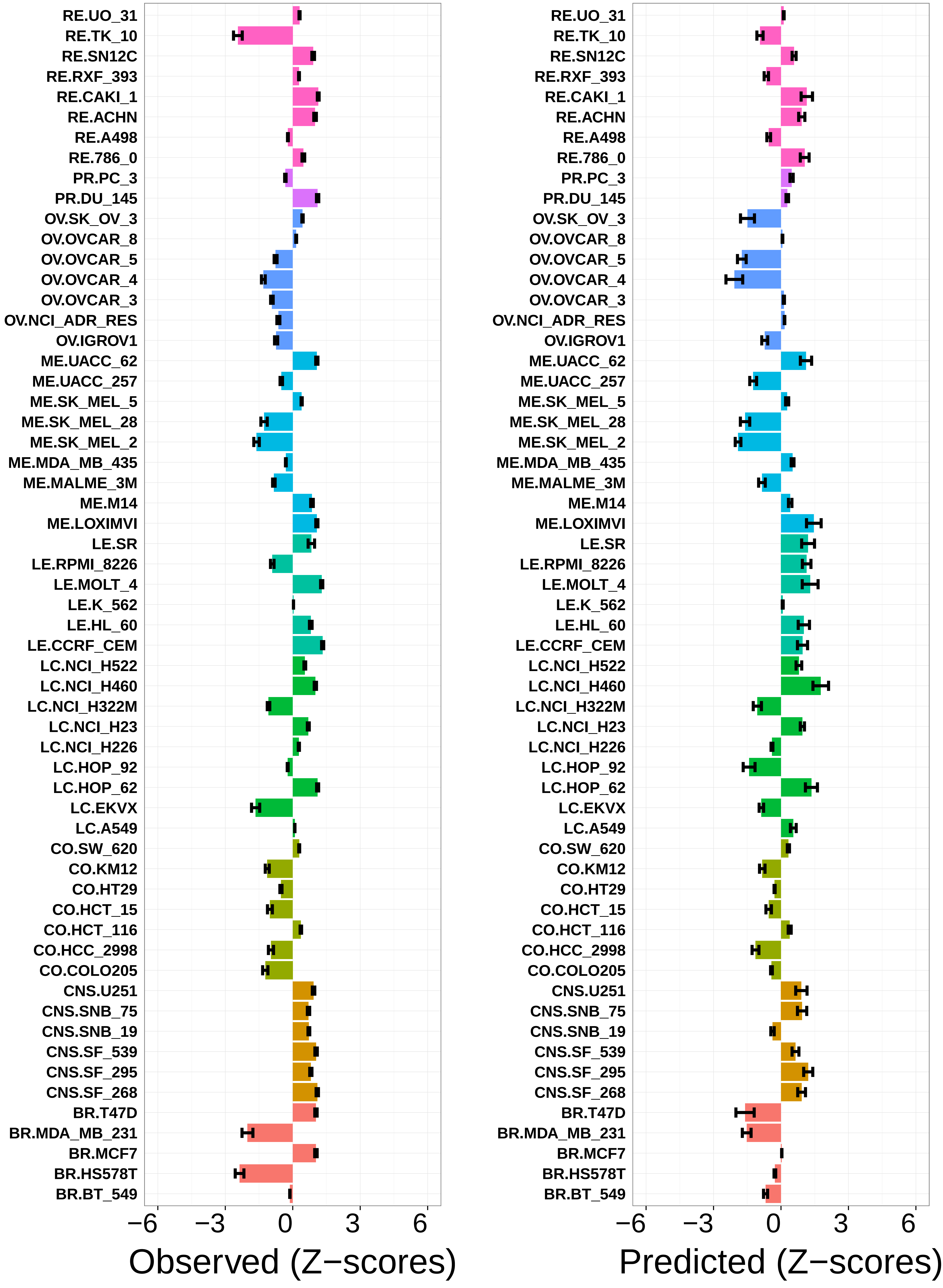

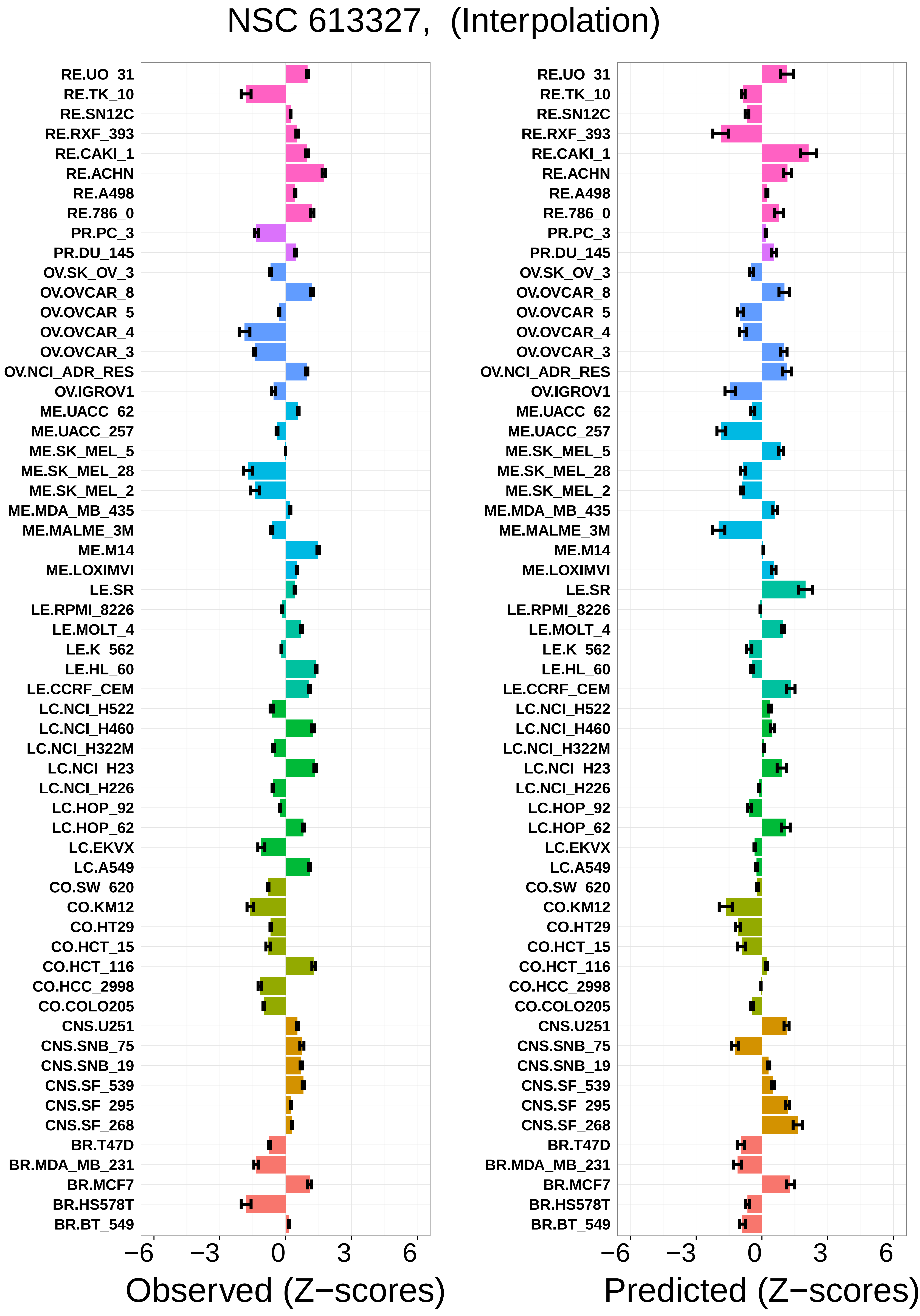

NSC 613327, (Interpolation)
RE.UO_31
RE.TK_10
RE.SN12C
RE.RXF_393
RE.CAKI_1
RE.ACHN
RE.A498
RE.786_0
PR.PC_3
PR.DU_145
OV.SK_OV_3
OV.OVCAR_8
OV.OVCAR_5
OV.OVCAR_4
OV.OVCAR_3
OV.NCI_ADR_RES
OV.IGROV1
ME.UACC_62
ME.UACC_257
ME.SK_MEL_5
ME.SK_MEL_28
ME.SK_MEL_2
ME.MDA_MB_435
ME.MALME_3M
ME.M14
ME.LOXIMVI
LE.SR
LE.RPMI_8226
LE.MOLT_4
LE.K_562
LE.HL_60
LE.CCRF_CEM
LC.NCI_H522
LC.NCI_H460
LC.NCI_H322M
LC.NCI_H23
LC.NCI_H226
LC.HOP_92
LC.HOP_62
LC.EKVX
LC.A549
CO.SW_620
CO.KM12
CO.HT29
CO.HCT_15
CO.HCT_116
CO.HCC_2998
CO.COLO205
CNS.U251
CNS.SNB_75
CNS.SNB_19
CNS.SF_539
CNS.SF_295
CNS.SF_268
BR.T47D
BR.MDA_MB_231
BR.MCF7
BR.HS578T
BR.BT_549
−6
−3
0
3
6
Observed (Z−scores)
Predicted (Z−scores)

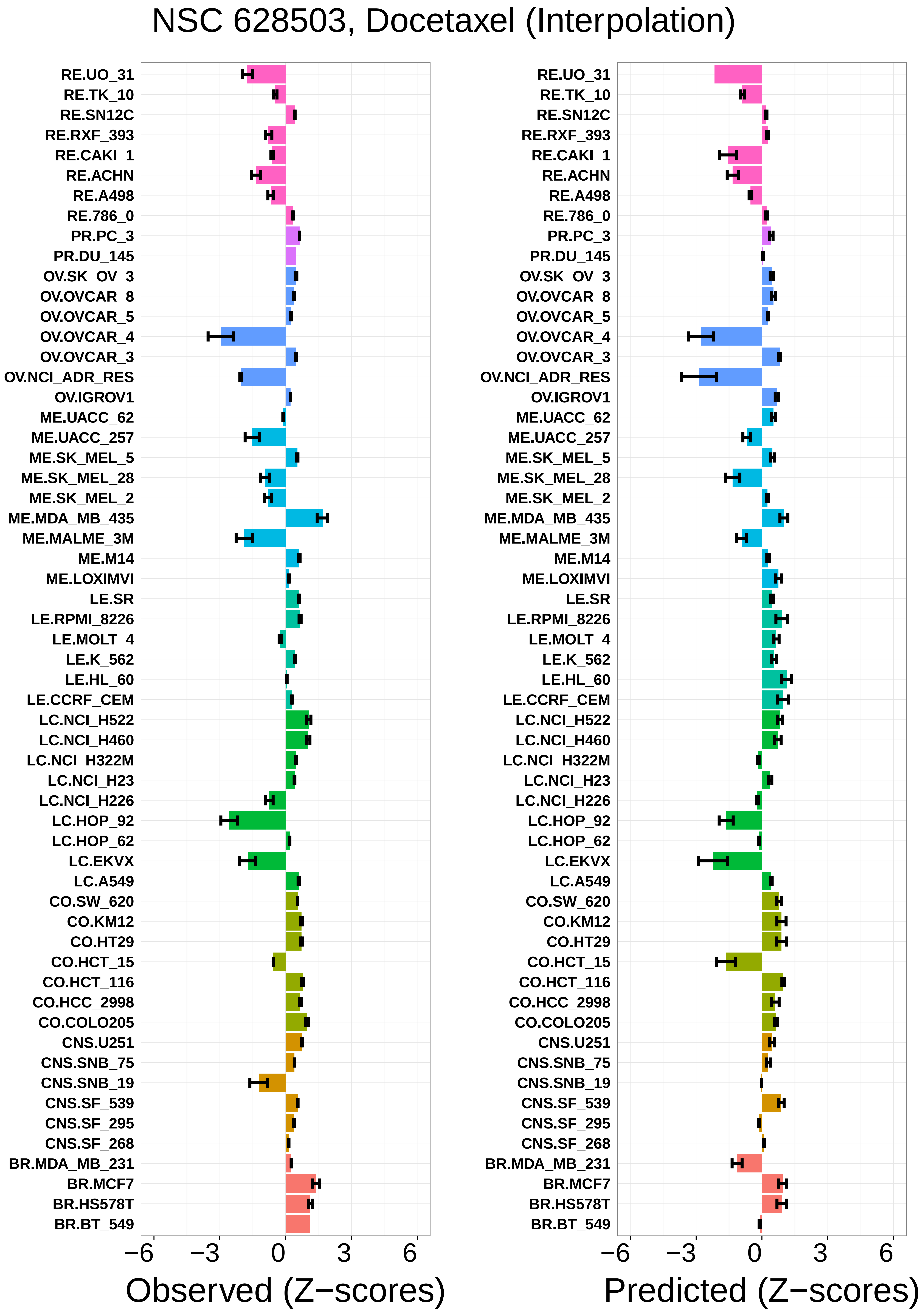
NSC 628503, Docetaxel (Interpolation)
RE.UO_31
RE.TK_10
RE.SN12C
RE.RXF_393
RE.CAKI_1
RE.ACHN
RE.A498
RE.786_0
PR.PC_3
PR.DU_145
OV.SK_OV_3
OV.OVCAR_8
OV.OVCAR_5
OV.OVCAR_4
OV.OVCAR_3
OV.NCI_ADR_RES
OV.IGROV1
ME.UACC_62
ME.UACC_257
ME.SK_MEL_5
ME.SK_MEL_28
ME.SK_MEL_2
ME.MDA_MB_435
ME.MALME_3M
ME.M14
ME.LOXIMVI
LE.SR
LE.RPMI_8226
LE.MOLT_4
LE.K_562
LE.HL_60
LE.CCRF_CEM
LC.NCI_H522
LC.NCI_H460
LC.NCI_H322M
LC.NCI_H23
LC.NCI_H226
LC.HOP_92
LC.HOP_62
LC.EKVX
LC.A549
CO.SW_620
CO.KM12
CO.HT29
CO.HCT_15
CO.HCT_116
CO.HCC_2998
CO.COLO205
CNS.U251
CNS.SNB_75
CNS.SNB_19
CNS.SF_539
CNS.SF_295
CNS.SF_268
BR.MDA_MB_231
BR.MCF7
BR.HS578T
BR.BT_549
−6 −3 0 3 6
Observed (Z−scores)
Predicted (Z−scores)

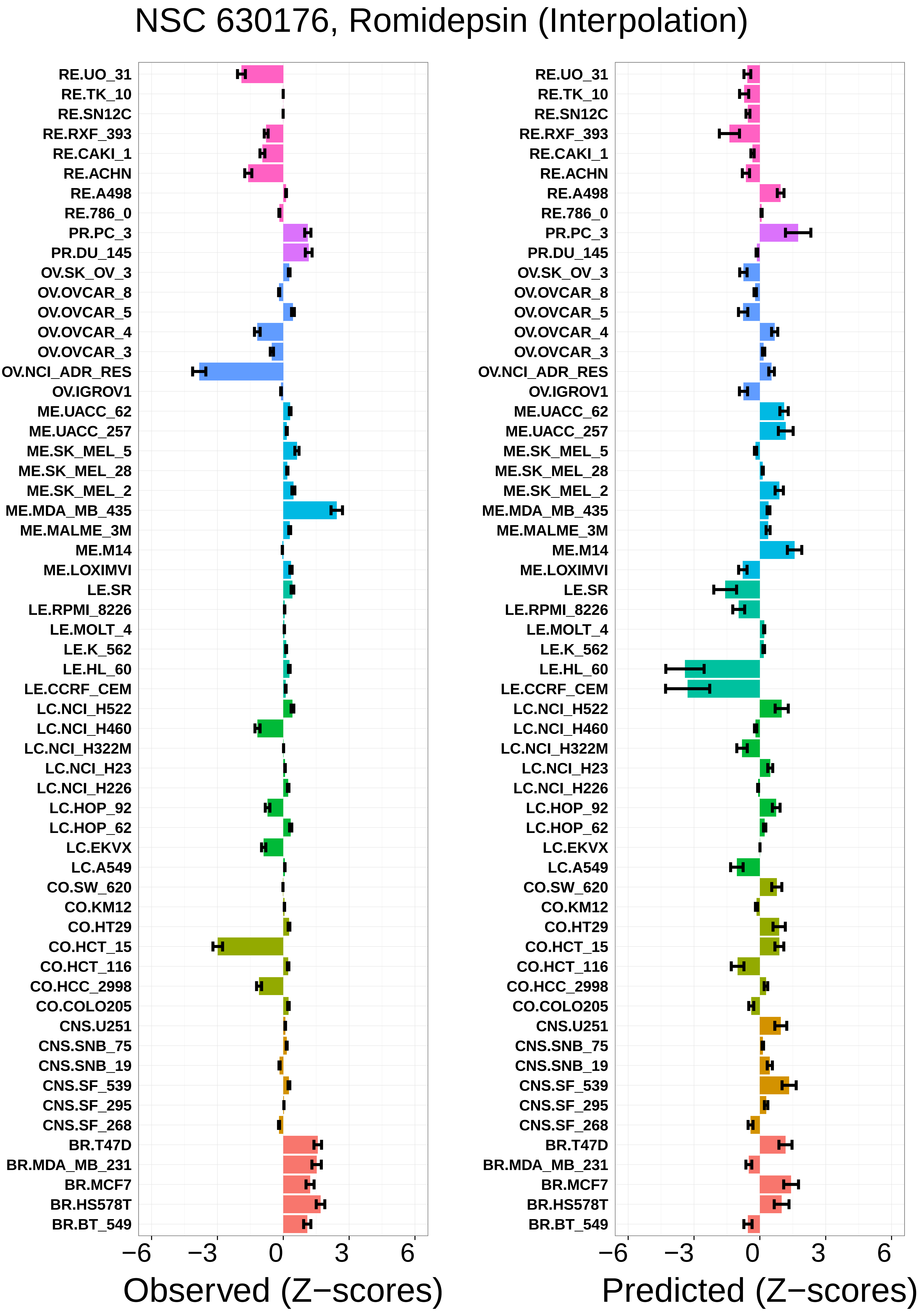
NSC 630176, Romidepsin (Interpolation)
RE.UO_31
RE.TK_10
RE.SN12C
RE.RXF_393
RE.CAKI_1
RE.ACHN
RE.A498
RE.786_0
PR.PC_3
PR.DU_145
OV.SK_OV_3
OV.OVCAR_8
OV.OVCAR_5
OV.OVCAR_4
OV.OVCAR_3
OV.NCI_ADR_RES
OV.IGROV1
ME.UACC_62
ME.UACC_257
ME.SK_MEL_5
ME.SK_MEL_28
ME.SK_MEL_2
ME.MDA_MB_435
ME.MALME_3M
ME.M14
ME.LOXIMVI
LE.SR
LE.RPMI_8226
LE.MOLT_4
LE.K_562
LE.HL_60
LE.CCRF_CEM
LC.NCI_H522
LC.NCI_H460
LC.NCI_H322M
LC.NCI_H23
LC.NCI_H226
LC.HOP_92
LC.HOP_62
LC.EKVX
LC.A549
CO.SW_620
CO.KM12
CO.HT29
CO.HCT_15
CO.HCT_116
CO.HCC_2998
CO.COLO205
CNS.U251
CNS.SNB_75
CNS.SNB_19
CNS.SF_539
CNS.SF_295
CNS.SF_268
BR.T47D
BR.MDA_MB_231
BR.MCF7
BR.HS578T
BR.BT_549
−6
−3
0
3
6
Observed (Z−scores)
Predicted (Z−scores)

NSC 633782, (Interpolation)

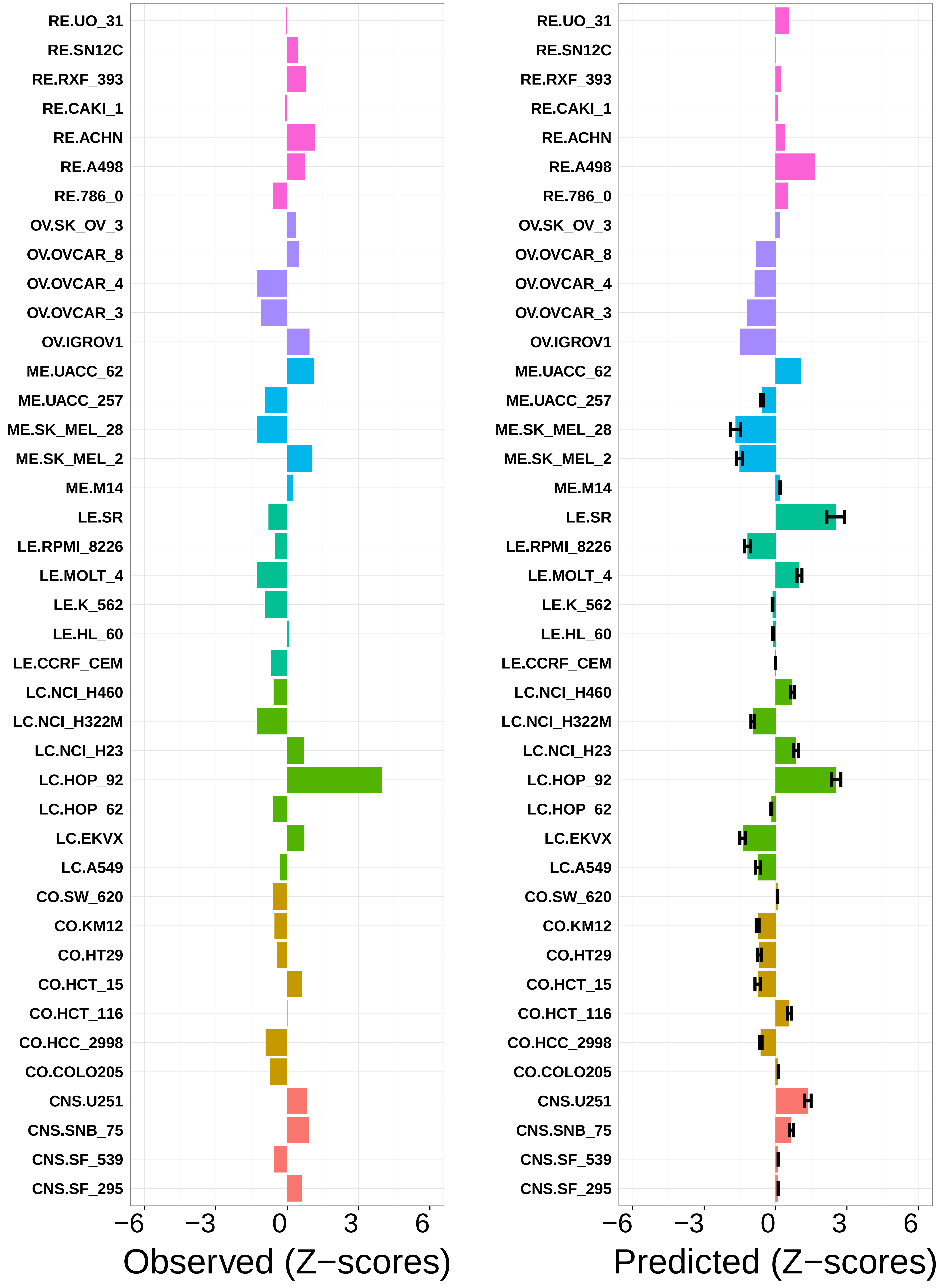

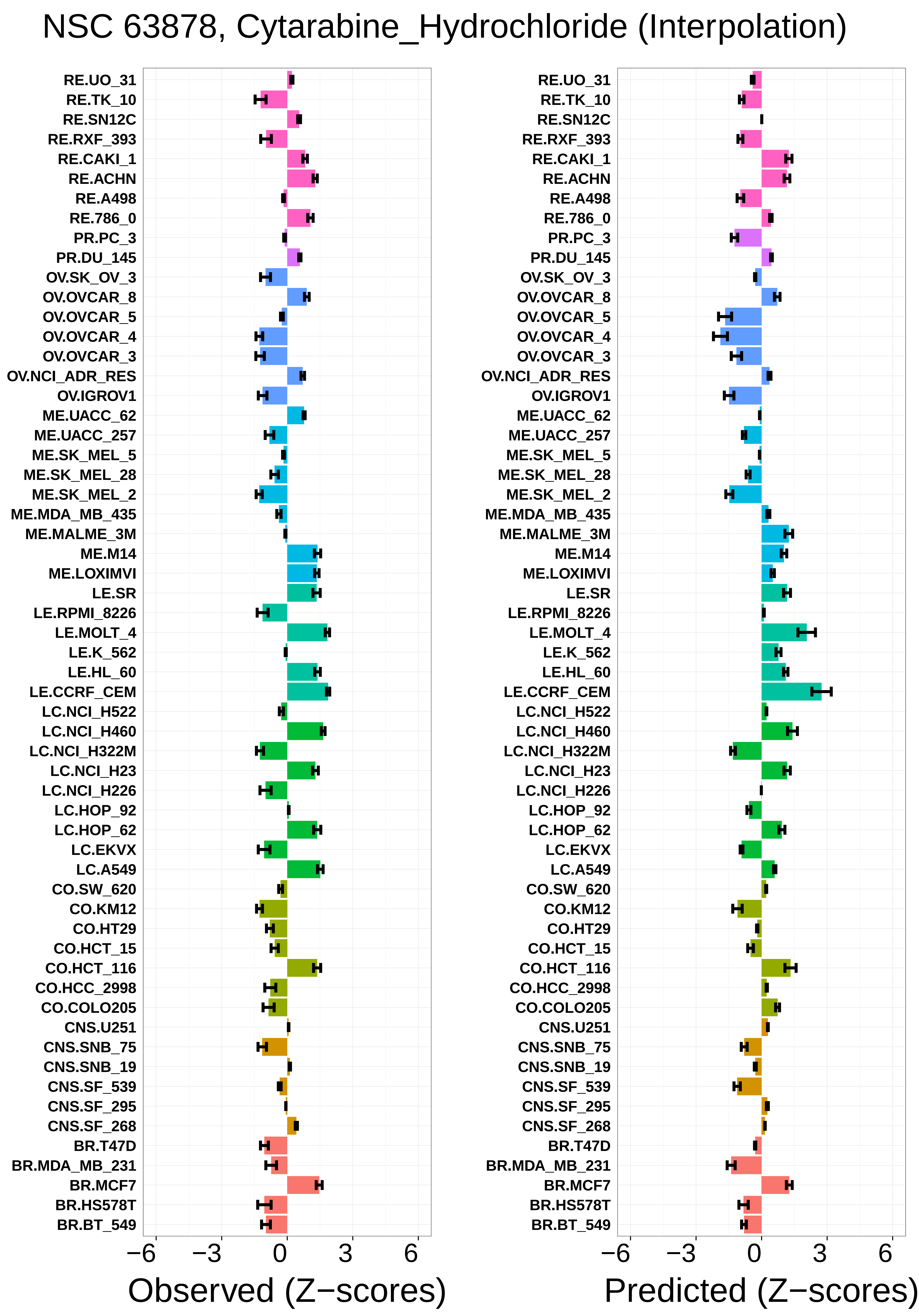
NSC 63878, Cytarabine_Hydrochloride (Interpolation)
RE.UO_31
RE.TK_10
RE.SN12C
RE.RXF_393
RE.CAKI_1
RE.ACHN
RE.A498
RE.786_0
PR.PC_3
PR.DU_145
OV.SK_OV_3
OV.OVCAR_8
OV.OVCAR_5
OV.OVCAR_4
OV.OVCAR_3
OV.NCI_ADR_RES
OV.IGROV1
ME.UACC_62
ME.UACC_257
ME.SK_MEL_5
ME.SK_MEL_28
ME.SK_MEL_2
ME.MDA_MB_435
ME.MALME_3M
ME.M14
ME.LOXIMVI
LE.SR
LE.RPMI_8226
LE.MOLT_4
LE.K_562
LE.HL_60
LE.CCRF_CEM
LC.NCI_H522
LC.NCI_H460
LC.NCI_H322M
LC.NCI_H23
LC.NCI_H226
LC.HOP_92
LC.HOP_62
LC.EKVX
LC.A549
CO.SW_620
CO.KM12
CO.HT29
CO.HCT_15
CO.HCT_116
CO.HCC_2998
CO.COLO205
CNS.U251
CNS.SNB_75
CNS.SNB_19
CNS.SF_539
CNS.SF_295
CNS.SF_268
BR.T47D
BR.MDA_MB_231
BR.MCF7
BR.HS578T
BR.BT_549
−6 −3 0 3 6
Observed (Z−scores)
Predicted (Z−scores)

NSC 639186, (Interpolation)

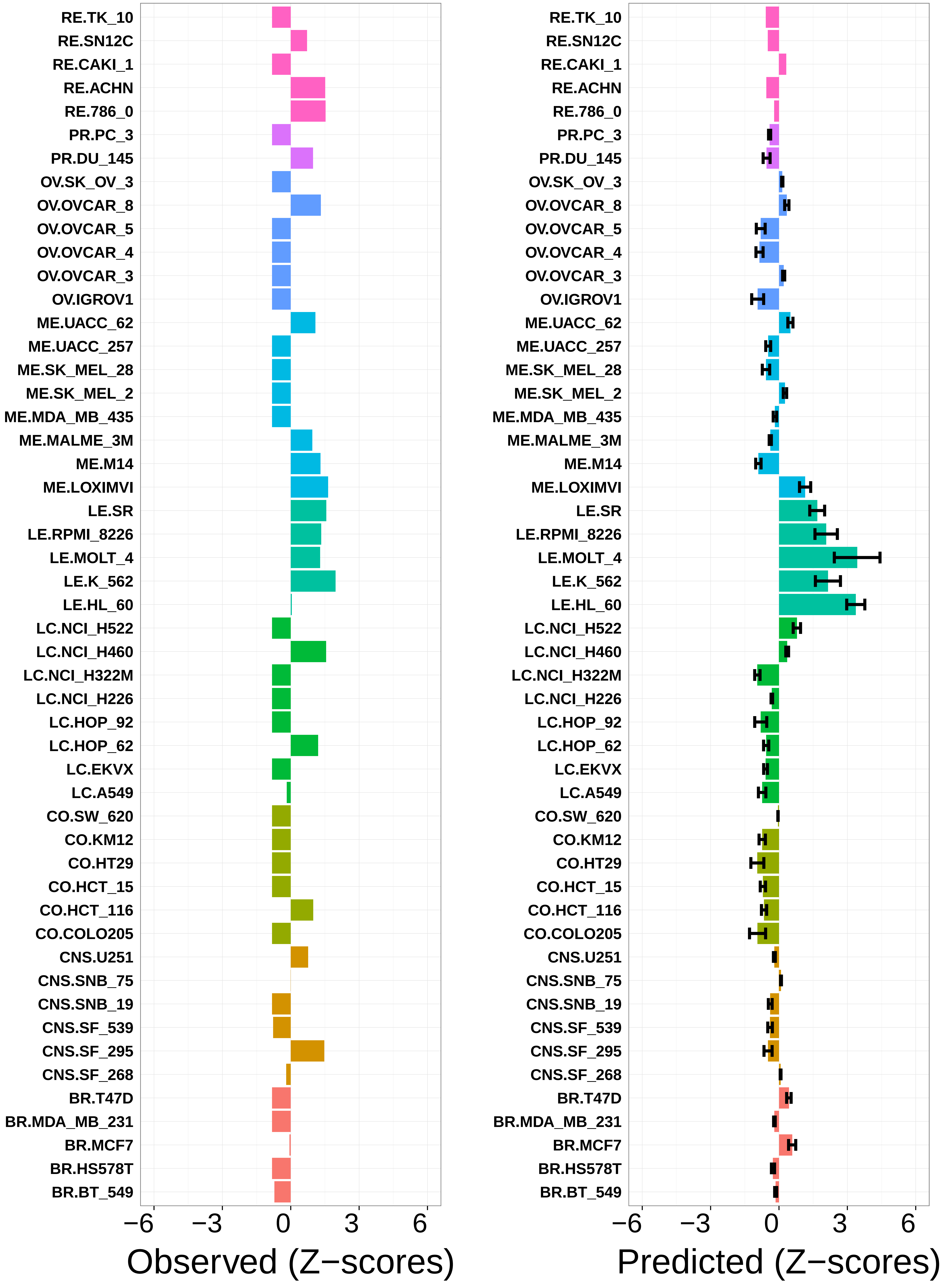

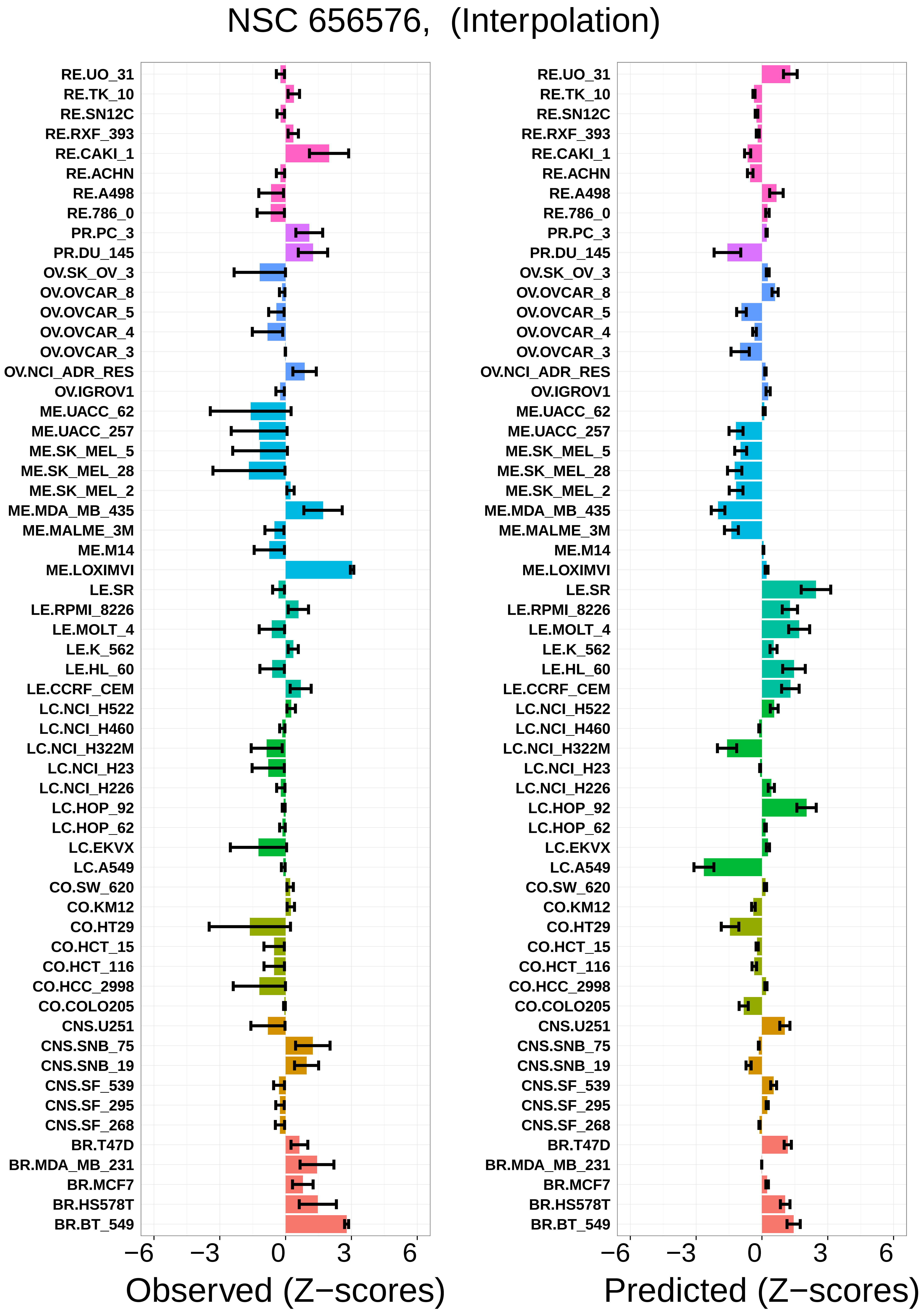

NSC 656576, (Interpolation)
RE.UO_31
RE.TK_10
RE.SN12C
RE.RXF_393
RE.CAKI_1
RE.ACHN
RE.A498
RE.786_0
PR.PC_3
PR.DU_145
OV.SK_OV_3
OV.OVCAR_8
OV.OVCAR_5
OV.OVCAR_4
OV.OVCAR_3
OV.NCI_ADR_RES
OV.IGROV1
ME.UACC_62
ME.UACC_257
ME.SK_MEL_5
ME.SK_MEL_28
ME.SK_MEL_2
ME.MDA_MB_435
ME.MALME_3M
ME.M14
ME.LOXIMVI
LE.SR
LE.RPMI_8226
LE.MOLT_4
LE.K_562
LE.HL_60
LE.CCRF_CEM
LC.NCI_H522
LC.NCI_H460
LC.NCI_H322M
LC.NCI_H23
LC.NCI_H226
LC.HOP_92
LC.HOP_62
LC.EKVX
LC.A549
CO.SW_620
CO.KM12
CO.HT29
CO.HCT_15
CO.HCT_116
CO.HCC_2998
CO.COLO205
CNS.U251
CNS.SNB_75
CNS.SNB_19
CNS.SF_539
CNS.SF_295
CNS.SF_268
BR.T47D
BR.MDA_MB_231
BR.MCF7
BR.HS578T
BR.BT_549
−6 −3 0 3 6
Observed (Z−scores)
Predicted (Z−scores)

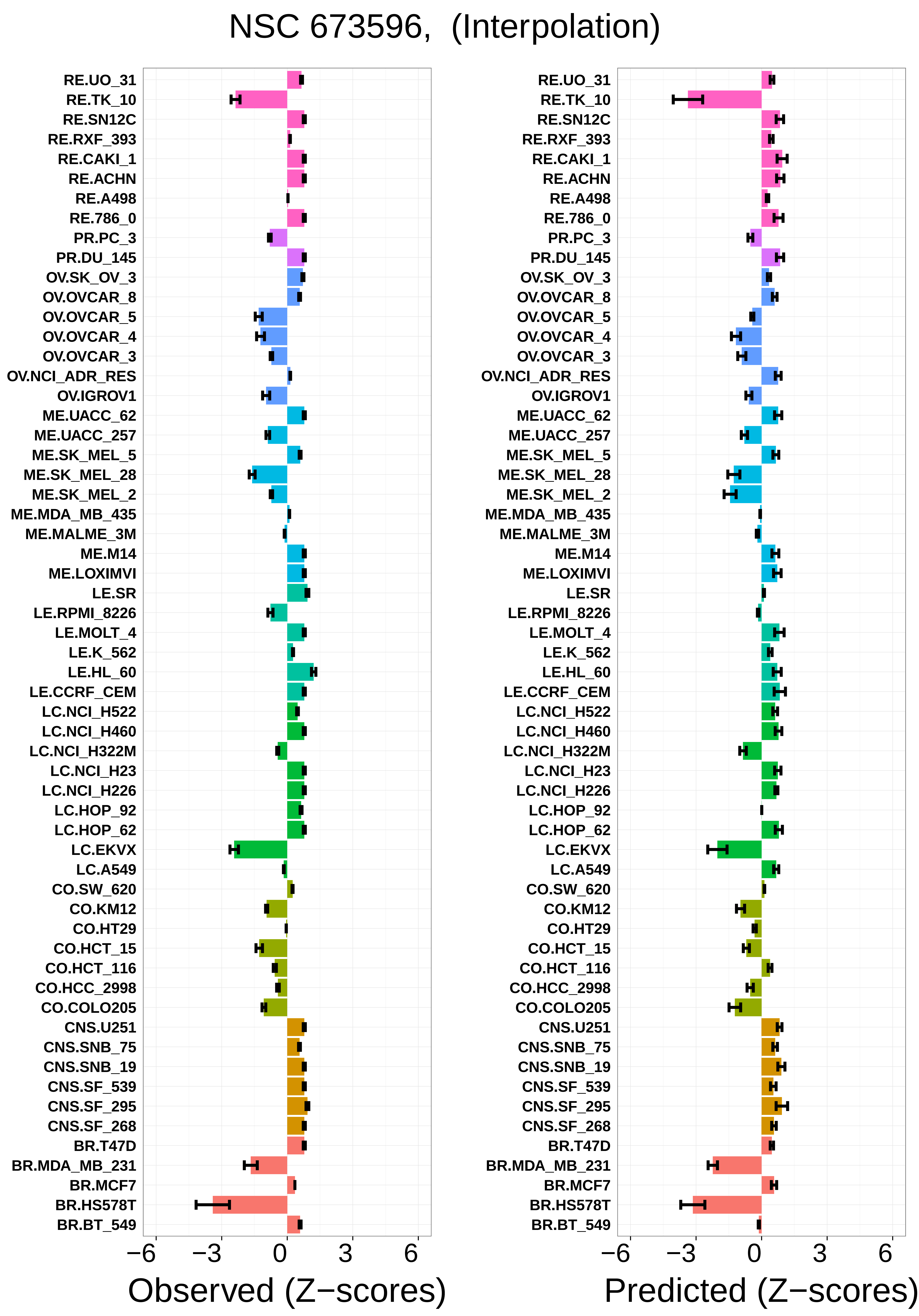
NSC 673596, (Interpolation)
RE.UO_31
RE.TK_10
RE.SN12C
RE.RXF_393
RE.CAKI_1
RE.ACHN
RE.A498
RE.786_0
PR.PC_3
PR.DU_145
OV.SK_OV_3
OV.OVCAR_8
OV.OVCAR_5
OV.OVCAR_4
OV.OVCAR_3
OV.NCI_ADR_RES
OV.IGROV1
ME.UACC_62
ME.UACC_257
ME.SK_MEL_5
ME.SK_MEL_28
ME.SK_MEL_2
ME.MDA_MB_435
ME.MALME_3M
ME.M14
ME.LOXIMVI
LE.SR
LE.RPMI_8226
LE.MOLT_4
LE.K_562
LE.HL_60
LE.CCRF_CEM
LC.NCI_H522
LC.NCI_H460
LC.NCI_H322M
LC.NCI_H23
LC.NCI_H226
LC.HOP_92
LC.HOP_62
LC.EKVX
LC.A549
CO.SW_620
CO.KM12
CO.HT29
CO.HCT_15
CO.HCT_116
CO.HCC_2998
CO.COLO205
CNS.U251
CNS.SNB_75
CNS.SNB_19
CNS.SF_539
CNS.SF_295
CNS.SF_268
BR.T47D
BR.MDA_MB_231
BR.MCF7
BR.HS578T
BR.BT_549
−6
−3
0
3
6
Observed (Z−scores)
Predicted (Z−scores)

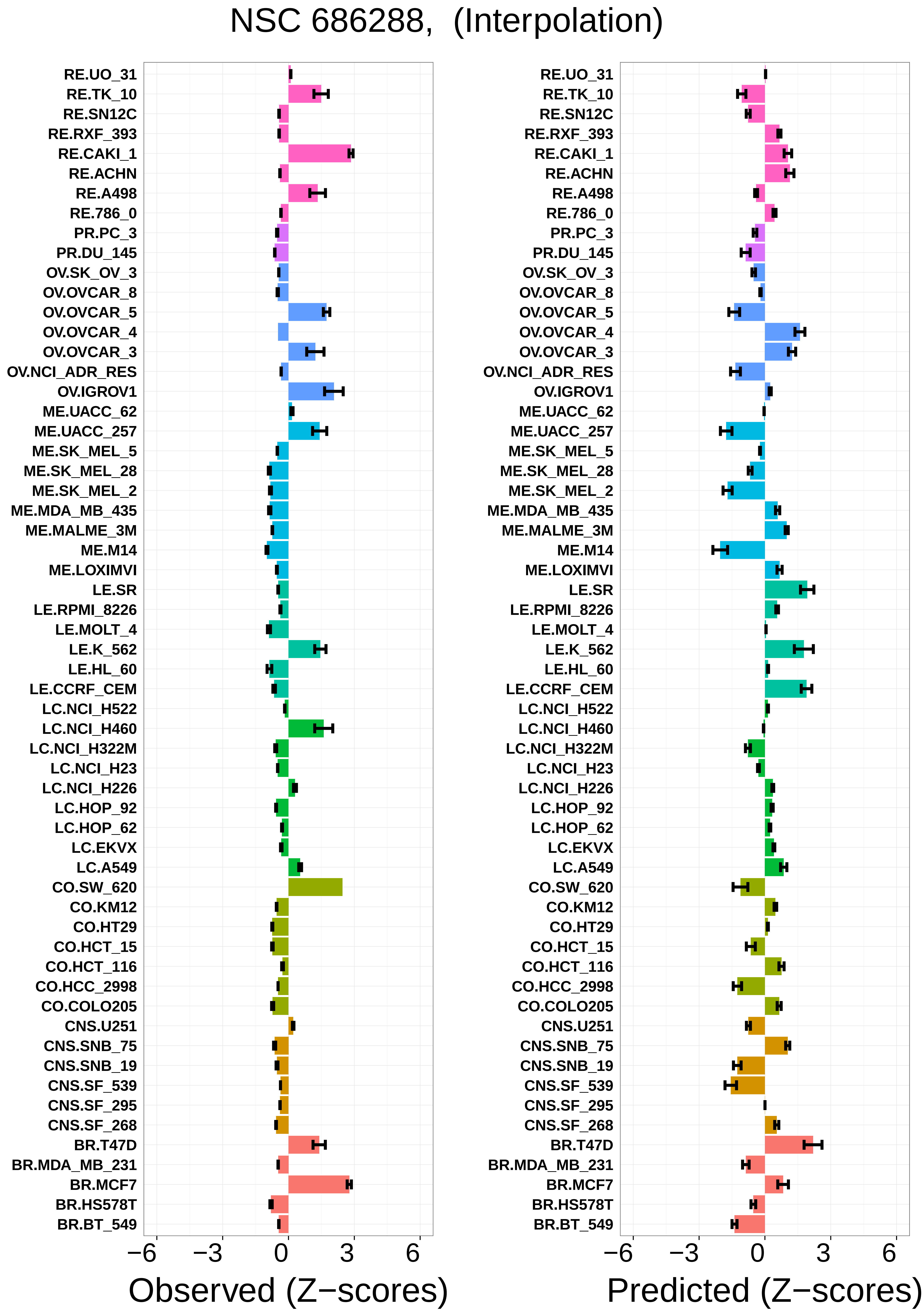
NSC 686288, (Interpolation)
RE.UO_31
RE.TK_10
RE.SN12C
RE.RXF_393
RE.CAKI_1
RE.ACHN
RE.A498
RE.786_0
PR.PC_3
PR.DU_145
OV.SK_OV_3
OV.OVCAR_8
OV.OVCAR_5
OV.OVCAR_4
OV.OVCAR_3
OV.NCI_ADR_RES
OV.IGROV1
ME.UACC_62
ME.UACC_257
ME.SK_MEL_5
ME.SK_MEL_28
ME.SK_MEL_2
ME.MDA_MB_435
ME.MALME_3M
ME.M14
ME.LOXIMVI
LE.SR
LE.RPMI_8226
LE.MOLT_4
LE.K_562
LE.HL_60
LE.CCRF_CEM
LC.NCI_H522
LC.NCI_H460
LC.NCI_H322M
LC.NCI_H23
LC.NCI_H226
LC.HOP_92
LC.HOP_62
LC.EKVX
LC.A549
CO.SW_620
CO.KM12
CO.HT29
CO.HCT_15
CO.HCT_116
CO.HCC_2998
CO.COLO205
CNS.U251
CNS.SNB_75
CNS.SNB_19
CNS.SF_539
CNS.SF_295
CNS.SF_268
BR.T47D
BR.MDA_MB_231
BR.MCF7
BR.HS578T
BR.BT_549
−6
−3
0
3
6
Observed (Z−scores)
Predicted (Z−scores)

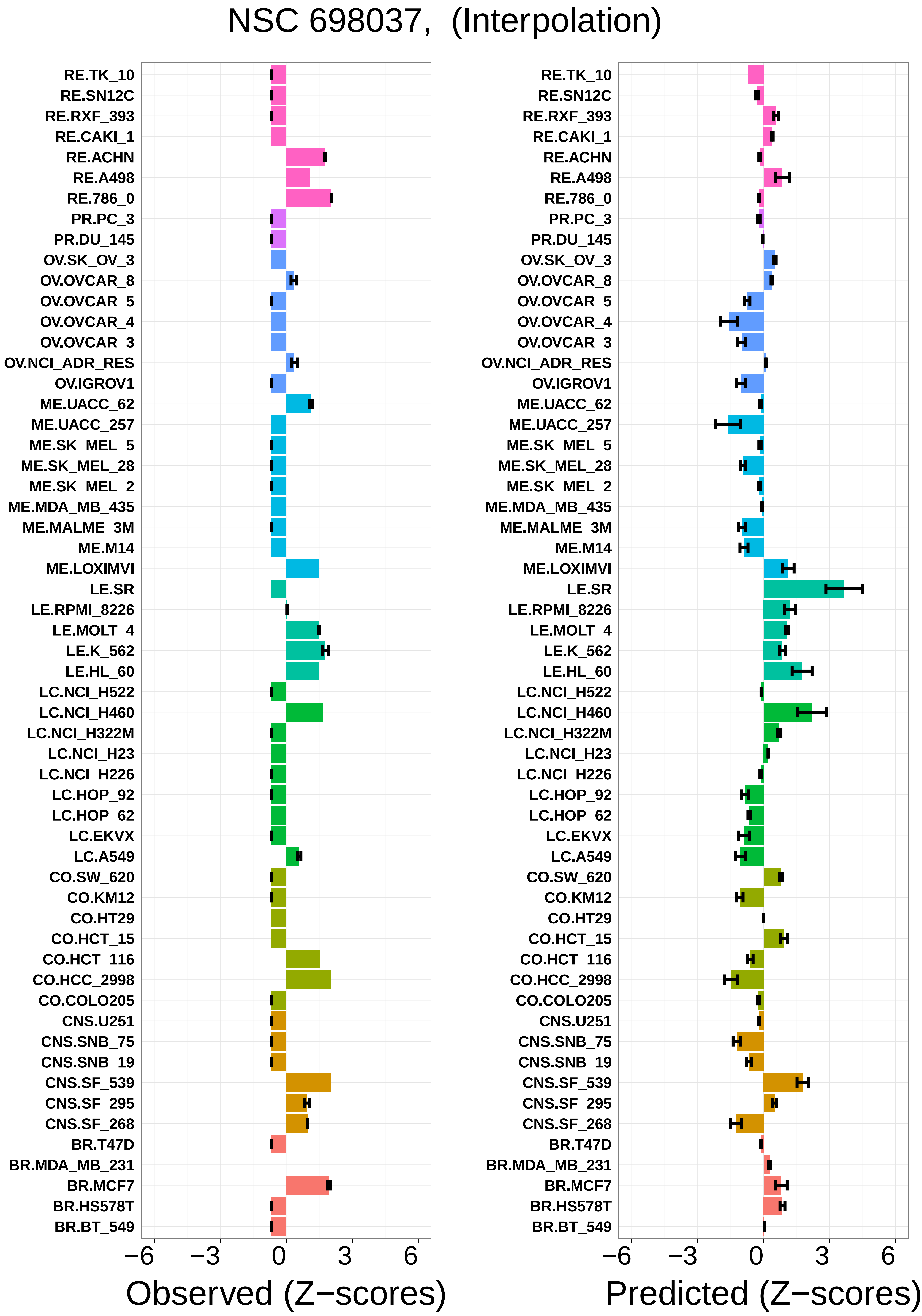
NSC 698037, (Interpolation)
RE.TK_10
RE.SN12C
RE.RXF_393
RE.CAKI_1
RE.ACHN
RE.A498
RE.786_0
PR.PC_3
PR.DU_145
OV.SK_OV_3
OV.OVCAR_8
OV.OVCAR_5
OV.OVCAR_4
OV.OVCAR_3
OV.NCI_ADR_RES
OV.IGROV1
ME.UACC_62
ME.UACC_257
ME.SK_MEL_5
ME.SK_MEL_28
ME.SK_MEL_2
ME.MDA_MB_435
ME.MALME_3M
ME.M14
ME.LOXIMVI
LE.SR
LE.RPMI_8226
LE.MOLT_4
LE.K_562
LE.HL_60
LC.NCI_H522
LC.NCI_H460
LC.NCI_H322M
LC.NCI_H23
LC.NCI_H226
LC.HOP_92
LC.HOP_62
LC.EKVX
LC.A549
CO.SW_620
CO.KM12
CO.HT29
CO.HCT_15
CO.HCT_116
CO.HCC_2998
CO.COLO205
CNS.U251
CNS.SNB_75
CNS.SNB_19
CNS.SF_539
CNS.SF_295
CNS.SF_268
BR.T47D
BR.MDA_MB_231
BR.MCF7
BR.HS578T
BR.BT_549
−6 −3 0 3 6
Observed (Z−scores)
Predicted (Z−scores)

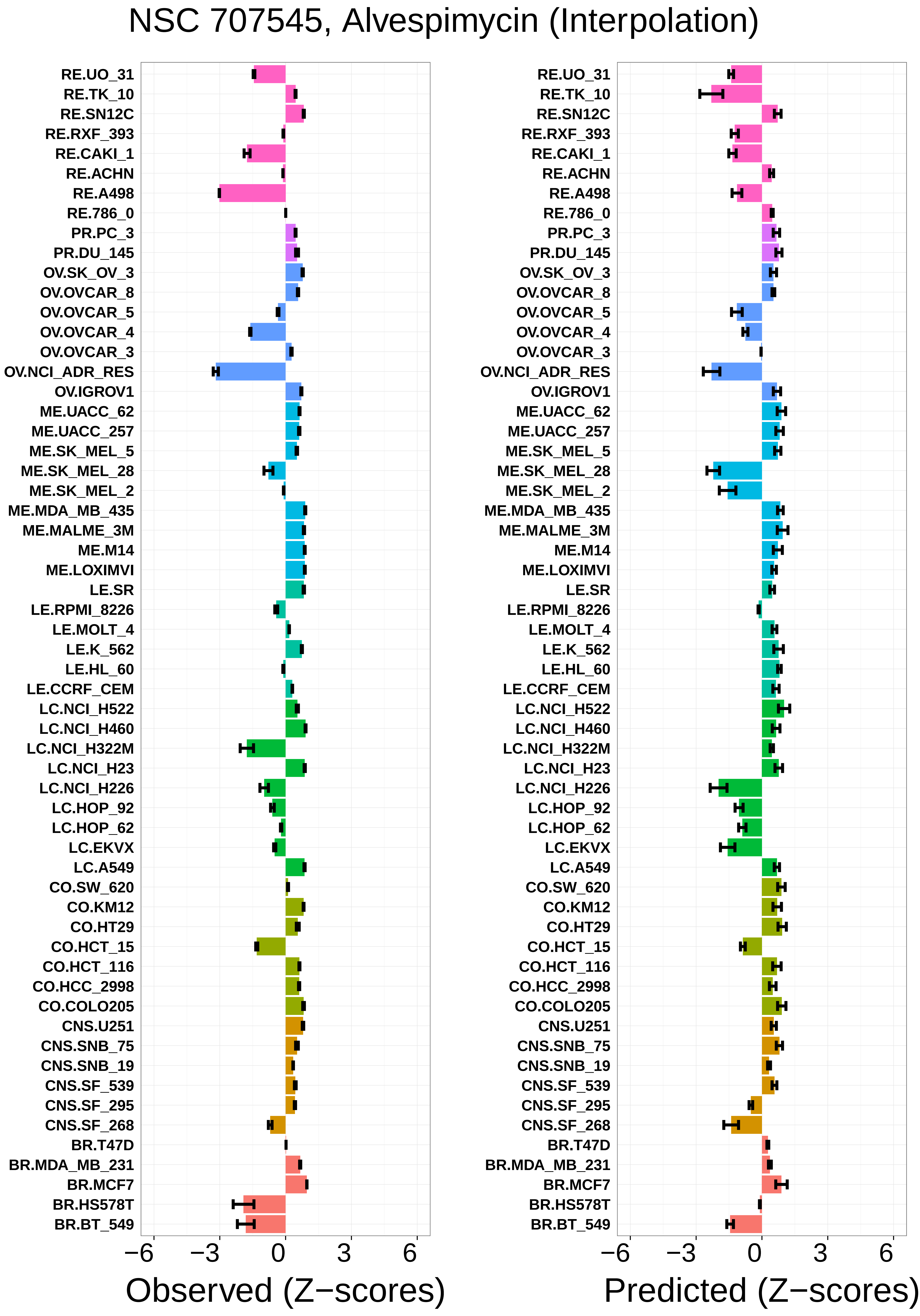
NSC 707545, Alvespimycin (Interpolation)
RE.UO_31
RE.TK_10
RE.SN12C
RE.RXF_393
RE.CAKI_1
RE.ACHN
RE.A498
RE.786_0
PR.PC_3
PR.DU_145
OV.SK_OV_3
OV.OVCAR_8
OV.OVCAR_5
OV.OVCAR_4
OV.OVCAR_3
OV.NCI_ADR_RES
OV.IGROV1
ME.UACC_62
ME.UACC_257
ME.SK_MEL_5
ME.SK_MEL_28
ME.SK_MEL_2
ME.MDA_MB_435
ME.MALME_3M
ME.M14
ME.LOXIMVI
LE.SR
LE.RPMI_8226
LE.MOLT_4
LE.K_562
LE.HL_60
LE.CCRF_CEM
LC.NCI_H522
LC.NCI_H460
LC.NCI_H322M
LC.NCI_H23
LC.NCI_H226
LC.HOP_92
LC.HOP_62
LC.EKVX
LC.A549
CO.SW_620
CO.KM12
CO.HT29
CO.HCT_15
CO.HCT_116
CO.HCC_2998
CO.COLO205
CNS.U251
CNS.SNB_75
CNS.SNB_19
CNS.SF_539
CNS.SF_295
CNS.SF_268
BR.T47D
BR.MDA_MB_231
BR.MCF7
BR.HS578T
BR.BT_549
−6
−3
0
3
6
Observed (Z−scores)
Predicted (Z−scores)

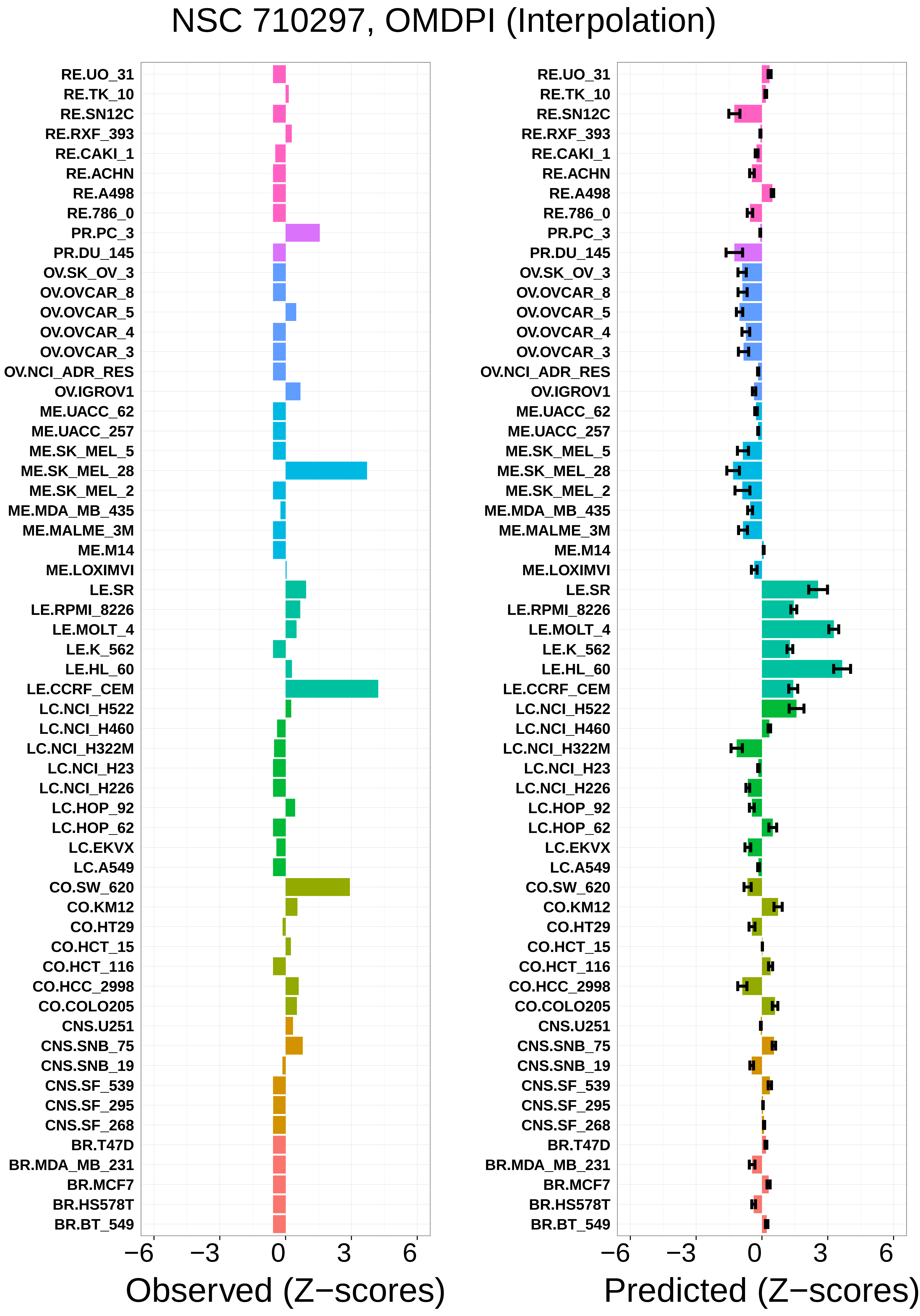
NSC 710297, OMDPI (Interpolation)
RE.UO_31
RE.TK_10
RE.SN12C
RE.RXF_393
RE.CAKI_1
RE.ACHN
RE.A498
RE.786_0
PR.PC_3
PR.DU_145
OV.SK_OV_3
OV.OVCAR_8
OV.OVCAR_5
OV.OVCAR_4
OV.OVCAR_3
OV.NCI_ADR_RES
OV.IGROV1
ME.UACC_62
ME.UACC_257
ME.SK_MEL_5
ME.SK_MEL_28
ME.SK_MEL_2
ME.MDA_MB_435
ME.MALME_3M
ME.M14
ME.LOXIMVI
LE.SR
LE.RPMI_8226
LE.MOLT_4
LE.K_562
LE.HL_60
LE.CCRF_CEM
LC.NCI_H522
LC.NCI_H460
LC.NCI_H322M
LC.NCI_H23
LC.NCI_H226
LC.HOP_92
LC.HOP_62
LC.EKVX
LC.A549
CO.SW_620
CO.KM12
CO.HT29
CO.HCT_15
CO.HCT_116
CO.HCC_2998
CO.COLO205
CNS.U251
CNS.SNB_75
CNS.SNB_19
CNS.SF_539
CNS.SF_295
CNS.SF_268
BR.T47D
BR.MDA_MB_231
BR.MCF7
BR.HS578T
BR.BT_549
−6 −3 0 3 6
Observed (Z−scores)
Predicted (Z−scores)

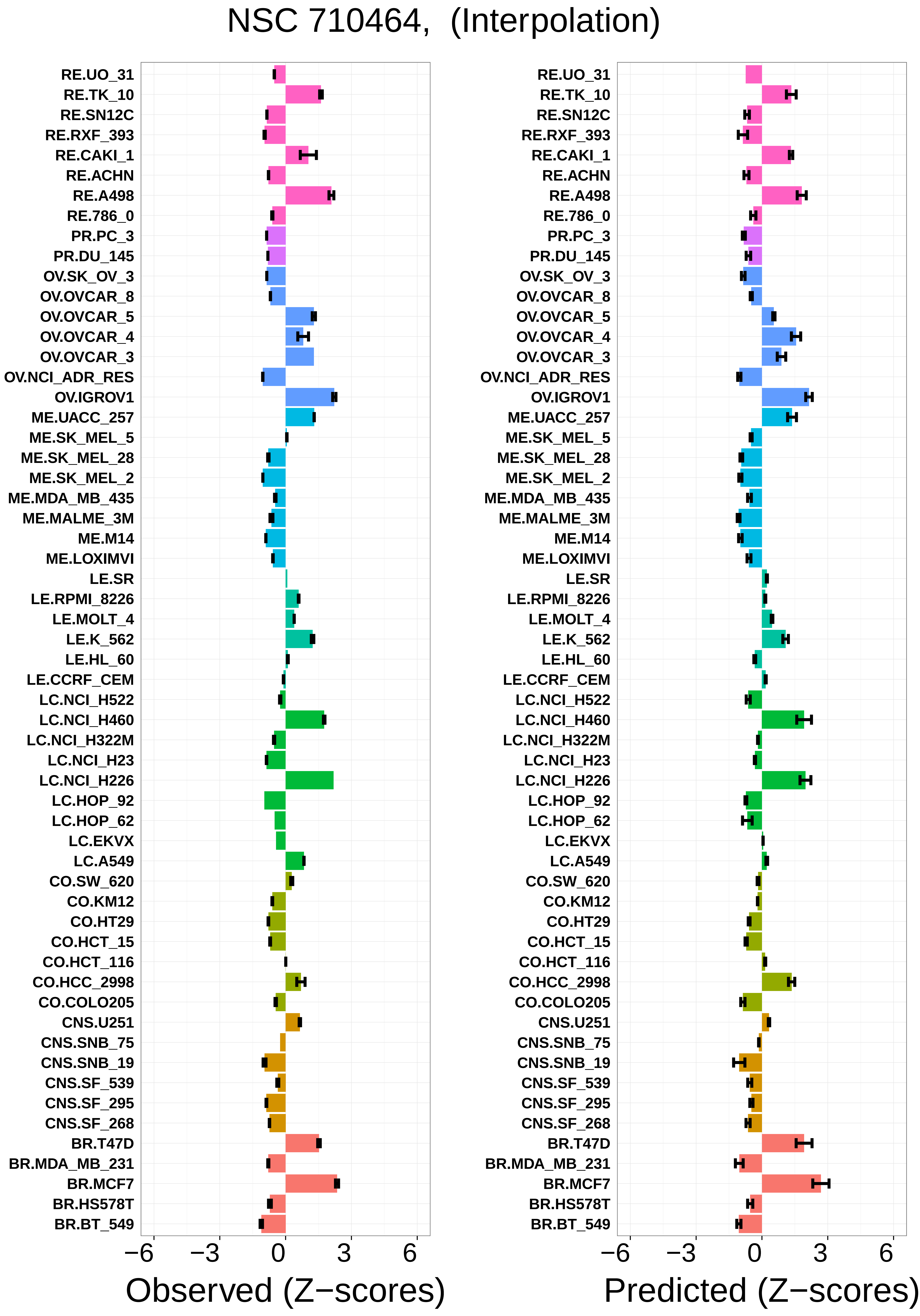

NSC 710464, (Interpolation)
RE.UO_31
RE.TK_10
RE.SN12C
RE.RXF_393
RE.CAKI_1
RE.ACHN
RE.A498
RE.786_0
PR.PC_3
PR.DU_145
OV.SK_OV_3
OV.OVCAR_8
OV.OVCAR_5
OV.OVCAR_4
OV.OVCAR_3
OV.NCI_ADR_RES
OV.IGROV1
ME.UACC_257
ME.SK_MEL_5
ME.SK_MEL_28
ME.SK_MEL_2
ME.MDA_MB_435
ME.MALME_3M
ME.M14
ME.LOXIMVI
LE.SR
LE.RPMI_8226
LE.MOLT_4
LE.K_562
LE.HL_60
LE.CCRF_CEM
LC.NCI_H522
LC.NCI_H460
LC.NCI_H322M
LC.NCI_H23
LC.NCI_H226
LC.HOP_92
LC.HOP_62
LC.EKVX
LC.A549
CO.SW_620
CO.KM12
CO.HT29
CO.HCT_15
CO.HCT_116
CO.HCC_2998
CO.COLO205
CNS.U251
CNS.SNB_75
CNS.SNB_19
CNS.SF_539
CNS.SF_295
CNS.SF_268
BR.T47D
BR.MDA_MB_231
BR.MCF7
BR.HS578T
BR.BT_549
−6
−3
0
3
6
Observed (Z−scores)
Predicted (Z−scores)

NSC 715055, Gefitinib (Interpolation)

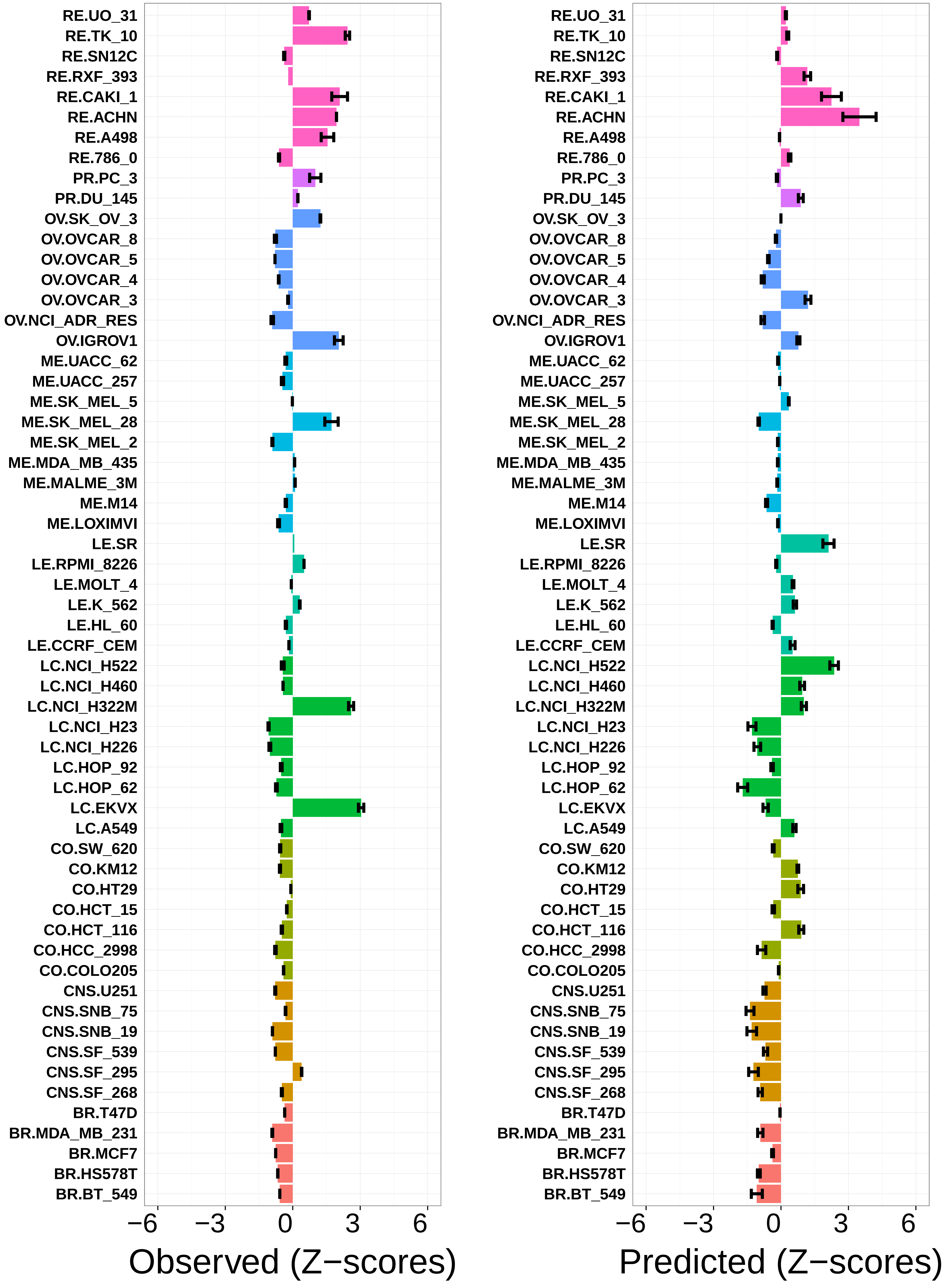

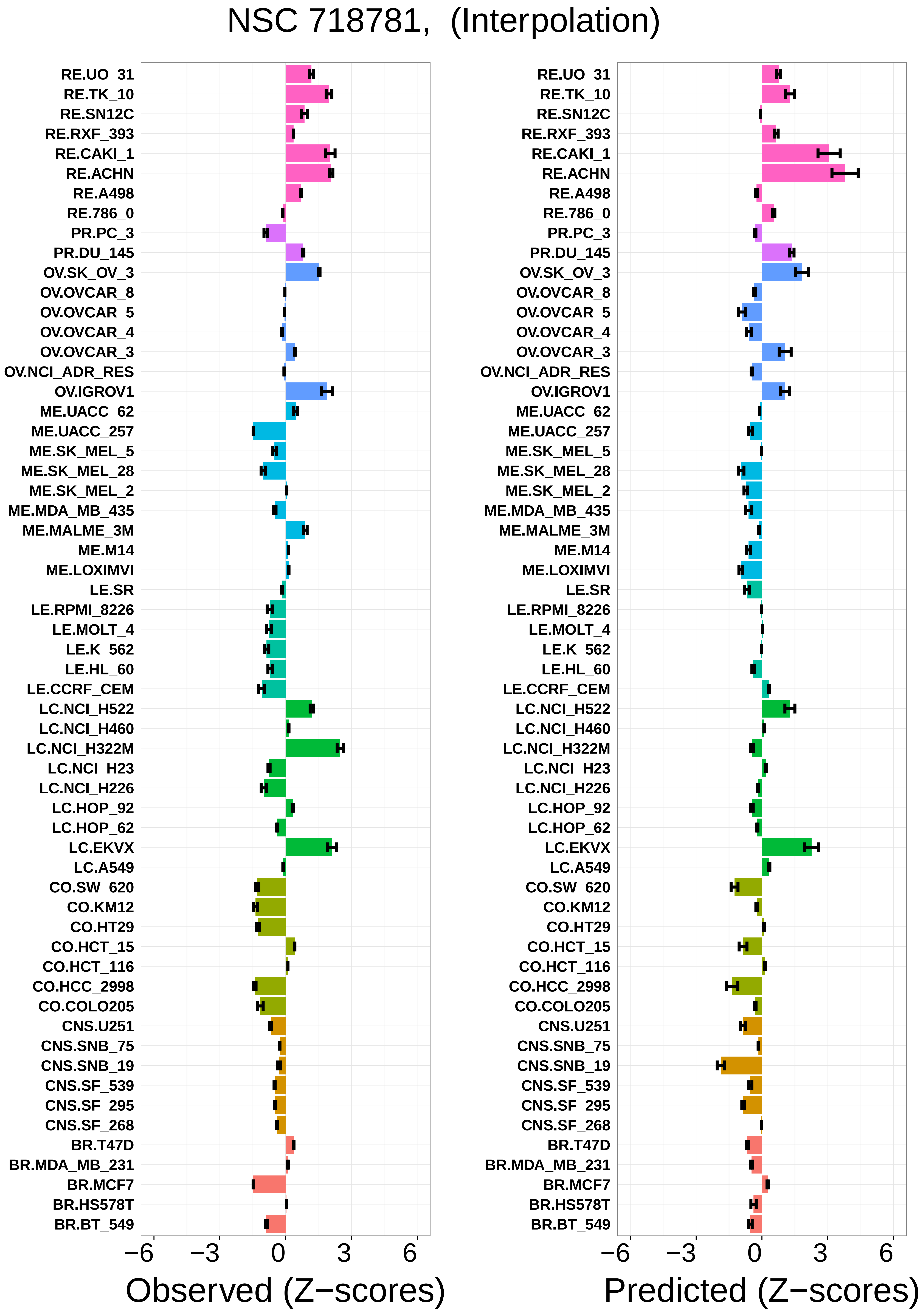

NSC 718781, (Interpolation)
RE.UO_31
RE.TK_10
RE.SN12C
RE.RXF_393
RE.CAKI_1
RE.ACHN
RE.A498
RE.786_0
PR.PC_3
PR.DU_145
OV.SK_OV_3
OV.OVCAR_8
OV.OVCAR_5
OV.OVCAR_4
OV.OVCAR_3
OV.NCI_ADR_RES
OV.IGROV1
ME.UACC_62
ME.UACC_257
ME.SK_MEL_5
ME.SK_MEL_28
ME.SK_MEL_2
ME.MDA_MB_435
ME.MALME_3M
ME.M14
ME.LOXIMVI
LE.SR
LE.RPMI_8226
LE.MOLT_4
LE.K_562
LE.HL_60
LE.CCRF_CEM
LC.NCI_H522
LC.NCI_H460
LC.NCI_H322M
LC.NCI_H23
LC.NCI_H226
LC.HOP_92
LC.HOP_62
LC.EKVX
LC.A549
CO.SW_620
CO.KM12
CO.HT29
CO.HCT_15
CO.HCT_116
CO.HCC_2998
CO.COLO205
CNS.U251
CNS.SNB_75
CNS.SNB_19
CNS.SF_539
CNS.SF_295
CNS.SF_268
BR.T47D
BR.MDA_MB_231
BR.MCF7
BR.HS578T
BR.BT_549
−6 −3 0 3 6
Observed (Z−scores)
Predicted (Z−scores)

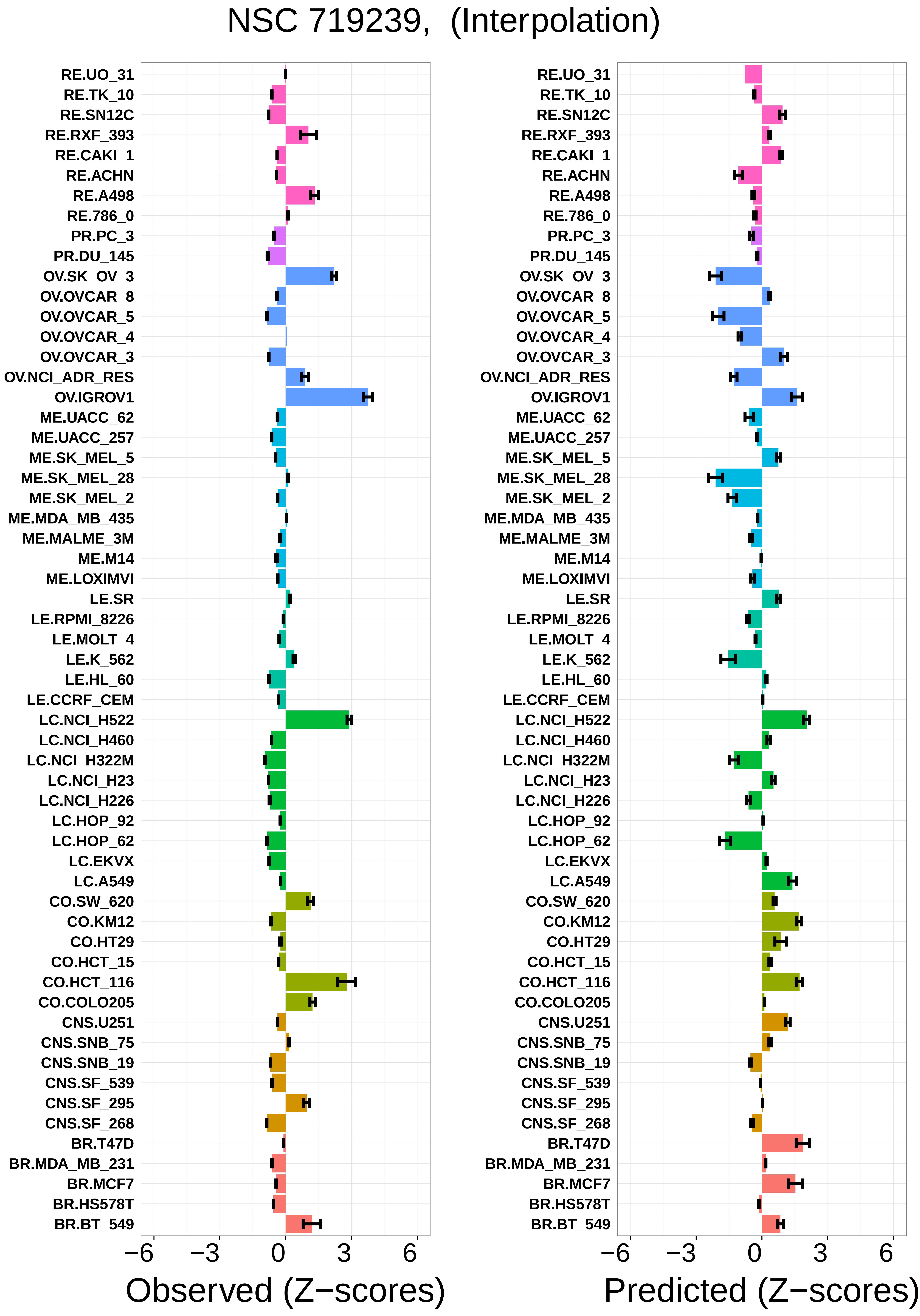

NSC 719239, (Interpolation)
RE.UO_31
RE.TK_10
RE.SN12C
RE.RXF_393
RE.CAKI_1
RE.ACHN
RE.A498
RE.786_0
PR.PC_3
PR.DU_145
OV.SK_OV_3
OV.OVCAR_8
OV.OVCAR_5
OV.OVCAR_4
OV.OVCAR_3
OV.NCI_ADR_RES
OV.IGROV1
ME.UACC_62
ME.UACC_257
ME.SK_MEL_5
ME.SK_MEL_28
ME.SK_MEL_2
ME.MDA_MB_435
ME.MALME_3M
ME.M14
ME.LOXIMVI
LE.SR
LE.RPMI_8226
LE.MOLT_4
LE.K_562
LE.HL_60
LE.CCRF_CEM
LC.NCI_H522
LC.NCI_H460
LC.NCI_H322M
LC.NCI_H23
LC.NCI_H226
LC.HOP_92
LC.HOP_62
LC.EKVX
LC.A549
CO.SW_620
CO.KM12
CO.HT29
CO.HCT_15
CO.HCT_116
CO.COLO205
CNS.U251
CNS.SNB_75
CNS.SNB_19
CNS.SF_539
CNS.SF_295
CNS.SF_268
BR.T47D
BR.MDA_MB_231
BR.MCF7
BR.HS578T
BR.BT_549
−6
−3
0
3
6
Observed (Z−scores)
Predicted (Z−scores)

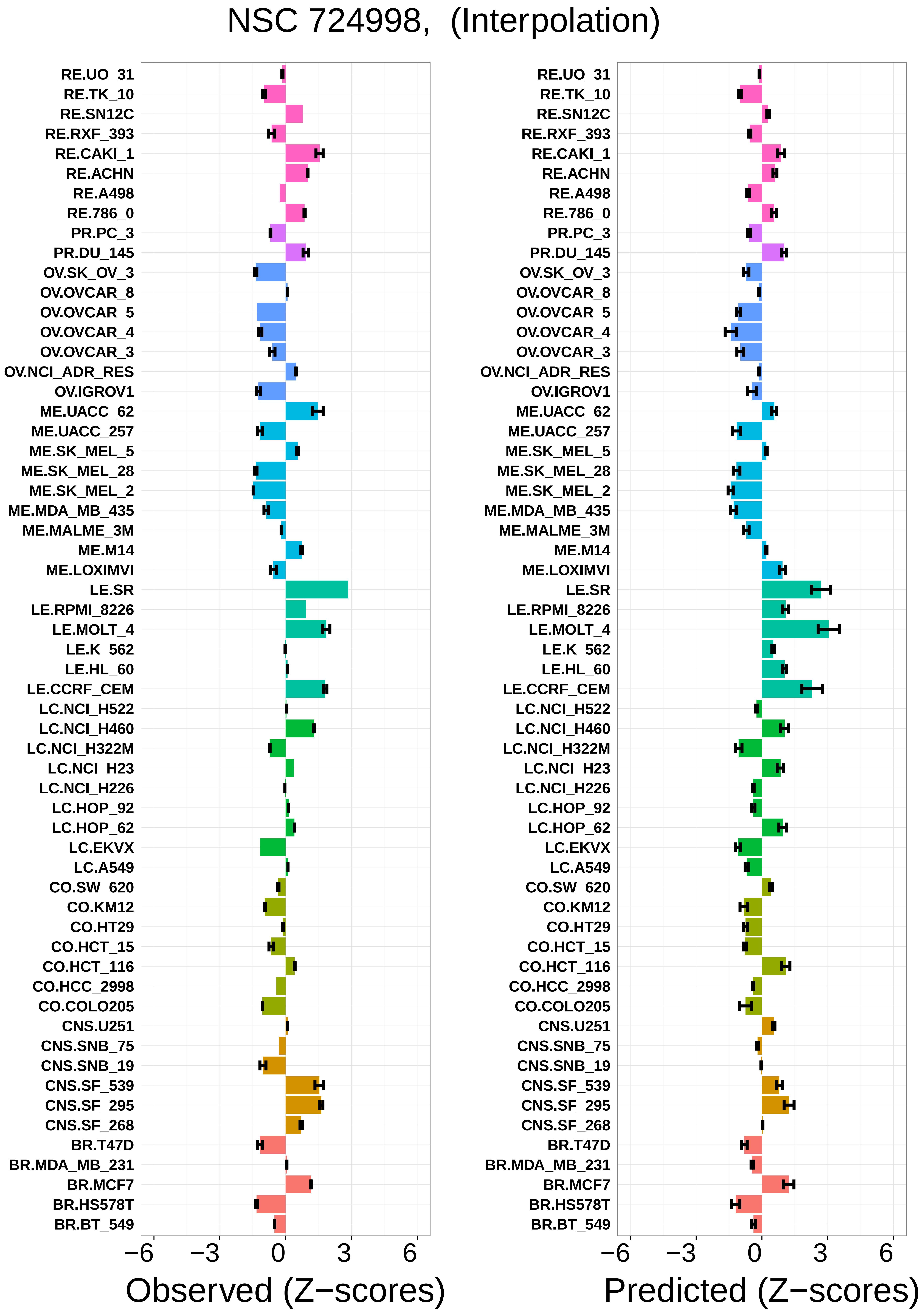

NSC 724998, (Interpolation)
RE.UO_31
RE.TK_10
RE.SN12C
RE.RXF_393
RE.CAKI_1
RE.ACHN
RE.A498
RE.786_0
PR.PC_3
PR.DU_145
OV.SK_OV_3
OV.OVCAR_8
OV.OVCAR_5
OV.OVCAR_4
OV.OVCAR_3
OV.NCI_ADR_RES
OV.IGROV1
ME.UACC_62
ME.UACC_257
ME.SK_MEL_5
ME.SK_MEL_28
ME.SK_MEL_2
ME.MDA_MB_435
ME.MALME_3M
ME.M14
ME.LOXIMVI
LE.SR
LE.RPMI_8226
LE.MOLT_4
LE.K_562
LE.HL_60
LE.CCRF_CEM
LC.NCI_H522
LC.NCI_H460
LC.NCI_H322M
LC.NCI_H23
LC.NCI_H226
LC.HOP_92
LC.HOP_62
LC.EKVX
LC.A549
CO.SW_620
CO.KM12
CO.HT29
CO.HCT_15
CO.HCT_116
CO.HCC_2998
CO.COLO205
CNS.U251
CNS.SNB_75
CNS.SNB_19
CNS.SF_539
CNS.SF_295
CNS.SF_268
BR.T47D
BR.MDA_MB_231
BR.MCF7
BR.HS578T
BR.BT_549
−6
−3
0
3
6
Observed (Z−scores)
Predicted (Z−scores)

NSC 725776, (Interpolation)

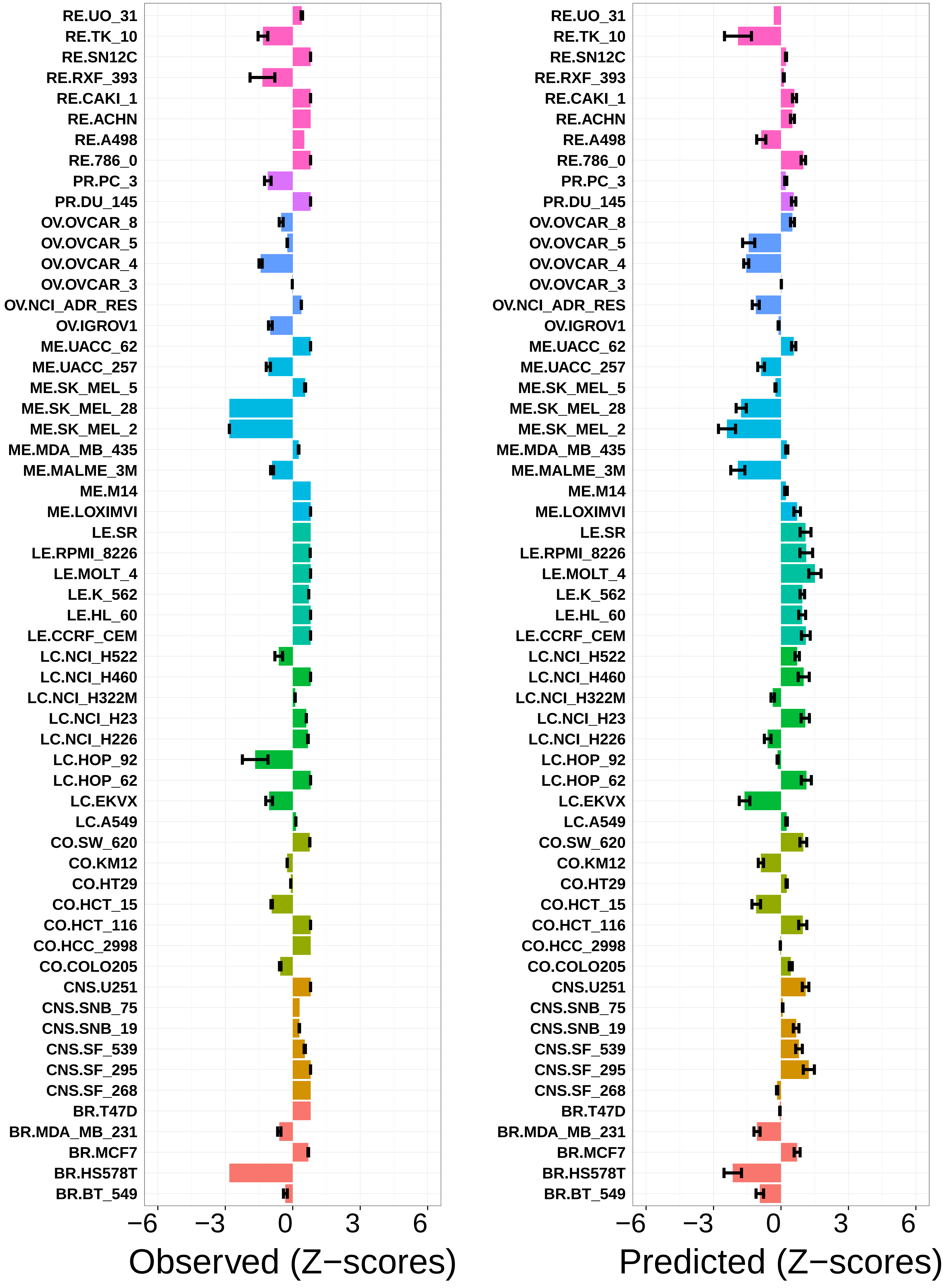

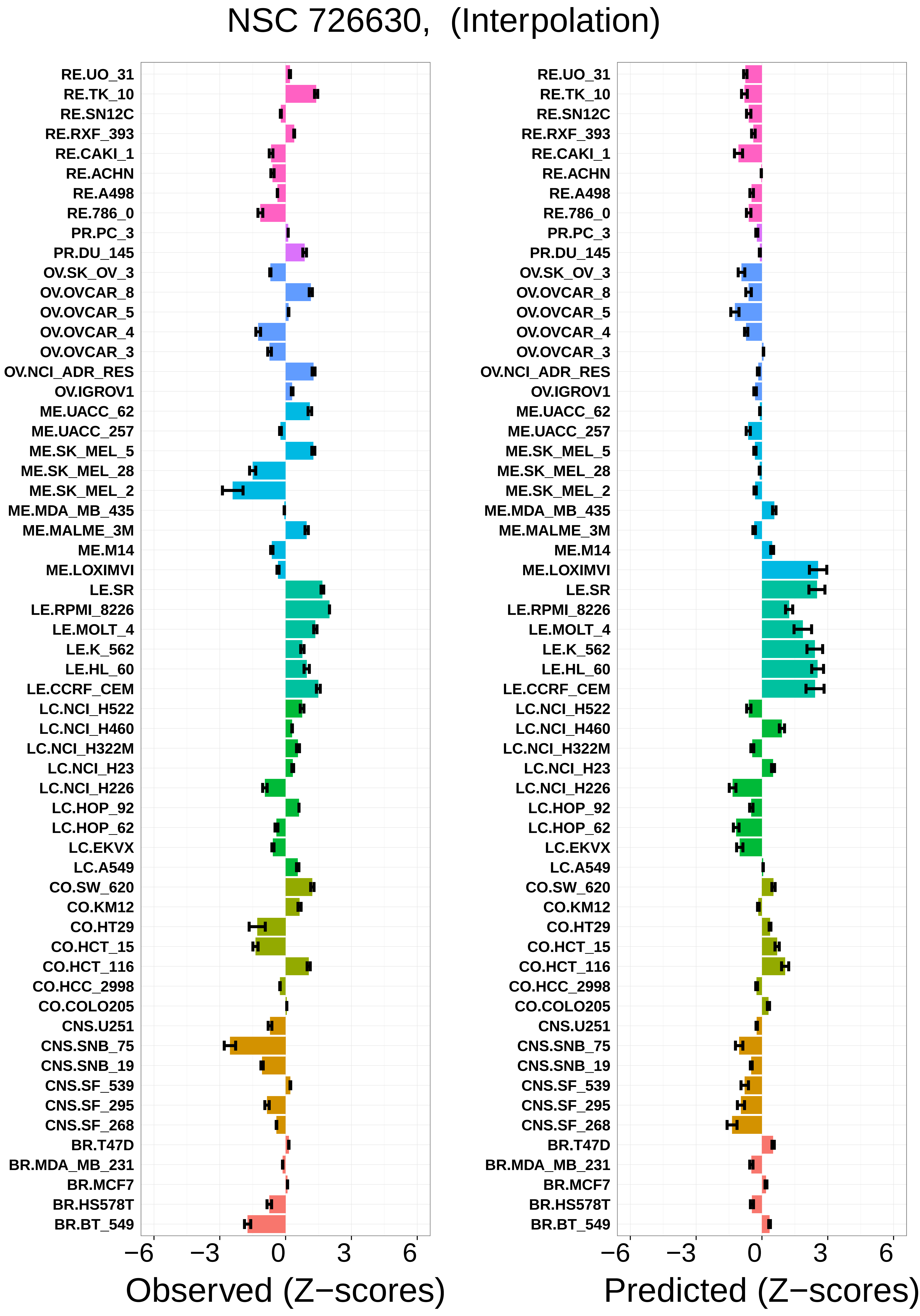

NSC 726630, (Interpolation)
RE.UO_31
RE.TK_10
RE.SN12C
RE.RXF_393
RE.CAKI_1
RE.ACHN
RE.A498
RE.786_0
PR.PC_3
PR.DU_145
OV.SK_OV_3
OV.OVCAR_8
OV.OVCAR_5
OV.OVCAR_4
OV.OVCAR_3
OV.NCI_ADR_RES
OV.IGROV1
ME.UACC_62
ME.UACC_257
ME.SK_MEL_5
ME.SK_MEL_28
ME.SK_MEL_2
ME.MDA_MB_435
ME.MALME_3M
ME.M14
ME.LOXIMVI
LE.SR
LE.RPMI_8226
LE.MOLT_4
LE.K_562
LE.HL_60
LE.CCRF_CEM
LC.NCI_H522
LC.NCI_H460
LC.NCI_H322M
LC.NCI_H23
LC.NCI_H226
LC.HOP_92
LC.HOP_62
LC.EKVX
LC.A549
CO.SW_620
CO.KM12
CO.HT29
CO.HCT_15
CO.HCT_116
CO.HCC_2998
CO.COLO205
CNS.U251
CNS.SNB_75
CNS.SNB_19
CNS.SF_539
CNS.SF_295
CNS.SF_268
BR.T47D
BR.MDA_MB_231
BR.MCF7
BR.HS578T
BR.BT_549
−6 −3 0 3 6
Observed (Z−scores)
Predicted (Z−scores)

NSC 728073, (Interpolation)

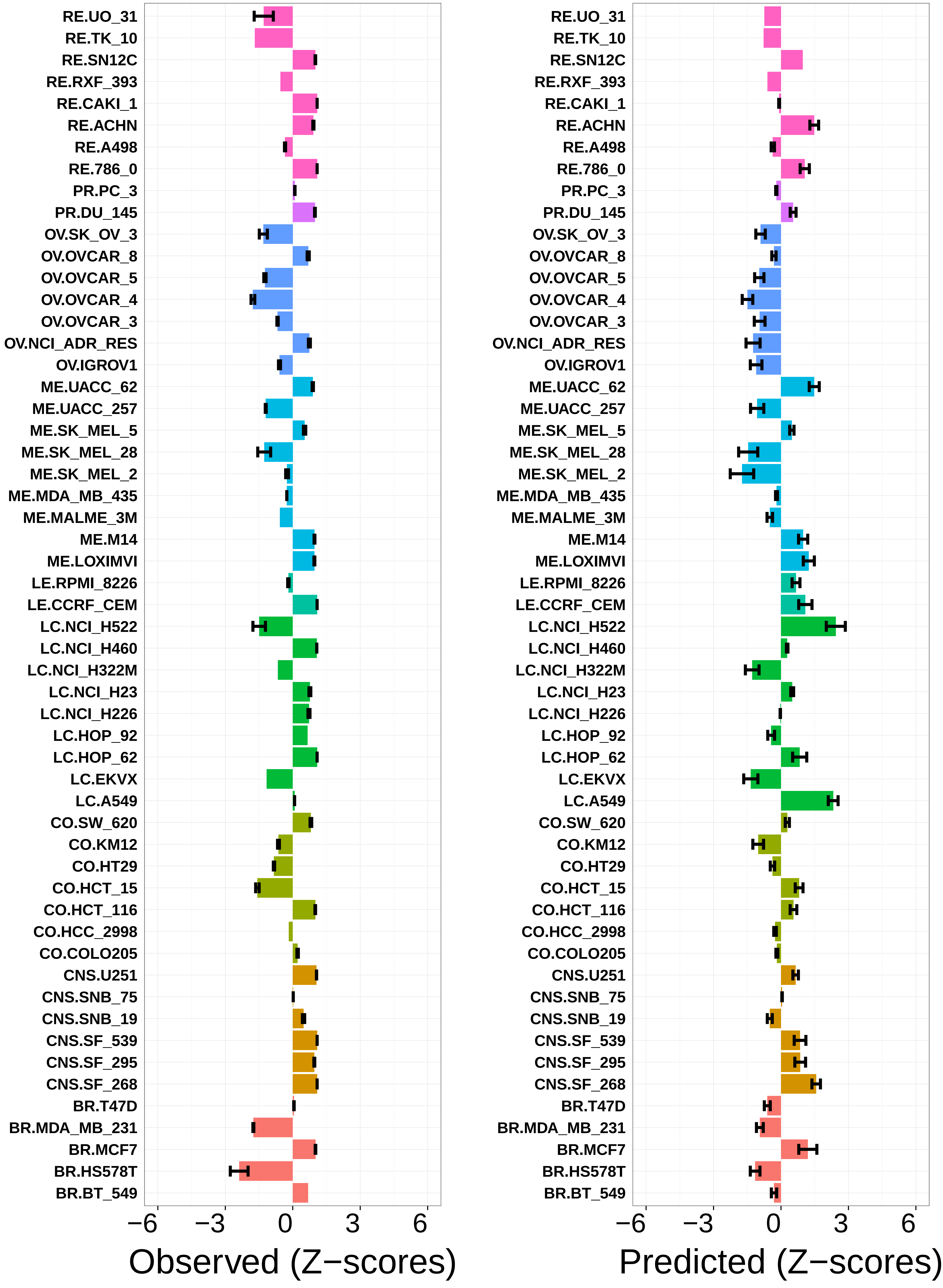

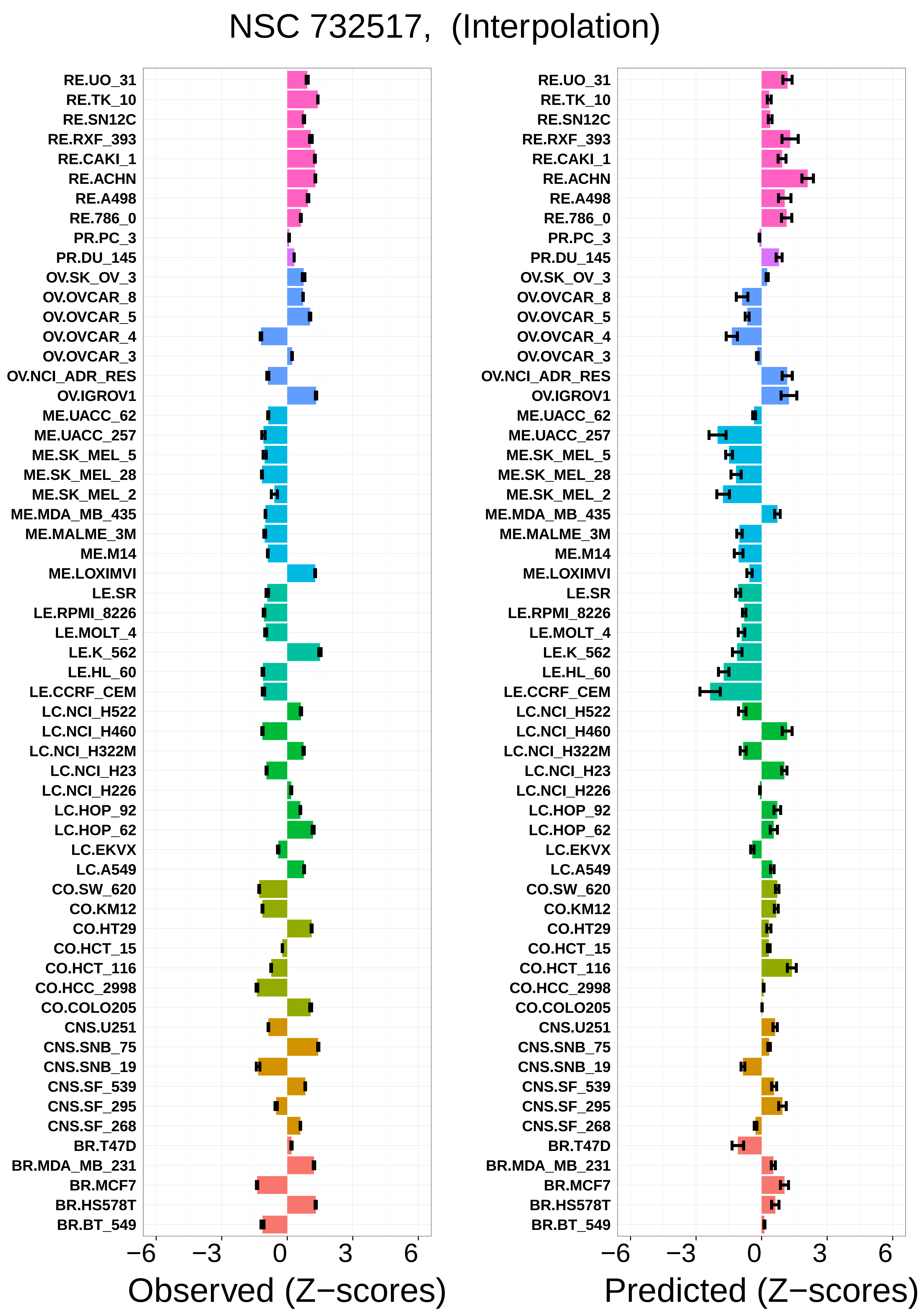
NSC 732517, (Interpolation)
RE.UO_31
RE.TK_10
RE.SN12C
RE.RXF_393
RE.CAKI_1
RE.ACHN
RE.A498
RE.786_0
PR.PC_3
PR.DU_145
OV.SK_OV_3
OV.OVCAR_8
OV.OVCAR_5
OV.OVCAR_4
OV.OVCAR_3
OV.NCI_ADR_RES
OV.IGROV1
ME.UACC_62
ME.UACC_257
ME.SK_MEL_5
ME.SK_MEL_28
ME.SK_MEL_2
ME.MDA_MB_435
ME.MALME_3M
ME.M14
ME.LOXIMVI
LE.SR
LE.RPMI_8226
LE.MOLT_4
LE.K_562
LE.HL_60
LE.CCRF_CEM
LC.NCI_H522
LC.NCI_H460
LC.NCI_H322M
LC.NCI_H23
LC.NCI_H226
LC.HOP_92
LC.HOP_62
LC.EKVX
LC.A549
CO.SW_620
CO.KM12
CO.HT29
CO.HCT_15
CO.HCT_116
CO.HCC_2998
CO.COLO205
CNS.U251
CNS.SNB_75
CNS.SNB_19
CNS.SF_539
CNS.SF_295
CNS.SF_268
BR.T47D
BR.MDA_MB_231
BR.MCF7
BR.HS578T
BR.BT_549
−6 −3 0 3 6
Observed (Z−scores)
Predicted (Z−scores)

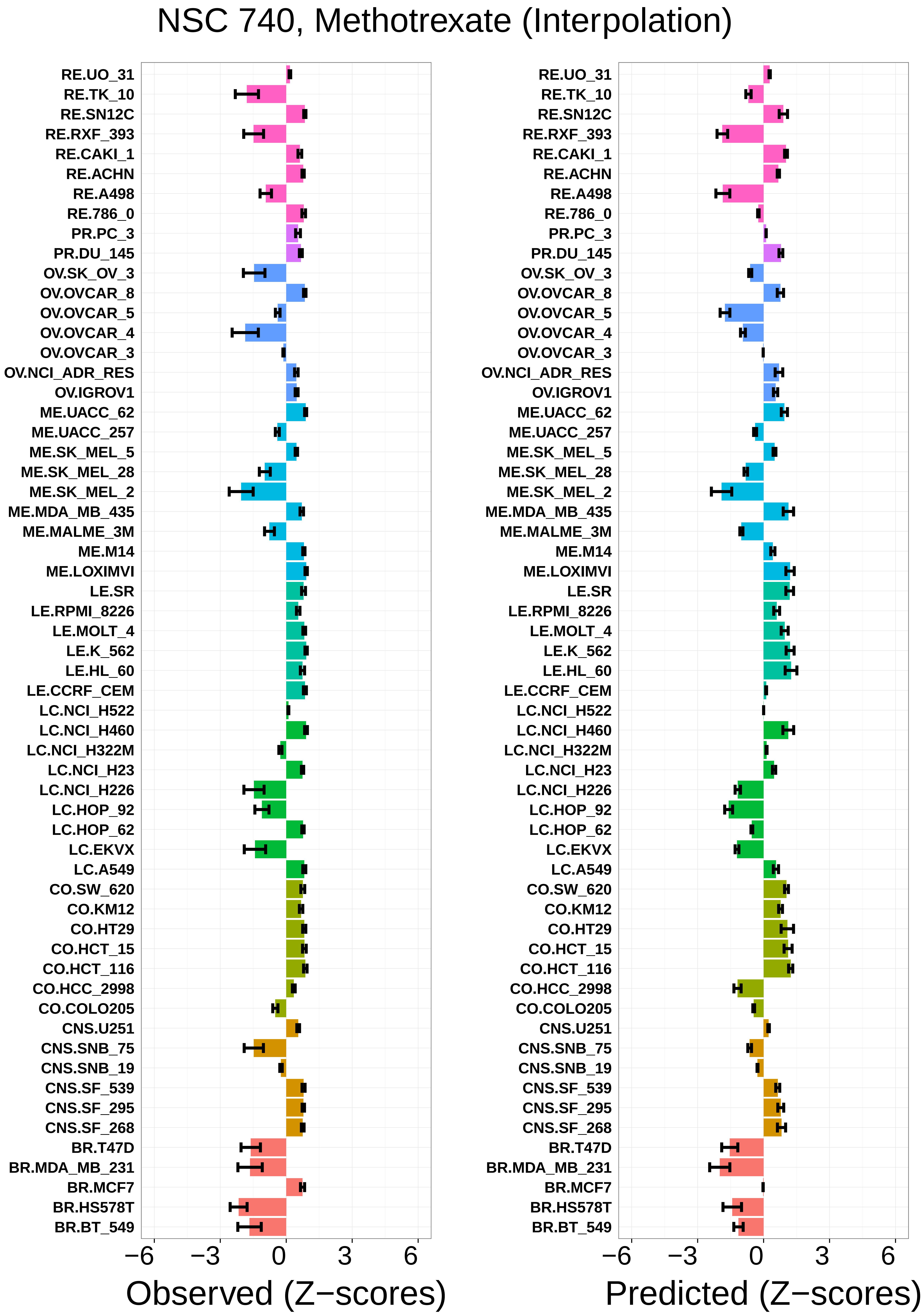
NSC 740, Methotrexate (Interpolation)
RE.UO_31
RE.TK_10
RE.SN12C
RE.RXF_393
RE.CAKI_1
RE.ACHN
RE.A498
RE.786_0
PR.PC_3
PR.DU_145
OV.SK_OV_3
OV.OVCAR_8
OV.OVCAR_5
OV.OVCAR_4
OV.OVCAR_3
OV.NCI_ADR_RES
OV.IGROV1
ME.UACC_62
ME.UACC_257
ME.SK_MEL_5
ME.SK_MEL_28
ME.SK_MEL_2
ME.MDA_MB_435
ME.MALME_3M
ME.M14
ME.LOXIMVI
LE.SR
LE.RPMI_8226
LE.MOLT_4
LE.K_562
LE.HL_60
LE.CCRF_CEM
LC.NCI_H522
LC.NCI_H460
LC.NCI_H322M
LC.NCI_H23
LC.NCI_H226
LC.HOP_92
LC.HOP_62
LC.EKVX
LC.A549
CO.SW_620
CO.KM12
CO.HT29
CO.HCT_15
CO.HCT_116
CO.HCC_2998
CO.COLO205
CNS.U251
CNS.SNB_75
CNS.SNB_19
CNS.SF_539
CNS.SF_295
CNS.SF_268
BR.T47D
BR.MDA_MB_231
BR.MCF7
BR.HS578T
BR.BT_549
−6
−3
0
3
6
Observed (Z−scores)
Predicted (Z−scores)

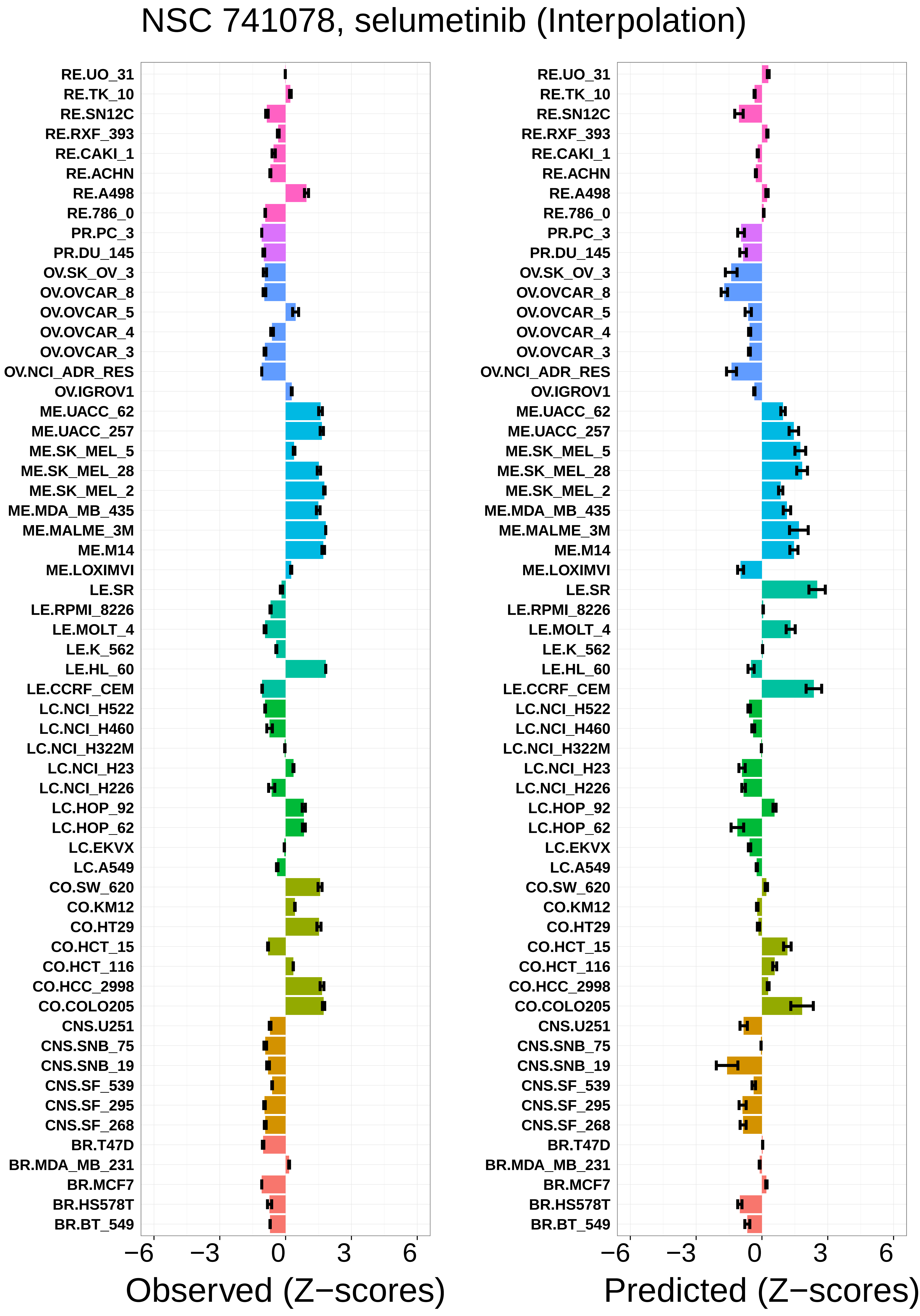

NSC 741078, selumetinib (Interpolation)
RE.UO_31
RE.TK_10
RE.SN12C
RE.RXF_393
RE.CAKI_1
RE.ACHN
RE.A498
RE.786_0
PR.PC_3
PR.DU_145
OV.SK_OV_3
OV.OVCAR_8
OV.OVCAR_5
OV.OVCAR_4
OV.OVCAR_3
OV.NCI_ADR_RES
OV.IGROV1
ME.UACC_62
ME.UACC_257
ME.SK_MEL_5
ME.SK_MEL_28
ME.SK_MEL_2
ME.MDA_MB_435
ME.MALME_3M
ME.M14
ME.LOXIMVI
LE.SR
LE.RPMI_8226
LE.MOLT_4
LE.K_562
LE.HL_60
LE.CCRF_CEM
LC.NCI_H522
LC.NCI_H460
LC.NCI_H322M
LC.NCI_H23
LC.NCI_H226
LC.HOP_92
LC.HOP_62
LC.EKVX
LC.A549
CO.SW_620
CO.KM12
CO.HT29
CO.HCT_15
CO.HCT_116
CO.HCC_2998
CO.COLO205
CNS.U251
CNS.SNB_75
CNS.SNB_19
CNS.SF_539
CNS.SF_295
CNS.SF_268
BR.T47D
BR.MDA_MB_231
BR.MCF7
BR.HS578T
BR.BT_549
−6
−3
0
3
6
Observed (Z−scores)
Predicted (Z−scores)

NSC 745750, Tykerb (Interpolation)

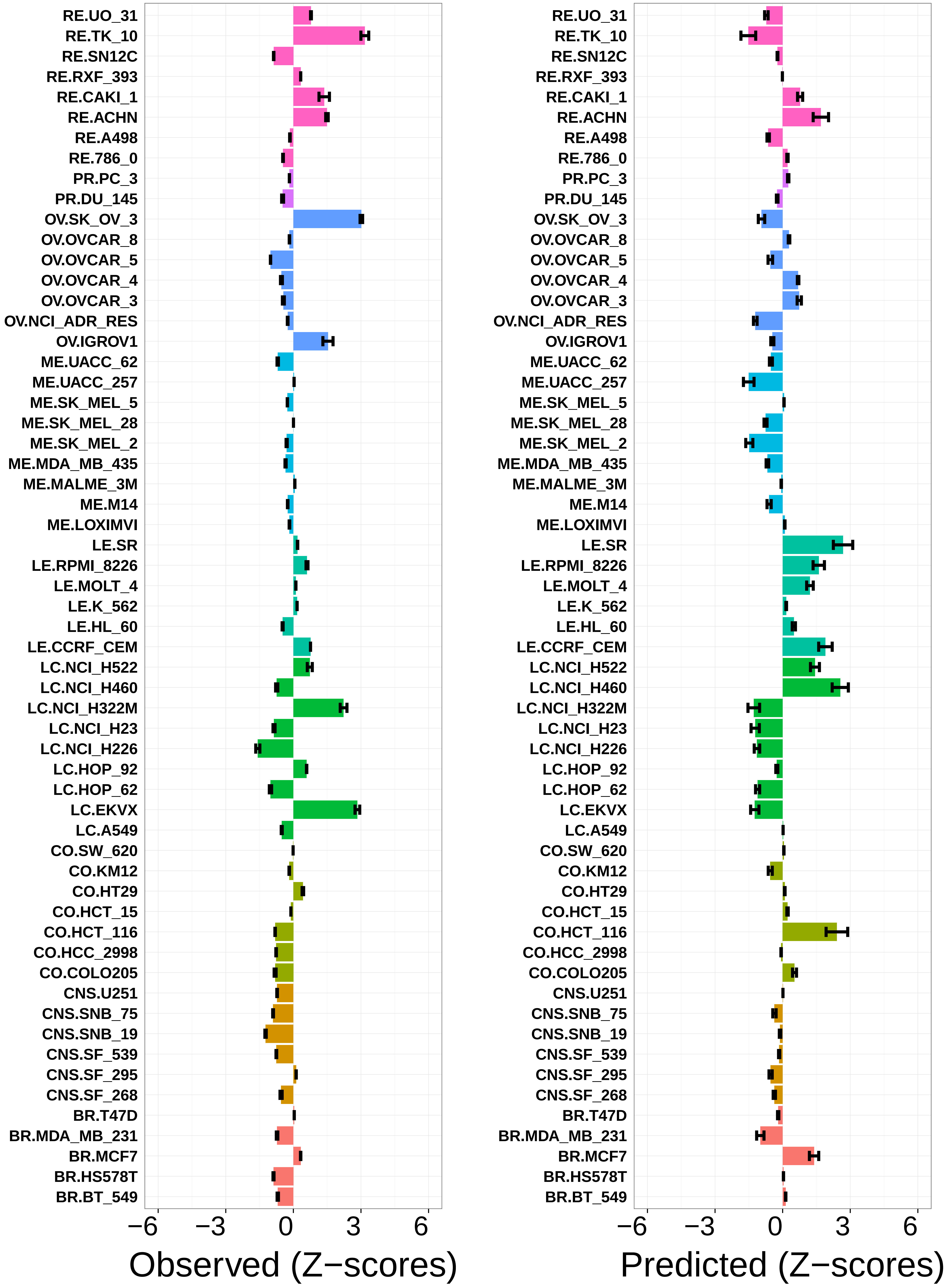

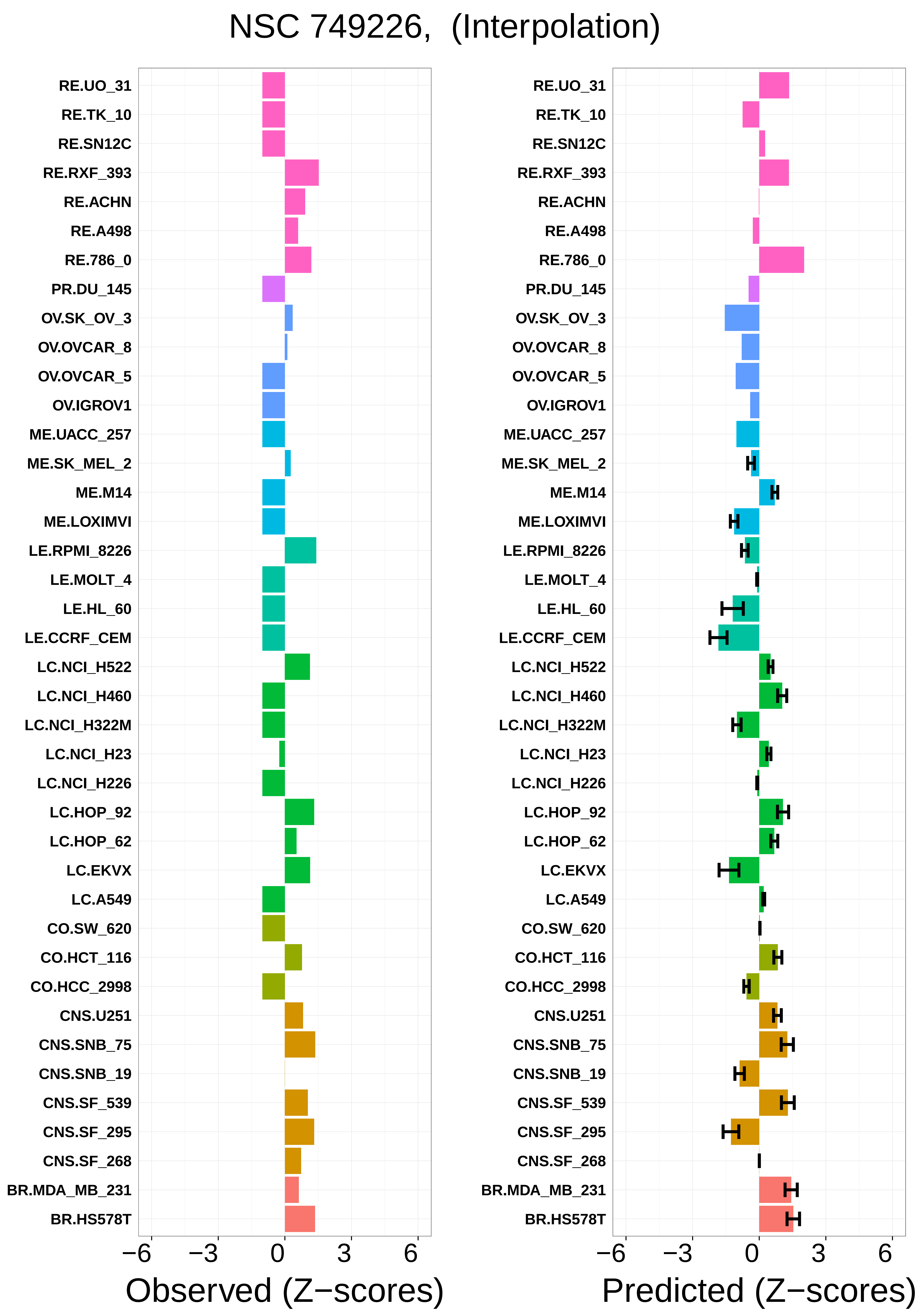

NSC 749226, (Interpolation)
RE.UO_31
RE.TK_10
RE.SN12C
RE.RXF_393
RE.ACHN
RE.A498
RE.786_0
PR.DU_145
OV.SK_OV_3
OV.OVCAR_8
OV.OVCAR_5
OV.IGROV1
ME.UACC_257
ME.SK_MEL_2
ME.M14
ME.LOXIMVI
LE.RPMI_8226
LE.MOLT_4
LE.HL_60
LE.CCRF_CEM
LC.NCI_H522
LC.NCI_H460
LC.NCI_H322M
LC.NCI_H23
LC.NCI_H226
LC.HOP_92
LC.HOP_62
LC.EKVX
LC.A549
CO.SW_620
CO.HCT_116
CO.HCC_2998
CNS.U251
CNS.SNB_75
CNS.SNB_19
CNS.SF_539
CNS.SF_295
CNS.SF_268
BR.MDA_MB_231
BR.HS578T
−6 −3 0 3 6
Observed (Z−scores)
Predicted (Z−scores)

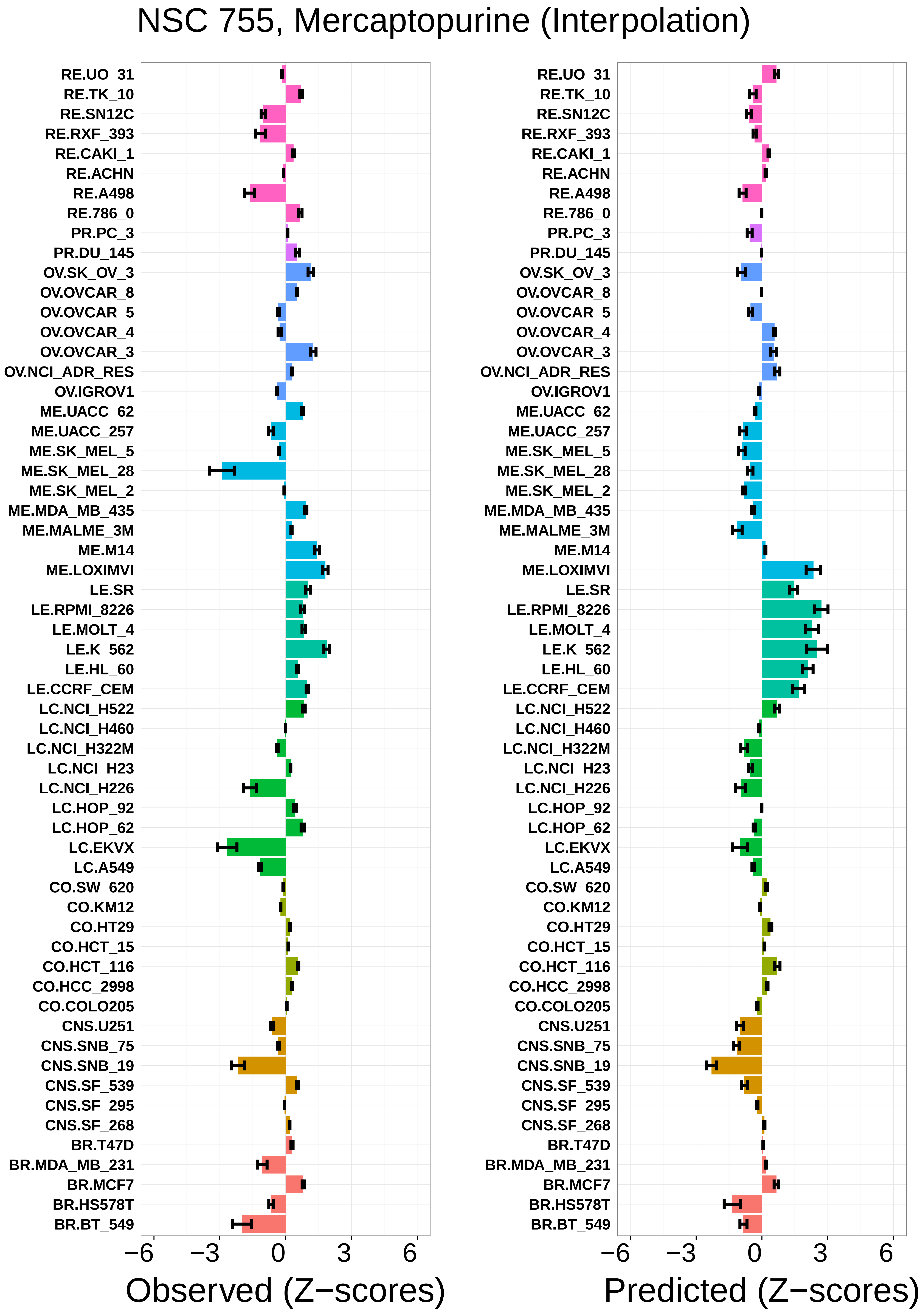
NSC 755, Mercaptopurine (Interpolation)
RE.UO_31
RE.TK_10
RE.SN12C
RE.RXF_393
RE.CAKI_1
RE.ACHN
RE.A498
RE.786_0
PR.PC_3
PR.DU_145
OV.SK_OV_3
OV.OVCAR_8
OV.OVCAR_5
OV.OVCAR_4
OV.OVCAR_3
OV.NCI_ADR_RES
OV.IGROV1
ME.UACC_62
ME.UACC_257
ME.SK_MEL_5
ME.SK_MEL_28
ME.SK_MEL_2
ME.MDA_MB_435
ME.MALME_3M
ME.M14
ME.LOXIMVI
LE.SR
LE.RPMI_8226
LE.MOLT_4
LE.K_562
LE.HL_60
LE.CCRF_CEM
LC.NCI_H522
LC.NCI_H460
LC.NCI_H322M
LC.NCI_H23
LC.NCI_H226
LC.HOP_92
LC.HOP_62
LC.EKVX
LC.A549
CO.SW_620
CO.KM12
CO.HT29
CO.HCT_15
CO.HCT_116
CO.HCC_2998
CO.COLO205
CNS.U251
CNS.SNB_75
CNS.SNB_19
CNS.SF_539
CNS.SF_295
CNS.SF_268
BR.T47D
BR.MDA_MB_231
BR.MCF7
BR.HS578T
BR.BT_549
−6 −3 0 3 6
Observed (Z−scores)
Predicted (Z−scores)

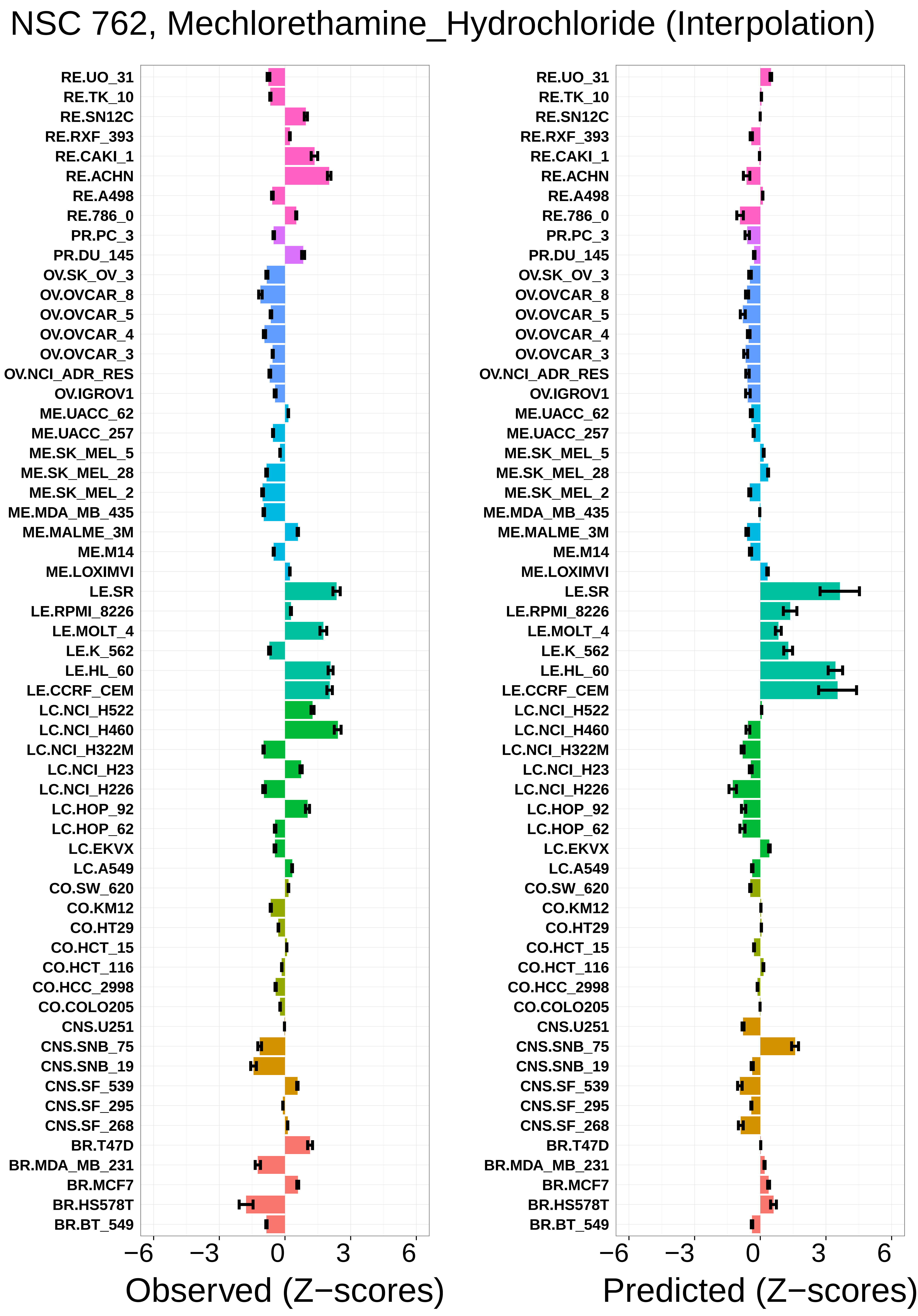
NSC 762, Mechlorethamine_Hydrochloride (Interpolation)
RE.UO_31
RE.TK_10
RE.SN12C
RE.RXF_393
RE.CAKI_1
RE.ACHN
RE.A498
RE.786_0
PR.PC_3
PR.DU_145
OV.SK_OV_3
OV.OVCAR_8
OV.OVCAR_5
OV.OVCAR_4
OV.OVCAR_3
OV.NCI_ADR_RES
OV.IGROV1
ME.UACC_62
ME.UACC_257
ME.SK_MEL_5
ME.SK_MEL_28
ME.SK_MEL_2
ME.MDA_MB_435
ME.MALME_3M
ME.M14
ME.LOXIMVI
LE.SR
LE.RPMI_8226
LE.MOLT_4
LE.K_562
LE.HL_60
LE.CCRF_CEM
LC.NCI_H522
LC.NCI_H460
LC.NCI_H322M
LC.NCI_H23
LC.NCI_H226
LC.HOP_92
LC.HOP_62
LC.EKVX
LC.A549
CO.SW_620
CO.KM12
CO.HT29
CO.HCT_15
CO.HCT_116
CO.HCC_2998
CO.COLO205
CNS.U251
CNS.SNB_75
CNS.SNB_19
CNS.SF_539
CNS.SF_295
CNS.SF_268
BR.T47D
BR.MDA_MB_231
BR.MCF7
BR.HS578T
BR.BT_549
−6
−3
0
3
6
Observed (Z−scores)
Predicted (Z−scores)

NSC 105014, Cladribine (LOCCO)

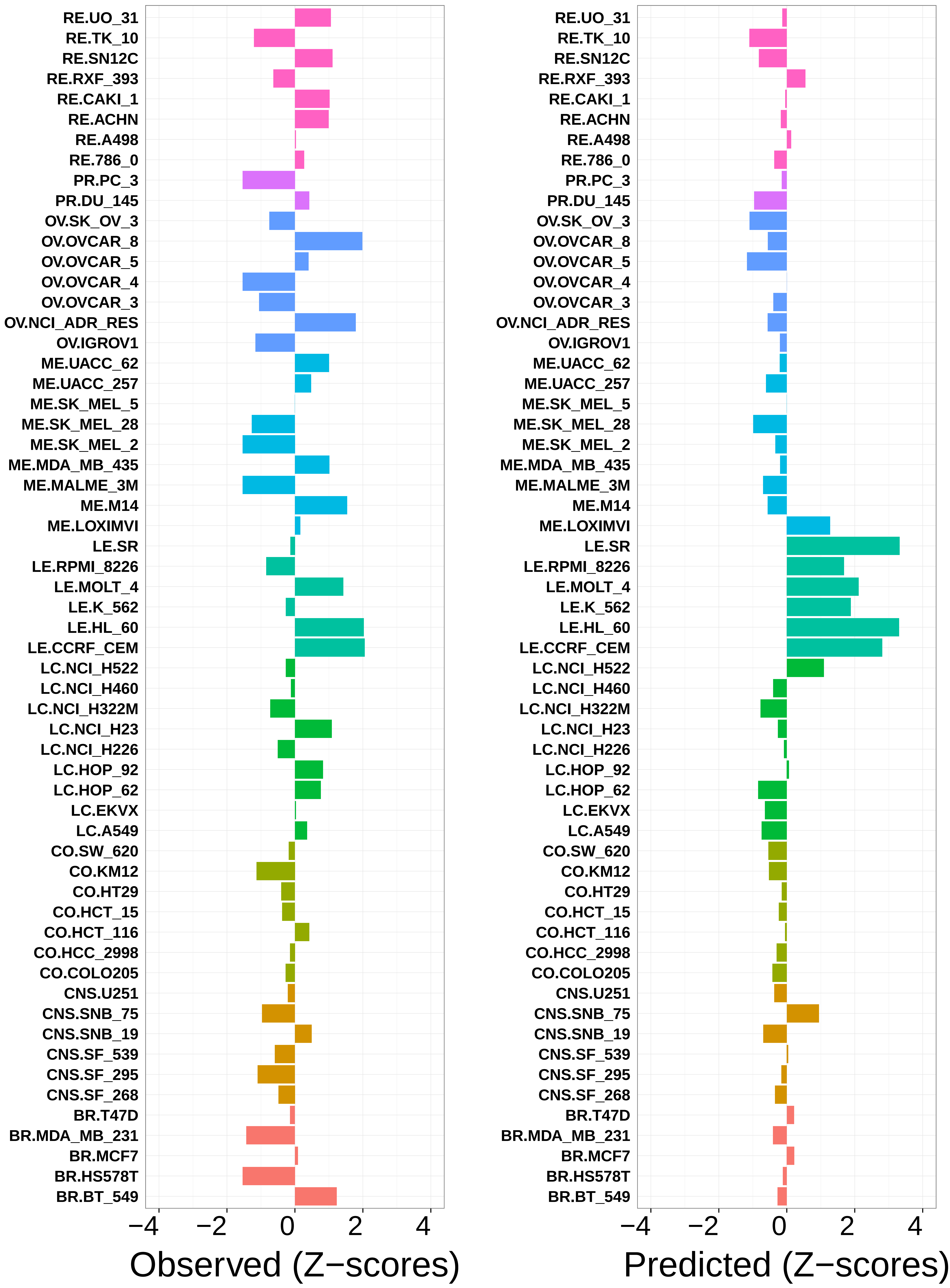

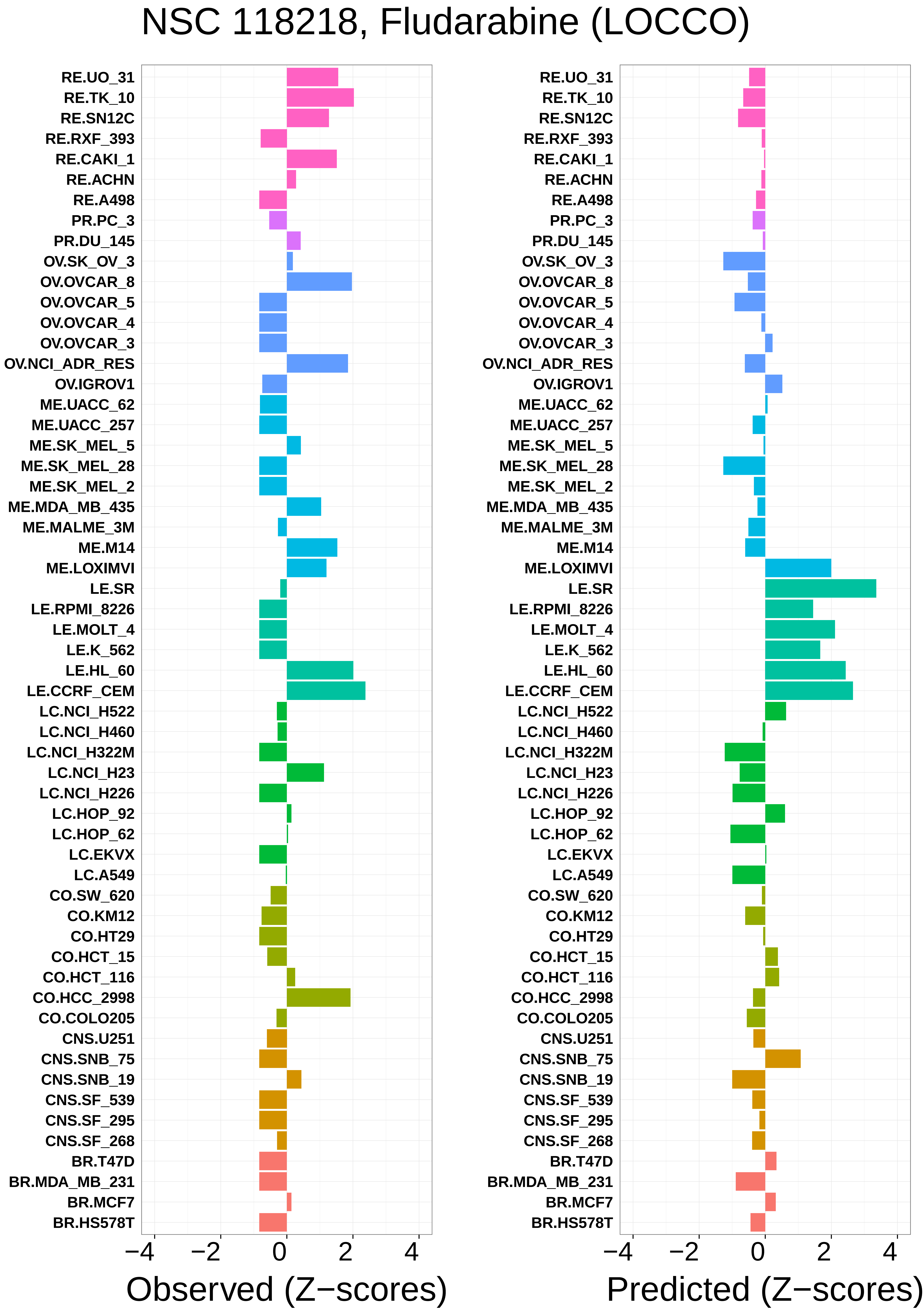
NSC 118218, Fludarabine (LOCCO)
RE.UO_31
RE.TK_10
RE.SN12C
RE.RXF_393
RE.CAKI_1
RE.ACHN
RE.A498
PR.PC_3
PR.DU_145
OV.SK_OV_3
OV.OVCAR_8
OV.OVCAR_5
OV.OVCAR_4
OV.OVCAR_3
OV.NCI_ADR_RES
OV.IGROV1
ME.UACC_62
ME.UACC_257
ME.SK_MEL_5
ME.SK_MEL_28
ME.SK_MEL_2
ME.MDA_MB_435
ME.MALME_3M
ME.M14
ME.LOXIMVI
LE.SR
LE.RPMI_8226
LE.MOLT_4
LE.K_562
LE.HL_60
LE.CCRF_CEM
LC.NCI_H522
LC.NCI_H460
LC.NCI_H322M
LC.NCI_H23
LC.NCI_H226
LC.HOP_92
LC.HOP_62
LC.EKVX
LC.A549
CO.SW_620
CO.KM12
CO.HT29
CO.HCT_15
CO.HCT_116
CO.HCC_2998
CO.COLO205
CNS.U251
CNS.SNB_75
CNS.SNB_19
CNS.SF_539
CNS.SF_295
CNS.SF_268
BR.T47D
BR.MDA_MB_231
BR.MCF7
BR.HS578T
−4
−2
0
2
4
Observed (Z−scores)
Predicted (Z−scores)

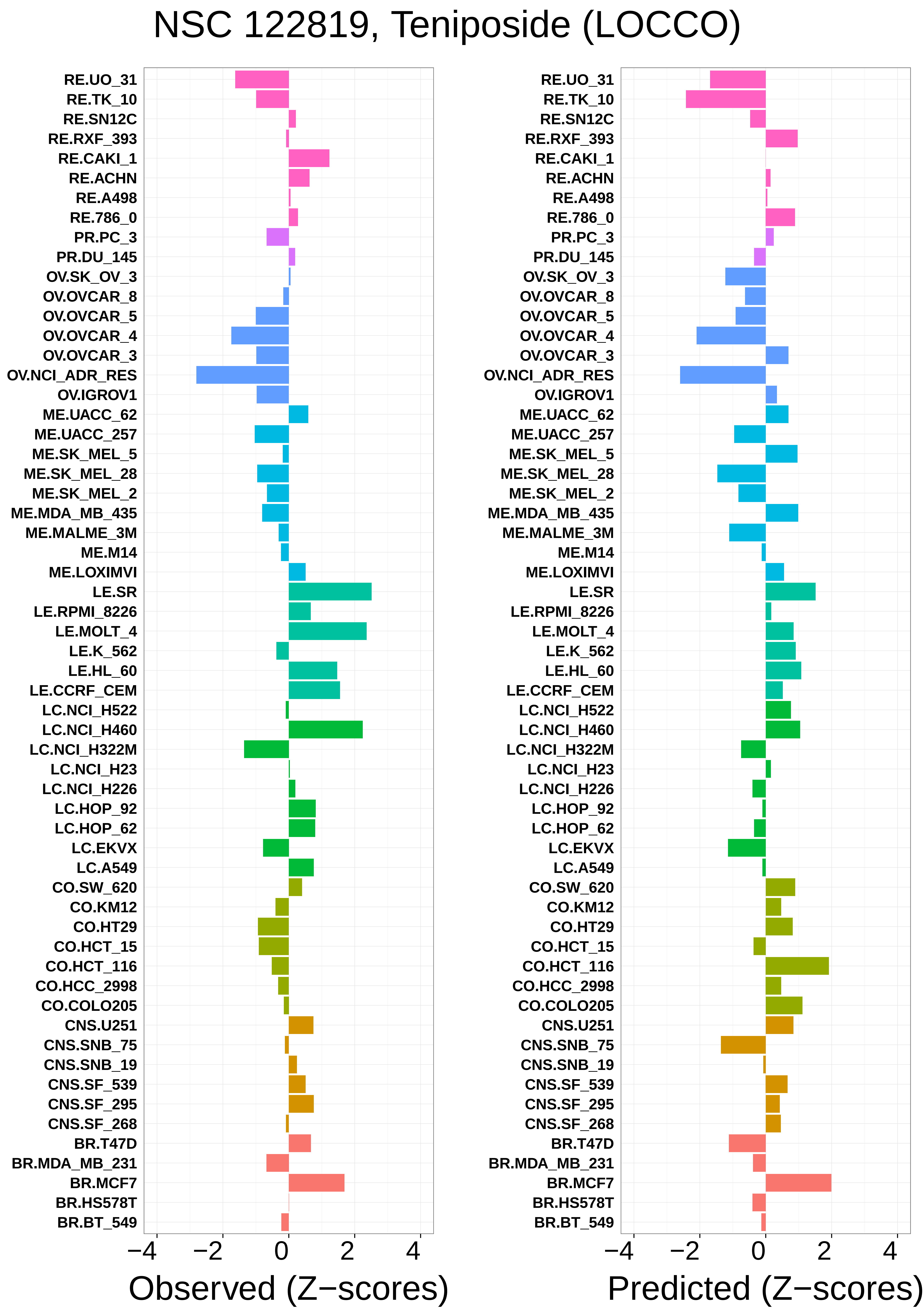

NSC 122819, Teniposide (LOCCO)
RE.UO_31
RE.TK_10
RE.SN12C
RE.RXF_393
RE.CAKI_1
RE.ACHN
RE.A498
RE.786_0
PR.PC_3
PR.DU_145
OV.SK_OV_3
OV.OVCAR_8
OV.OVCAR_5
OV.OVCAR_4
OV.OVCAR_3
OV.NCI_ADR_RES
OV.IGROV1
ME.UACC_62
ME.UACC_257
ME.SK_MEL_5
ME.SK_MEL_28
ME.SK_MEL_2
ME.MDA_MB_435
ME.MALME_3M
ME.M14
ME.LOXIMVI
LE.SR
LE.RPMI_8226
LE.MOLT_4
LE.K_562
LE.HL_60
LE.CCRF_CEM
LC.NCI_H522
LC.NCI_H460
LC.NCI_H322M
LC.NCI_H23
LC.NCI_H226
LC.HOP_92
LC.HOP_62
LC.EKVX
LC.A549
CO.SW_620
CO.KM12
CO.HT29
CO.HCT_15
CO.HCT_116
CO.HCC_2998
CO.COLO205
CNS.U251
CNS.SNB_75
CNS.SNB_19
CNS.SF_539
CNS.SF_295
CNS.SF_268
BR.T47D
BR.MDA_MB_231
BR.MCF7
BR.HS578T
BR.BT_549
−4 −2 0 2 4
Observed (Z−scores)
−4 −2 0 2 4
Predicted (Z−scores)

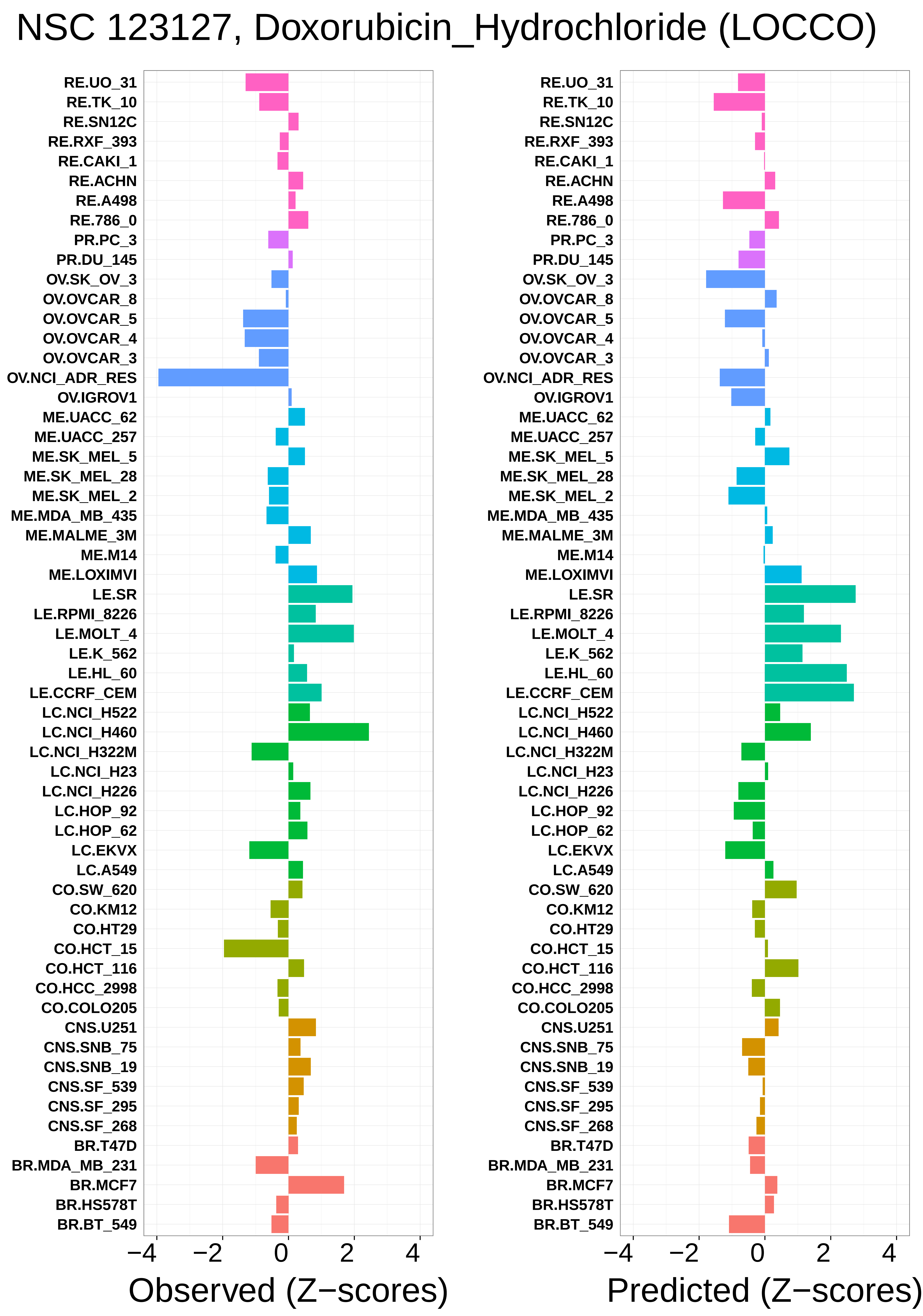
NSC 123127, Doxorubicin_Hydrochloride (LOCCO)
RE.UO_31
RE.TK_10
RE.SN12C
RE.RXF_393
RE.CAKI_1
RE.ACHN
RE.A498
RE.786_0
PR.PC_3
PR.DU_145
OV.SK_OV_3
OV.OVCAR_8
OV.OVCAR_5
OV.OVCAR_4
OV.OVCAR_3
OV.NCI_ADR_RES
OV.IGROV1
ME.UACC_62
ME.UACC_257
ME.SK_MEL_5
ME.SK_MEL_28
ME.SK_MEL_2
ME.MDA_MB_435
ME.MALME_3M
ME.M14
ME.LOXIMVI
LE.SR
LE.RPMI_8226
LE.MOLT_4
LE.K_562
LE.HL_60
LE.CCRF_CEM
LC.NCI_H522
LC.NCI_H460
LC.NCI_H322M
LC.NCI_H23
LC.NCI_H226
LC.HOP_92
LC.HOP_62
LC.EKVX
LC.A549
CO.SW_620
CO.KM12
CO.HT29
CO.HCT_15
CO.HCT_116
CO.HCC_2998
CO.COLO205
CNS.U251
CNS.SNB_75
CNS.SNB_19
CNS.SF_539
CNS.SF_295
CNS.SF_268
BR.T47D
BR.MDA_MB_231
BR.MCF7
BR.HS578T
BR.BT_549
−4
−2
0
2
4
Observed (Z−scores)
Predicted (Z−scores)

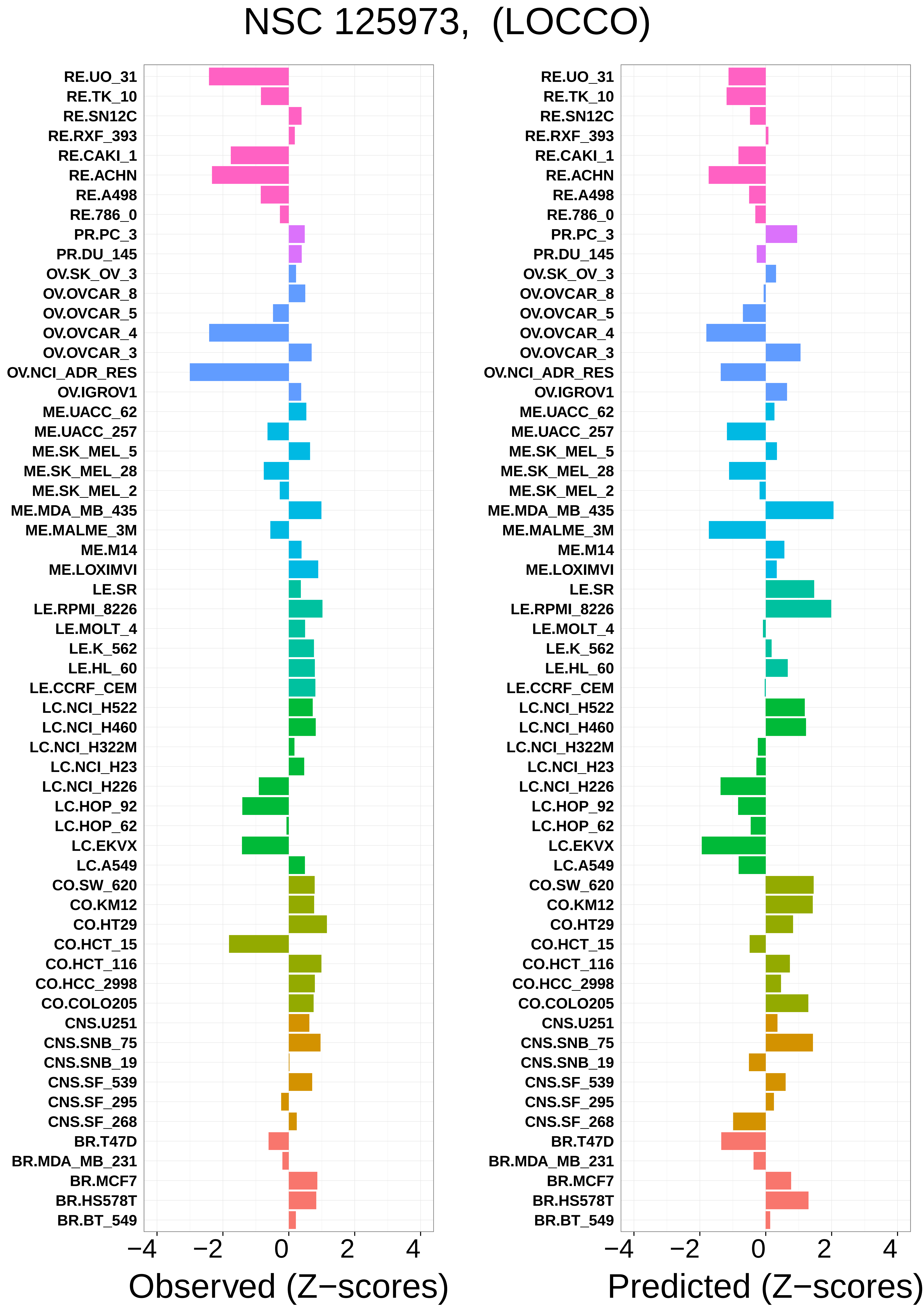
NSC 125973, (LOCCO)
RE.UO_31
RE.TK_10
RE.SN12C
RE.RXF_393
RE.CAKI_1
RE.ACHN
RE.A498
RE.786_0
PR.PC_3
PR.DU_145
OV.SK_OV_3
OV.OVCAR_8
OV.OVCAR_5
OV.OVCAR_4
OV.OVCAR_3
OV.NCI_ADR_RES
OV.IGROV1
ME.UACC_62
ME.UACC_257
ME.SK_MEL_5
ME.SK_MEL_28
ME.SK_MEL_2
ME.MDA_MB_435
ME.MALME_3M
ME.M14
ME.LOXIMVI
LE.SR
LE.RPMI_8226
LE.MOLT_4
LE.K_562
LE.HL_60
LE.CCRF_CEM
LC.NCI_H522
LC.NCI_H460
LC.NCI_H322M
LC.NCI_H23
LC.NCI_H226
LC.HOP_92
LC.HOP_62
LC.EKVX
LC.A549
CO.SW_620
CO.KM12
CO.HT29
CO.HCT_15
CO.HCT_116
CO.HCC_2998
CO.COLO205
CNS.U251
CNS.SNB_75
CNS.SNB_19
CNS.SF_539
CNS.SF_295
CNS.SF_268
BR.T47D
BR.MDA_MB_231
BR.MCF7
BR.HS578T
BR.BT_549
−4
−2
0
2
4
Observed (Z−scores)
Predicted (Z−scores)

NSC 127716, Decitabine (LOCCO)

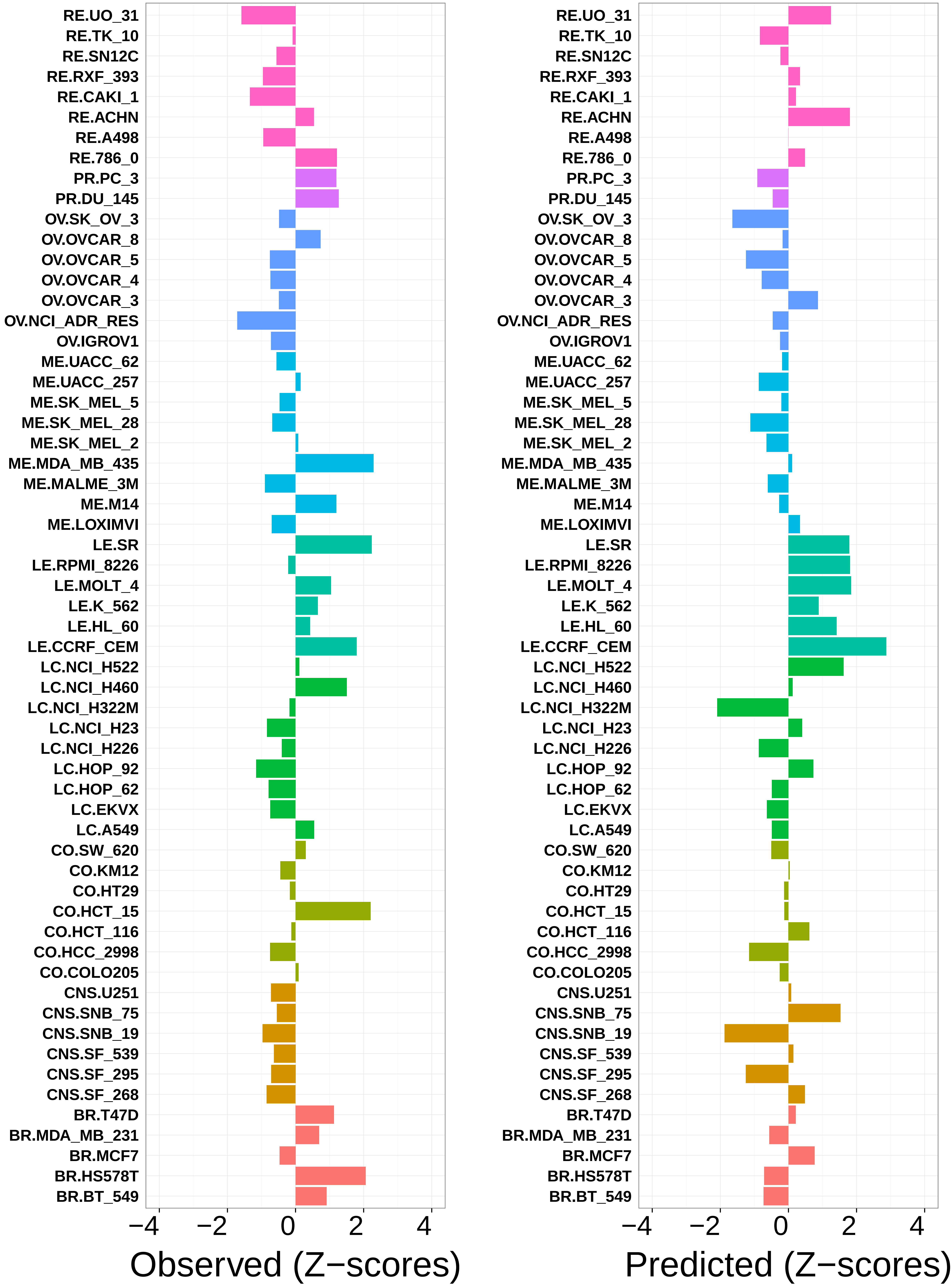

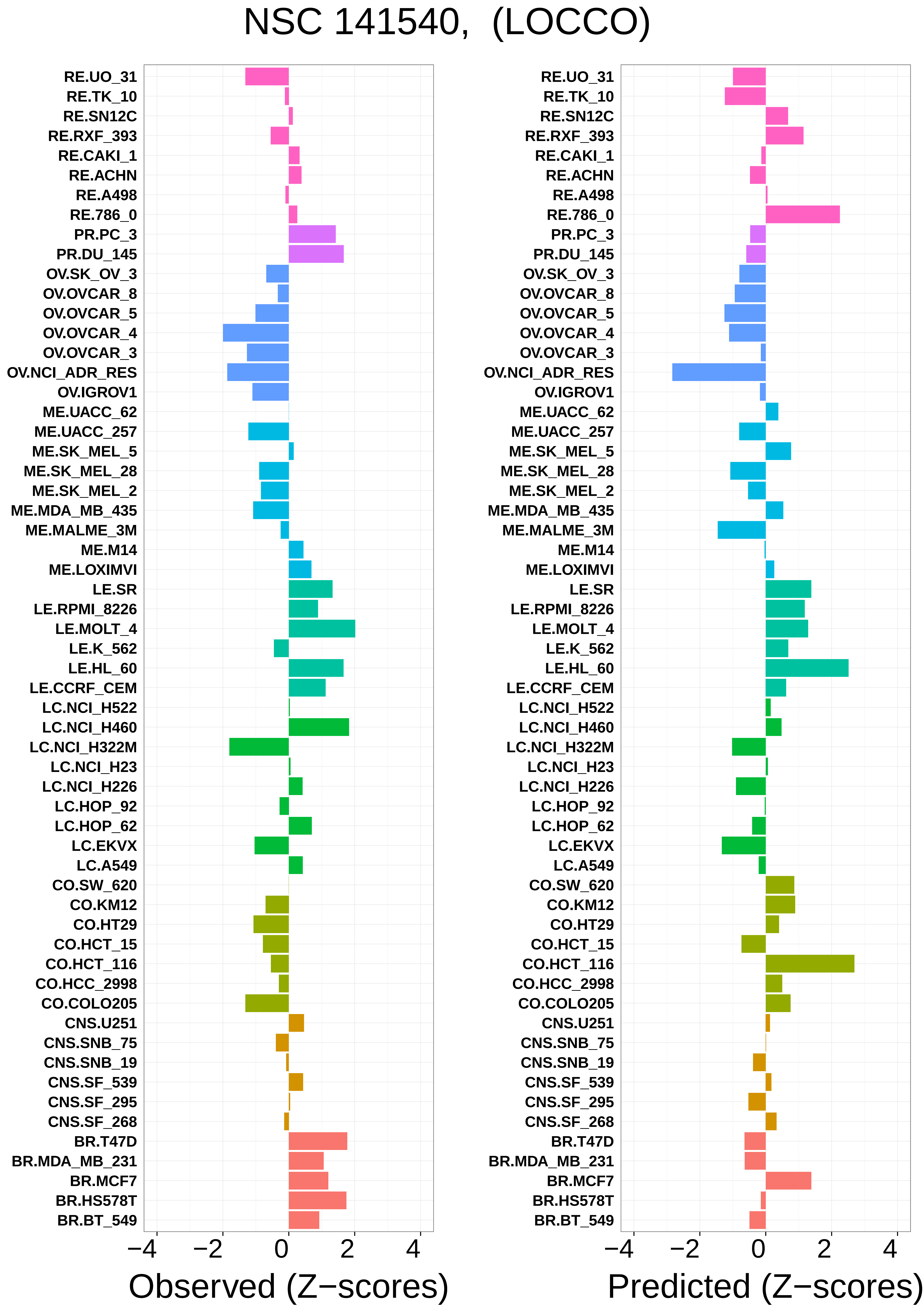
NSC 141540, (LOCCO)
RE.UO_31
RE.TK_10
RE.SN12C
RE.RXF_393
RE.CAKI_1
RE.ACHN
RE.A498
RE.786_0
PR.PC_3
PR.DU_145
OV.SK_OV_3
OV.OVCAR_8
OV.OVCAR_5
OV.OVCAR_4
OV.OVCAR_3
OV.NCI_ADR_RES
OV.IGROV1
ME.UACC_62
ME.UACC_257
ME.SK_MEL_5
ME.SK_MEL_28
ME.SK_MEL_2
ME.MDA_MB_435
ME.MALME_3M
ME.M14
ME.LOXIMVI
LE.SR
LE.RPMI_8226
LE.MOLT_4
LE.K_562
LE.HL_60
LE.CCRF_CEM
LC.NCI_H522
LC.NCI_H460
LC.NCI_H322M
LC.NCI_H23
LC.NCI_H226
LC.HOP_92
LC.HOP_62
LC.EKVX
LC.A549
CO.SW_620
CO.KM12
CO.HT29
CO.HCT_15
CO.HCT_116
CO.HCC_2998
CO.COLO205
CNS.U251
CNS.SNB_75
CNS.SNB_19
CNS.SF_539
CNS.SF_295
CNS.SF_268
BR.T47D
BR.MDA_MB_231
BR.MCF7
BR.HS578T
BR.BT_549
−4 −2 0 2 4
Observed (Z−scores)
−4 −2 0 2 4
Predicted (Z−scores)

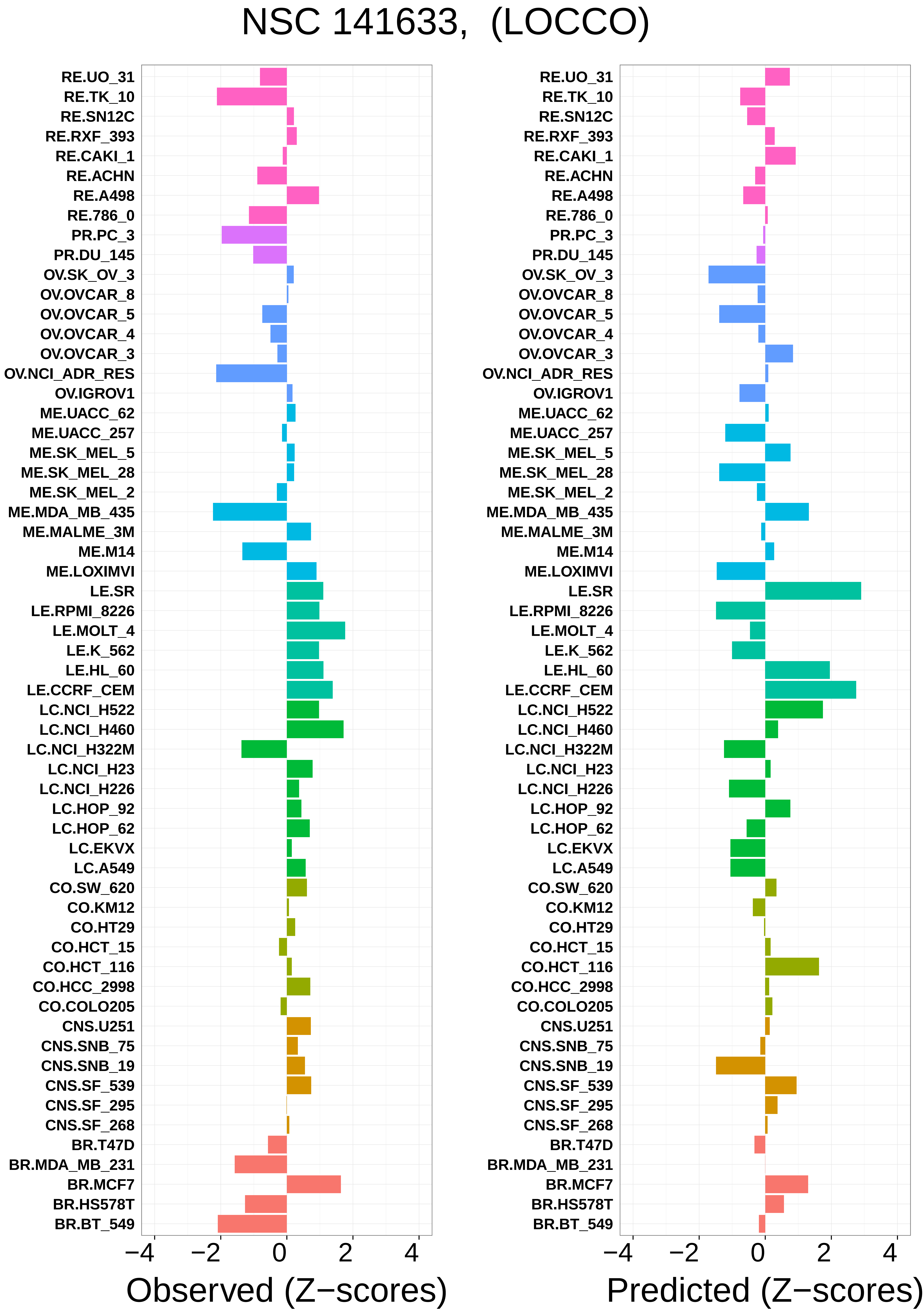

NSC 141633, (LOCCO)
RE.UO_31
RE.TK_10
RE.SN12C
RE.RXF_393
RE.CAKI_1
RE.ACHN
RE.A498
RE.786_0
PR.PC_3
PR.DU_145
OV.SK_OV_3
OV.OVCAR_8
OV.OVCAR_5
OV.OVCAR_4
OV.OVCAR_3
OV.NCI_ADR_RES
OV.IGROV1
ME.UACC_62
ME.UACC_257
ME.SK_MEL_5
ME.SK_MEL_28
ME.SK_MEL_2
ME.MDA_MB_435
ME.MALME_3M
ME.M14
ME.LOXIMVI
LE.SR
LE.RPMI_8226
LE.MOLT_4
LE.K_562
LE.HL_60
LE.CCRF_CEM
LC.NCI_H522
LC.NCI_H460
LC.NCI_H322M
LC.NCI_H23
LC.NCI_H226
LC.HOP_92
LC.HOP_62
LC.EKVX
LC.A549
CO.SW_620
CO.KM12
CO.HT29
CO.HCT_15
CO.HCT_116
CO.HCC_2998
CO.COLO205
CNS.U251
CNS.SNB_75
CNS.SNB_19
CNS.SF_539
CNS.SF_295
CNS.SF_268
BR.T47D
BR.MDA_MB_231
BR.MCF7
BR.HS578T
BR.BT_549
−4 −2 0 2 4
Observed (Z−scores)
Predicted (Z−scores)

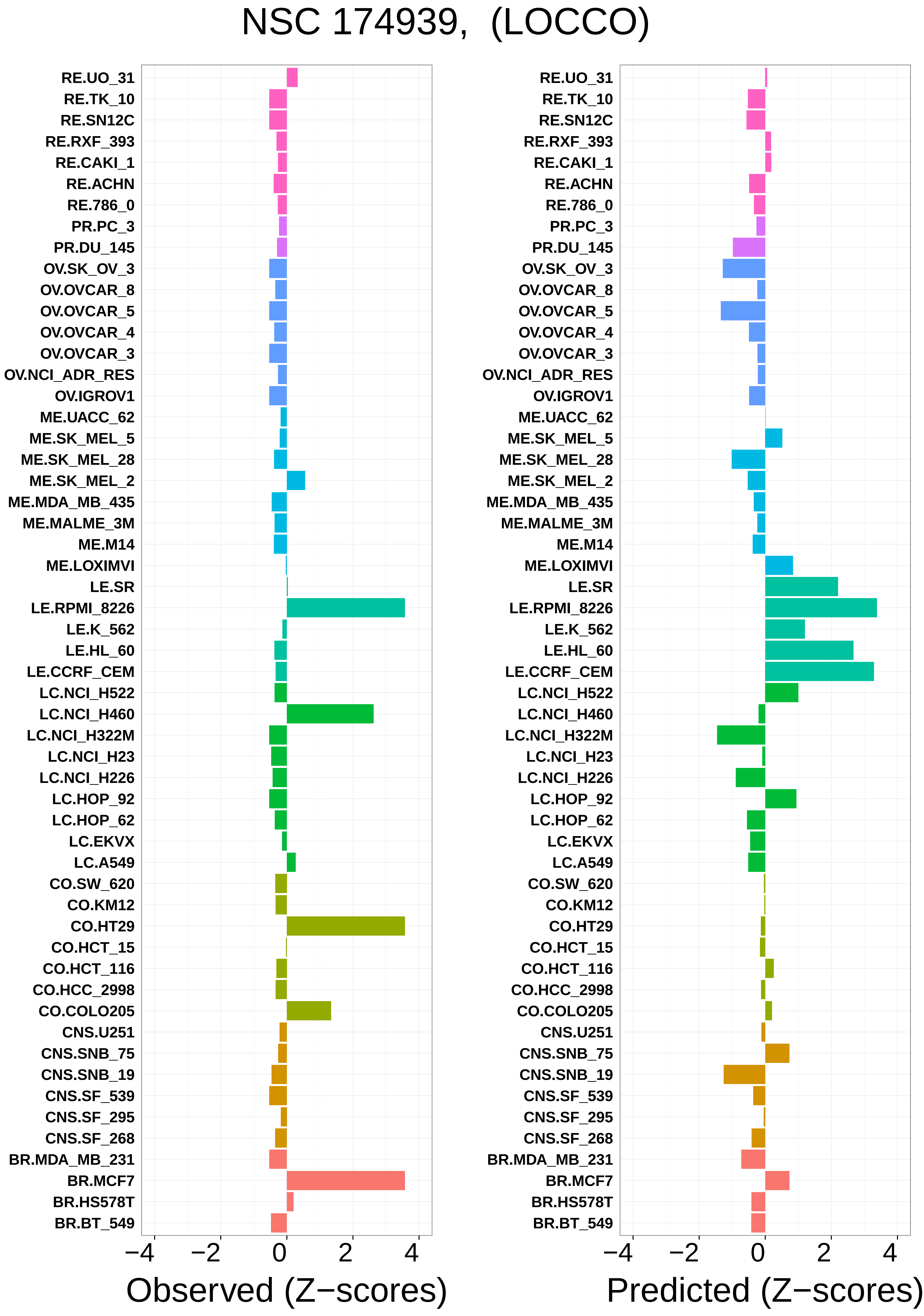
NSC 174939, (LOCCO)
RE.UO_31
RE.TK_10
RE.SN12C
RE.RXF_393
RE.CAKI_1
RE.ACHN
RE.786_0
PR.PC_3
PR.DU_145
OV.SK_OV_3
OV.OVCAR_8
OV.OVCAR_5
OV.OVCAR_4
OV.OVCAR_3
OV.NCI_ADR_RES
OV.IGROV1
ME.UACC_62
ME.SK_MEL_5
ME.SK_MEL_28
ME.SK_MEL_2
ME.MDA_MB_435
ME.MALME_3M
ME.M14
ME.LOXIMVI
LE.SR
LE.RPMI_8226
LE.K_562
LE.HL_60
LE.CCRF_CEM
LC.NCI_H522
LC.NCI_H460
LC.NCI_H322M
LC.NCI_H23
LC.NCI_H226
LC.HOP_92
LC.HOP_62
LC.EKVX
LC.A549
CO.SW_620
CO.KM12
CO.HT29
CO.HCT_15
CO.HCT_116
CO.HCC_2998
CO.COLO205
CNS.U251
CNS.SNB_75
CNS.SNB_19
CNS.SF_539
CNS.SF_295
CNS.SF_268
BR.MDA_MB_231
BR.MCF7
BR.HS578T
BR.BT_549
−4 −2 0 2 4
Observed (Z−scores)
−4 −2 0 2 4
Predicted (Z−scores)

NSC 19893, Fluorouracil (LOCCO)

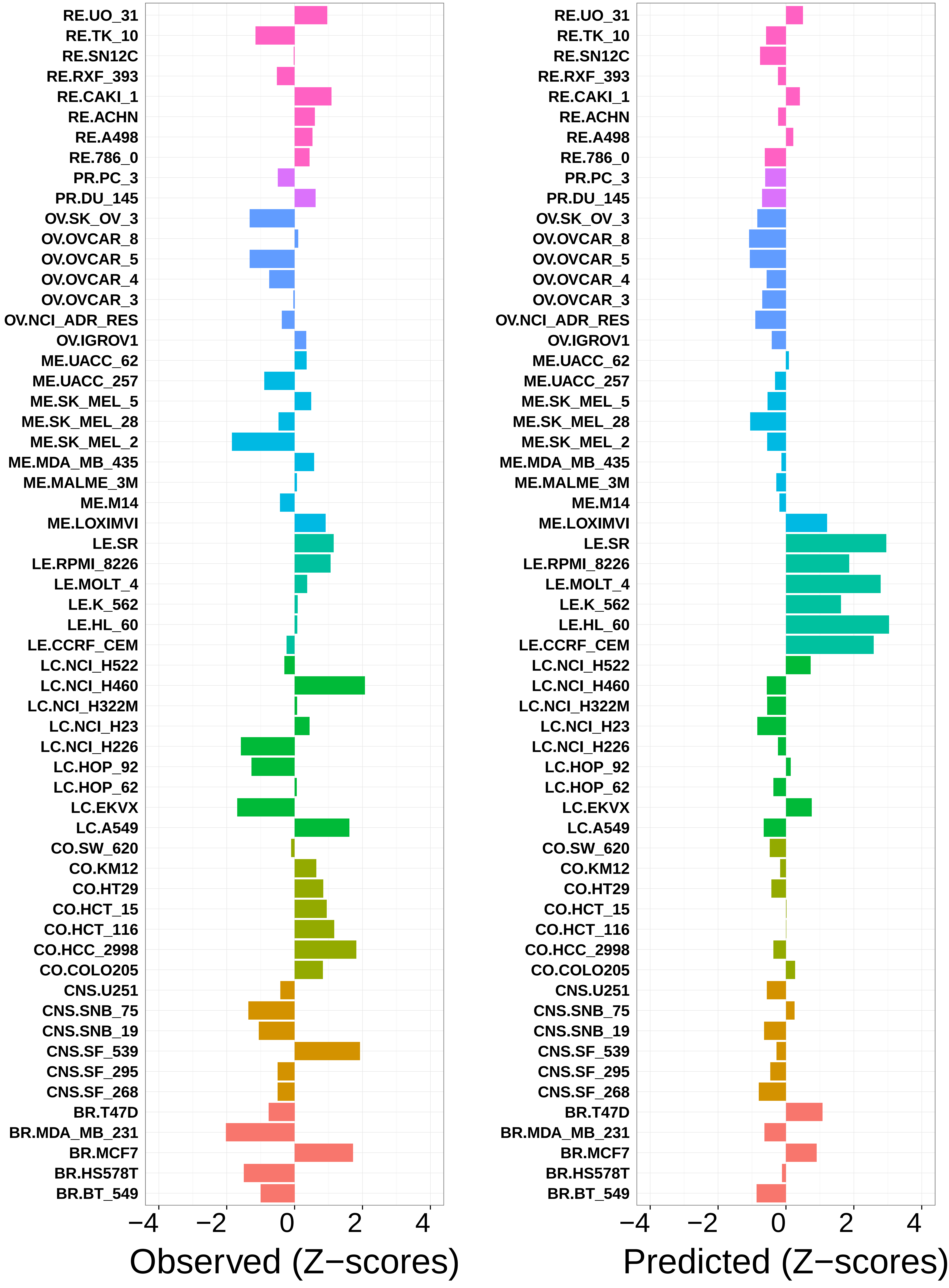

NSC 221019, (LOCCO)

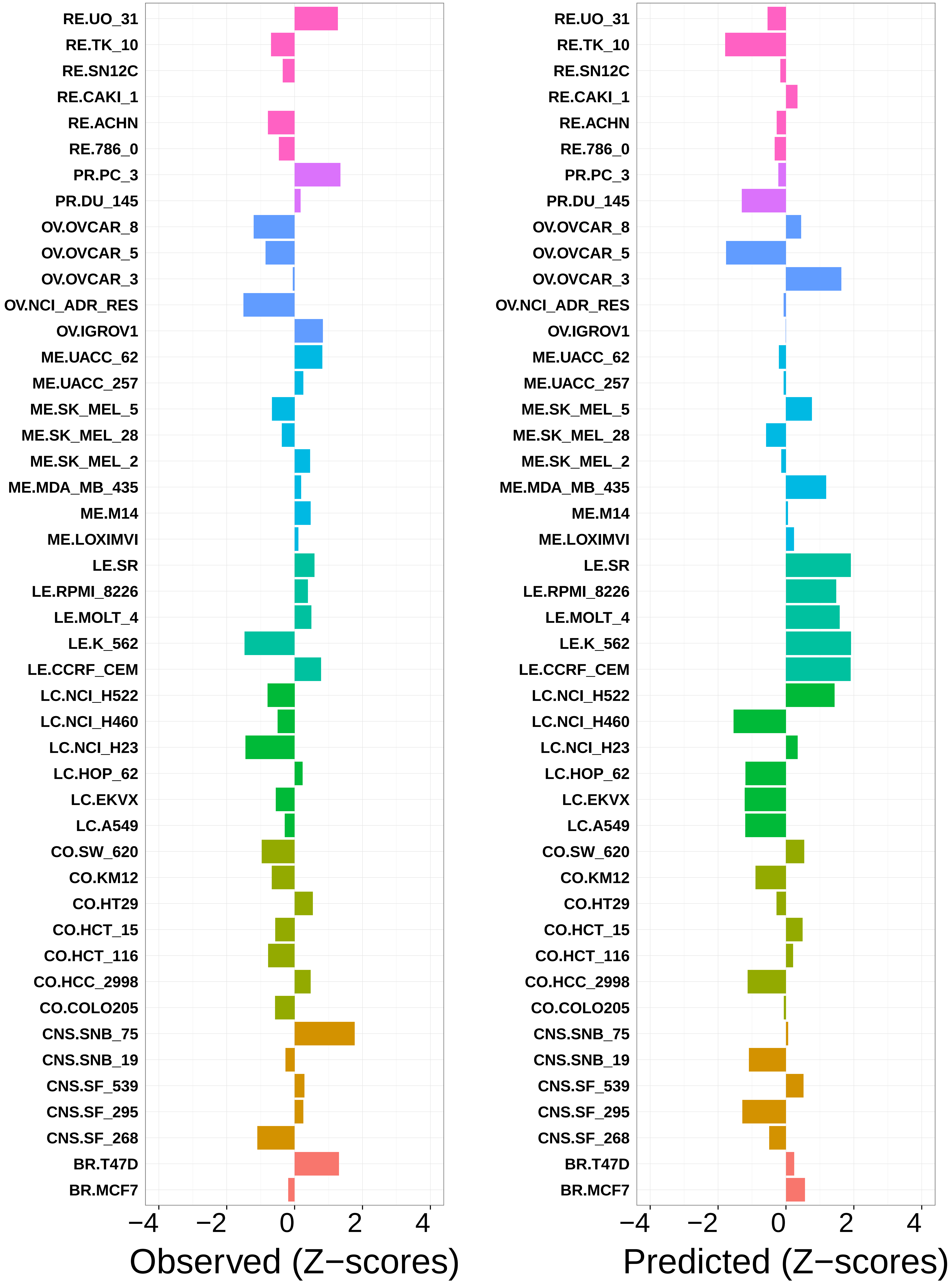

NSC 246131, (LOCCO)

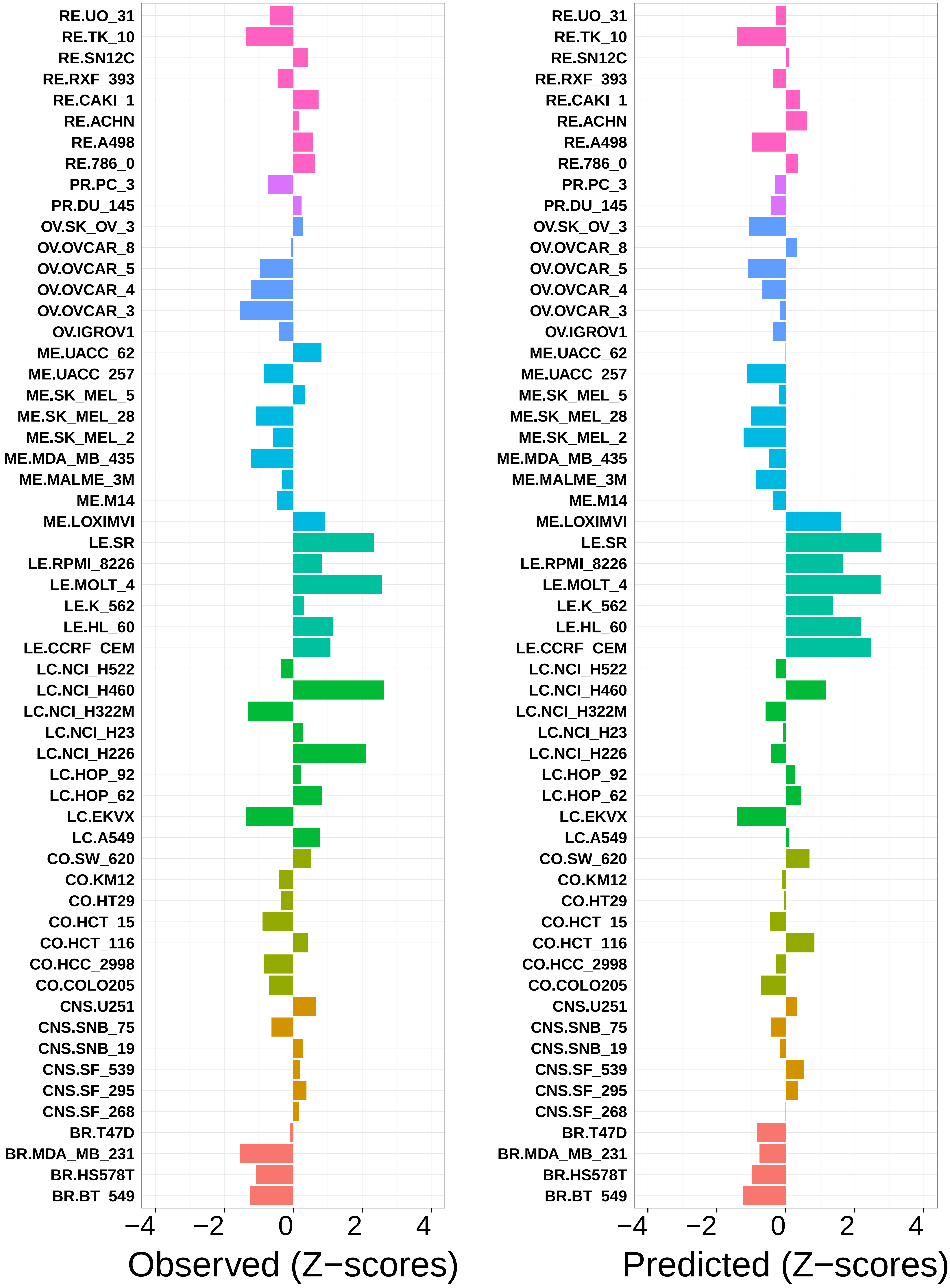

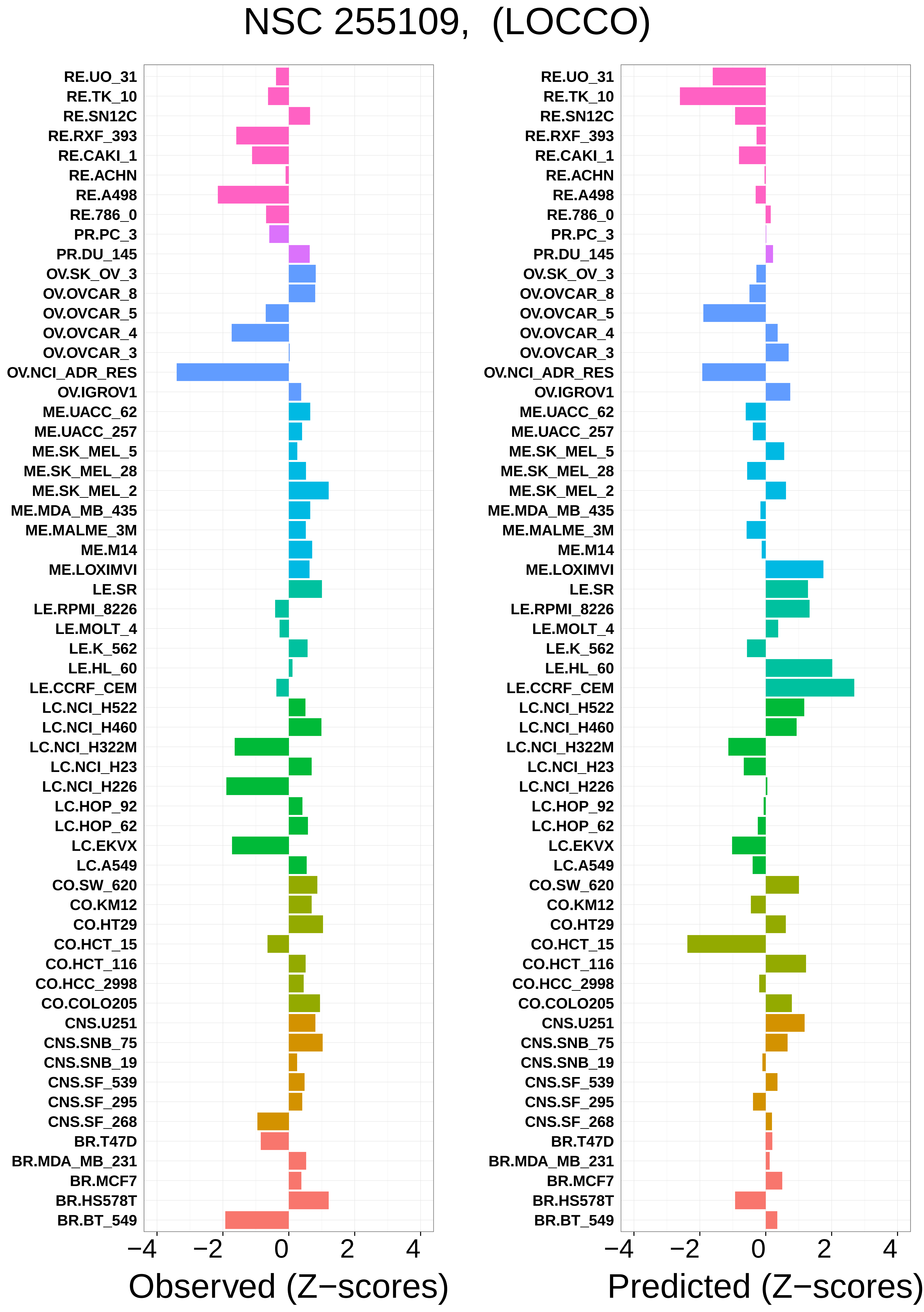
NSC 255109, (LOCCO)
RE.UO_31
RE.TK_10
RE.SN12C
RE.RXF_393
RE.CAKI_1
RE.ACHN
RE.A498
RE.786_0
PR.PC_3
PR.DU_145
OV.SK_OV_3
OV.OVCAR_8
OV.OVCAR_5
OV.OVCAR_4
OV.OVCAR_3
OV.NCI_ADR_RES
OV.IGROV1
ME.UACC_62
ME.UACC_257
ME.SK_MEL_5
ME.SK_MEL_28
ME.SK_MEL_2
ME.MDA_MB_435
ME.MALME_3M
ME.M14
ME.LOXIMVI
LE.SR
LE.RPMI_8226
LE.MOLT_4
LE.K_562
LE.HL_60
LE.CCRF_CEM
LC.NCI_H522
LC.NCI_H460
LC.NCI_H322M
LC.NCI_H23
LC.NCI_H226
LC.HOP_92
LC.HOP_62
LC.EKVX
LC.A549
CO.SW_620
CO.KM12
CO.HT29
CO.HCT_15
CO.HCT_116
CO.HCC_2998
CO.COLO205
CNS.U251
CNS.SNB_75
CNS.SNB_19
CNS.SF_539
CNS.SF_295
CNS.SF_268
BR.T47D
BR.MDA_MB_231
BR.MCF7
BR.HS578T
BR.BT_549
−4
−2
0
2
4
Observed (Z−scores)
Predicted (Z−scores)

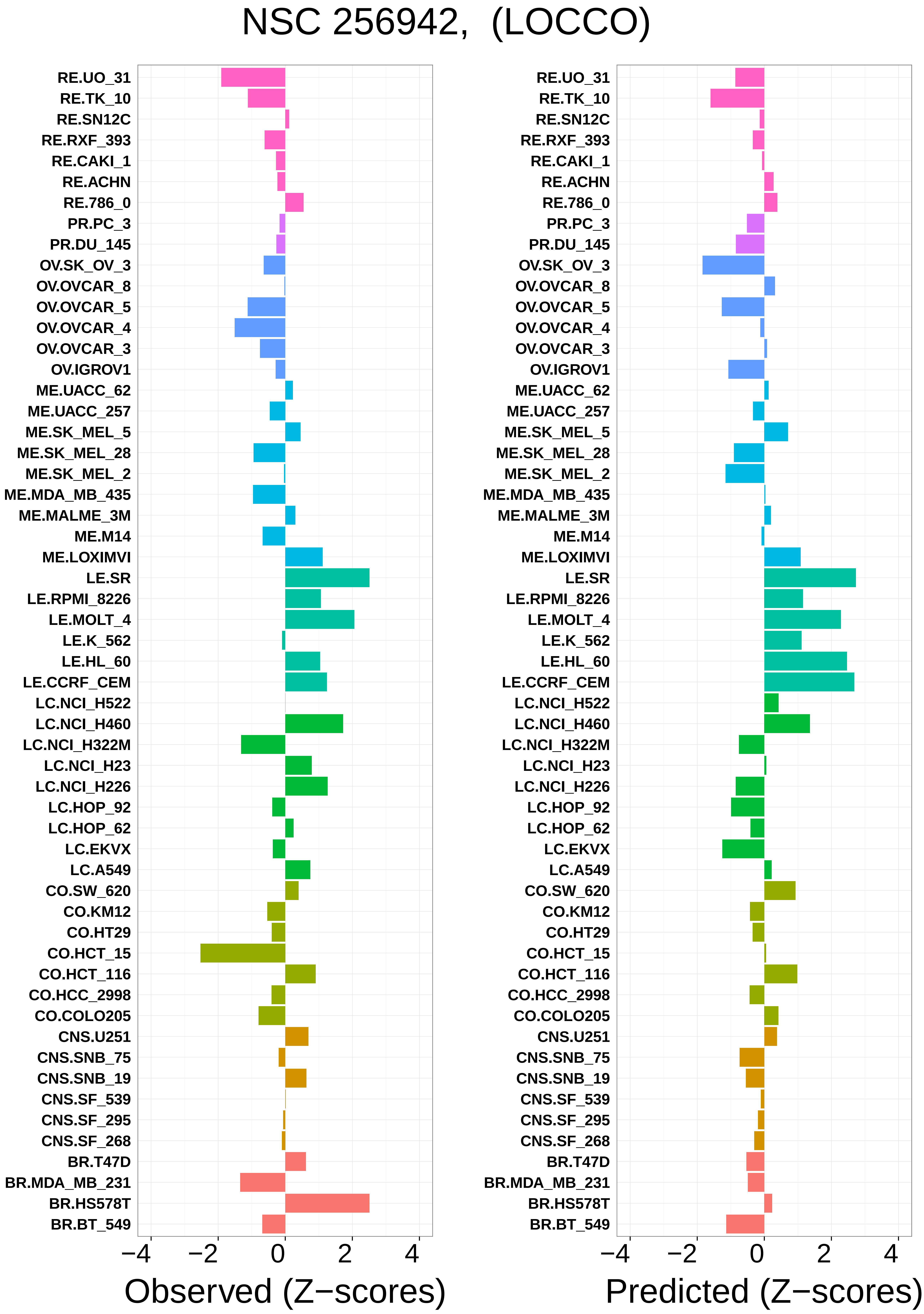
NSC 256942, (LOCCO)
RE.UO_31
RE.TK_10
RE.SN12C
RE.RXF_393
RE.CAKI_1
RE.ACHN
RE.786_0
PR.PC_3
PR.DU_145
OV.SK_OV_3
OV.OVCAR_8
OV.OVCAR_5
OV.OVCAR_4
OV.OVCAR_3
OV.IGROV1
ME.UACC_62
ME.UACC_257
ME.SK_MEL_5
ME.SK_MEL_28
ME.SK_MEL_2
ME.MDA_MB_435
ME.MALME_3M
ME.M14
ME.LOXIMVI
LE.SR
LE.RPMI_8226
LE.MOLT_4
LE.K_562
LE.HL_60
LE.CCRF_CEM
LC.NCI_H522
LC.NCI_H460
LC.NCI_H322M
LC.NCI_H23
LC.NCI_H226
LC.HOP_92
LC.HOP_62
LC.EKVX
LC.A549
CO.SW_620
CO.KM12
CO.HT29
CO.HCT_15
CO.HCT_116
CO.HCC_2998
CO.COLO205
CNS.U251
CNS.SNB_75
CNS.SNB_19
CNS.SF_539
CNS.SF_295
CNS.SF_268
BR.T47D
BR.MDA_MB_231
BR.HS578T
BR.BT_549
−4 −2 0 2 4
Observed (Z−scores)
Predicted (Z−scores)

NSC 26980, Mitomycin (LOCCO)

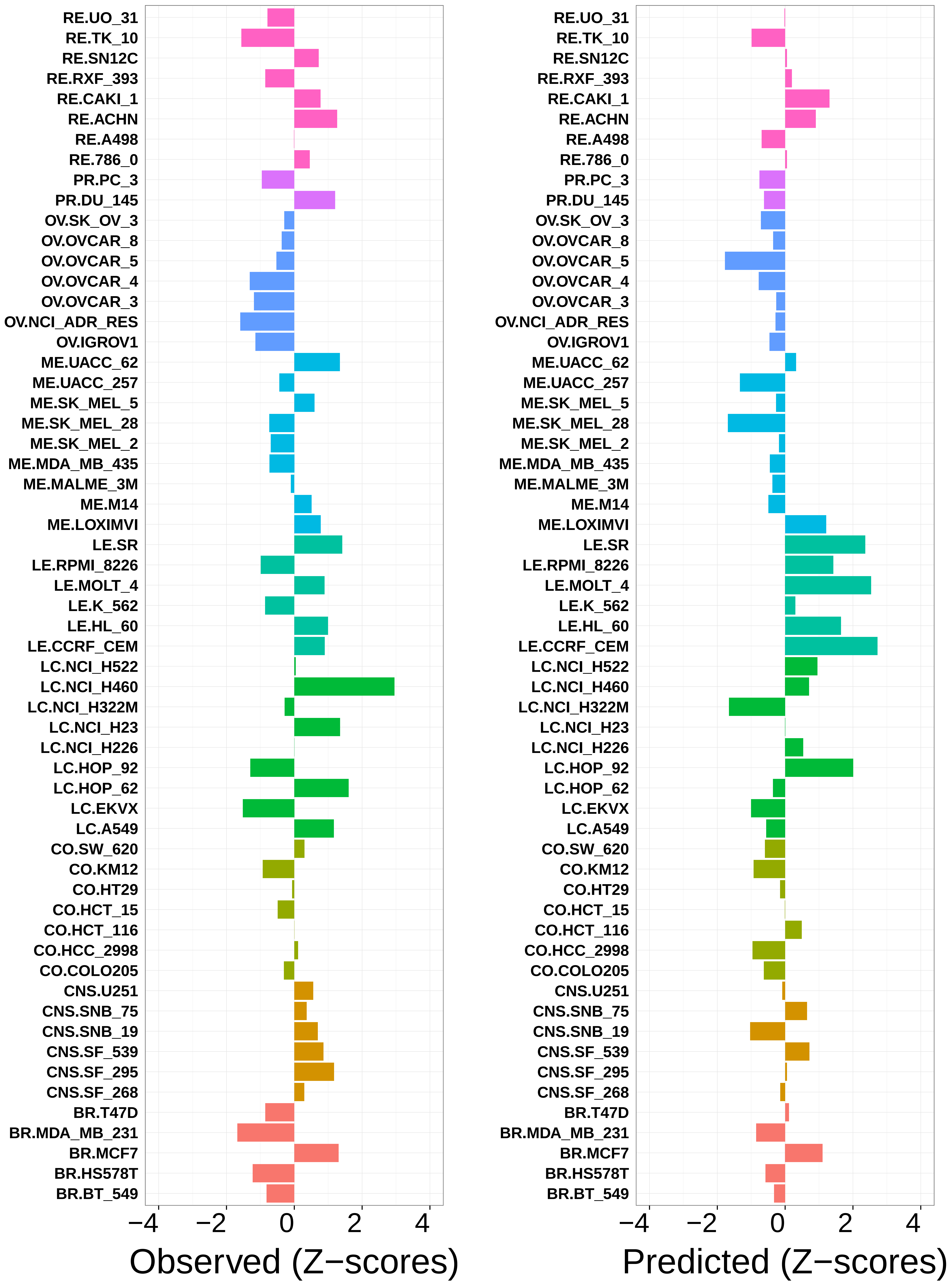

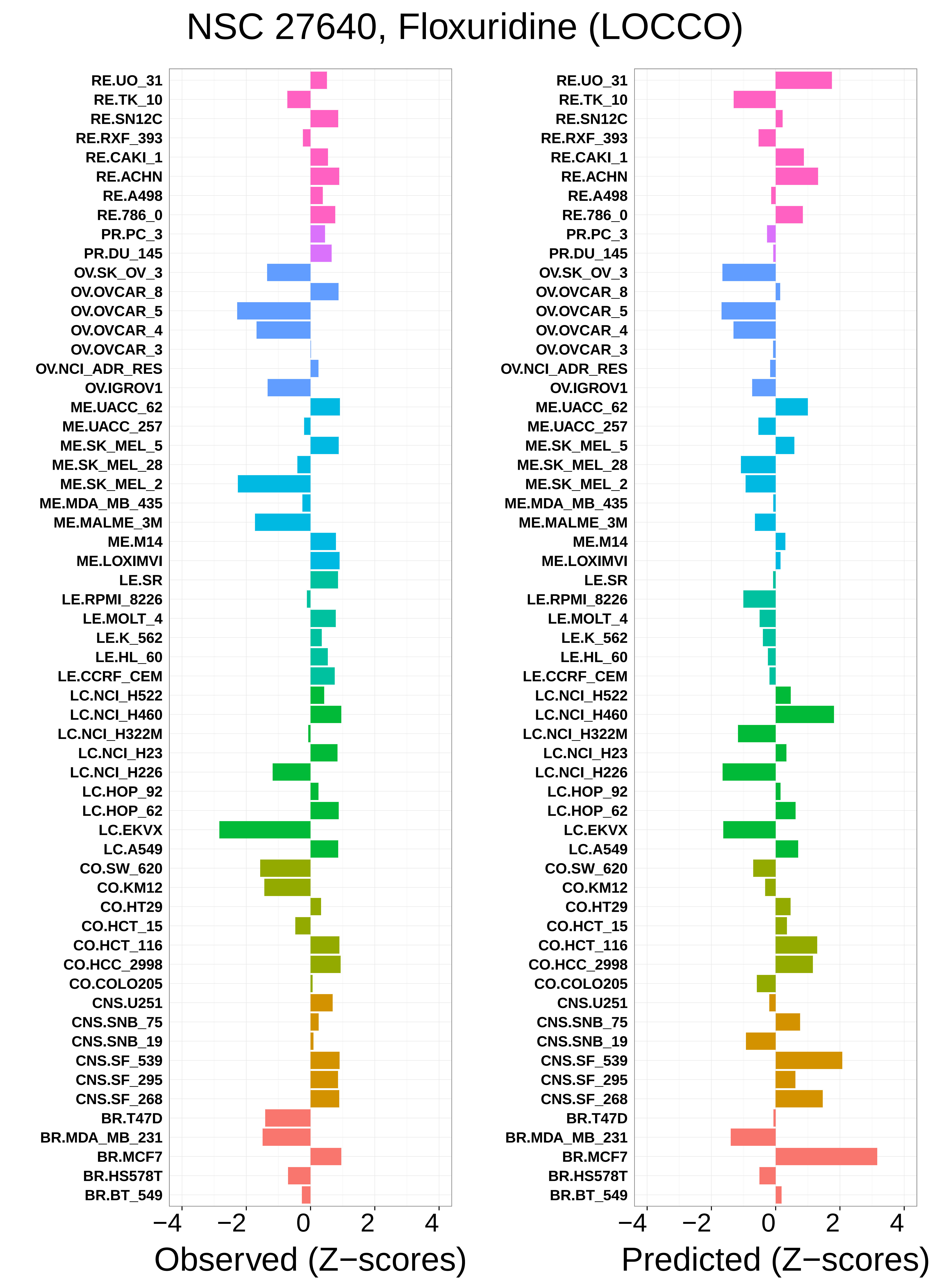

NSC 27640, Floxuridine (LOCCO)
RE.UO_31
RE.TK_10
RE.SN12C
RE.RXF_393
RE.CAKI_1
RE.ACHN
RE.A498
RE.786_0
PR.PC_3
PR.DU_145
OV.SK_OV_3
OV.OVCAR_8
OV.OVCAR_5
OV.OVCAR_4
OV.OVCAR_3
OV.NCI_ADR_RES
OV.IGROV1
ME.UACC_62
ME.UACC_257
ME.SK_MEL_5
ME.SK_MEL_28
ME.SK_MEL_2
ME.MDA_MB_435
ME.MALME_3M
ME.M14
ME.LOXIMVI
LE.SR
LE.RPMI_8226
LE.MOLT_4
LE.K_562
LE.HL_60
LE.CCRF_CEM
LC.NCI_H522
LC.NCI_H460
LC.NCI_H322M
LC.NCI_H23
LC.NCI_H226
LC.HOP_92
LC.HOP_62
LC.EKVX
LC.A549
CO.SW_620
CO.KM12
CO.HT29
CO.HCT_15
CO.HCT_116
CO.HCC_2998
CO.COLO205
CNS.U251
CNS.SNB_75
CNS.SNB_19
CNS.SF_539
CNS.SF_295
CNS.SF_268
BR.T47D
BR.MDA_MB_231
BR.MCF7
BR.HS578T
BR.BT_549
−4 −2 0 2 4
Observed (Z−scores)
Predicted (Z−scores)

NSC 279836, (LOCCO)

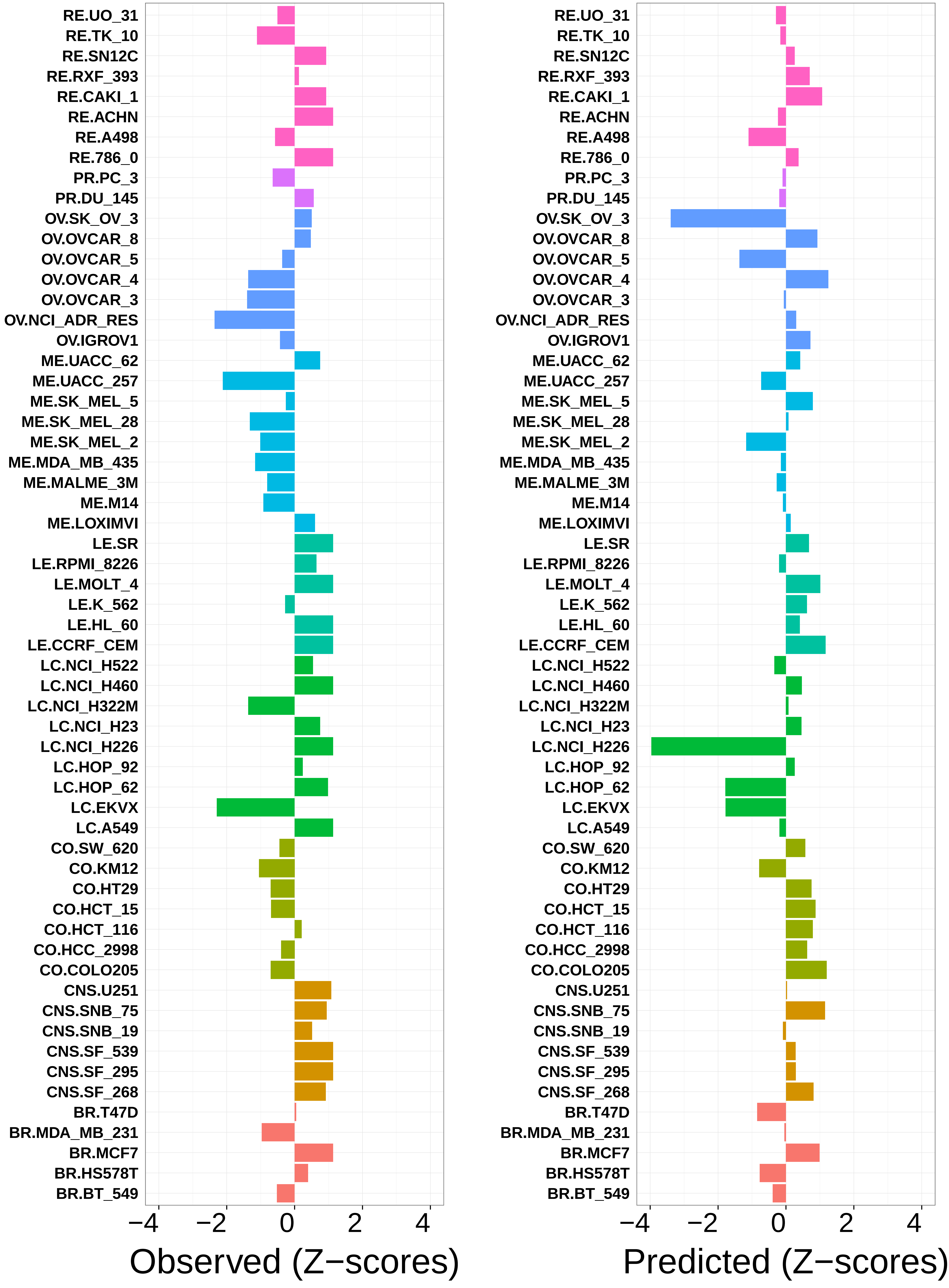

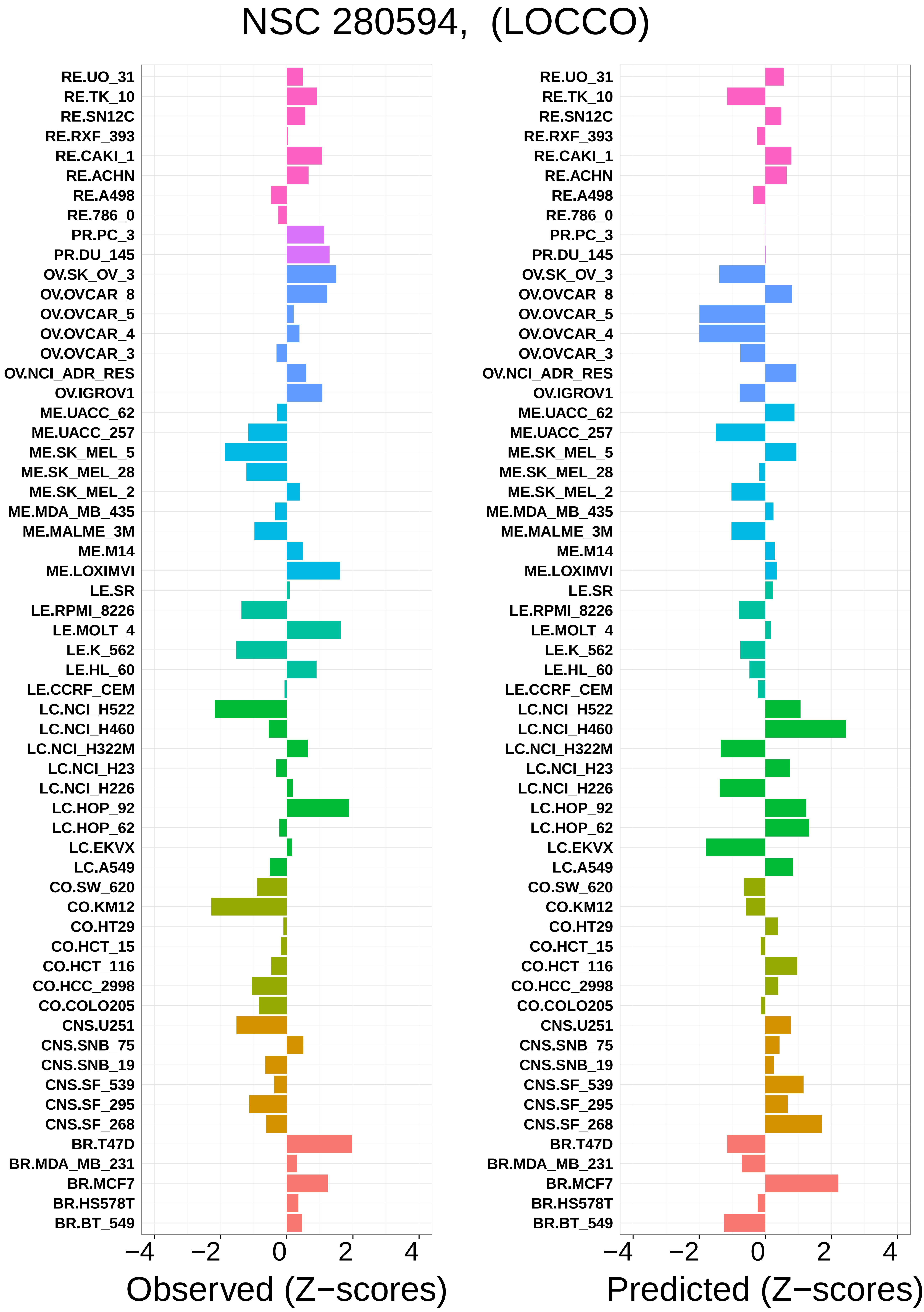
NSC 280594, (LOCCO)
RE.UO_31
RE.TK_10
RE.SN12C
RE.RXF_393
RE.CAKI_1
RE.ACHN
RE.A498
RE.786_0
PR.PC_3
PR.DU_145
OV.SK_OV_3
OV.OVCAR_8
OV.OVCAR_5
OV.OVCAR_4
OV.OVCAR_3
OV.NCI_ADR_RES
OV.IGROV1
ME.UACC_62
ME.UACC_257
ME.SK_MEL_5
ME.SK_MEL_28
ME.SK_MEL_2
ME.MDA_MB_435
ME.MALME_3M
ME.M14
ME.LOXIMVI
LE.SR
LE.RPMI_8226
LE.MOLT_4
LE.K_562
LE.HL_60
LE.CCRF_CEM
LC.NCI_H522
LC.NCI_H460
LC.NCI_H322M
LC.NCI_H23
LC.NCI_H226
LC.HOP_92
LC.HOP_62
LC.EKVX
LC.A549
CO.SW_620
CO.KM12
CO.HT29
CO.HCT_15
CO.HCT_116
CO.HCC_2998
CO.COLO205
CNS.U251
CNS.SNB_75
CNS.SNB_19
CNS.SF_539
CNS.SF_295
CNS.SF_268
BR.T47D
BR.MDA_MB_231
BR.MCF7
BR.HS578T
BR.BT_549
−4
−2
0
2
4
Observed (Z−scores)
Predicted (Z−scores)

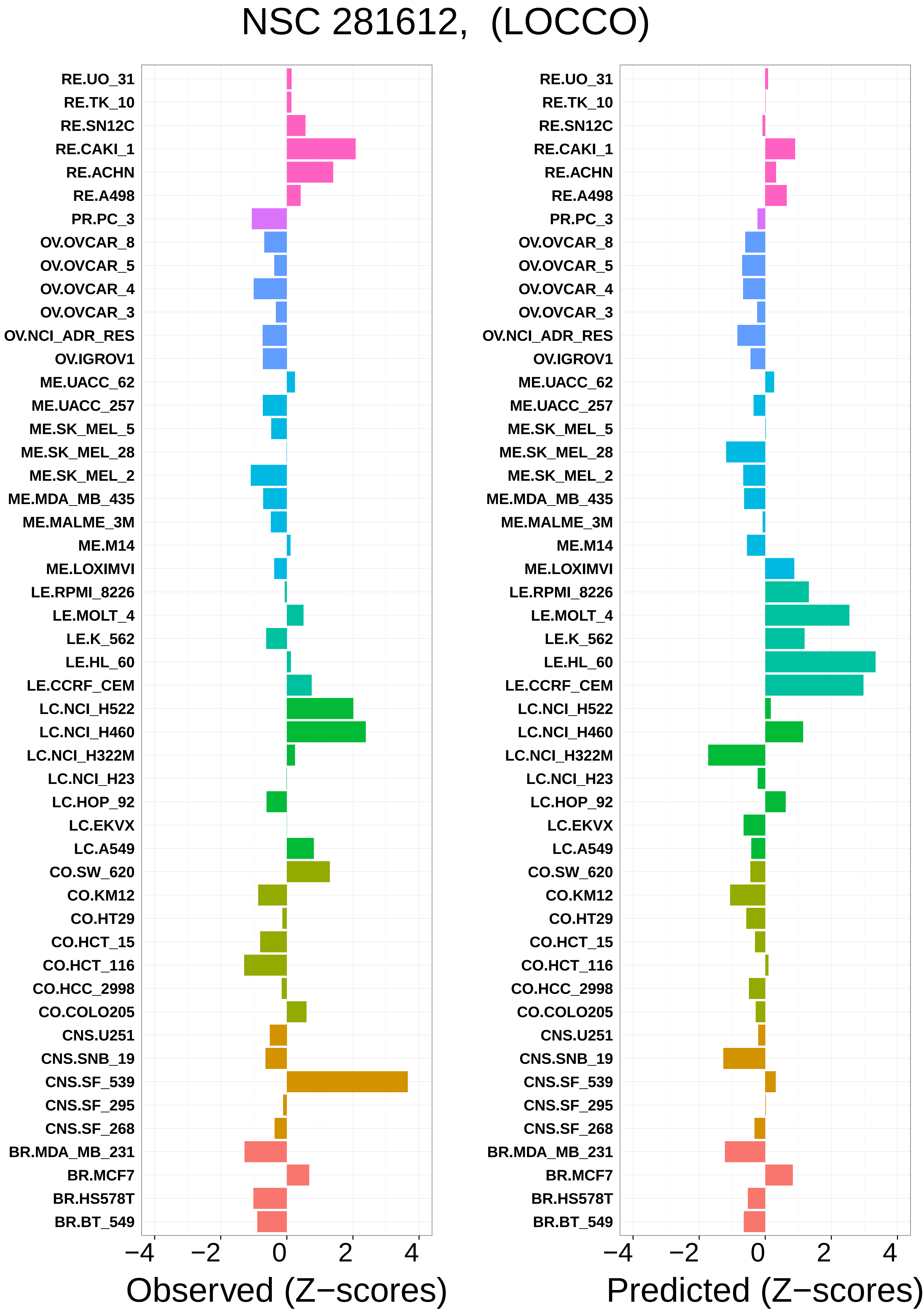
NSC 281612, (LOCCO)
RE.UO_31
RE.TK_10
RE.SN12C
RE.CAKI_1
RE.ACHN
RE.A498
PR.PC_3
OV.OVCAR_8
OV.OVCAR_5
OV.OVCAR_4
OV.OVCAR_3
OV.NCI_ADR_RES
OV.IGROV1
ME.UACC_62
ME.UACC_257
ME.SK_MEL_5
ME.SK_MEL_28
ME.SK_MEL_2
ME.MDA_MB_435
ME.MALME_3M
ME.M14
ME.LOXIMVI
LE.RPMI_8226
LE.MOLT_4
LE.K_562
LE.HL_60
LE.CCRF_CEM
LC.NCI_H522
LC.NCI_H460
LC.NCI_H322M
LC.NCI_H23
LC.HOP_92
LC.EKVX
LC.A549
CO.SW_620
CO.KM12
CO.HT29
CO.HCT_15
CO.HCT_116
CO.HCC_2998
CO.COLO205
CNS.U251
CNS.SNB_19
CNS.SF_539
CNS.SF_295
CNS.SF_268
BR.MDA_MB_231
BR.MCF7
BR.HS578T
BR.BT_549
−4 −2 0 2 4
Observed (Z−scores)
Predicted (Z−scores)

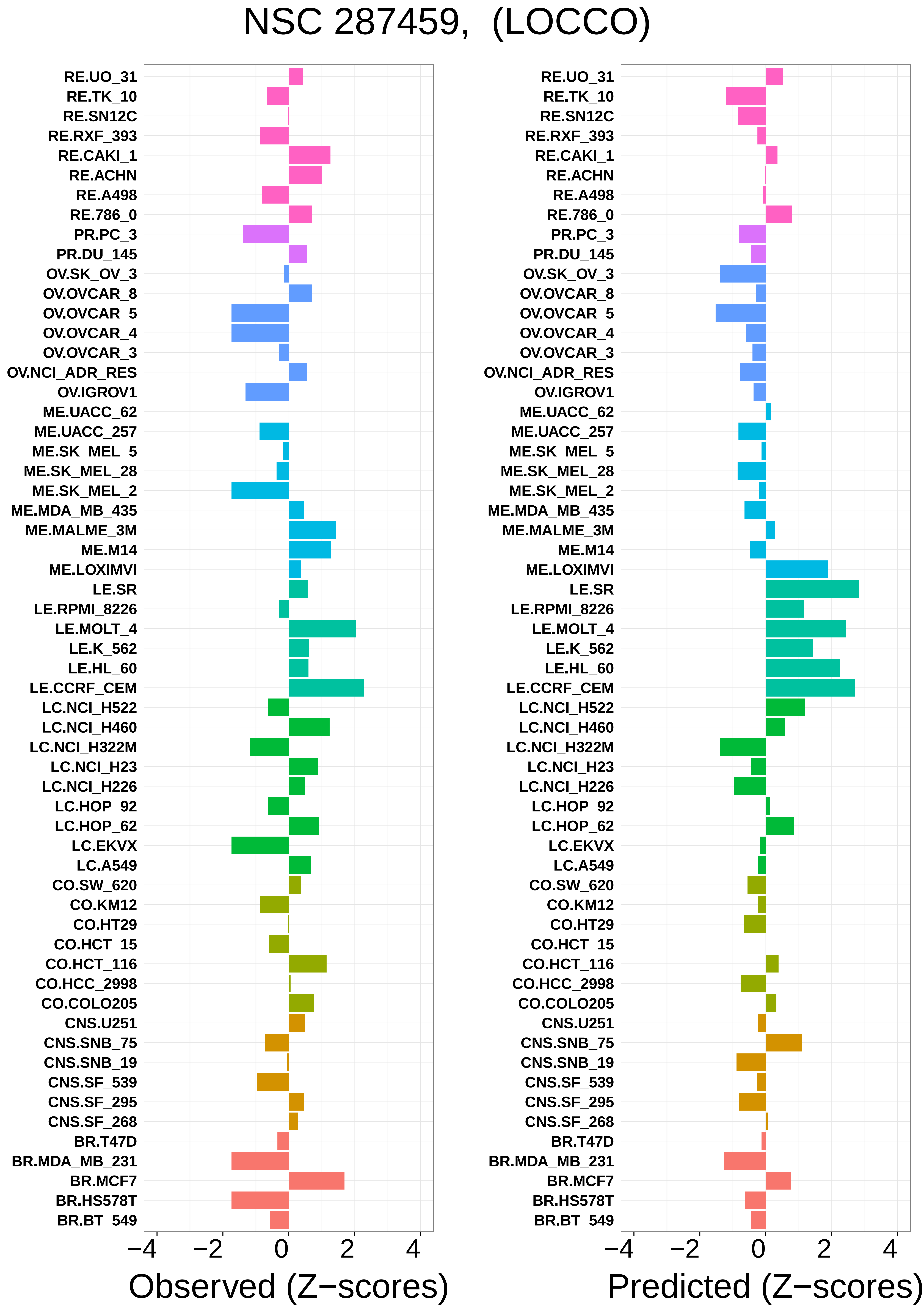
NSC 287459, (LOCCO)
RE.UO_31
RE.TK_10
RE.SN12C
RE.RXF_393
RE.CAKI_1
RE.ACHN
RE.A498
RE.786_0
PR.PC_3
PR.DU_145
OV.SK_OV_3
OV.OVCAR_8
OV.OVCAR_5
OV.OVCAR_4
OV.OVCAR_3
OV.NCI_ADR_RES
OV.IGROV1
ME.UACC_62
ME.UACC_257
ME.SK_MEL_5
ME.SK_MEL_28
ME.SK_MEL_2
ME.MDA_MB_435
ME.MALME_3M
ME.M14
ME.LOXIMVI
LE.SR
LE.RPMI_8226
LE.MOLT_4
LE.K_562
LE.HL_60
LE.CCRF_CEM
LC.NCI_H522
LC.NCI_H460
LC.NCI_H322M
LC.NCI_H23
LC.NCI_H226
LC.HOP_92
LC.HOP_62
LC.EKVX
LC.A549
CO.SW_620
CO.KM12
CO.HT29
CO.HCT_15
CO.HCT_116
CO.HCC_2998
CO.COLO205
CNS.U251
CNS.SNB_75
CNS.SNB_19
CNS.SF_539
CNS.SF_295
CNS.SF_268
BR.T47D
BR.MDA_MB_231
BR.MCF7
BR.HS578T
BR.BT_549
−4 −2 0 2 4
Observed (Z−scores)
Predicted (Z−scores)

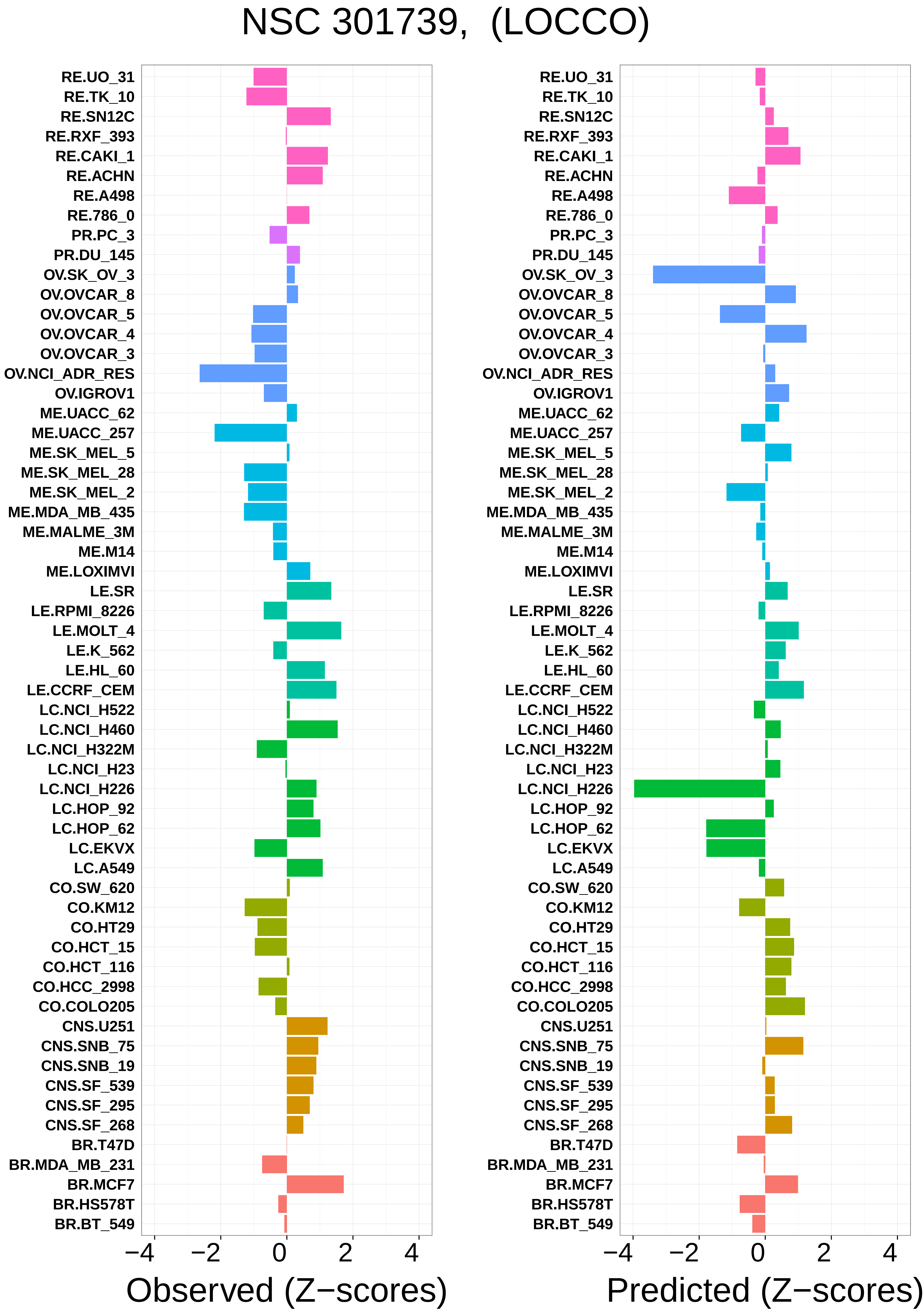
NSC 301739, (LOCCO)
RE.UO_31
RE.TK_10
RE.SN12C
RE.RXF_393
RE.CAKI_1
RE.ACHN
RE.A498
RE.786_0
PR.PC_3
PR.DU_145
OV.SK_OV_3
OV.OVCAR_8
OV.OVCAR_5
OV.OVCAR_4
OV.OVCAR_3
OV.NCI_ADR_RES
OV.IGROV1
ME.UACC_62
ME.UACC_257
ME.SK_MEL_5
ME.SK_MEL_28
ME.SK_MEL_2
ME.MDA_MB_435
ME.MALME_3M
ME.M14
ME.LOXIMVI
LE.SR
LE.RPMI_8226
LE.MOLT_4
LE.K_562
LE.HL_60
LE.CCRF_CEM
LC.NCI_H522
LC.NCI_H460
LC.NCI_H322M
LC.NCI_H23
LC.NCI_H226
LC.HOP_92
LC.HOP_62
LC.EKVX
LC.A549
CO.SW_620
CO.KM12
CO.HT29
CO.HCT_15
CO.HCT_116
CO.HCC_2998
CO.COLO205
CNS.U251
CNS.SNB_75
CNS.SNB_19
CNS.SF_539
CNS.SF_295
CNS.SF_268
BR.T47D
BR.MDA_MB_231
BR.MCF7
BR.HS578T
BR.BT_549
−4
−2
0
2
4
Observed (Z−scores)
Predicted (Z−scores)

NSC 312887, Fludarabine_phosphate_(USAN) (LOCCO)

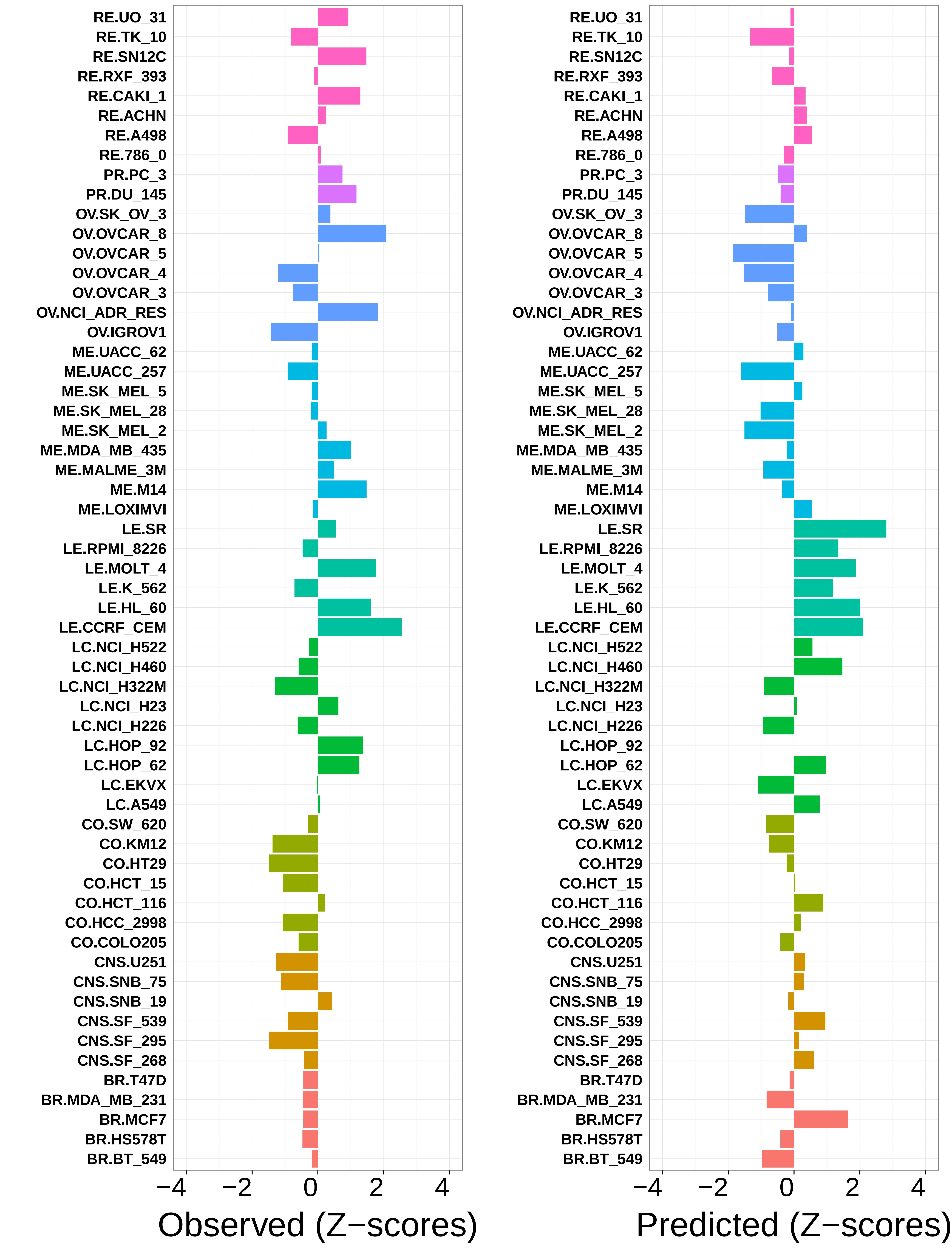

NSC 330507, Tanespimycin (LOCCO)

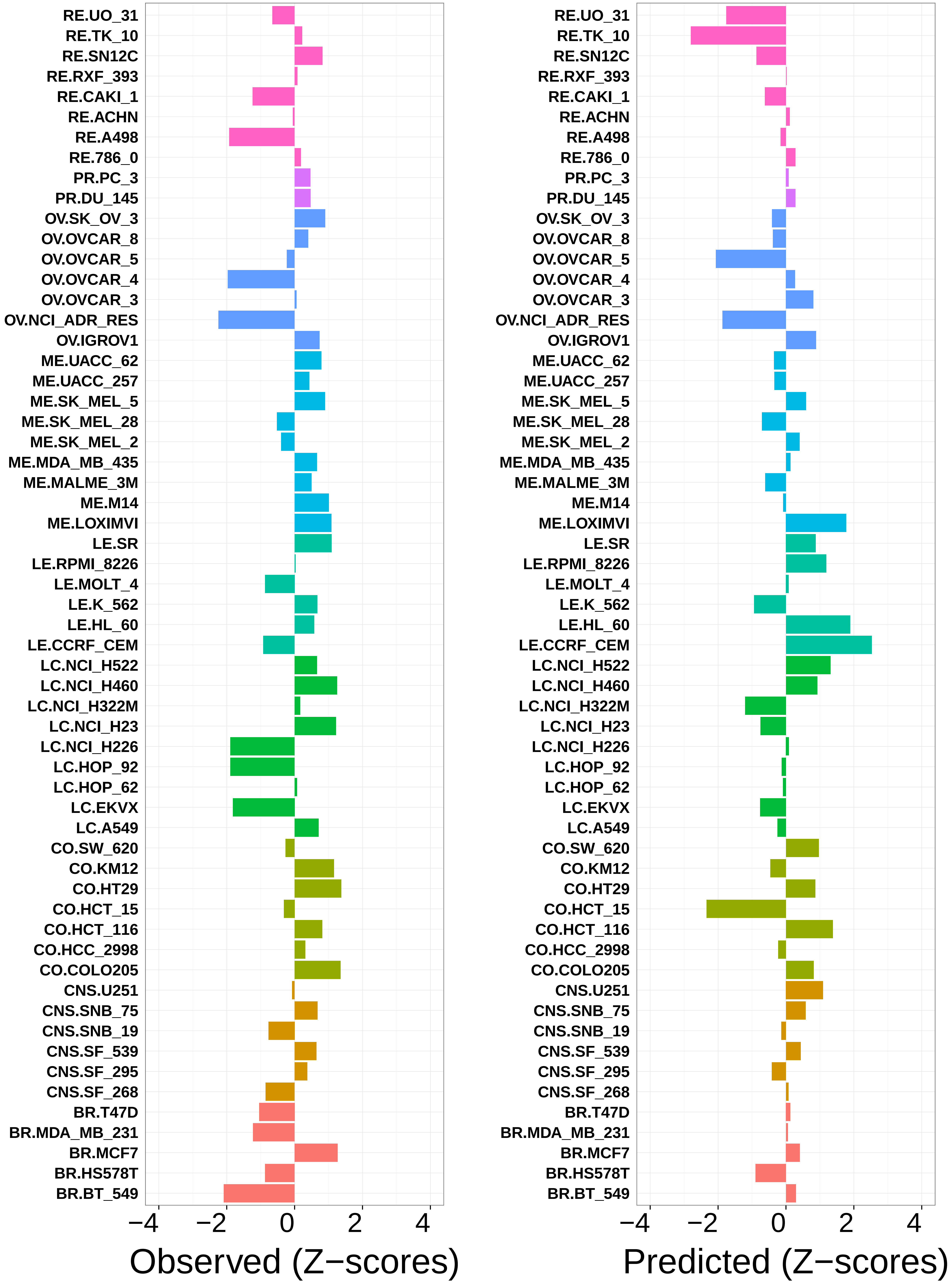

NSC 34521, Dextromethorphan_Hydrobromide (LOCCO)

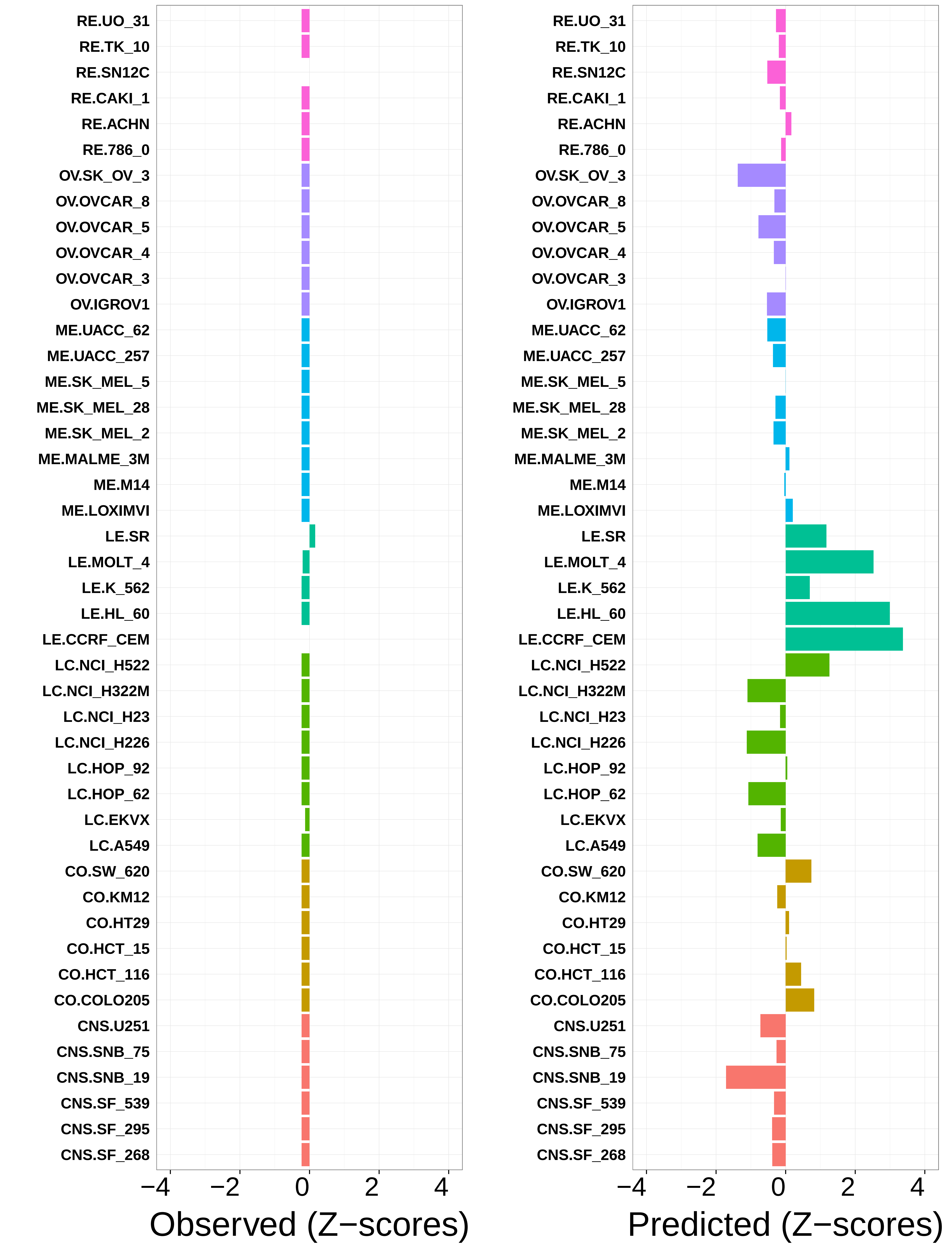

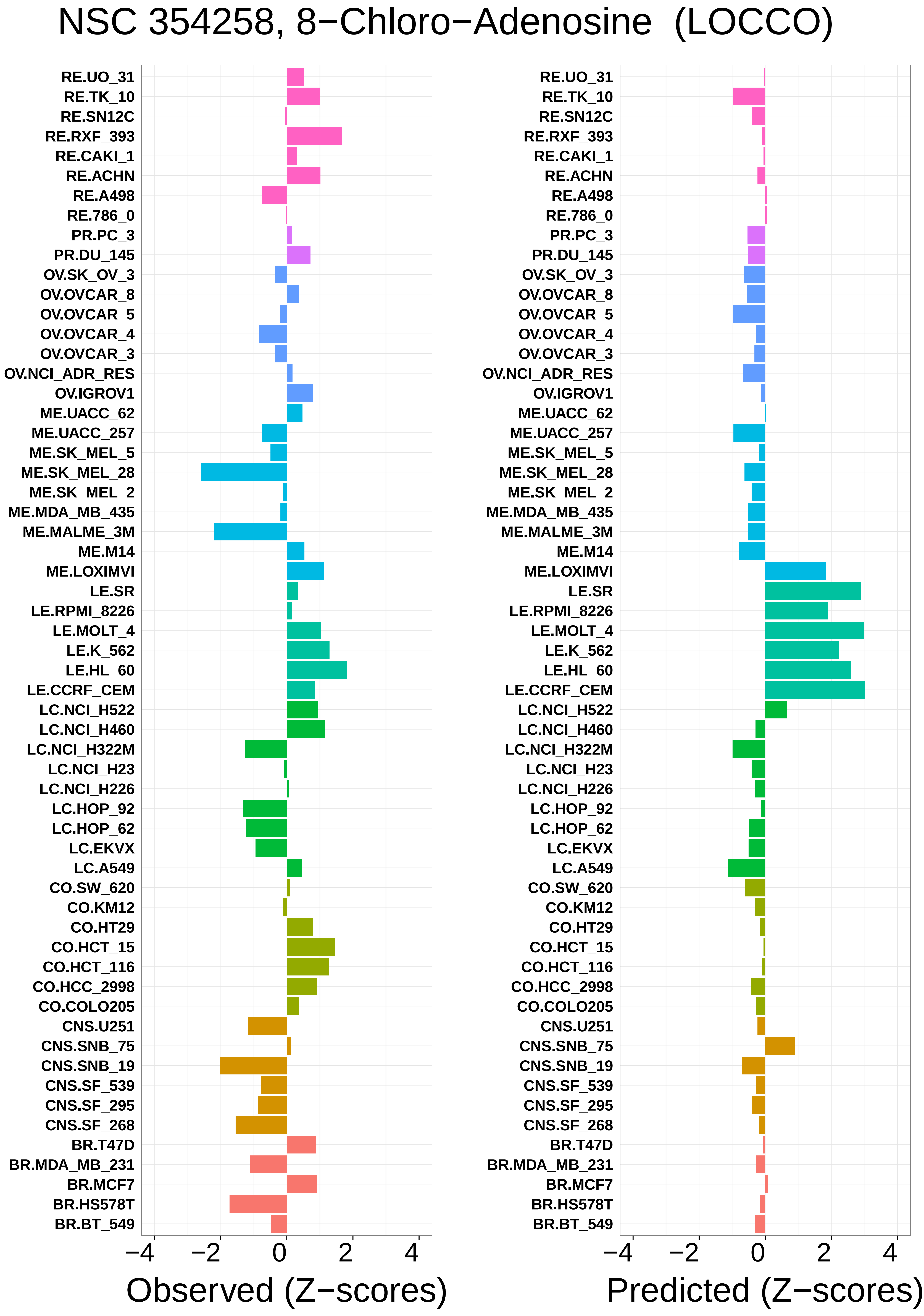
NSC 354258, 8−Chloro−Adenosine (LOCCO)
RE.UO_31
RE.TK_10
RE.SN12C
RE.RXF_393
RE.CAKI_1
RE.ACHN
RE.A498
RE.786_0
PR.PC_3
PR.DU_145
OV.SK_OV_3
OV.OVCAR_8
OV.OVCAR_5
OV.OVCAR_4
OV.OVCAR_3
OV.NCI_ADR_RES
OV.IGROV1
ME.UACC_62
ME.UACC_257
ME.SK_MEL_5
ME.SK_MEL_28
ME.SK_MEL_2
ME.MDA_MB_435
ME.MALME_3M
ME.M14
ME.LOXIMVI
LE.SR
LE.RPMI_8226
LE.MOLT_4
LE.K_562
LE.HL_60
LE.CCRF_CEM
LC.NCI_H522
LC.NCI_H460
LC.NCI_H322M
LC.NCI_H23
LC.NCI_H226
LC.HOP_92
LC.HOP_62
LC.EKVX
LC.A549
CO.SW_620
CO.KM12
CO.HT29
CO.HCT_15
CO.HCT_116
CO.HCC_2998
CO.COLO205
CNS.U251
CNS.SNB_75
CNS.SNB_19
CNS.SF_539
CNS.SF_295
CNS.SF_268
BR.T47D
BR.MDA_MB_231
BR.MCF7
BR.HS578T
BR.BT_549
−4 −2 0 2 4
Observed (Z−scores)
−4 −2 0 2 4
Predicted (Z−scores)

NSC 354462, Hypothemycin (LOCCO)

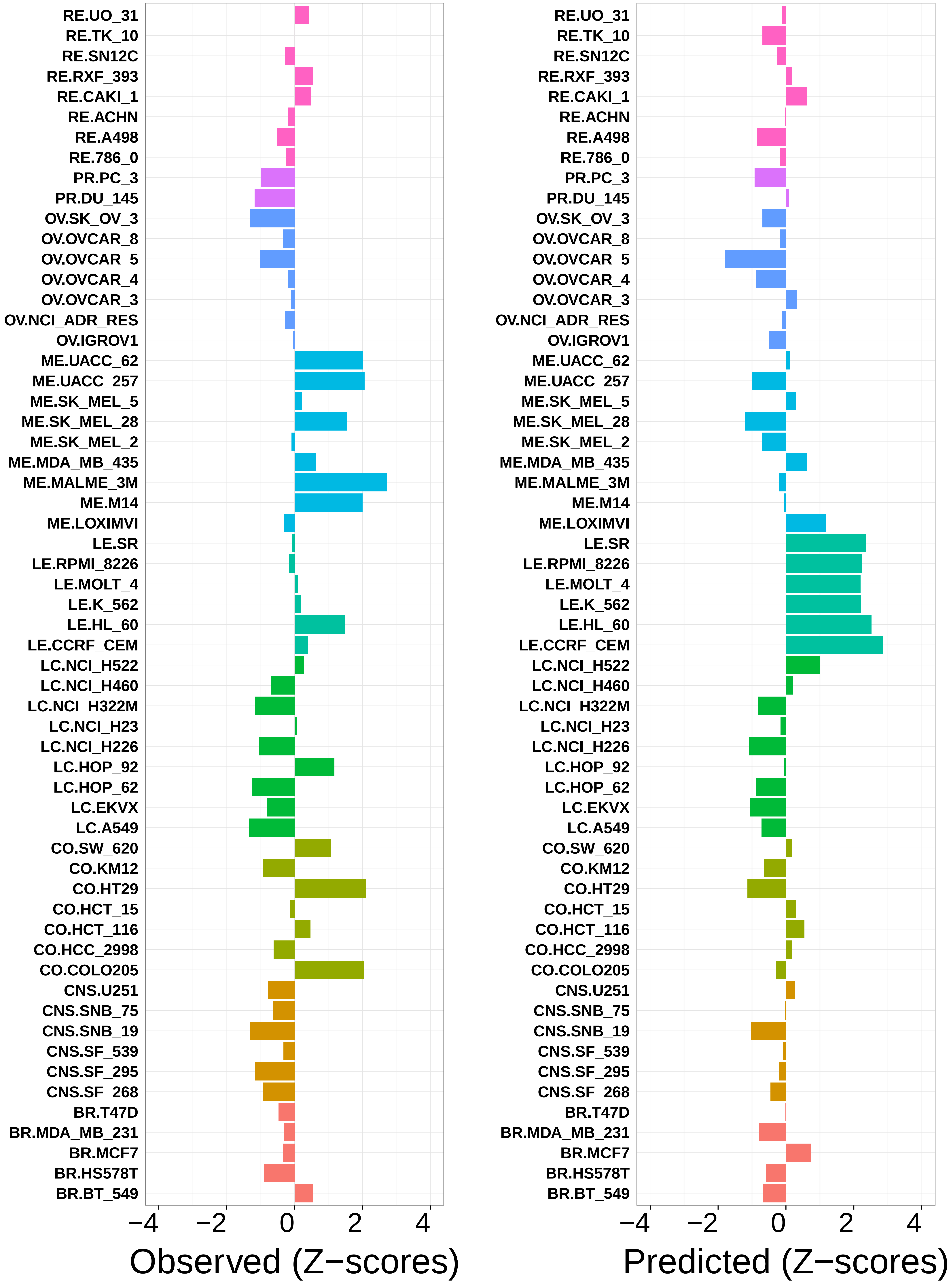

NSC 376128, Dolastatin_10 (LOCCO)

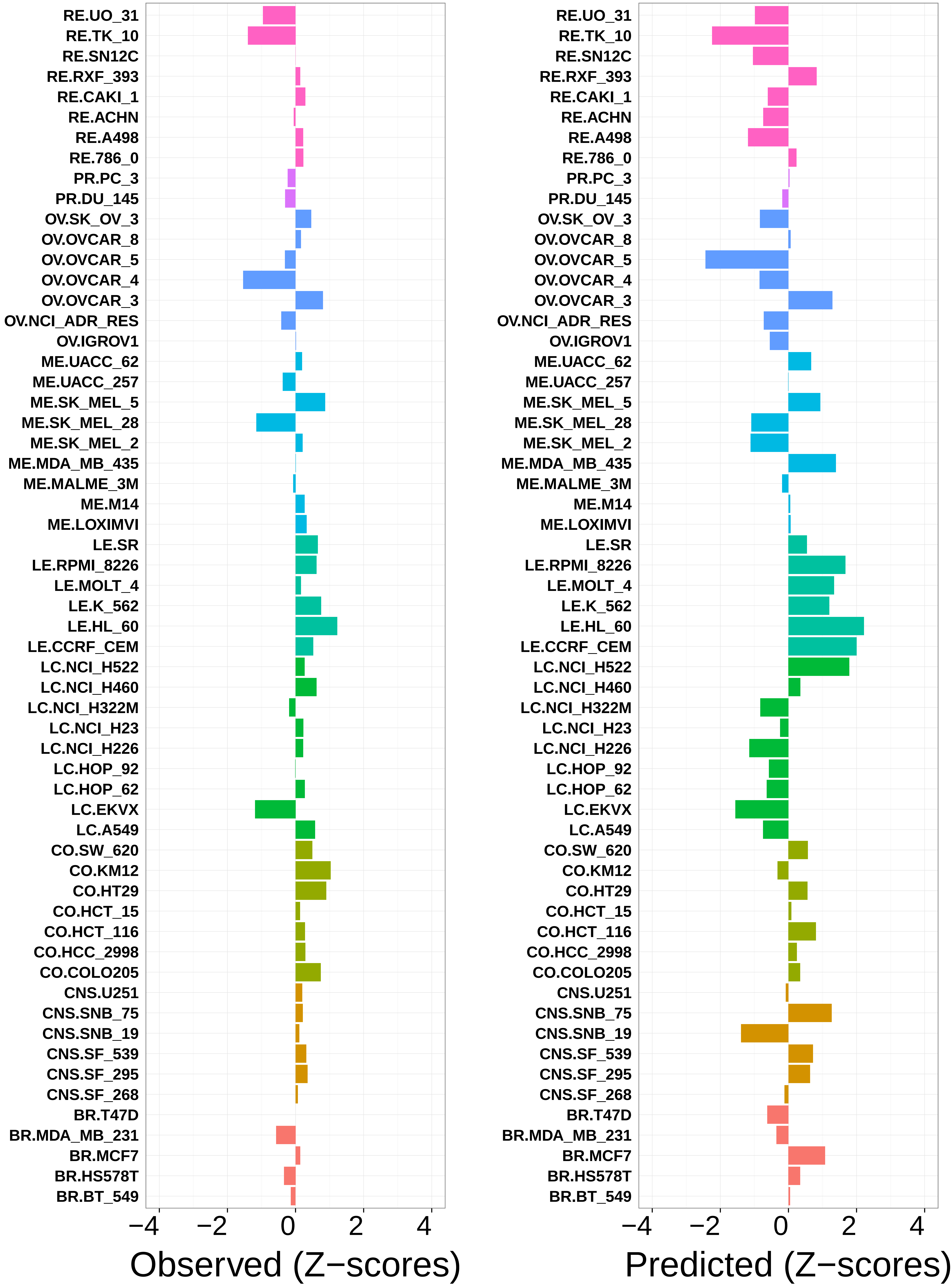

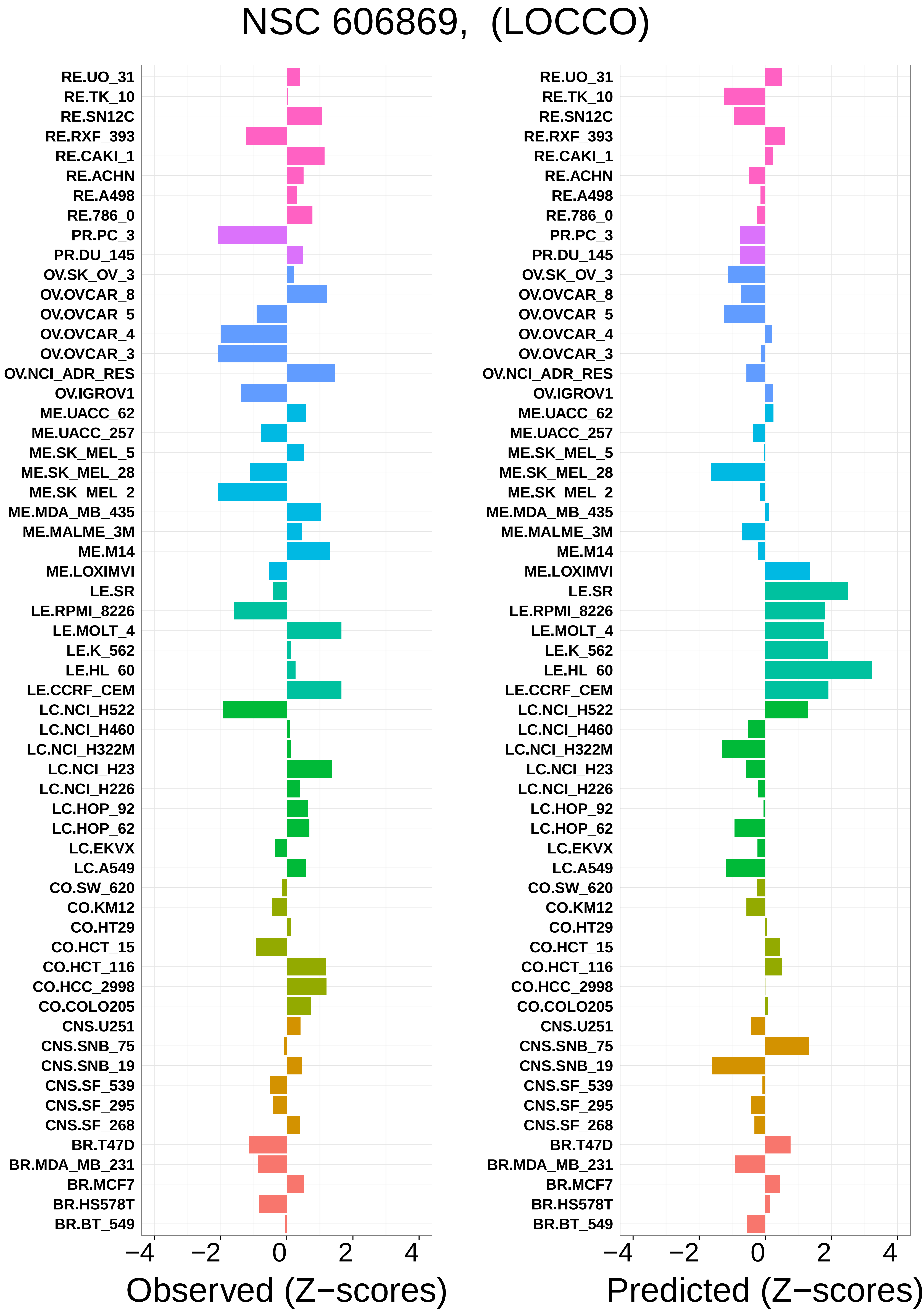
NSC 606869, (LOCCO)
RE.UO_31
RE.TK_10
RE.SN12C
RE.RXF_393
RE.CAKI_1
RE.ACHN
RE.A498
RE.786_0
PR.PC_3
PR.DU_145
OV.SK_OV_3
OV.OVCAR_8
OV.OVCAR_5
OV.OVCAR_4
OV.OVCAR_3
OV.NCI_ADR_RES
OV.IGROV1
ME.UACC_62
ME.UACC_257
ME.SK_MEL_5
ME.SK_MEL_28
ME.SK_MEL_2
ME.MDA_MB_435
ME.MALME_3M
ME.M14
ME.LOXIMVI
LE.SR
LE.RPMI_8226
LE.MOLT_4
LE.K_562
LE.HL_60
LE.CCRF_CEM
LC.NCI_H522
LC.NCI_H460
LC.NCI_H322M
LC.NCI_H23
LC.NCI_H226
LC.HOP_92
LC.HOP_62
LC.EKVX
LC.A549
CO.SW_620
CO.KM12
CO.HT29
CO.HCT_15
CO.HCT_116
CO.HCC_2998
CO.COLO205
CNS.U251
CNS.SNB_75
CNS.SNB_19
CNS.SF_539
CNS.SF_295
CNS.SF_268
BR.T47D
BR.MDA_MB_231
BR.MCF7
BR.HS578T
BR.BT_549
−4 −2 0 2 4
Observed (Z−scores)
−4 −2 0 2 4
Predicted (Z−scores)

NSC 609699, Topotecan_Hydrochloride (LOCCO)

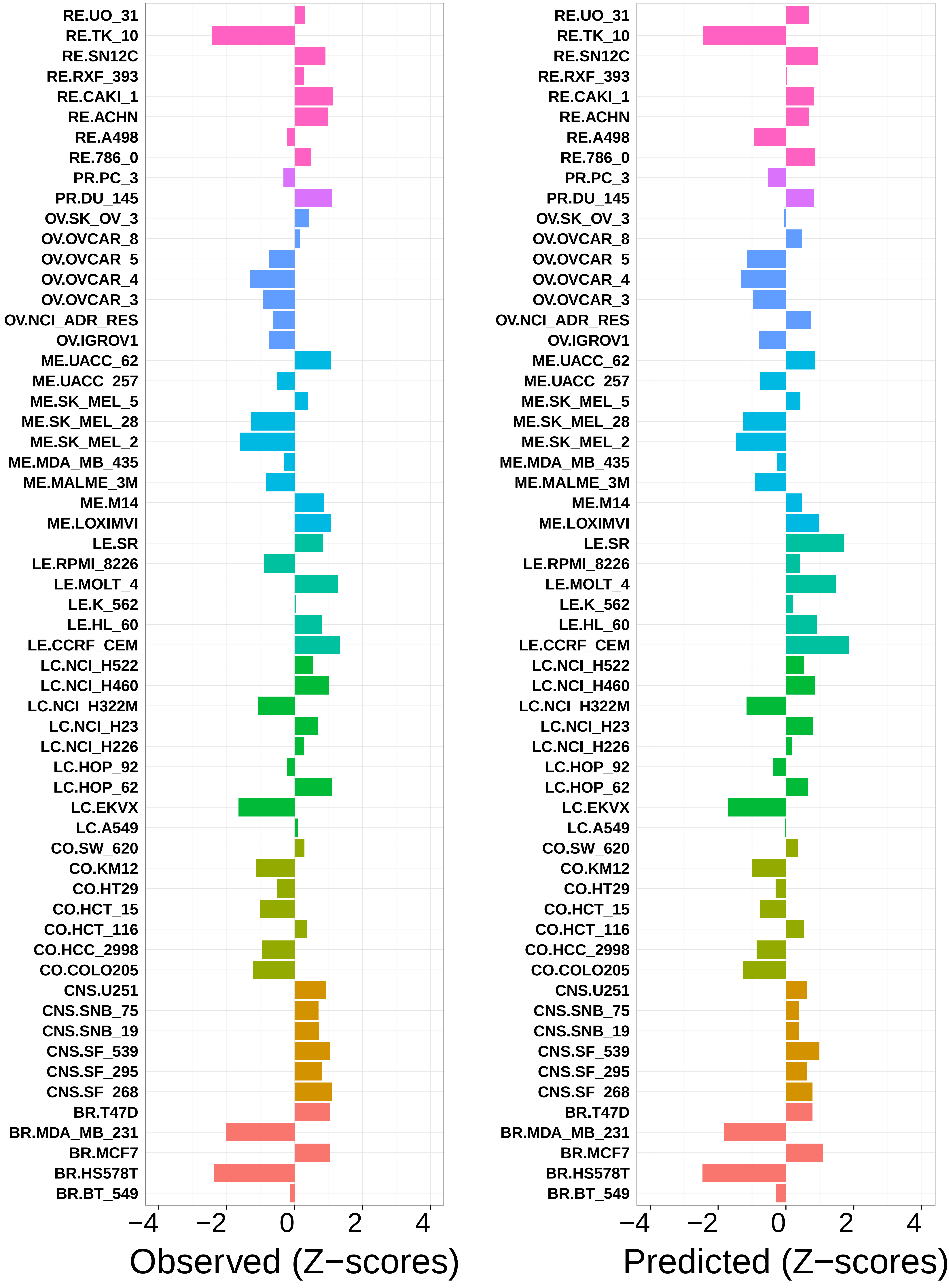

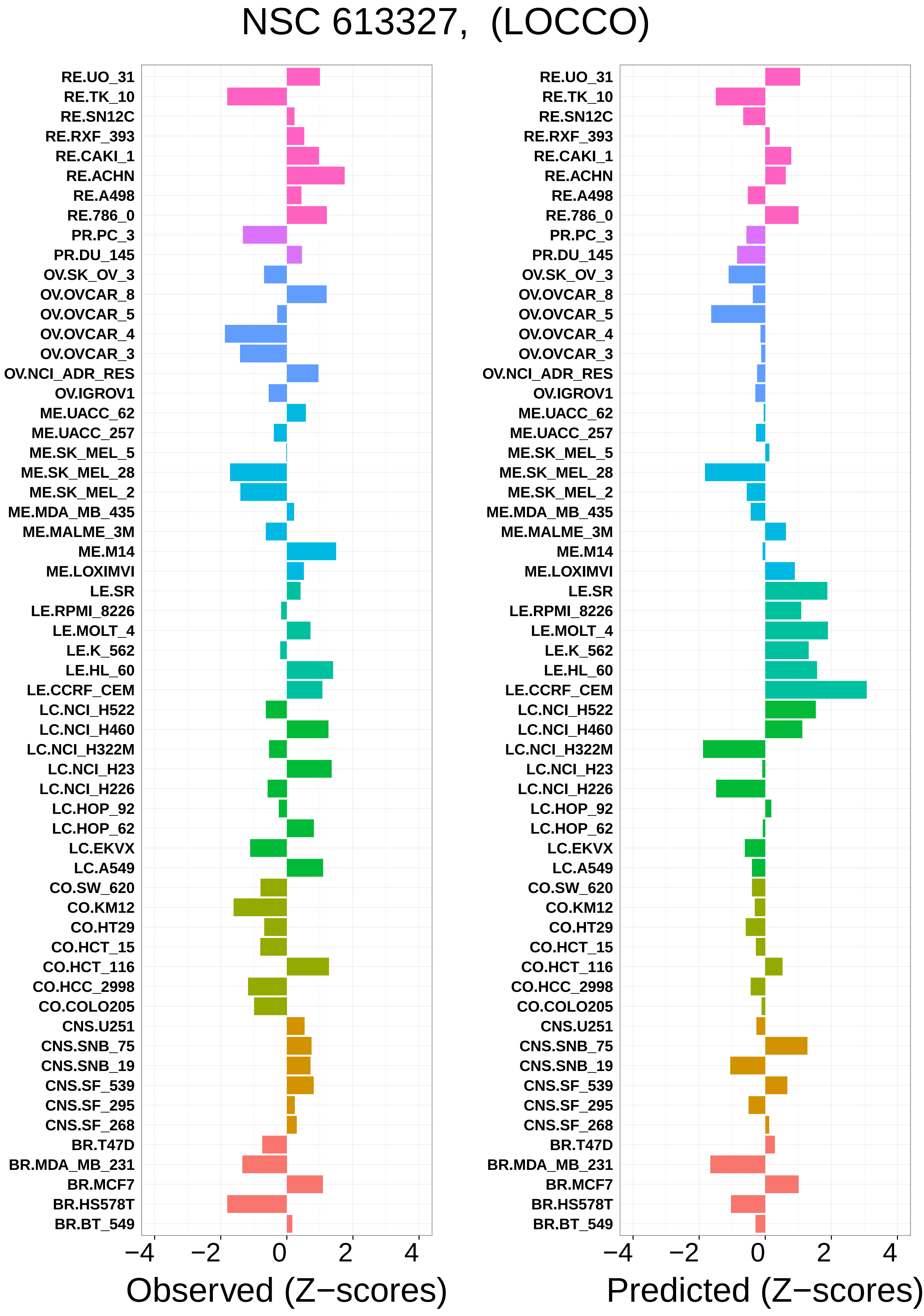
NSC 613327, (LOCCO)
RE.UO_31
RE.TK_10
RE.SN12C
RE.RXF_393
RE.CAKI_1
RE.ACHN
RE.A498
RE.786_0
PR.PC_3
PR.DU_145
OV.SK_OV_3
OV.OVCAR_8
OV.OVCAR_5
OV.OVCAR_4
OV.OVCAR_3
OV.NCI_ADR_RES
OV.IGROV1
ME.UACC_62
ME.UACC_257
ME.SK_MEL_5
ME.SK_MEL_28
ME.SK_MEL_2
ME.MDA_MB_435
ME.MALME_3M
ME.M14
ME.LOXIMVI
LE.SR
LE.RPMI_8226
LE.MOLT_4
LE.K_562
LE.HL_60
LE.CCRF_CEM
LC.NCI_H522
LC.NCI_H460
LC.NCI_H322M
LC.NCI_H23
LC.NCI_H226
LC.HOP_92
LC.HOP_62
LC.EKVX
LC.A549
CO.SW_620
CO.KM12
CO.HT29
CO.HCT_15
CO.HCT_116
CO.HCC_2998
CO.COLO205
CNS.U251
CNS.SNB_75
CNS.SNB_19
CNS.SF_539
CNS.SF_295
CNS.SF_268
BR.T47D
BR.MDA_MB_231
BR.MCF7
BR.HS578T
BR.BT_549
−4 −2 0 2 4
Observed (Z−scores)
Predicted (Z−scores)

NSC 628503, Docetaxel (LOCCO)

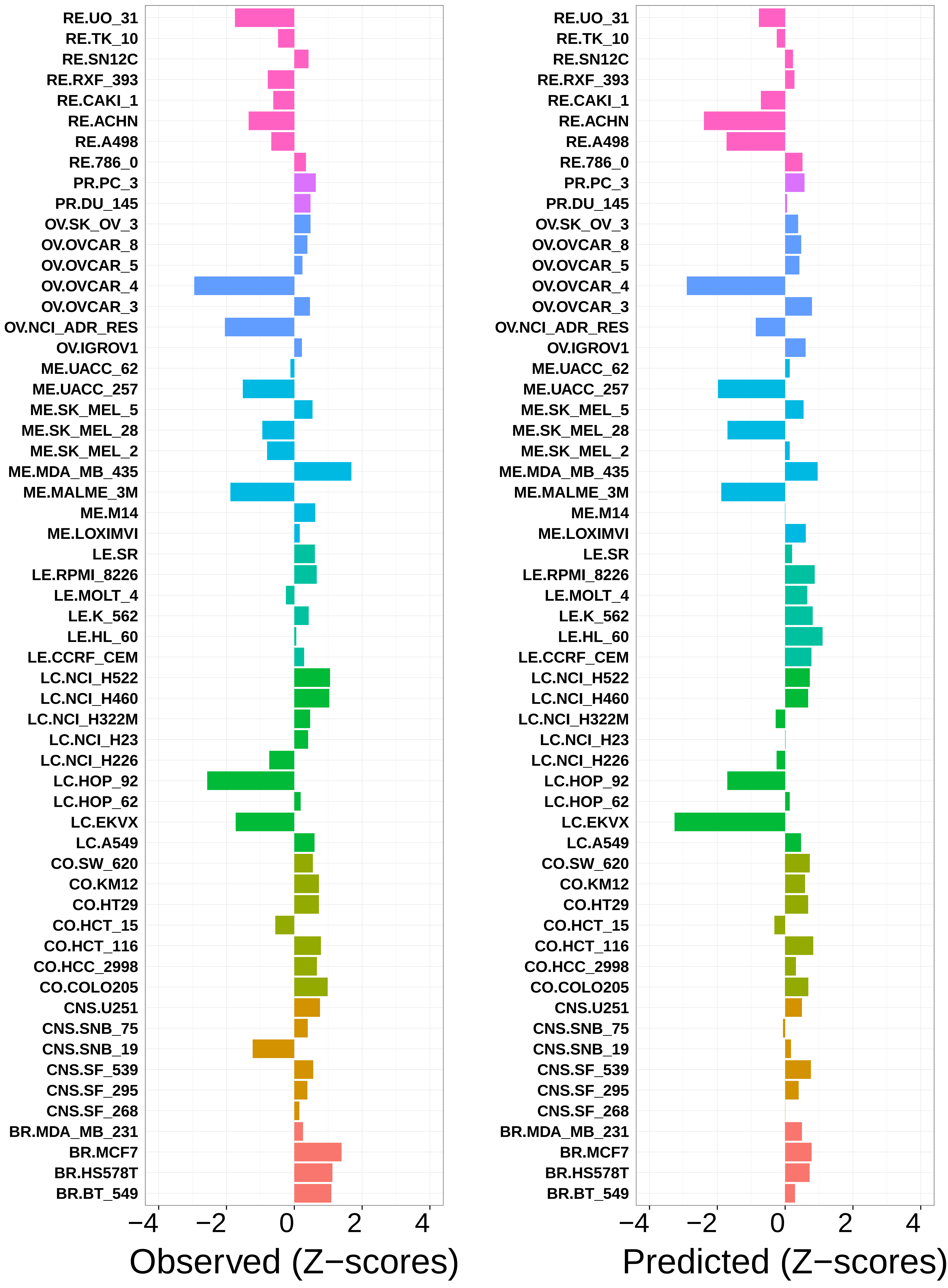

NSC 630176, Romidepsin (LOCCO)

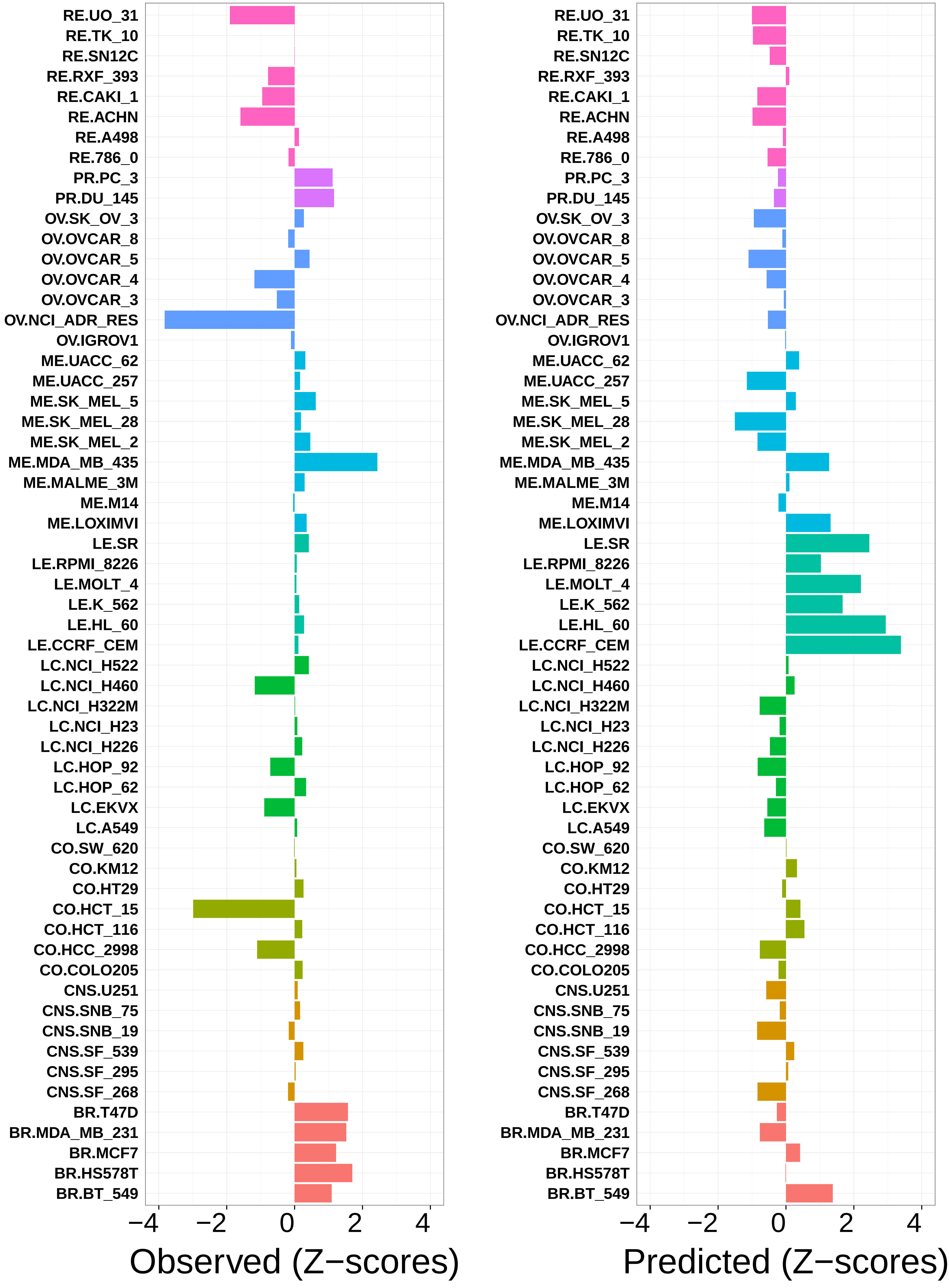

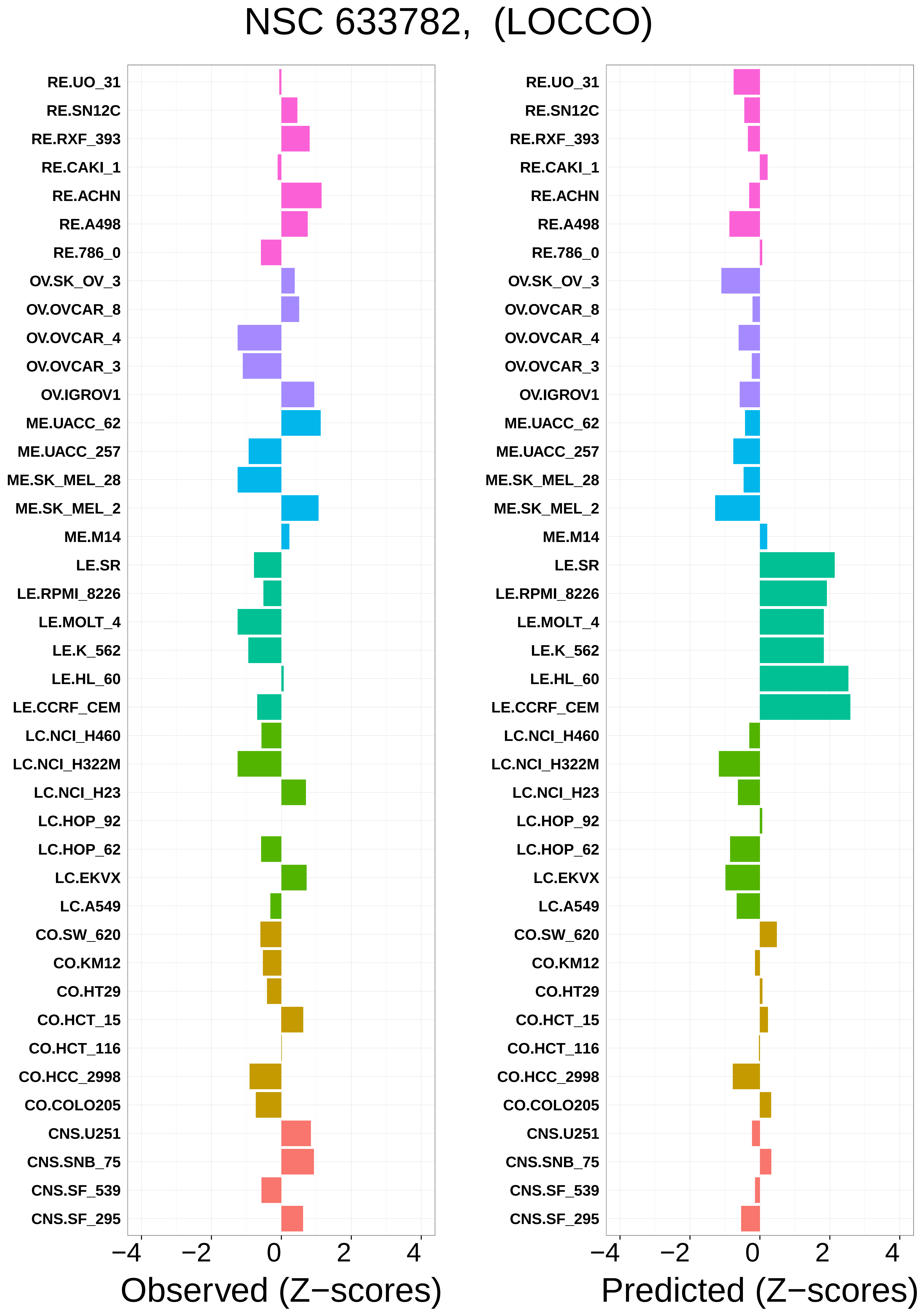

NSC 633782, (LOCCO)
RE.UO_31
RE.SN12C
RE.RXF_393
RE.CAKI_1
RE.ACHN
RE.A498
RE.786_0
OV.SK_OV_3
OV.OVCAR_8
OV.OVCAR_4
OV.OVCAR_3
OV.IGROV1
ME.UACC_62
ME.UACC_257
ME.SK_MEL_28
ME.SK_MEL_2
ME.M14
LE.SR
LE.RPMI_8226
LE.MOLT_4
LE.K_562
LE.HL_60
LE.CCRF_CEM
LC.NCI_H460
LC.NCI_H322M
LC.NCI_H23
LC.HOP_92
LC.HOP_62
LC.EKVX
LC.A549
CO.SW_620
CO.KM12
CO.HT29
CO.HCT_15
CO.HCT_116
CO.HCC_2998
CO.COLO205
CNS.U251
CNS.SNB_75
CNS.SF_539
CNS.SF_295
−4 −2 0 2 4
Observed (Z−scores)
Predicted (Z−scores)

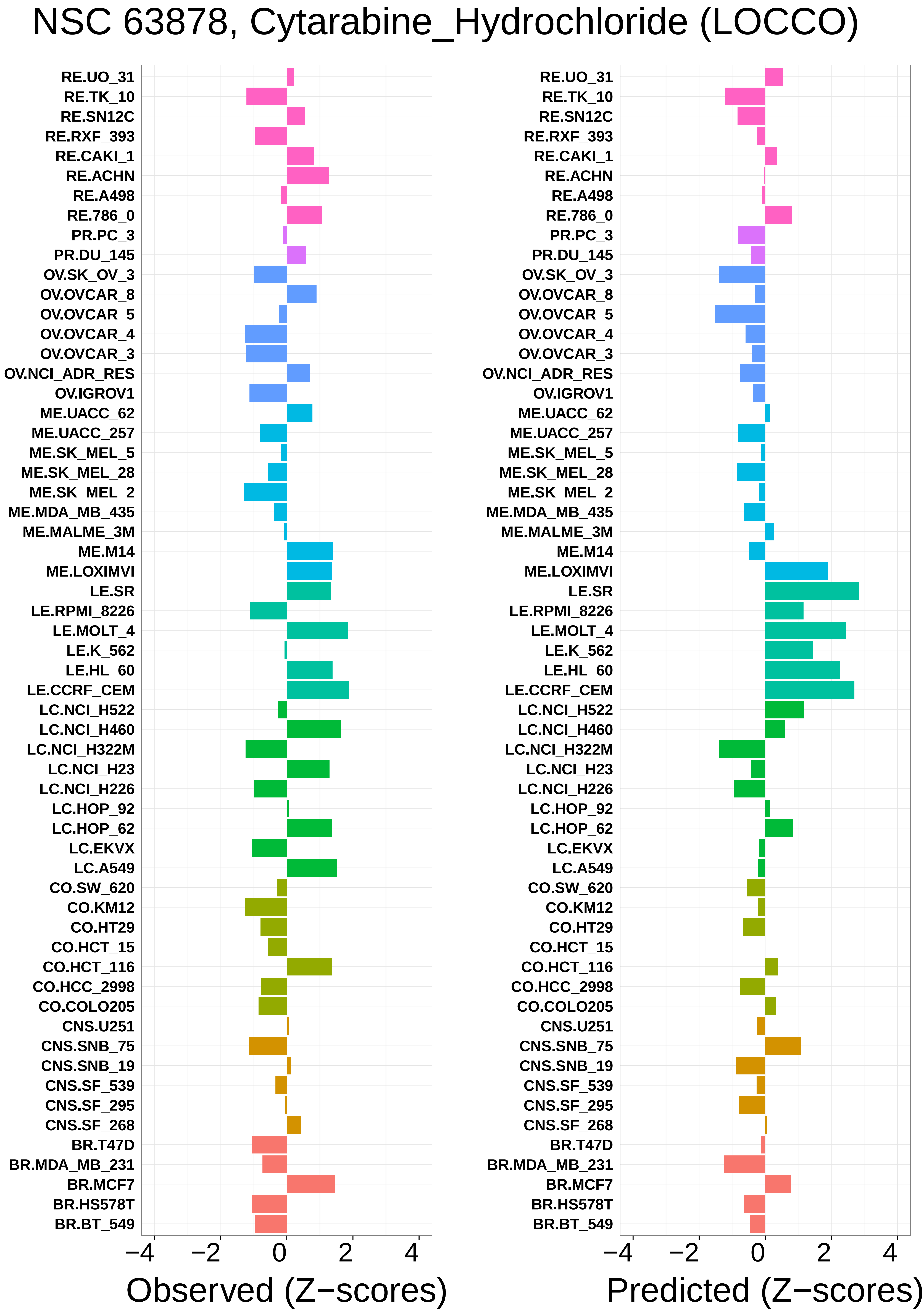
NSC 63878, Cytarabine_Hydrochloride (LOCCO)
RE.UO_31
RE.TK_10
RE.SN12C
RE.RXF_393
RE.CAKI_1
RE.ACHN
RE.A498
RE.786_0
PR.PC_3
PR.DU_145
OV.SK_OV_3
OV.OVCAR_8
OV.OVCAR_5
OV.OVCAR_4
OV.OVCAR_3
OV.NCI_ADR_RES
OV.IGROV1
ME.UACC_62
ME.UACC_257
ME.SK_MEL_5
ME.SK_MEL_28
ME.SK_MEL_2
ME.MDA_MB_435
ME.MALME_3M
ME.M14
ME.LOXIMVI
LE.SR
LE.RPMI_8226
LE.MOLT_4
LE.K_562
LE.HL_60
LE.CCRF_CEM
LC.NCI_H522
LC.NCI_H460
LC.NCI_H322M
LC.NCI_H23
LC.NCI_H226
LC.HOP_92
LC.HOP_62
LC.EKVX
LC.A549
CO.SW_620
CO.KM12
CO.HT29
CO.HCT_15
CO.HCT_116
CO.HCC_2998
CO.COLO205
CNS.U251
CNS.SNB_75
CNS.SNB_19
CNS.SF_539
CNS.SF_295
CNS.SF_268
BR.T47D
BR.MDA_MB_231
BR.MCF7
BR.HS578T
BR.BT_549
−4
−2
0
2
4
Observed (Z−scores)
Predicted (Z−scores)

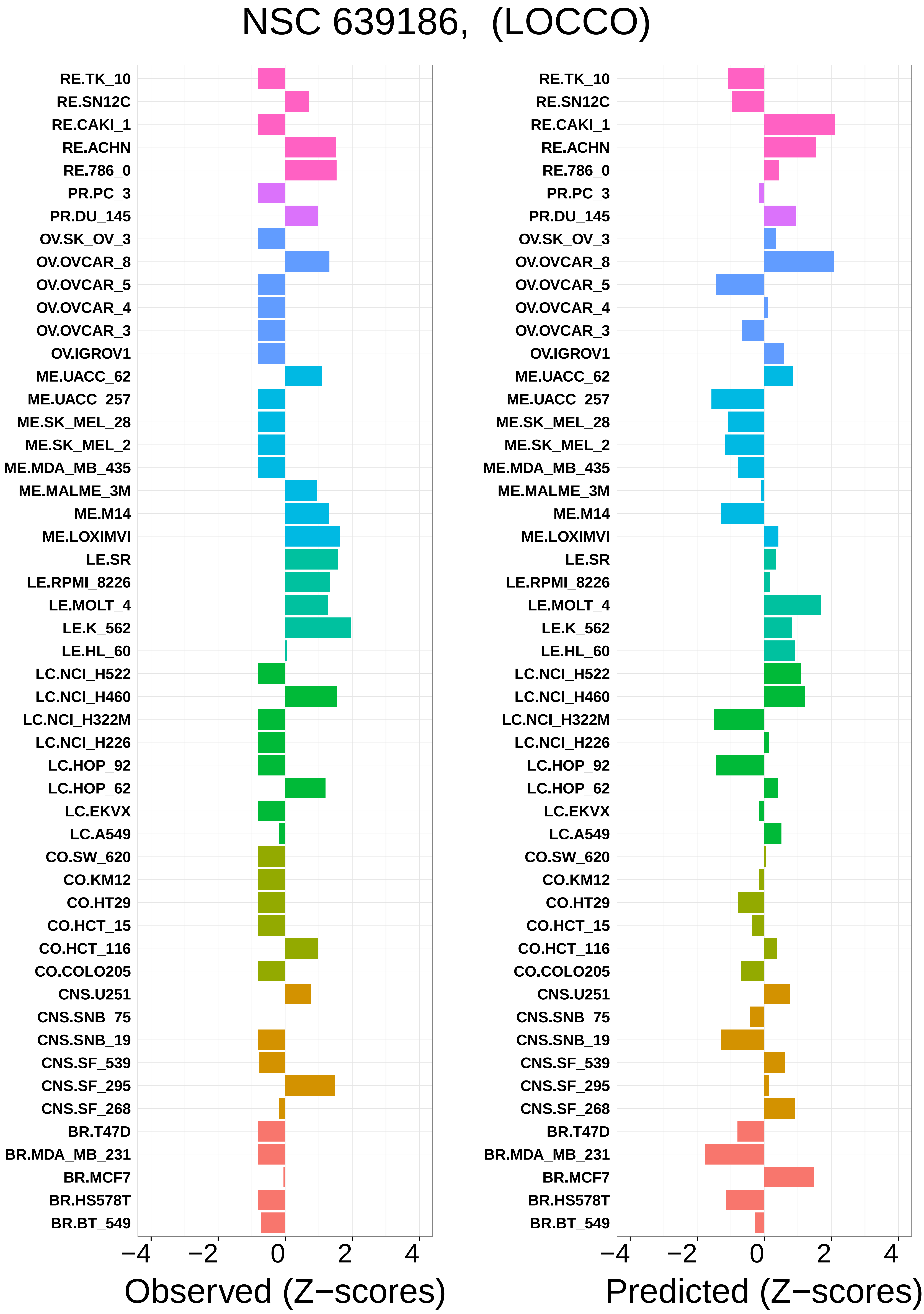

NSC 639186, (LOCCO)
RE.TK_10
RE.SN12C
RE.CAKI_1
RE.ACHN
RE.786_0
PR.PC_3
PR.DU_145
OV.SK_OV_3
OV.OVCAR_8
OV.OVCAR_5
OV.OVCAR_4
OV.OVCAR_3
OV.IGROV1
ME.UACC_62
ME.UACC_257
ME.SK_MEL_28
ME.SK_MEL_2
ME.MDA_MB_435
ME.MALME_3M
ME.M14
ME.LOXIMVI
LE.SR
LE.RPMI_8226
LE.MOLT_4
LE.K_562
LE.HL_60
LC.NCI_H522
LC.NCI_H460
LC.NCI_H322M
LC.NCI_H226
LC.HOP_92
LC.HOP_62
LC.EKVX
LC.A549
CO.SW_620
CO.KM12
CO.HT29
CO.HCT_15
CO.HCT_116
CO.COLO205
CNS.U251
CNS.SNB_75
CNS.SNB_19
CNS.SF_539
CNS.SF_295
CNS.SF_268
BR.T47D
BR.MDA_MB_231
BR.MCF7
BR.HS578T
BR.BT_549
−4 −2 0 2 4
Observed (Z−scores)
−4 −2 0 2 4
Predicted (Z−scores)

NSC 656576, (LOCCO)

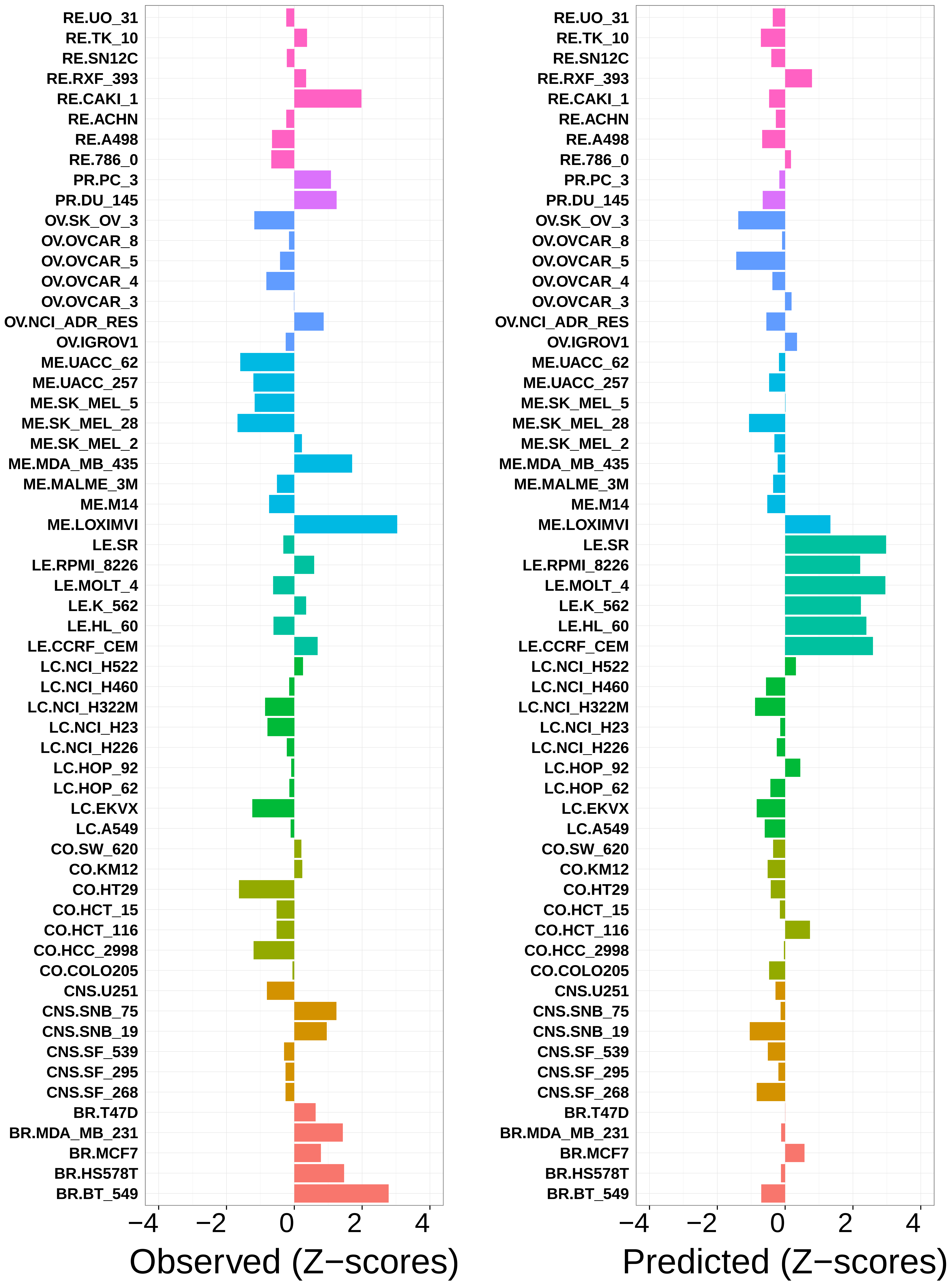

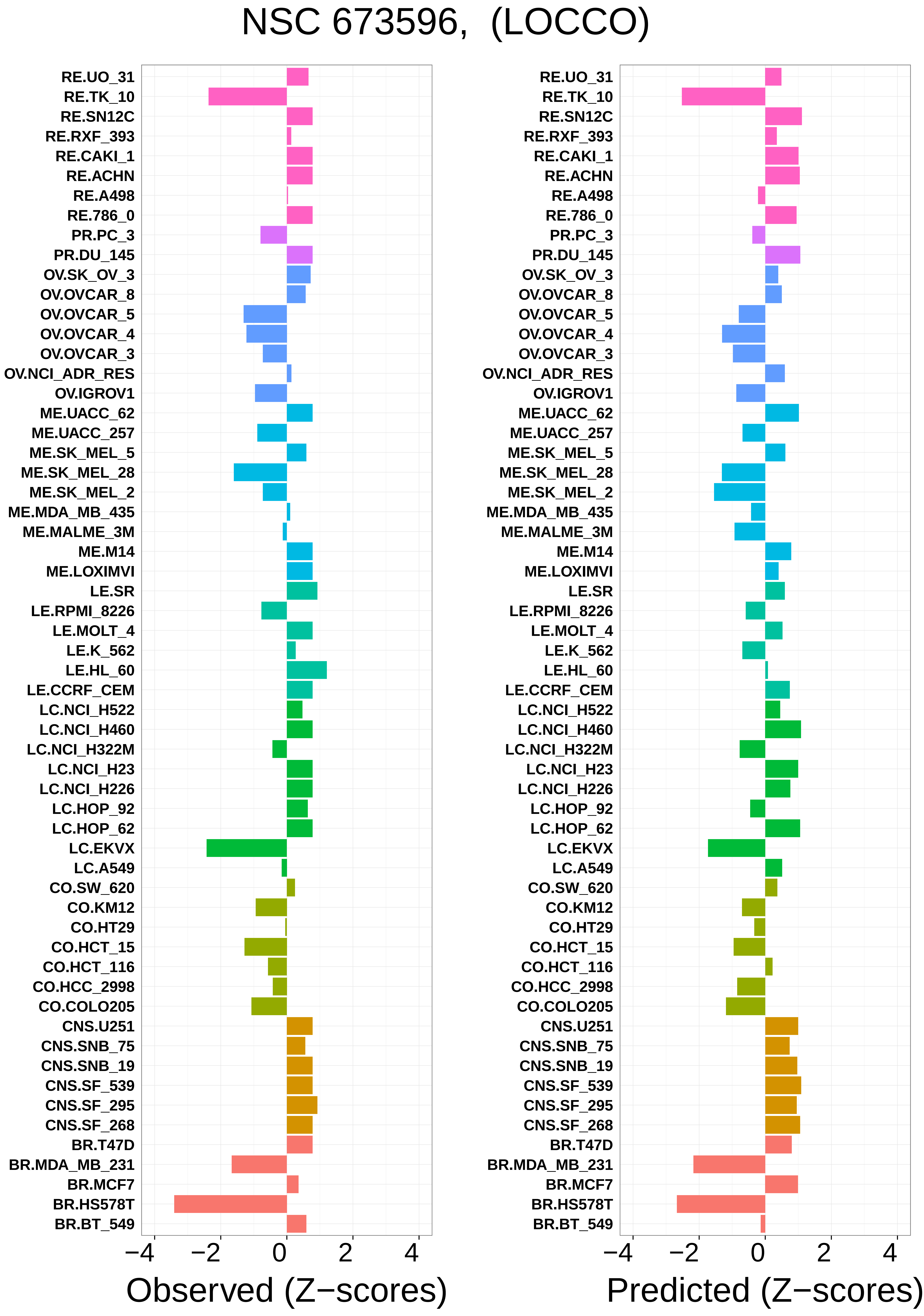
NSC 673596, (LOCCO)
RE.UO_31
RE.TK_10
RE.SN12C
RE.RXF_393
RE.CAKI_1
RE.ACHN
RE.A498
RE.786_0
PR.PC_3
PR.DU_145
OV.SK_OV_3
OV.OVCAR_8
OV.OVCAR_5
OV.OVCAR_4
OV.OVCAR_3
OV.NCI_ADR_RES
OV.IGROV1
ME.UACC_62
ME.UACC_257
ME.SK_MEL_5
ME.SK_MEL_28
ME.SK_MEL_2
ME.MDA_MB_435
ME.MALME_3M
ME.M14
ME.LOXIMVI
LE.SR
LE.RPMI_8226
LE.MOLT_4
LE.K_562
LE.HL_60
LE.CCRF_CEM
LC.NCI_H522
LC.NCI_H460
LC.NCI_H322M
LC.NCI_H23
LC.NCI_H226
LC.HOP_92
LC.HOP_62
LC.EKVX
LC.A549
CO.SW_620
CO.KM12
CO.HT29
CO.HCT_15
CO.HCT_116
CO.HCC_2998
CO.COLO205
CNS.U251
CNS.SNB_75
CNS.SNB_19
CNS.SF_539
CNS.SF_295
CNS.SF_268
BR.T47D
BR.MDA_MB_231
BR.MCF7
BR.HS578T
BR.BT_549
−4 −2 0 2 4
Observed (Z−scores)
Predicted (Z−scores)

NSC 686288, (LOCCO)

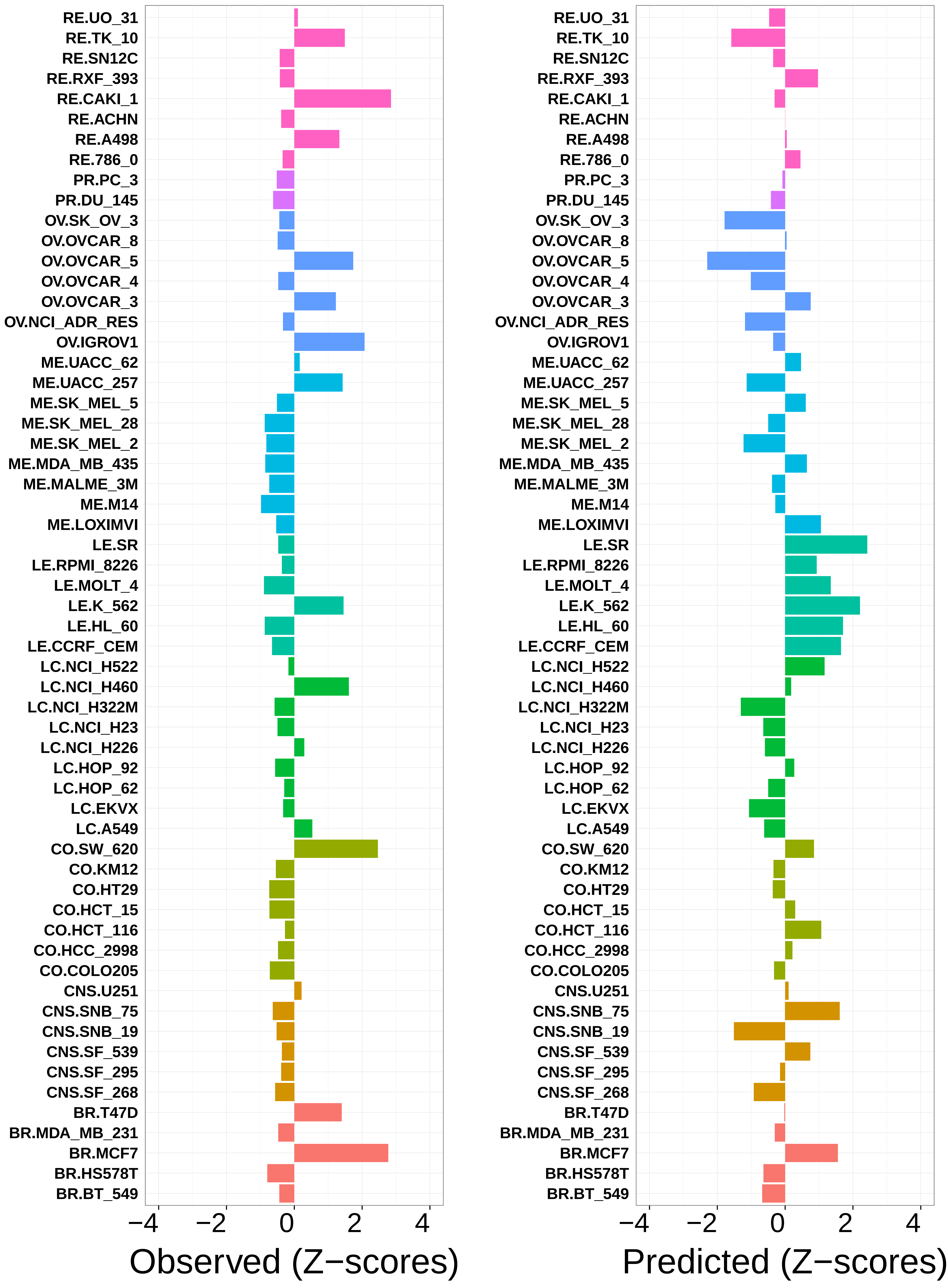

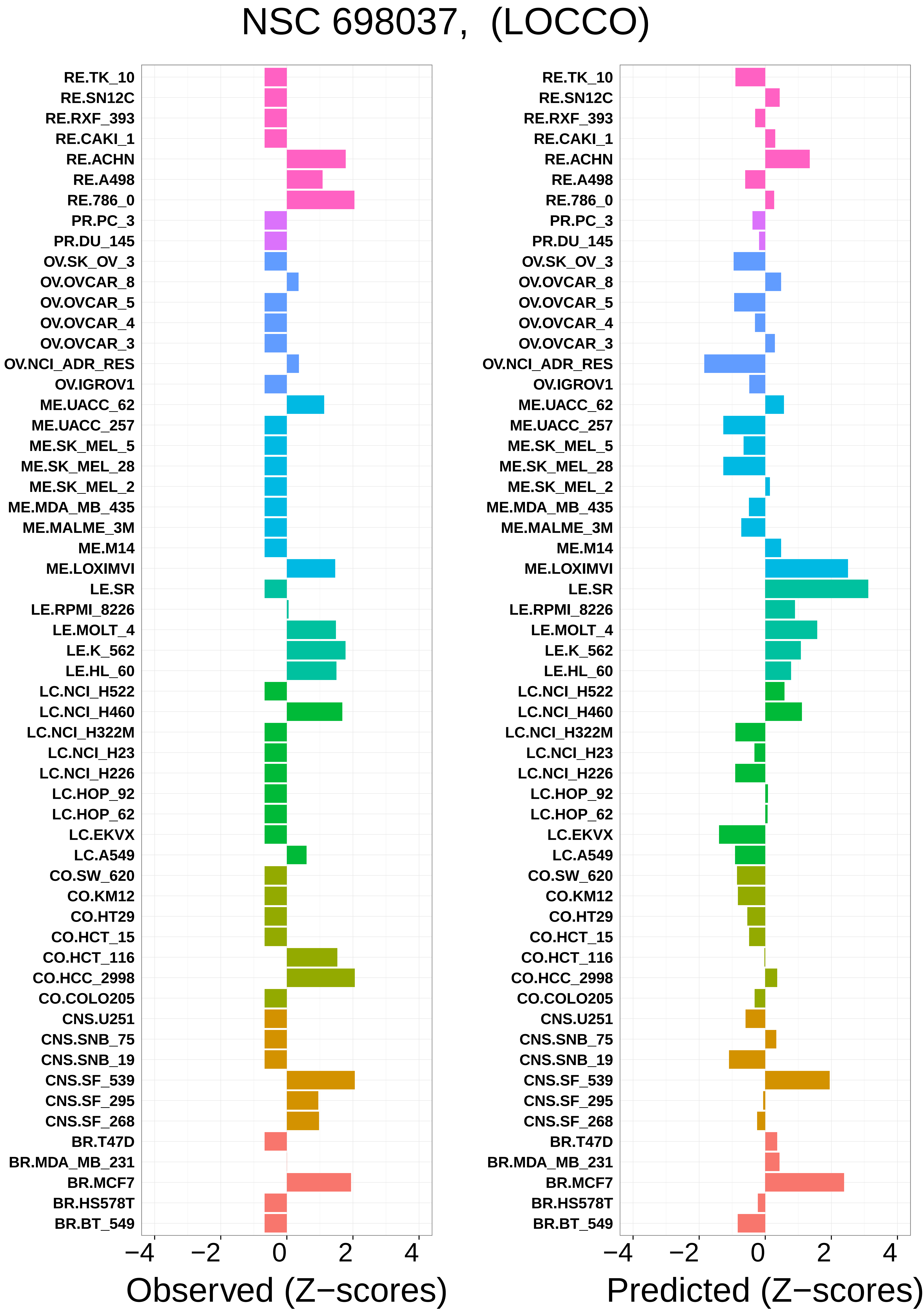
NSC 698037, (LOCCO)
RE.TK_10
RE.SN12C
RE.RXF_393
RE.CAKI_1
RE.ACHN
RE.A498
RE.786_0
PR.PC_3
PR.DU_145
OV.SK_OV_3
OV.OVCAR_8
OV.OVCAR_5
OV.OVCAR_4
OV.OVCAR_3
OV.NCI_ADR_RES
OV.IGROV1
ME.UACC_62
ME.UACC_257
ME.SK_MEL_5
ME.SK_MEL_28
ME.SK_MEL_2
ME.MDA_MB_435
ME.MALME_3M
ME.M14
ME.LOXIMVI
LE.SR
LE.RPMI_8226
LE.MOLT_4
LE.K_562
LE.HL_60
LC.NCI_H522
LC.NCI_H460
LC.NCI_H322M
LC.NCI_H23
LC.NCI_H226
LC.HOP_92
LC.HOP_62
LC.EKVX
LC.A549
CO.SW_620
CO.KM12
CO.HT29
CO.HCT_15
CO.HCT_116
CO.HCC_2998
CO.COLO205
CNS.U251
CNS.SNB_75
CNS.SNB_19
CNS.SF_539
CNS.SF_295
CNS.SF_268
BR.T47D
BR.MDA_MB_231
BR.MCF7
BR.HS578T
BR.BT_549
−4
−2
0
2
4
Observed (Z−scores)
Predicted (Z−scores)

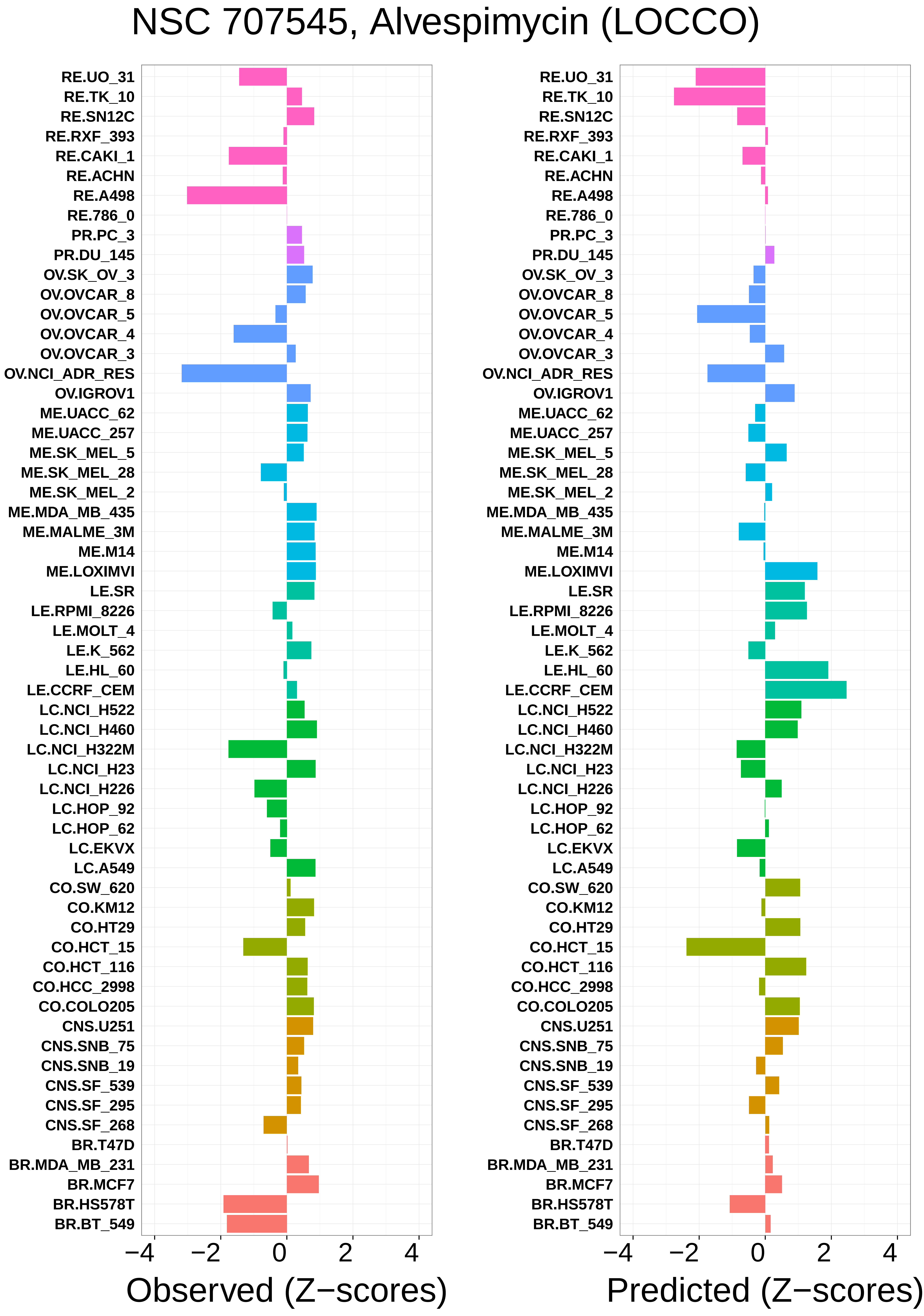

NSC 707545, Alvespimycin (LOCCO)
RE.UO_31
RE.TK_10
RE.SN12C
RE.RXF_393
RE.CAKI_1
RE.ACHN
RE.A498
RE.786_0
PR.PC_3
PR.DU_145
OV.SK_OV_3
OV.OVCAR_8
OV.OVCAR_5
OV.OVCAR_4
OV.OVCAR_3
OV.NCI_ADR_RES
OV.IGROV1
ME.UACC_62
ME.UACC_257
ME.SK_MEL_5
ME.SK_MEL_28
ME.SK_MEL_2
ME.MDA_MB_435
ME.MALME_3M
ME.M14
ME.LOXIMVI
LE.SR
LE.RPMI_8226
LE.MOLT_4
LE.K_562
LE.HL_60
LE.CCRF_CEM
LC.NCI_H522
LC.NCI_H460
LC.NCI_H322M
LC.NCI_H23
LC.NCI_H226
LC.HOP_92
LC.HOP_62
LC.EKVX
LC.A549
CO.SW_620
CO.KM12
CO.HT29
CO.HCT_15
CO.HCT_116
CO.HCC_2998
CO.COLO205
CNS.U251
CNS.SNB_75
CNS.SNB_19
CNS.SF_539
CNS.SF_295
CNS.SF_268
BR.T47D
BR.MDA_MB_231
BR.MCF7
BR.HS578T
BR.BT_549
−4 −2 0 2 4
Observed (Z−scores)
Predicted (Z−scores)

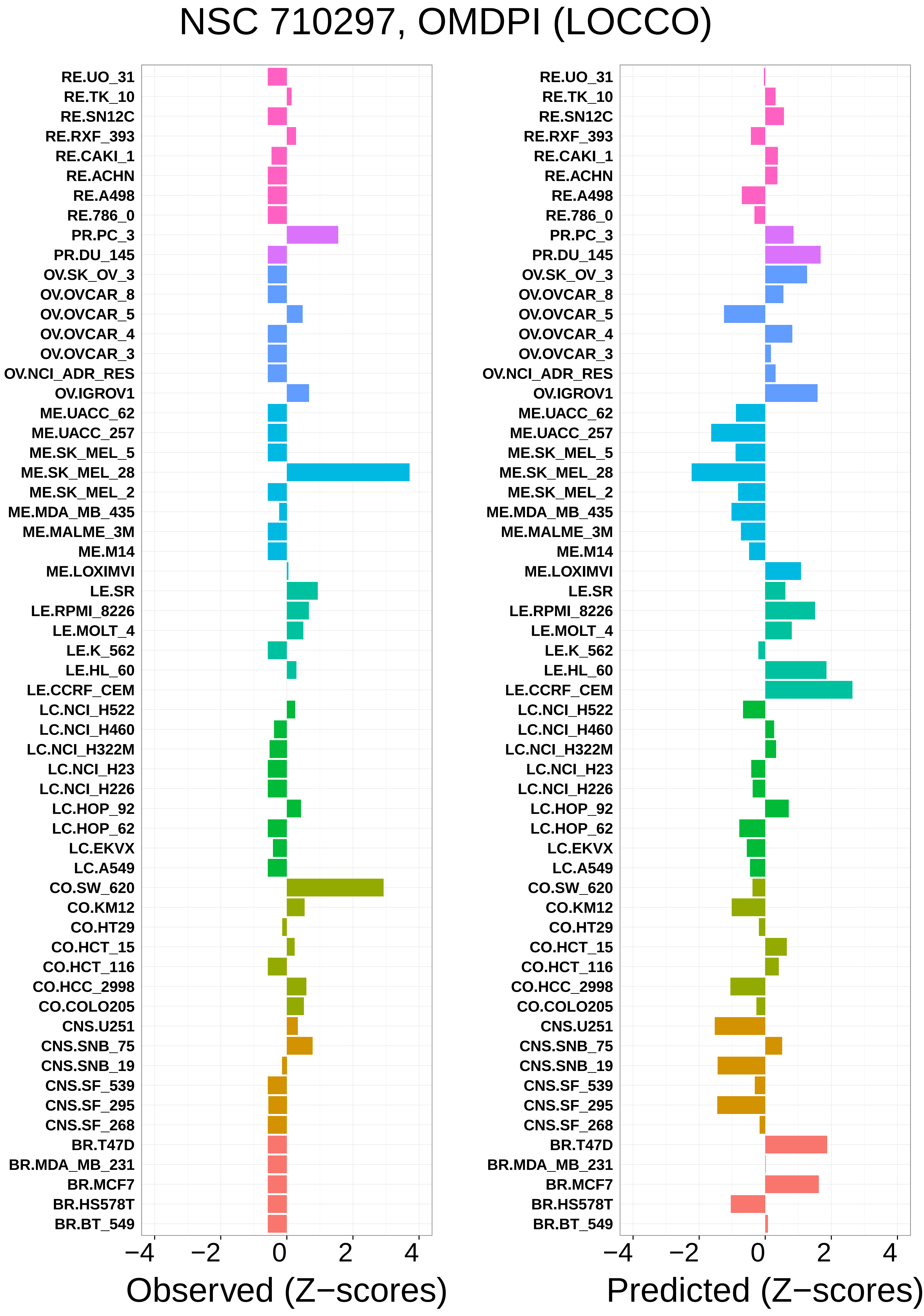

NSC 710297, OMDPI (LOCCO)
RE.UO_31
RE.TK_10
RE.SN12C
RE.RXF_393
RE.CAKI_1
RE.ACHN
RE.A498
RE.786_0
PR.PC_3
PR.DU_145
OV.SK_OV_3
OV.OVCAR_8
OV.OVCAR_5
OV.OVCAR_4
OV.OVCAR_3
OV.NCI_ADR_RES
OV.IGROV1
ME.UACC_62
ME.UACC_257
ME.SK_MEL_5
ME.SK_MEL_28
ME.SK_MEL_2
ME.MDA_MB_435
ME.MALME_3M
ME.M14
ME.LOXIMVI
LE.SR
LE.RPMI_8226
LE.MOLT_4
LE.K_562
LE.HL_60
LE.CCRF_CEM
LC.NCI_H522
LC.NCI_H460
LC.NCI_H322M
LC.NCI_H23
LC.NCI_H226
LC.HOP_92
LC.HOP_62
LC.EKVX
LC.A549
CO.SW_620
CO.KM12
CO.HT29
CO.HCT_15
CO.HCT_116
CO.HCC_2998
CO.COLO205
CNS.U251
CNS.SNB_75
CNS.SNB_19
CNS.SF_539
CNS.SF_295
CNS.SF_268
BR.T47D
BR.MDA_MB_231
BR.MCF7
BR.HS578T
BR.BT_549
−4 −2 0 2 4
Observed (Z−scores)
Predicted (Z−scores)

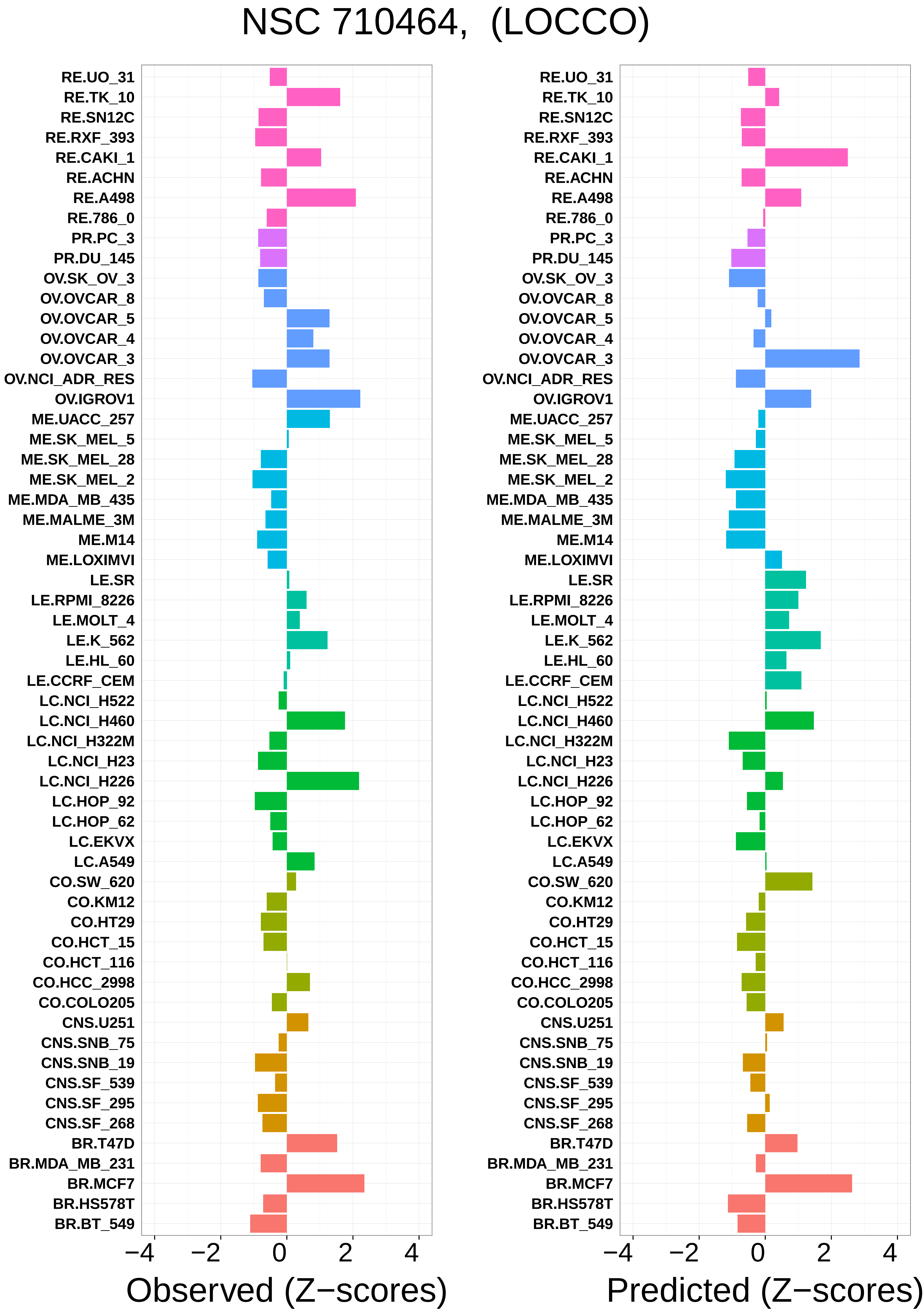
NSC 710464, (LOCCO)
RE.UO_31
RE.TK_10
RE.SN12C
RE.RXF_393
RE.CAKI_1
RE.ACHN
RE.A498
RE.786_0
PR.PC_3
PR.DU_145
OV.SK_OV_3
OV.OVCAR_8
OV.OVCAR_5
OV.OVCAR_4
OV.OVCAR_3
OV.NCI_ADR_RES
OV.IGROV1
ME.UACC_257
ME.SK_MEL_5
ME.SK_MEL_28
ME.SK_MEL_2
ME.MDA_MB_435
ME.MALME_3M
ME.M14
ME.LOXIMVI
LE.SR
LE.RPMI_8226
LE.MOLT_4
LE.K_562
LE.HL_60
LE.CCRF_CEM
LC.NCI_H522
LC.NCI_H460
LC.NCI_H322M
LC.NCI_H23
LC.NCI_H226
LC.HOP_92
LC.HOP_62
LC.EKVX
LC.A549
CO.SW_620
CO.KM12
CO.HT29
CO.HCT_15
CO.HCT_116
CO.HCC_2998
CO.COLO205
CNS.U251
CNS.SNB_75
CNS.SNB_19
CNS.SF_539
CNS.SF_295
CNS.SF_268
BR.T47D
BR.MDA_MB_231
BR.MCF7
BR.HS578T
BR.BT_549
−4 −2 0 2 4
Observed (Z−scores)
Predicted (Z−scores)

NSC 715055, Gefitinib (LOCCO)

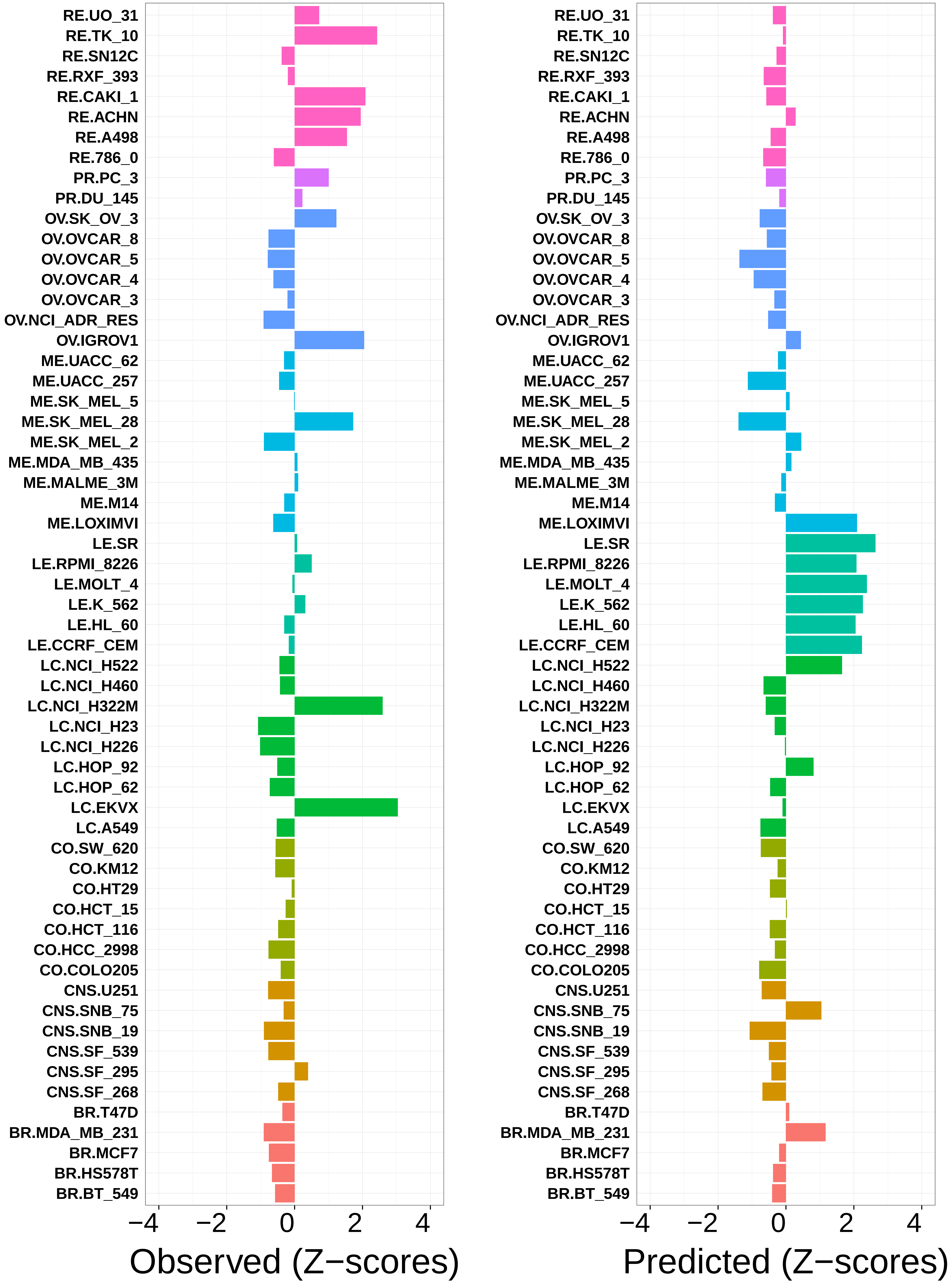

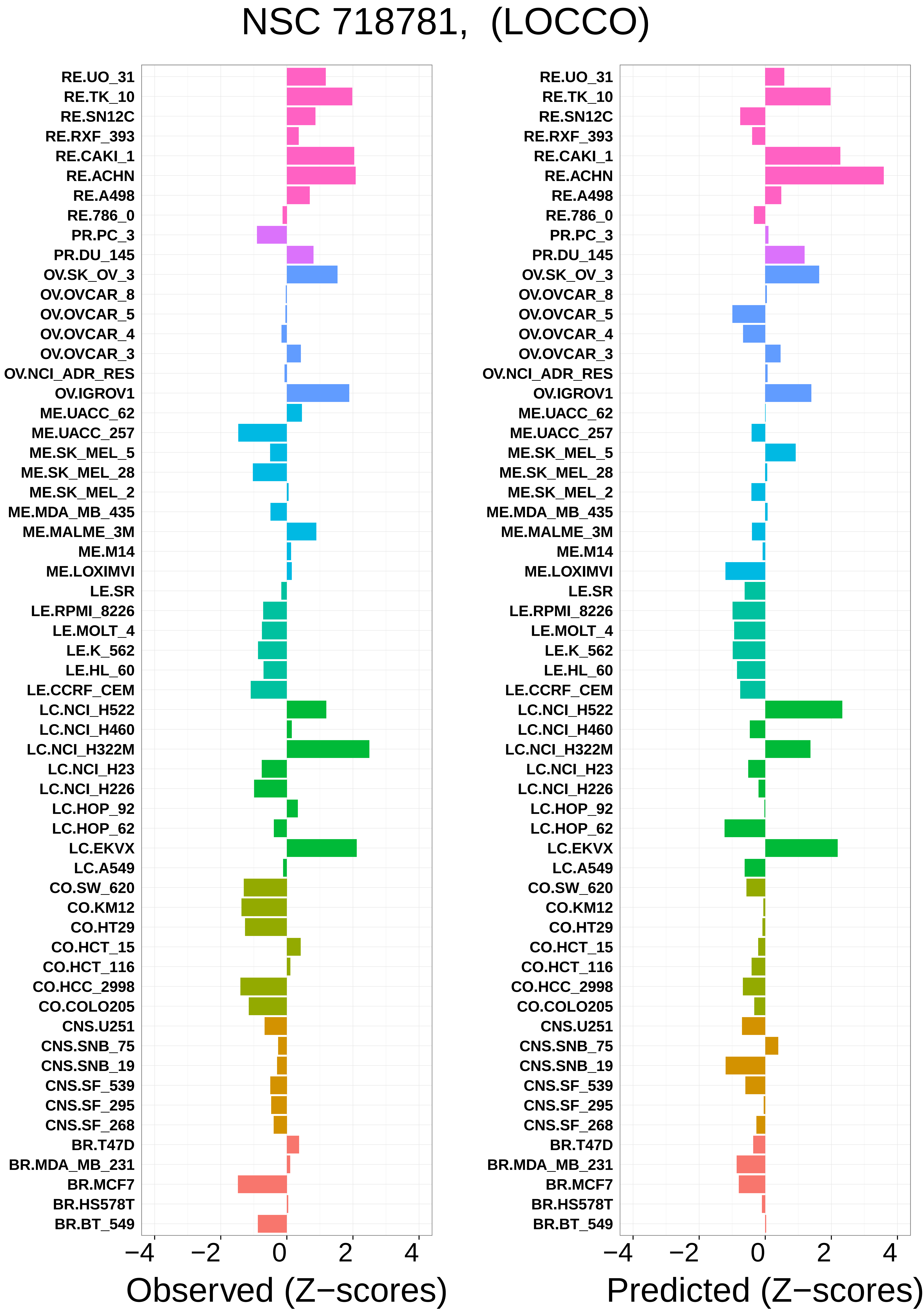
NSC 718781, (LOCCO)
RE.UO_31
RE.TK_10
RE.SN12C
RE.RXF_393
RE.CAKI_1
RE.ACHN
RE.A498
RE.786_0
PR.PC_3
PR.DU_145
OV.SK_OV_3
OV.OVCAR_8
OV.OVCAR_5
OV.OVCAR_4
OV.OVCAR_3
OV.NCI_ADR_RES
OV.IGROV1
ME.UACC_62
ME.UACC_257
ME.SK_MEL_5
ME.SK_MEL_28
ME.SK_MEL_2
ME.MDA_MB_435
ME.MALME_3M
ME.M14
ME.LOXIMVI
LE.SR
LE.RPMI_8226
LE.MOLT_4
LE.K_562
LE.HL_60
LE.CCRF_CEM
LC.NCI_H522
LC.NCI_H460
LC.NCI_H322M
LC.NCI_H23
LC.NCI_H226
LC.HOP_92
LC.HOP_62
LC.EKVX
LC.A549
CO.SW_620
CO.KM12
CO.HT29
CO.HCT_15
CO.HCT_116
CO.HCC_2998
CO.COLO205
CNS.U251
CNS.SNB_75
CNS.SNB_19
CNS.SF_539
CNS.SF_295
CNS.SF_268
BR.T47D
BR.MDA_MB_231
BR.MCF7
BR.HS578T
BR.BT_549
−4 −2 0 2 4
Observed (Z−scores)
Predicted (Z−scores)

NSC 719239, (LOCCO)

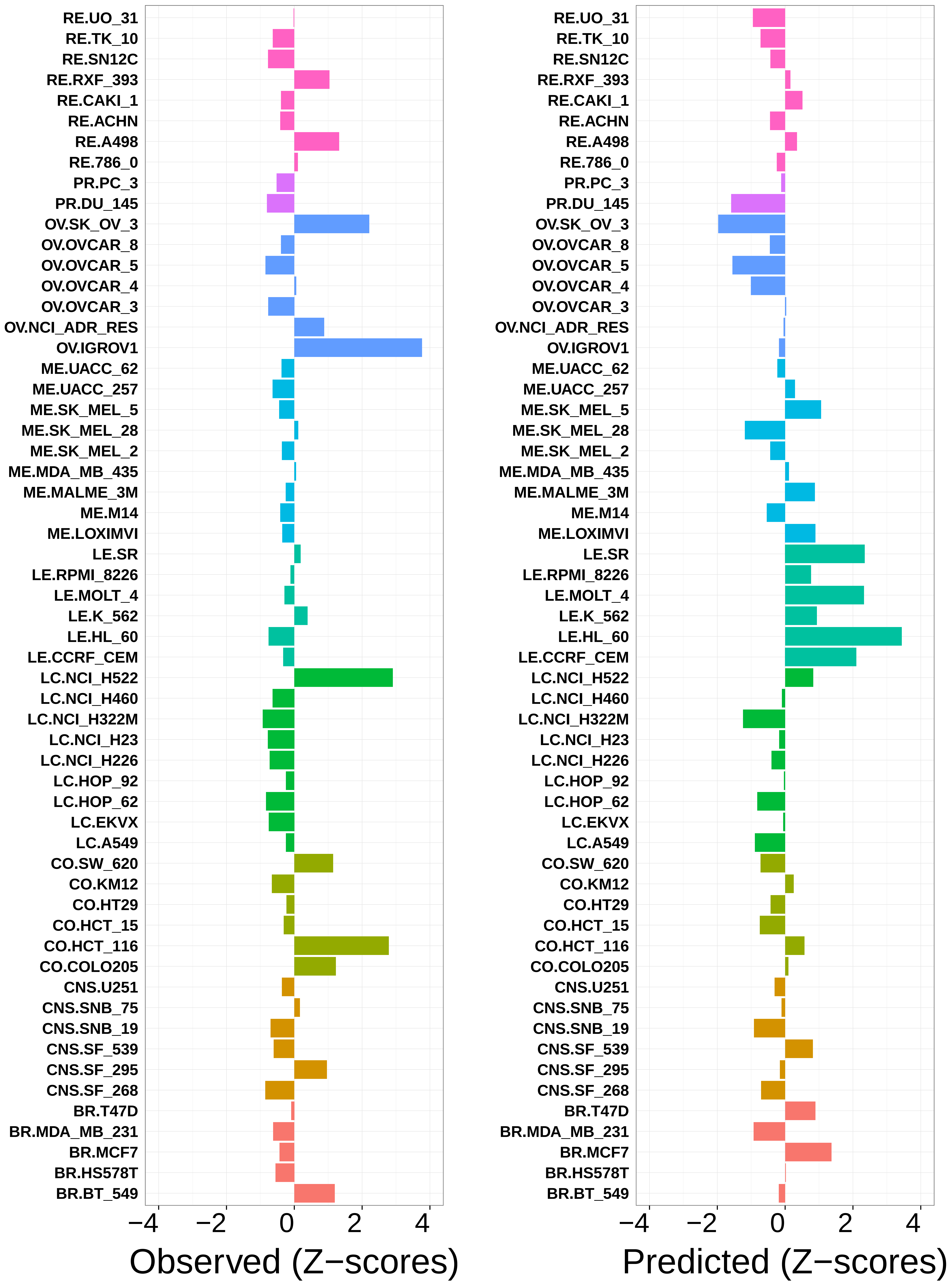

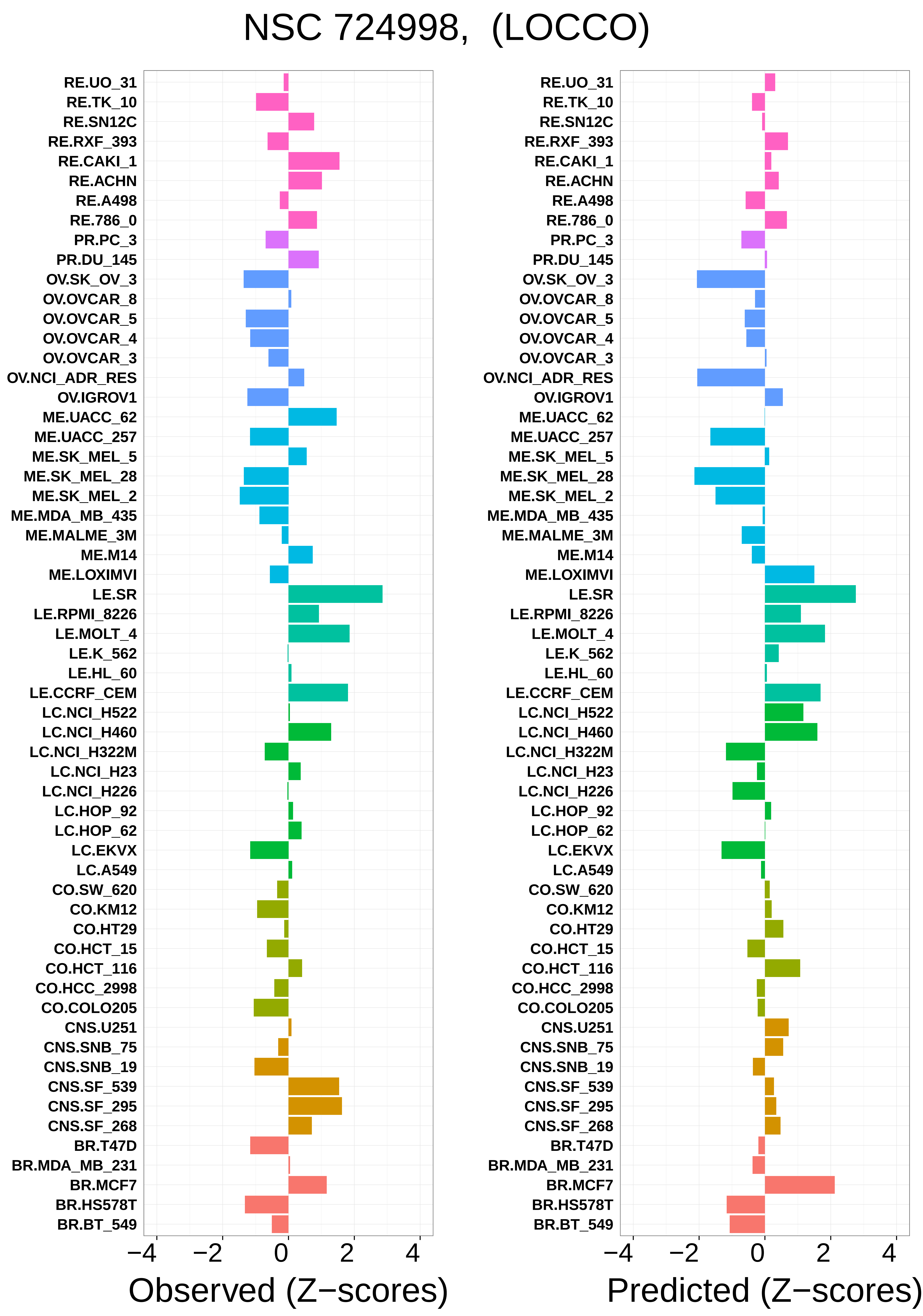
NSC 724998, (LOCCO)
RE.UO_31
RE.TK_10
RE.SN12C
RE.RXF_393
RE.CAKI_1
RE.ACHN
RE.A498
RE.786_0
PR.PC_3
PR.DU_145
OV.SK_OV_3
OV.OVCAR_8
OV.OVCAR_5
OV.OVCAR_4
OV.OVCAR_3
OV.NCI_ADR_RES
OV.IGROV1
ME.UACC_62
ME.UACC_257
ME.SK_MEL_5
ME.SK_MEL_28
ME.SK_MEL_2
ME.MDA_MB_435
ME.MALME_3M
ME.M14
ME.LOXIMVI
LE.SR
LE.RPMI_8226
LE.MOLT_4
LE.K_562
LE.HL_60
LE.CCRF_CEM
LC.NCI_H522
LC.NCI_H460
LC.NCI_H322M
LC.NCI_H23
LC.NCI_H226
LC.HOP_92
LC.HOP_62
LC.EKVX
LC.A549
CO.SW_620
CO.KM12
CO.HT29
CO.HCT_15
CO.HCT_116
CO.HCC_2998
CO.COLO205
CNS.U251
CNS.SNB_75
CNS.SNB_19
CNS.SF_539
CNS.SF_295
CNS.SF_268
BR.T47D
BR.MDA_MB_231
BR.MCF7
BR.HS578T
BR.BT_549
−4
−2
0
2
4
Observed (Z−scores)
Predicted (Z−scores)

NSC 725776, (LOCCO)

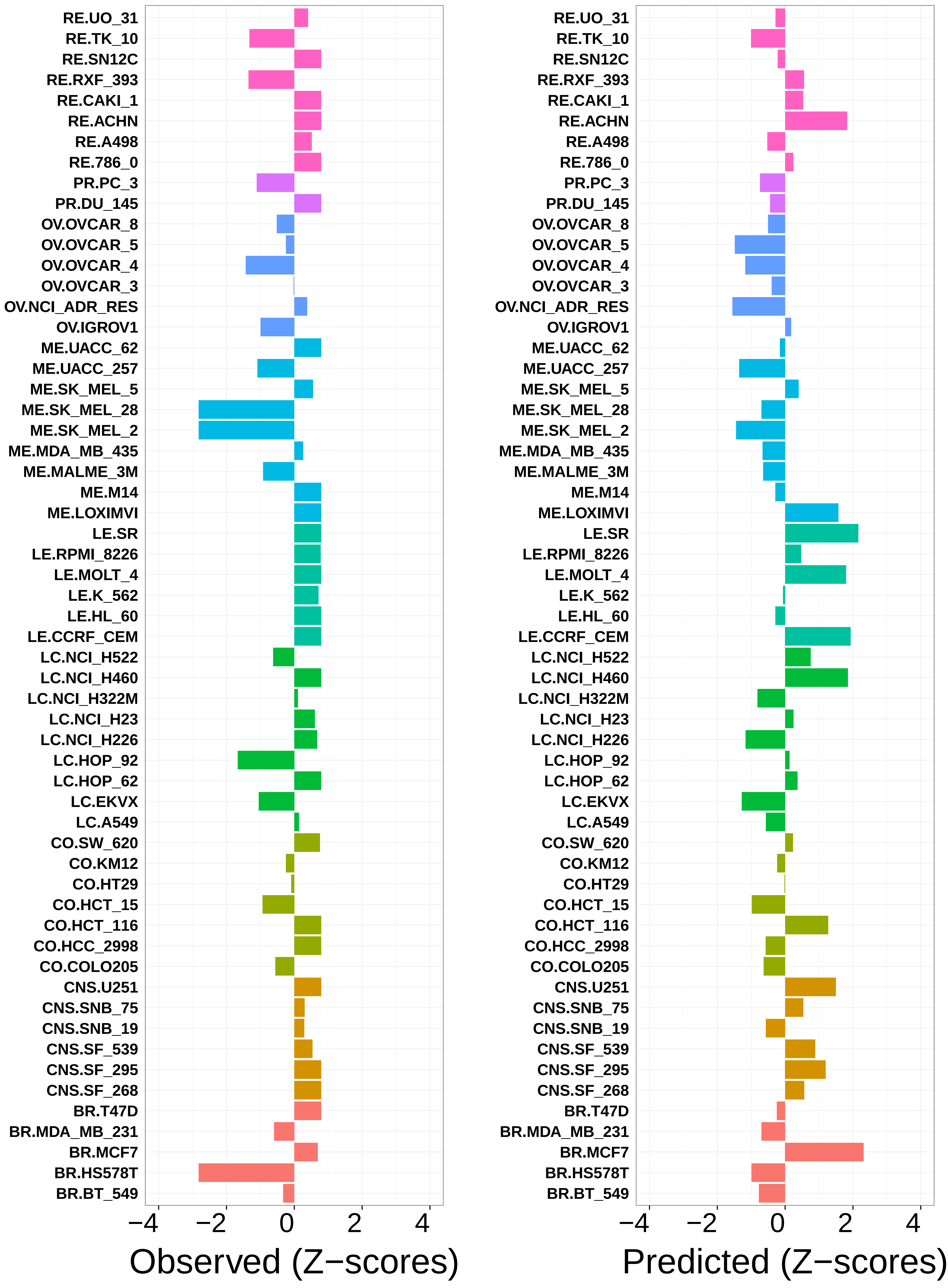

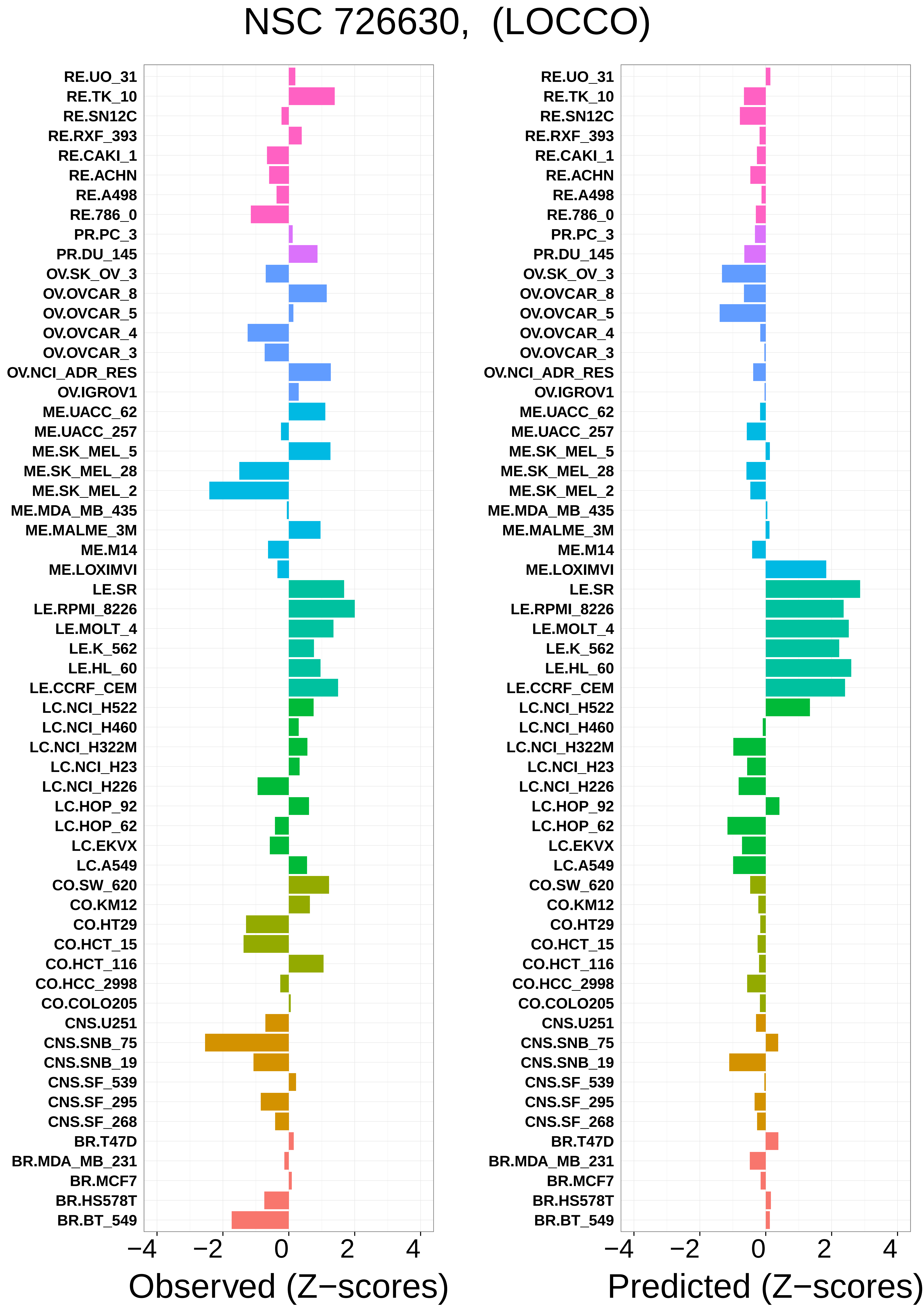

NSC 726630, (LOCCO)
RE.UO_31
RE.TK_10
RE.SN12C
RE.RXF_393
RE.CAKI_1
RE.ACHN
RE.A498
RE.786_0
PR.PC_3
PR.DU_145
OV.SK_OV_3
OV.OVCAR_8
OV.OVCAR_5
OV.OVCAR_4
OV.OVCAR_3
OV.NCI_ADR_RES
OV.IGROV1
ME.UACC_62
ME.UACC_257
ME.SK_MEL_5
ME.SK_MEL_28
ME.SK_MEL_2
ME.MDA_MB_435
ME.MALME_3M
ME.M14
ME.LOXIMVI
LE.SR
LE.RPMI_8226
LE.MOLT_4
LE.K_562
LE.HL_60
LE.CCRF_CEM
LC.NCI_H522
LC.NCI_H460
LC.NCI_H322M
LC.NCI_H23
LC.NCI_H226
LC.HOP_92
LC.HOP_62
LC.EKVX
LC.A549
CO.SW_620
CO.KM12
CO.HT29
CO.HCT_15
CO.HCT_116
CO.HCC_2998
CO.COLO205
CNS.U251
CNS.SNB_75
CNS.SNB_19
CNS.SF_539
CNS.SF_295
CNS.SF_268
BR.T47D
BR.MDA_MB_231
BR.MCF7
BR.HS578T
BR.BT_549
−4 −2 0 2 4
Observed (Z−scores)
Predicted (Z−scores)

NSC 728073, (LOCCO)

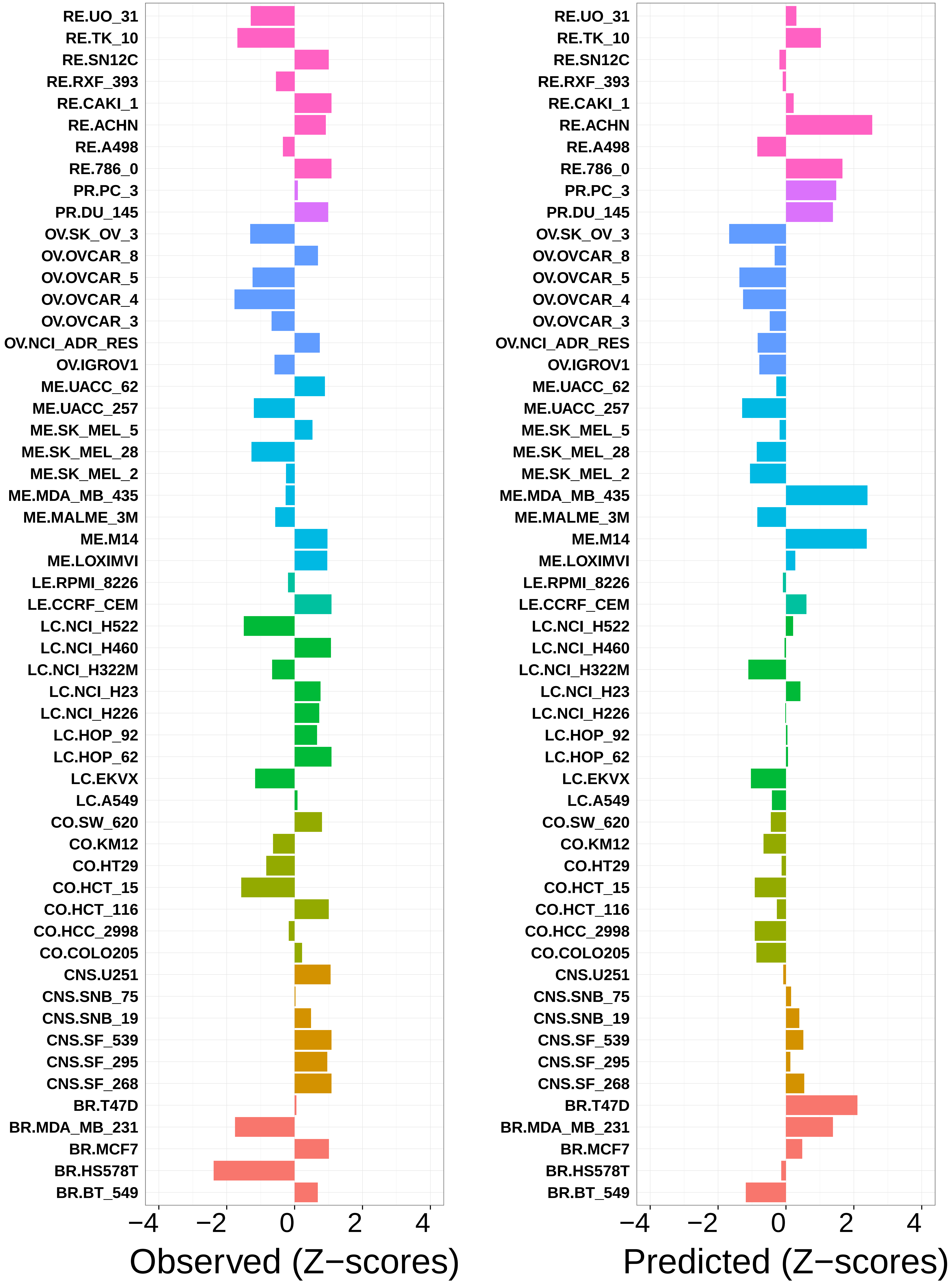

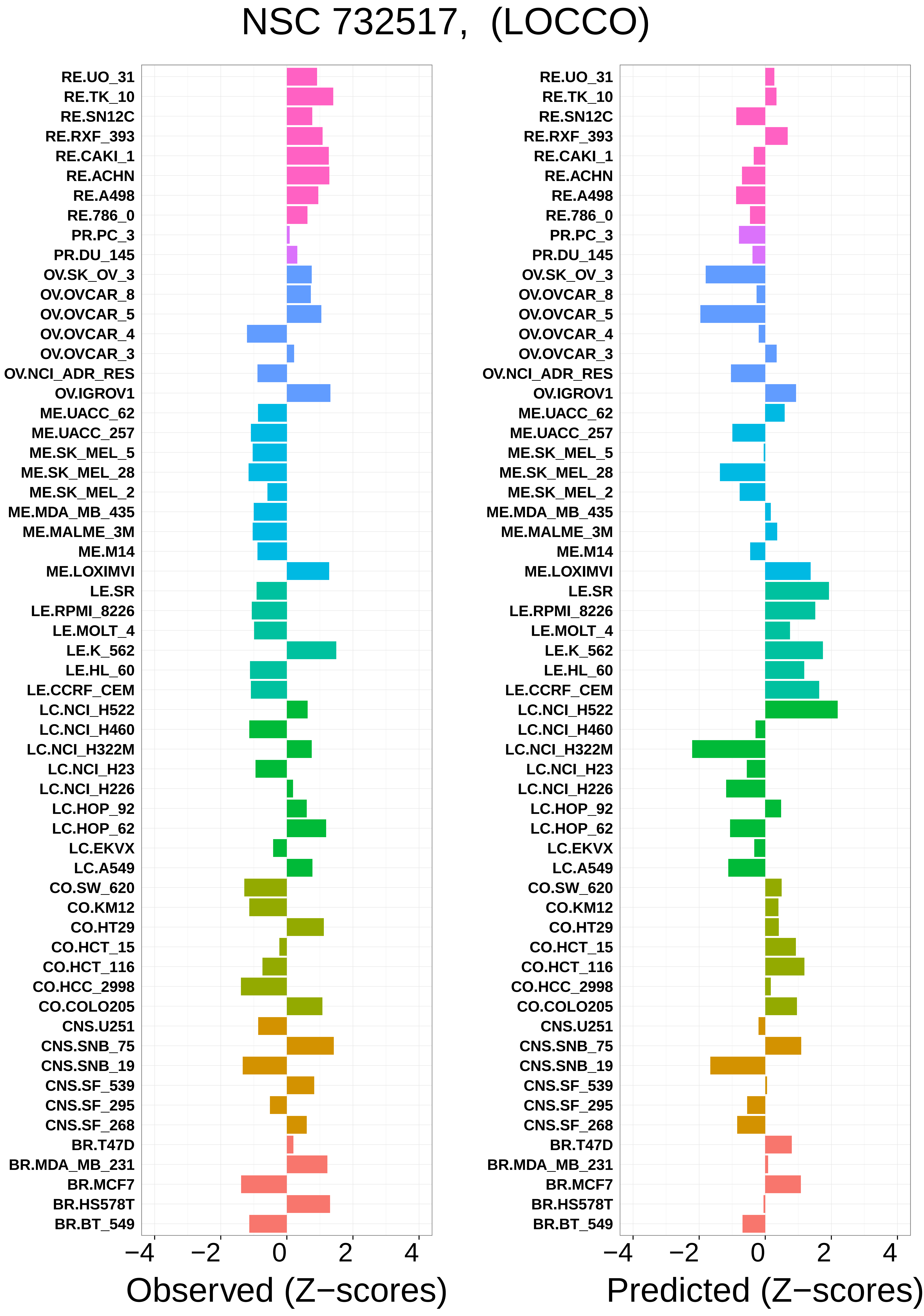
NSC 732517, (LOCCO)
RE.UO_31
RE.TK_10
RE.SN12C
RE.RXF_393
RE.CAKI_1
RE.ACHN
RE.A498
RE.786_0
PR.PC_3
PR.DU_145
OV.SK_OV_3
OV.OVCAR_8
OV.OVCAR_5
OV.OVCAR_4
OV.OVCAR_3
OV.NCI_ADR_RES
OV.IGROV1
ME.UACC_62
ME.UACC_257
ME.SK_MEL_5
ME.SK_MEL_28
ME.SK_MEL_2
ME.MDA_MB_435
ME.MALME_3M
ME.M14
ME.LOXIMVI
LE.SR
LE.RPMI_8226
LE.MOLT_4
LE.K_562
LE.HL_60
LE.CCRF_CEM
LC.NCI_H522
LC.NCI_H460
LC.NCI_H322M
LC.NCI_H23
LC.NCI_H226
LC.HOP_92
LC.HOP_62
LC.EKVX
LC.A549
CO.SW_620
CO.KM12
CO.HT29
CO.HCT_15
CO.HCT_116
CO.HCC_2998
CO.COLO205
CNS.U251
CNS.SNB_75
CNS.SNB_19
CNS.SF_539
CNS.SF_295
CNS.SF_268
BR.T47D
BR.MDA_MB_231
BR.MCF7
BR.HS578T
BR.BT_549
−4
−2
0
2
4
Observed (Z−scores)
Predicted (Z−scores)

NSC 740, Methotrexate (LOCCO)

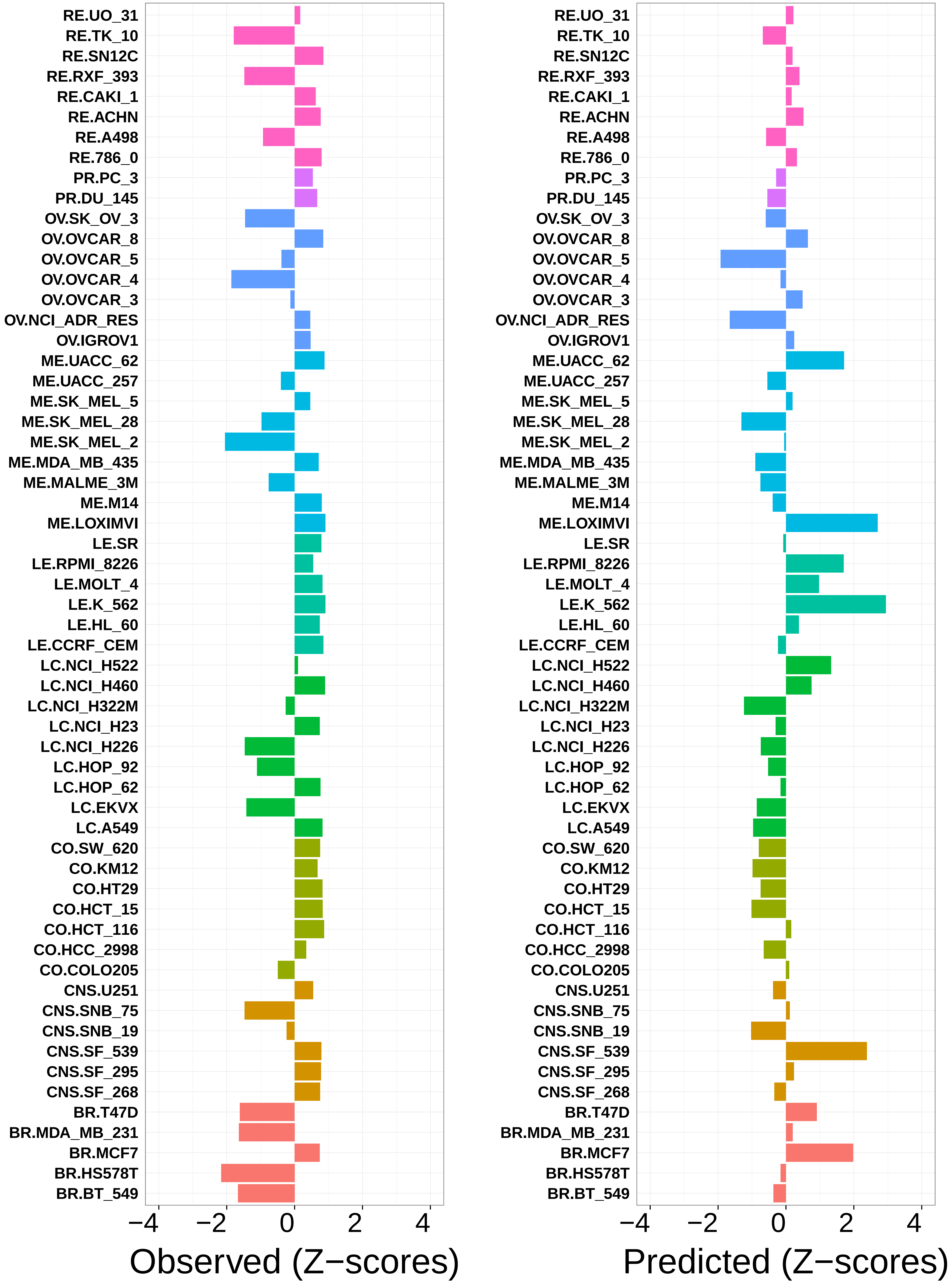

NSC 741078, selumetinib (LOCCO)

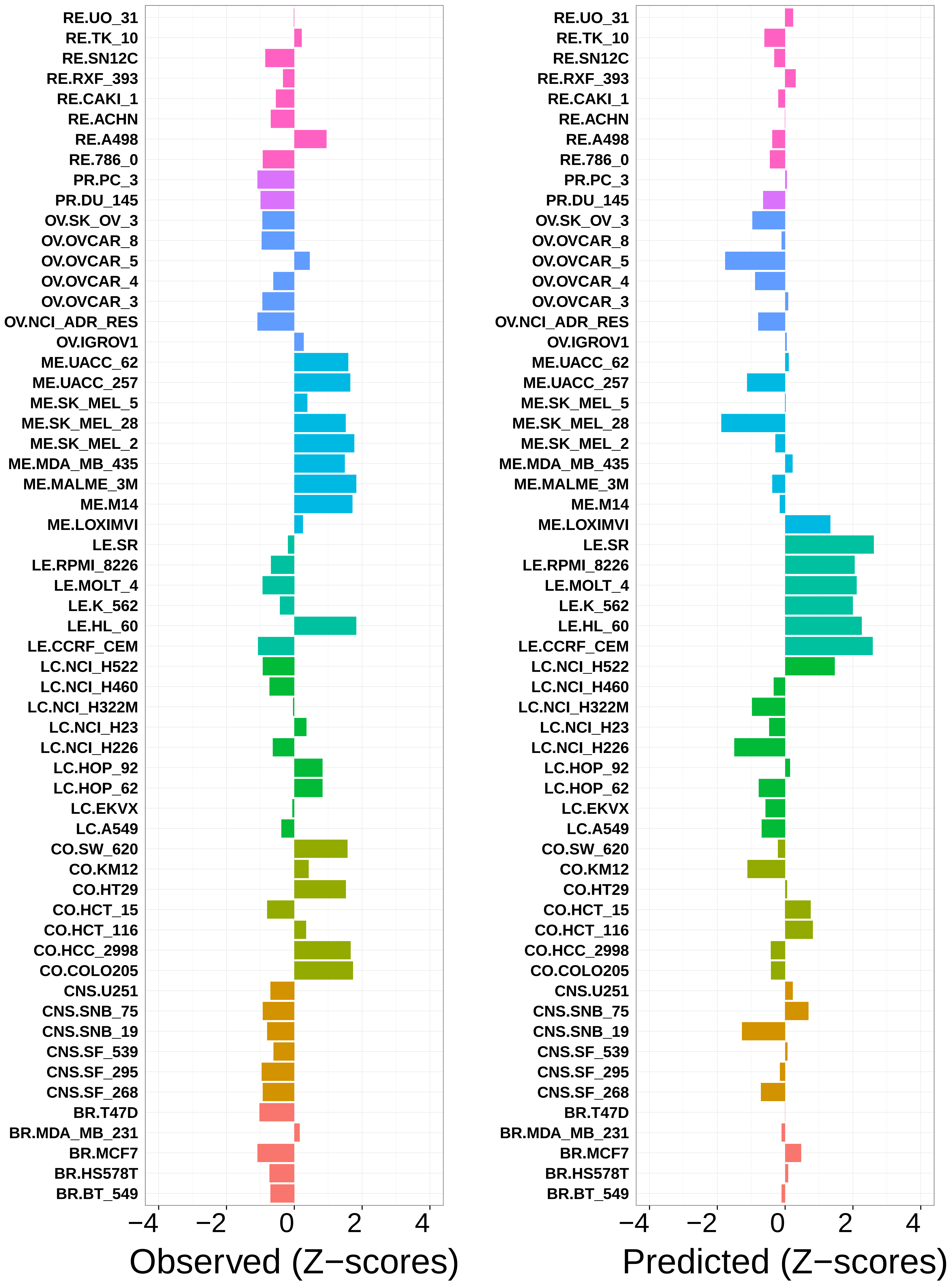

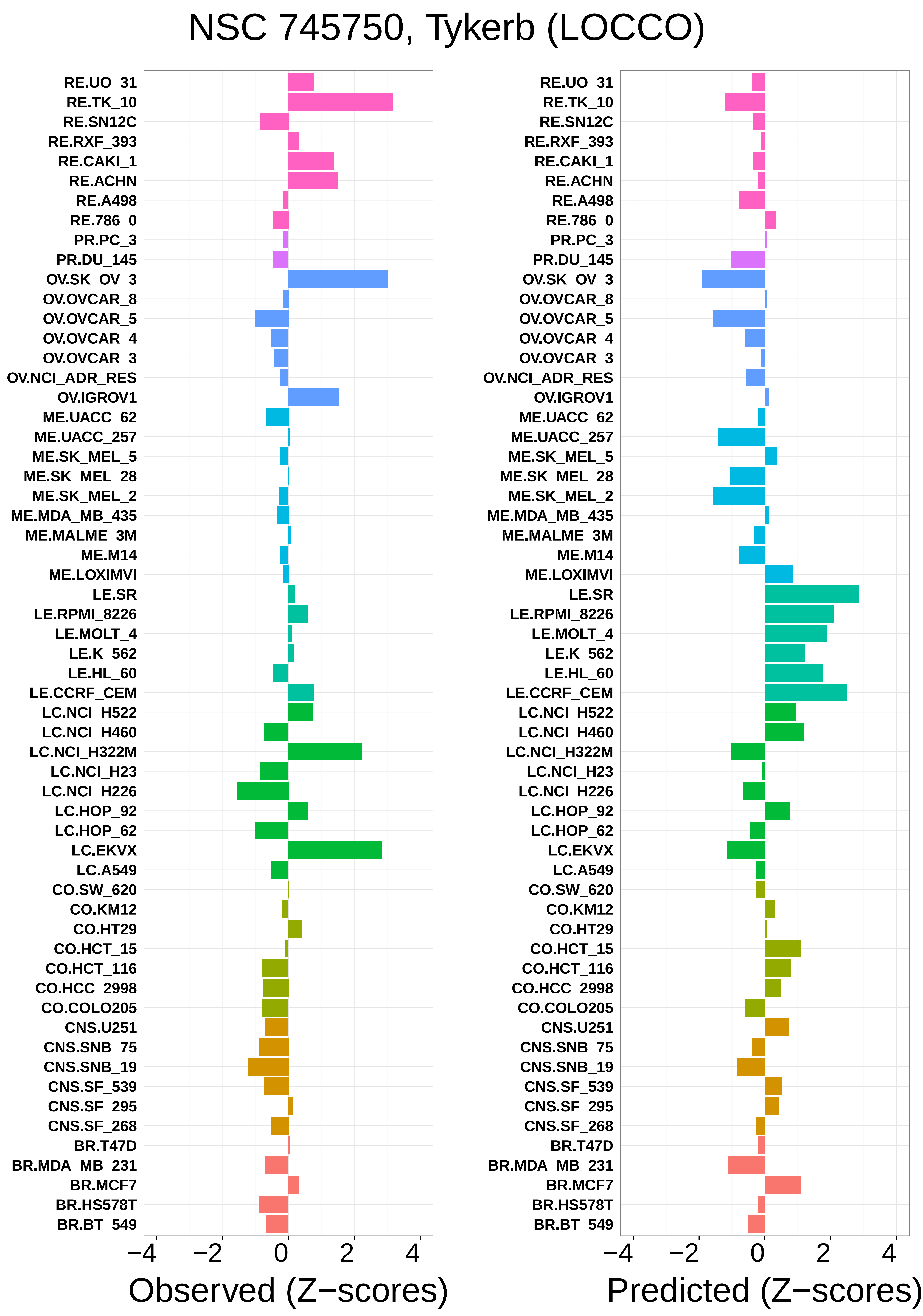
NSC 745750, Tykerb (LOCCO)
RE.UO_31
RE.TK_10
RE.SN12C
RE.RXF_393
RE.CAKI_1
RE.ACHN
RE.A498
RE.786_0
PR.PC_3
PR.DU_145
OV.SK_OV_3
OV.OVCAR_8
OV.OVCAR_5
OV.OVCAR_4
OV.OVCAR_3
OV.NCI_ADR_RES
OV.IGROV1
ME.UACC_62
ME.UACC_257
ME.SK_MEL_5
ME.SK_MEL_28
ME.SK_MEL_2
ME.MDA_MB_435
ME.MALME_3M
ME.M14
ME.LOXIMVI
LE.SR
LE.RPMI_8226
LE.MOLT_4
LE.K_562
LE.HL_60
LE.CCRF_CEM
LC.NCI_H522
LC.NCI_H460
LC.NCI_H322M
LC.NCI_H23
LC.NCI_H226
LC.HOP_92
LC.HOP_62
LC.EKVX
LC.A549
CO.SW_620
CO.KM12
CO.HT29
CO.HCT_15
CO.HCT_116
CO.HCC_2998
CO.COLO205
CNS.U251
CNS.SNB_75
CNS.SNB_19
CNS.SF_539
CNS.SF_295
CNS.SF_268
BR.T47D
BR.MDA_MB_231
BR.MCF7
BR.HS578T
BR.BT_549
−4
−2
0
2
4
Observed (Z−scores)
Predicted (Z−scores)

NSC 749226, (LOCCO)

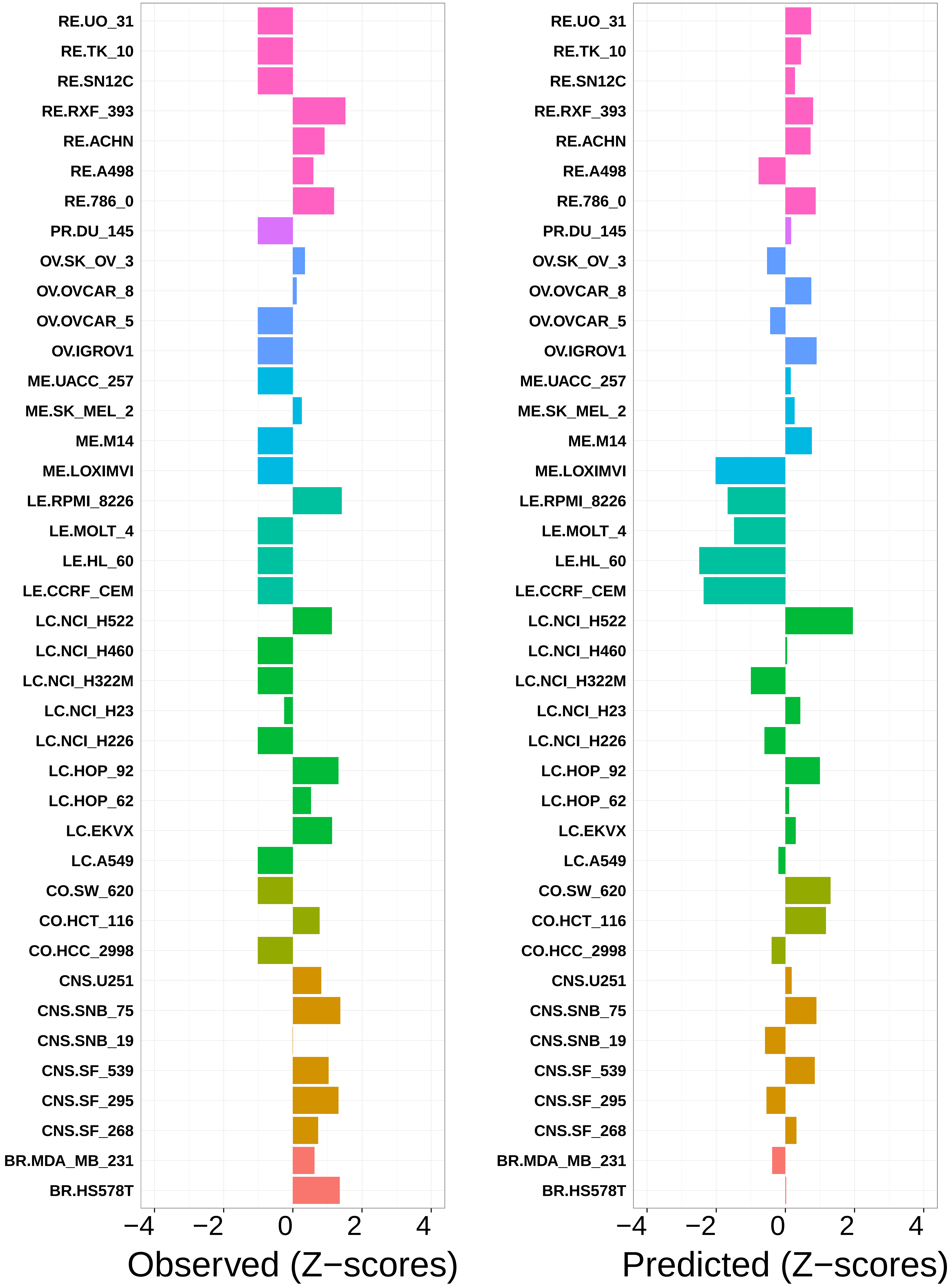

NSC 755, Mercaptopurine (LOCCO)

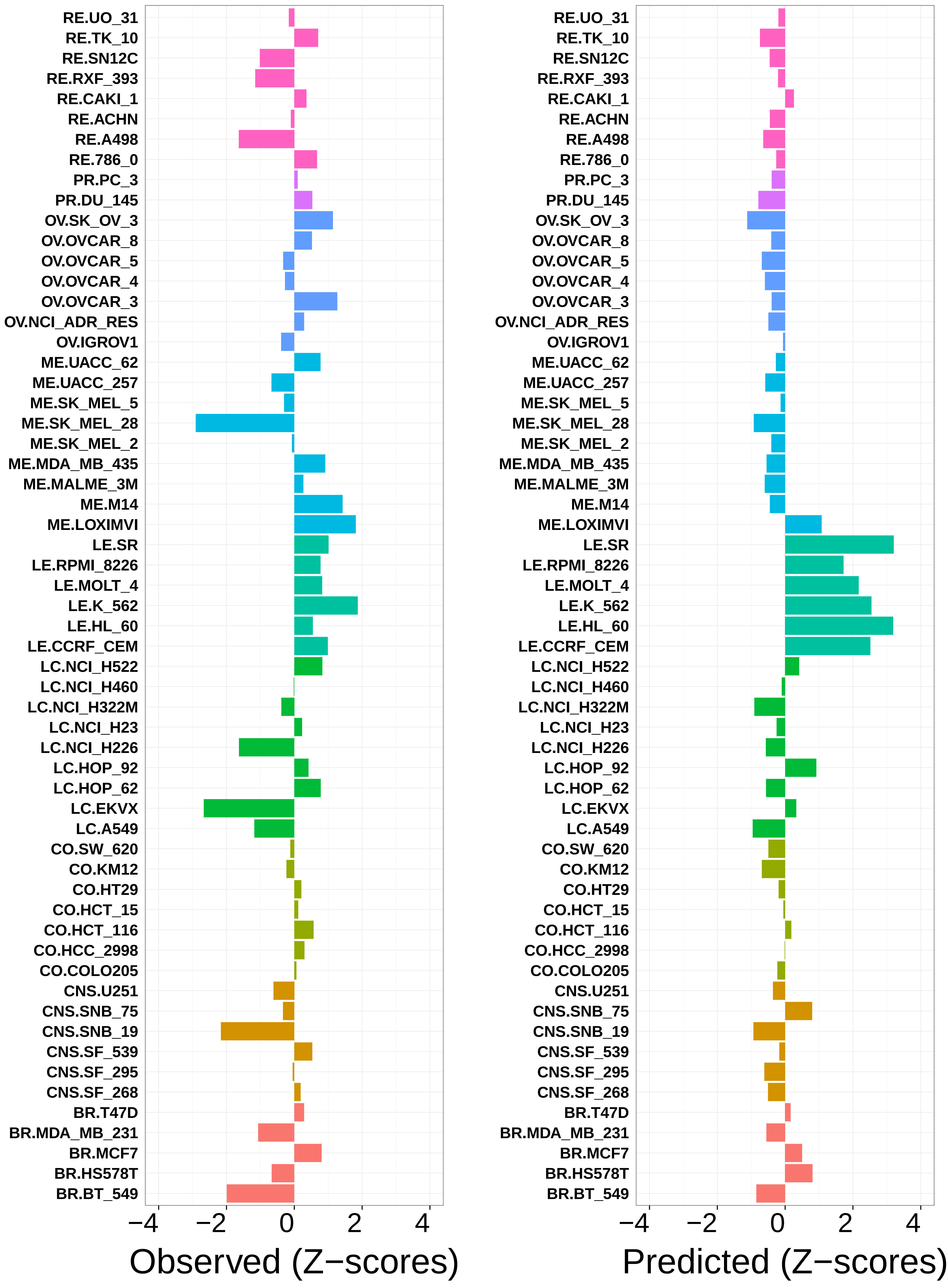

NSC 762, Mechlorethamine_Hydrochloride (LOCCO)

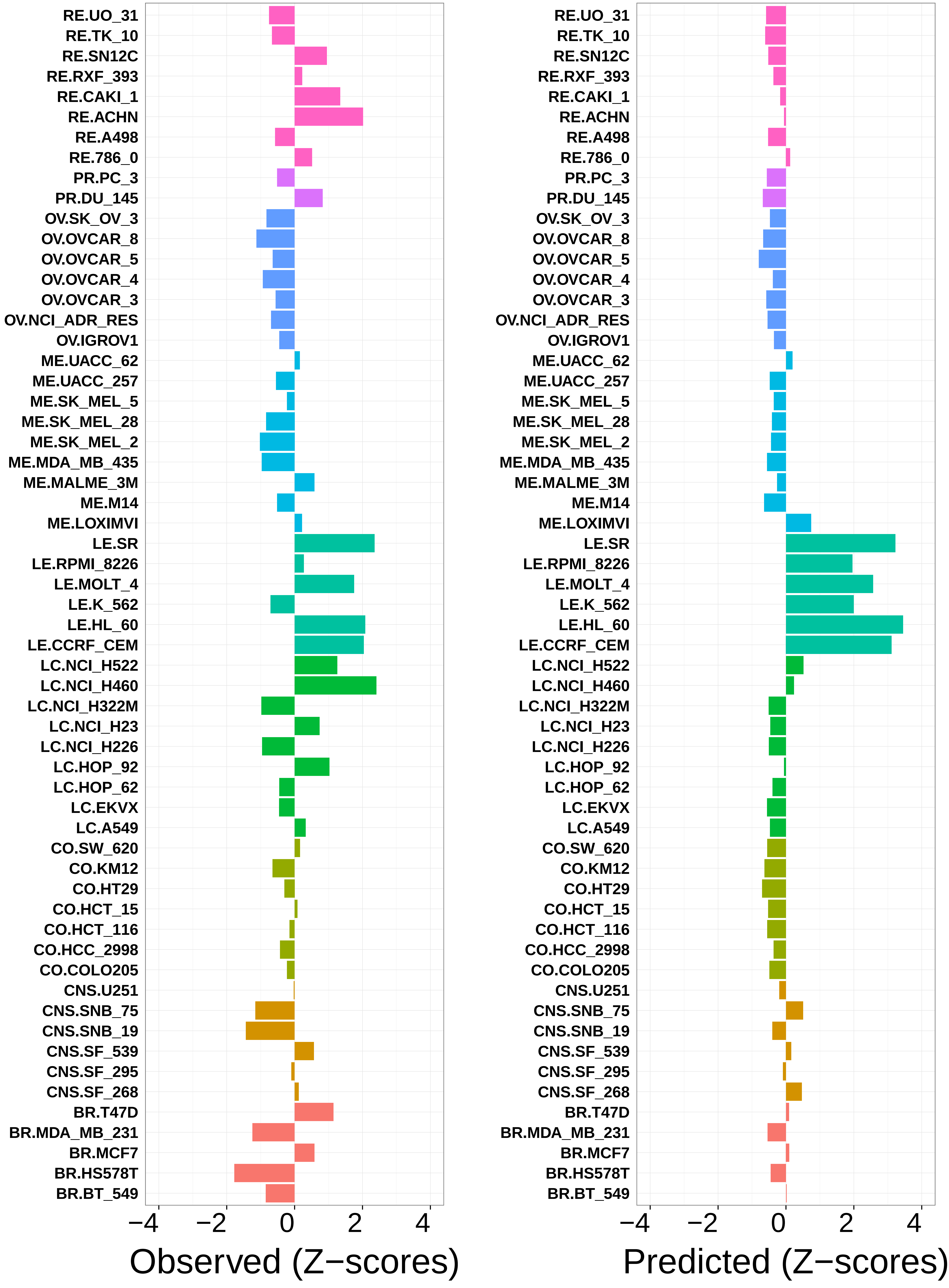

NSC 105014, Cladribine (LOCO)

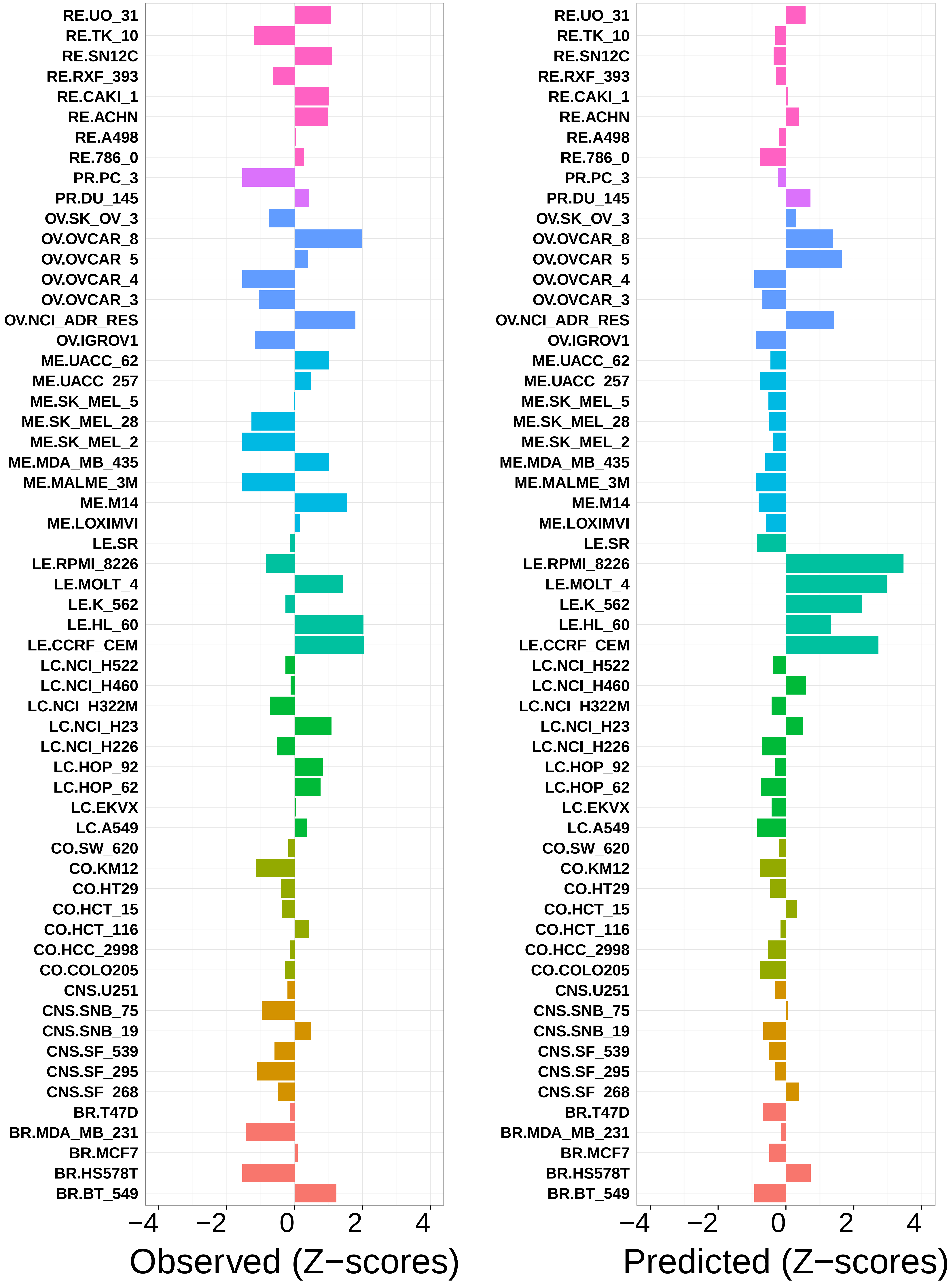

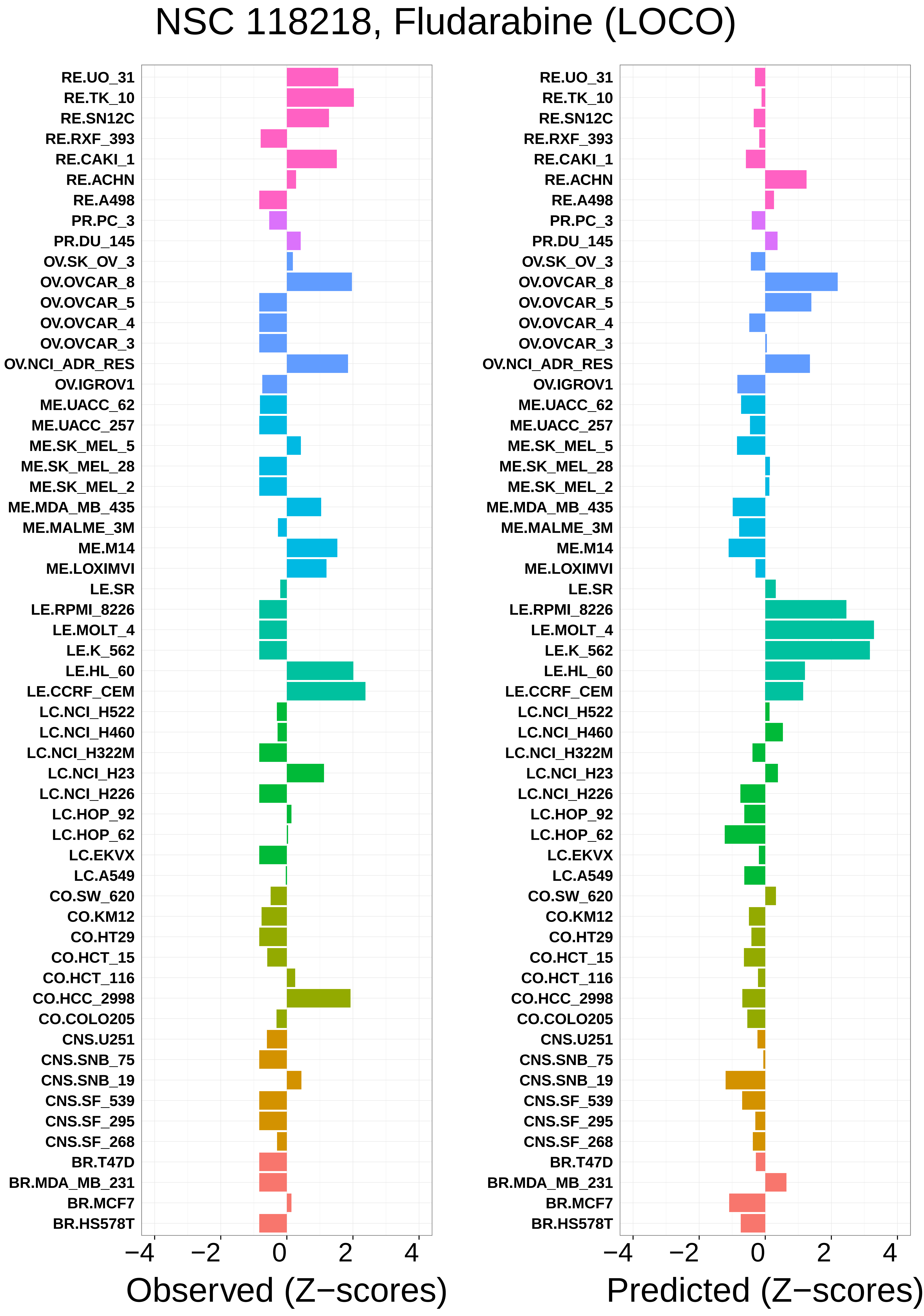
NSC 118218, Fludarabine (LOCO)
RE.UO_31
RE.TK_10
RE.SN12C
RE.RXF_393
RE.CAKI_1
RE.ACHN
RE.A498
PR.PC_3
PR.DU_145
OV.SK_OV_3
OV.OVCAR_8
OV.OVCAR_5
OV.OVCAR_4
OV.OVCAR_3
OV.NCI_ADR_RES
OV.IGROV1
ME.UACC_62
ME.UACC_257
ME.SK_MEL_5
ME.SK_MEL_28
ME.SK_MEL_2
ME.MDA_MB_435
ME.MALME_3M
ME.M14
ME.LOXIMVI
LE.SR
LE.RPMI_8226
LE.MOLT_4
LE.K_562
LE.HL_60
LE.CCRF_CEM
LC.NCI_H522
LC.NCI_H460
LC.NCI_H322M
LC.NCI_H23
LC.NCI_H226
LC.HOP_92
LC.HOP_62
LC.EKVX
LC.A549
CO.SW_620
CO.KM12
CO.HT29
CO.HCT_15
CO.HCT_116
CO.HCC_2998
CO.COLO205
CNS.U251
CNS.SNB_75
CNS.SNB_19
CNS.SF_539
CNS.SF_295
CNS.SF_268
BR.T47D
BR.MDA_MB_231
BR.MCF7
BR.HS578T
−4
−2
0
2
4
Observed (Z−scores)
Predicted (Z−scores)

NSC 122819, Teniposide (LOCO)

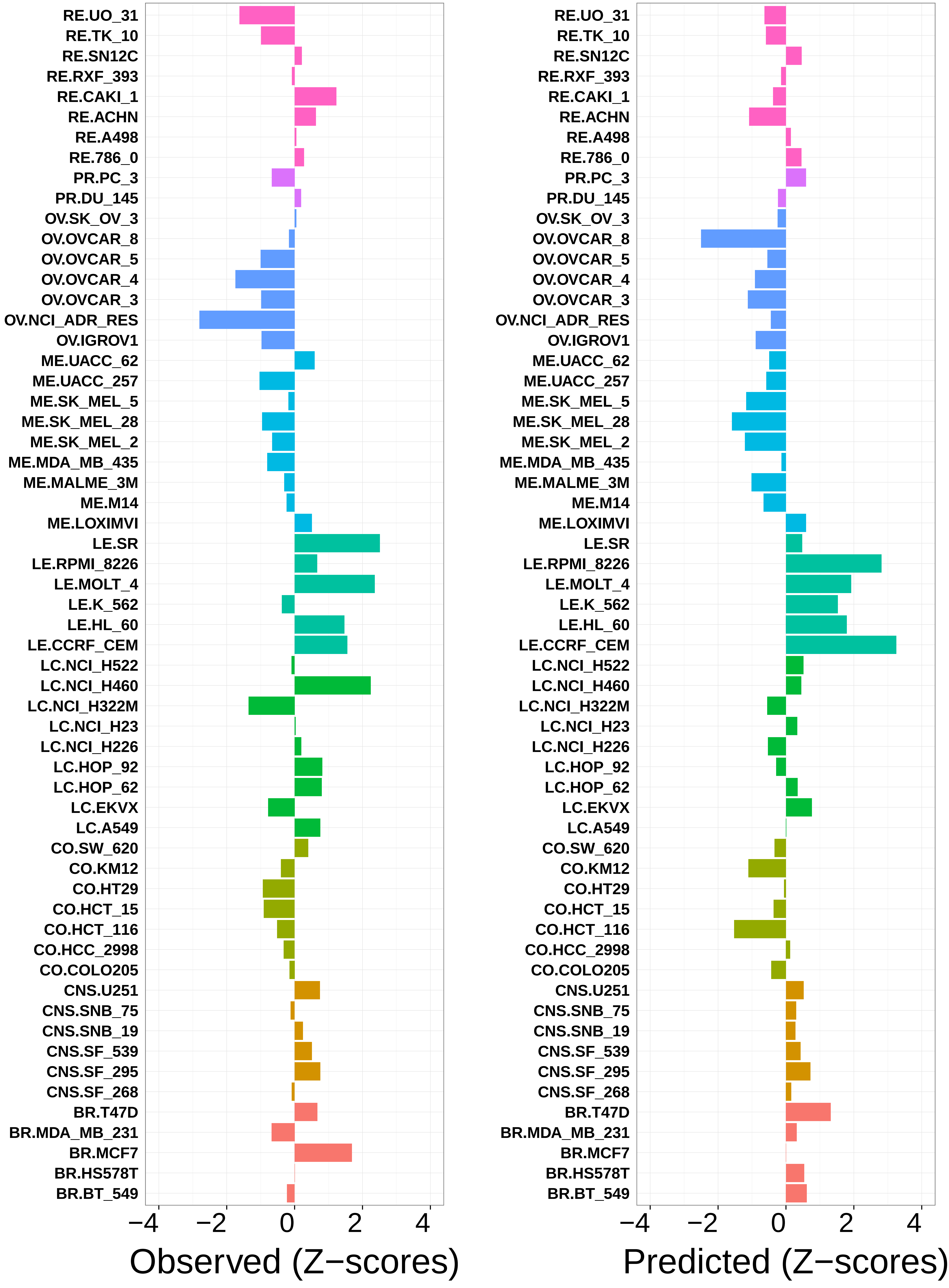

NSC 123127, Doxorubicin_Hydrochloride (LOCO)

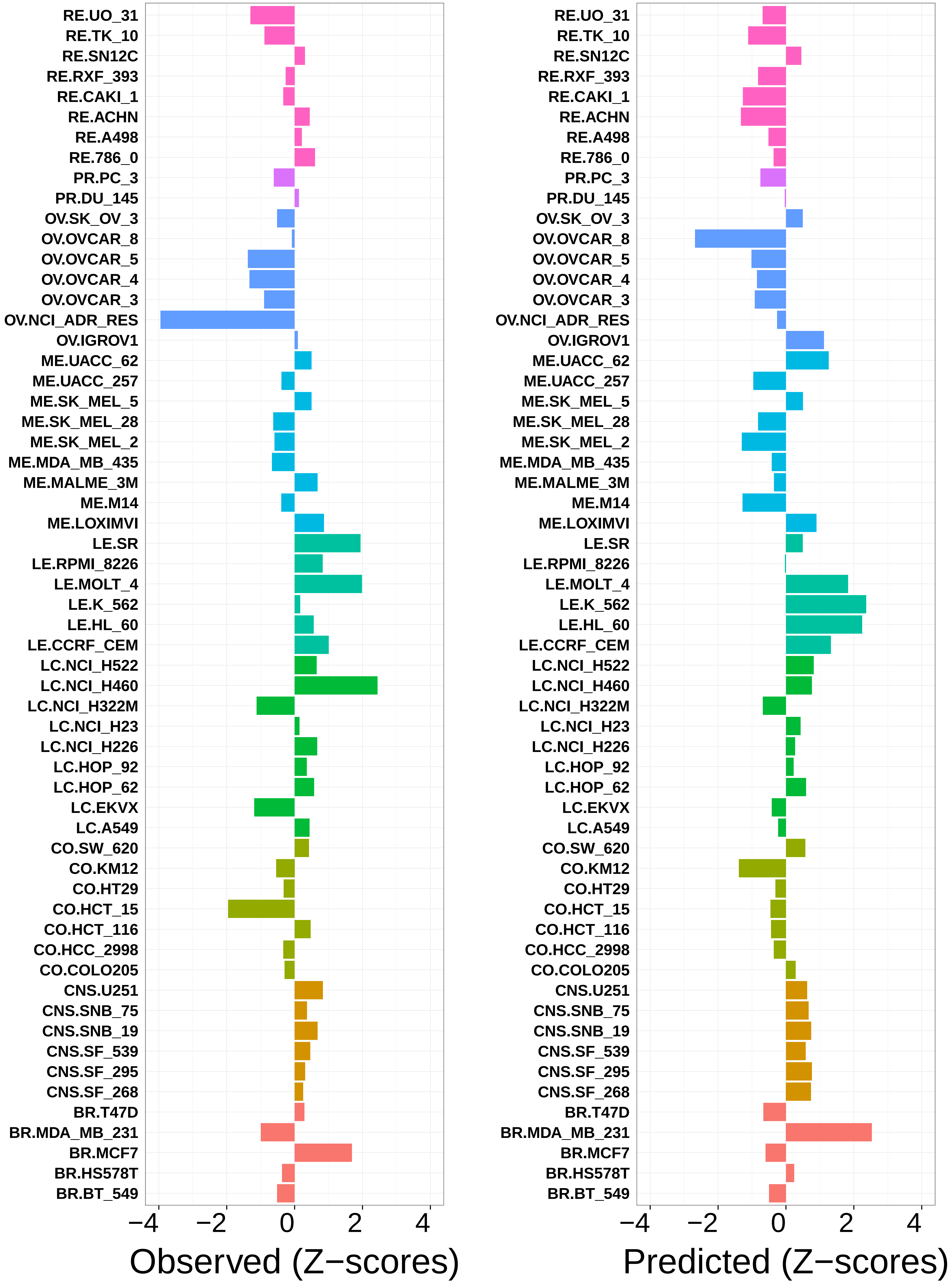

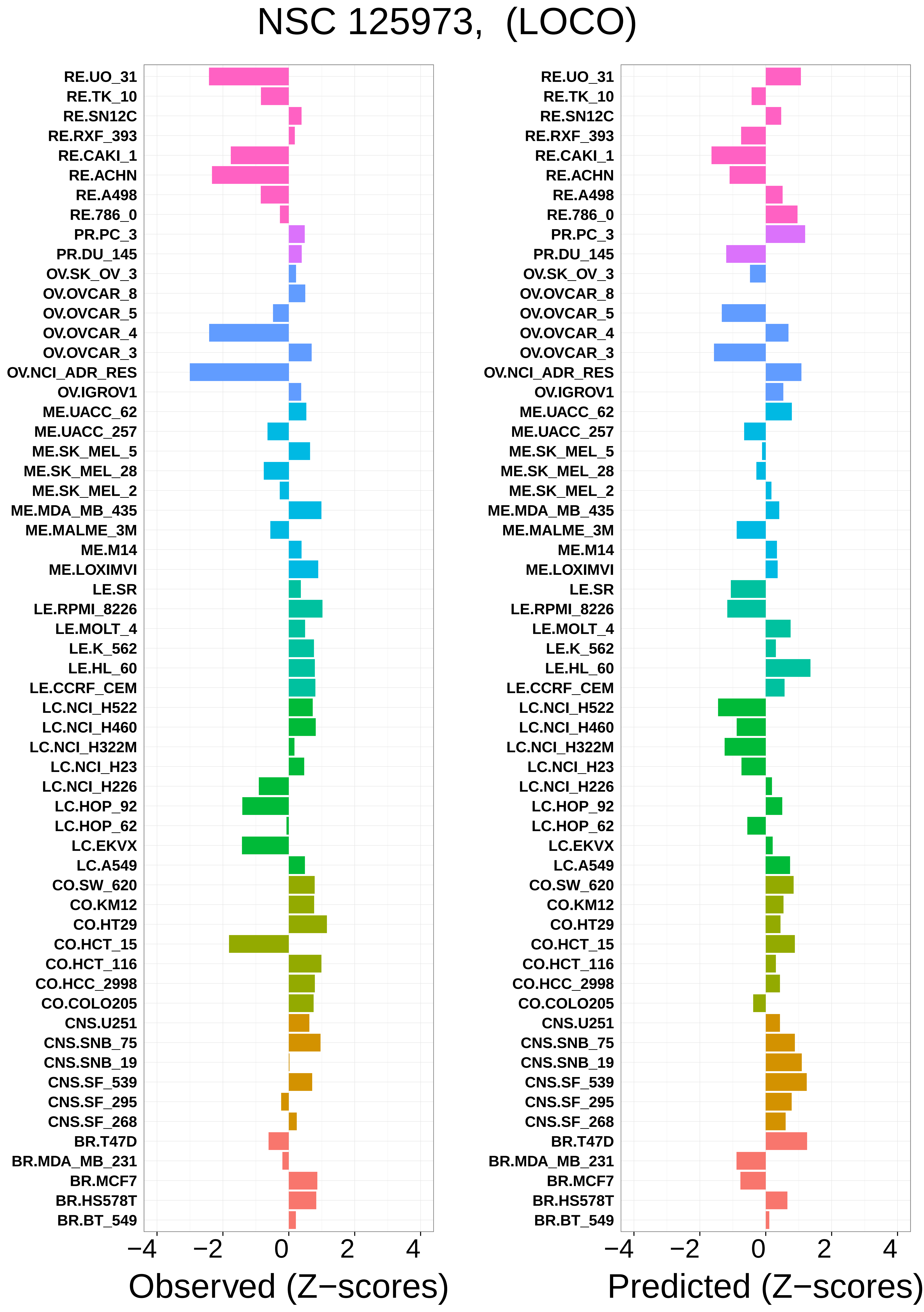
NSC 125973, (LOCO)
RE.UO_31
RE.TK_10
RE.SN12C
RE.RXF_393
RE.CAKI_1
RE.ACHN
RE.A498
RE.786_0
PR.PC_3
PR.DU_145
OV.SK_OV_3
OV.OVCAR_8
OV.OVCAR_5
OV.OVCAR_4
OV.OVCAR_3
OV.NCI_ADR_RES
OV.IGROV1
ME.UACC_62
ME.UACC_257
ME.SK_MEL_5
ME.SK_MEL_28
ME.SK_MEL_2
ME.MDA_MB_435
ME.MALME_3M
ME.M14
ME.LOXIMVI
LE.SR
LE.RPMI_8226
LE.MOLT_4
LE.K_562
LE.HL_60
LE.CCRF_CEM
LC.NCI_H522
LC.NCI_H460
LC.NCI_H322M
LC.NCI_H23
LC.NCI_H226
LC.HOP_92
LC.HOP_62
LC.EKVX
LC.A549
CO.SW_620
CO.KM12
CO.HT29
CO.HCT_15
CO.HCT_116
CO.HCC_2998
CO.COLO205
CNS.U251
CNS.SNB_75
CNS.SNB_19
CNS.SF_539
CNS.SF_295
CNS.SF_268
BR.T47D
BR.MDA_MB_231
BR.MCF7
BR.HS578T
BR.BT_549
−4 −2 0 2 4
Observed (Z−scores)
Predicted (Z−scores)

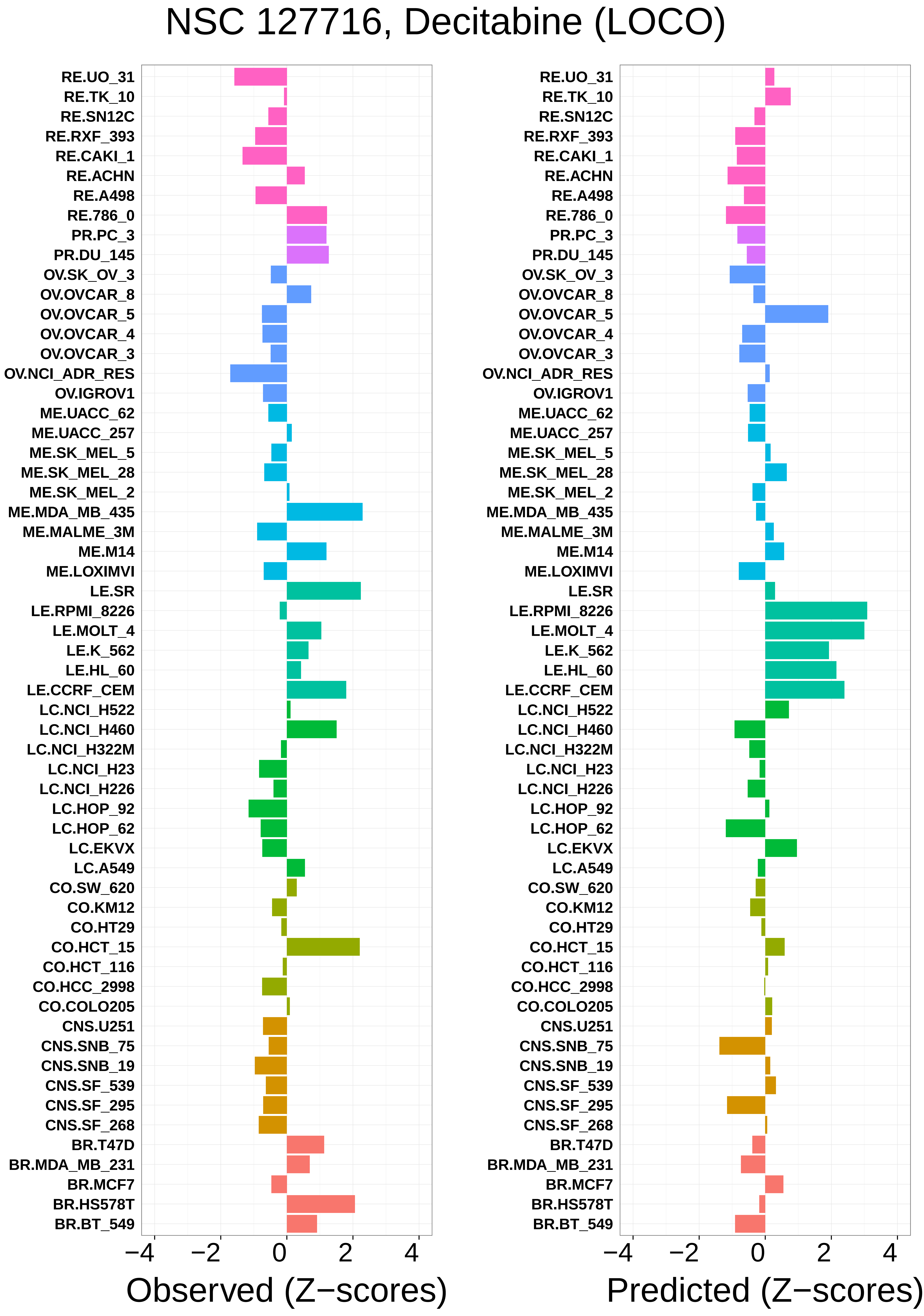
NSC 127716, Decitabine (LOCO)
RE.UO_31
RE.TK_10
RE.SN12C
RE.RXF_393
RE.CAKI_1
RE.ACHN
RE.A498
RE.786_0
PR.PC_3
PR.DU_145
OV.SK_OV_3
OV.OVCAR_8
OV.OVCAR_5
OV.OVCAR_4
OV.OVCAR_3
OV.NCI_ADR_RES
OV.IGROV1
ME.UACC_62
ME.UACC_257
ME.SK_MEL_5
ME.SK_MEL_28
ME.SK_MEL_2
ME.MDA_MB_435
ME.MALME_3M
ME.M14
ME.LOXIMVI
LE.SR
LE.RPMI_8226
LE.MOLT_4
LE.K_562
LE.HL_60
LE.CCRF_CEM
LC.NCI_H522
LC.NCI_H460
LC.NCI_H322M
LC.NCI_H23
LC.NCI_H226
LC.HOP_92
LC.HOP_62
LC.EKVX
LC.A549
CO.SW_620
CO.KM12
CO.HT29
CO.HCT_15
CO.HCT_116
CO.HCC_2998
CO.COLO205
CNS.U251
CNS.SNB_75
CNS.SNB_19
CNS.SF_539
CNS.SF_295
CNS.SF_268
BR.T47D
BR.MDA_MB_231
BR.MCF7
BR.HS578T
BR.BT_549
−4 −2 0 2 4
Observed (Z−scores)
−4 −2 0 2 4
Predicted (Z−scores)

NSC 141540, (LOCO)

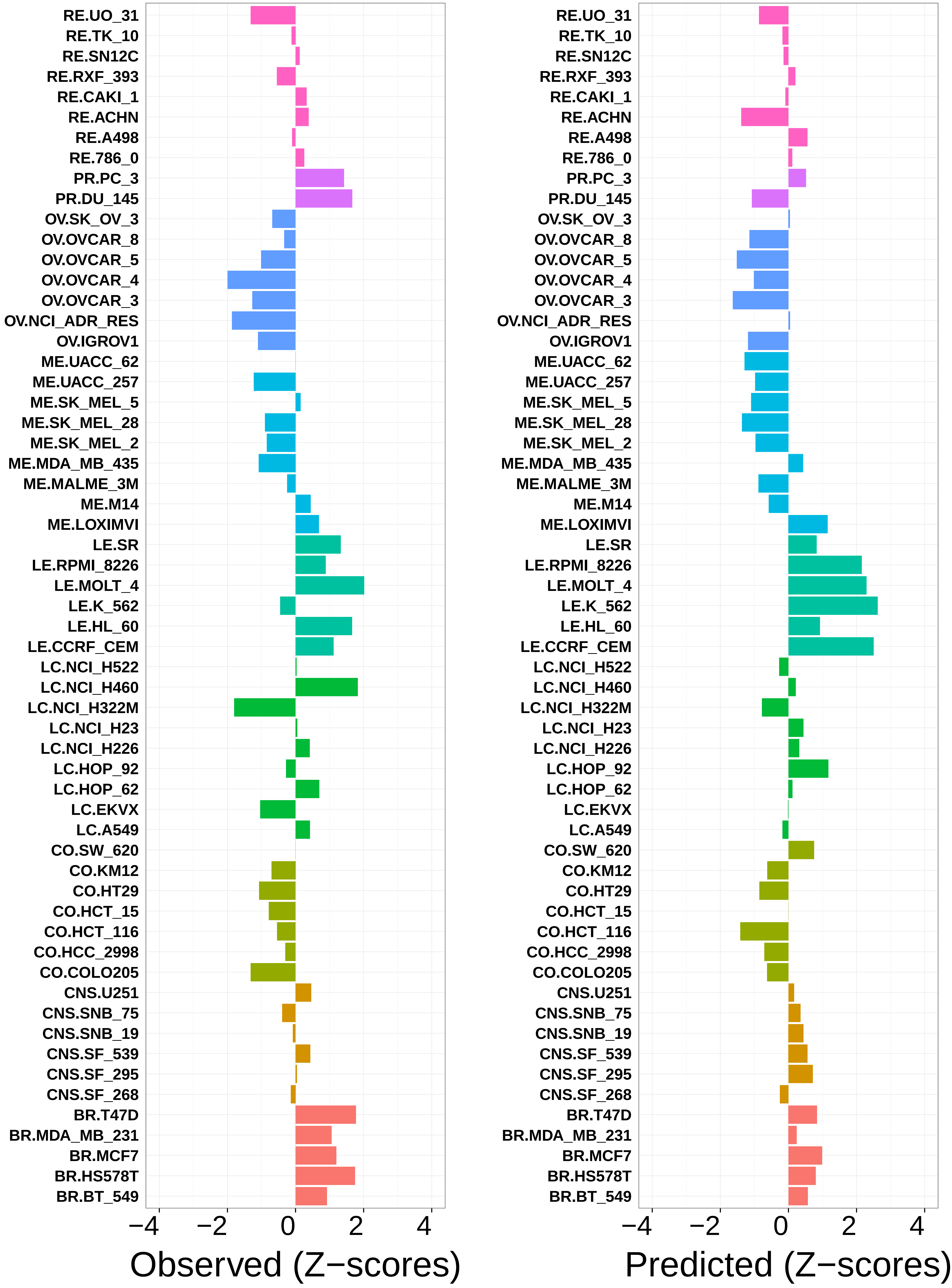

NSC 141633, (LOCO)

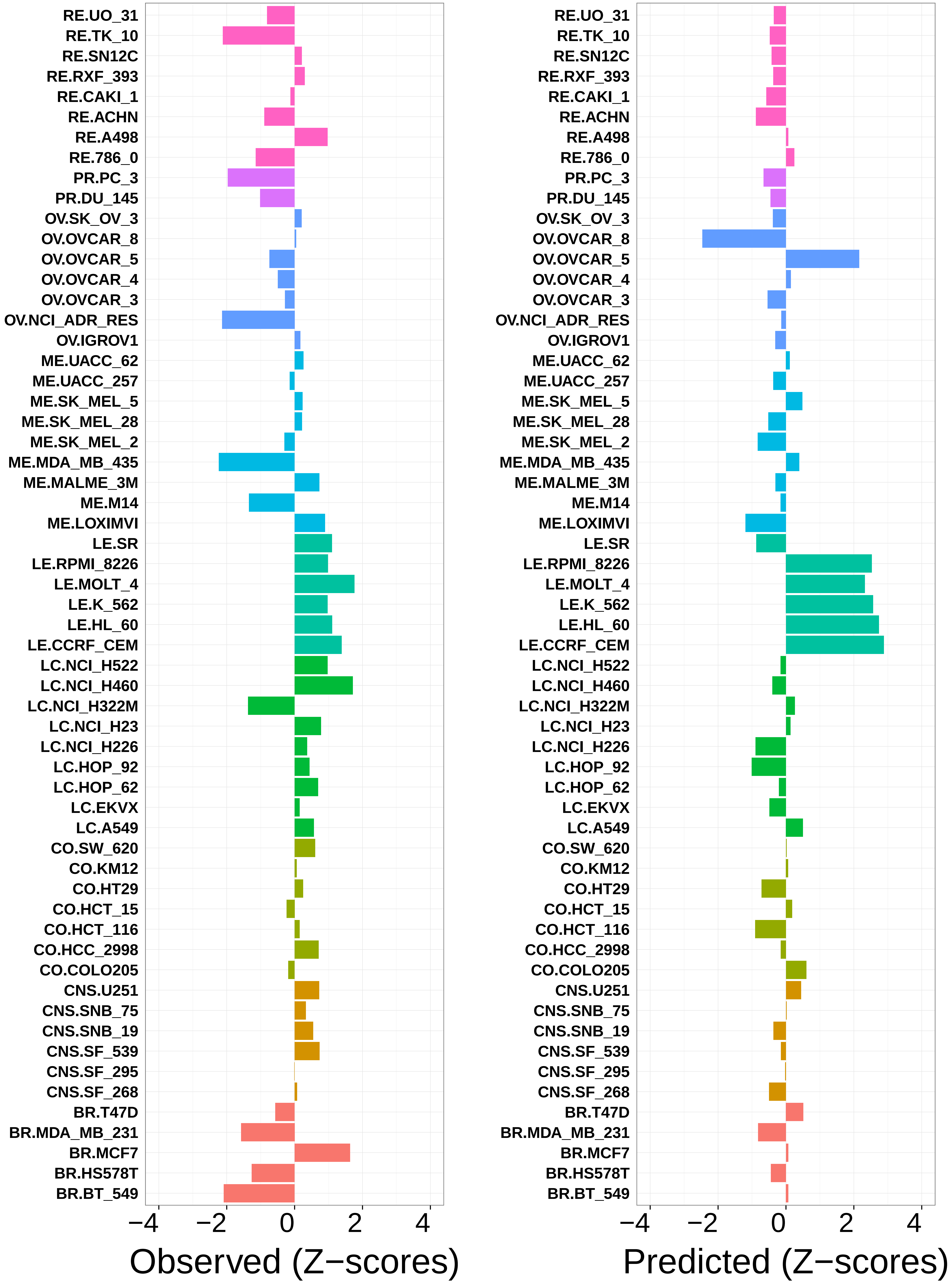

NSC 174939, (LOCO)

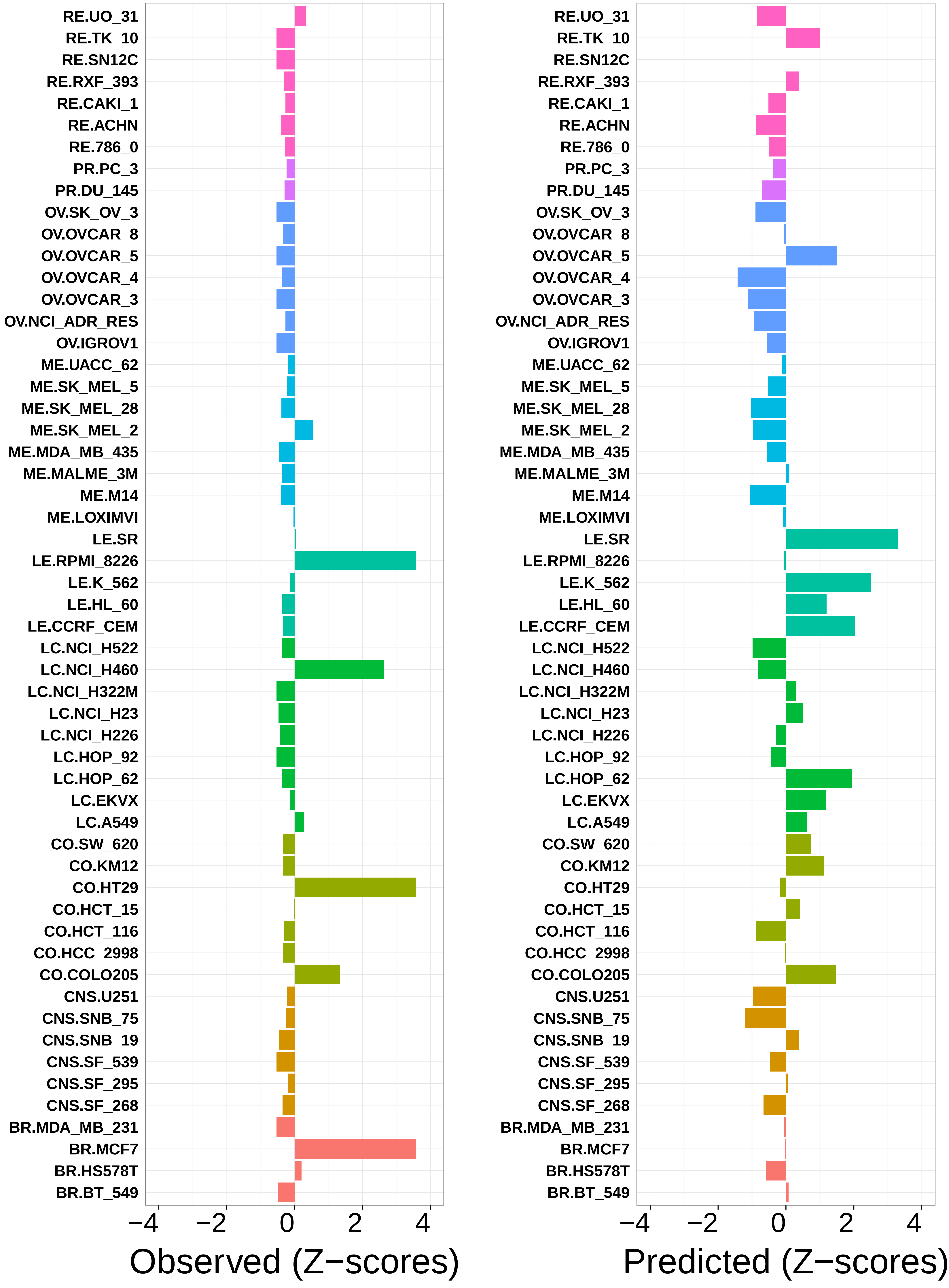

NSC 19893, Fluorouracil (LOCO)

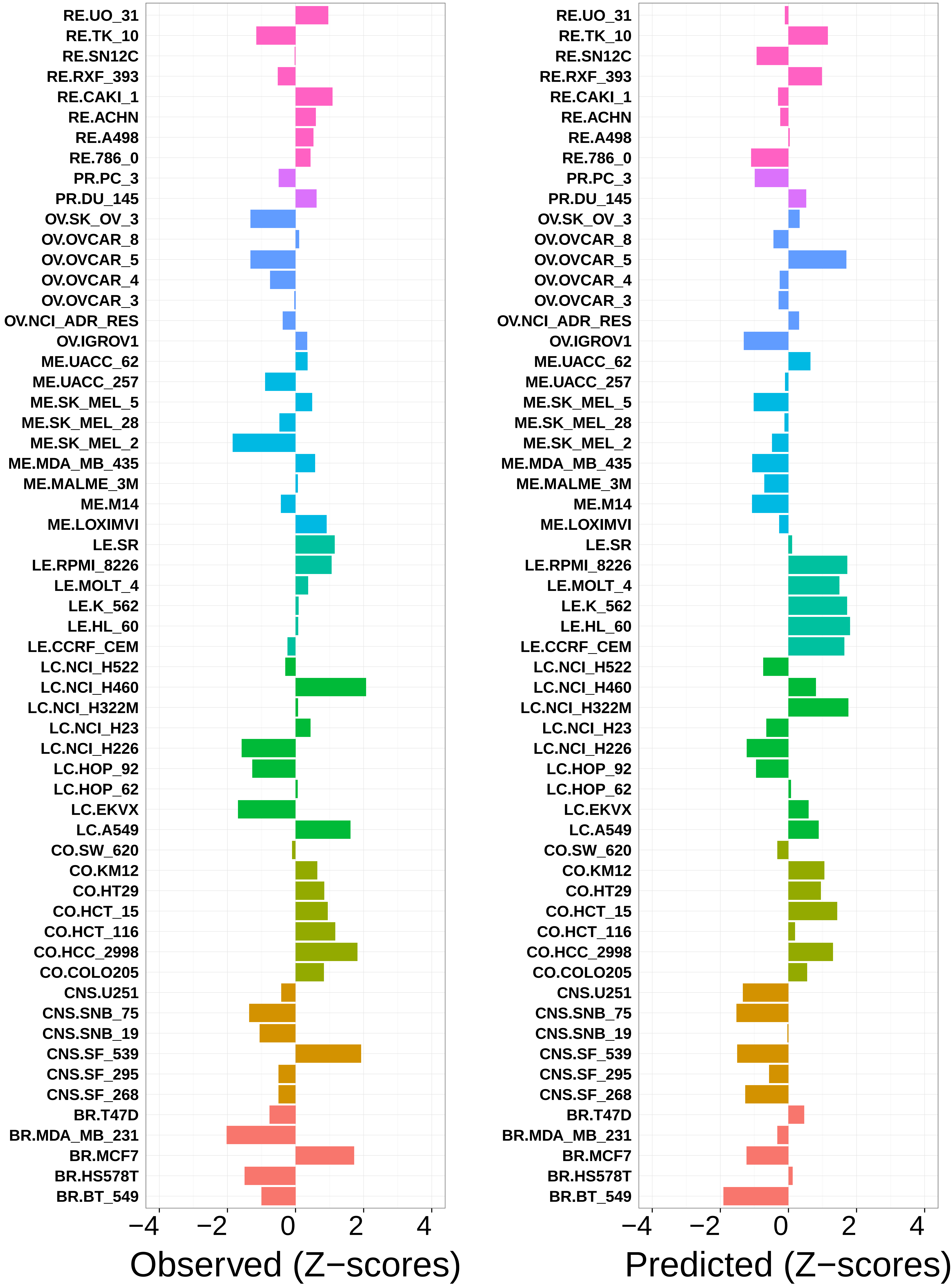

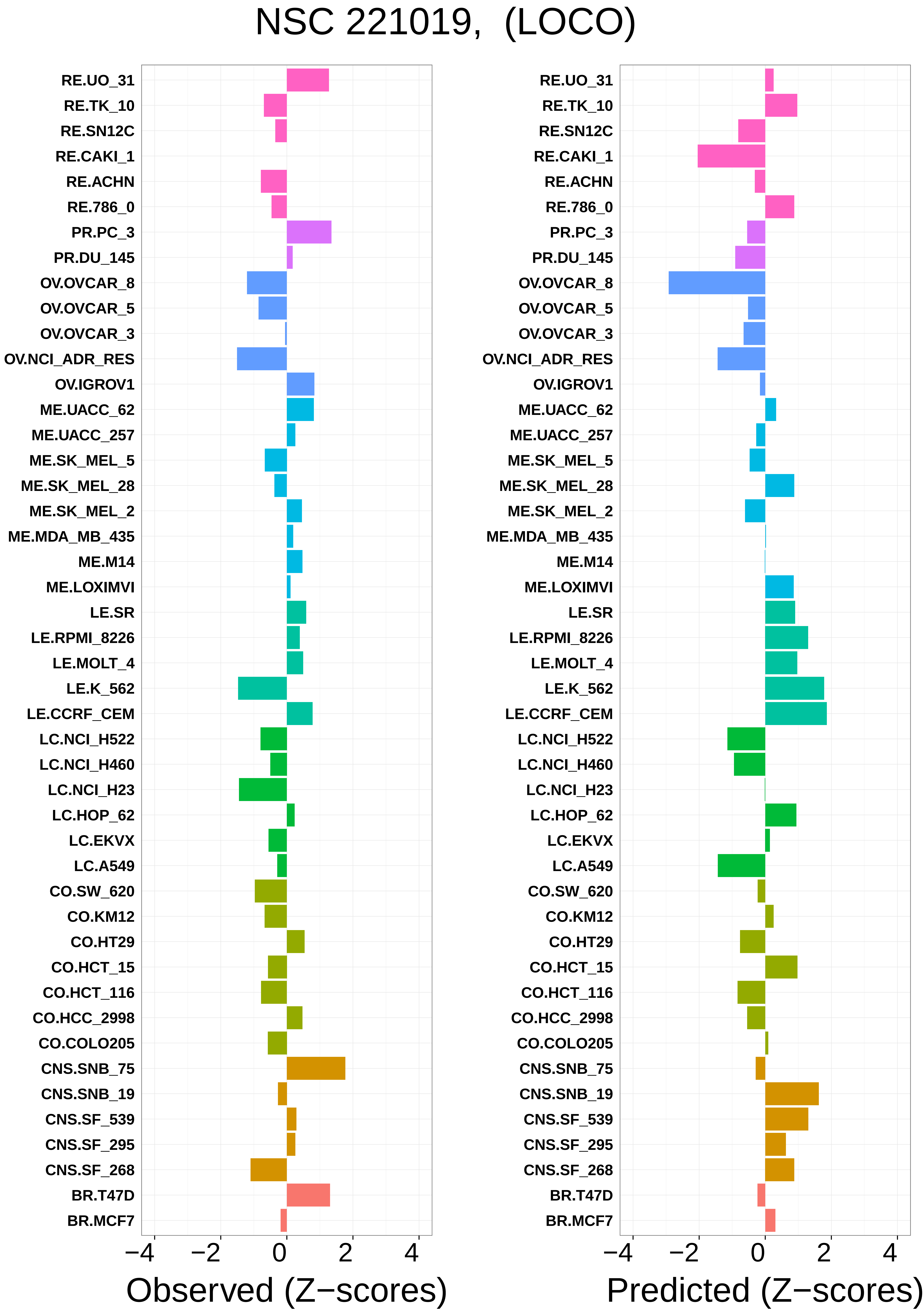
NSC 221019, (LOCO)
RE.UO_31
RE.TK_10
RE.SN12C
RE.CAKI_1
RE.ACHN
RE.786_0
PR.PC_3
PR.DU_145
OV.OVCAR_8
OV.OVCAR_5
OV.OVCAR_3
OV.NCI_ADR_RES
OV.IGROV1
ME.UACC_62
ME.UACC_257
ME.SK_MEL_5
ME.SK_MEL_28
ME.SK_MEL_2
ME.MDA_MB_435
ME.M14
ME.LOXIMVI
LE.SR
LE.RPMI_8226
LE.MOLT_4
LE.K_562
LE.CCRF_CEM
LC.NCI_H522
LC.NCI_H460
LC.NCI_H23
LC.HOP_62
LC.EKVX
LC.A549
CO.SW_620
CO.KM12
CO.HT29
CO.HCT_15
CO.HCT_116
CO.HCC_2998
CO.COLO205
CNS.SNB_75
CNS.SNB_19
CNS.SF_539
CNS.SF_295
CNS.SF_268
BR.T47D
BR.MCF7
−4
−2
0
2
4
Observed (Z−scores)
Predicted (Z−scores)

NSC 246131, (LOCO)

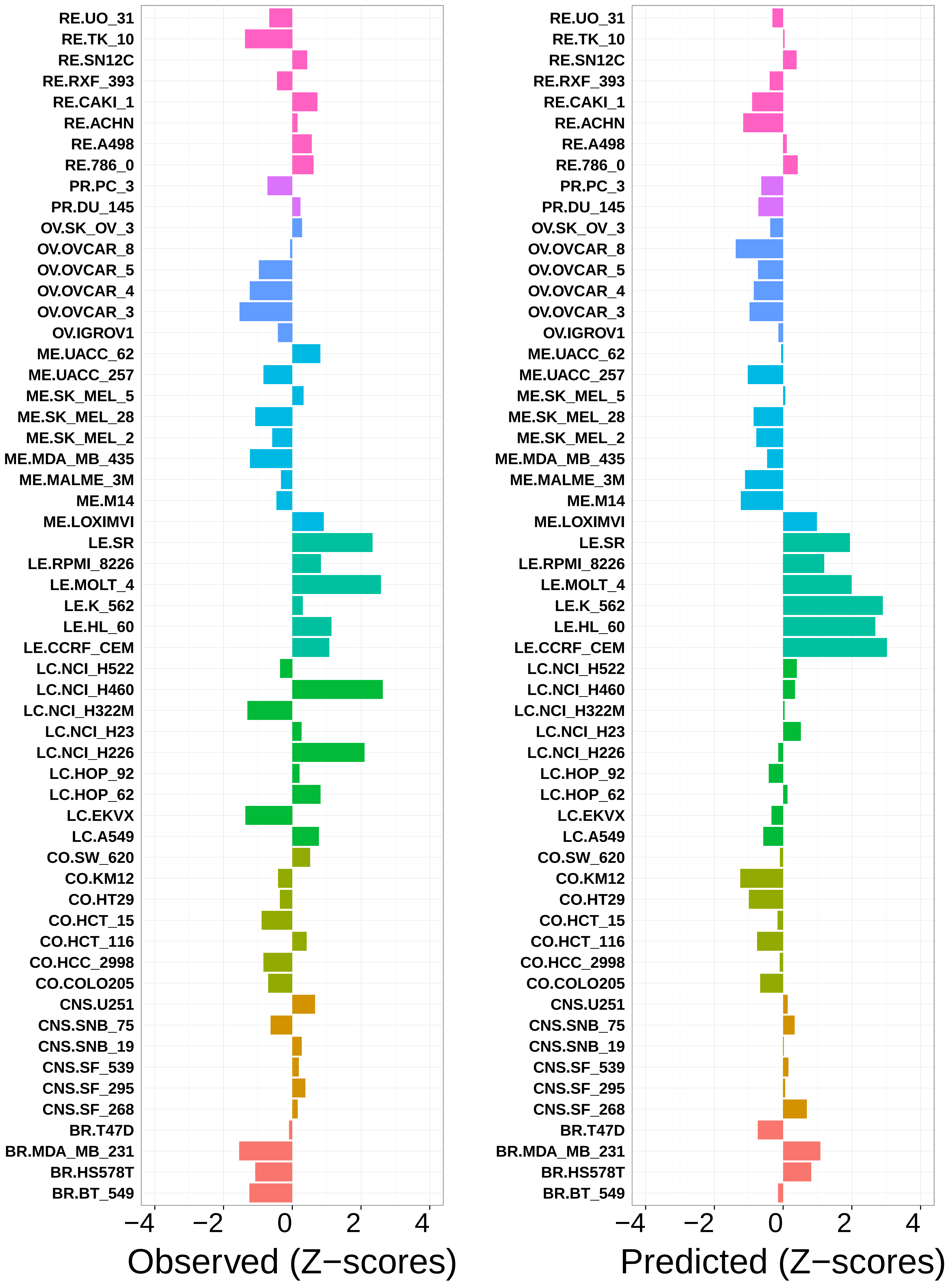

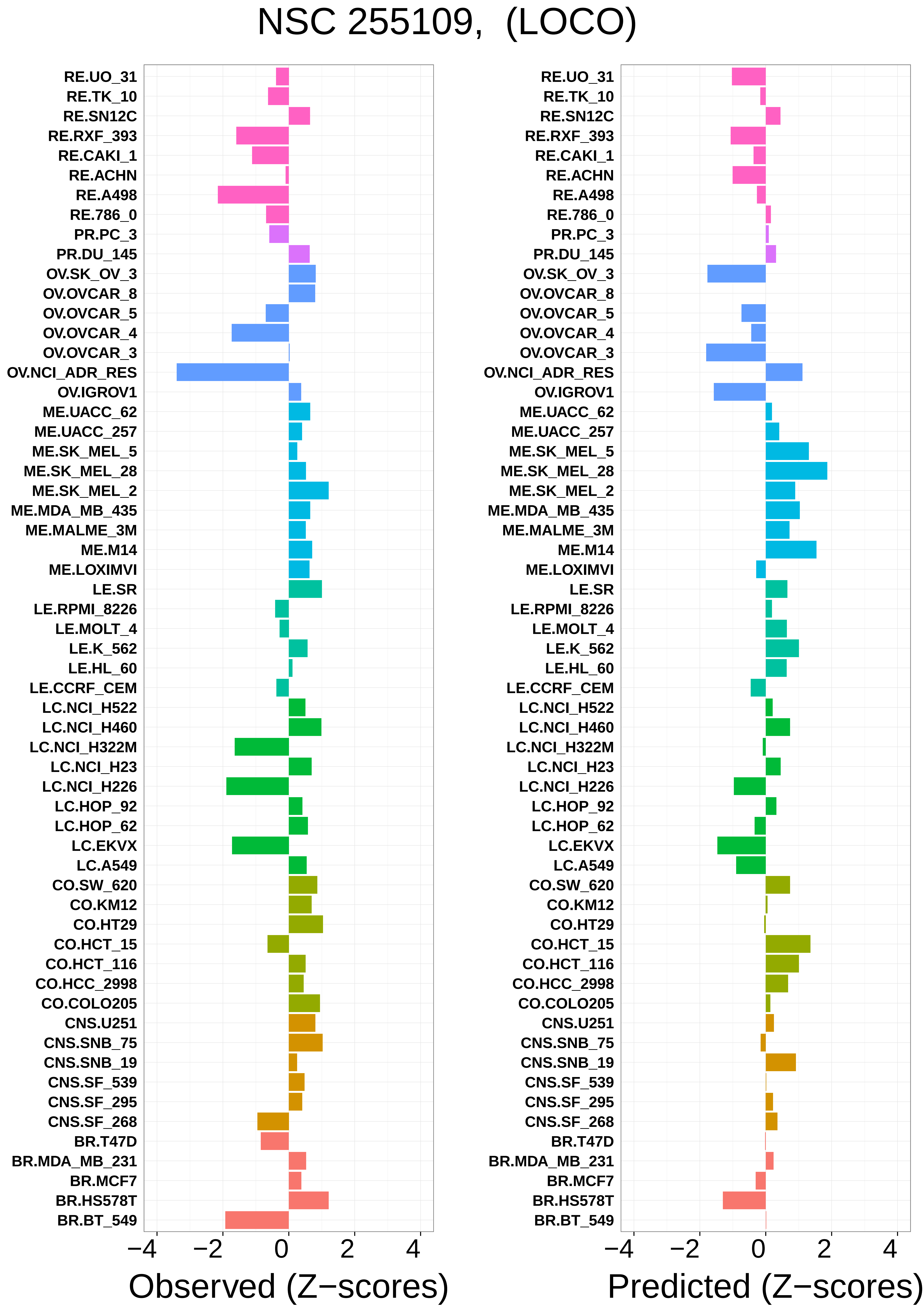
NSC 255109, (LOCO)
RE.UO_31
RE.TK_10
RE.SN12C
RE.RXF_393
RE.CAKI_1
RE.ACHN
RE.A498
RE.786_0
PR.PC_3
PR.DU_145
OV.SK_OV_3
OV.OVCAR_8
OV.OVCAR_5
OV.OVCAR_4
OV.OVCAR_3
OV.NCI_ADR_RES
OV.IGROV1
ME.UACC_62
ME.UACC_257
ME.SK_MEL_5
ME.SK_MEL_28
ME.SK_MEL_2
ME.MDA_MB_435
ME.MALME_3M
ME.M14
ME.LOXIMVI
LE.SR
LE.RPMI_8226
LE.MOLT_4
LE.K_562
LE.HL_60
LE.CCRF_CEM
LC.NCI_H522
LC.NCI_H460
LC.NCI_H322M
LC.NCI_H23
LC.NCI_H226
LC.HOP_92
LC.HOP_62
LC.EKVX
LC.A549
CO.SW_620
CO.KM12
CO.HT29
CO.HCT_15
CO.HCT_116
CO.HCC_2998
CO.COLO205
CNS.U251
CNS.SNB_75
CNS.SNB_19
CNS.SF_539
CNS.SF_295
CNS.SF_268
BR.T47D
BR.MDA_MB_231
BR.MCF7
BR.HS578T
BR.BT_549
−4
−2
0
2
4
Observed (Z−scores)
Predicted (Z−scores)

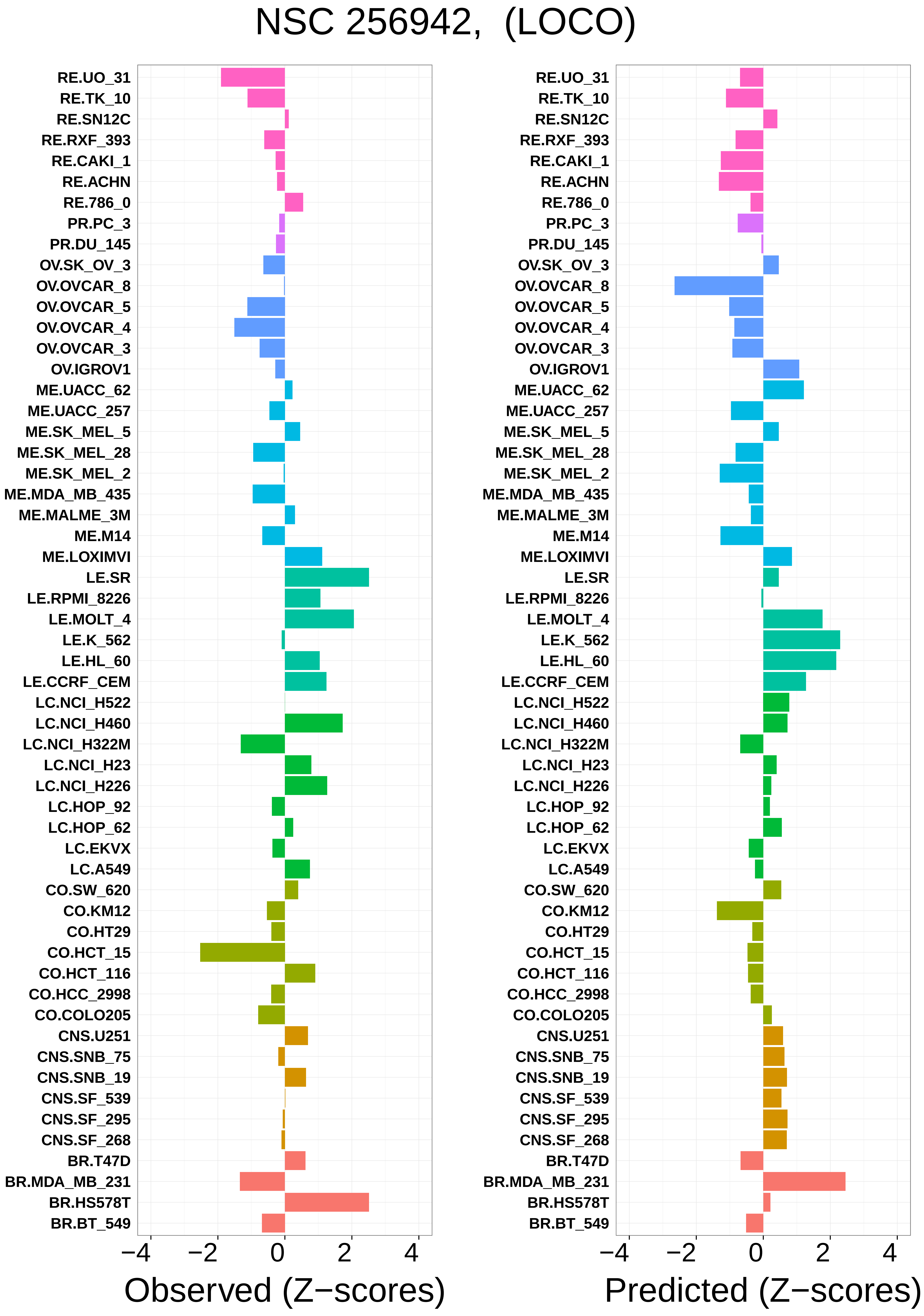
NSC 256942, (LOCO)
RE.UO_31
RE.TK_10
RE.SN12C
RE.RXF_393
RE.CAKI_1
RE.ACHN
RE.786_0
PR.PC_3
PR.DU_145
OV.SK_OV_3
OV.OVCAR_8
OV.OVCAR_5
OV.OVCAR_4
OV.OVCAR_3
OV.IGROV1
ME.UACC_62
ME.UACC_257
ME.SK_MEL_5
ME.SK_MEL_28
ME.SK_MEL_2
ME.MDA_MB_435
ME.MALME_3M
ME.M14
ME.LOXIMVI
LE.SR
LE.RPMI_8226
LE.MOLT_4
LE.K_562
LE.HL_60
LE.CCRF_CEM
LC.NCI_H522
LC.NCI_H460
LC.NCI_H322M
LC.NCI_H23
LC.NCI_H226
LC.HOP_92
LC.HOP_62
LC.EKVX
LC.A549
CO.SW_620
CO.KM12
CO.HT29
CO.HCT_15
CO.HCT_116
CO.HCC_2998
CO.COLO205
CNS.U251
CNS.SNB_75
CNS.SNB_19
CNS.SF_539
CNS.SF_295
CNS.SF_268
BR.T47D
BR.MDA_MB_231
BR.HS578T
BR.BT_549
−4
−2
0
2
4
Observed (Z−scores)
Predicted (Z−scores)

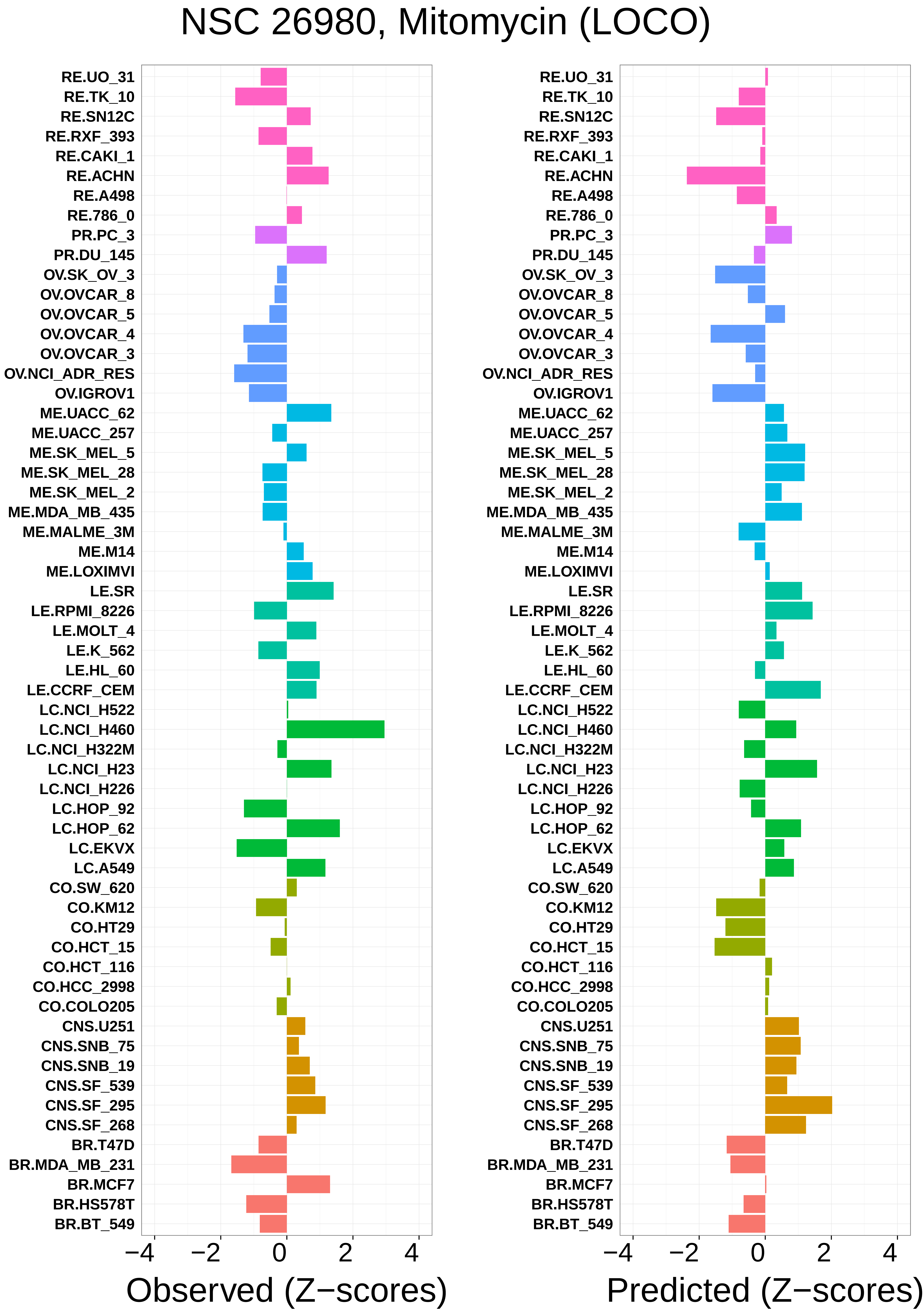
NSC 26980, Mitomycin (LOCO)
RE.UO_31
RE.TK_10
RE.SN12C
RE.RXF_393
RE.CAKI_1
RE.ACHN
RE.A498
RE.786_0
PR.PC_3
PR.DU_145
OV.SK_OV_3
OV.OVCAR_8
OV.OVCAR_5
OV.OVCAR_4
OV.OVCAR_3
OV.NCI_ADR_RES
OV.IGROV1
ME.UACC_62
ME.UACC_257
ME.SK_MEL_5
ME.SK_MEL_28
ME.SK_MEL_2
ME.MDA_MB_435
ME.MALME_3M
ME.M14
ME.LOXIMVI
LE.SR
LE.RPMI_8226
LE.MOLT_4
LE.K_562
LE.HL_60
LE.CCRF_CEM
LC.NCI_H522
LC.NCI_H460
LC.NCI_H322M
LC.NCI_H23
LC.NCI_H226
LC.HOP_92
LC.HOP_62
LC.EKVX
LC.A549
CO.SW_620
CO.KM12
CO.HT29
CO.HCT_15
CO.HCT_116
CO.HCC_2998
CO.COLO205
CNS.U251
CNS.SNB_75
CNS.SNB_19
CNS.SF_539
CNS.SF_295
CNS.SF_268
BR.T47D
BR.MDA_MB_231
BR.MCF7
BR.HS578T
BR.BT_549
−4
−2
0
2
4
Observed (Z−scores)
Predicted (Z−scores)

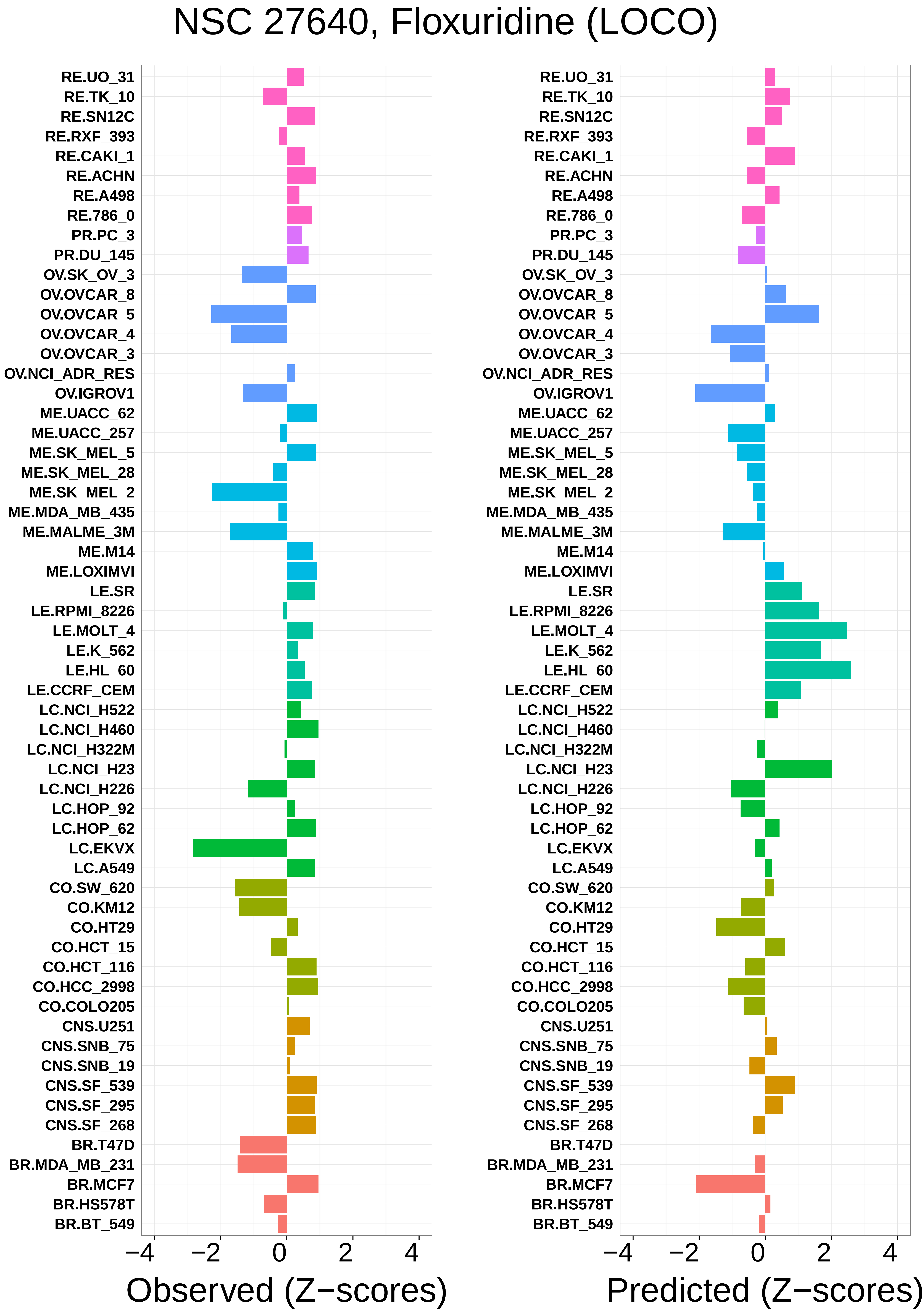
NSC 27640, Floxuridine (LOCO)
RE.UO_31
RE.TK_10
RE.SN12C
RE.RXF_393
RE.CAKI_1
RE.ACHN
RE.A498
RE.786_0
PR.PC_3
PR.DU_145
OV.SK_OV_3
OV.OVCAR_8
OV.OVCAR_5
OV.OVCAR_4
OV.OVCAR_3
OV.NCI_ADR_RES
OV.IGROV1
ME.UACC_62
ME.UACC_257
ME.SK_MEL_5
ME.SK_MEL_28
ME.SK_MEL_2
ME.MDA_MB_435
ME.MALME_3M
ME.M14
ME.LOXIMVI
LE.SR
LE.RPMI_8226
LE.MOLT_4
LE.K_562
LE.HL_60
LE.CCRF_CEM
LC.NCI_H522
LC.NCI_H460
LC.NCI_H322M
LC.NCI_H23
LC.NCI_H226
LC.HOP_92
LC.HOP_62
LC.EKVX
LC.A549
CO.SW_620
CO.KM12
CO.HT29
CO.HCT_15
CO.HCT_116
CO.HCC_2998
CO.COLO205
CNS.U251
CNS.SNB_75
CNS.SNB_19
CNS.SF_539
CNS.SF_295
CNS.SF_268
BR.T47D
BR.MDA_MB_231
BR.MCF7
BR.HS578T
BR.BT_549
−4 −2 0 2 4
Observed (Z−scores)
Predicted (Z−scores)

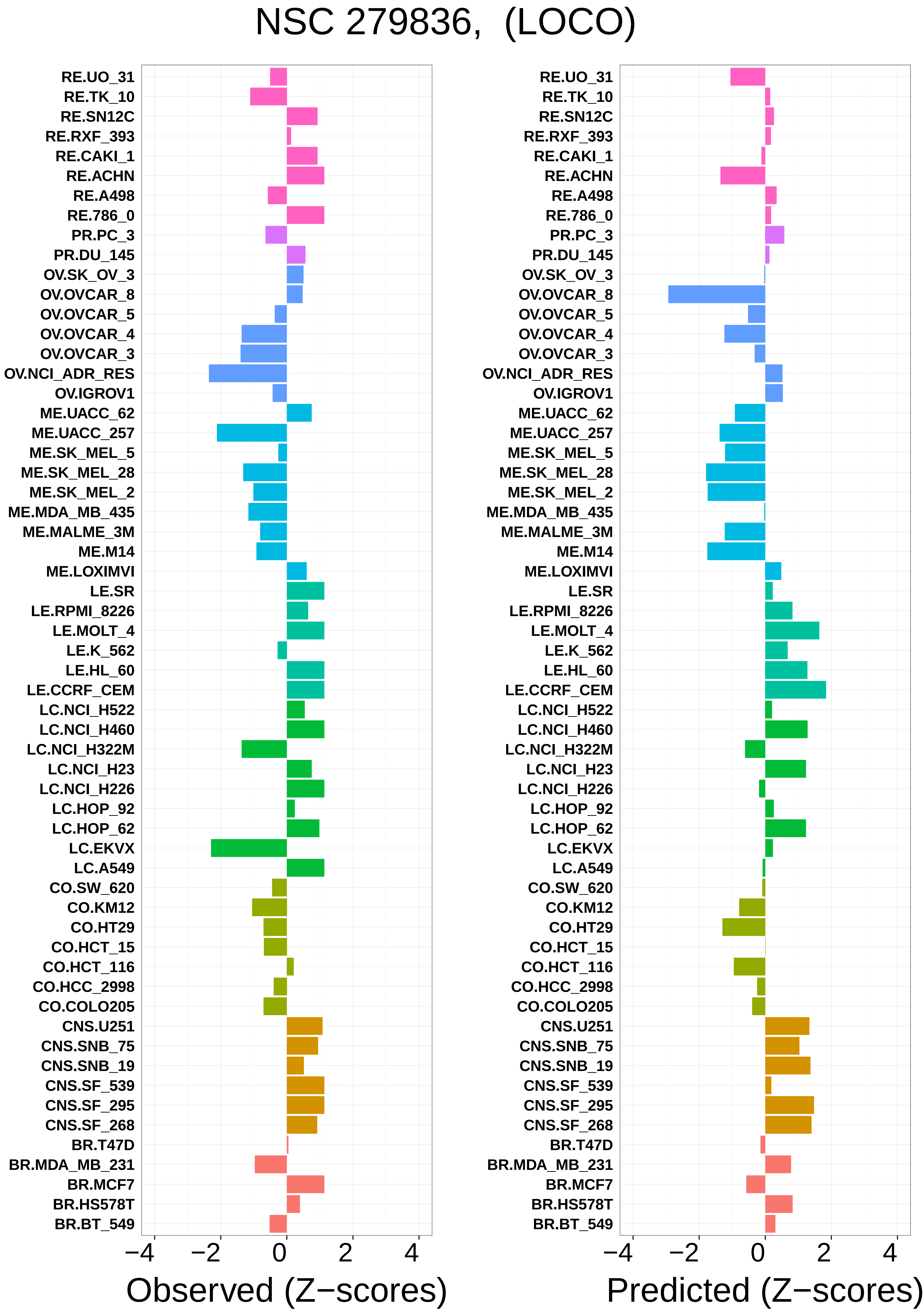
NSC 279836, (LOCO)
RE.UO_31
RE.TK_10
RE.SN12C
RE.RXF_393
RE.CAKI_1
RE.ACHN
RE.A498
RE.786_0
PR.PC_3
PR.DU_145
OV.SK_OV_3
OV.OVCAR_8
OV.OVCAR_5
OV.OVCAR_4
OV.OVCAR_3
OV.NCI_ADR_RES
OV.IGROV1
ME.UACC_62
ME.UACC_257
ME.SK_MEL_5
ME.SK_MEL_28
ME.SK_MEL_2
ME.MDA_MB_435
ME.MALME_3M
ME.M14
ME.LOXIMVI
LE.SR
LE.RPMI_8226
LE.MOLT_4
LE.K_562
LE.HL_60
LE.CCRF_CEM
LC.NCI_H522
LC.NCI_H460
LC.NCI_H322M
LC.NCI_H23
LC.NCI_H226
LC.HOP_92
LC.HOP_62
LC.EKVX
LC.A549
CO.SW_620
CO.KM12
CO.HT29
CO.HCT_15
CO.HCT_116
CO.HCC_2998
CO.COLO205
CNS.U251
CNS.SNB_75
CNS.SNB_19
CNS.SF_539
CNS.SF_295
CNS.SF_268
BR.T47D
BR.MDA_MB_231
BR.MCF7
BR.HS578T
BR.BT_549
−4 −2 0 2 4
Observed (Z−scores)
Predicted (Z−scores)

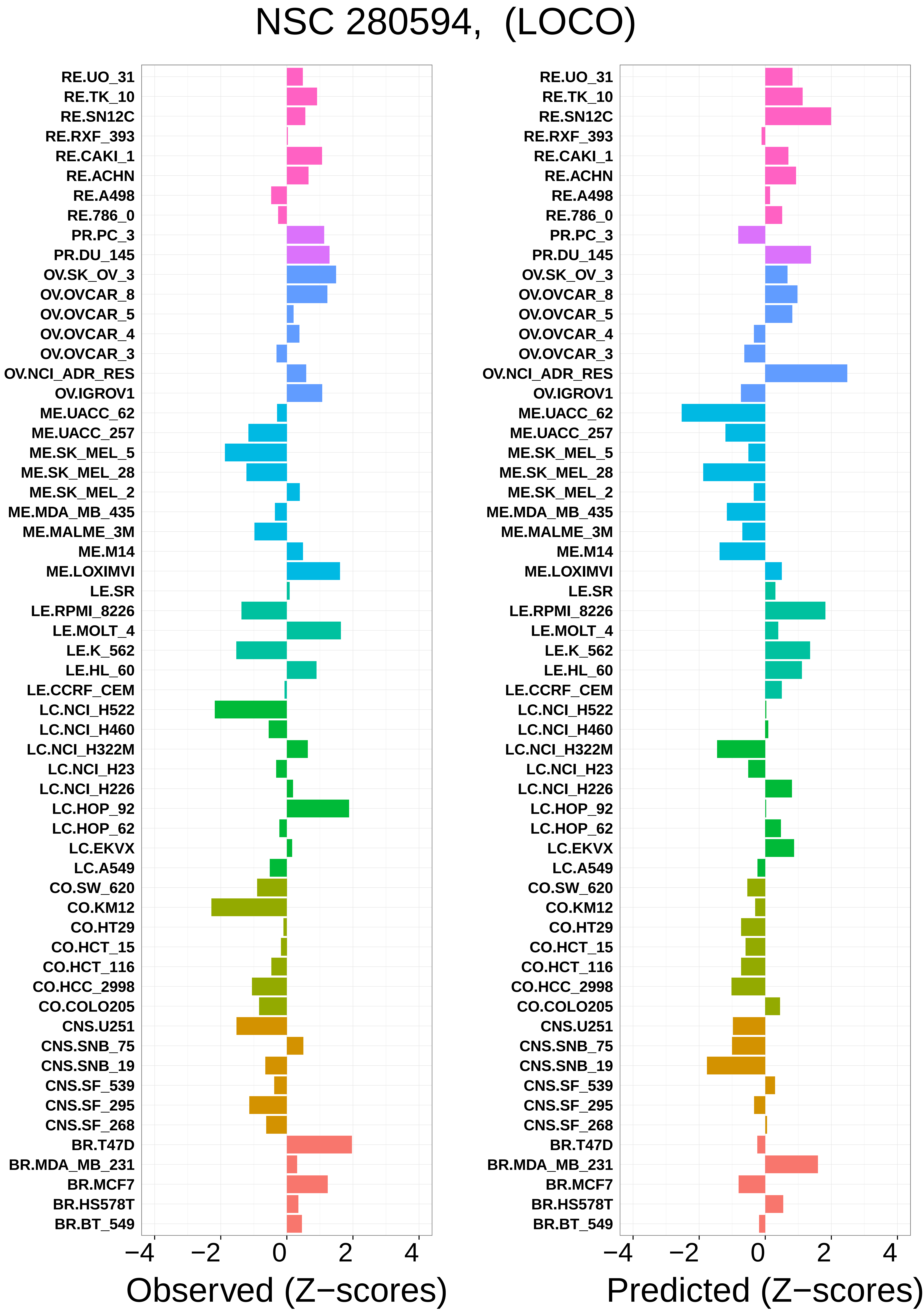
NSC 280594, (LOCO)
RE.UO_31
RE.TK_10
RE.SN12C
RE.RXF_393
RE.CAKI_1
RE.ACHN
RE.A498
RE.786_0
PR.PC_3
PR.DU_145
OV.SK_OV_3
OV.OVCAR_8
OV.OVCAR_5
OV.OVCAR_4
OV.OVCAR_3
OV.NCI_ADR_RES
OV.IGROV1
ME.UACC_62
ME.UACC_257
ME.SK_MEL_5
ME.SK_MEL_28
ME.SK_MEL_2
ME.MDA_MB_435
ME.MALME_3M
ME.M14
ME.LOXIMVI
LE.SR
LE.RPMI_8226
LE.MOLT_4
LE.K_562
LE.HL_60
LE.CCRF_CEM
LC.NCI_H522
LC.NCI_H460
LC.NCI_H322M
LC.NCI_H23
LC.NCI_H226
LC.HOP_92
LC.HOP_62
LC.EKVX
LC.A549
CO.SW_620
CO.KM12
CO.HT29
CO.HCT_15
CO.HCT_116
CO.HCC_2998
CO.COLO205
CNS.U251
CNS.SNB_75
CNS.SNB_19
CNS.SF_539
CNS.SF_295
CNS.SF_268
BR.T47D
BR.MDA_MB_231
BR.MCF7
BR.HS578T
BR.BT_549
−4 −2 0 2 4
Observed (Z−scores)
Predicted (Z−scores)

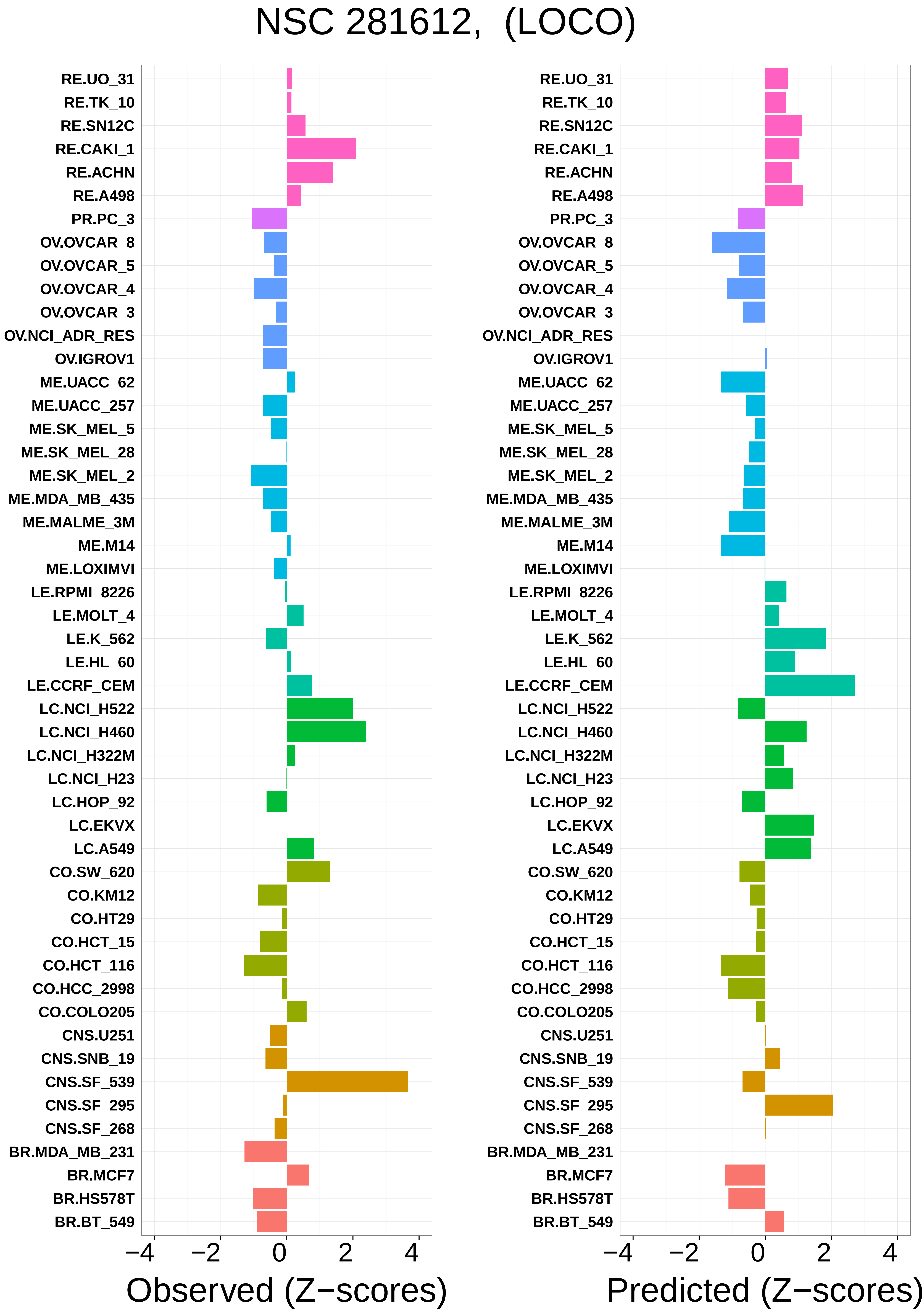
NSC 281612, (LOCO)
RE.UO_31
RE.TK_10
RE.SN12C
RE.CAKI_1
RE.ACHN
RE.A498
PR.PC_3
OV.OVCAR_8
OV.OVCAR_5
OV.OVCAR_4
OV.OVCAR_3
OV.NCI_ADR_RES
OV.IGROV1
ME.UACC_62
ME.UACC_257
ME.SK_MEL_5
ME.SK_MEL_28
ME.SK_MEL_2
ME.MDA_MB_435
ME.MALME_3M
ME.M14
ME.LOXIMVI
LE.RPMI_8226
LE.MOLT_4
LE.K_562
LE.HL_60
LE.CCRF_CEM
LC.NCI_H522
LC.NCI_H460
LC.NCI_H322M
LC.NCI_H23
LC.HOP_92
LC.EKVX
LC.A549
CO.SW_620
CO.KM12
CO.HT29
CO.HCT_15
CO.HCT_116
CO.HCC_2998
CO.COLO205
CNS.U251
CNS.SNB_19
CNS.SF_539
CNS.SF_295
CNS.SF_268
BR.MDA_MB_231
BR.MCF7
BR.HS578T
BR.BT_549
−4 −2 0 2 4
Observed (Z−scores)
−4 −2 0 2 4
Predicted (Z−scores)

NSC 287459, (LOCO)

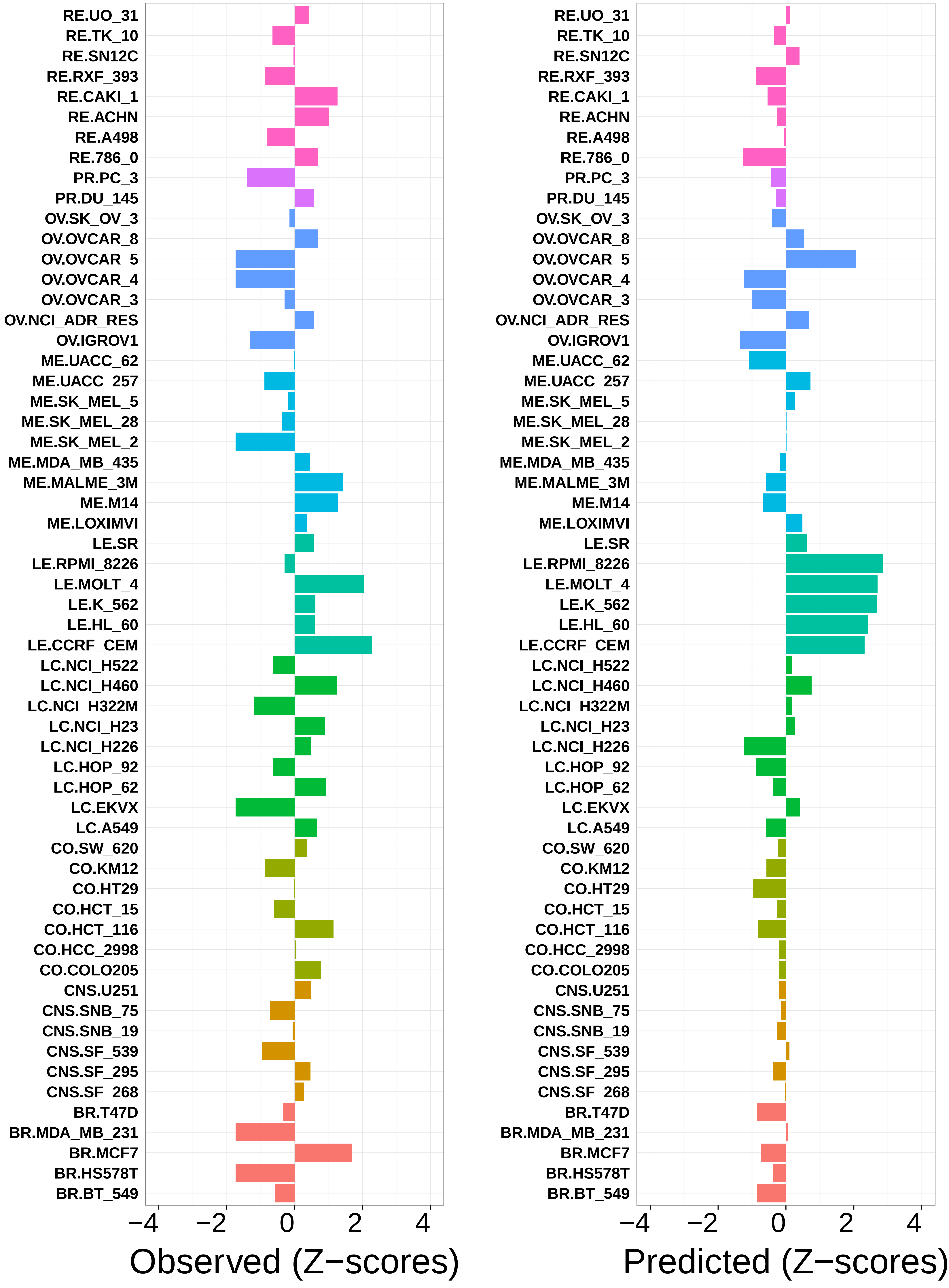

NSC 301739, (LOCO)

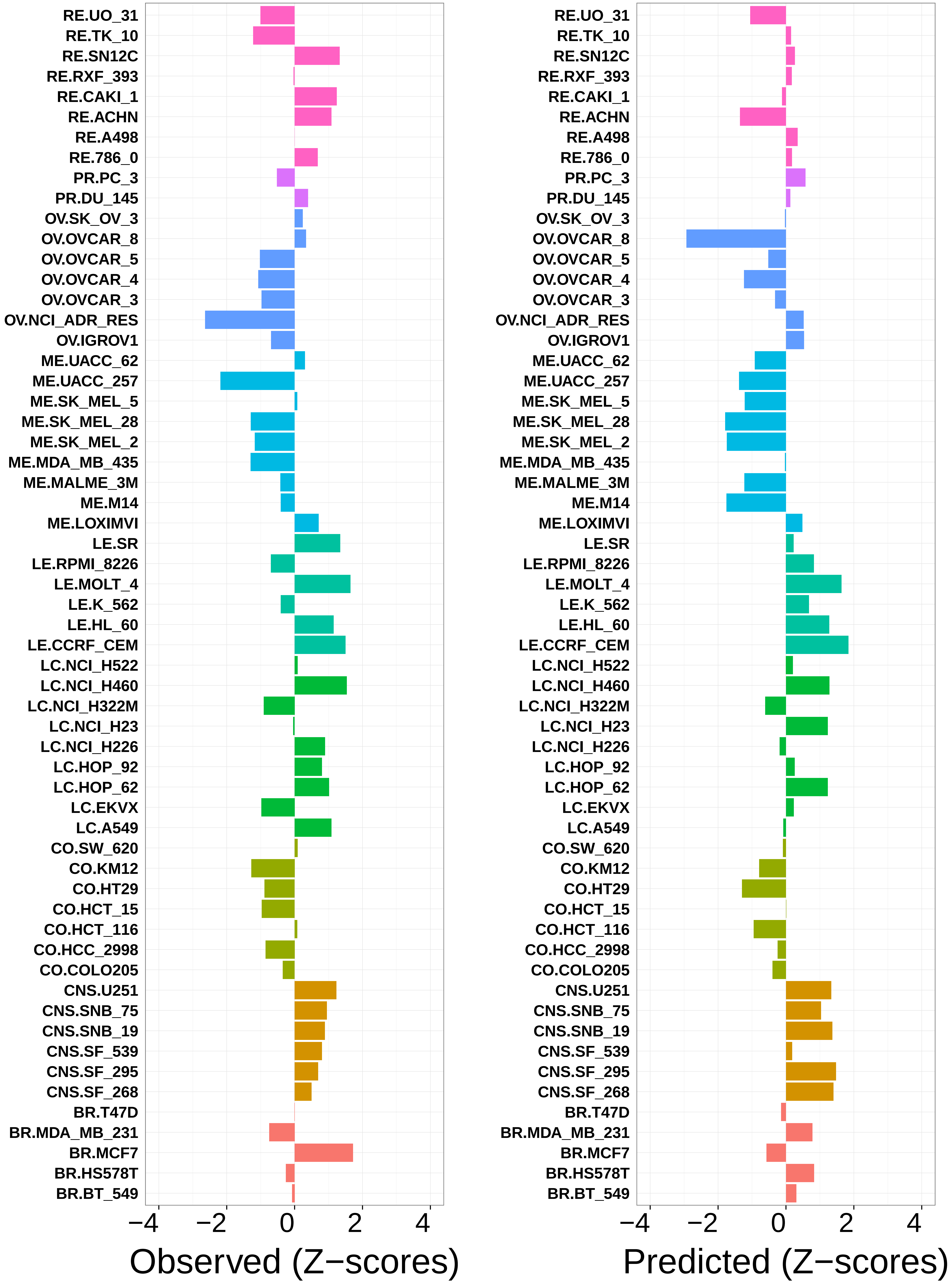

NSC 312887, Fludarabine_phosphate_(USAN) (LOCO)

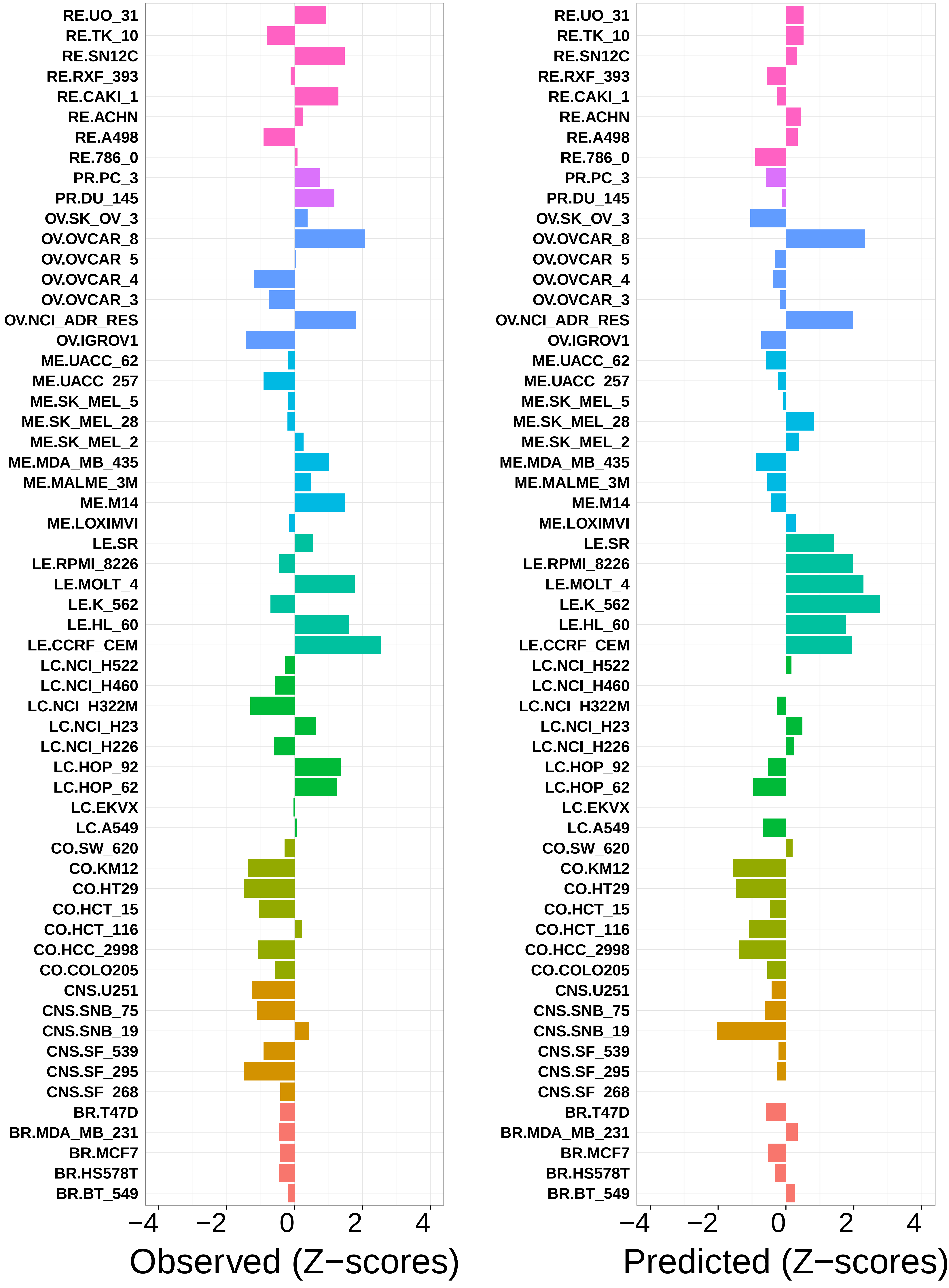

NSC 330507, Tanespimycin (LOCO)

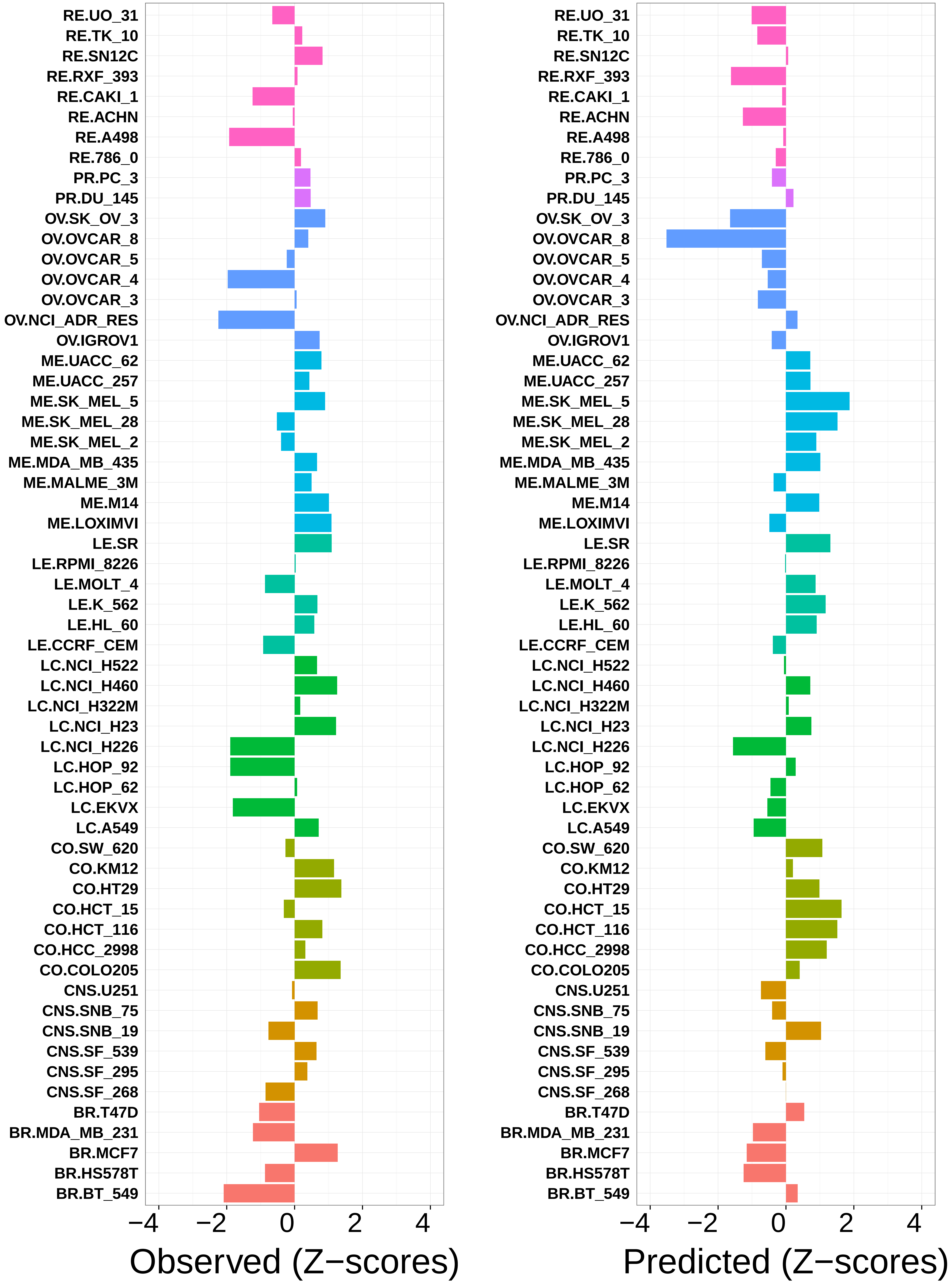

NSC 34521, Dextromethorphan_Hydrobromide (LOCO)

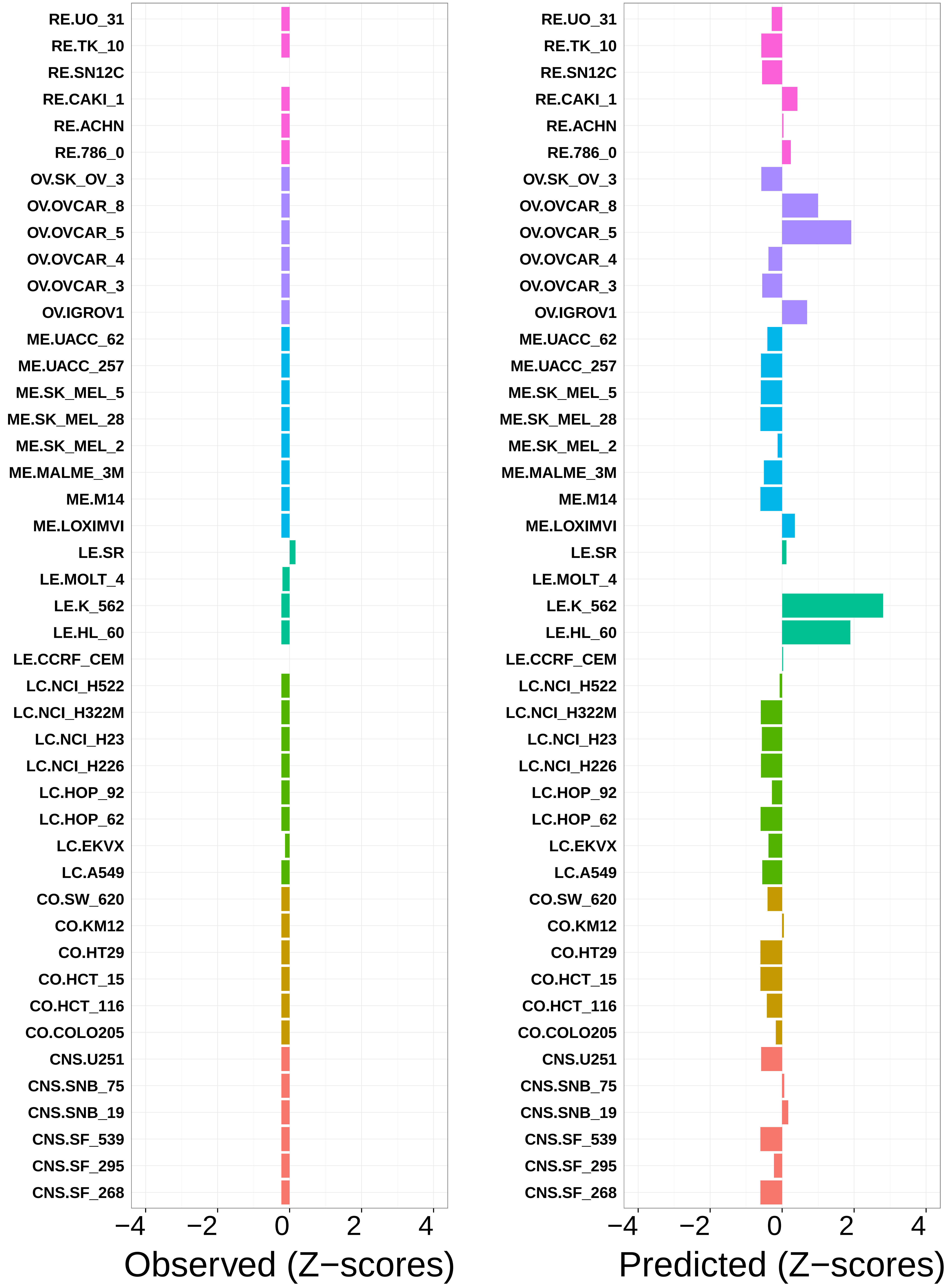

NSC 354258, 8−Chloro−Adenosine (LOCO)

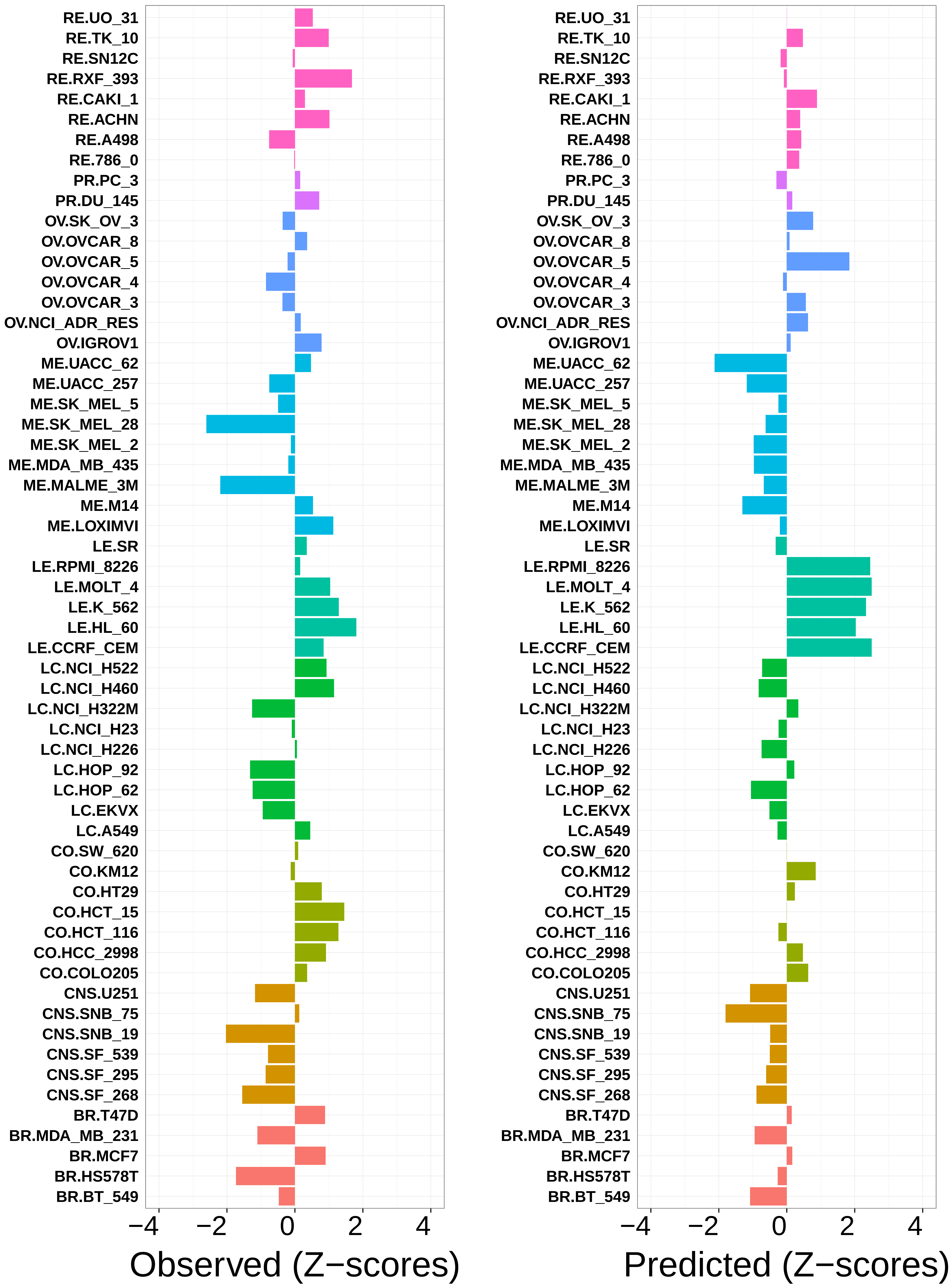

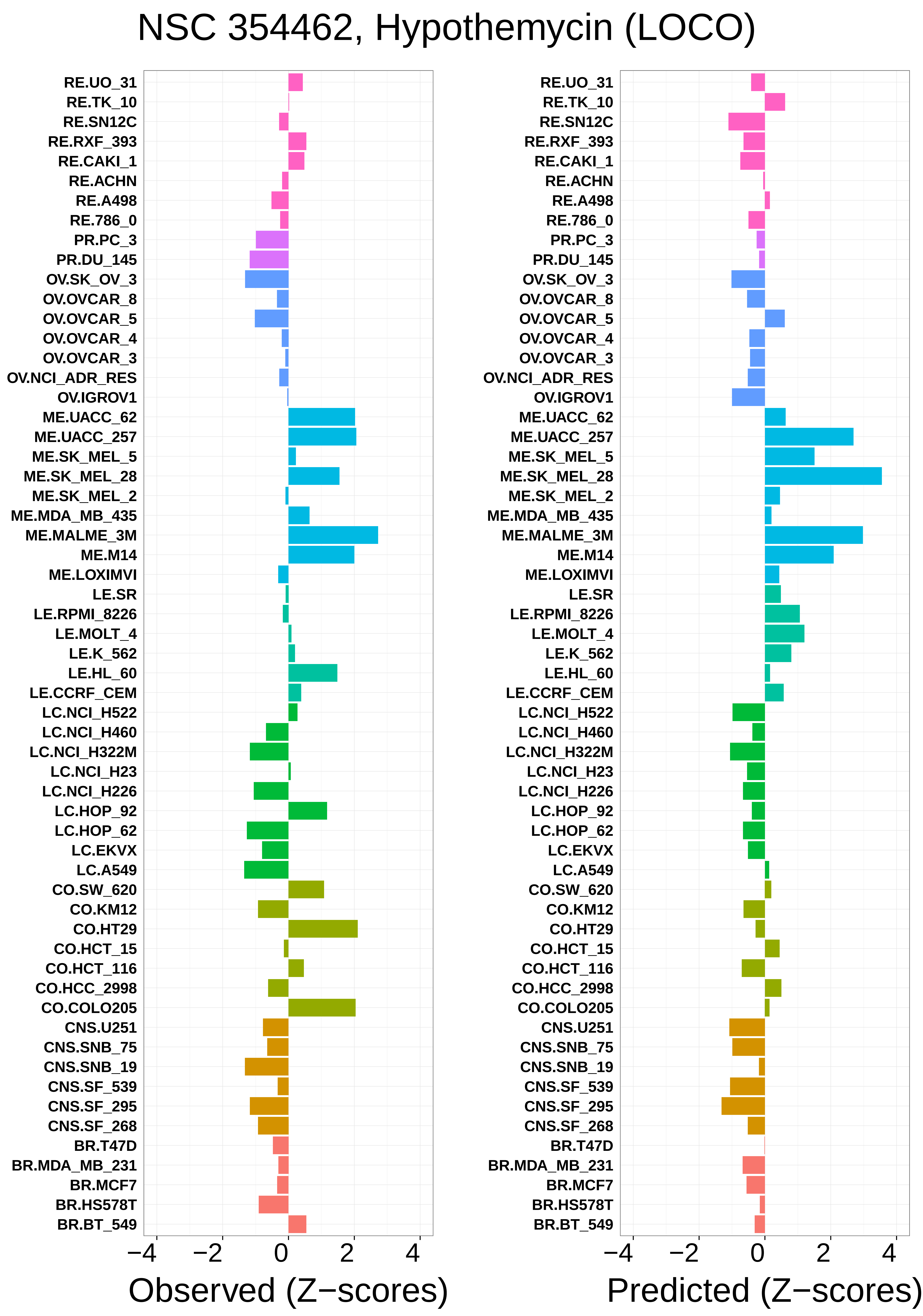
NSC 354462, Hypothemycin (LOCO)
RE.UO_31
RE.TK_10
RE.SN12C
RE.RXF_393
RE.CAKI_1
RE.ACHN
RE.A498
RE.786_0
PR.PC_3
PR.DU_145
OV.SK_OV_3
OV.OVCAR_8
OV.OVCAR_5
OV.OVCAR_4
OV.OVCAR_3
OV.NCI_ADR_RES
OV.IGROV1
ME.UACC_62
ME.UACC_257
ME.SK_MEL_5
ME.SK_MEL_28
ME.SK_MEL_2
ME.MDA_MB_435
ME.MALME_3M
ME.M14
ME.LOXIMVI
LE.SR
LE.RPMI_8226
LE.MOLT_4
LE.K_562
LE.HL_60
LE.CCRF_CEM
LC.NCI_H522
LC.NCI_H460
LC.NCI_H322M
LC.NCI_H23
LC.NCI_H226
LC.HOP_92
LC.HOP_62
LC.EKVX
LC.A549
CO.SW_620
CO.KM12
CO.HT29
CO.HCT_15
CO.HCT_116
CO.HCC_2998
CO.COLO205
CNS.U251
CNS.SNB_75
CNS.SNB_19
CNS.SF_539
CNS.SF_295
CNS.SF_268
BR.T47D
BR.MDA_MB_231
BR.MCF7
BR.HS578T
BR.BT_549
−4 −2 0 2 4
Observed (Z−scores)
Predicted (Z−scores)

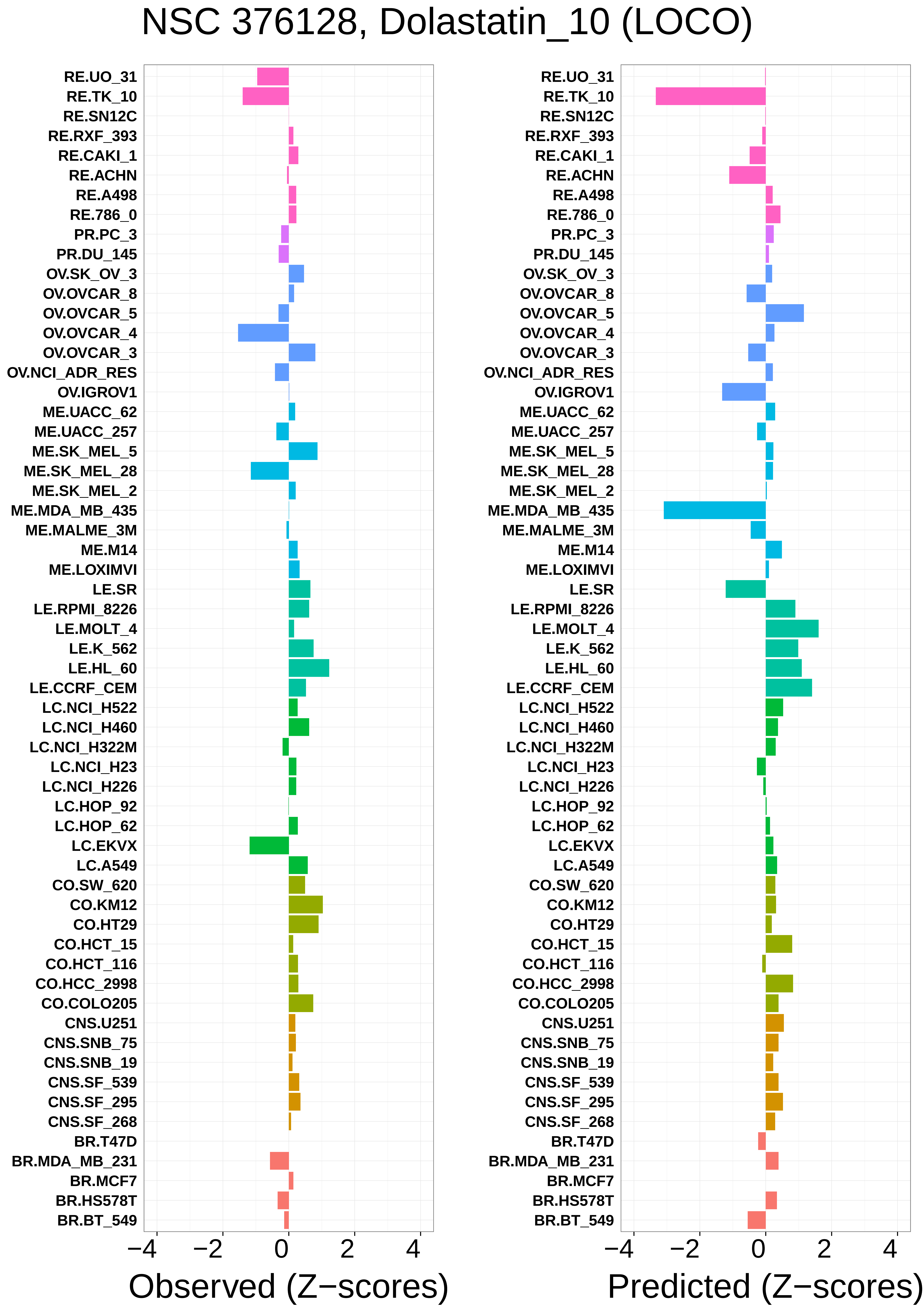
NSC 376128, Dolastatin_10 (LOCO)
RE.UO_31
RE.TK_10
RE.SN12C
RE.RXF_393
RE.CAKI_1
RE.ACHN
RE.A498
RE.786_0
PR.PC_3
PR.DU_145
OV.SK_OV_3
OV.OVCAR_8
OV.OVCAR_5
OV.OVCAR_4
OV.OVCAR_3
OV.NCI_ADR_RES
OV.IGROV1
ME.UACC_62
ME.UACC_257
ME.SK_MEL_5
ME.SK_MEL_28
ME.SK_MEL_2
ME.MDA_MB_435
ME.MALME_3M
ME.M14
ME.LOXIMVI
LE.SR
LE.RPMI_8226
LE.MOLT_4
LE.K_562
LE.HL_60
LE.CCRF_CEM
LC.NCI_H522
LC.NCI_H460
LC.NCI_H322M
LC.NCI_H23
LC.NCI_H226
LC.HOP_92
LC.HOP_62
LC.EKVX
LC.A549
CO.SW_620
CO.KM12
CO.HT29
CO.HCT_15
CO.HCT_116
CO.HCC_2998
CO.COLO205
CNS.U251
CNS.SNB_75
CNS.SNB_19
CNS.SF_539
CNS.SF_295
CNS.SF_268
BR.T47D
BR.MDA_MB_231
BR.MCF7
BR.HS578T
BR.BT_549
−4 −2 0 2 4
Observed (Z−scores)
Predicted (Z−scores)

NSC 606869, (LOCO)

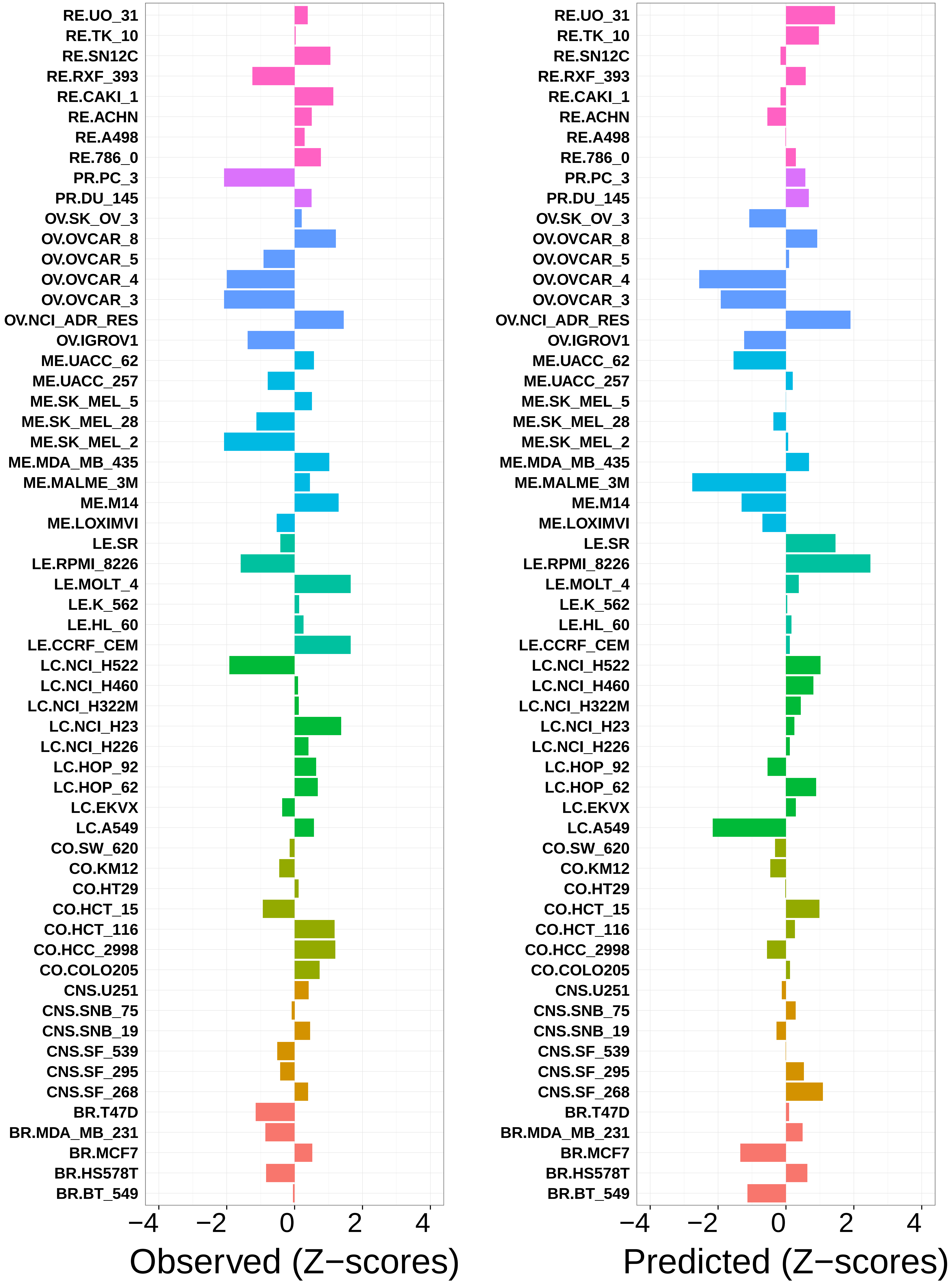

NSC 609699, Topotecan_Hydrochloride (LOCO)

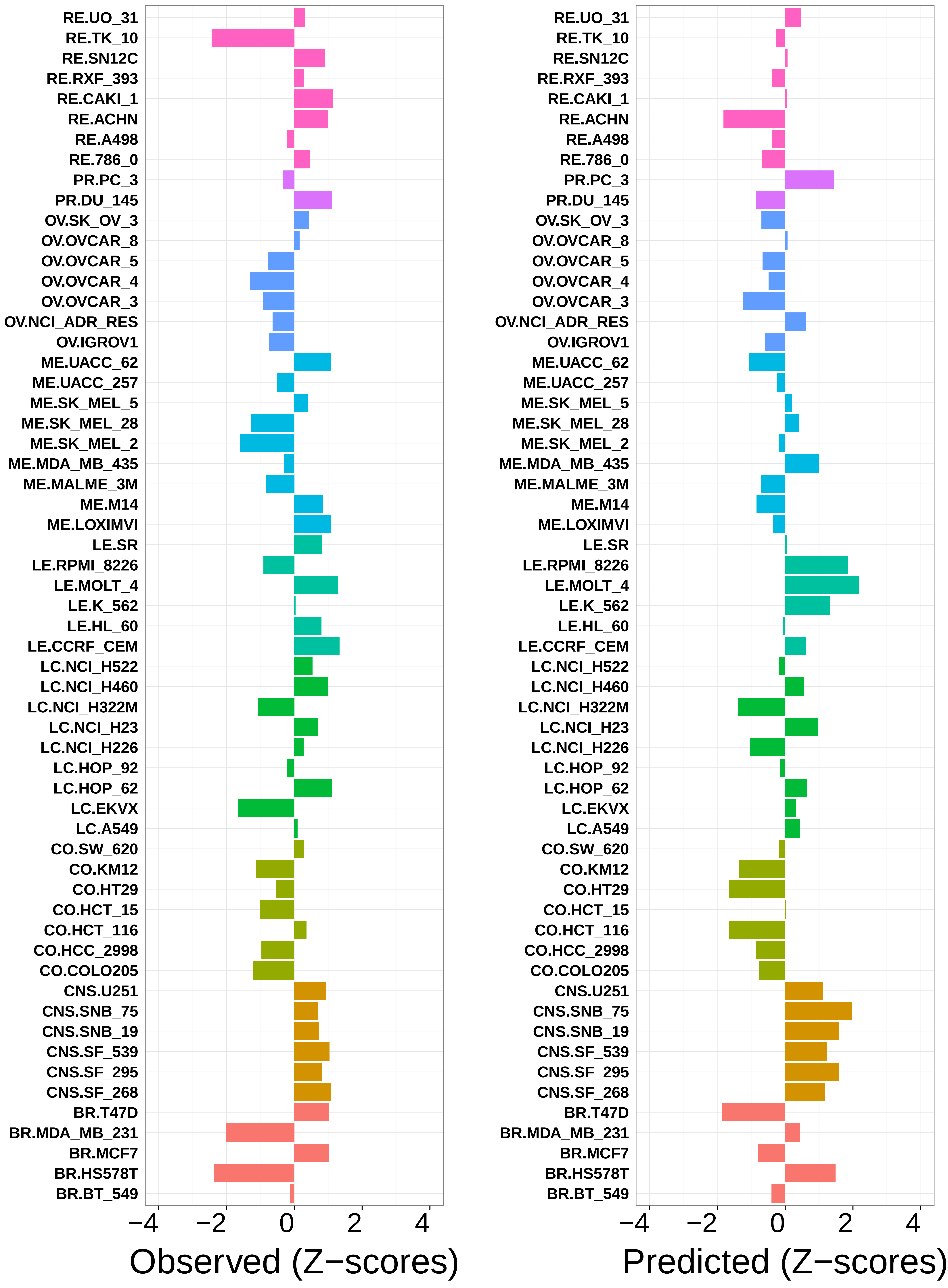

NSC 613327, (LOCO)

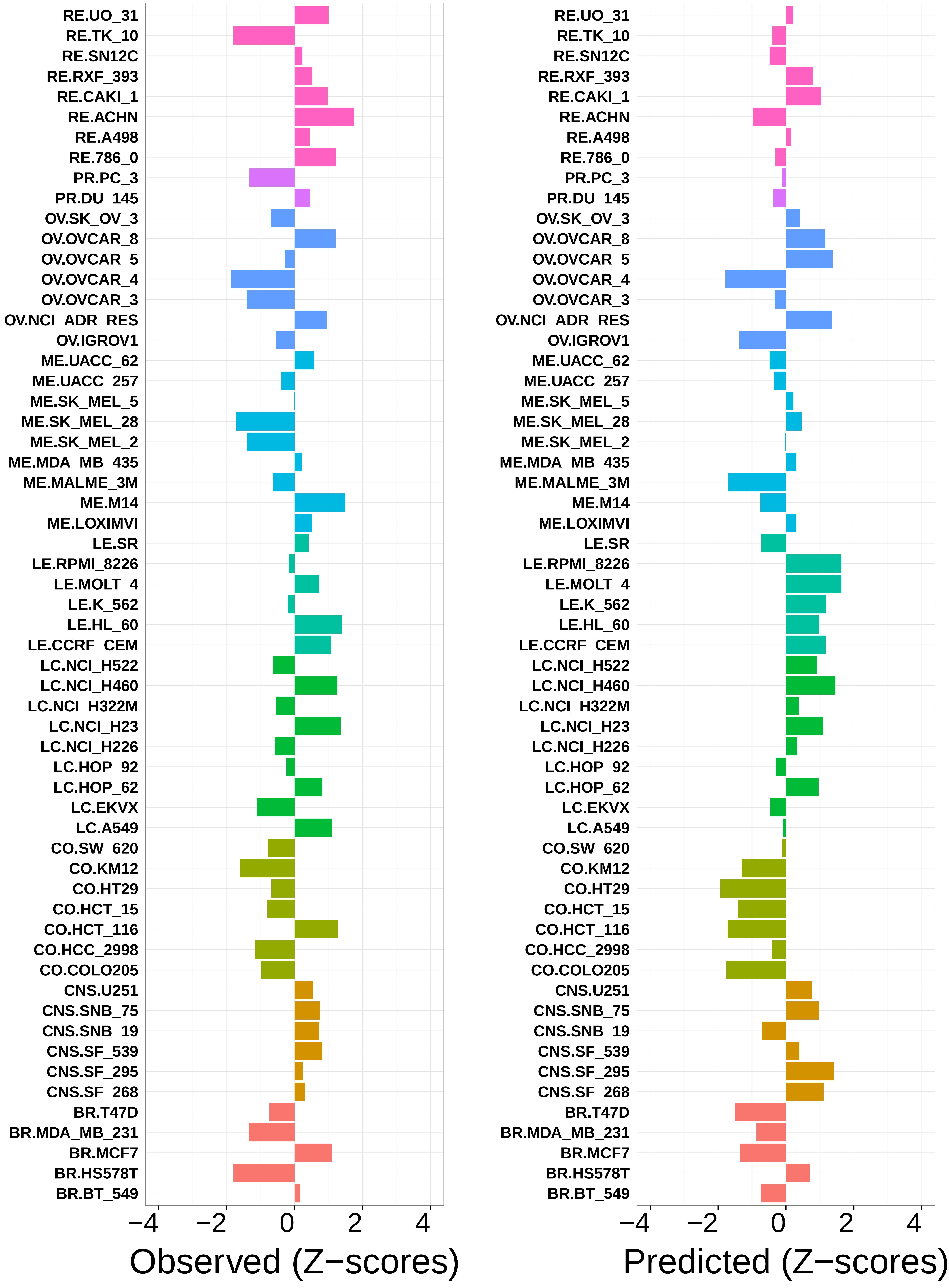

NSC 628503, Docetaxel (LOCO)

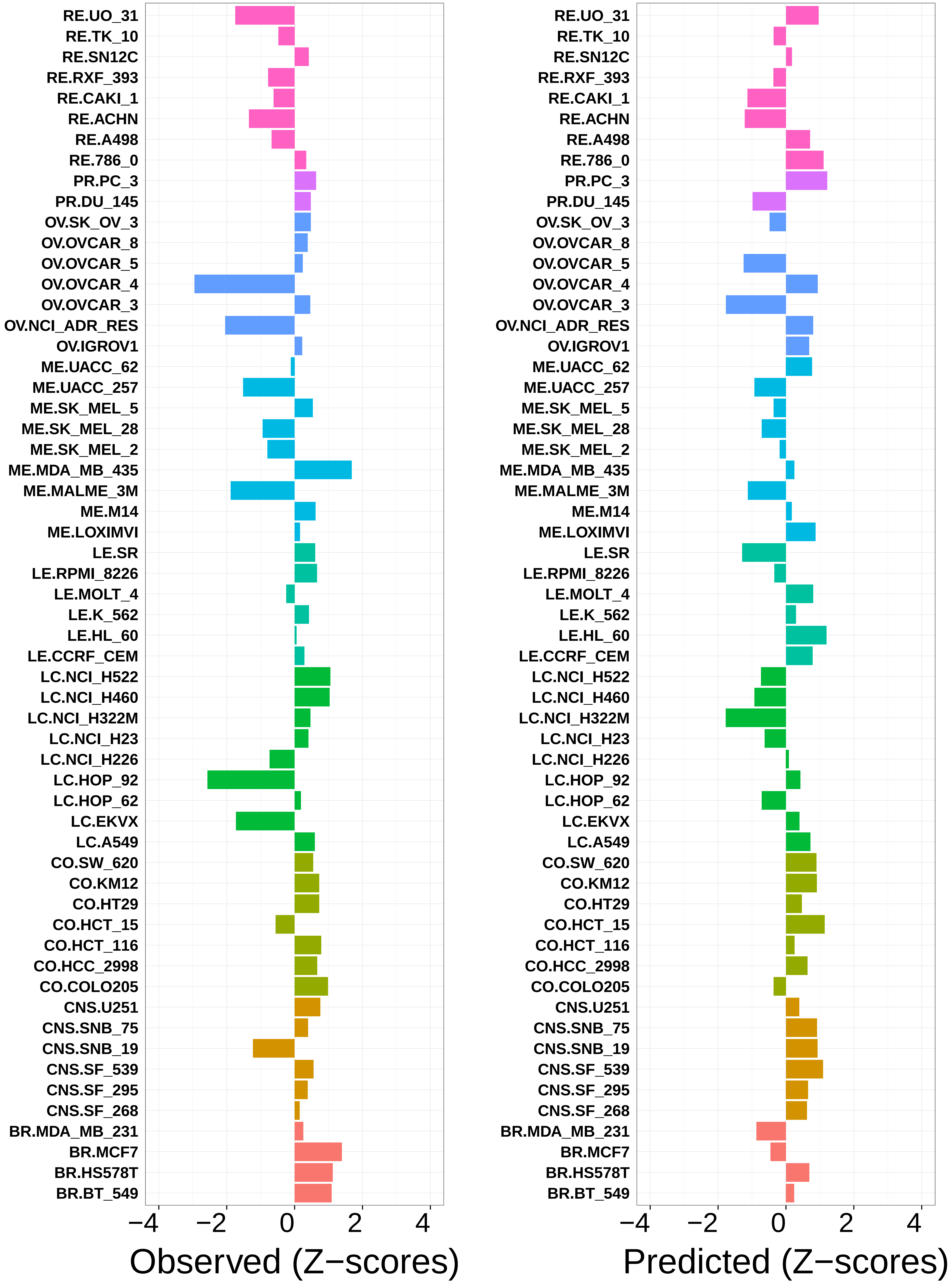

NSC 630176, Romidepsin (LOCO)

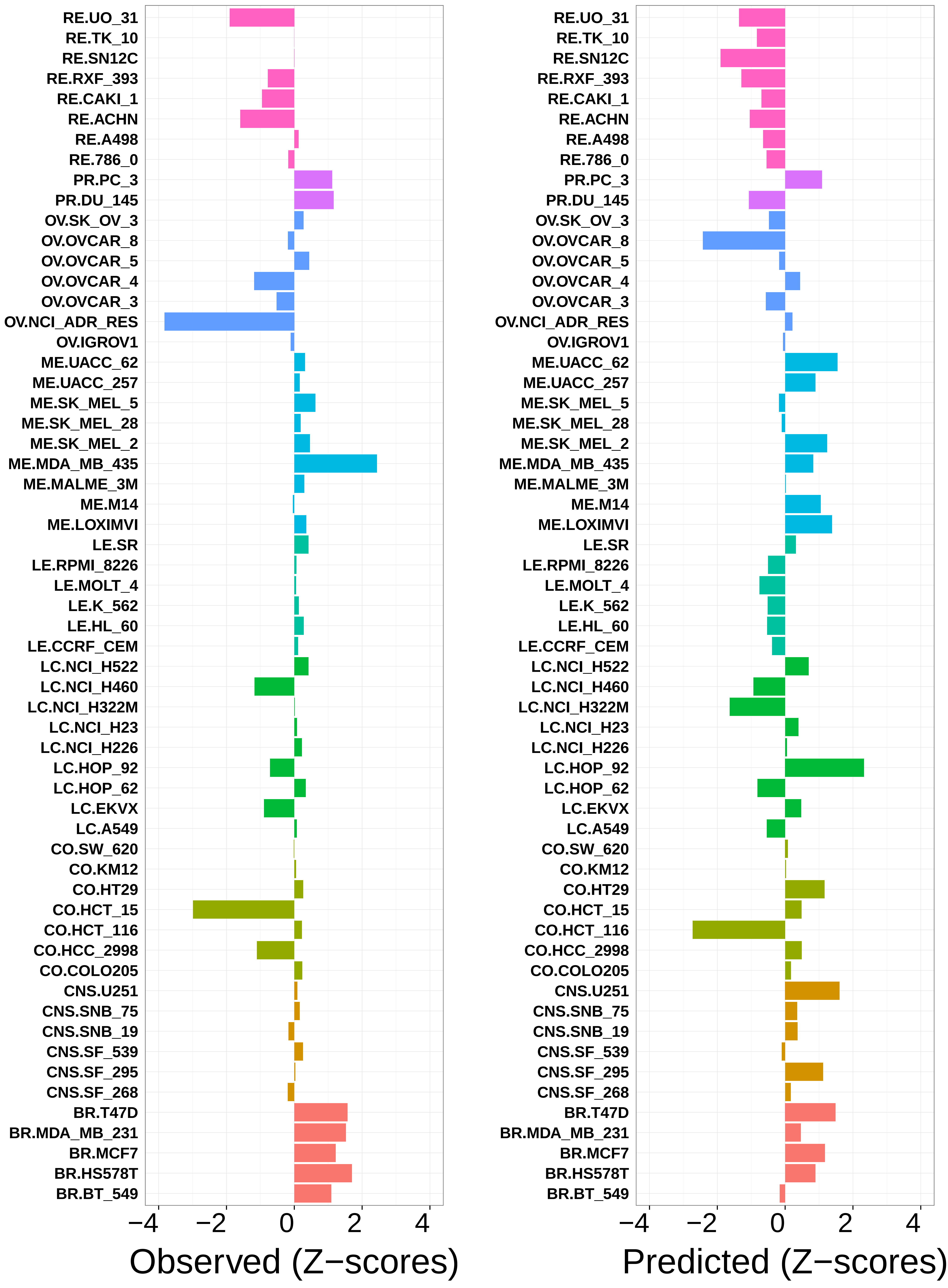

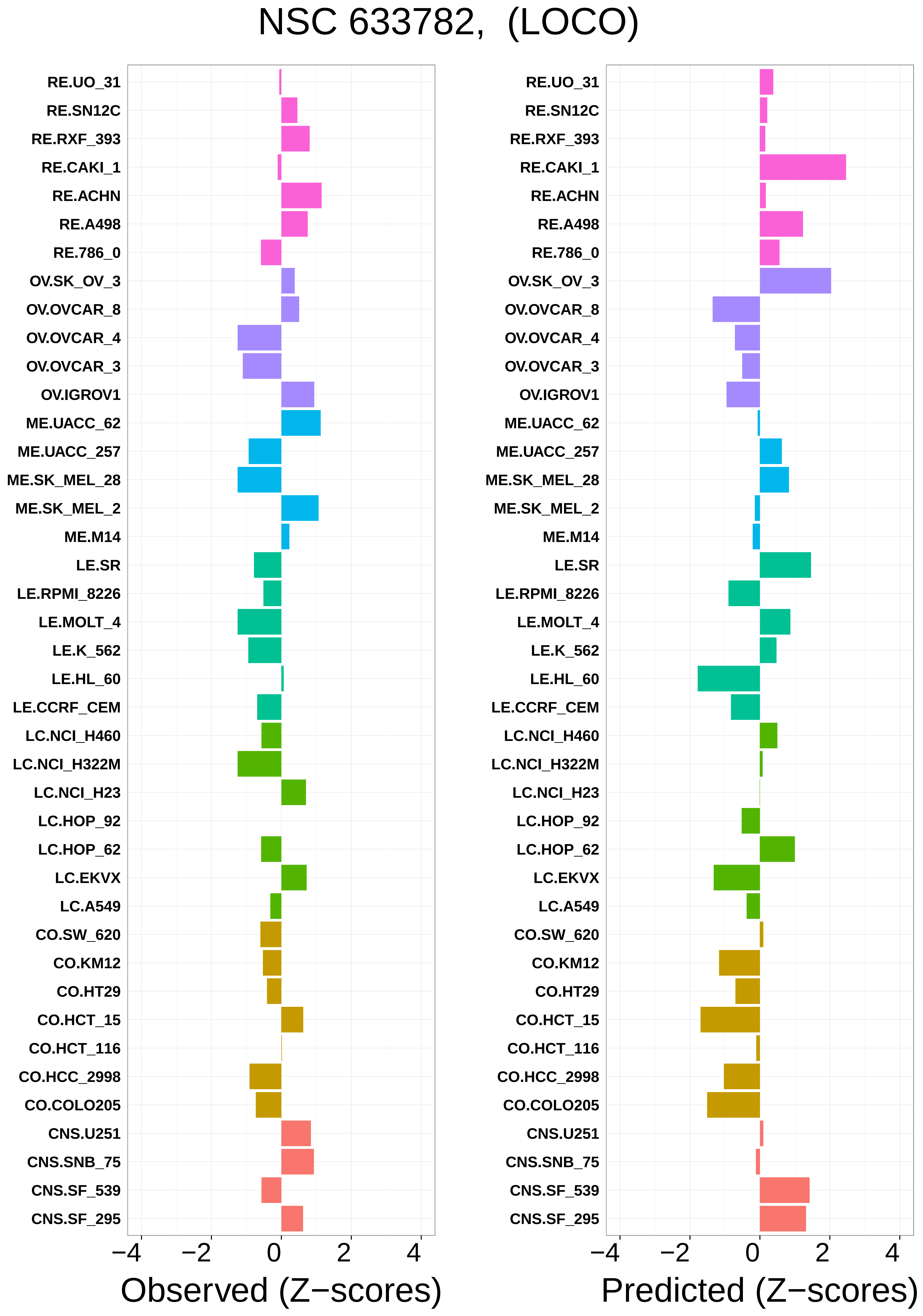
NSC 633782, (LOCO)
RE.UO_31
RE.SN12C
RE.RXF_393
RE.CAKI_1
RE.ACHN
RE.A498
RE.786_0
OV.SK_OV_3
OV.OVCAR_8
OV.OVCAR_4
OV.OVCAR_3
OV.IGROV1
ME.UACC_62
ME.UACC_257
ME.SK_MEL_28
ME.SK_MEL_2
ME.M14
LE.SR
LE.RPMI_8226
LE.MOLT_4
LE.K_562
LE.HL_60
LE.CCRF_CEM
LC.NCI_H460
LC.NCI_H322M
LC.NCI_H23
LC.HOP_92
LC.HOP_62
LC.EKVX
LC.A549
CO.SW_620
CO.KM12
CO.HT29
CO.HCT_15
CO.HCT_116
CO.HCC_2998
CO.COLO205
CNS.U251
CNS.SNB_75
CNS.SF_539
CNS.SF_295
−4 −2 0 2 4
Observed (Z−scores)
Predicted (Z−scores)

NSC 63878, Cytarabine_Hydrochloride (LOCO)

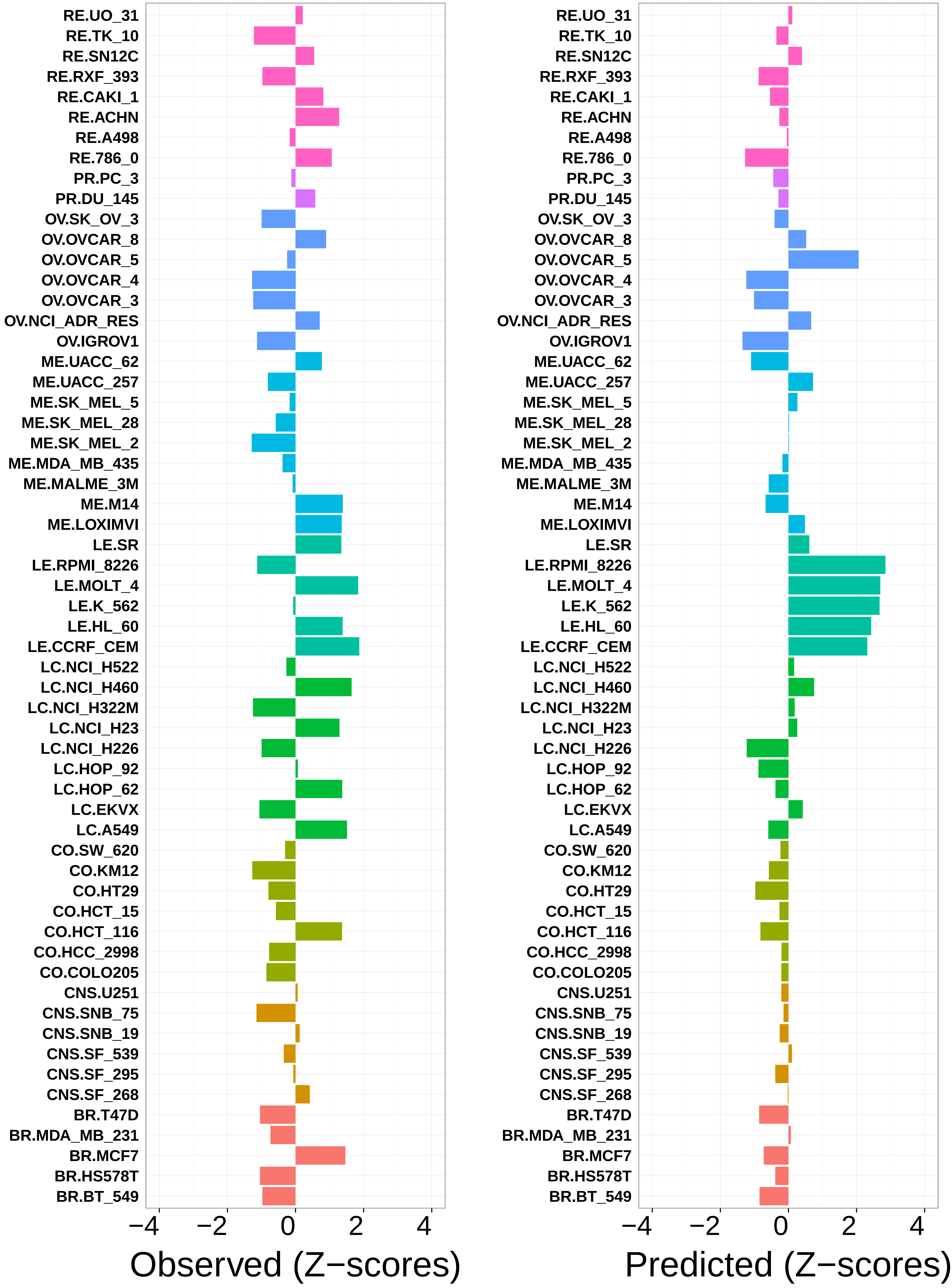

NSC 639186, (LOCO)

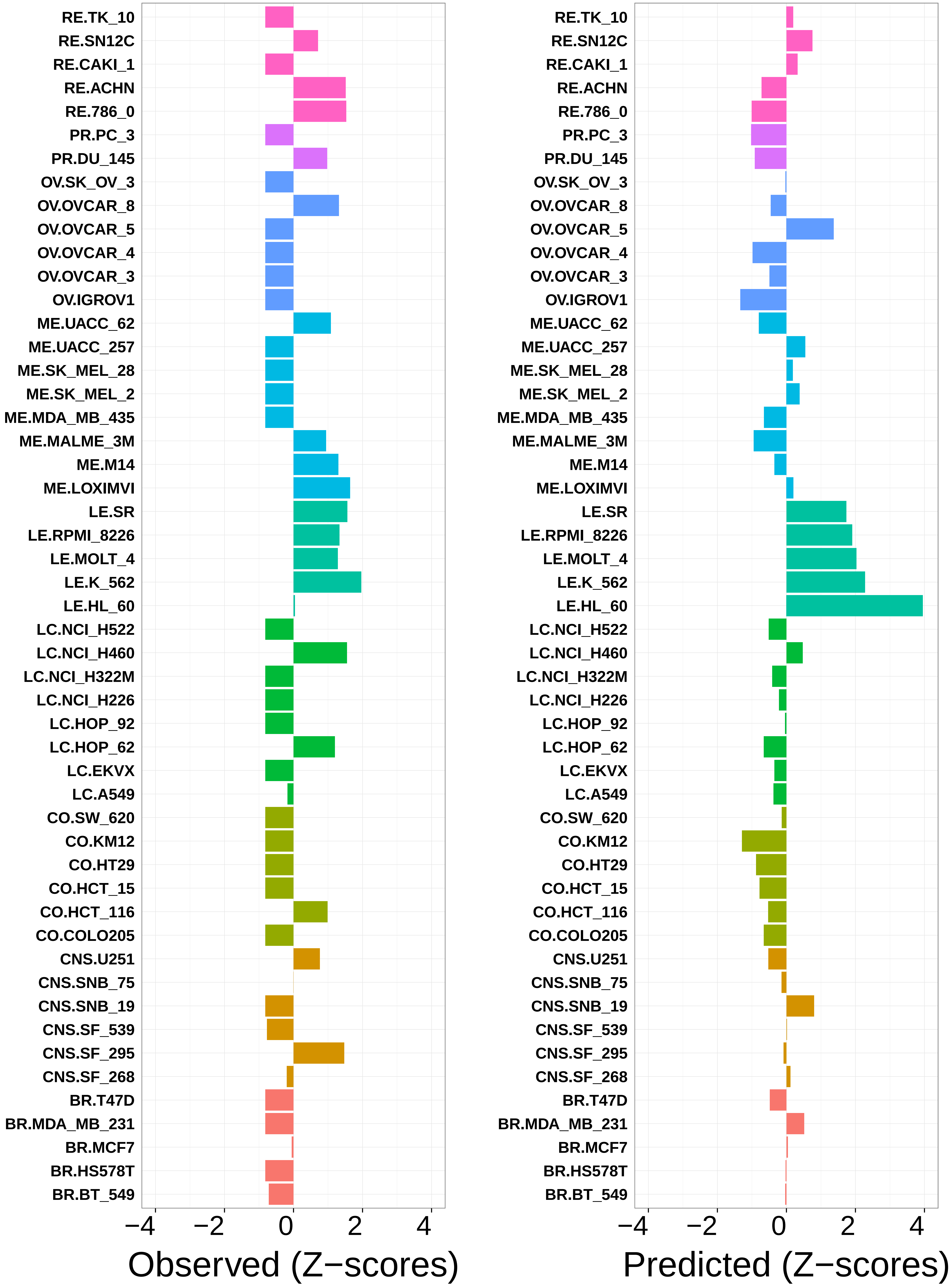

NSC 656576, (LOCO)

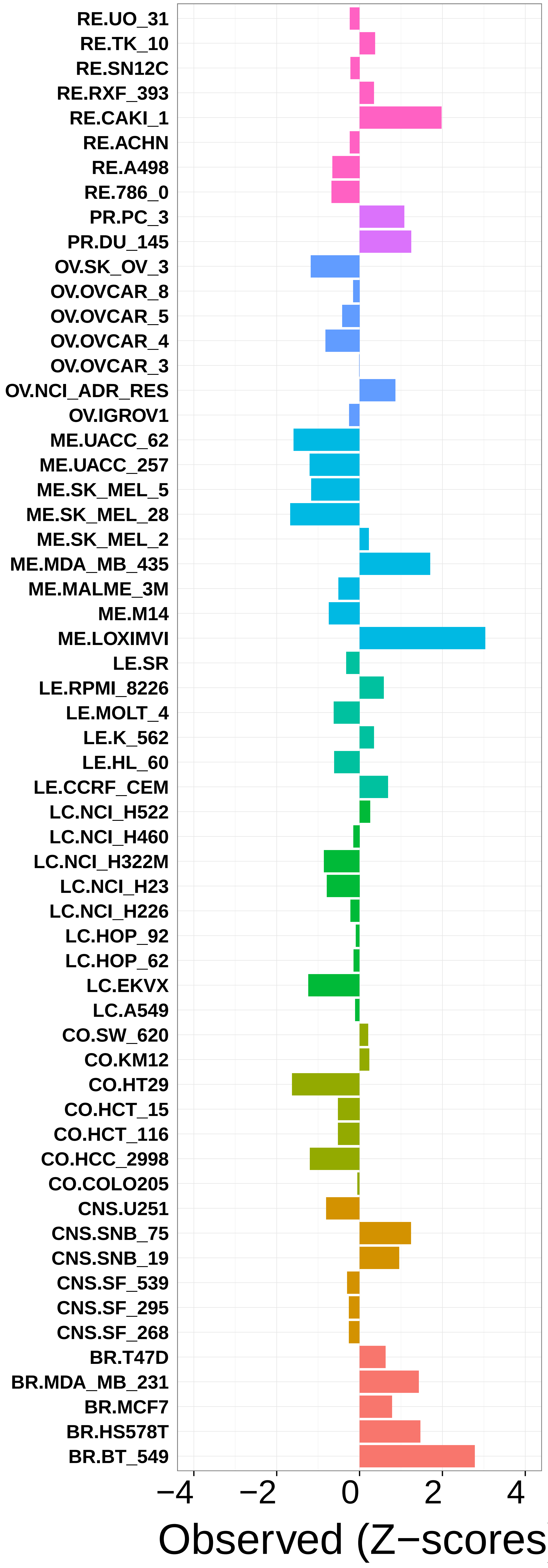

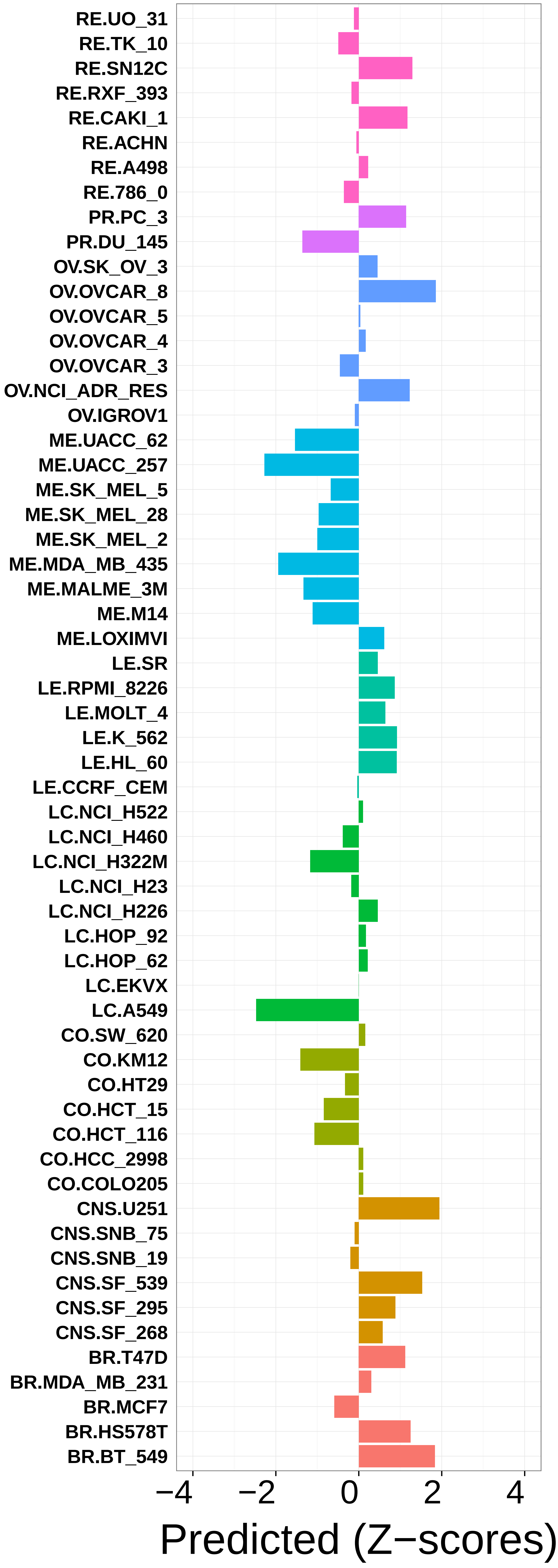

NSC 673596, (LOCO)

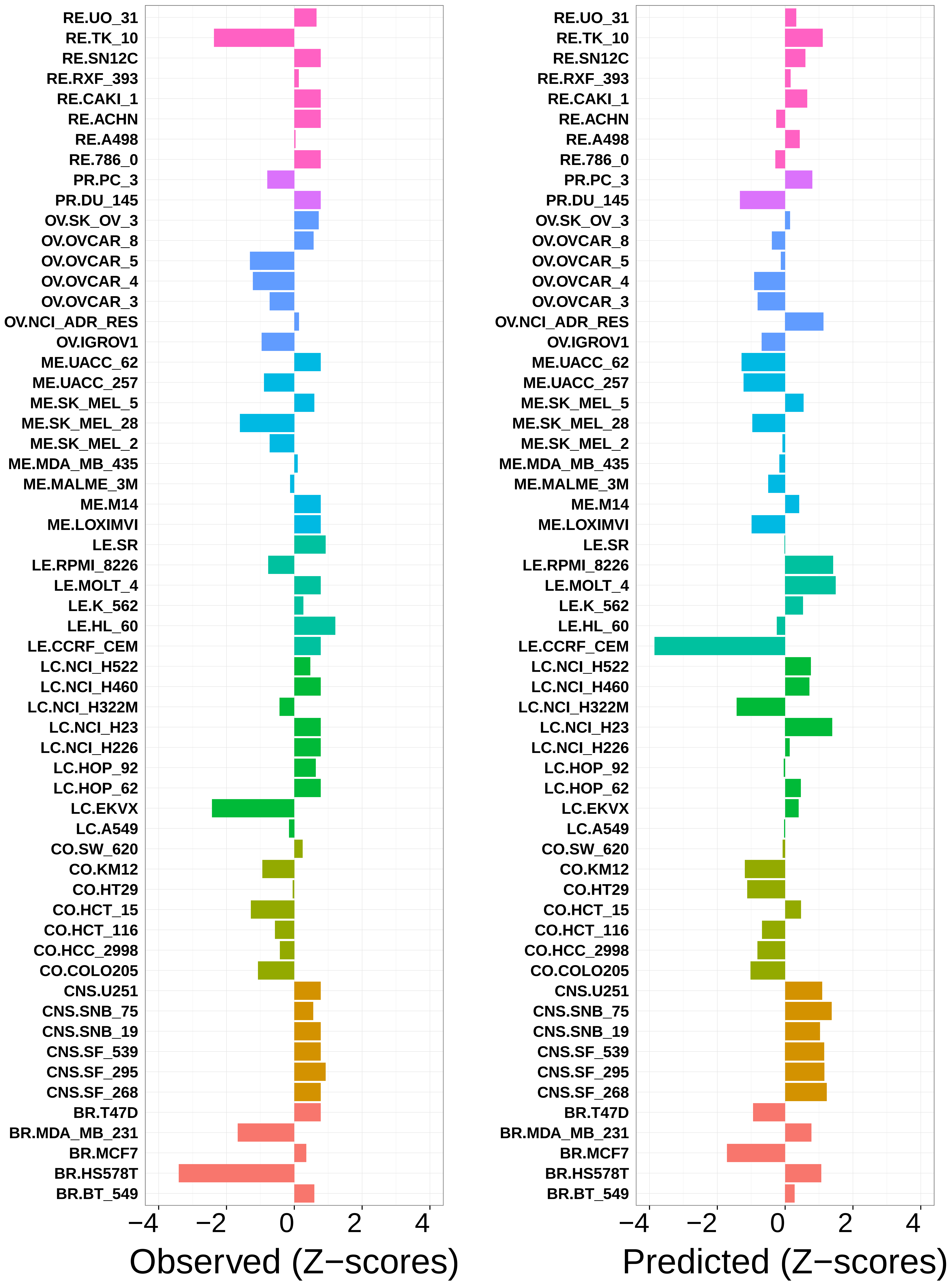

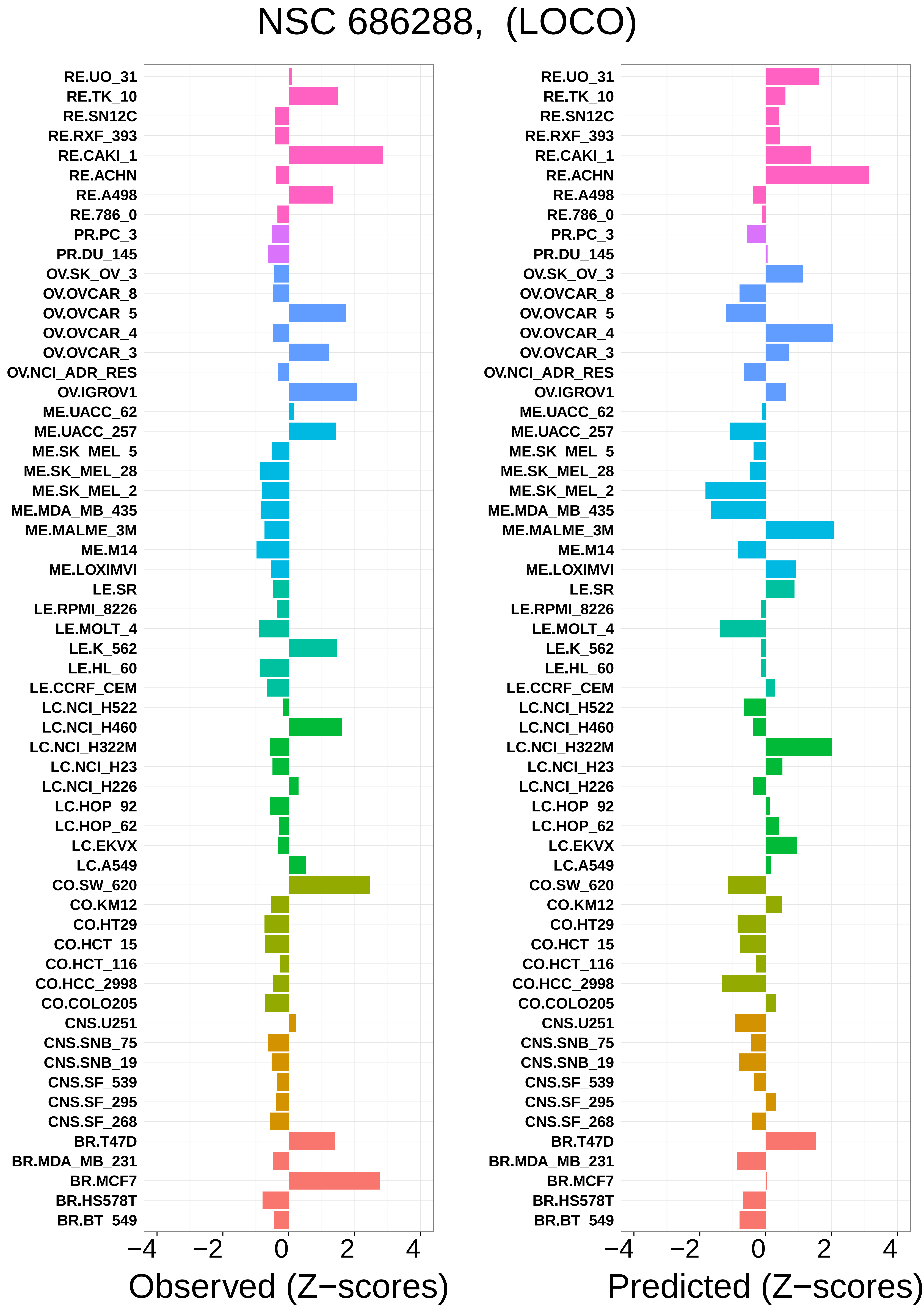

NSC 686288, (LOCO)
RE.UO_31
RE.TK_10
RE.SN12C
RE.RXF_393
RE.CAKI_1
RE.ACHN
RE.A498
RE.786_0
PR.PC_3
PR.DU_145
OV.SK_OV_3
OV.OVCAR_8
OV.OVCAR_5
OV.OVCAR_4
OV.OVCAR_3
OV.NCI_ADR_RES
OV.IGROV1
ME.UACC_62
ME.UACC_257
ME.SK_MEL_5
ME.SK_MEL_28
ME.SK_MEL_2
ME.MDA_MB_435
ME.MALME_3M
ME.M14
ME.LOXIMVI
LE.SR
LE.RPMI_8226
LE.MOLT_4
LE.K_562
LE.HL_60
LE.CCRF_CEM
LC.NCI_H522
LC.NCI_H460
LC.NCI_H322M
LC.NCI_H23
LC.NCI_H226
LC.HOP_92
LC.HOP_62
LC.EKVX
LC.A549
CO.SW_620
CO.KM12
CO.HT29
CO.HCT_15
CO.HCT_116
CO.HCC_2998
CO.COLO205
CNS.U251
CNS.SNB_75
CNS.SNB_19
CNS.SF_539
CNS.SF_295
CNS.SF_268
BR.T47D
BR.MDA_MB_231
BR.MCF7
BR.HS578T
BR.BT_549
−4 −2 0 2 4
Observed (Z−scores)
Predicted (Z−scores)

NSC 698037, (LOCO)

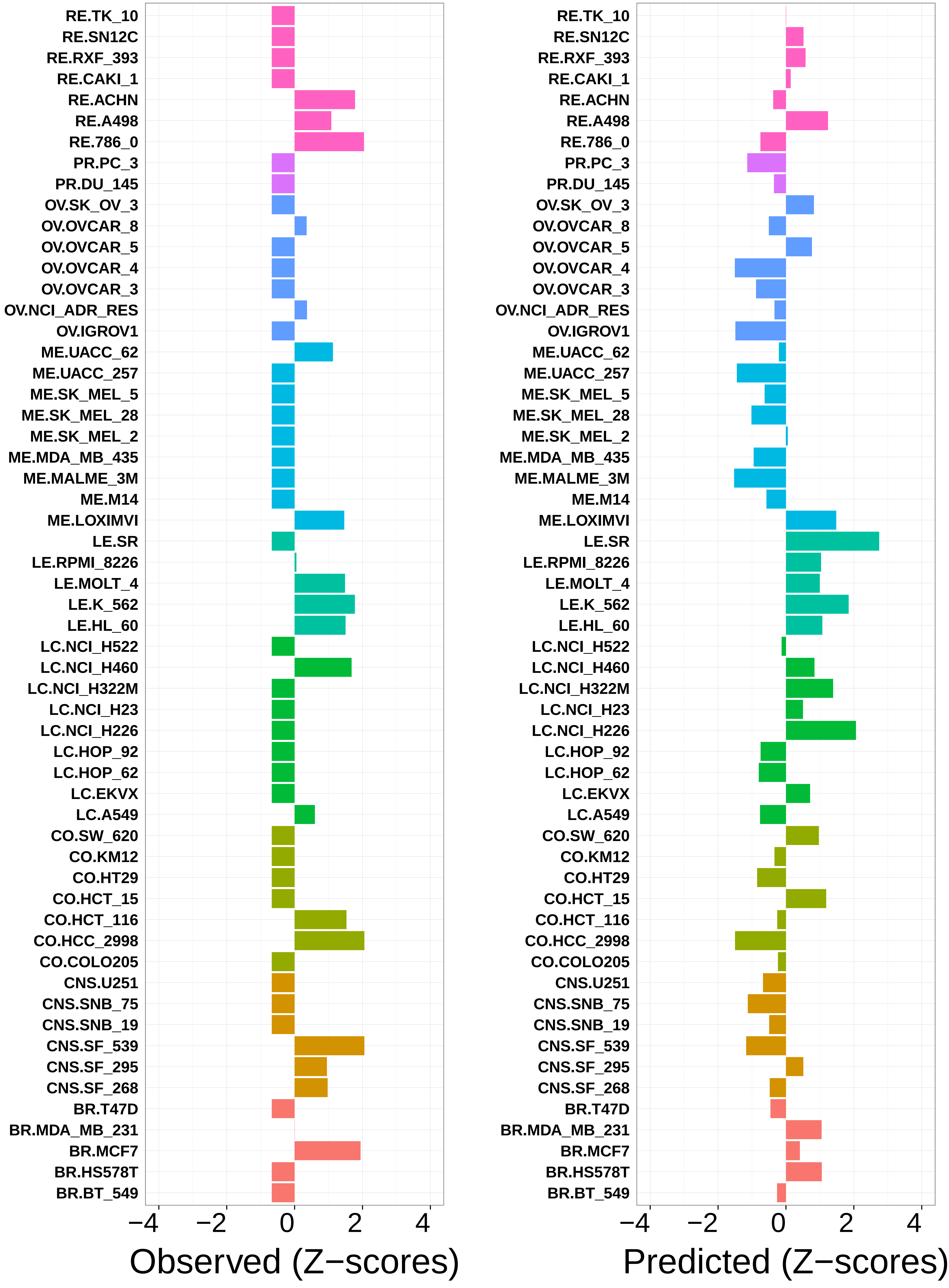

NSC 707545, Alvespimycin (LOCO)

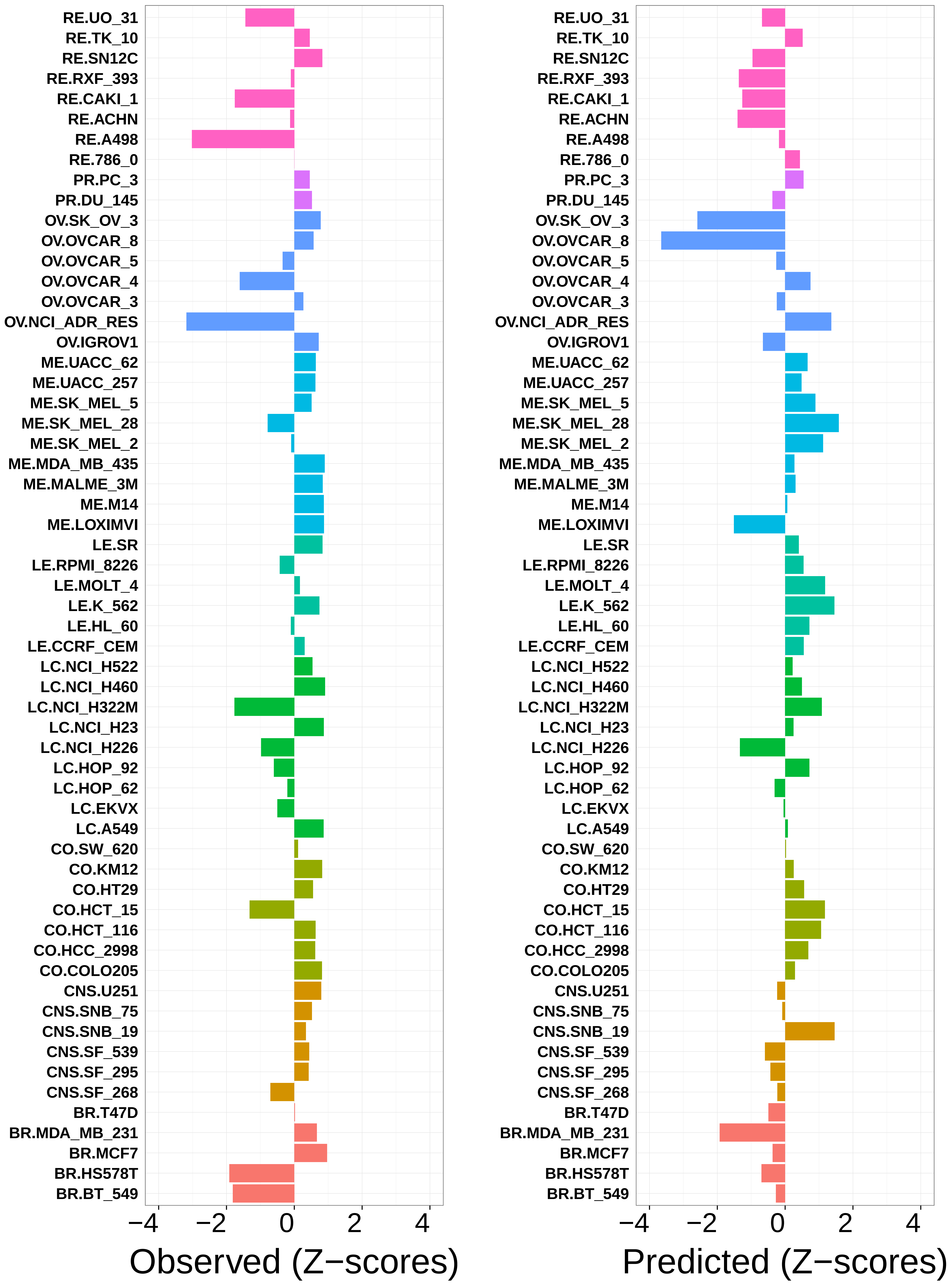

NSC 710297, OMDPI (LOCO)

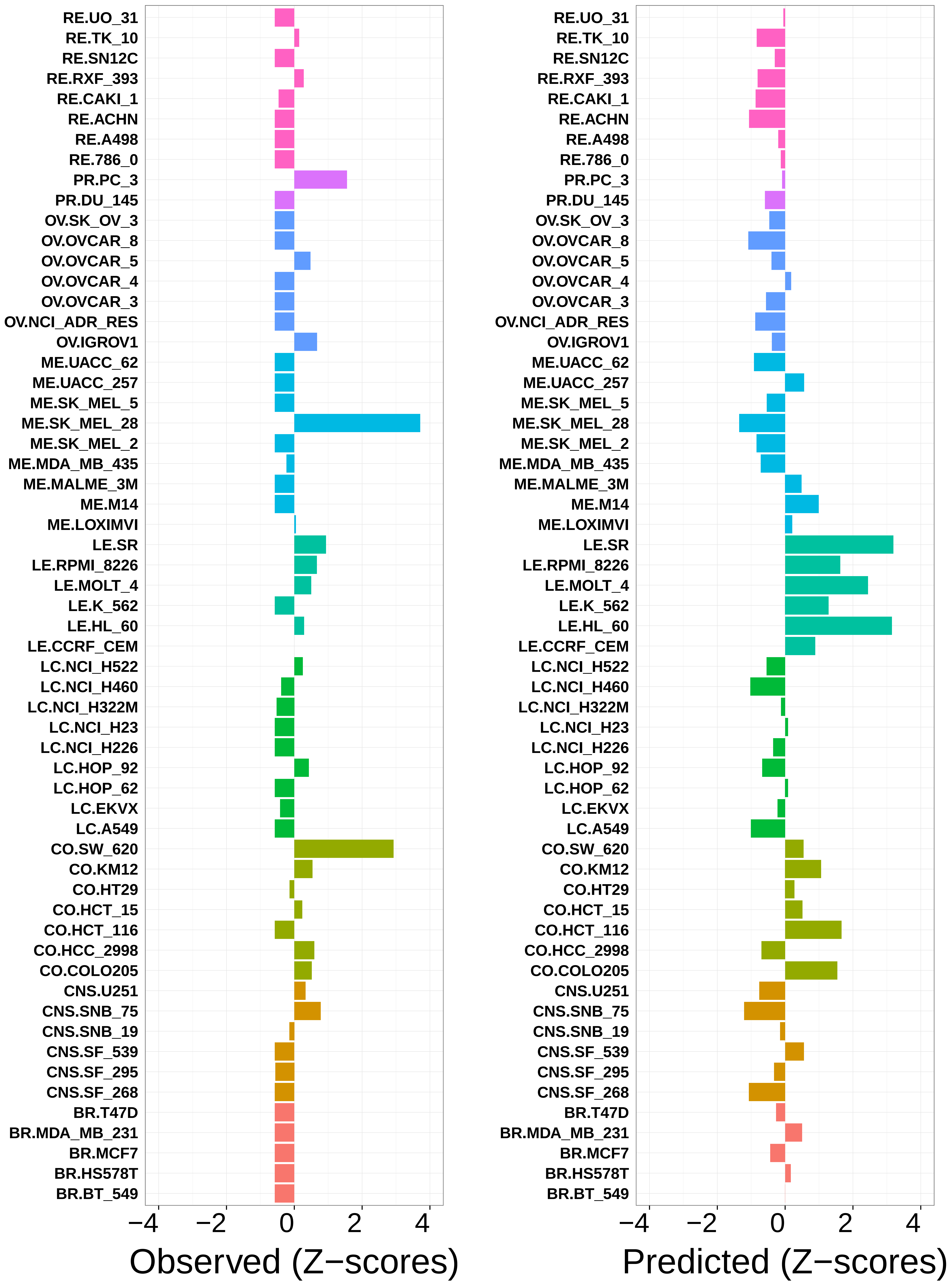

NSC 710464, (LOCO)

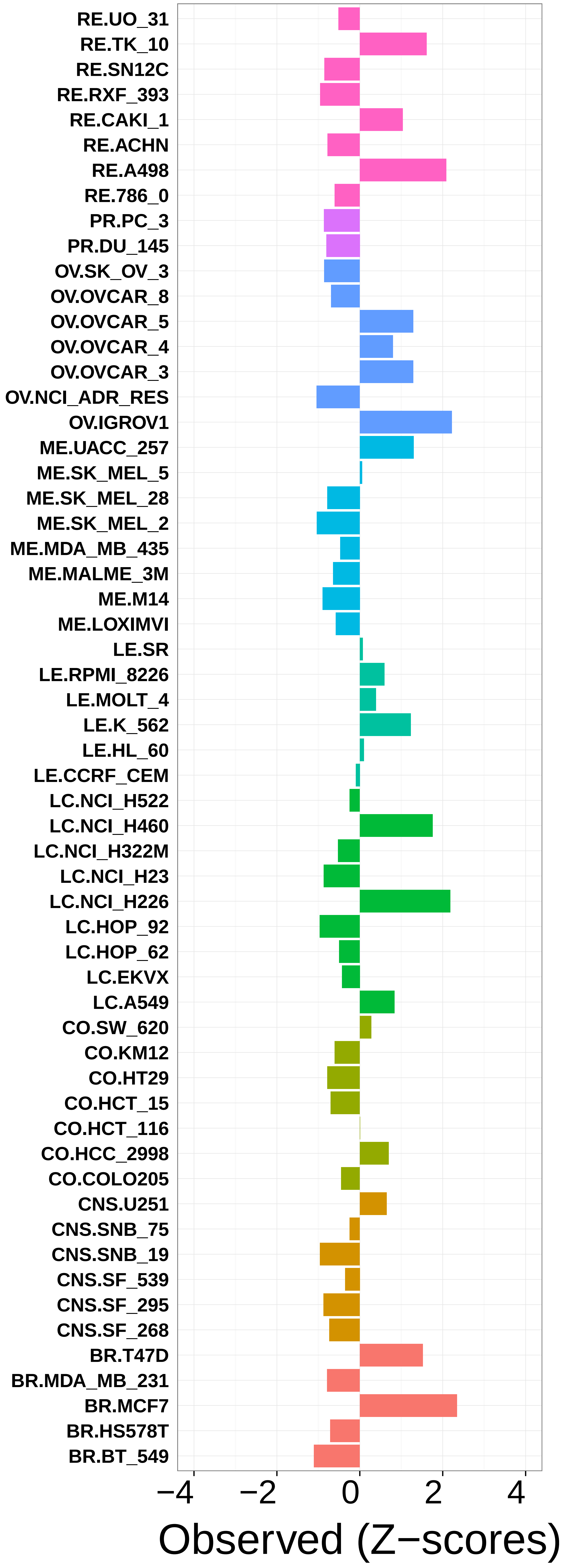

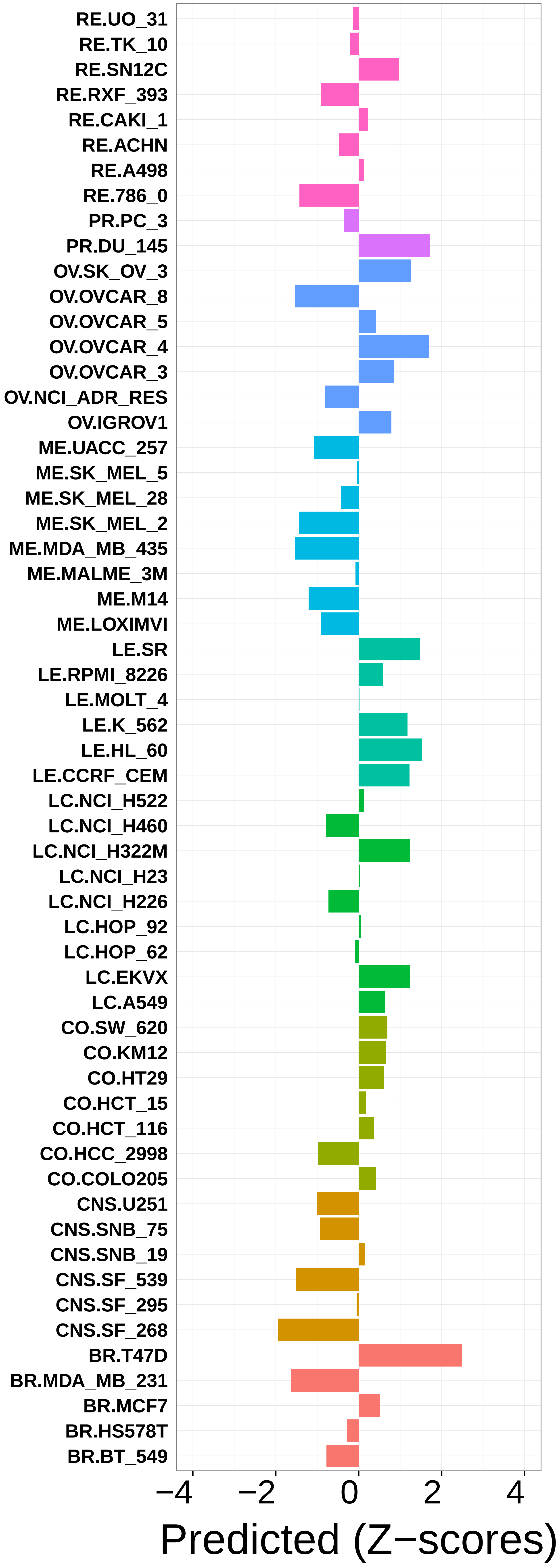

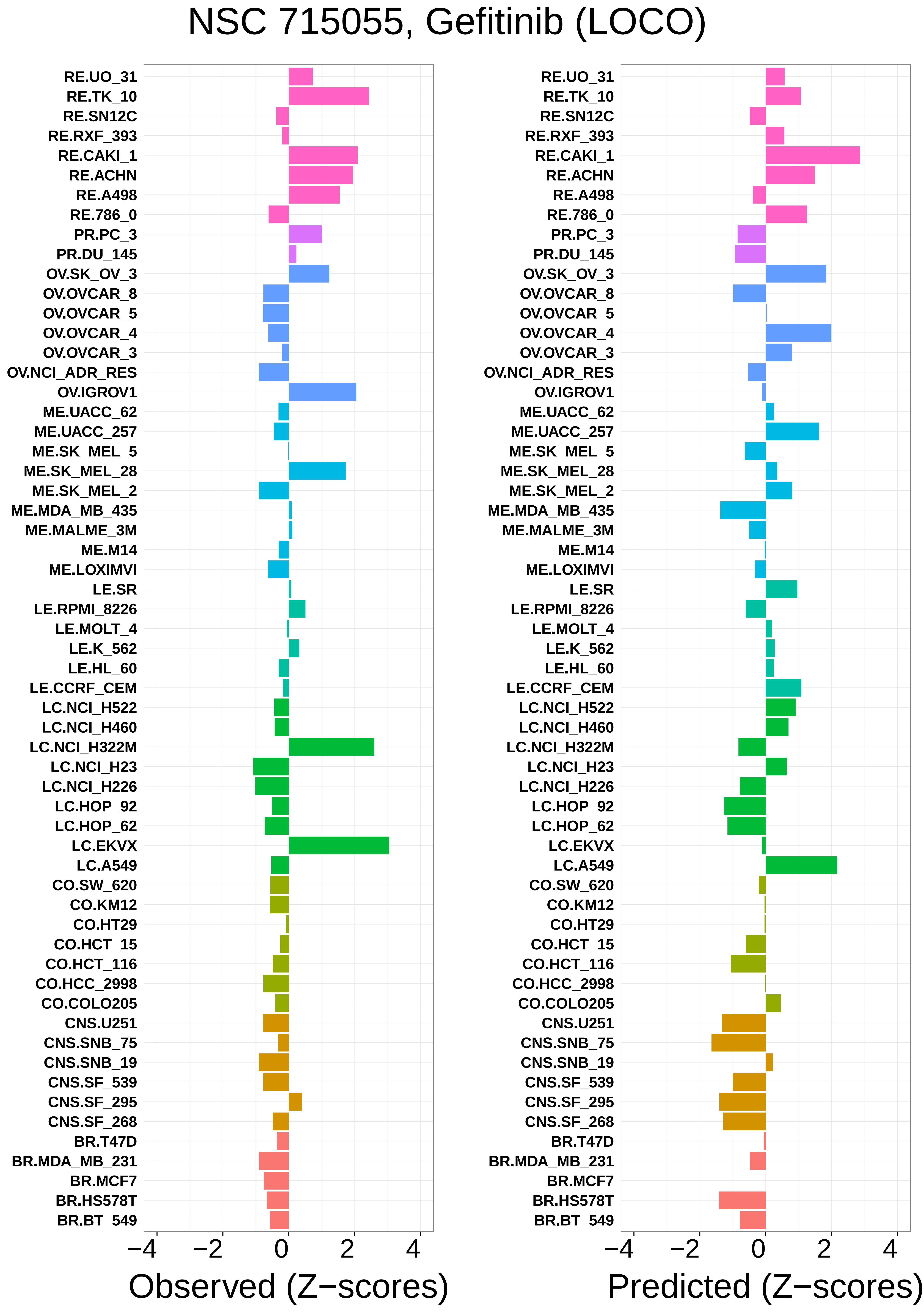

NSC 715055, Gefitinib (LOCO)
RE.UO_31
RE.TK_10
RE.SN12C
RE.RXF_393
RE.CAKI_1
RE.ACHN
RE.A498
RE.786_0
PR.PC_3
PR.DU_145
OV.SK_OV_3
OV.OVCAR_8
OV.OVCAR_5
OV.OVCAR_4
OV.OVCAR_3
OV.NCI_ADR_RES
OV.IGROV1
ME.UACC_62
ME.UACC_257
ME.SK_MEL_5
ME.SK_MEL_28
ME.SK_MEL_2
ME.MDA_MB_435
ME.MALME_3M
ME.M14
ME.LOXIMVI
LE.SR
LE.RPMI_8226
LE.MOLT_4
LE.K_562
LE.HL_60
LE.CCRF_CEM
LC.NCI_H522
LC.NCI_H460
LC.NCI_H322M
LC.NCI_H23
LC.NCI_H226
LC.HOP_92
LC.HOP_62
LC.EKVX
LC.A549
CO.SW_620
CO.KM12
CO.HT29
CO.HCT_15
CO.HCT_116
CO.HCC_2998
CO.COLO205
CNS.U251
CNS.SNB_75
CNS.SNB_19
CNS.SF_539
CNS.SF_295
CNS.SF_268
BR.T47D
BR.MDA_MB_231
BR.MCF7
BR.HS578T
BR.BT_549
−4
−2
0
2
4
Observed (Z−scores)
Predicted (Z−scores)

NSC 718781, (LOCO)

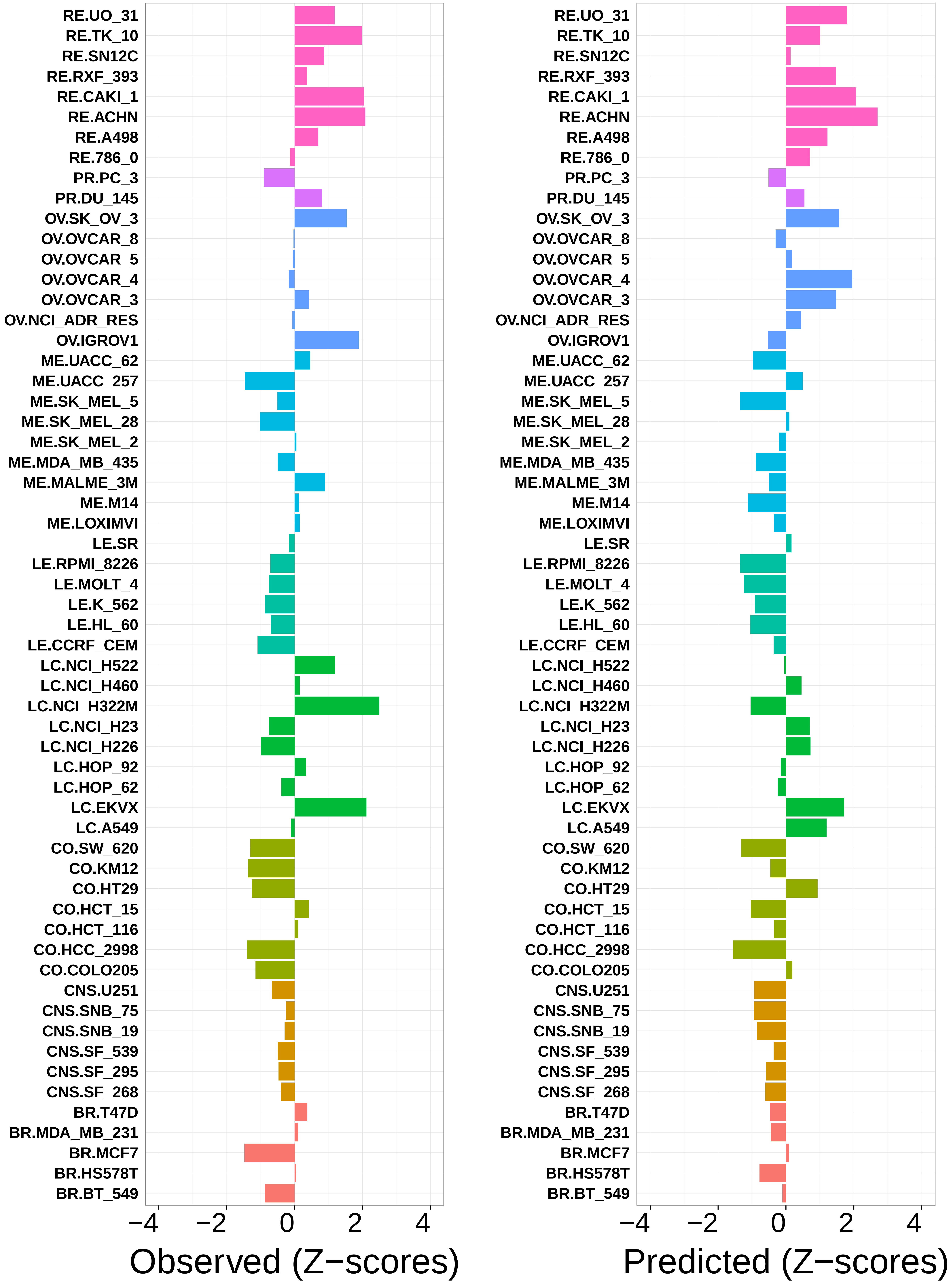

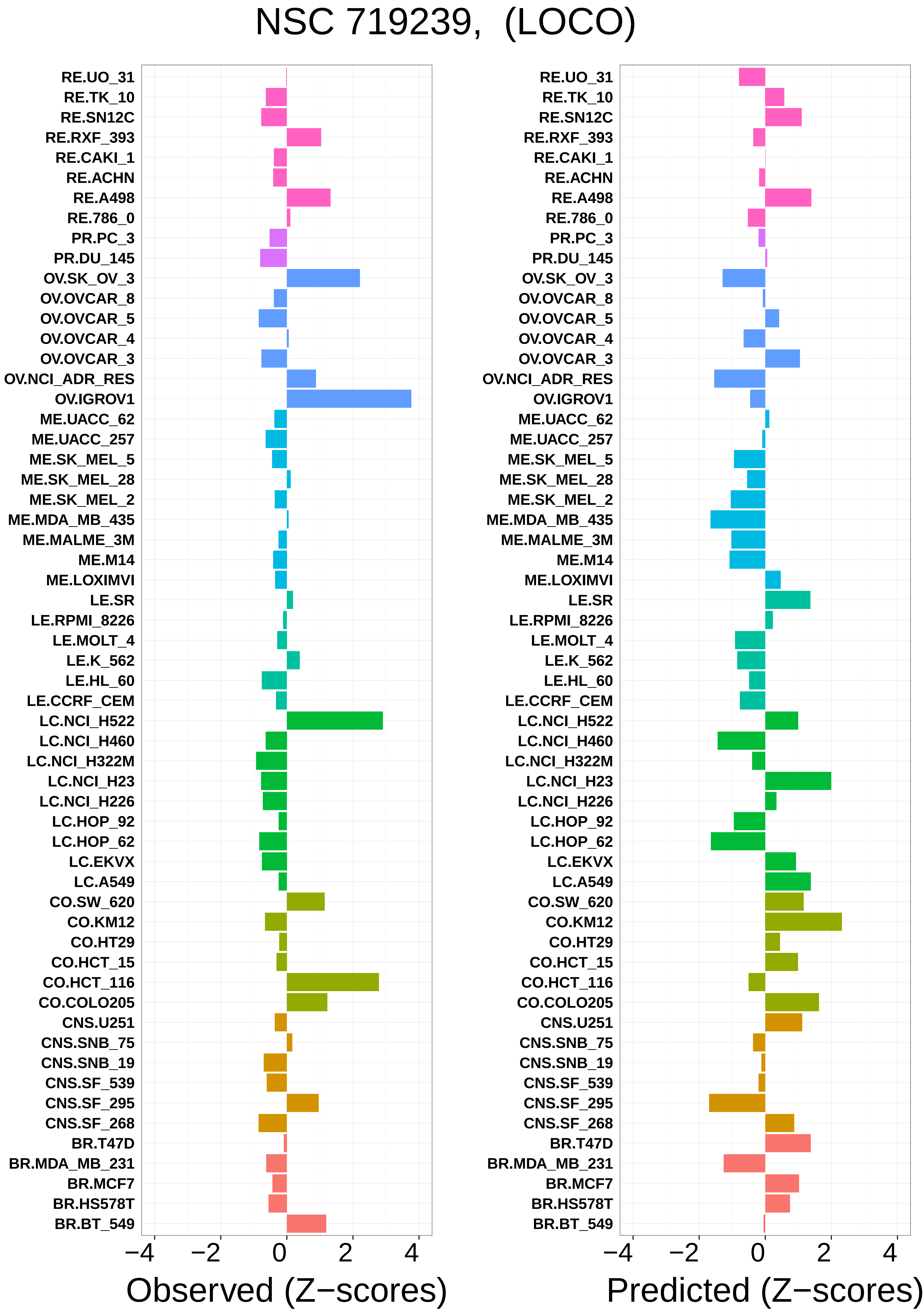
NSC 719239, (LOCO)
RE.UO_31
RE.TK_10
RE.SN12C
RE.RXF_393
RE.CAKI_1
RE.ACHN
RE.A498
RE.786_0
PR.PC_3
PR.DU_145
OV.SK_OV_3
OV.OVCAR_8
OV.OVCAR_5
OV.OVCAR_4
OV.OVCAR_3
OV.NCI_ADR_RES
OV.IGROV1
ME.UACC_62
ME.UACC_257
ME.SK_MEL_5
ME.SK_MEL_28
ME.SK_MEL_2
ME.MDA_MB_435
ME.MALME_3M
ME.M14
ME.LOXIMVI
LE.SR
LE.RPMI_8226
LE.MOLT_4
LE.K_562
LE.HL_60
LE.CCRF_CEM
LC.NCI_H522
LC.NCI_H460
LC.NCI_H322M
LC.NCI_H23
LC.NCI_H226
LC.HOP_92
LC.HOP_62
LC.EKVX
LC.A549
CO.SW_620
CO.KM12
CO.HT29
CO.HCT_15
CO.HCT_116
CO.COLO205
CNS.U251
CNS.SNB_75
CNS.SNB_19
CNS.SF_539
CNS.SF_295
CNS.SF_268
BR.T47D
BR.MDA_MB_231
BR.MCF7
BR.HS578T
BR.BT_549
−4
−2
0
2
4
Observed (Z−scores)
Predicted (Z−scores)

NSC 724998, (LOCO)

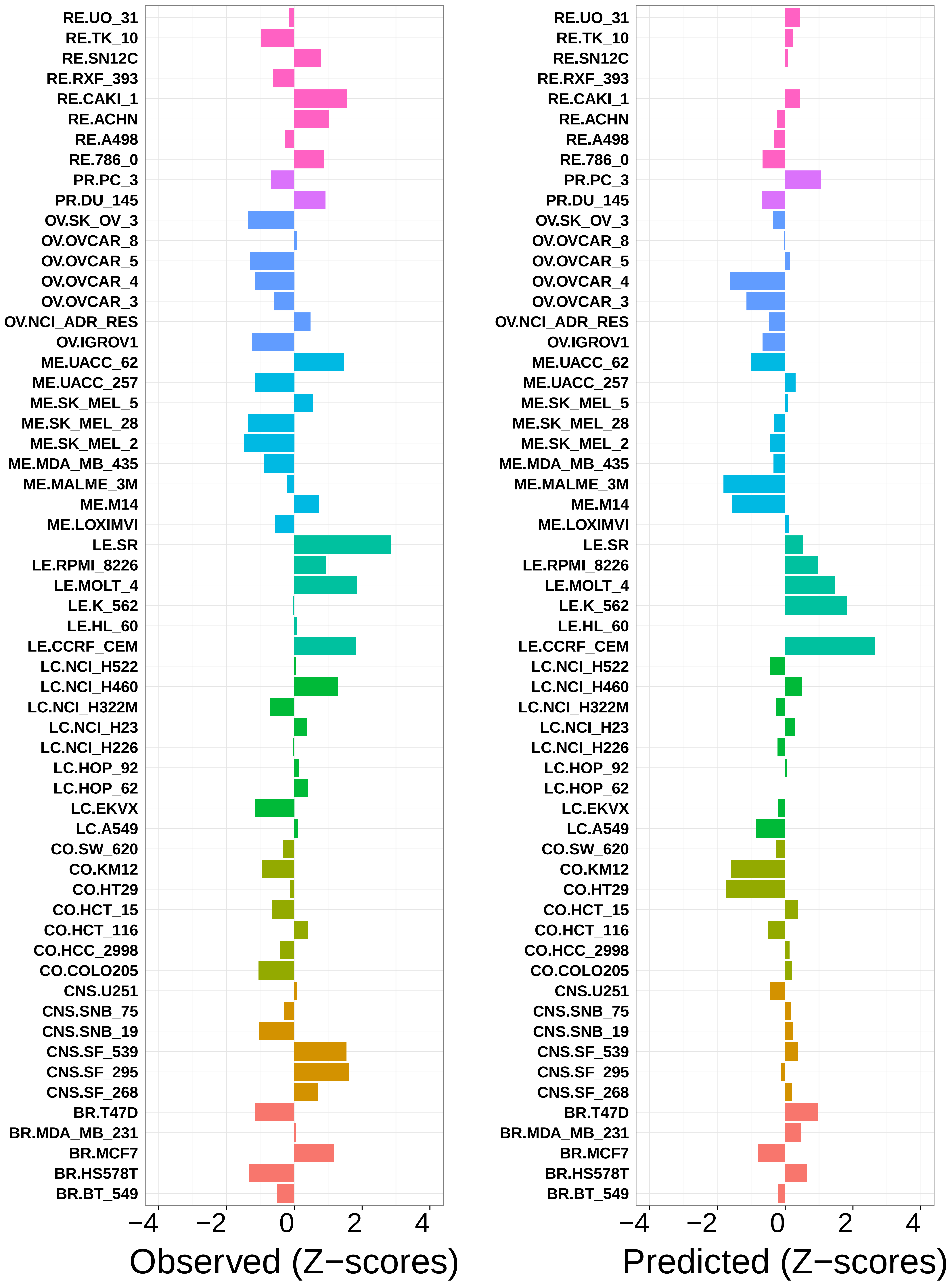

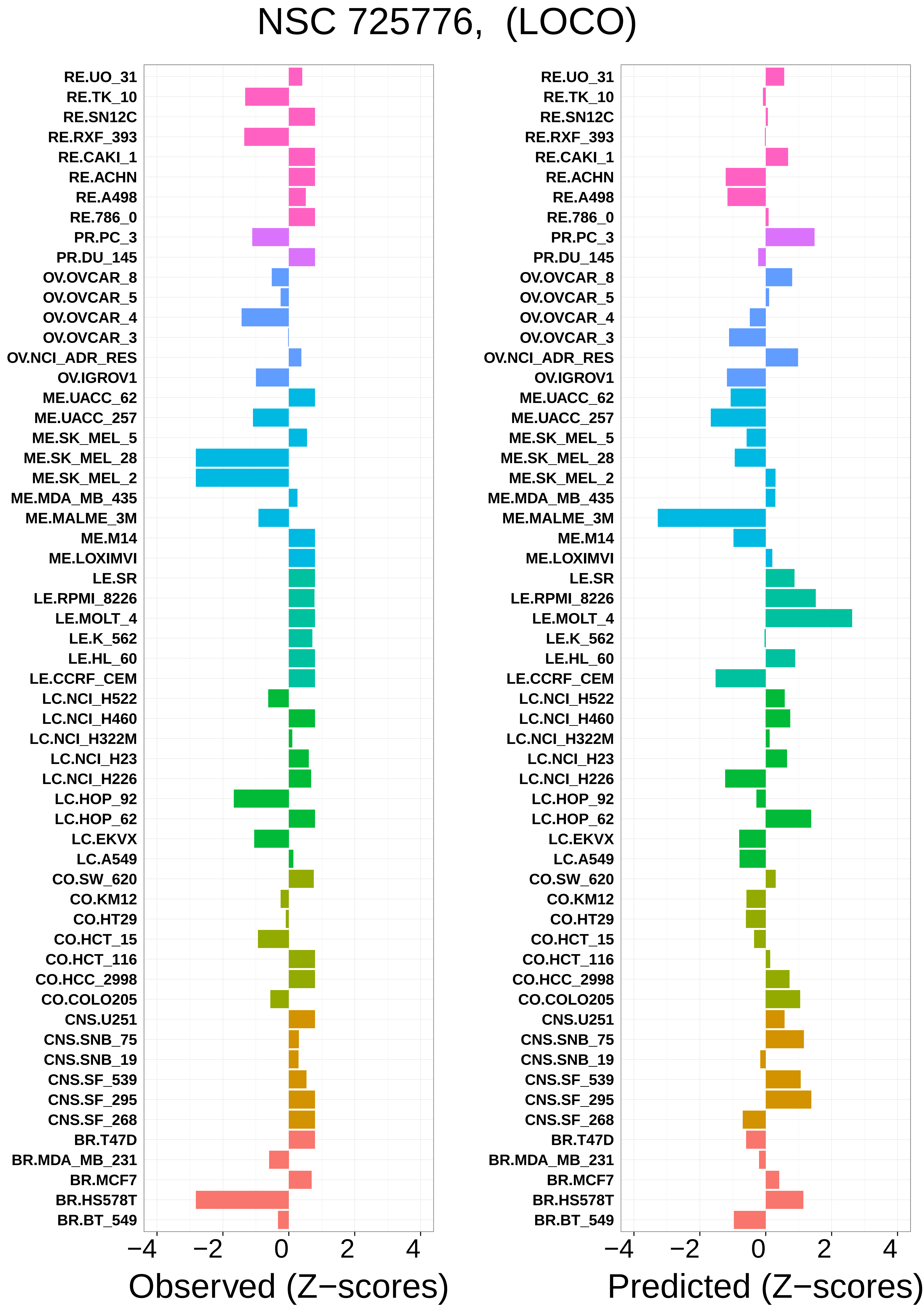
NSC 725776, (LOCO)
RE.UO_31
RE.TK_10
RE.SN12C
RE.RXF_393
RE.CAKI_1
RE.ACHN
RE.A498
RE.786_0
PR.PC_3
PR.DU_145
OV.OVCAR_8
OV.OVCAR_5
OV.OVCAR_4
OV.OVCAR_3
OV.NCI_ADR_RES
OV.IGROV1
ME.UACC_62
ME.UACC_257
ME.SK_MEL_5
ME.SK_MEL_28
ME.SK_MEL_2
ME.MDA_MB_435
ME.MALME_3M
ME.M14
ME.LOXIMVI
LE.SR
LE.RPMI_8226
LE.MOLT_4
LE.K_562
LE.HL_60
LE.CCRF_CEM
LC.NCI_H522
LC.NCI_H460
LC.NCI_H322M
LC.NCI_H23
LC.NCI_H226
LC.HOP_92
LC.HOP_62
LC.EKVX
LC.A549
CO.SW_620
CO.KM12
CO.HT29
CO.HCT_15
CO.HCT_116
CO.HCC_2998
CO.COLO205
CNS.U251
CNS.SNB_75
CNS.SNB_19
CNS.SF_539
CNS.SF_295
CNS.SF_268
BR.T47D
BR.MDA_MB_231
BR.MCF7
BR.HS578T
BR.BT_549
−4 −2 0 2 4
Observed (Z−scores)
Predicted (Z−scores)

NSC 726630, (LOCO)

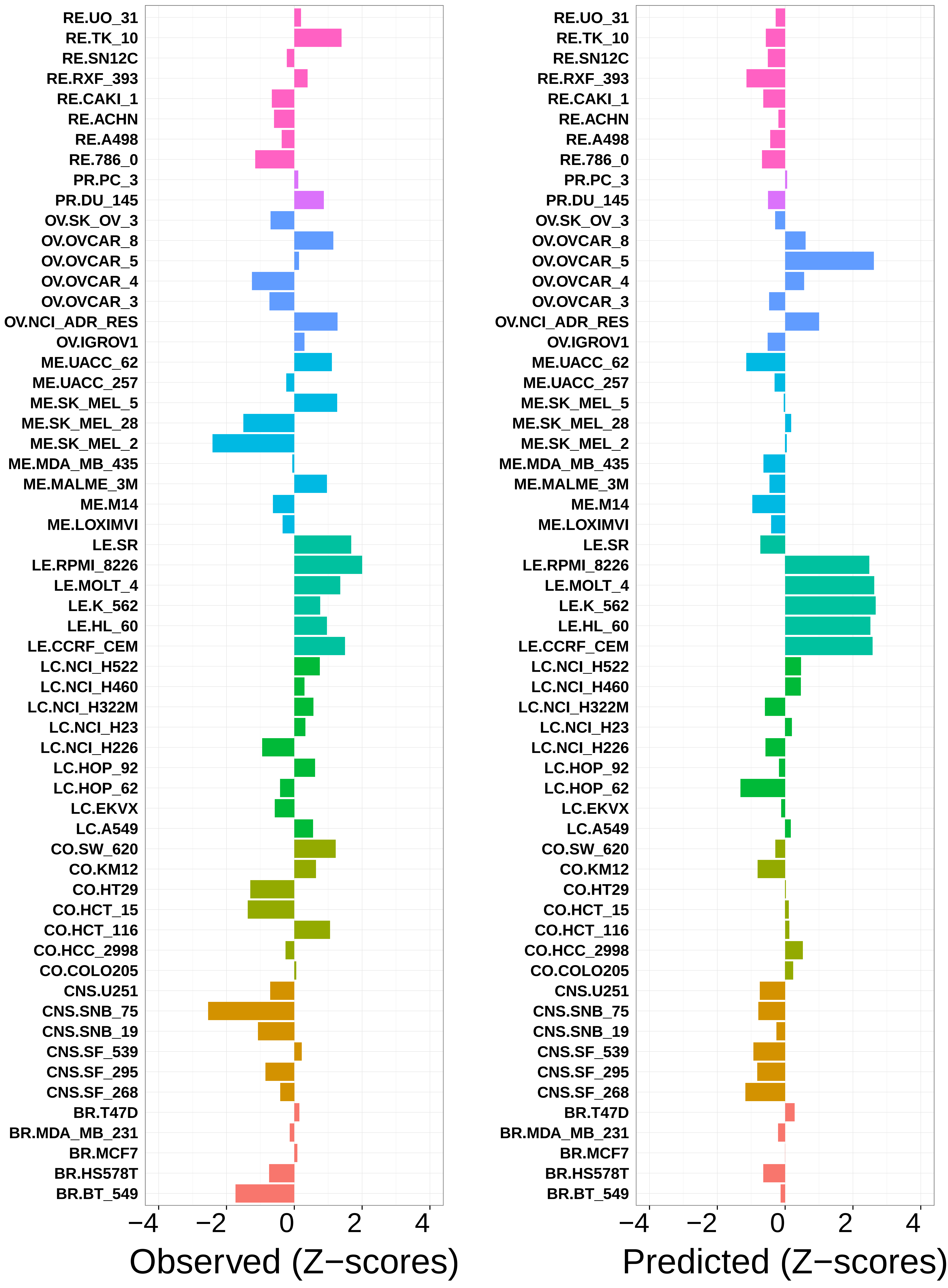

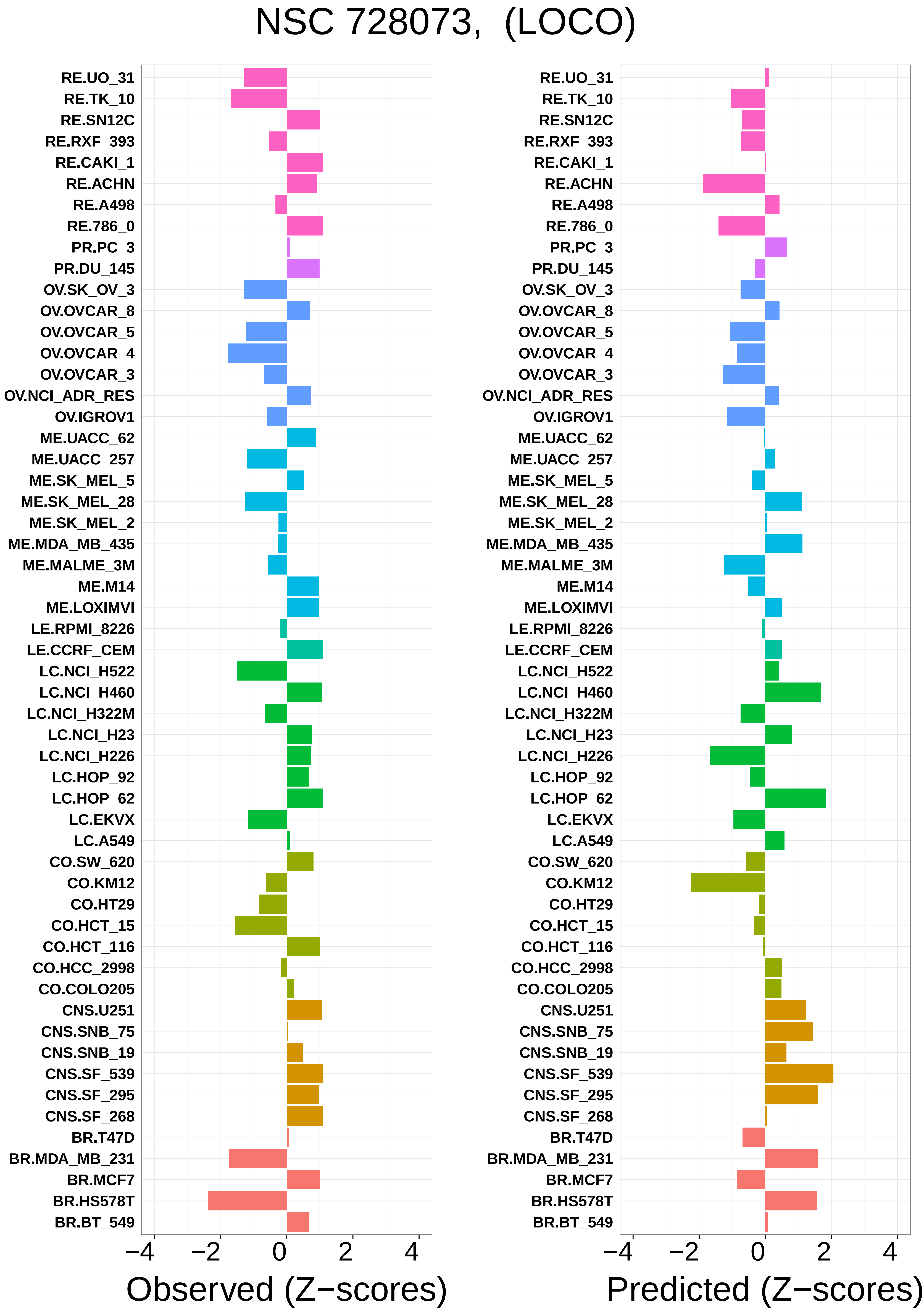

NSC 728073, (LOCO)
RE.UO_31
RE.TK_10
RE.SN12C
RE.RXF_393
RE.CAKI_1
RE.ACHN
RE.A498
RE.786_0
PR.PC_3
PR.DU_145
OV.SK_OV_3
OV.OVCAR_8
OV.OVCAR_5
OV.OVCAR_4
OV.OVCAR_3
OV.NCI_ADR_RES
OV.IGROV1
ME.UACC_62
ME.UACC_257
ME.SK_MEL_5
ME.SK_MEL_28
ME.SK_MEL_2
ME.MDA_MB_435
ME.MALME_3M
ME.M14
ME.LOXIMVI
LE.RPMI_8226
LE.CCRF_CEM
LC.NCI_H522
LC.NCI_H460
LC.NCI_H322M
LC.NCI_H23
LC.NCI_H226
LC.HOP_92
LC.HOP_62
LC.EKVX
LC.A549
CO.SW_620
CO.KM12
CO.HT29
CO.HCT_15
CO.HCT_116
CO.HCC_2998
CO.COLO205
CNS.U251
CNS.SNB_75
CNS.SNB_19
CNS.SF_539
CNS.SF_295
CNS.SF_268
BR.T47D
BR.MDA_MB_231
BR.MCF7
BR.HS578T
BR.BT_549
−4 −2 0 2 4
Observed (Z−scores)
Predicted (Z−scores)

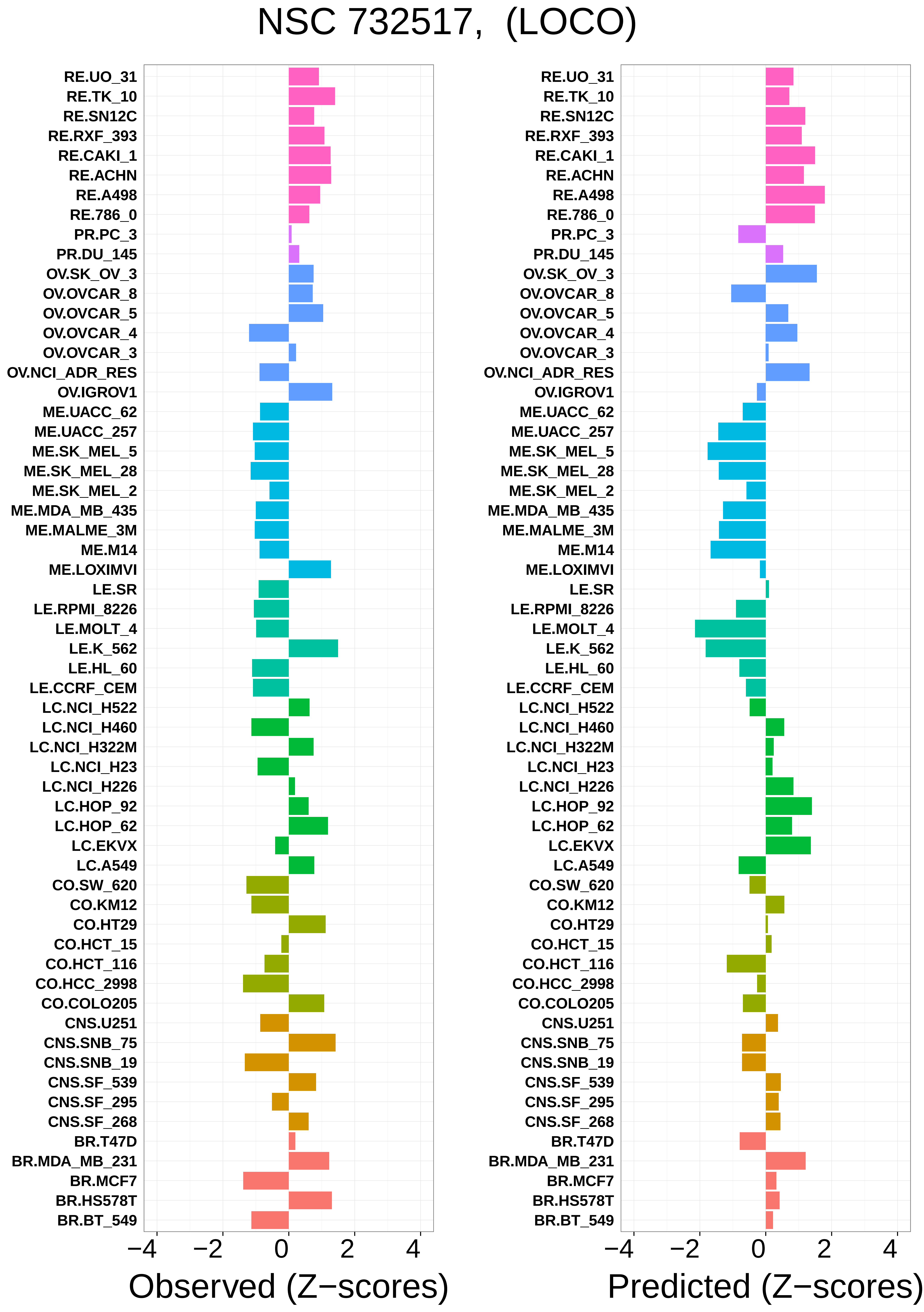
NSC 732517, (LOCO)
RE.UO_31
RE.TK_10
RE.SN12C
RE.RXF_393
RE.CAKI_1
RE.ACHN
RE.A498
RE.786_0
PR.PC_3
PR.DU_145
OV.SK_OV_3
OV.OVCAR_8
OV.OVCAR_5
OV.OVCAR_4
OV.OVCAR_3
OV.NCI_ADR_RES
OV.IGROV1
ME.UACC_62
ME.UACC_257
ME.SK_MEL_5
ME.SK_MEL_28
ME.SK_MEL_2
ME.MDA_MB_435
ME.MALME_3M
ME.M14
ME.LOXIMVI
LE.SR
LE.RPMI_8226
LE.MOLT_4
LE.K_562
LE.HL_60
LE.CCRF_CEM
LC.NCI_H522
LC.NCI_H460
LC.NCI_H322M
LC.NCI_H23
LC.NCI_H226
LC.HOP_92
LC.HOP_62
LC.EKVX
LC.A549
CO.SW_620
CO.KM12
CO.HT29
CO.HCT_15
CO.HCT_116
CO.HCC_2998
CO.COLO205
CNS.U251
CNS.SNB_75
CNS.SNB_19
CNS.SF_539
CNS.SF_295
CNS.SF_268
BR.T47D
BR.MDA_MB_231
BR.MCF7
BR.HS578T
BR.BT_549
−4 −2 0 2 4
Observed (Z−scores)
Predicted (Z−scores)

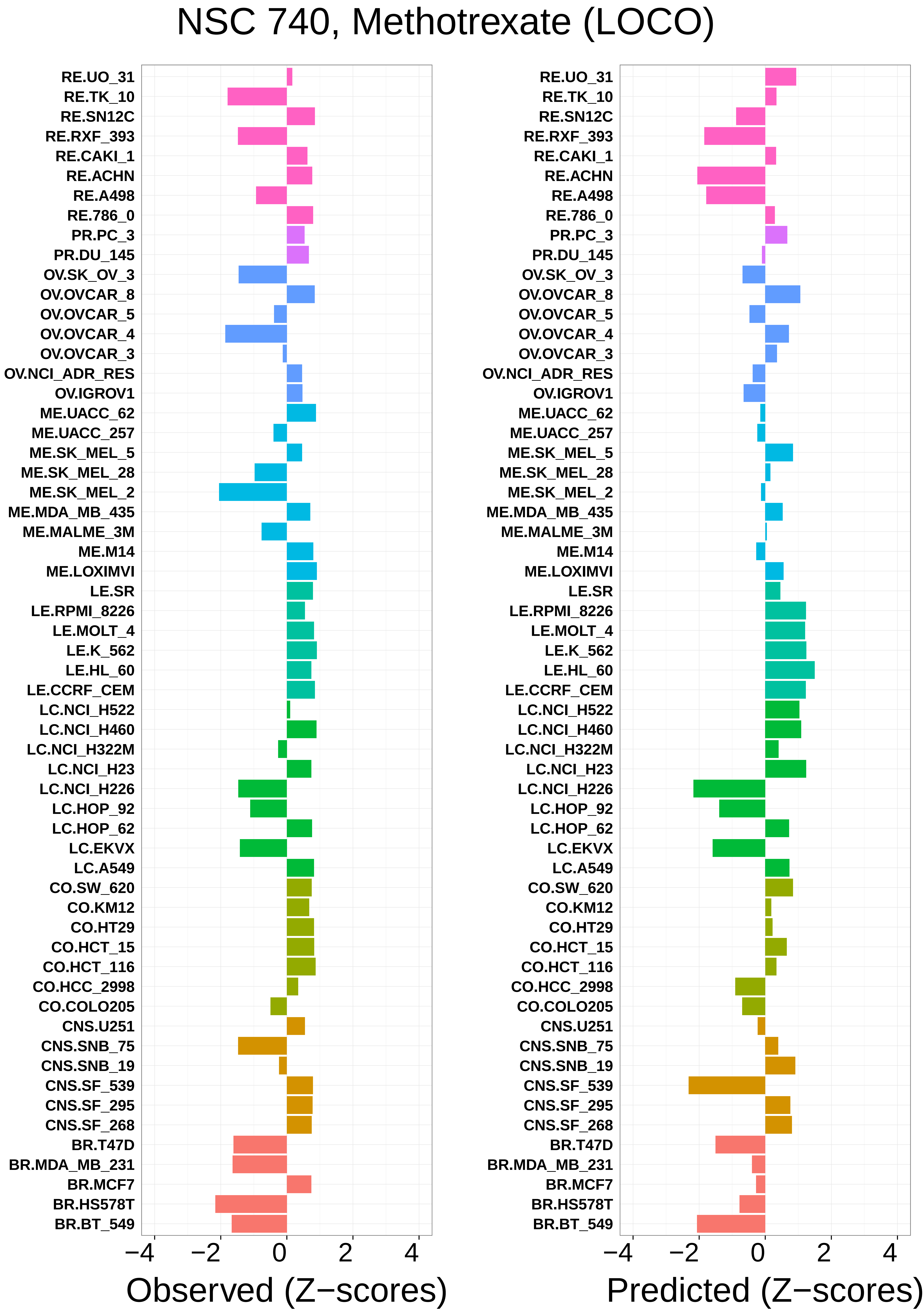
NSC 740, Methotrexate (LOCO)
RE.UO_31
RE.TK_10
RE.SN12C
RE.RXF_393
RE.CAKI_1
RE.ACHN
RE.A498
RE.786_0
PR.PC_3
PR.DU_145
OV.SK_OV_3
OV.OVCAR_8
OV.OVCAR_5
OV.OVCAR_4
OV.OVCAR_3
OV.NCI_ADR_RES
OV.IGROV1
ME.UACC_62
ME.UACC_257
ME.SK_MEL_5
ME.SK_MEL_28
ME.SK_MEL_2
ME.MDA_MB_435
ME.MALME_3M
ME.M14
ME.LOXIMVI
LE.SR
LE.RPMI_8226
LE.MOLT_4
LE.K_562
LE.HL_60
LE.CCRF_CEM
LC.NCI_H522
LC.NCI_H460
LC.NCI_H322M
LC.NCI_H23
LC.NCI_H226
LC.HOP_92
LC.HOP_62
LC.EKVX
LC.A549
CO.SW_620
CO.KM12
CO.HT29
CO.HCT_15
CO.HCT_116
CO.HCC_2998
CO.COLO205
CNS.U251
CNS.SNB_75
CNS.SNB_19
CNS.SF_539
CNS.SF_295
CNS.SF_268
BR.T47D
BR.MDA_MB_231
BR.MCF7
BR.HS578T
BR.BT_549
−4 −2 0 2 4
Observed (Z−scores)
−4 −2 0 2 4
Predicted (Z−scores)

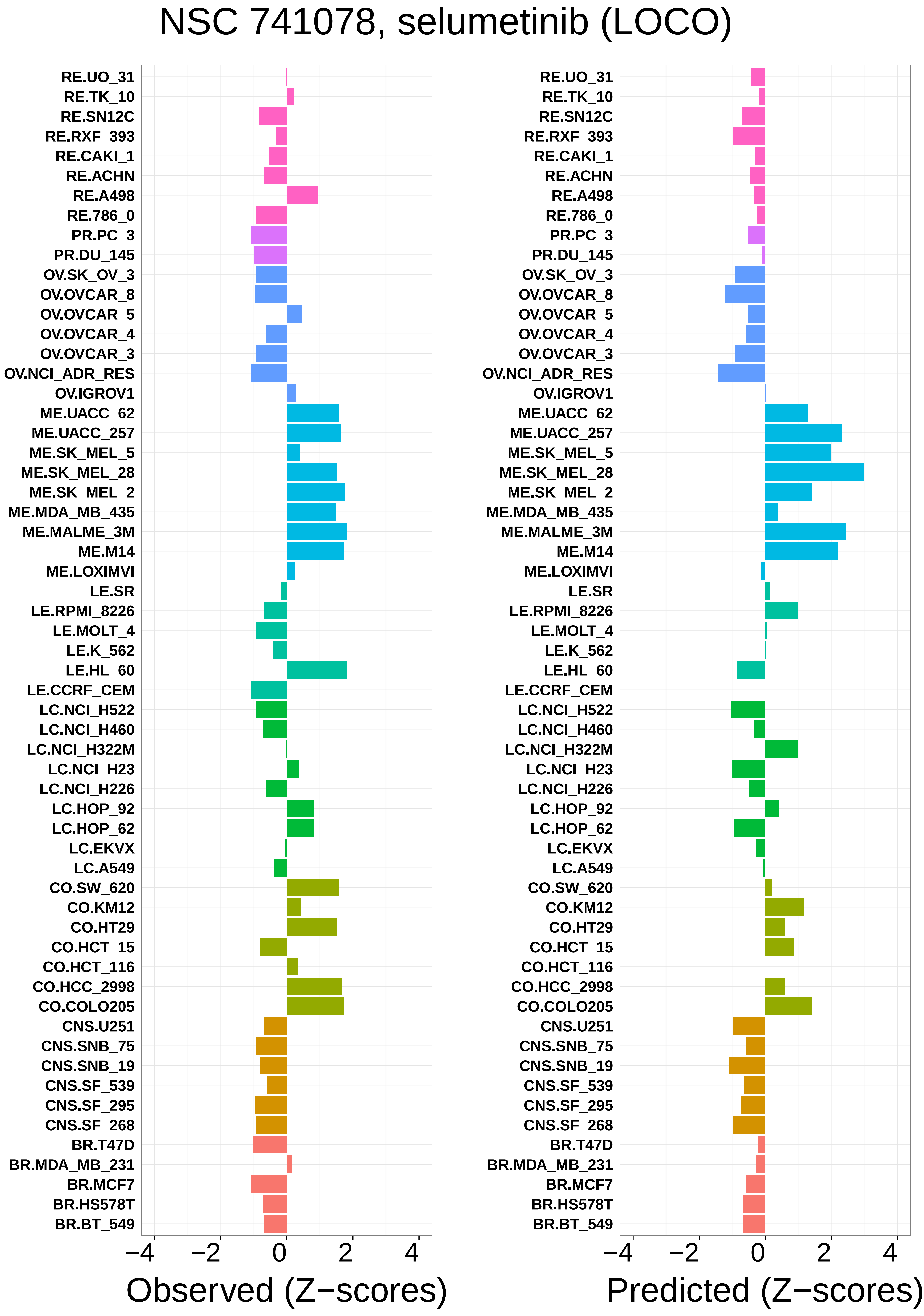
NSC 741078, selumetinib (LOCO)
RE.UO_31
RE.TK_10
RE.SN12C
RE.RXF_393
RE.CAKI_1
RE.ACHN
RE.A498
RE.786_0
PR.PC_3
PR.DU_145
OV.SK_OV_3
OV.OVCAR_8
OV.OVCAR_5
OV.OVCAR_4
OV.OVCAR_3
OV.NCI_ADR_RES
OV.IGROV1
ME.UACC_62
ME.UACC_257
ME.SK_MEL_5
ME.SK_MEL_28
ME.SK_MEL_2
ME.MDA_MB_435
ME.MALME_3M
ME.M14
ME.LOXIMVI
LE.SR
LE.RPMI_8226
LE.MOLT_4
LE.K_562
LE.HL_60
LE.CCRF_CEM
LC.NCI_H522
LC.NCI_H460
LC.NCI_H322M
LC.NCI_H23
LC.NCI_H226
LC.HOP_92
LC.HOP_62
LC.EKVX
LC.A549
CO.SW_620
CO.KM12
CO.HT29
CO.HCT_15
CO.HCT_116
CO.HCC_2998
CO.COLO205
CNS.U251
CNS.SNB_75
CNS.SNB_19
CNS.SF_539
CNS.SF_295
CNS.SF_268
BR.T47D
BR.MDA_MB_231
BR.MCF7
BR.HS578T
BR.BT_549
−4
−2
0
2
4
Observed (Z−scores)
Predicted (Z−scores)

NSC 745750, Tykerb (LOCO)

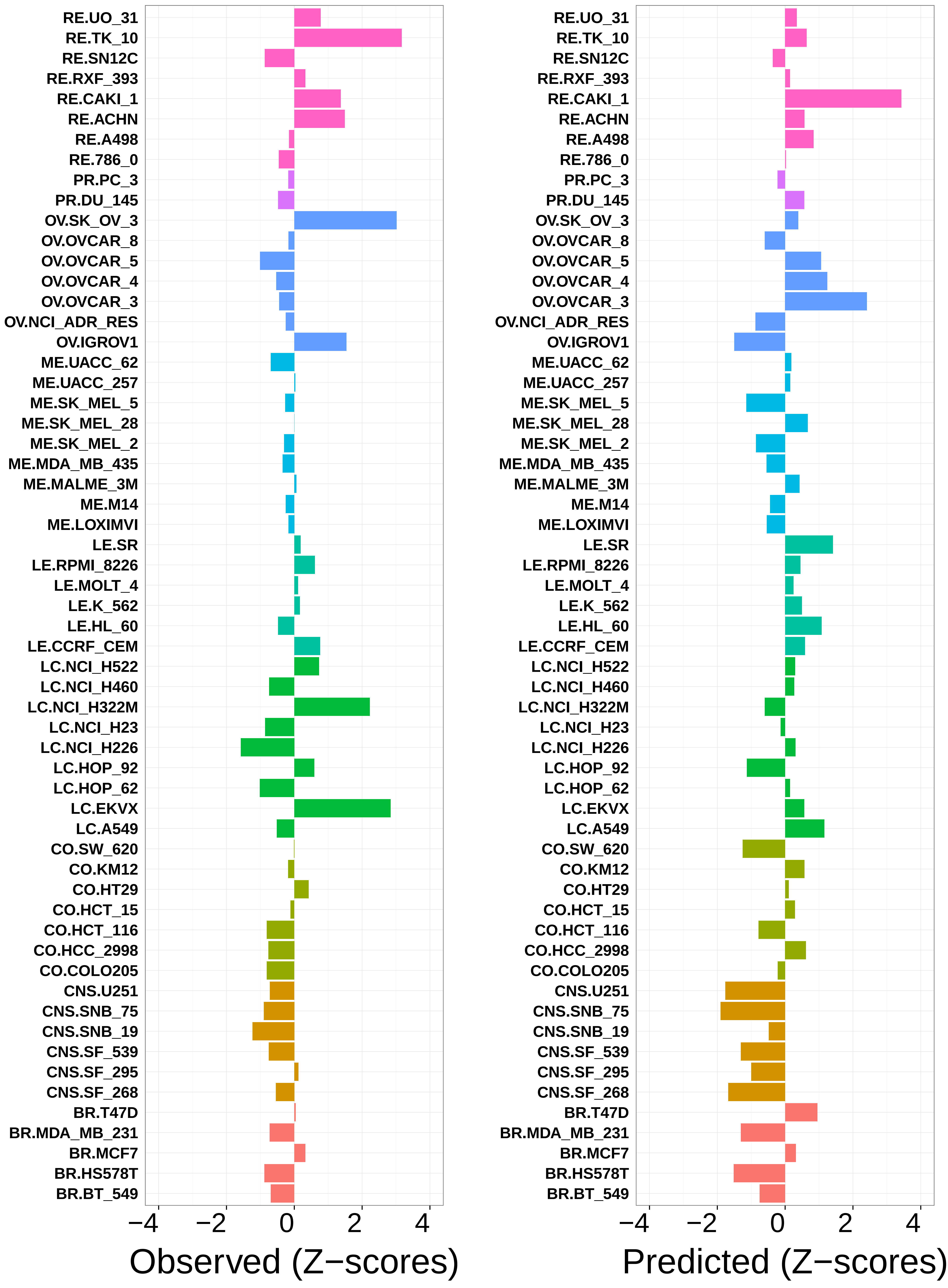

NSC 749226, (LOCO)

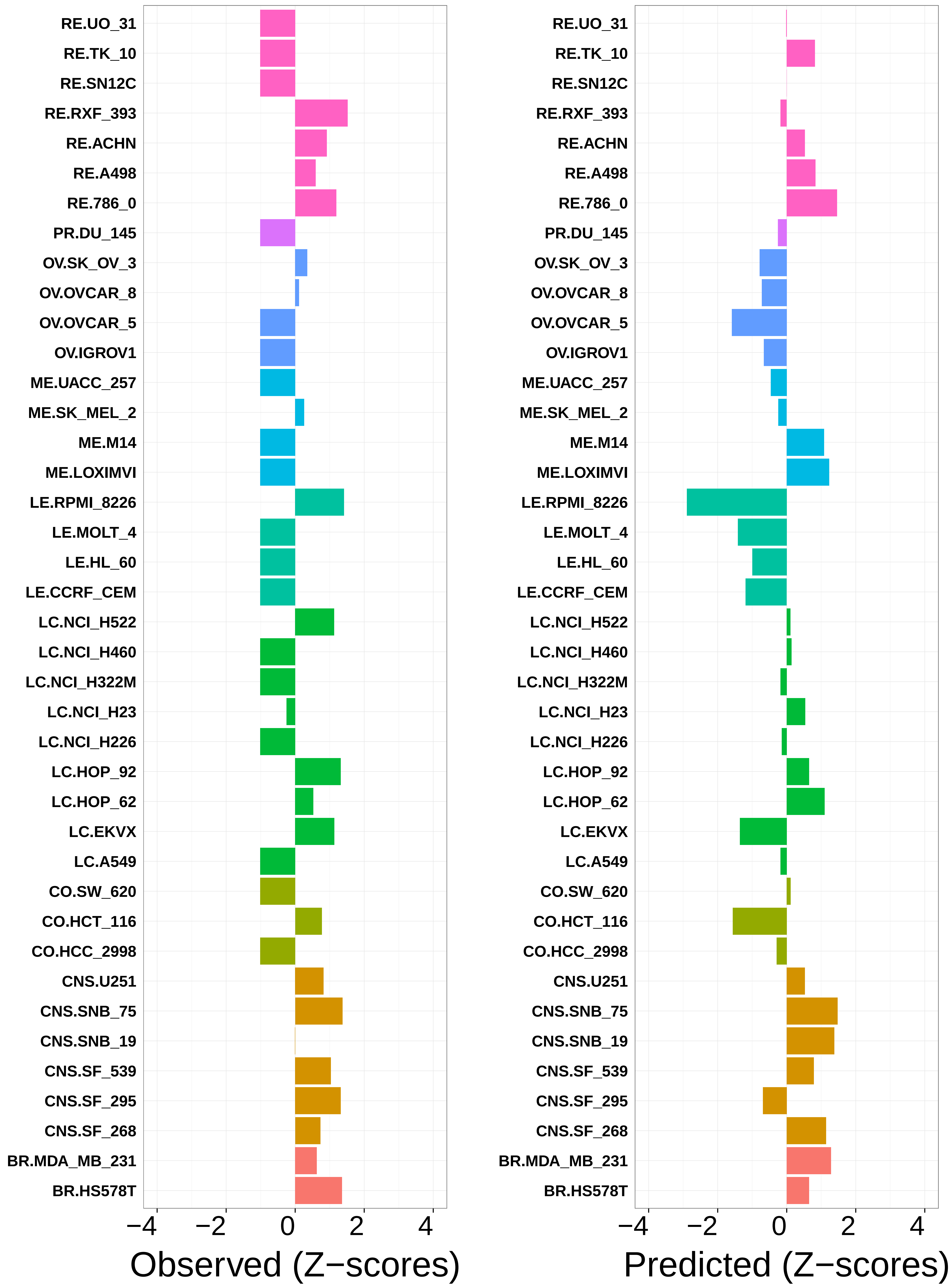

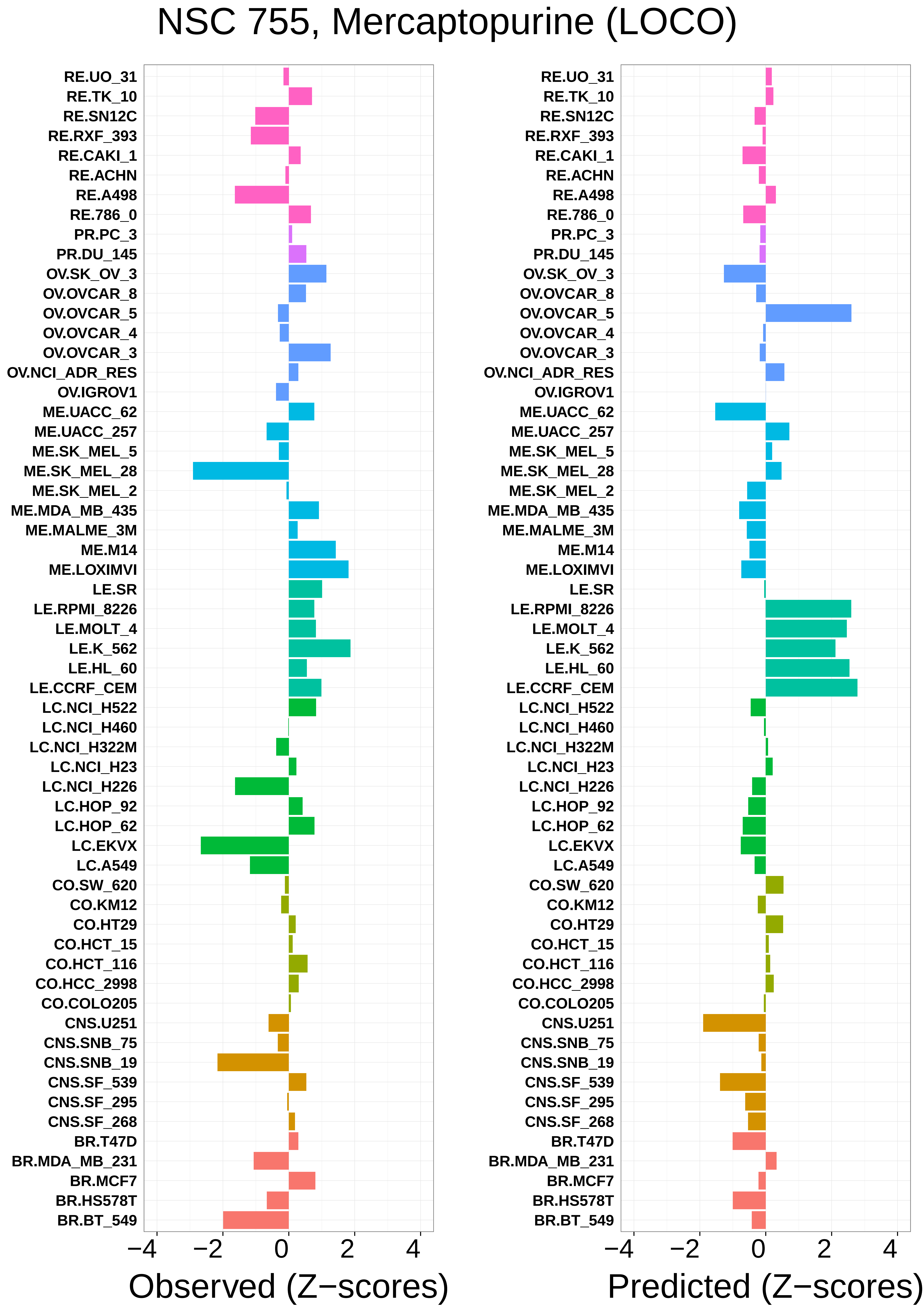
NSC 755, Mercaptopurine (LOCO)
RE.UO_31
RE.TK_10
RE.SN12C
RE.RXF_393
RE.CAKI_1
RE.ACHN
RE.A498
RE.786_0
PR.PC_3
PR.DU_145
OV.SK_OV_3
OV.OVCAR_8
OV.OVCAR_5
OV.OVCAR_4
OV.OVCAR_3
OV.NCI_ADR_RES
OV.IGROV1
ME.UACC_62
ME.UACC_257
ME.SK_MEL_5
ME.SK_MEL_28
ME.SK_MEL_2
ME.MDA_MB_435
ME.MALME_3M
ME.M14
ME.LOXIMVI
LE.SR
LE.RPMI_8226
LE.MOLT_4
LE.K_562
LE.HL_60
LE.CCRF_CEM
LC.NCI_H522
LC.NCI_H460
LC.NCI_H322M
LC.NCI_H23
LC.NCI_H226
LC.HOP_92
LC.HOP_62
LC.EKVX
LC.A549
CO.SW_620
CO.KM12
CO.HT29
CO.HCT_15
CO.HCT_116
CO.HCC_2998
CO.COLO205
CNS.U251
CNS.SNB_75
CNS.SNB_19
CNS.SF_539
CNS.SF_295
CNS.SF_268
BR.T47D
BR.MDA_MB_231
BR.MCF7
BR.HS578T
BR.BT_549
−4 −2 0 2 4
Observed (Z−scores)
−4 −2 0 2 4
Predicted (Z−scores)

NSC 762, Mechlorethamine_Hydrochloride (LOCO)

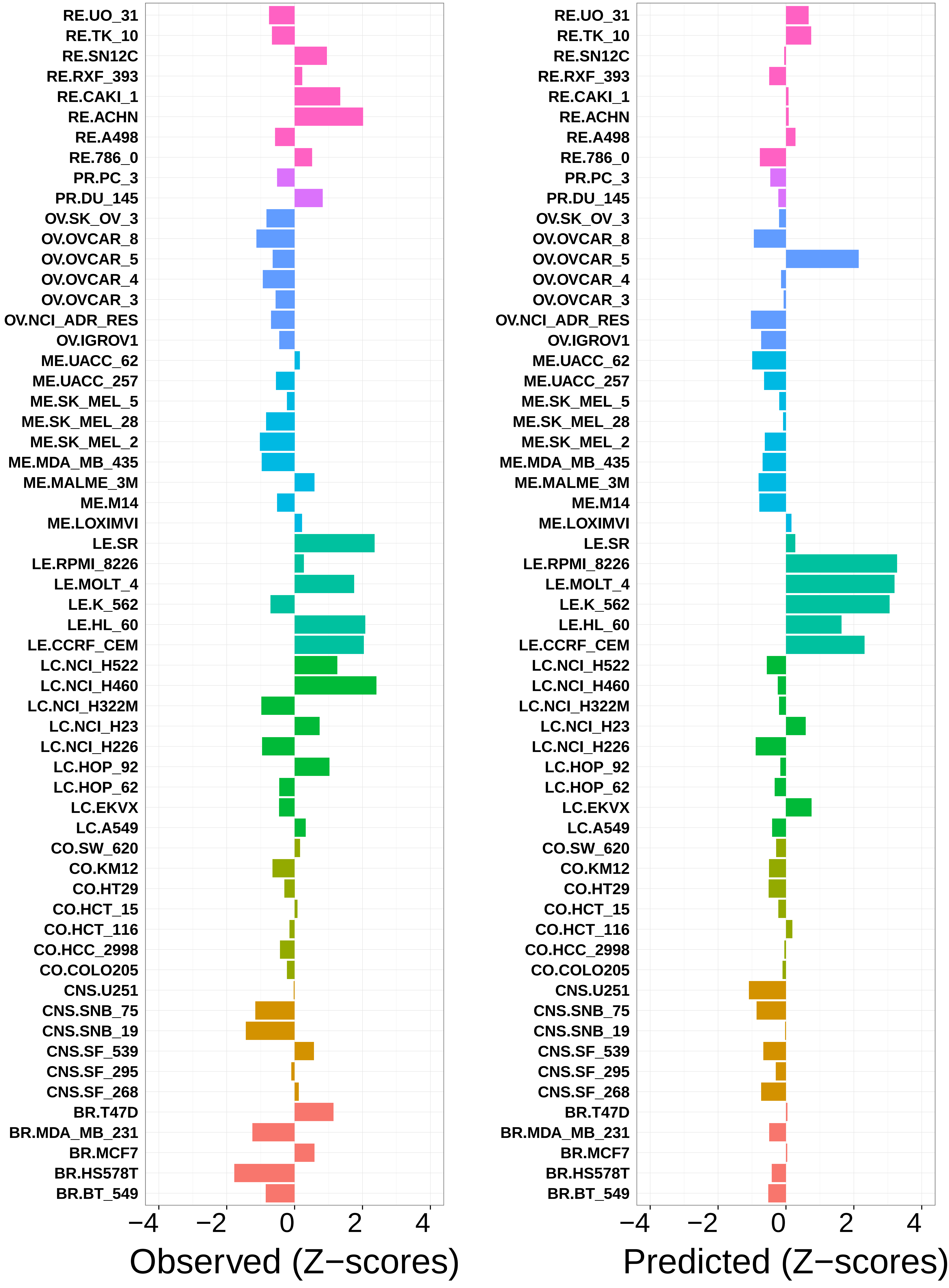

NSC 105014, Cladribine (LOTO)

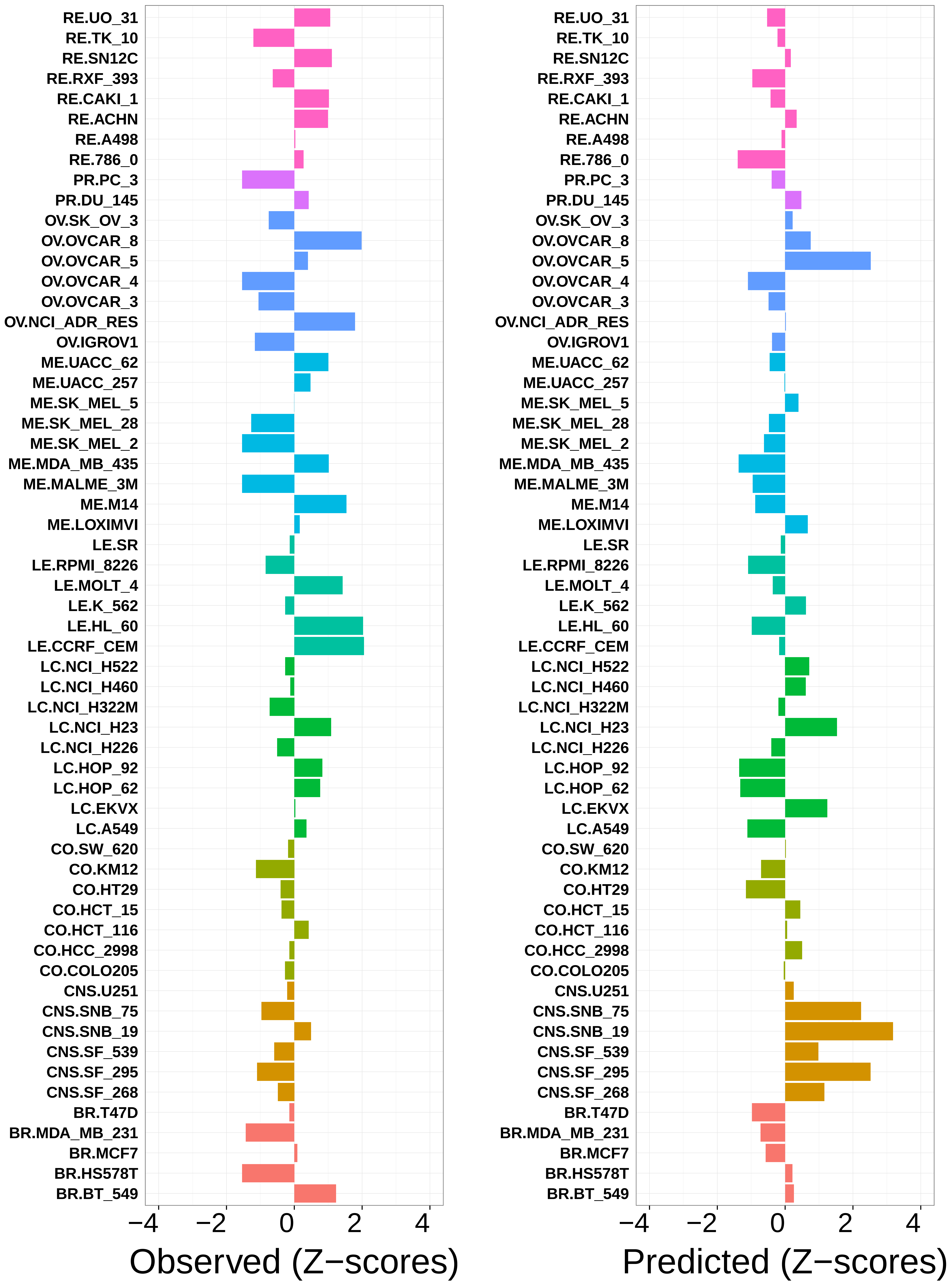

NSC 118218, Fludarabine (LOTO)

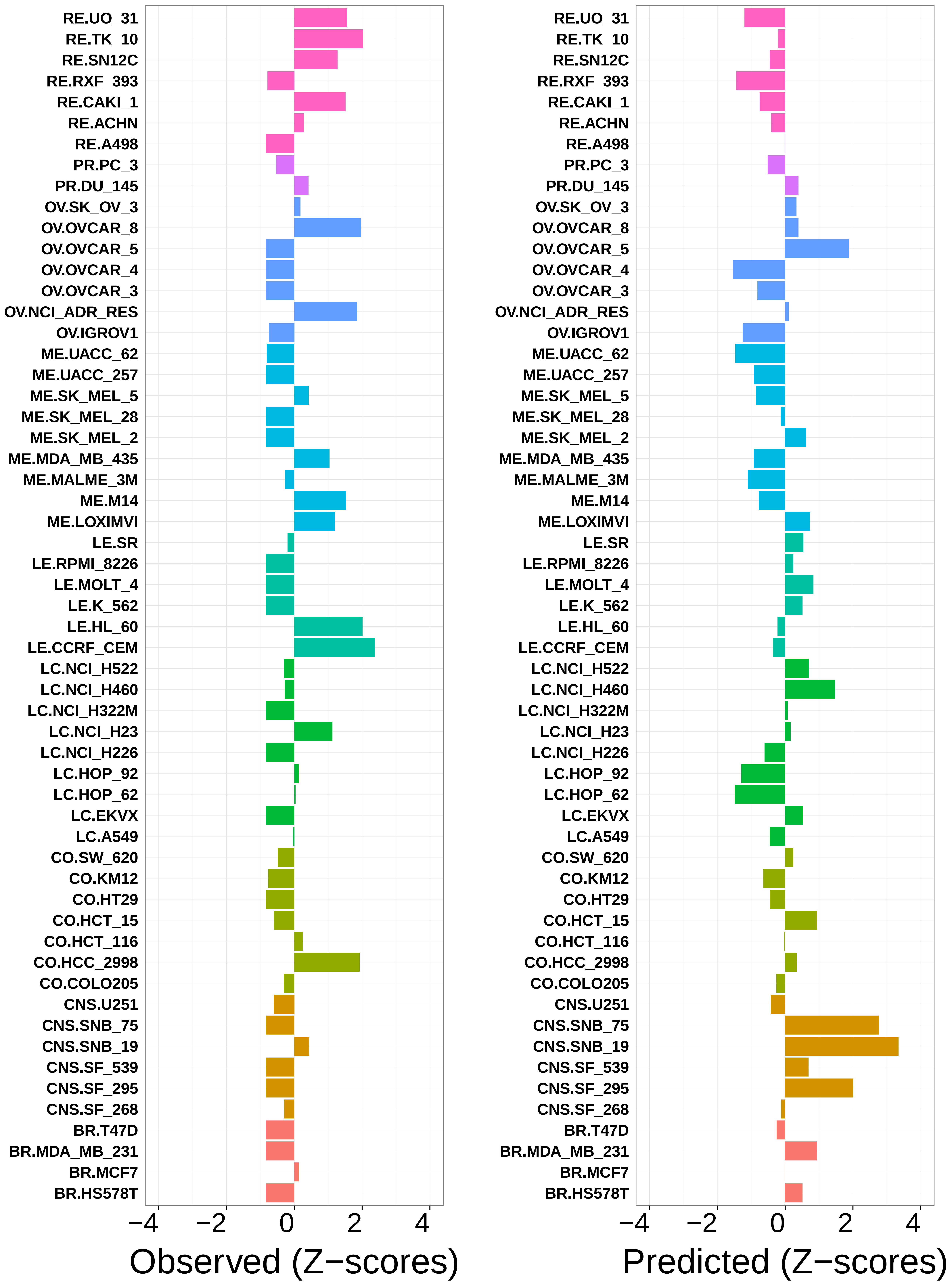

NSC 122819, Teniposide (LOTO)

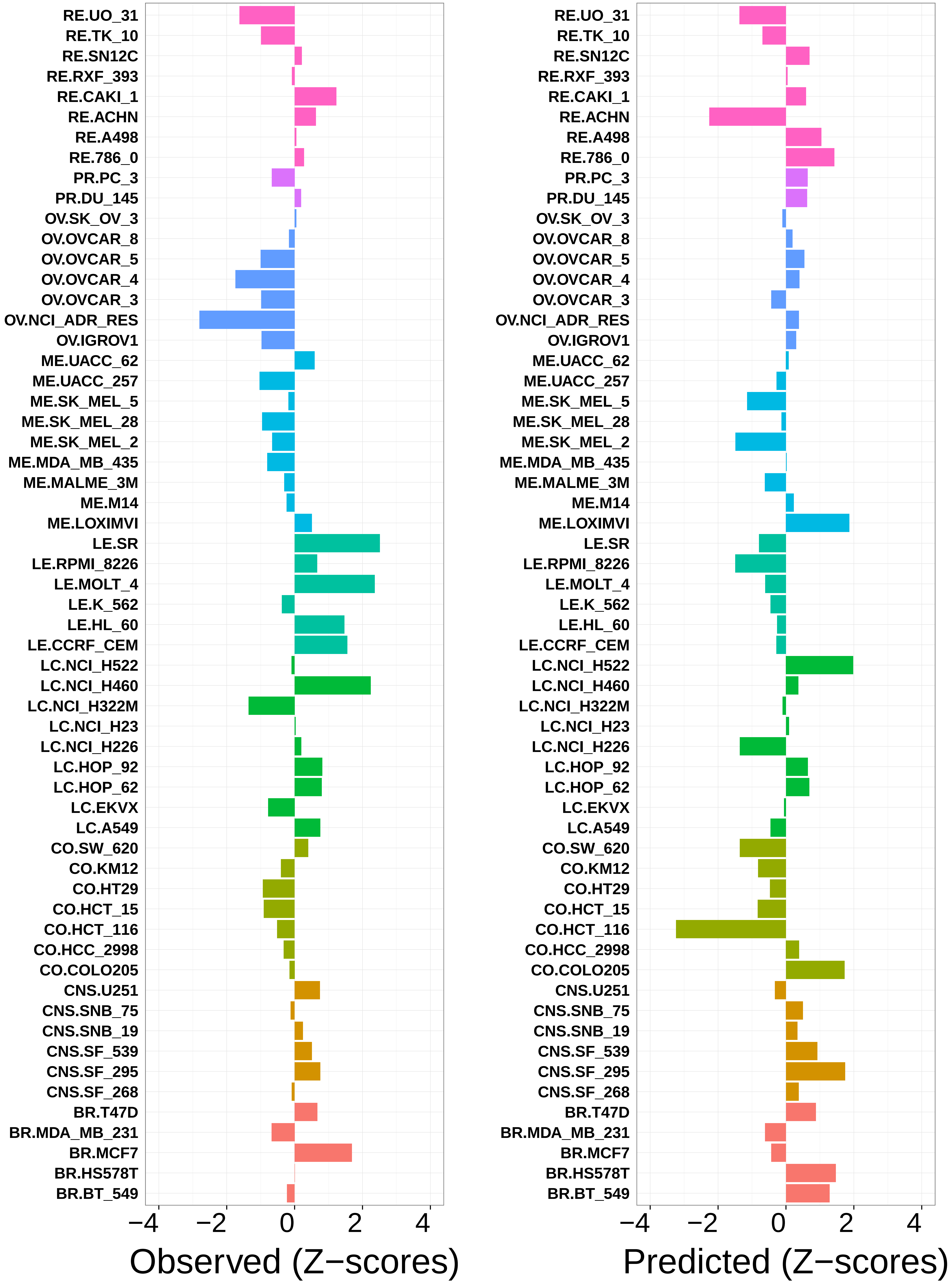

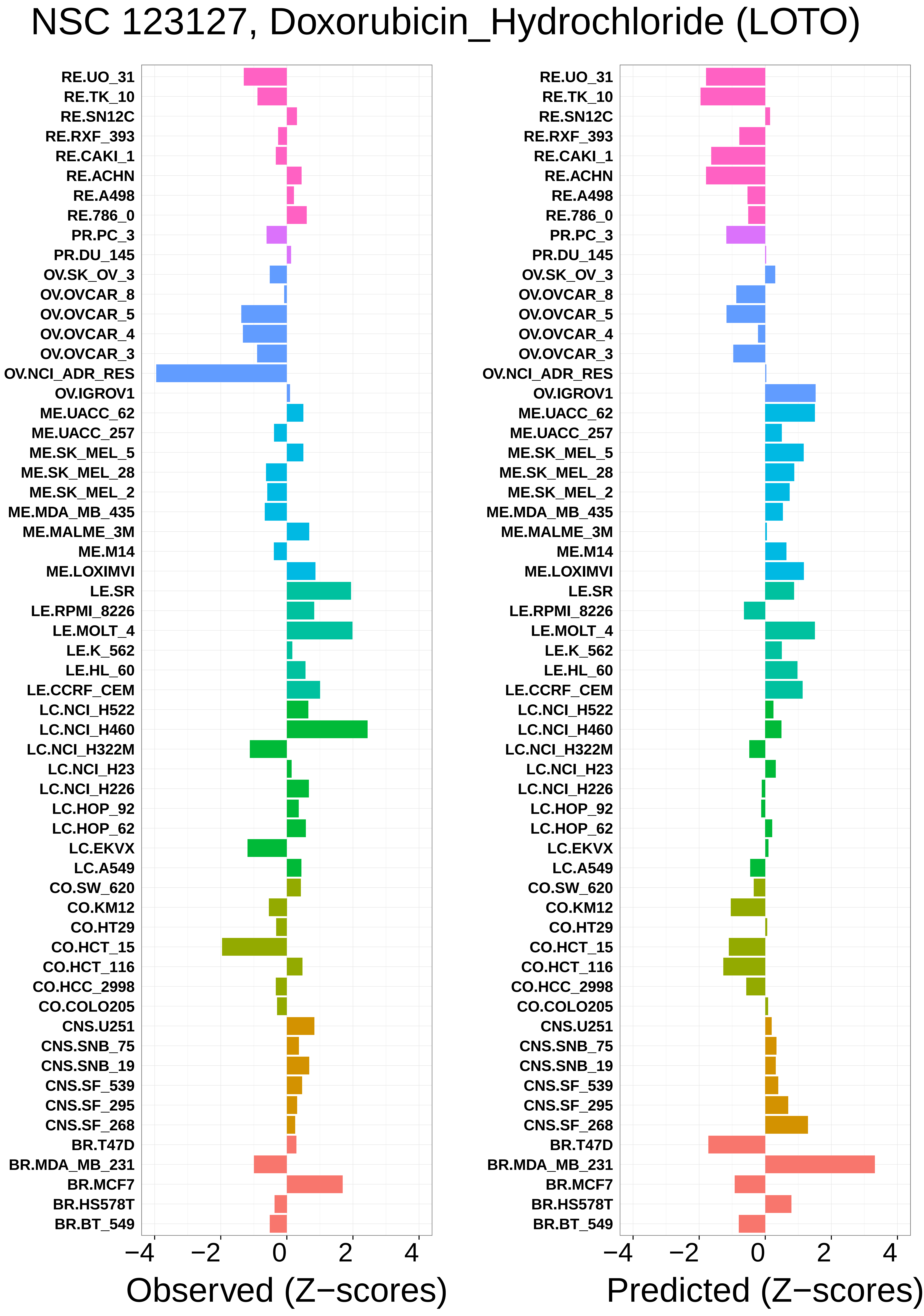
NSC 123127, Doxorubicin_Hydrochloride (LOTO)
RE.UO_31
RE.TK_10
RE.SN12C
RE.RXF_393
RE.CAKI_1
RE.ACHN
RE.A498
RE.786_0
PR.PC_3
PR.DU_145
OV.SK_OV_3
OV.OVCAR_8
OV.OVCAR_5
OV.OVCAR_4
OV.OVCAR_3
OV.NCI_ADR_RES
OV.IGROV1
ME.UACC_62
ME.UACC_257
ME.SK_MEL_5
ME.SK_MEL_28
ME.SK_MEL_2
ME.MDA_MB_435
ME.MALME_3M
ME.M14
ME.LOXIMVI
LE.SR
LE.RPMI_8226
LE.MOLT_4
LE.K_562
LE.HL_60
LE.CCRF_CEM
LC.NCI_H522
LC.NCI_H460
LC.NCI_H322M
LC.NCI_H23
LC.NCI_H226
LC.HOP_92
LC.HOP_62
LC.EKVX
LC.A549
CO.SW_620
CO.KM12
CO.HT29
CO.HCT_15
CO.HCT_116
CO.HCC_2998
CO.COLO205
CNS.U251
CNS.SNB_75
CNS.SNB_19
CNS.SF_539
CNS.SF_295
CNS.SF_268
BR.T47D
BR.MDA_MB_231
BR.MCF7
BR.HS578T
BR.BT_549
−4
−2
0
2
4
Observed (Z−scores)
Predicted (Z−scores)

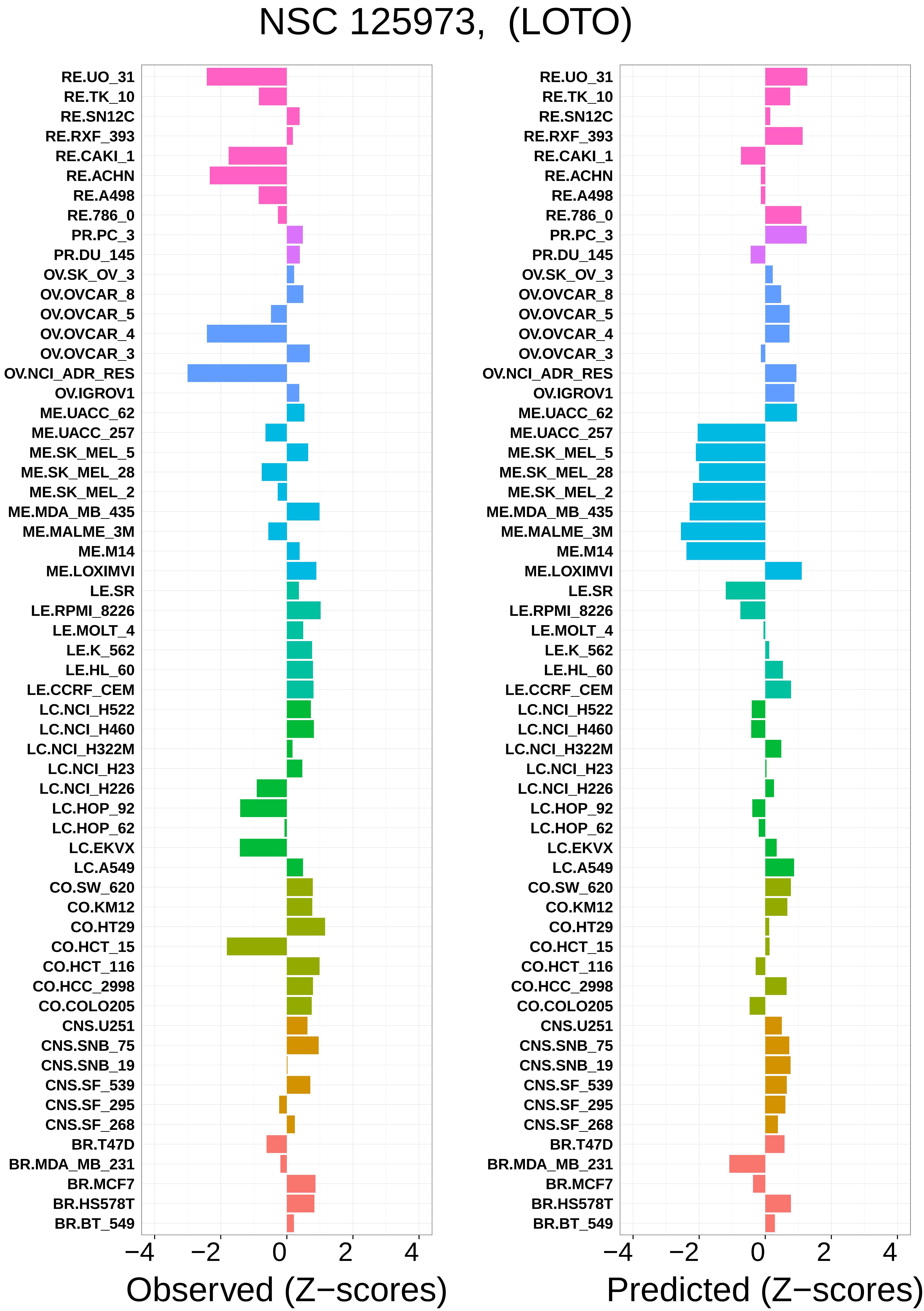

NSC 125973, (LOTO)
RE.UO_31
RE.TK_10
RE.SN12C
RE.RXF_393
RE.CAKI_1
RE.ACHN
RE.A498
RE.786_0
PR.PC_3
PR.DU_145
OV.SK_OV_3
OV.OVCAR_8
OV.OVCAR_5
OV.OVCAR_4
OV.OVCAR_3
OV.NCI_ADR_RES
OV.IGROV1
ME.UACC_62
ME.UACC_257
ME.SK_MEL_5
ME.SK_MEL_28
ME.SK_MEL_2
ME.MDA_MB_435
ME.MALME_3M
ME.M14
ME.LOXIMVI
LE.SR
LE.RPMI_8226
LE.MOLT_4
LE.K_562
LE.HL_60
LE.CCRF_CEM
LC.NCI_H522
LC.NCI_H460
LC.NCI_H322M
LC.NCI_H23
LC.NCI_H226
LC.HOP_92
LC.HOP_62
LC.EKVX
LC.A549
CO.SW_620
CO.KM12
CO.HT29
CO.HCT_15
CO.HCT_116
CO.HCC_2998
CO.COLO205
CNS.U251
CNS.SNB_75
CNS.SNB_19
CNS.SF_539
CNS.SF_295
CNS.SF_268
BR.T47D
BR.MDA_MB_231
BR.MCF7
BR.HS578T
BR.BT_549
−4 −2 0 2 4
Observed (Z−scores)
−4 −2 0 2 4
Predicted (Z−scores)

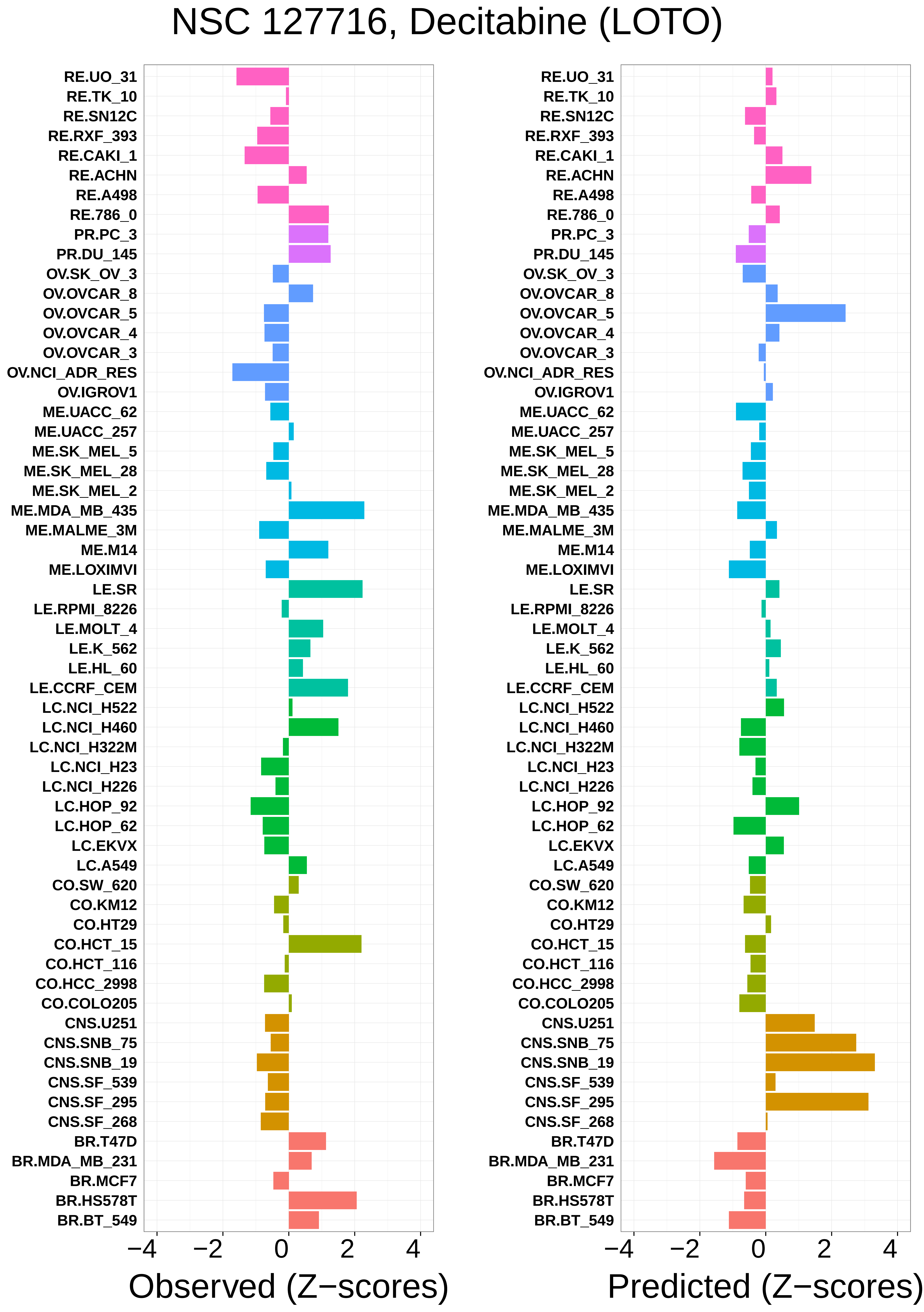
NSC 127716, Decitabine (LOTO)
RE.UO_31
RE.TK_10
RE.SN12C
RE.RXF_393
RE.CAKI_1
RE.ACHN
RE.A498
RE.786_0
PR.PC_3
PR.DU_145
OV.SK_OV_3
OV.OVCAR_8
OV.OVCAR_5
OV.OVCAR_4
OV.OVCAR_3
OV.NCI_ADR_RES
OV.IGROV1
ME.UACC_62
ME.UACC_257
ME.SK_MEL_5
ME.SK_MEL_28
ME.SK_MEL_2
ME.MDA_MB_435
ME.MALME_3M
ME.M14
ME.LOXIMVI
LE.SR
LE.RPMI_8226
LE.MOLT_4
LE.K_562
LE.HL_60
LE.CCRF_CEM
LC.NCI_H522
LC.NCI_H460
LC.NCI_H322M
LC.NCI_H23
LC.NCI_H226
LC.HOP_92
LC.HOP_62
LC.EKVX
LC.A549
CO.SW_620
CO.KM12
CO.HT29
CO.HCT_15
CO.HCT_116
CO.HCC_2998
CO.COLO205
CNS.U251
CNS.SNB_75
CNS.SNB_19
CNS.SF_539
CNS.SF_295
CNS.SF_268
BR.T47D
BR.MDA_MB_231
BR.MCF7
BR.HS578T
BR.BT_549
−4 −2 0 2 4
Observed (Z−scores)
Predicted (Z−scores)

NSC 141540, (LOTO)

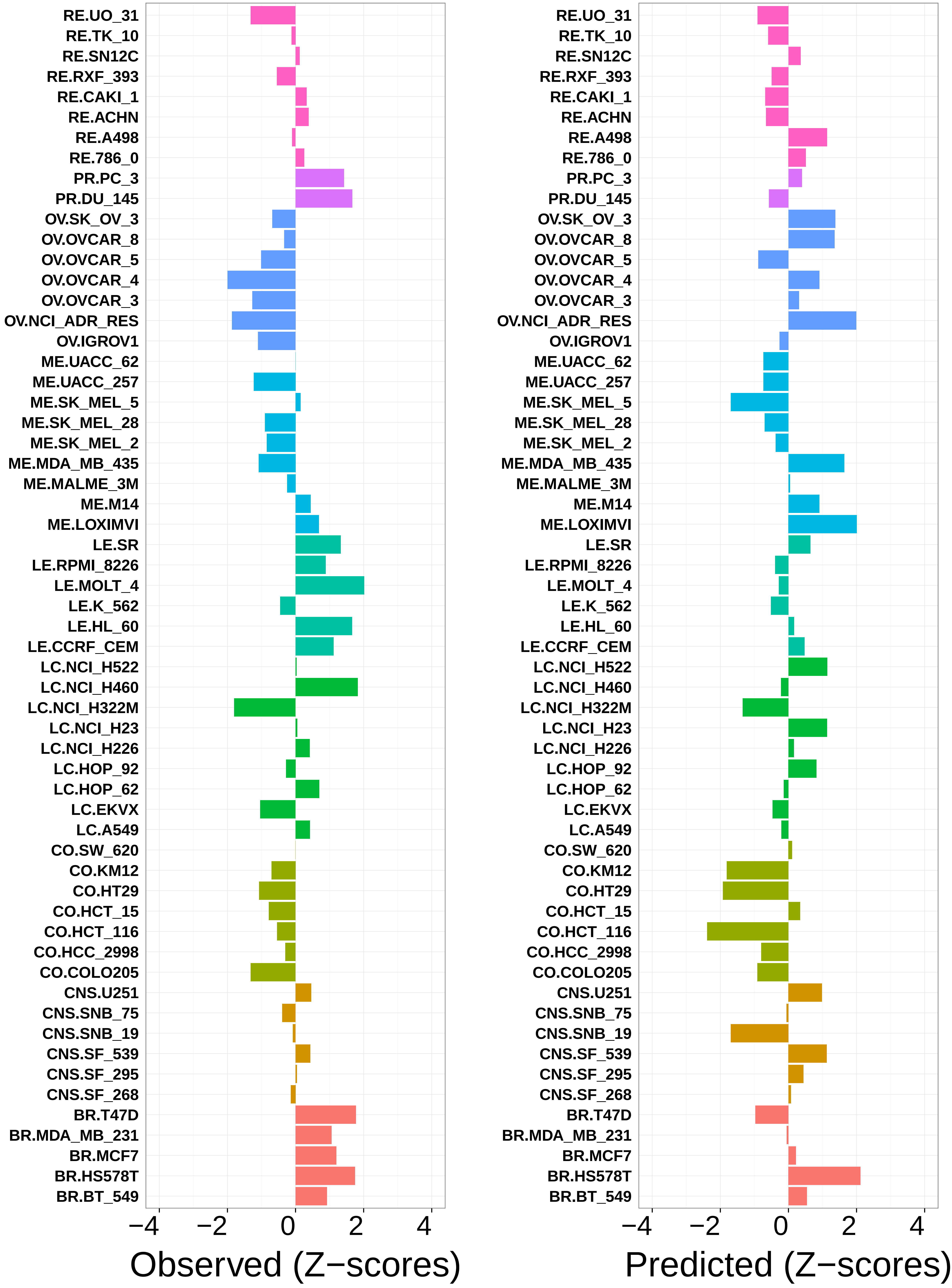

NSC 141633, (LOTO)

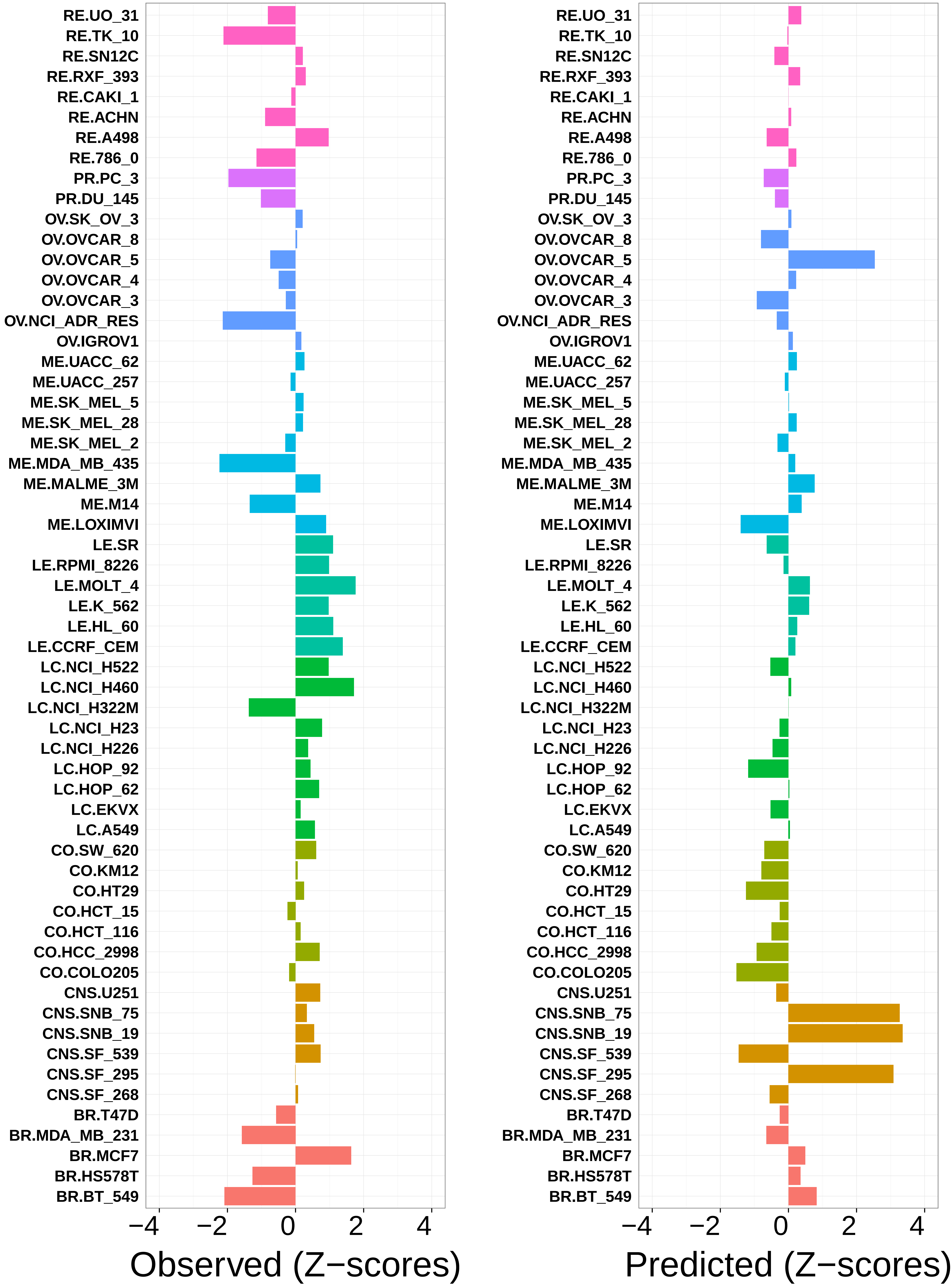

NSC 174939, (LOTO)

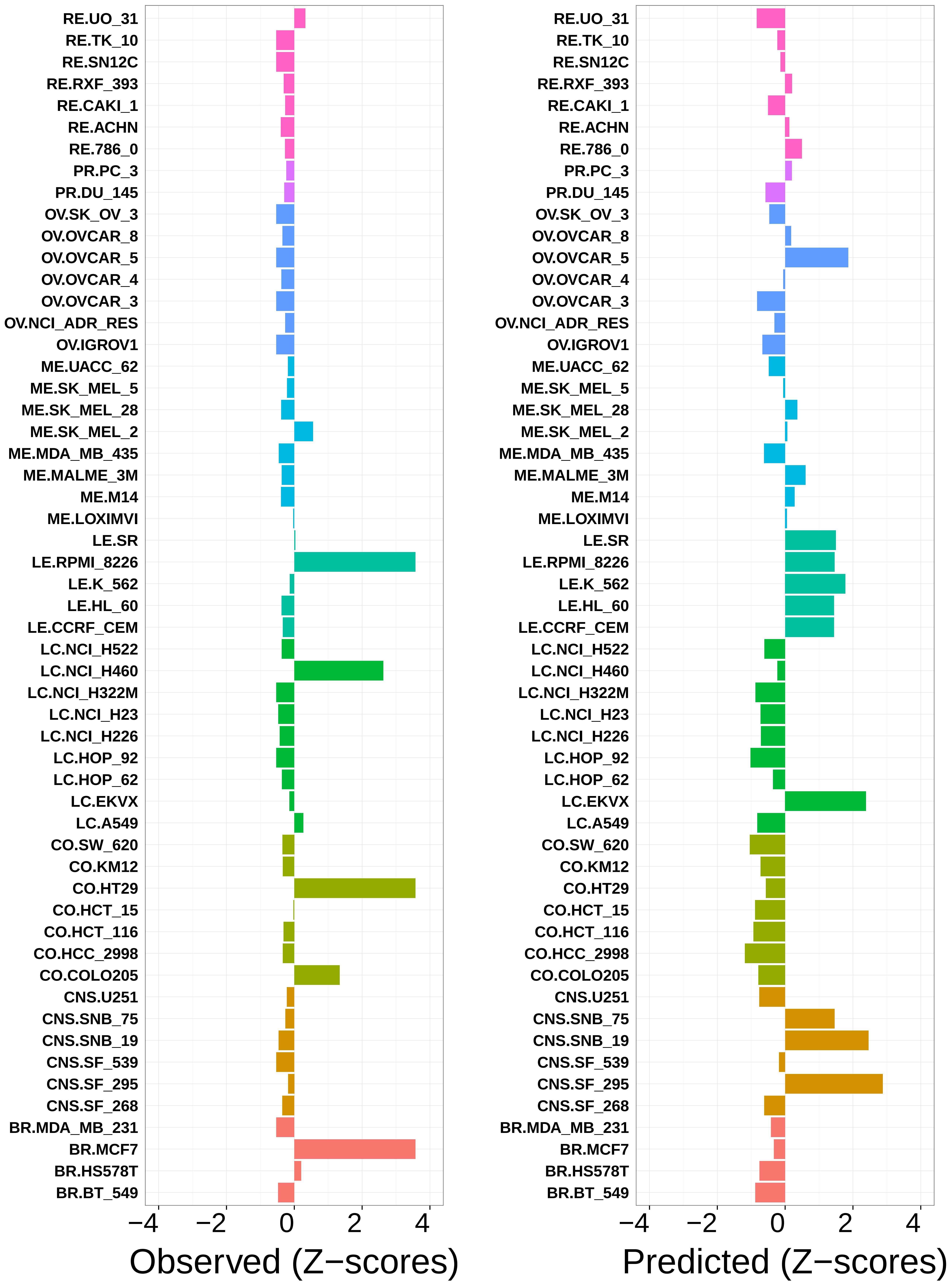

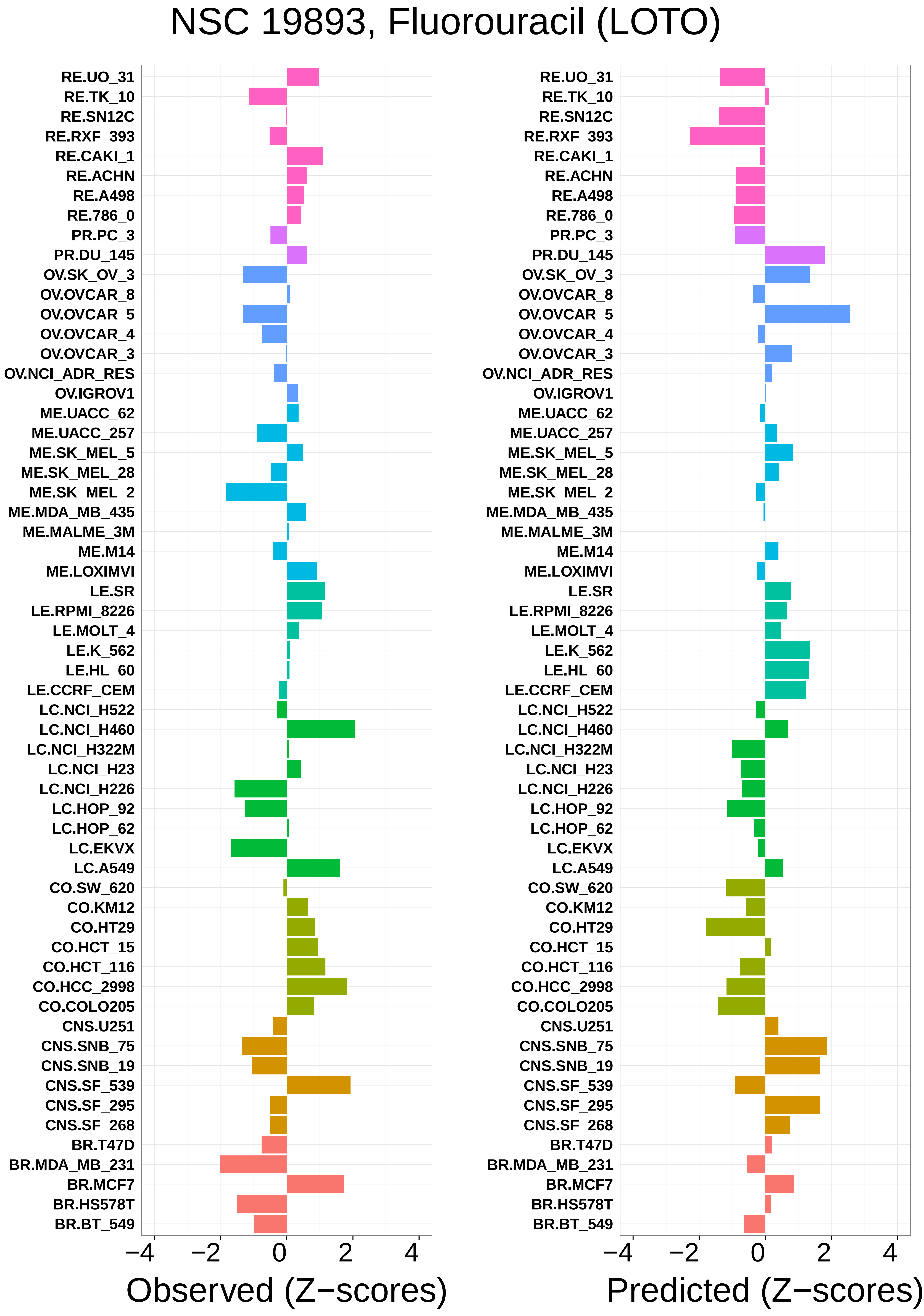
NSC 19893, Fluorouracil (LOTO)
RE.UO_31
RE.TK_10
RE.SN12C
RE.RXF_393
RE.CAKI_1
RE.ACHN
RE.A498
RE.786_0
PR.PC_3
PR.DU_145
OV.SK_OV_3
OV.OVCAR_8
OV.OVCAR_5
OV.OVCAR_4
OV.OVCAR_3
OV.NCI_ADR_RES
OV.IGROV1
ME.UACC_62
ME.UACC_257
ME.SK_MEL_5
ME.SK_MEL_28
ME.SK_MEL_2
ME.MDA_MB_435
ME.MALME_3M
ME.M14
ME.LOXIMVI
LE.SR
LE.RPMI_8226
LE.MOLT_4
LE.K_562
LE.HL_60
LE.CCRF_CEM
LC.NCI_H522
LC.NCI_H460
LC.NCI_H322M
LC.NCI_H23
LC.NCI_H226
LC.HOP_92
LC.HOP_62
LC.EKVX
LC.A549
CO.SW_620
CO.KM12
CO.HT29
CO.HCT_15
CO.HCT_116
CO.HCC_2998
CO.COLO205
CNS.U251
CNS.SNB_75
CNS.SNB_19
CNS.SF_539
CNS.SF_295
CNS.SF_268
BR.T47D
BR.MDA_MB_231
BR.MCF7
BR.HS578T
BR.BT_549
−4
−2
0
2
4
Observed (Z−scores)
Predicted (Z−scores)

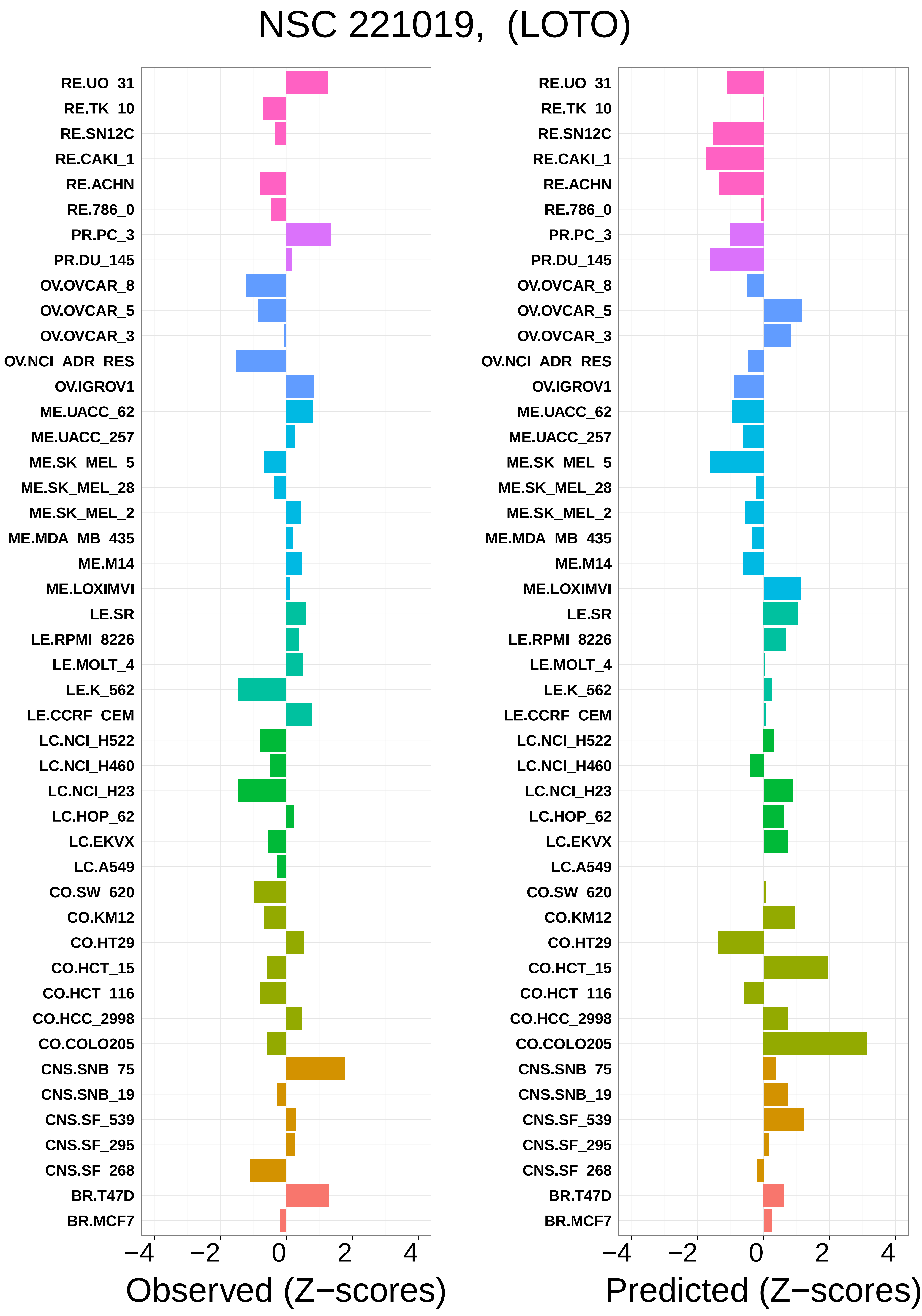
NSC 221019, (LOTO)
RE.UO_31
RE.TK_10
RE.SN12C
RE.CAKI_1
RE.ACHN
RE.786_0
PR.PC_3
PR.DU_145
OV.OVCAR_8
OV.OVCAR_5
OV.OVCAR_3
OV.NCI_ADR_RES
OV.IGROV1
ME.UACC_62
ME.UACC_257
ME.SK_MEL_5
ME.SK_MEL_28
ME.SK_MEL_2
ME.MDA_MB_435
ME.M14
ME.LOXIMVI
LE.SR
LE.RPMI_8226
LE.MOLT_4
LE.K_562
LE.CCRF_CEM
LC.NCI_H522
LC.NCI_H460
LC.NCI_H23
LC.HOP_62
LC.EKVX
LC.A549
CO.SW_620
CO.KM12
CO.HT29
CO.HCT_15
CO.HCT_116
CO.HCC_2998
CO.COLO205
CNS.SNB_75
CNS.SNB_19
CNS.SF_539
CNS.SF_295
CNS.SF_268
BR.T47D
BR.MCF7
−4 −2 0 2 4
Observed (Z−scores)
Predicted (Z−scores)

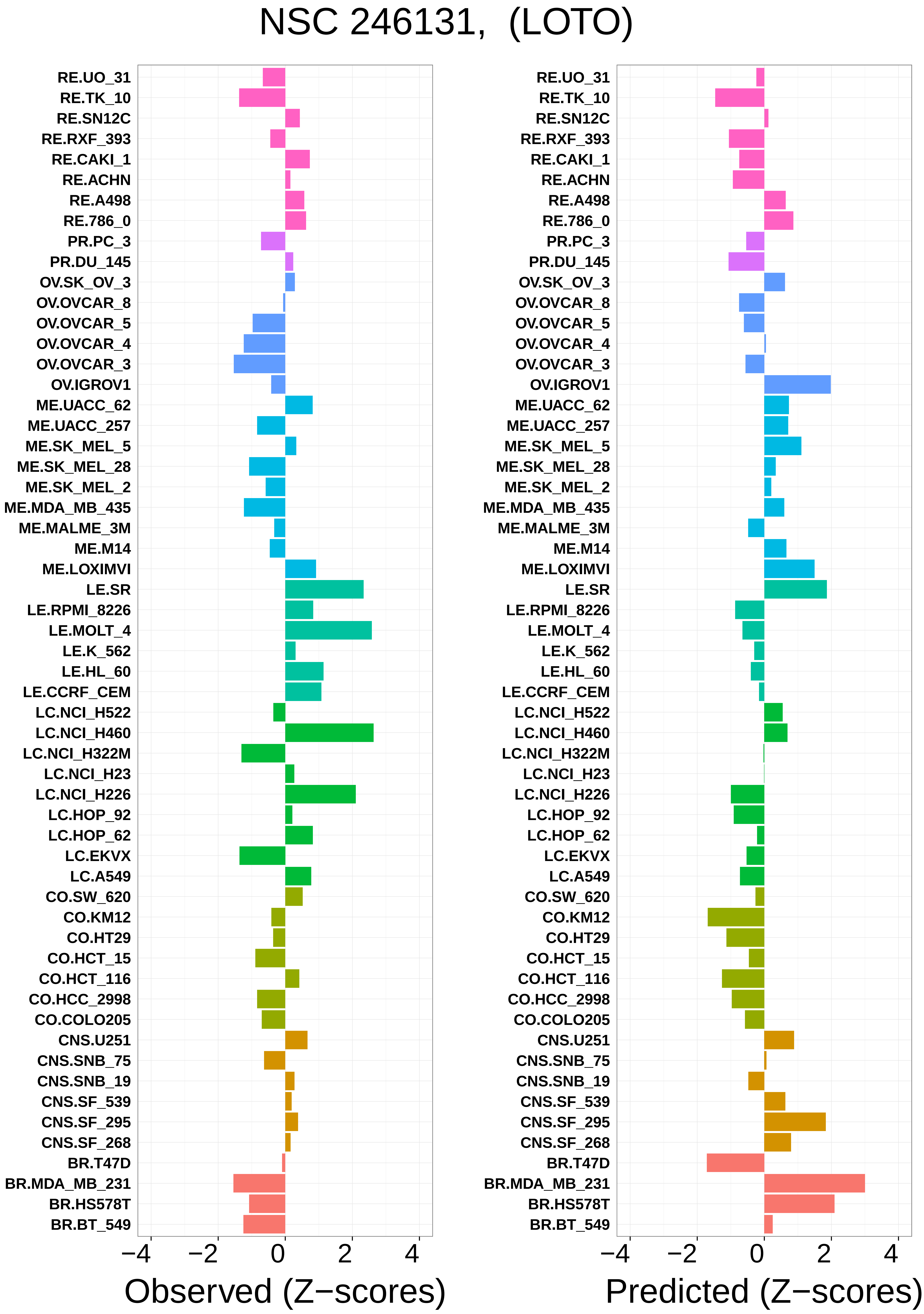
NSC 246131, (LOTO)
RE.UO_31
RE.TK_10
RE.SN12C
RE.RXF_393
RE.CAKI_1
RE.ACHN
RE.A498
RE.786_0
PR.PC_3
PR.DU_145
OV.SK_OV_3
OV.OVCAR_8
OV.OVCAR_5
OV.OVCAR_4
OV.OVCAR_3
OV.IGROV1
ME.UACC_62
ME.UACC_257
ME.SK_MEL_5
ME.SK_MEL_28
ME.SK_MEL_2
ME.MDA_MB_435
ME.MALME_3M
ME.M14
ME.LOXIMVI
LE.SR
LE.RPMI_8226
LE.MOLT_4
LE.K_562
LE.HL_60
LE.CCRF_CEM
LC.NCI_H522
LC.NCI_H460
LC.NCI_H322M
LC.NCI_H23
LC.NCI_H226
LC.HOP_92
LC.HOP_62
LC.EKVX
LC.A549
CO.SW_620
CO.KM12
CO.HT29
CO.HCT_15
CO.HCT_116
CO.HCC_2998
CO.COLO205
CNS.U251
CNS.SNB_75
CNS.SNB_19
CNS.SF_539
CNS.SF_295
CNS.SF_268
BR.T47D
BR.MDA_MB_231
BR.HS578T
BR.BT_549
−4 −2 0 2 4
Observed (Z−scores)
Predicted (Z−scores)

NSC 255109, (LOTO)

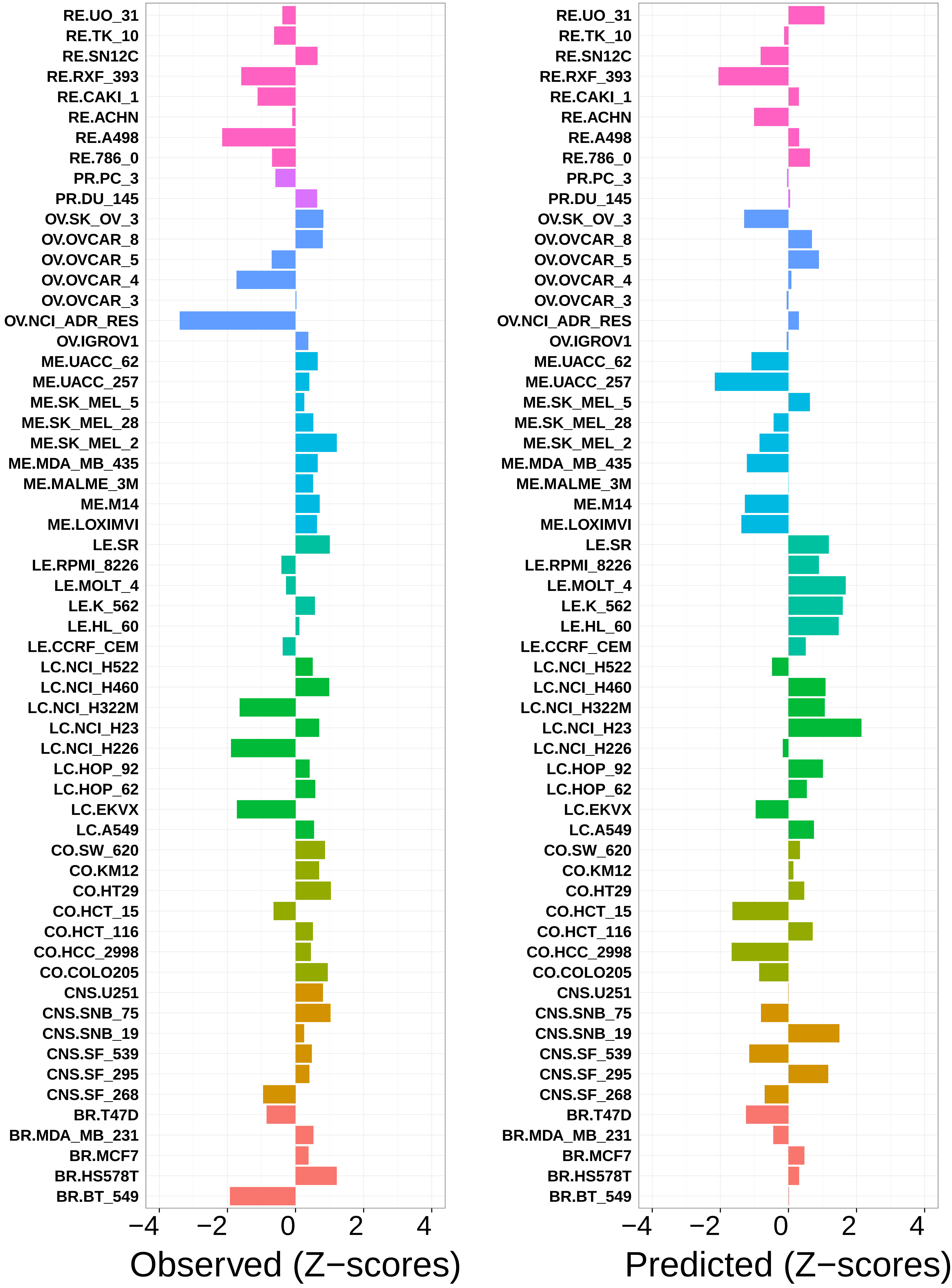

NSC 256942, (LOTO)

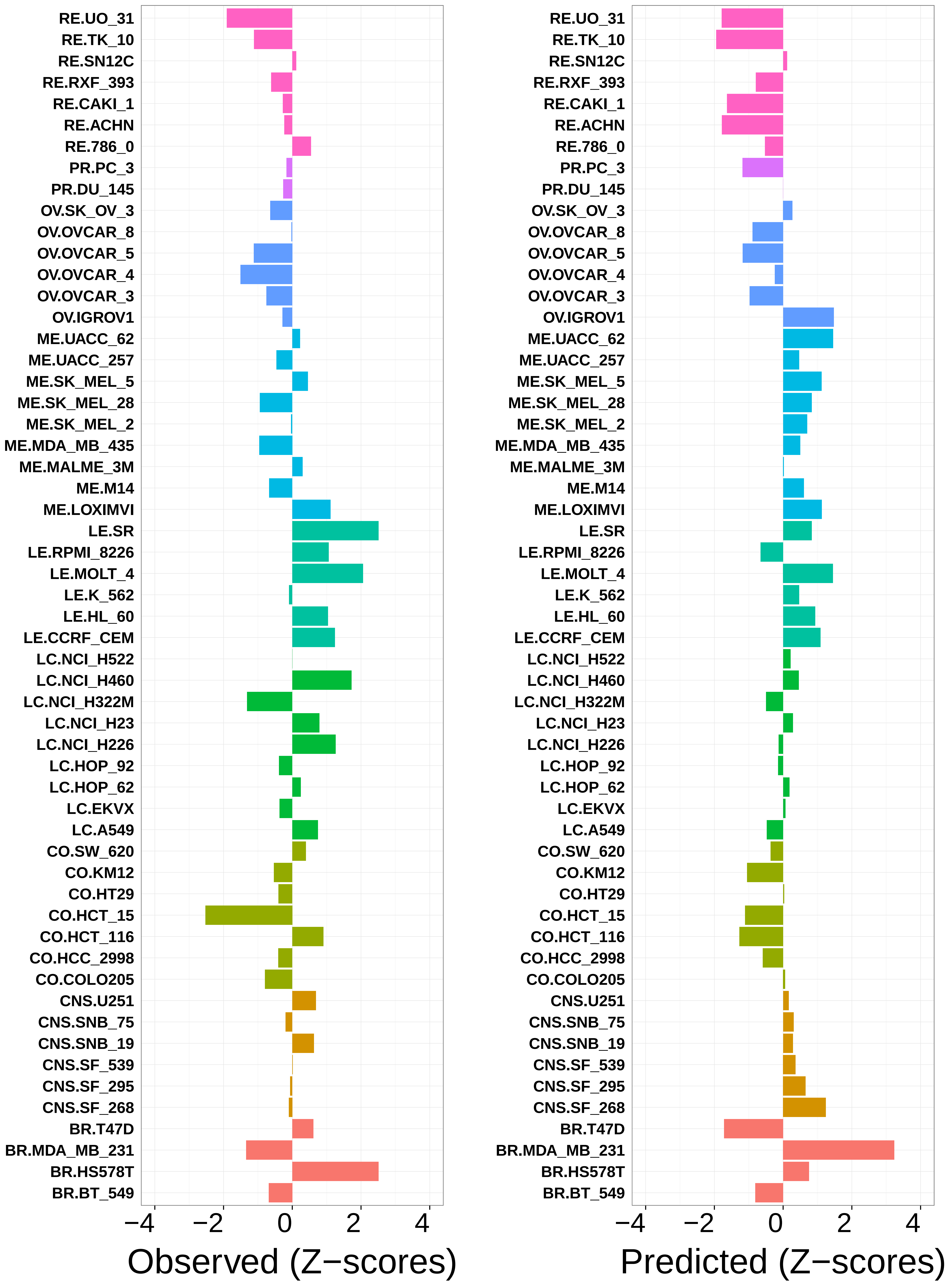

NSC 26980, Mitomycin (LOTO)

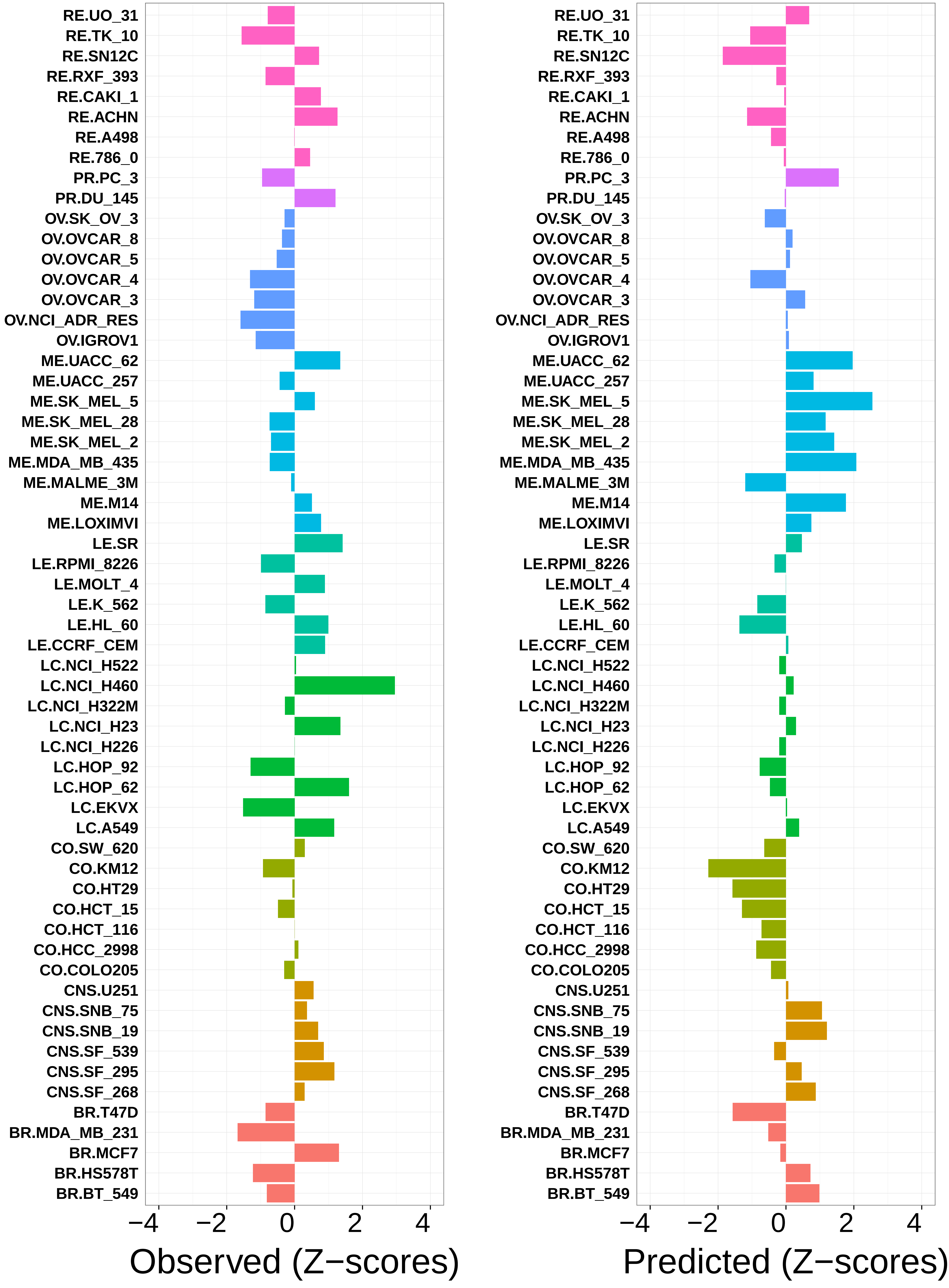

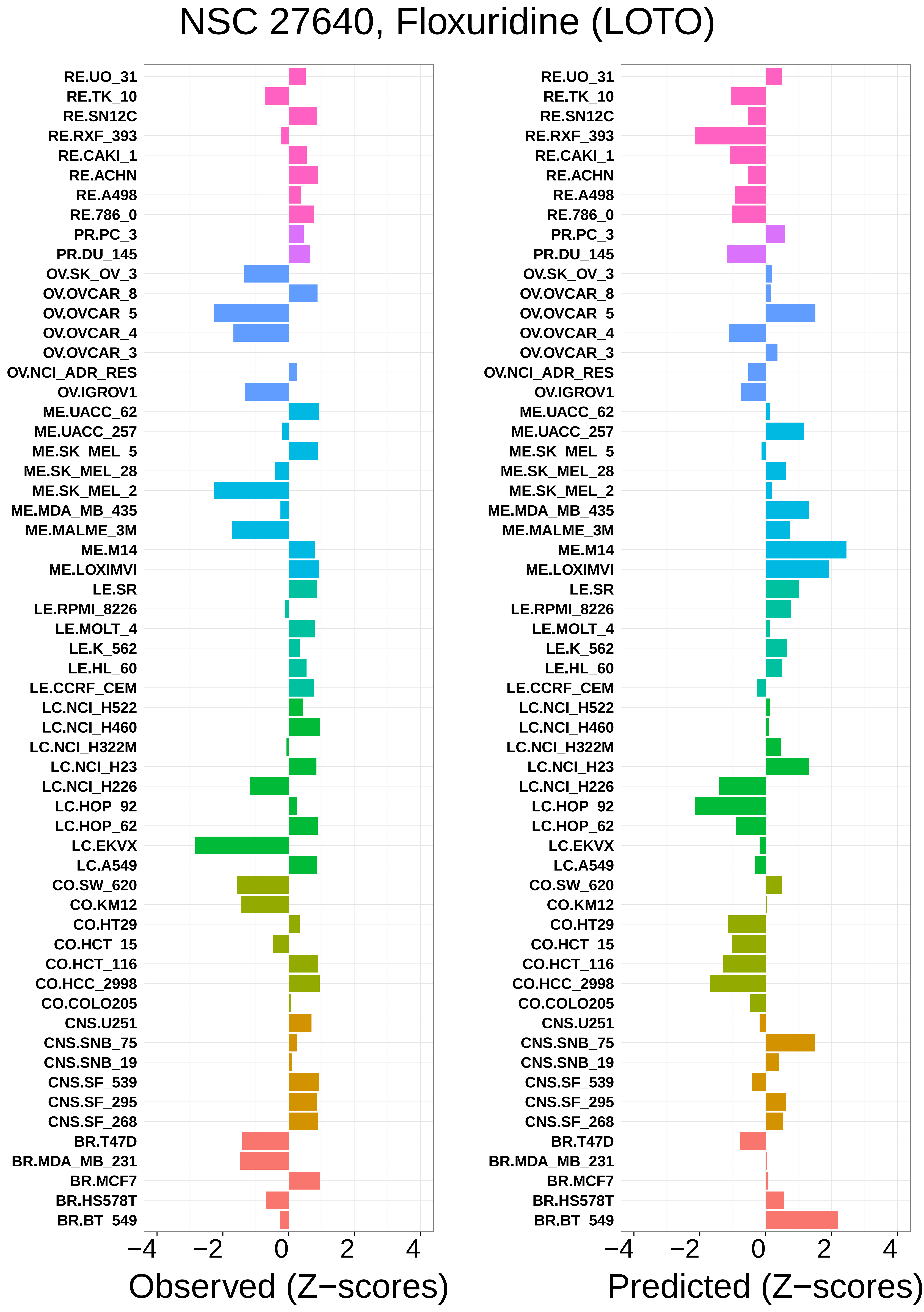

NSC 27640, Floxuridine (LOTO)
RE.UO_31
RE.TK_10
RE.SN12C
RE.RXF_393
RE.CAKI_1
RE.ACHN
RE.A498
RE.786_0
PR.PC_3
PR.DU_145
OV.SK_OV_3
OV.OVCAR_8
OV.OVCAR_5
OV.OVCAR_4
OV.OVCAR_3
OV.NCI_ADR_RES
OV.IGROV1
ME.UACC_62
ME.UACC_257
ME.SK_MEL_5
ME.SK_MEL_28
ME.SK_MEL_2
ME.MDA_MB_435
ME.MALME_3M
ME.M14
ME.LOXIMVI
LE.SR
LE.RPMI_8226
LE.MOLT_4
LE.K_562
LE.HL_60
LE.CCRF_CEM
LC.NCI_H522
LC.NCI_H460
LC.NCI_H322M
LC.NCI_H23
LC.NCI_H226
LC.HOP_92
LC.HOP_62
LC.EKVX
LC.A549
CO.SW_620
CO.KM12
CO.HT29
CO.HCT_15
CO.HCT_116
CO.HCC_2998
CO.COLO205
CNS.U251
CNS.SNB_75
CNS.SNB_19
CNS.SF_539
CNS.SF_295
CNS.SF_268
BR.T47D
BR.MDA_MB_231
BR.MCF7
BR.HS578T
BR.BT_549
−4 −2 0 2 4
Observed (Z−scores)
Predicted (Z−scores)

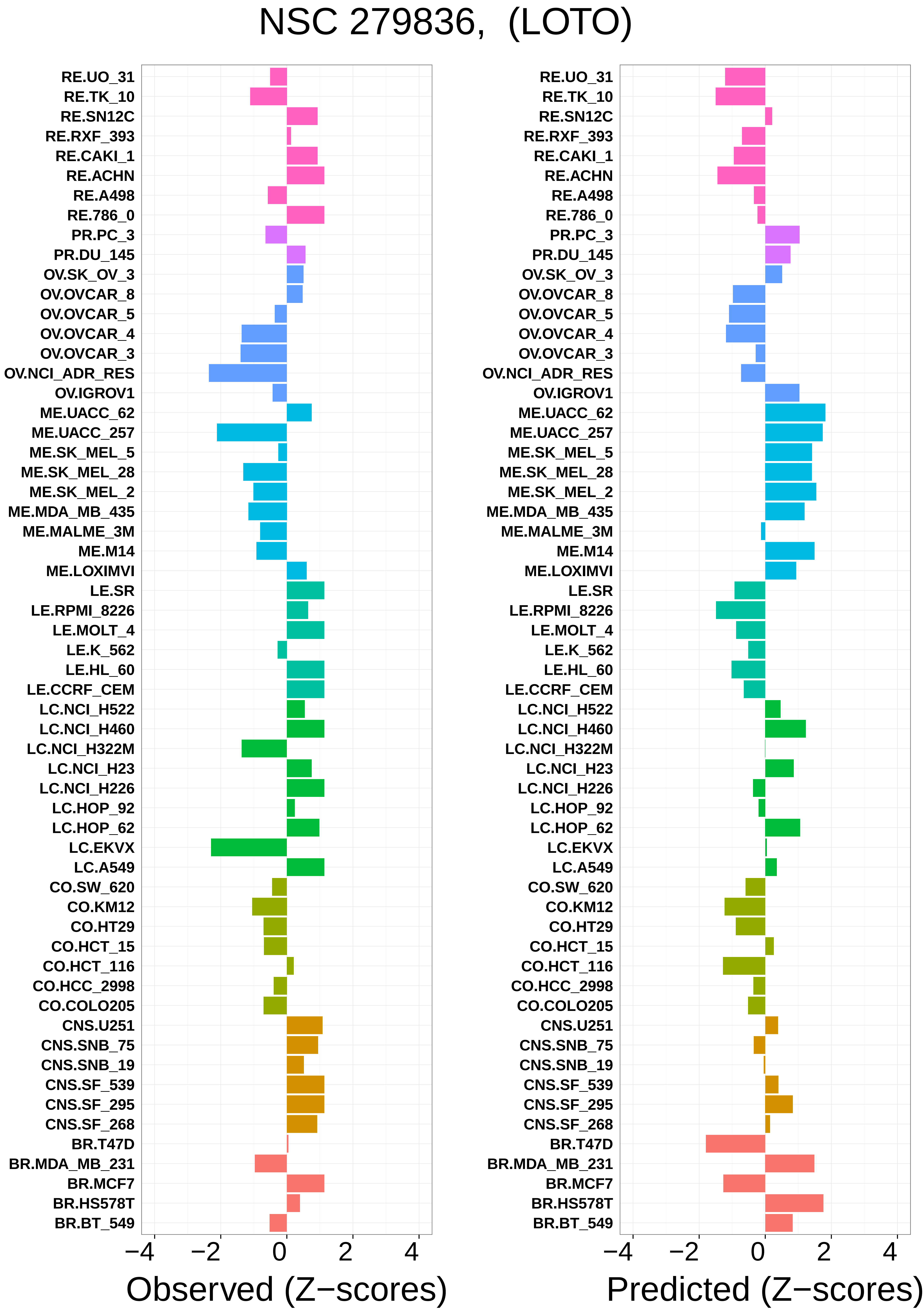
NSC 279836, (LOTO)
RE.UO_31
RE.TK_10
RE.SN12C
RE.RXF_393
RE.CAKI_1
RE.ACHN
RE.A498
RE.786_0
PR.PC_3
PR.DU_145
OV.SK_OV_3
OV.OVCAR_8
OV.OVCAR_5
OV.OVCAR_4
OV.OVCAR_3
OV.NCI_ADR_RES
OV.IGROV1
ME.UACC_62
ME.UACC_257
ME.SK_MEL_5
ME.SK_MEL_28
ME.SK_MEL_2
ME.MDA_MB_435
ME.MALME_3M
ME.M14
ME.LOXIMVI
LE.SR
LE.RPMI_8226
LE.MOLT_4
LE.K_562
LE.HL_60
LE.CCRF_CEM
LC.NCI_H522
LC.NCI_H460
LC.NCI_H322M
LC.NCI_H23
LC.NCI_H226
LC.HOP_92
LC.HOP_62
LC.EKVX
LC.A549
CO.SW_620
CO.KM12
CO.HT29
CO.HCT_15
CO.HCT_116
CO.HCC_2998
CO.COLO205
CNS.U251
CNS.SNB_75
CNS.SNB_19
CNS.SF_539
CNS.SF_295
CNS.SF_268
BR.T47D
BR.MDA_MB_231
BR.MCF7
BR.HS578T
BR.BT_549
−4
−2
0
2
4
Observed (Z−scores)
Predicted (Z−scores)

NSC 280594, (LOTO)

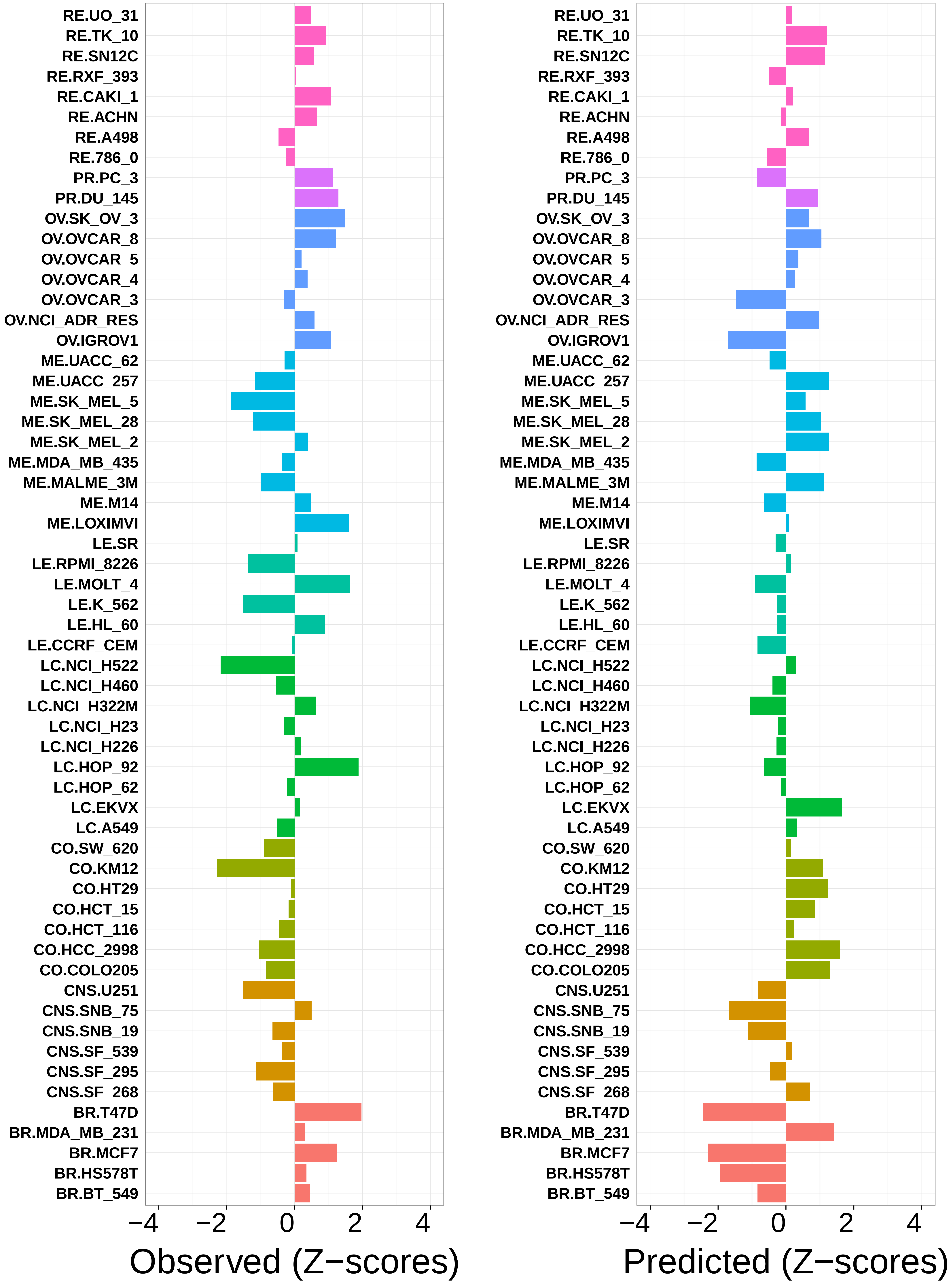

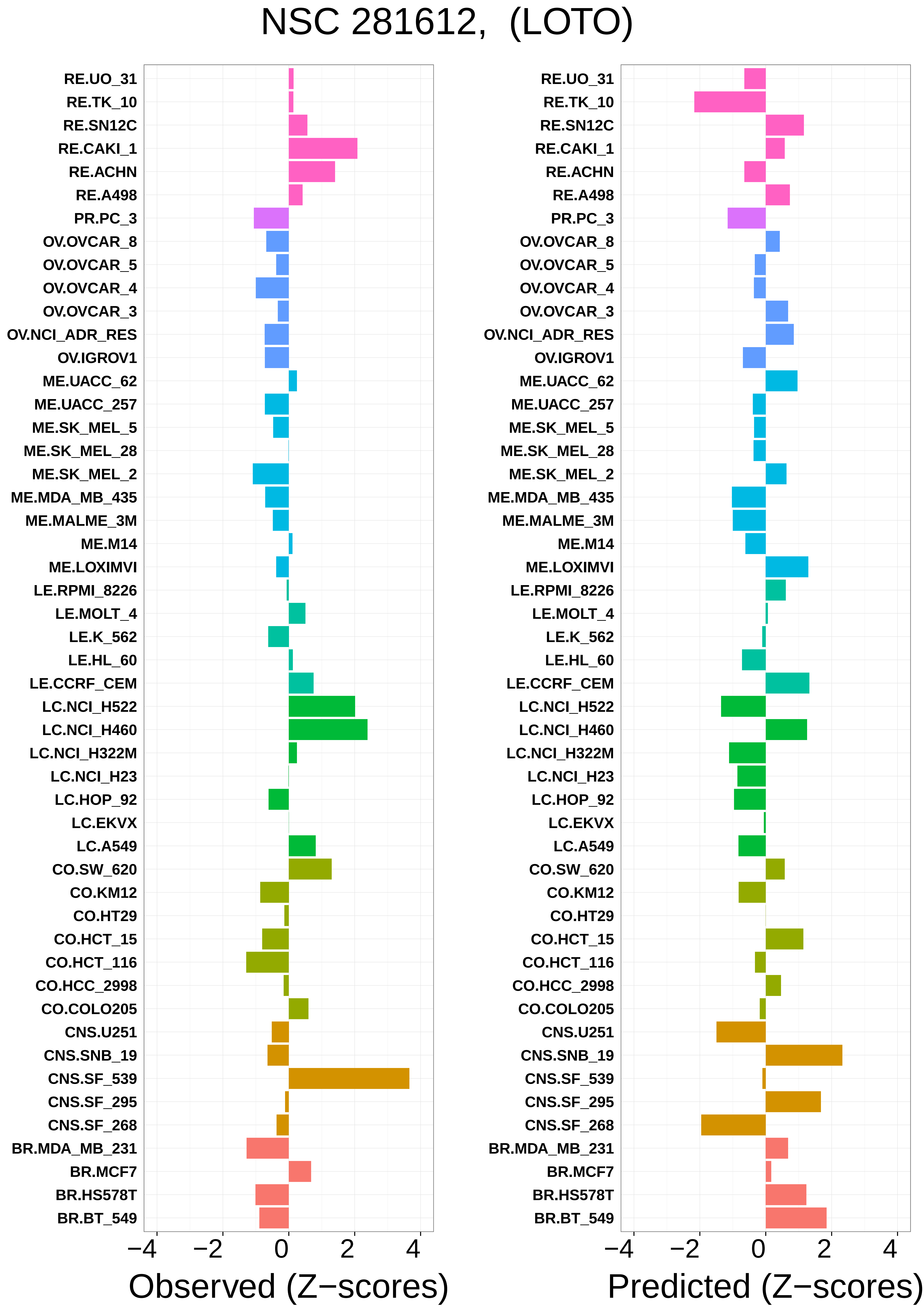
NSC 281612, (LOTO)
RE.UO_31
RE.TK_10
RE.SN12C
RE.CAKI_1
RE.ACHN
RE.A498
PR.PC_3
OV.OVCAR_8
OV.OVCAR_5
OV.OVCAR_4
OV.OVCAR_3
OV.NCI_ADR_RES
OV.IGROV1
ME.UACC_62
ME.UACC_257
ME.SK_MEL_5
ME.SK_MEL_28
ME.SK_MEL_2
ME.MDA_MB_435
ME.MALME_3M
ME.M14
ME.LOXIMVI
LE.RPMI_8226
LE.MOLT_4
LE.K_562
LE.HL_60
LE.CCRF_CEM
LC.NCI_H522
LC.NCI_H460
LC.NCI_H322M
LC.NCI_H23
LC.HOP_92
LC.EKVX
LC.A549
CO.SW_620
CO.KM12
CO.HT29
CO.HCT_15
CO.HCT_116
CO.HCC_2998
CO.COLO205
CNS.U251
CNS.SNB_19
CNS.SF_539
CNS.SF_295
CNS.SF_268
BR.MDA_MB_231
BR.MCF7
BR.HS578T
BR.BT_549
−4 −2 0 2 4
Observed (Z−scores)
Predicted (Z−scores)

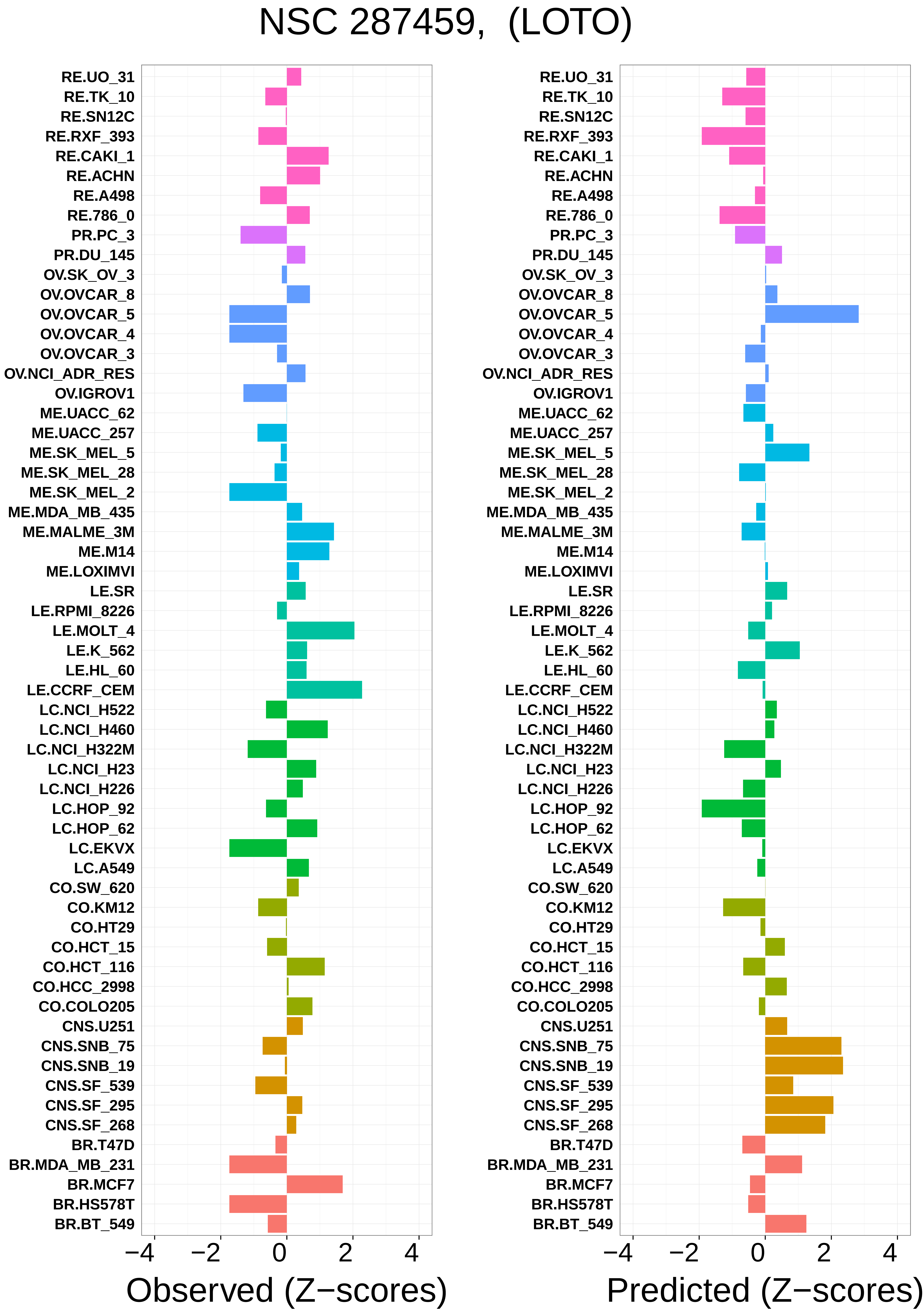
NSC 287459, (LOTO)
RE.UO_31
RE.TK_10
RE.SN12C
RE.RXF_393
RE.CAKI_1
RE.ACHN
RE.A498
RE.786_0
PR.PC_3
PR.DU_145
OV.SK_OV_3
OV.OVCAR_8
OV.OVCAR_5
OV.OVCAR_4
OV.OVCAR_3
OV.NCI_ADR_RES
OV.IGROV1
ME.UACC_62
ME.UACC_257
ME.SK_MEL_5
ME.SK_MEL_28
ME.SK_MEL_2
ME.MDA_MB_435
ME.MALME_3M
ME.M14
ME.LOXIMVI
LE.SR
LE.RPMI_8226
LE.MOLT_4
LE.K_562
LE.HL_60
LE.CCRF_CEM
LC.NCI_H522
LC.NCI_H460
LC.NCI_H322M
LC.NCI_H23
LC.NCI_H226
LC.HOP_92
LC.HOP_62
LC.EKVX
LC.A549
CO.SW_620
CO.KM12
CO.HT29
CO.HCT_15
CO.HCT_116
CO.HCC_2998
CO.COLO205
CNS.U251
CNS.SNB_75
CNS.SNB_19
CNS.SF_539
CNS.SF_295
CNS.SF_268
BR.T47D
BR.MDA_MB_231
BR.MCF7
BR.HS578T
BR.BT_549
−4 −2 0 2 4
Observed (Z−scores)
Predicted (Z−scores)

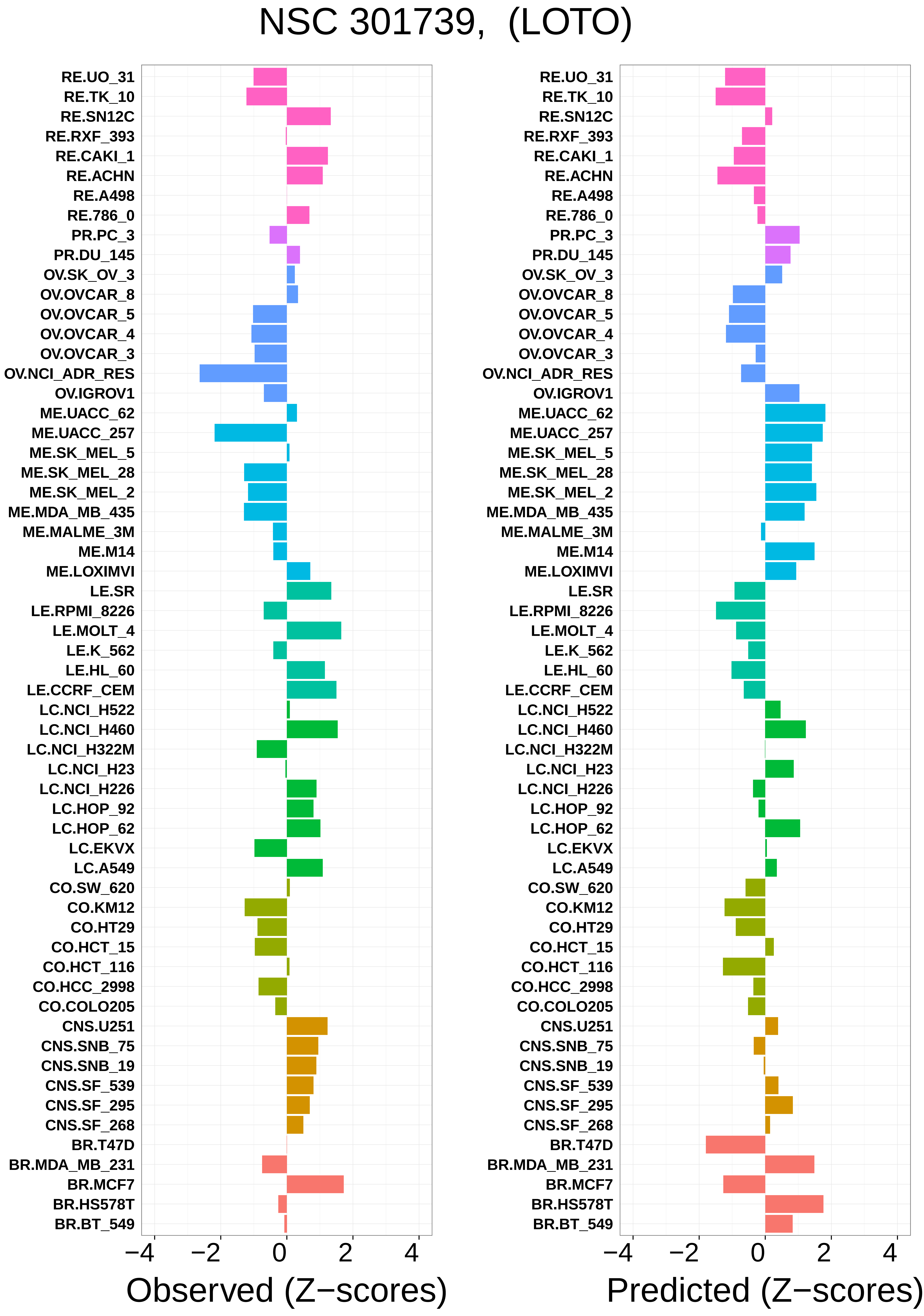

NSC 301739, (LOTO)
RE.UO_31
RE.TK_10
RE.SN12C
RE.RXF_393
RE.CAKI_1
RE.ACHN
RE.A498
RE.786_0
PR.PC_3
PR.DU_145
OV.SK_OV_3
OV.OVCAR_8
OV.OVCAR_5
OV.OVCAR_4
OV.OVCAR_3
OV.NCI_ADR_RES
OV.IGROV1
ME.UACC_62
ME.UACC_257
ME.SK_MEL_5
ME.SK_MEL_28
ME.SK_MEL_2
ME.MDA_MB_435
ME.MALME_3M
ME.M14
ME.LOXIMVI
LE.SR
LE.RPMI_8226
LE.MOLT_4
LE.K_562
LE.HL_60
LE.CCRF_CEM
LC.NCI_H522
LC.NCI_H460
LC.NCI_H322M
LC.NCI_H23
LC.NCI_H226
LC.HOP_92
LC.HOP_62
LC.EKVX
LC.A549
CO.SW_620
CO.KM12
CO.HT29
CO.HCT_15
CO.HCT_116
CO.HCC_2998
CO.COLO205
CNS.U251
CNS.SNB_75
CNS.SNB_19
CNS.SF_539
CNS.SF_295
CNS.SF_268
BR.T47D
BR.MDA_MB_231
BR.MCF7
BR.HS578T
BR.BT_549
−4
−2
0
2
4
Observed (Z−scores)
Predicted (Z−scores)

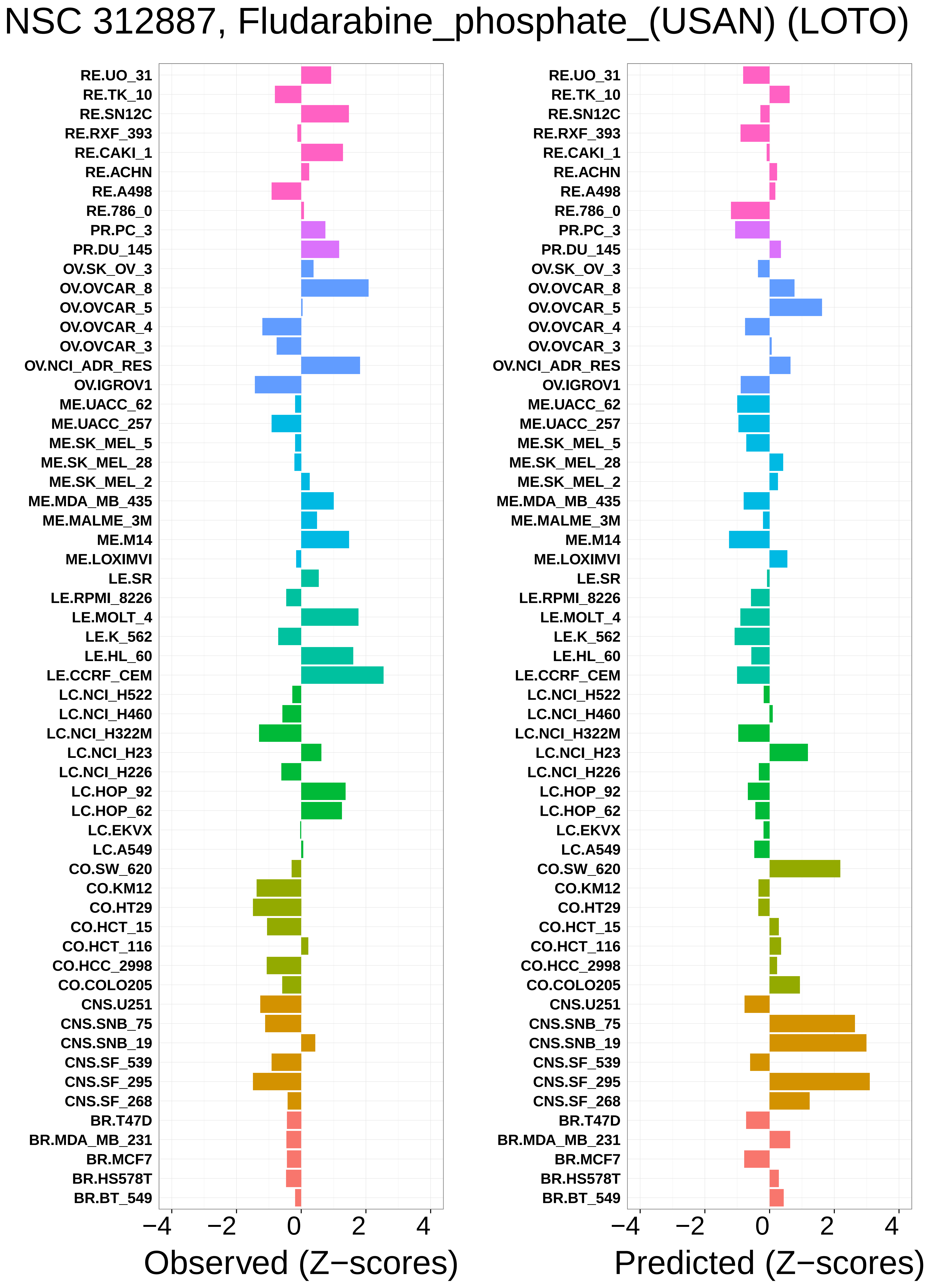
NSC 312887, Fludarabine_phosphate_(USAN) (LOTO)
RE.UO_31
RE.TK_10
RE.SN12C
RE.RXF_393
RE.CAKI_1
RE.ACHN
RE.A498
RE.786_0
PR.PC_3
PR.DU_145
OV.SK_OV_3
OV.OVCAR_8
OV.OVCAR_5
OV.OVCAR_4
OV.OVCAR_3
OV.NCI_ADR_RES
OV.IGROV1
ME.UACC_62
ME.UACC_257
ME.SK_MEL_5
ME.SK_MEL_28
ME.SK_MEL_2
ME.MDA_MB_435
ME.MALME_3M
ME.M14
ME.LOXIMVI
LE.SR
LE.RPMI_8226
LE.MOLT_4
LE.K_562
LE.HL_60
LE.CCRF_CEM
LC.NCI_H522
LC.NCI_H460
LC.NCI_H322M
LC.NCI_H23
LC.NCI_H226
LC.HOP_92
LC.HOP_62
LC.EKVX
LC.A549
CO.SW_620
CO.KM12
CO.HT29
CO.HCT_15
CO.HCT_116
CO.HCC_2998
CO.COLO205
CNS.U251
CNS.SNB_75
CNS.SNB_19
CNS.SF_539
CNS.SF_295
CNS.SF_268
BR.T47D
BR.MDA_MB_231
BR.MCF7
BR.HS578T
BR.BT_549
−4 −2 0 2 4
Observed (Z−scores)
−4 −2 0 2 4
Predicted (Z−scores)

NSC 330507, Tanespimycin (LOTO)

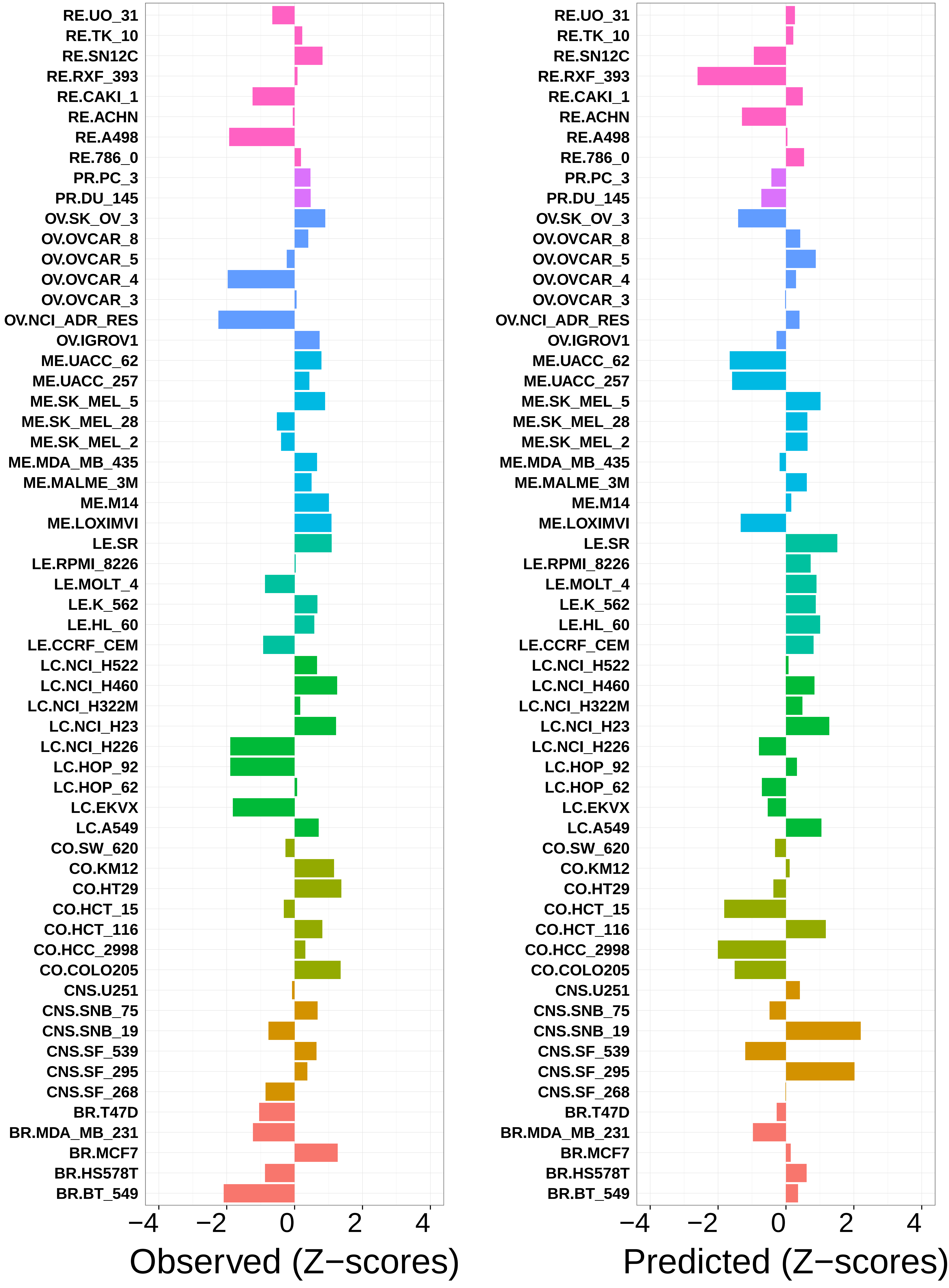

NSC 34521, Dextromethorphan_Hydrobromide (LOTO)

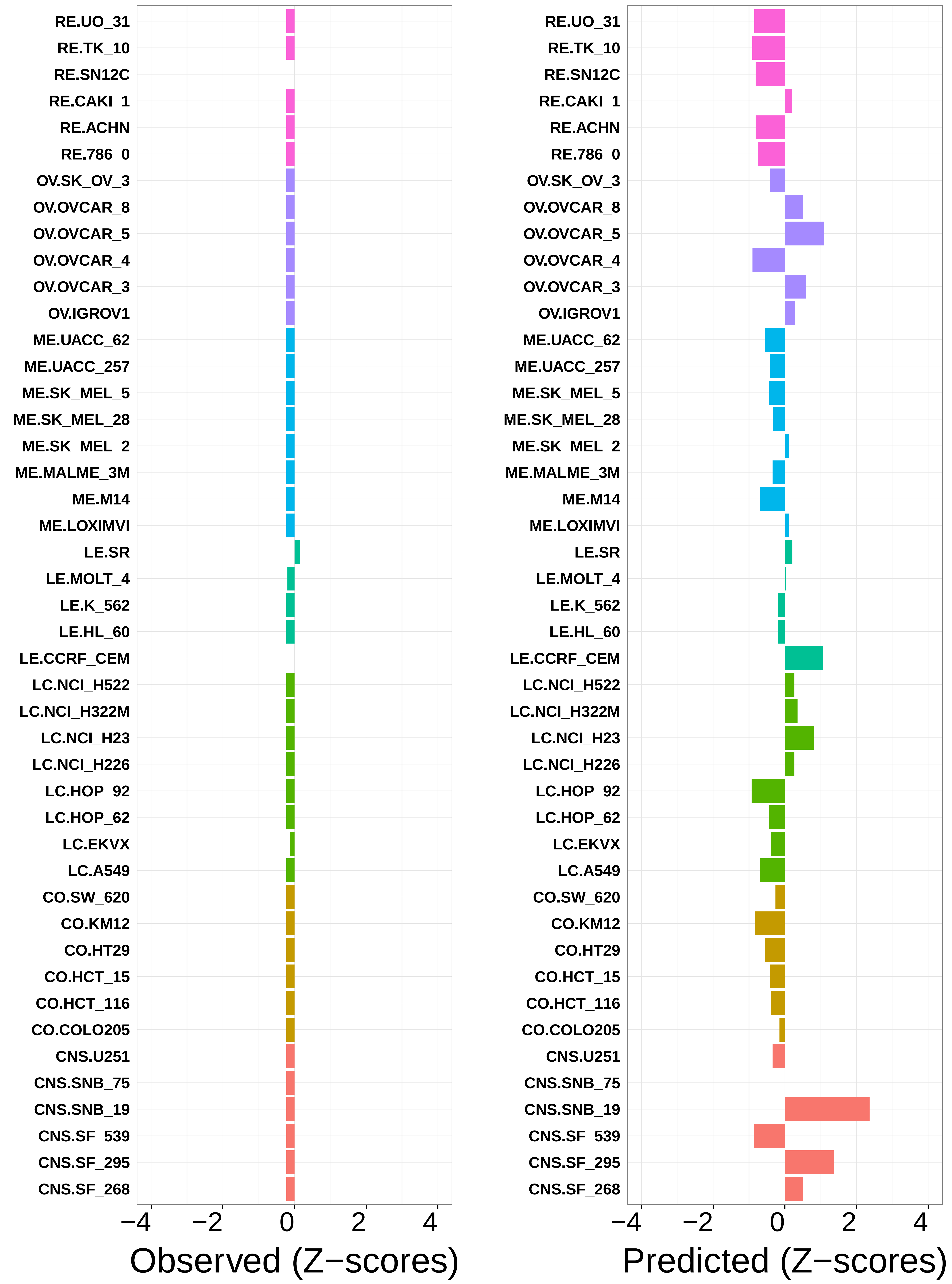

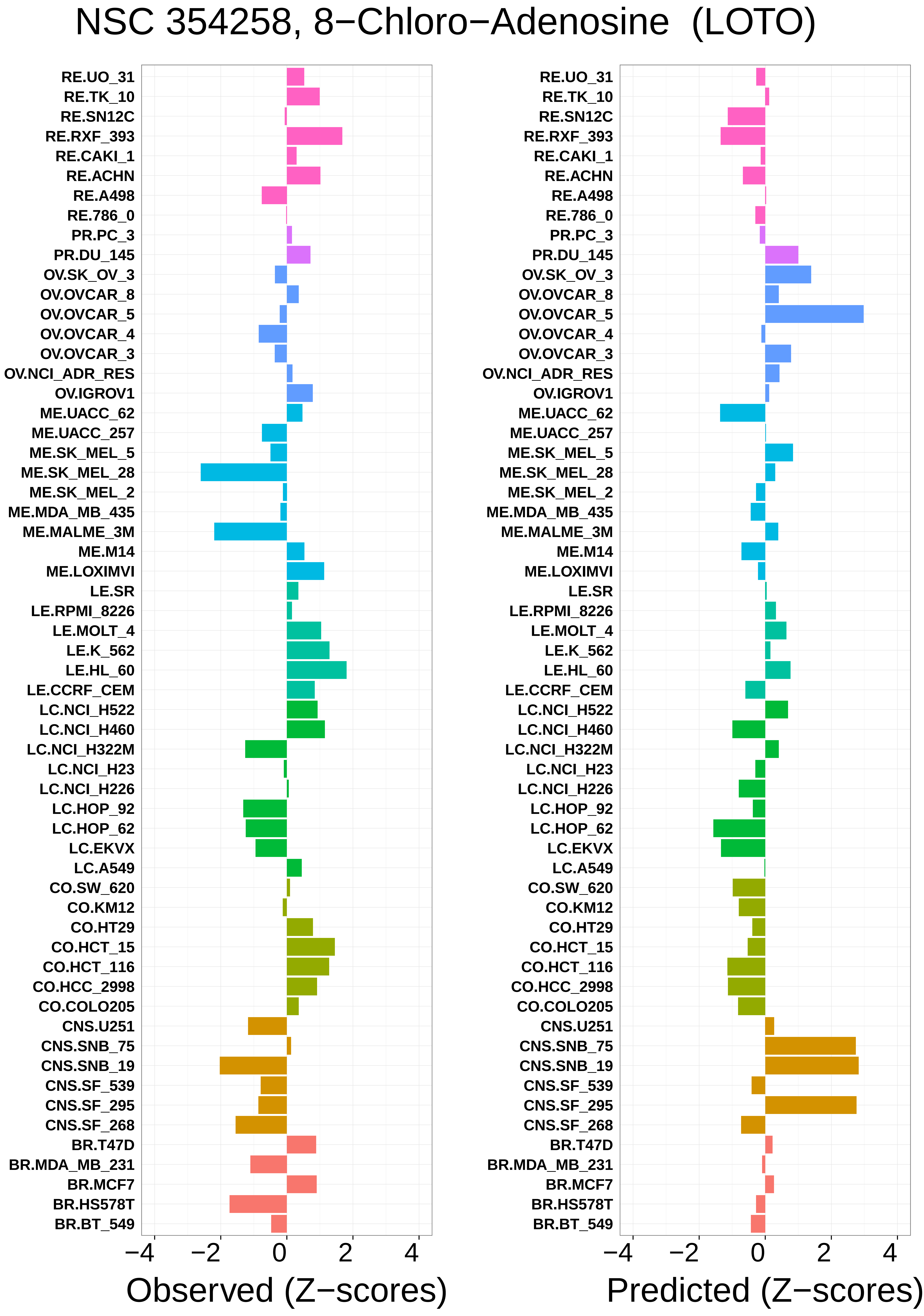
NSC 354258, 8−Chloro−Adenosine (LOTO)
RE.UO_31
RE.TK_10
RE.SN12C
RE.RXF_393
RE.CAKI_1
RE.ACHN
RE.A498
RE.786_0
PR.PC_3
PR.DU_145
OV.SK_OV_3
OV.OVCAR_8
OV.OVCAR_5
OV.OVCAR_4
OV.OVCAR_3
OV.NCI_ADR_RES
OV.IGROV1
ME.UACC_62
ME.UACC_257
ME.SK_MEL_5
ME.SK_MEL_28
ME.SK_MEL_2
ME.MDA_MB_435
ME.MALME_3M
ME.M14
ME.LOXIMVI
LE.SR
LE.RPMI_8226
LE.MOLT_4
LE.K_562
LE.HL_60
LE.CCRF_CEM
LC.NCI_H522
LC.NCI_H460
LC.NCI_H322M
LC.NCI_H23
LC.NCI_H226
LC.HOP_92
LC.HOP_62
LC.EKVX
LC.A549
CO.SW_620
CO.KM12
CO.HT29
CO.HCT_15
CO.HCT_116
CO.HCC_2998
CO.COLO205
CNS.U251
CNS.SNB_75
CNS.SNB_19
CNS.SF_539
CNS.SF_295
CNS.SF_268
BR.T47D
BR.MDA_MB_231
BR.MCF7
BR.HS578T
BR.BT_549
−4 −2 0 2 4
Observed (Z−scores)
−4 −2 0 2 4
Predicted (Z−scores)

NSC 354462, Hypothemycin (LOTO)

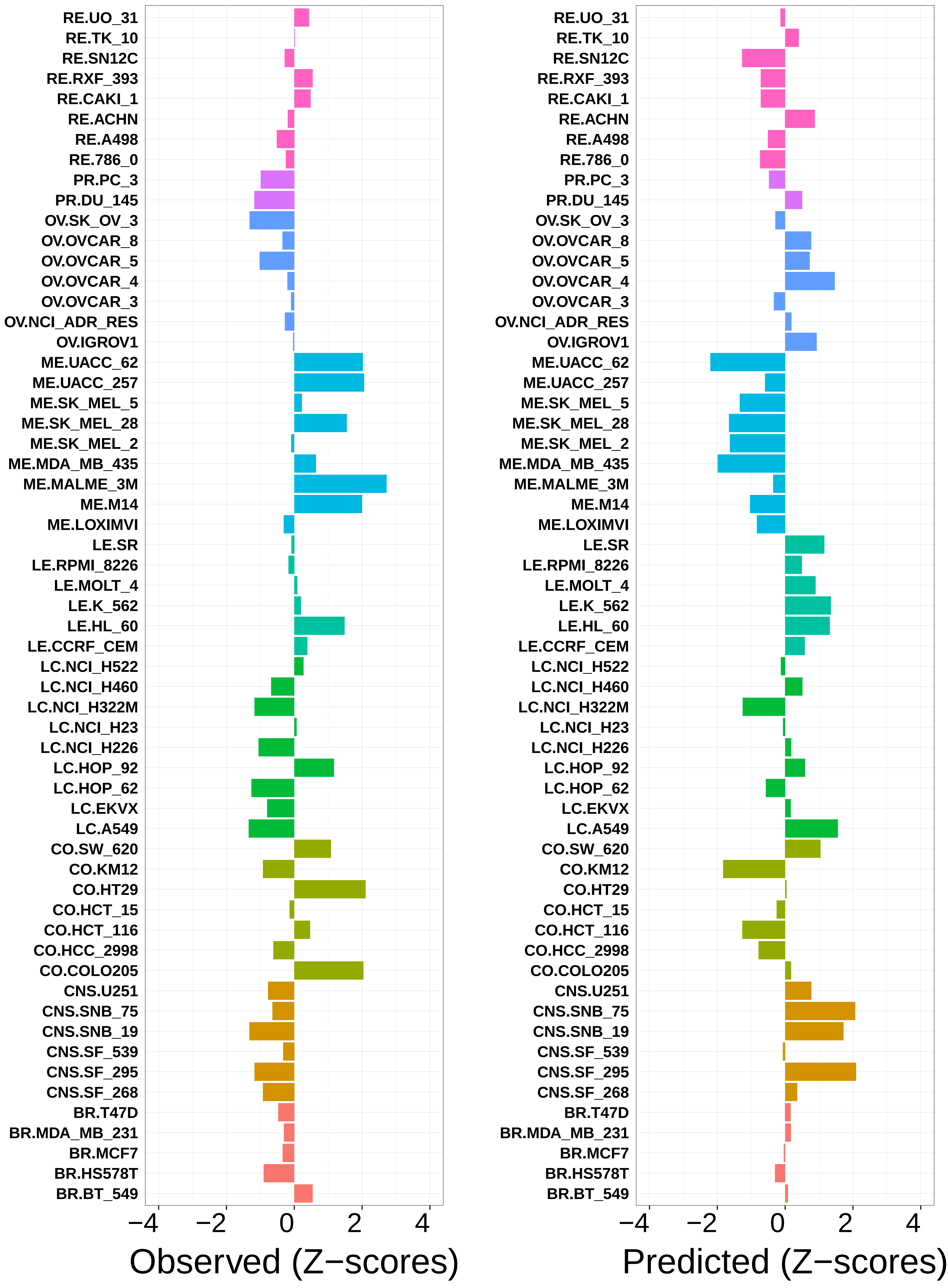

NSC 376128, Dolastatin_10 (LOTO)

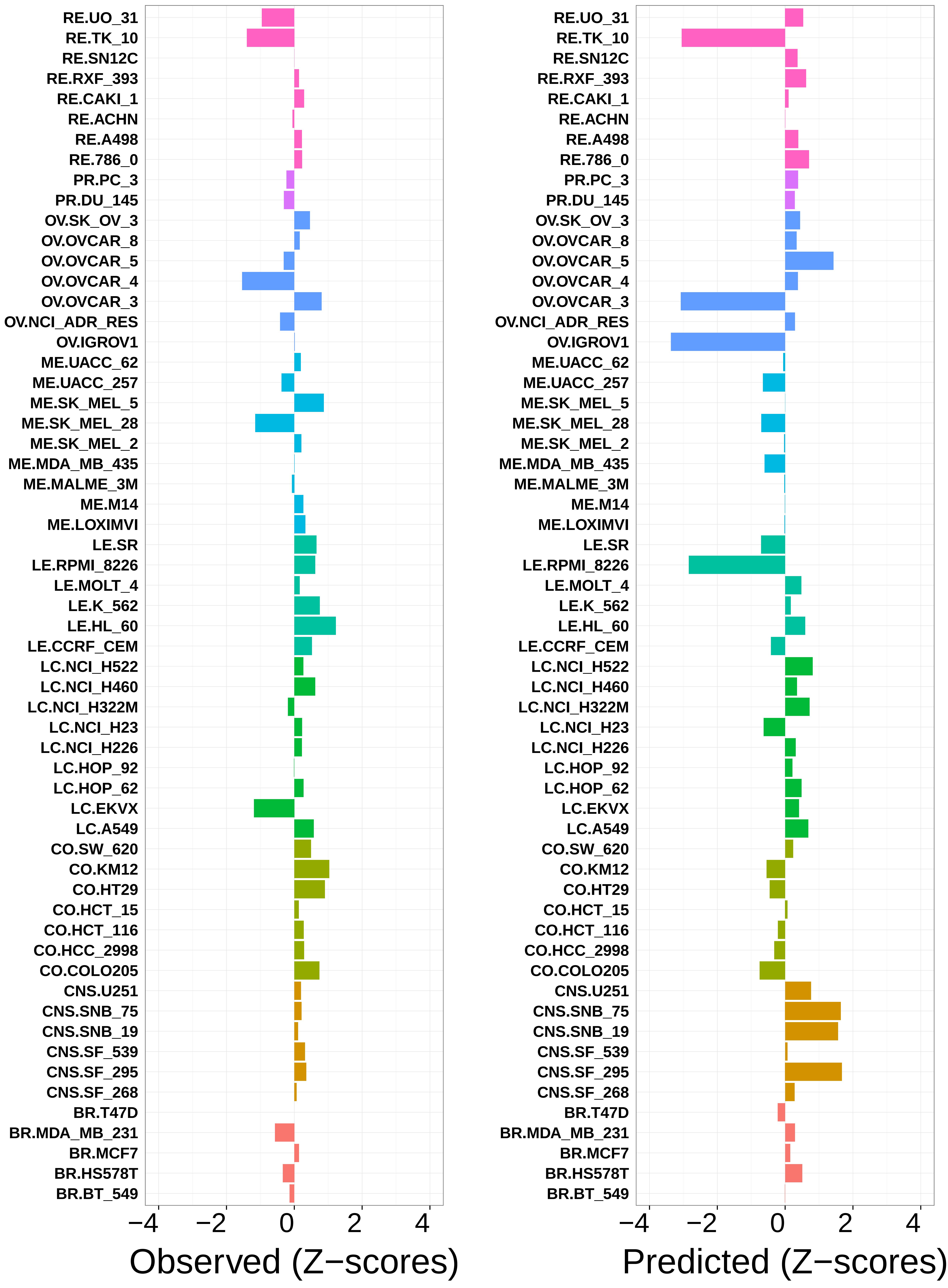

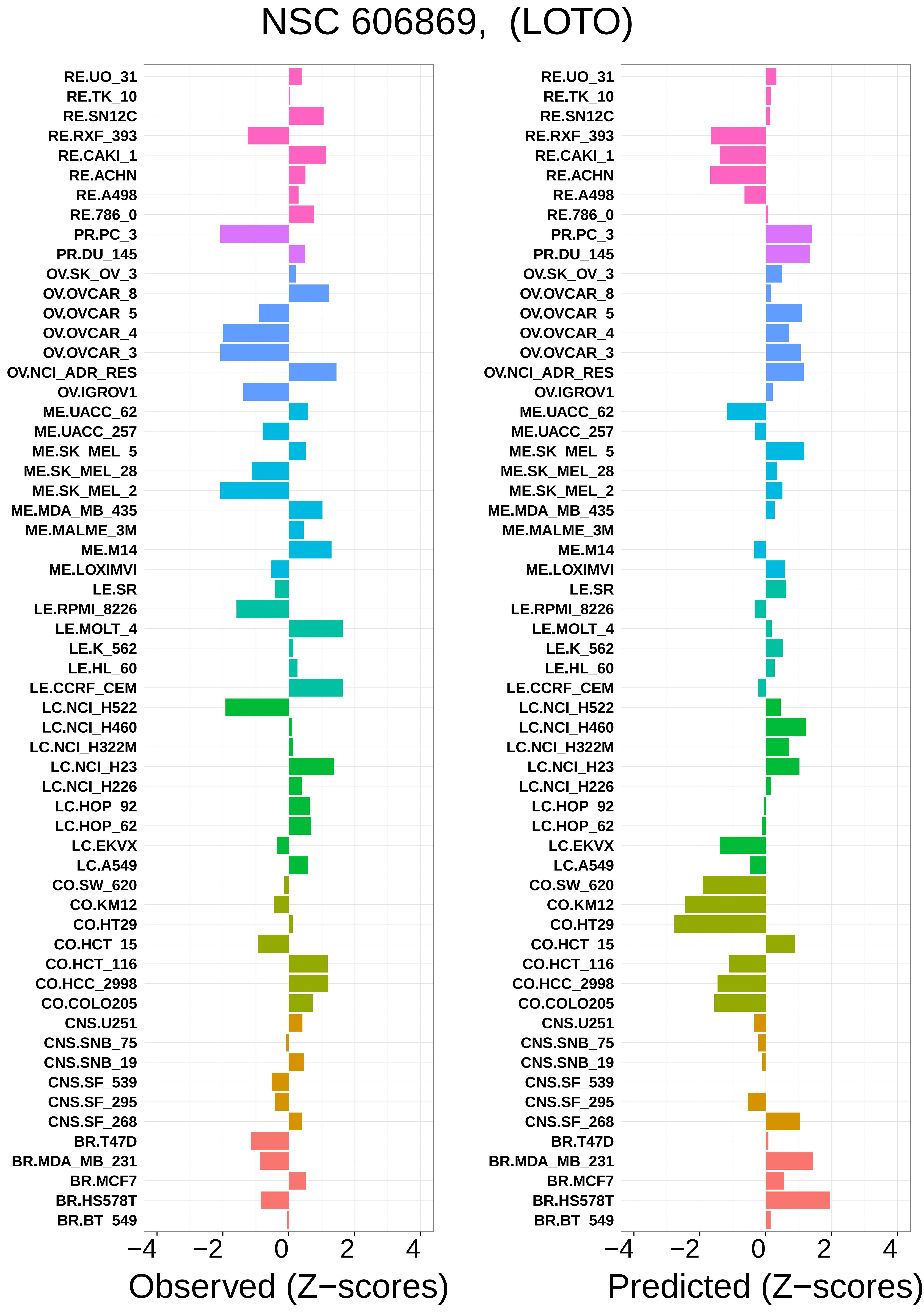

NSC 606869, (LOTO)
RE.UO_31
RE.TK_10
RE.SN12C
RE.RXF_393
RE.CAKI_1
RE.ACHN
RE.A498
RE.786_0
PR.PC_3
PR.DU_145
OV.SK_OV_3
OV.OVCAR_8
OV.OVCAR_5
OV.OVCAR_4
OV.OVCAR_3
OV.NCI_ADR_RES
OV.IGROV1
ME.UACC_62
ME.UACC_257
ME.SK_MEL_5
ME.SK_MEL_28
ME.SK_MEL_2
ME.MDA_MB_435
ME.MALME_3M
ME.M14
ME.LOXIMVI
LE.SR
LE.RPMI_8226
LE.MOLT_4
LE.K_562
LE.HL_60
LE.CCRF_CEM
LC.NCI_H522
LC.NCI_H460
LC.NCI_H322M
LC.NCI_H23
LC.NCI_H226
LC.HOP_92
LC.HOP_62
LC.EKVX
LC.A549
CO.SW_620
CO.KM12
CO.HT29
CO.HCT_15
CO.HCT_116
CO.HCC_2998
CO.COLO205
CNS.U251
CNS.SNB_75
CNS.SNB_19
CNS.SF_539
CNS.SF_295
CNS.SF_268
BR.T47D
BR.MDA_MB_231
BR.MCF7
BR.HS578T
BR.BT_549
−4 −2 0 2 4
Observed (Z−scores)
Predicted (Z−scores)

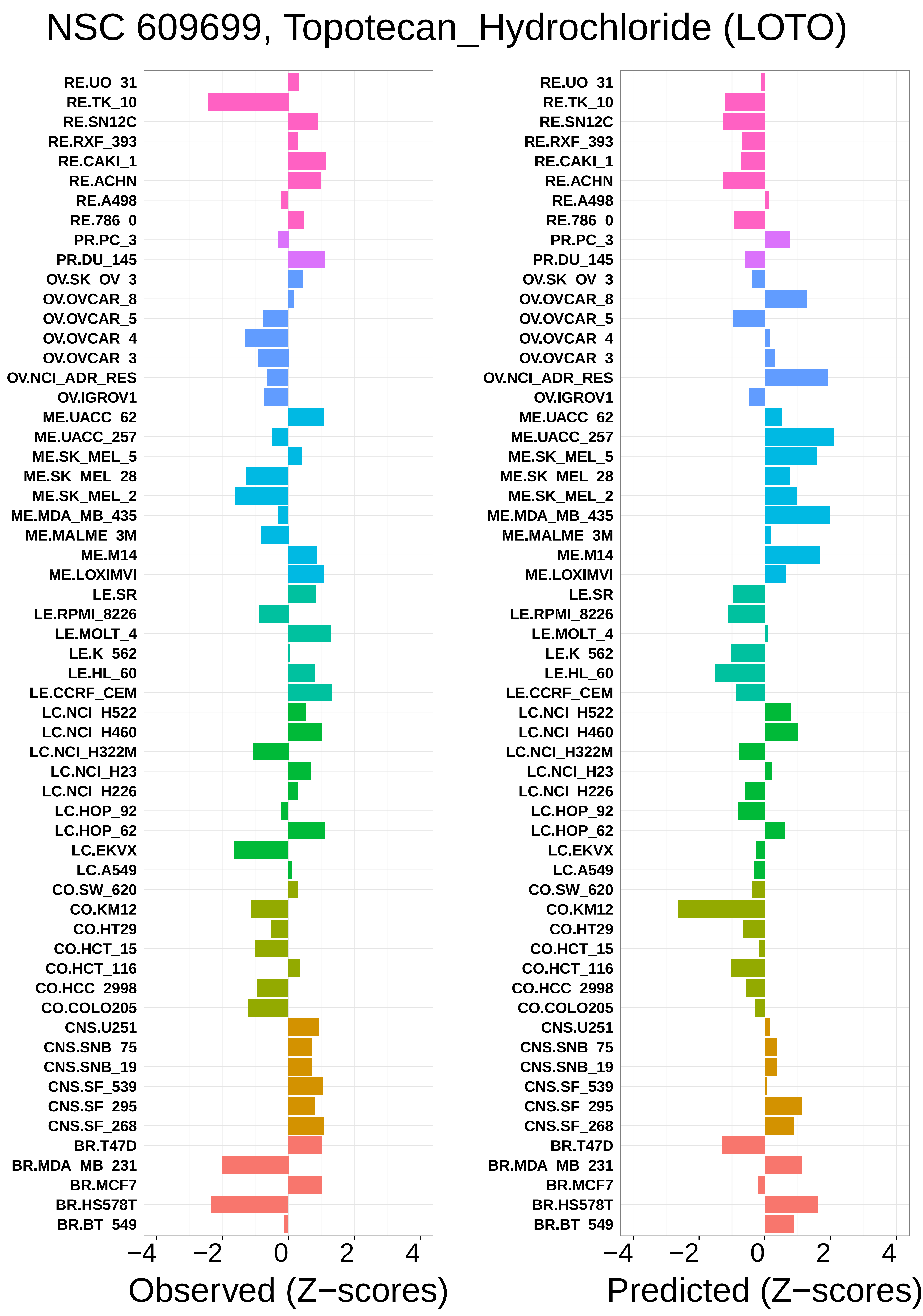
NSC 609699, Topotecan_Hydrochloride (LOTO)
RE.UO_31
RE.TK_10
RE.SN12C
RE.RXF_393
RE.CAKI_1
RE.ACHN
RE.A498
RE.786_0
PR.PC_3
PR.DU_145
OV.SK_OV_3
OV.OVCAR_8
OV.OVCAR_5
OV.OVCAR_4
OV.OVCAR_3
OV.NCI_ADR_RES
OV.IGROV1
ME.UACC_62
ME.UACC_257
ME.SK_MEL_5
ME.SK_MEL_28
ME.SK_MEL_2
ME.MDA_MB_435
ME.MALME_3M
ME.M14
ME.LOXIMVI
LE.SR
LE.RPMI_8226
LE.MOLT_4
LE.K_562
LE.HL_60
LE.CCRF_CEM
LC.NCI_H522
LC.NCI_H460
LC.NCI_H322M
LC.NCI_H23
LC.NCI_H226
LC.HOP_92
LC.HOP_62
LC.EKVX
LC.A549
CO.SW_620
CO.KM12
CO.HT29
CO.HCT_15
CO.HCT_116
CO.HCC_2998
CO.COLO205
CNS.U251
CNS.SNB_75
CNS.SNB_19
CNS.SF_539
CNS.SF_295
CNS.SF_268
BR.T47D
BR.MDA_MB_231
BR.MCF7
BR.HS578T
BR.BT_549
−4 −2 0 2 4
Observed (Z−scores)
−4 −2 0 2 4
Predicted (Z−scores)

NSC 613327, (LOTO)

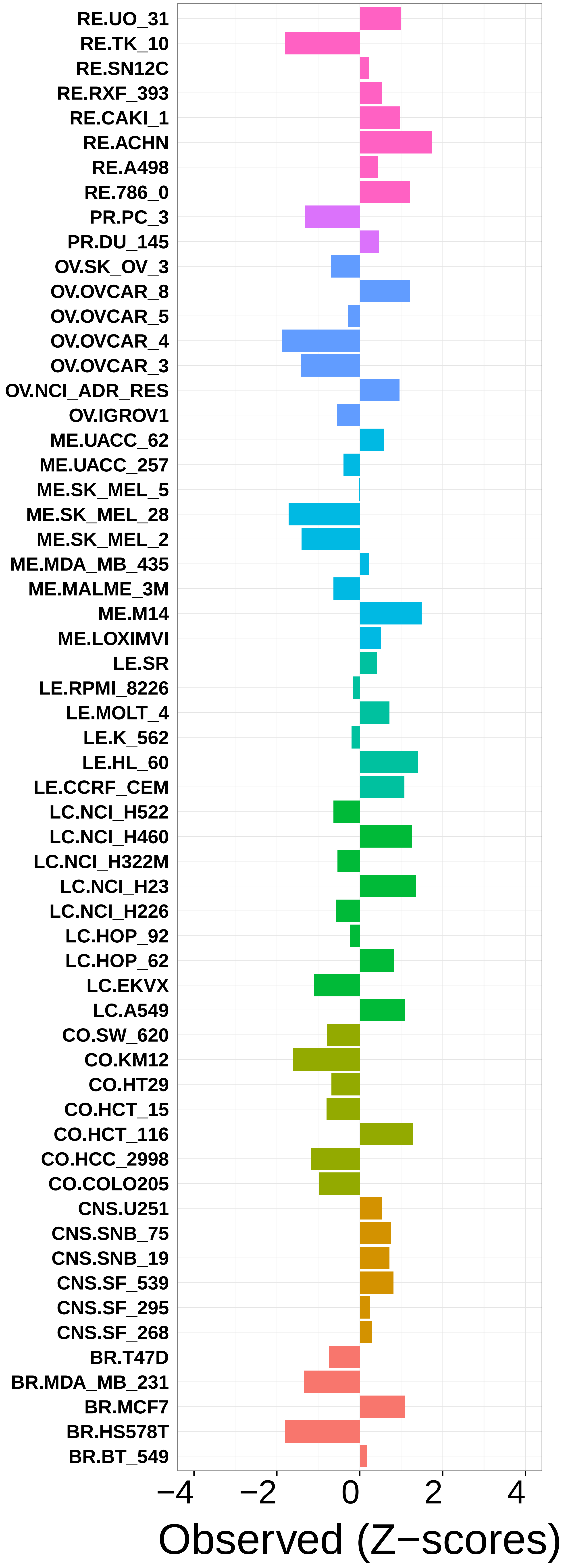

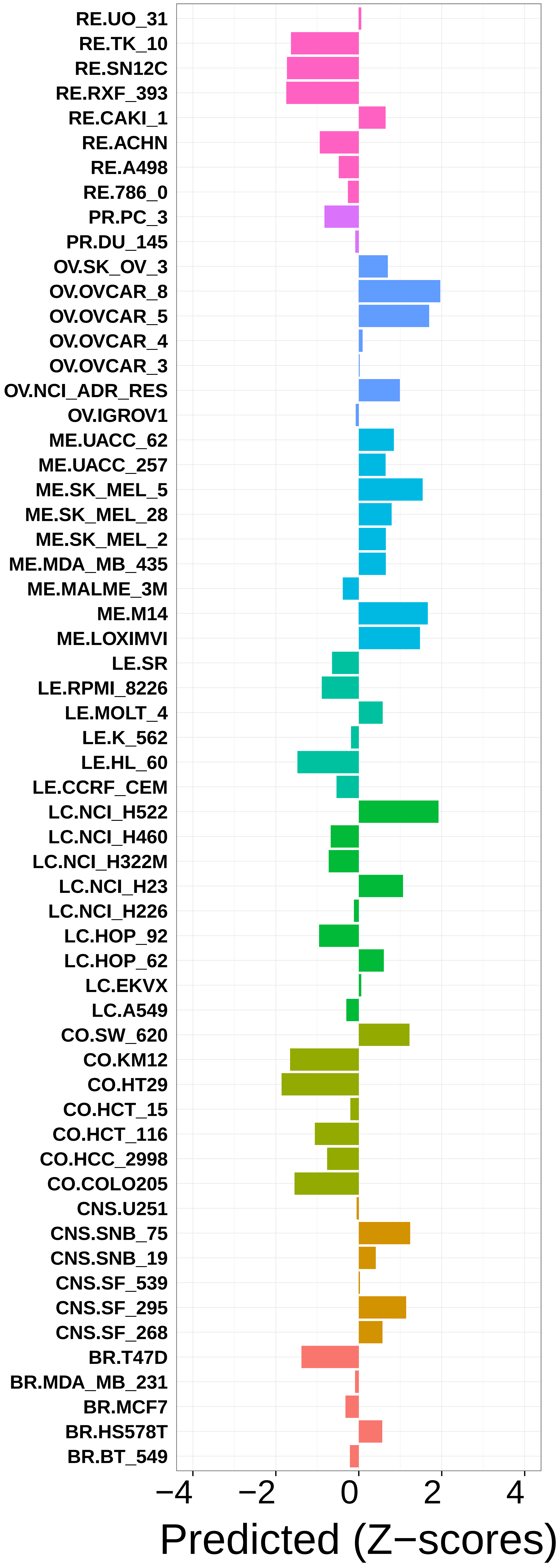

NSC 628503, Docetaxel (LOTO)

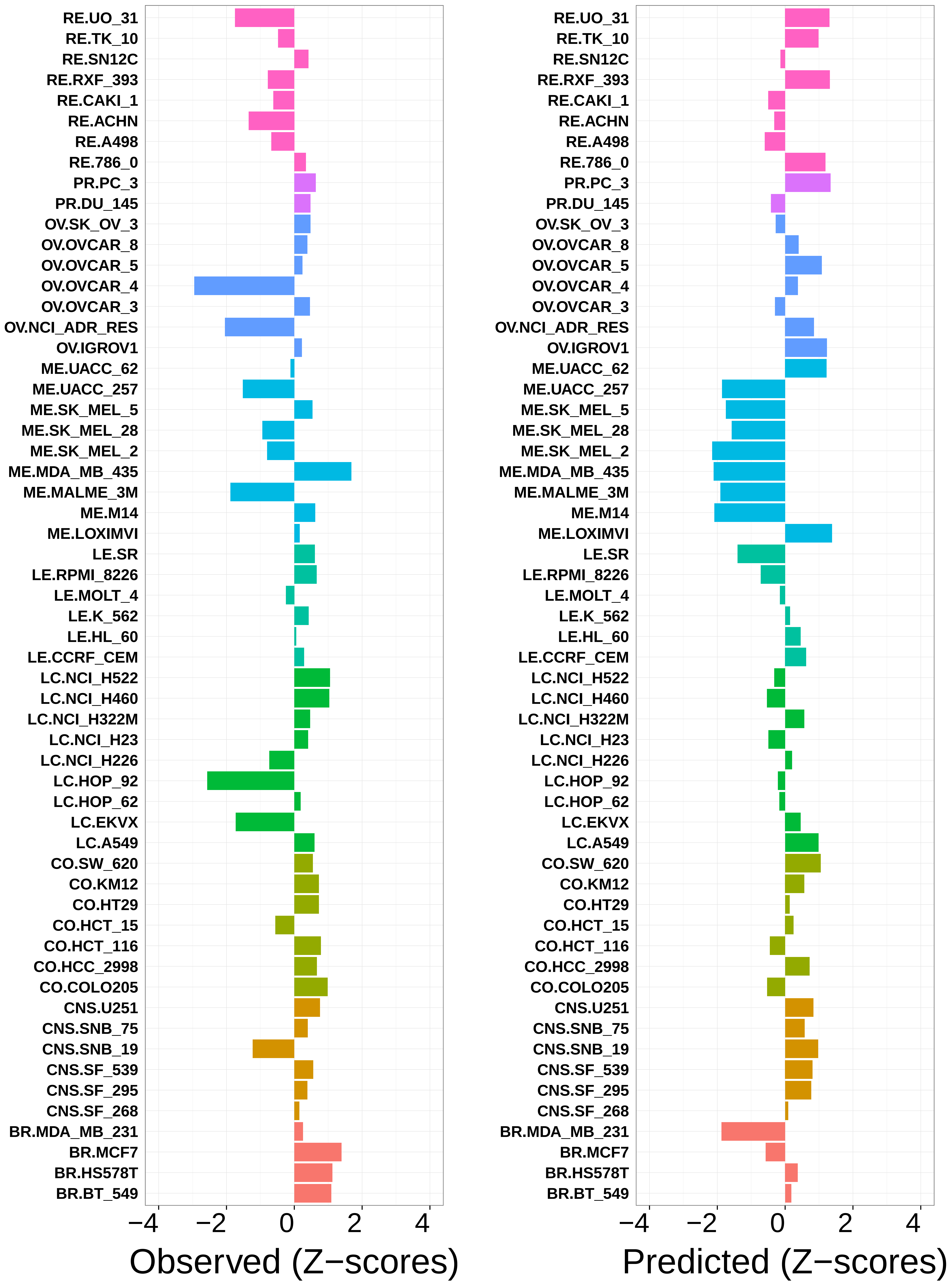

NSC 630176, Romidepsin (LOTO)

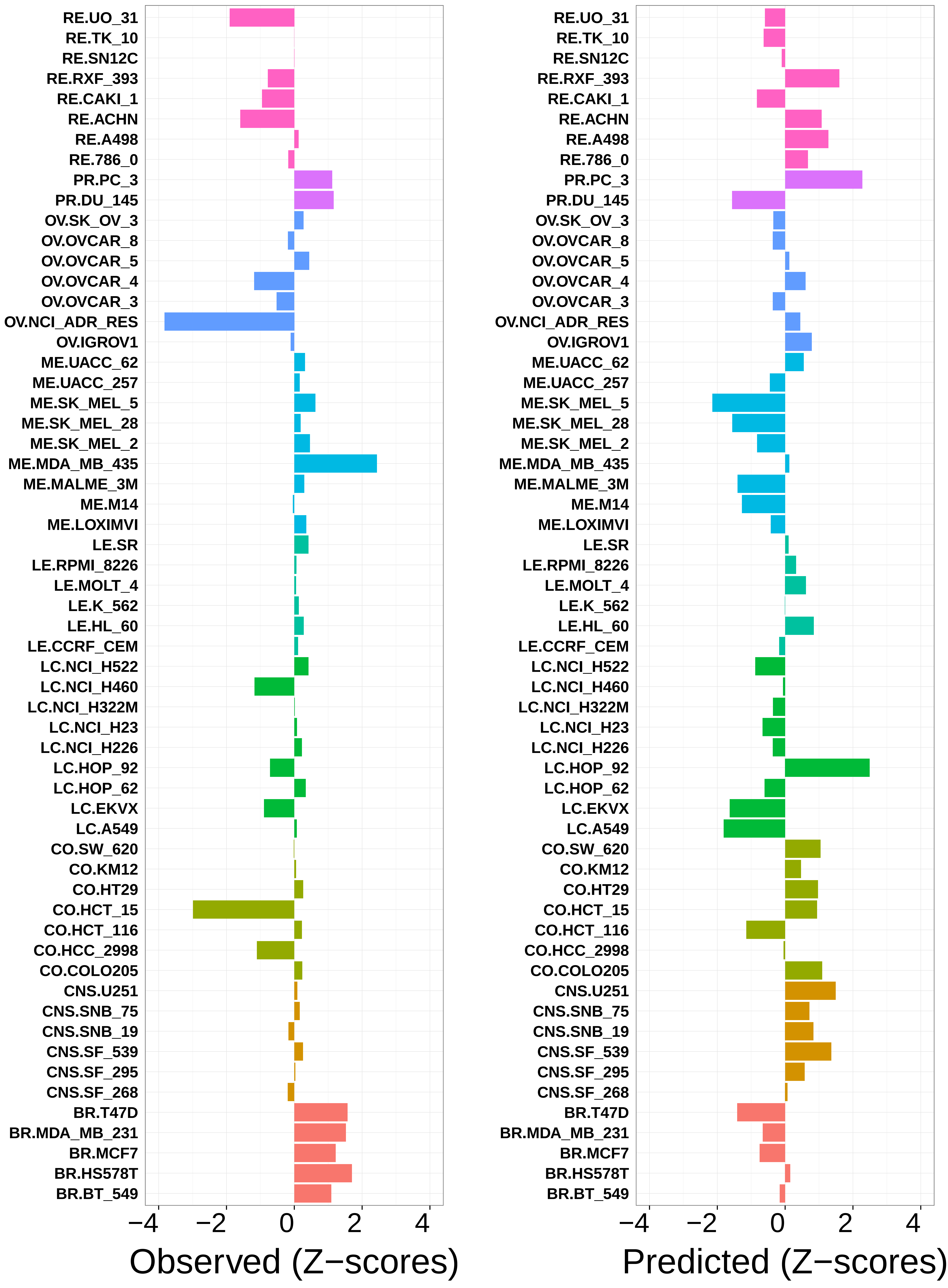

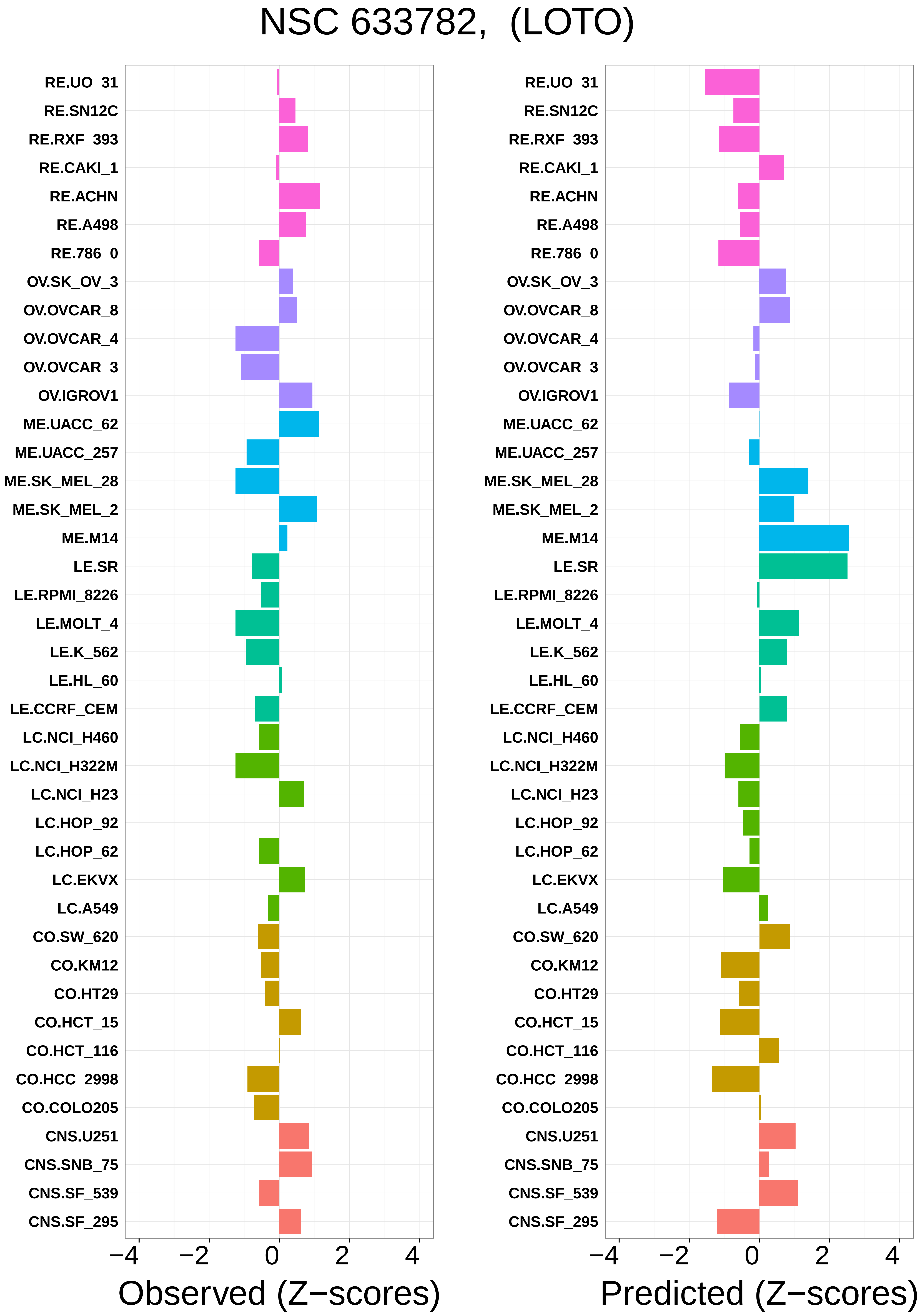
NSC 633782, (LOTO)
RE.UO_31
RE.SN12C
RE.RXF_393
RE.CAKI_1
RE.ACHN
RE.A498
RE.786_0
OV.SK_OV_3
OV.OVCAR_8
OV.OVCAR_4
OV.OVCAR_3
OV.IGROV1
ME.UACC_62
ME.UACC_257
ME.SK_MEL_28
ME.SK_MEL_2
ME.M14
LE.SR
LE.RPMI_8226
LE.MOLT_4
LE.K_562
LE.HL_60
LE.CCRF_CEM
LC.NCI_H460
LC.NCI_H322M
LC.NCI_H23
LC.HOP_92
LC.HOP_62
LC.EKVX
LC.A549
CO.SW_620
CO.KM12
CO.HT29
CO.HCT_15
CO.HCT_116
CO.HCC_2998
CO.COLO205
CNS.U251
CNS.SNB_75
CNS.SF_539
CNS.SF_295
−4 −2 0 2 4
Observed (Z−scores)
Predicted (Z−scores)

NSC 63878, Cytarabine_Hydrochloride (LOTO)

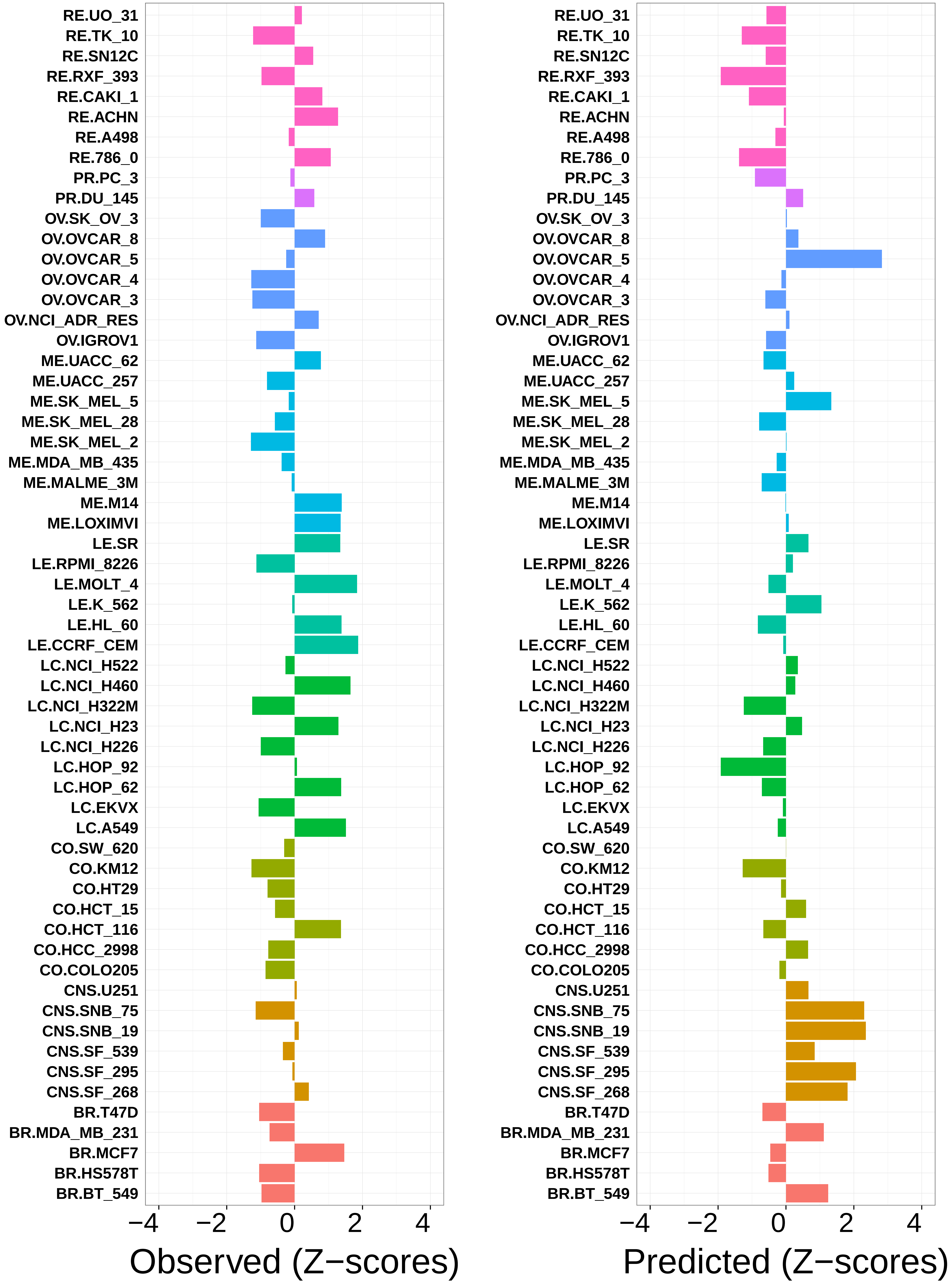

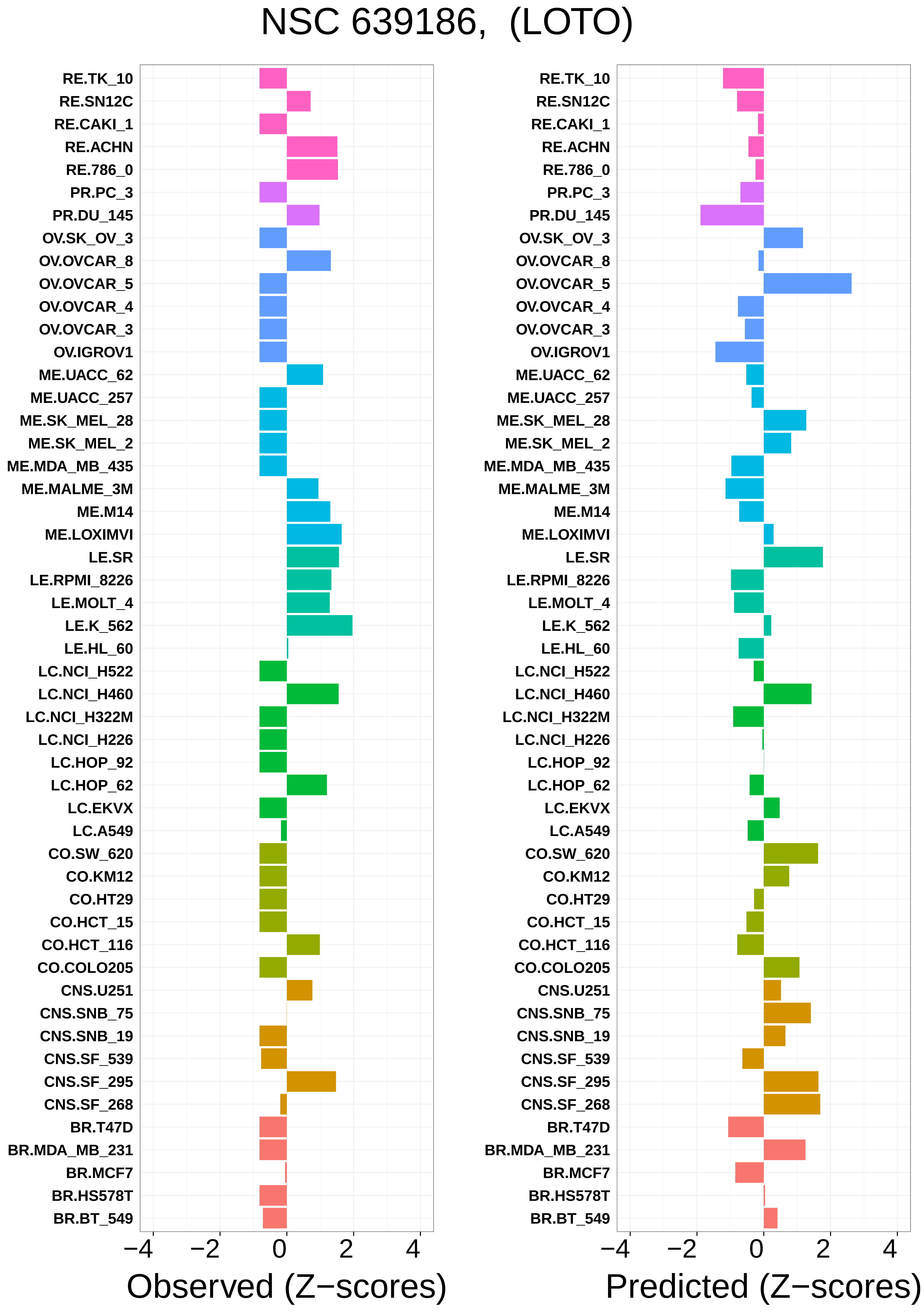

NSC 639186, (LOTO)
RE.TK_10
RE.SN12C
RE.CAKI_1
RE.ACHN
RE.786_0
PR.PC_3
PR.DU_145
OV.SK_OV_3
OV.OVCAR_8
OV.OVCAR_5
OV.OVCAR_4
OV.OVCAR_3
OV.IGROV1
ME.UACC_62
ME.UACC_257
ME.SK_MEL_28
ME.SK_MEL_2
ME.MDA_MB_435
ME.MALME_3M
ME.M14
ME.LOXIMVI
LE.SR
LE.RPMI_8226
LE.MOLT_4
LE.K_562
LE.HL_60
LC.NCI_H522
LC.NCI_H460
LC.NCI_H322M
LC.NCI_H226
LC.HOP_92
LC.HOP_62
LC.EKVX
LC.A549
CO.SW_620
CO.KM12
CO.HT29
CO.HCT_15
CO.HCT_116
CO.COLO205
CNS.U251
CNS.SNB_75
CNS.SNB_19
CNS.SF_539
CNS.SF_295
CNS.SF_268
BR.T47D
BR.MDA_MB_231
BR.MCF7
BR.HS578T
BR.BT_549
−4
−2
0
2
4
Observed (Z−scores)
Predicted (Z−scores)

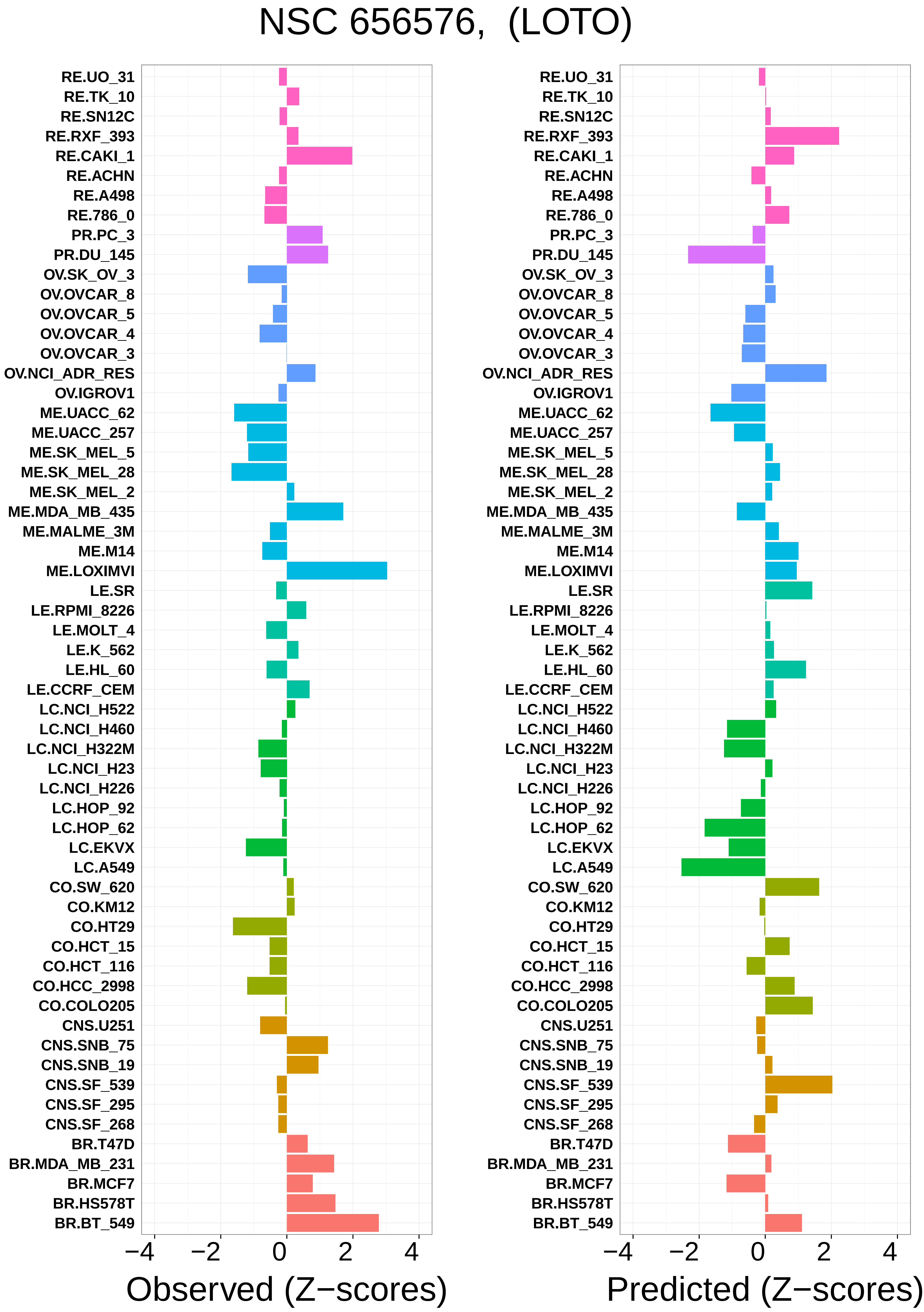

NSC 656576, (LOTO)
RE.UO_31
RE.TK_10
RE.SN12C
RE.RXF_393
RE.CAKI_1
RE.ACHN
RE.A498
RE.786_0
PR.PC_3
PR.DU_145
OV.SK_OV_3
OV.OVCAR_8
OV.OVCAR_5
OV.OVCAR_4
OV.OVCAR_3
OV.NCI_ADR_RES
OV.IGROV1
ME.UACC_62
ME.UACC_257
ME.SK_MEL_5
ME.SK_MEL_28
ME.SK_MEL_2
ME.MDA_MB_435
ME.MALME_3M
ME.M14
ME.LOXIMVI
LE.SR
LE.RPMI_8226
LE.MOLT_4
LE.K_562
LE.HL_60
LE.CCRF_CEM
LC.NCI_H522
LC.NCI_H460
LC.NCI_H322M
LC.NCI_H23
LC.NCI_H226
LC.HOP_92
LC.HOP_62
LC.EKVX
LC.A549
CO.SW_620
CO.KM12
CO.HT29
CO.HCT_15
CO.HCT_116
CO.HCC_2998
CO.COLO205
CNS.U251
CNS.SNB_75
CNS.SNB_19
CNS.SF_539
CNS.SF_295
CNS.SF_268
BR.T47D
BR.MDA_MB_231
BR.MCF7
BR.HS578T
BR.BT_549
−4
−2
0
2
4
Observed (Z−scores)
Predicted (Z−scores)

NSC 673596, (LOTO)

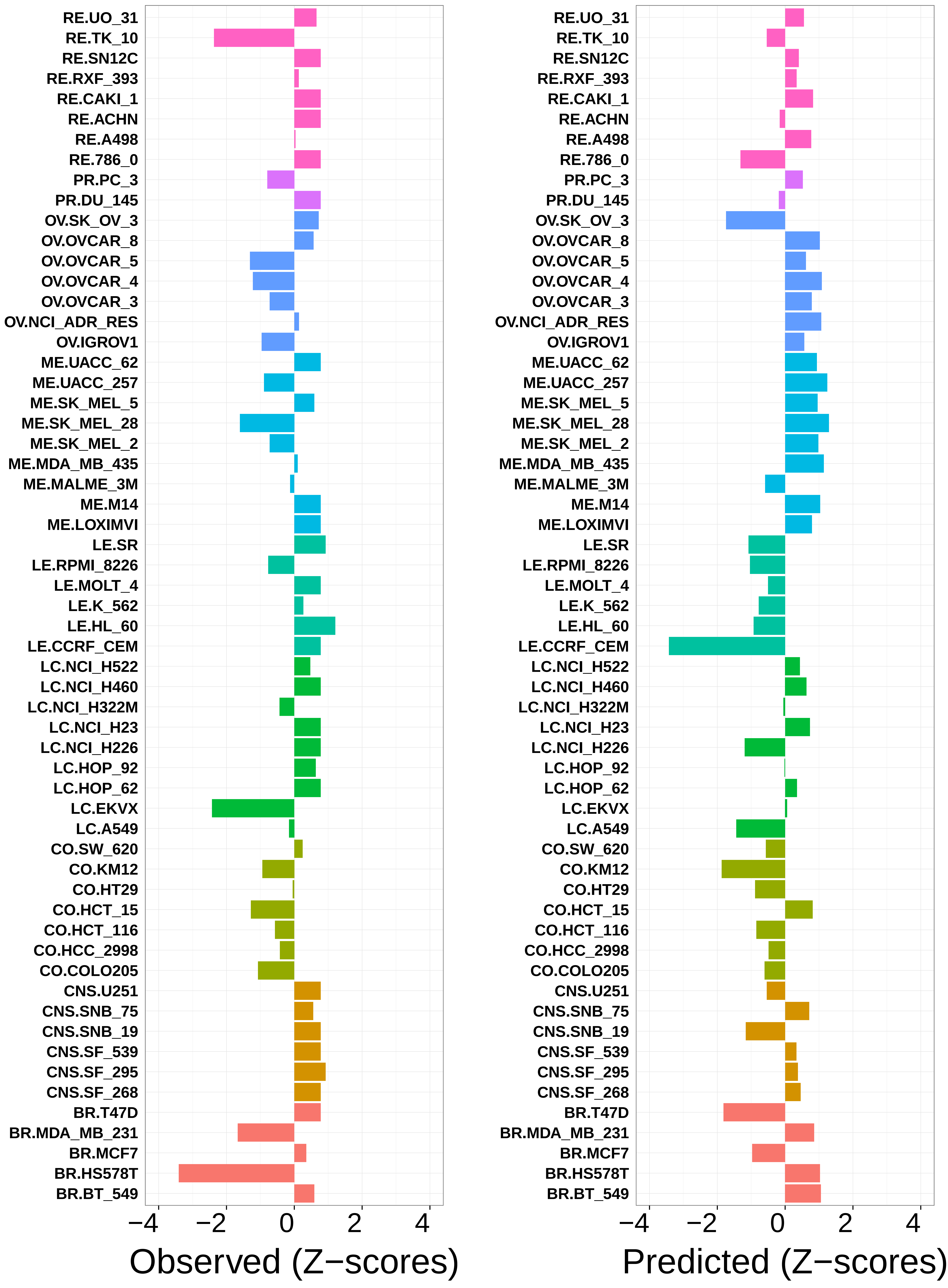

NSC 686288, (LOTO)

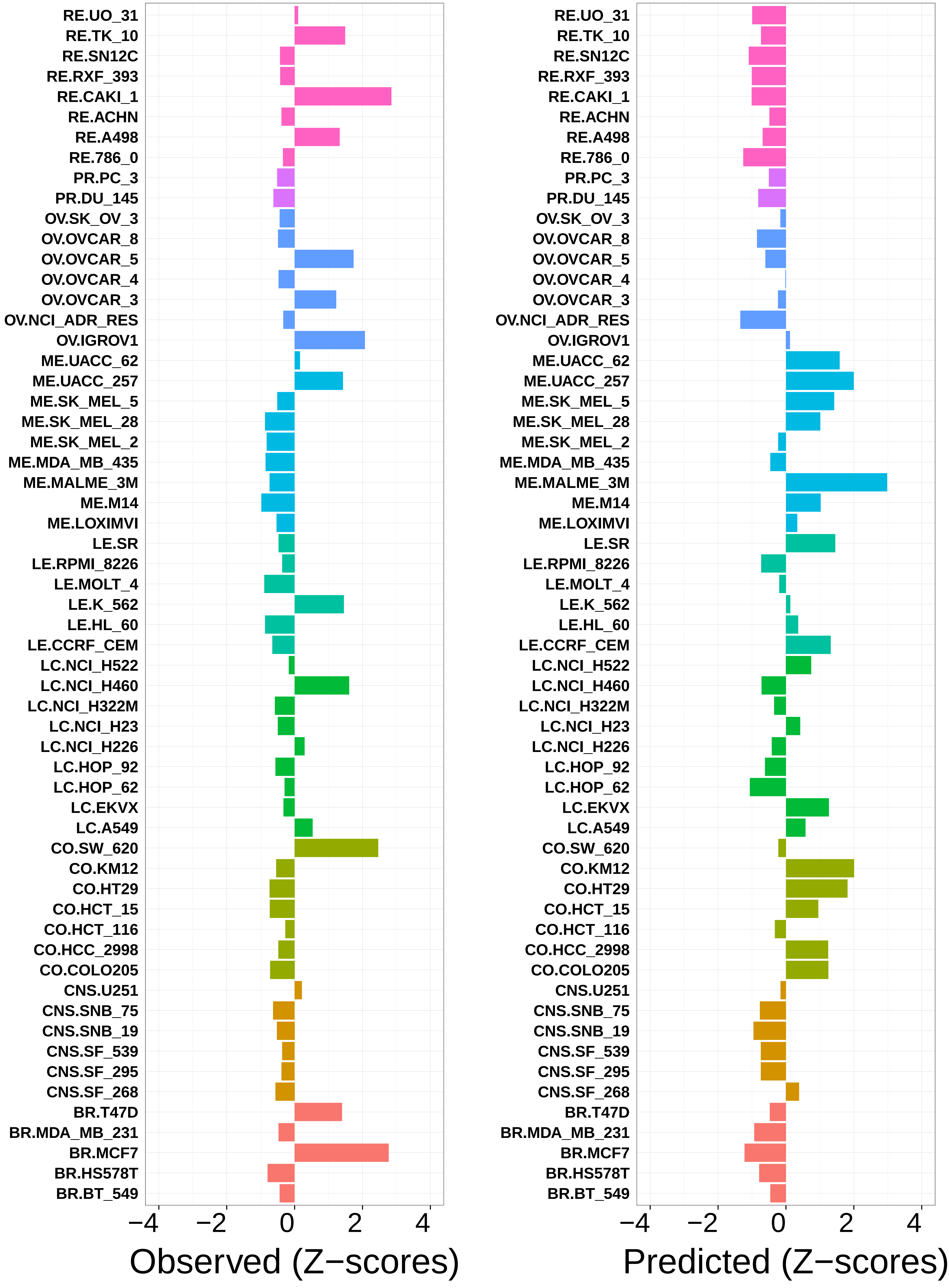

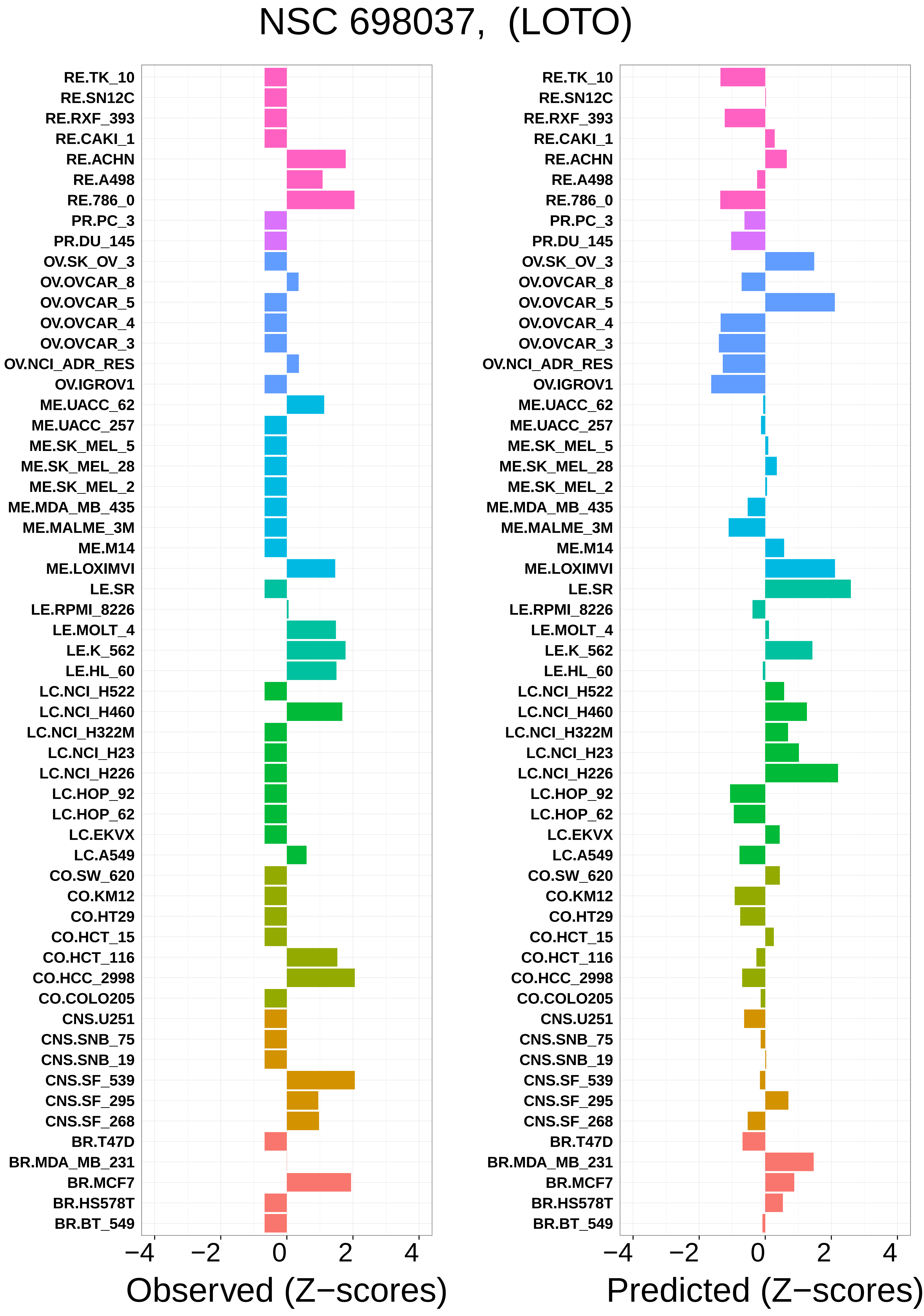
NSC 698037, (LOTO)
RE.TK_10
RE.SN12C
RE.RXF_393
RE.CAKI_1
RE.ACHN
RE.A498
RE.786_0
PR.PC_3
PR.DU_145
OV.SK_OV_3
OV.OVCAR_8
OV.OVCAR_5
OV.OVCAR_4
OV.OVCAR_3
OV.NCI_ADR_RES
OV.IGROV1
ME.UACC_62
ME.UACC_257
ME.SK_MEL_5
ME.SK_MEL_28
ME.SK_MEL_2
ME.MDA_MB_435
ME.MALME_3M
ME.M14
ME.LOXIMVI
LE.SR
LE.RPMI_8226
LE.MOLT_4
LE.K_562
LE.HL_60
LC.NCI_H522
LC.NCI_H460
LC.NCI_H322M
LC.NCI_H23
LC.NCI_H226
LC.HOP_92
LC.HOP_62
LC.EKVX
LC.A549
CO.SW_620
CO.KM12
CO.HT29
CO.HCT_15
CO.HCT_116
CO.HCC_2998
CO.COLO205
CNS.U251
CNS.SNB_75
CNS.SNB_19
CNS.SF_539
CNS.SF_295
CNS.SF_268
BR.T47D
BR.MDA_MB_231
BR.MCF7
BR.HS578T
BR.BT_549
−4 −2 0 2 4
Observed (Z−scores)
−4 −2 0 2 4
Predicted (Z−scores)

NSC 707545, Alvespimycin (LOTO)

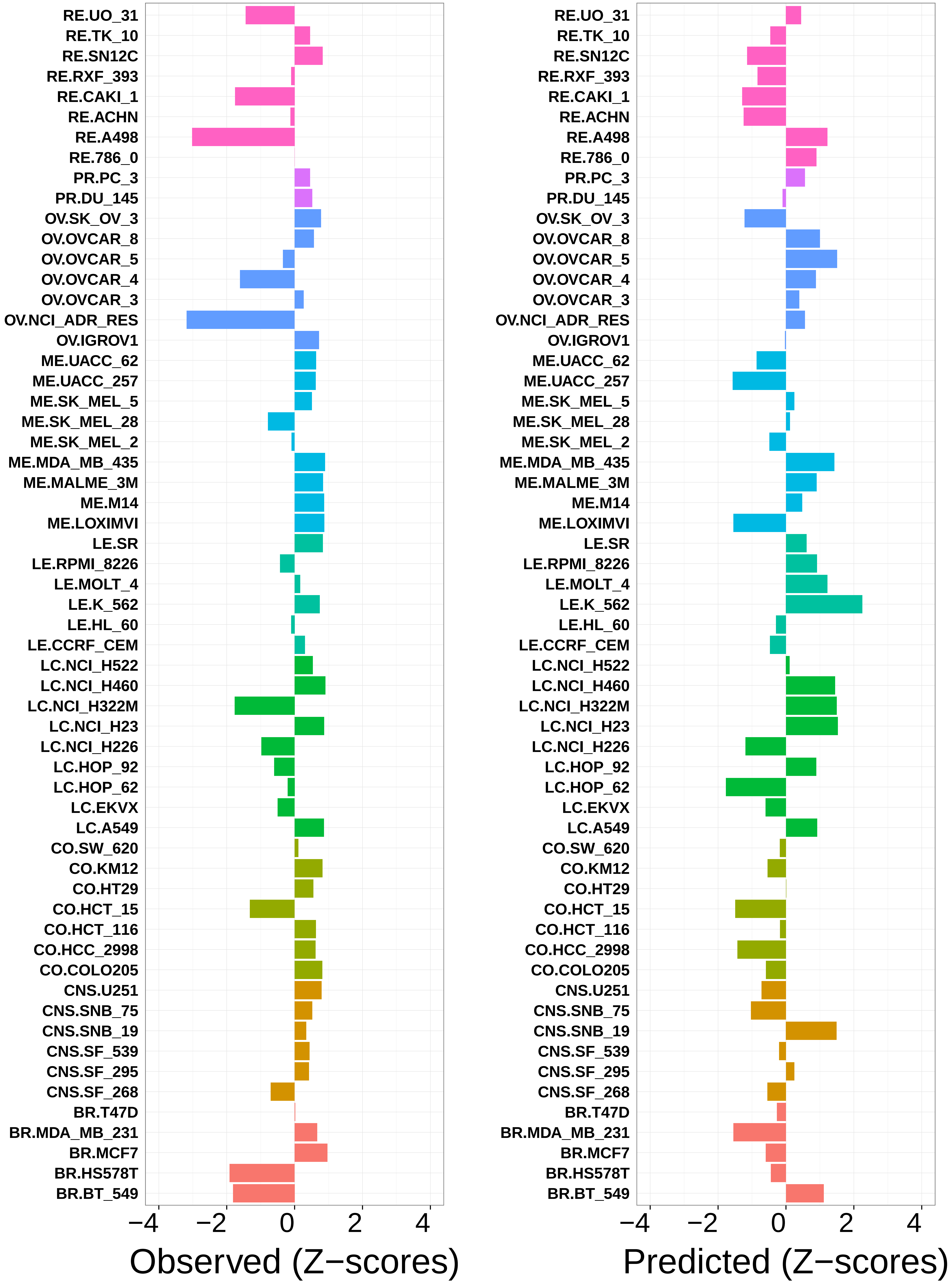

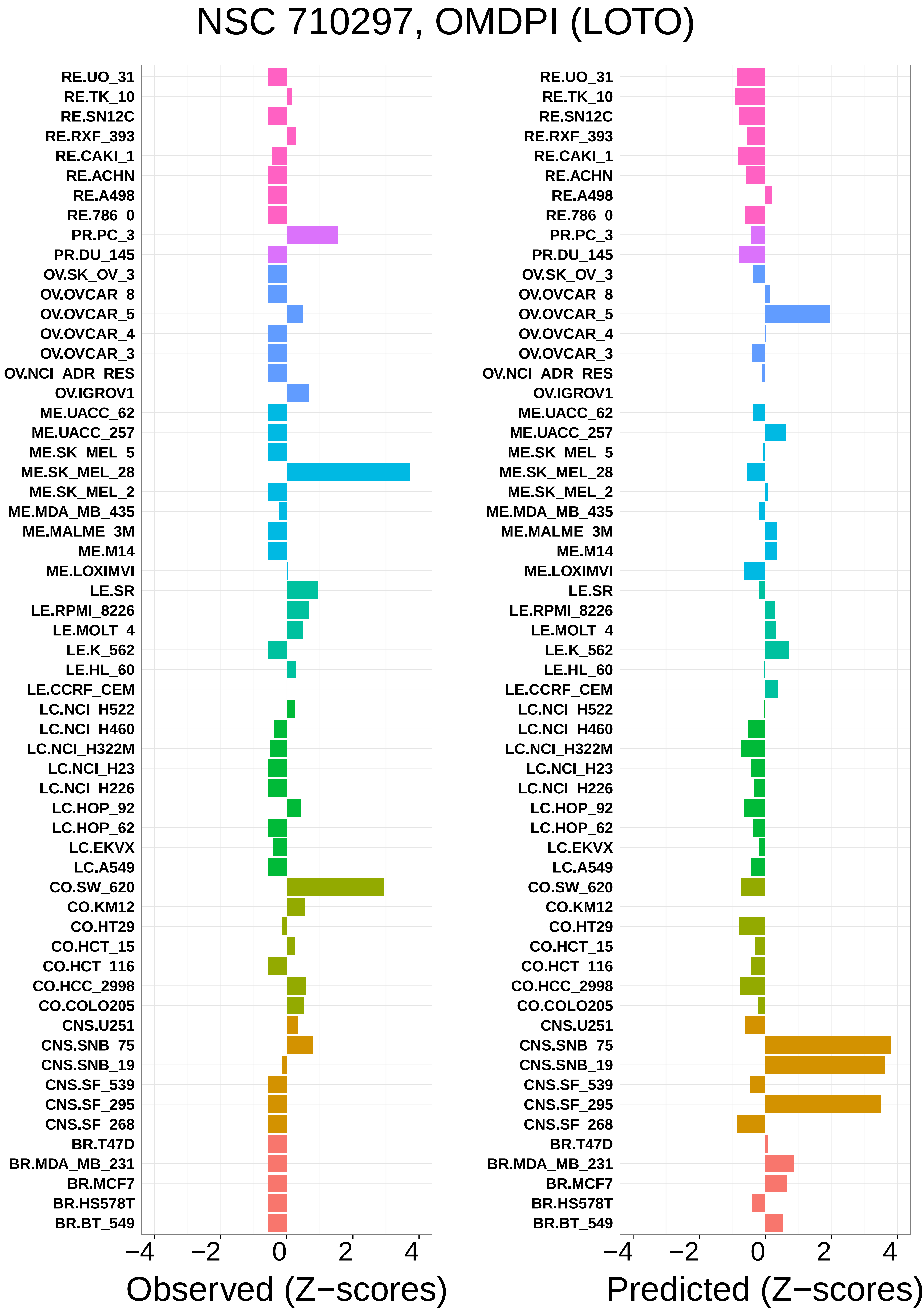
NSC 710297, OMDPI (LOTO)
RE.UO_31
RE.TK_10
RE.SN12C
RE.RXF_393
RE.CAKI_1
RE.ACHN
RE.A498
RE.786_0
PR.PC_3
PR.DU_145
OV.SK_OV_3
OV.OVCAR_8
OV.OVCAR_5
OV.OVCAR_4
OV.OVCAR_3
OV.NCI_ADR_RES
OV.IGROV1
ME.UACC_62
ME.UACC_257
ME.SK_MEL_5
ME.SK_MEL_28
ME.SK_MEL_2
ME.MDA_MB_435
ME.MALME_3M
ME.M14
ME.LOXIMVI
LE.SR
LE.RPMI_8226
LE.MOLT_4
LE.K_562
LE.HL_60
LE.CCRF_CEM
LC.NCI_H522
LC.NCI_H460
LC.NCI_H322M
LC.NCI_H23
LC.NCI_H226
LC.HOP_92
LC.HOP_62
LC.EKVX
LC.A549
CO.SW_620
CO.KM12
CO.HT29
CO.HCT_15
CO.HCT_116
CO.HCC_2998
CO.COLO205
CNS.U251
CNS.SNB_75
CNS.SNB_19
CNS.SF_539
CNS.SF_295
CNS.SF_268
BR.T47D
BR.MDA_MB_231
BR.MCF7
BR.HS578T
BR.BT_549
−4
−2
0
2
4
Observed (Z−scores)
Predicted (Z−scores)

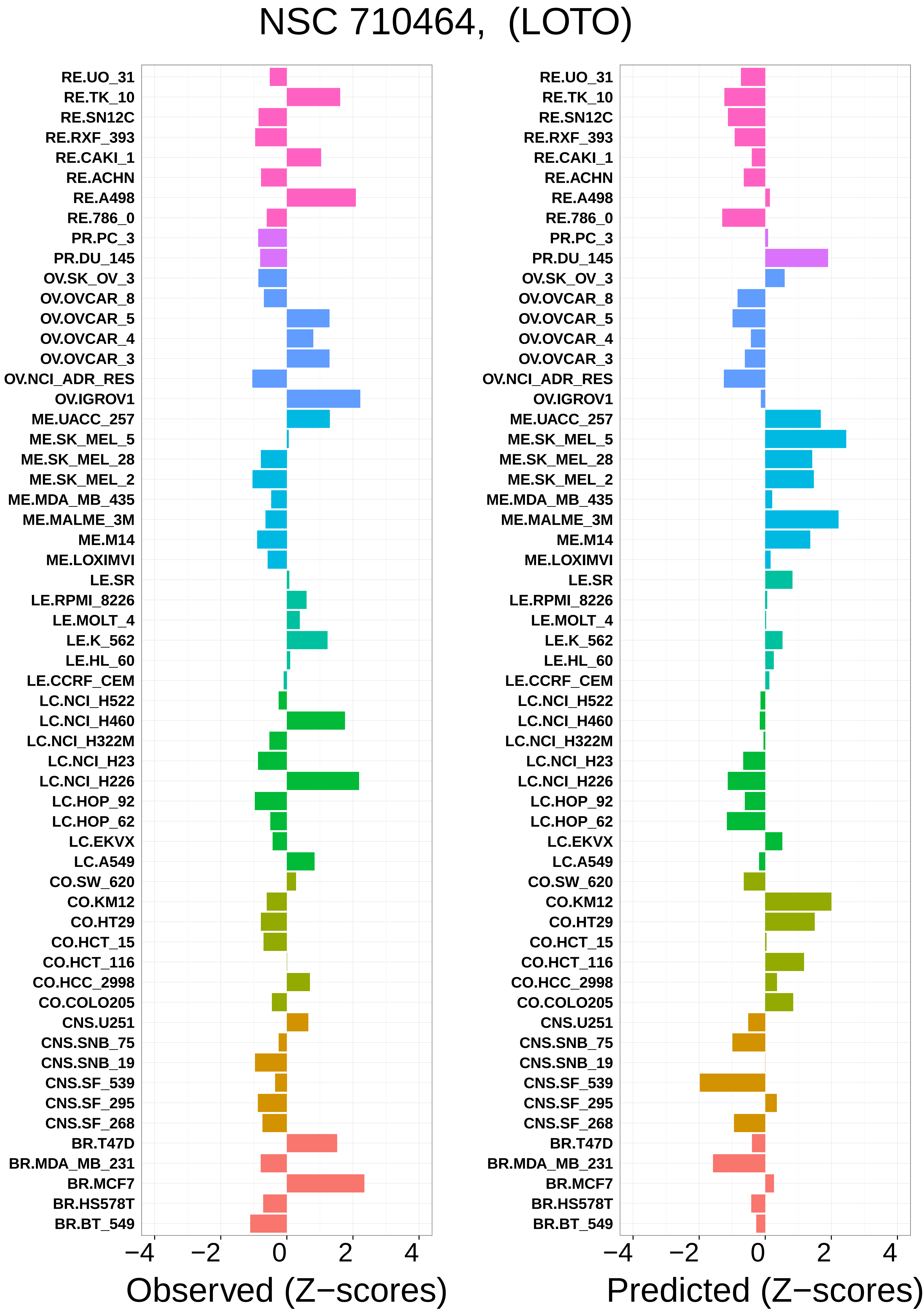

NSC 710464, (LOTO)
RE.UO_31
RE.TK_10
RE.SN12C
RE.RXF_393
RE.CAKI_1
RE.ACHN
RE.A498
RE.786_0
PR.PC_3
PR.DU_145
OV.SK_OV_3
OV.OVCAR_8
OV.OVCAR_5
OV.OVCAR_4
OV.OVCAR_3
OV.NCI_ADR_RES
OV.IGROV1
ME.UACC_257
ME.SK_MEL_5
ME.SK_MEL_28
ME.SK_MEL_2
ME.MDA_MB_435
ME.MALME_3M
ME.M14
ME.LOXIMVI
LE.SR
LE.RPMI_8226
LE.MOLT_4
LE.K_562
LE.HL_60
LE.CCRF_CEM
LC.NCI_H522
LC.NCI_H460
LC.NCI_H322M
LC.NCI_H23
LC.NCI_H226
LC.HOP_92
LC.HOP_62
LC.EKVX
LC.A549
CO.SW_620
CO.KM12
CO.HT29
CO.HCT_15
CO.HCT_116
CO.HCC_2998
CO.COLO205
CNS.U251
CNS.SNB_75
CNS.SNB_19
CNS.SF_539
CNS.SF_295
CNS.SF_268
BR.T47D
BR.MDA_MB_231
BR.MCF7
BR.HS578T
BR.BT_549
−4
−2
0
2
4
Observed (Z−scores)
Predicted (Z−scores)

NSC 715055, Gefitinib (LOTO)

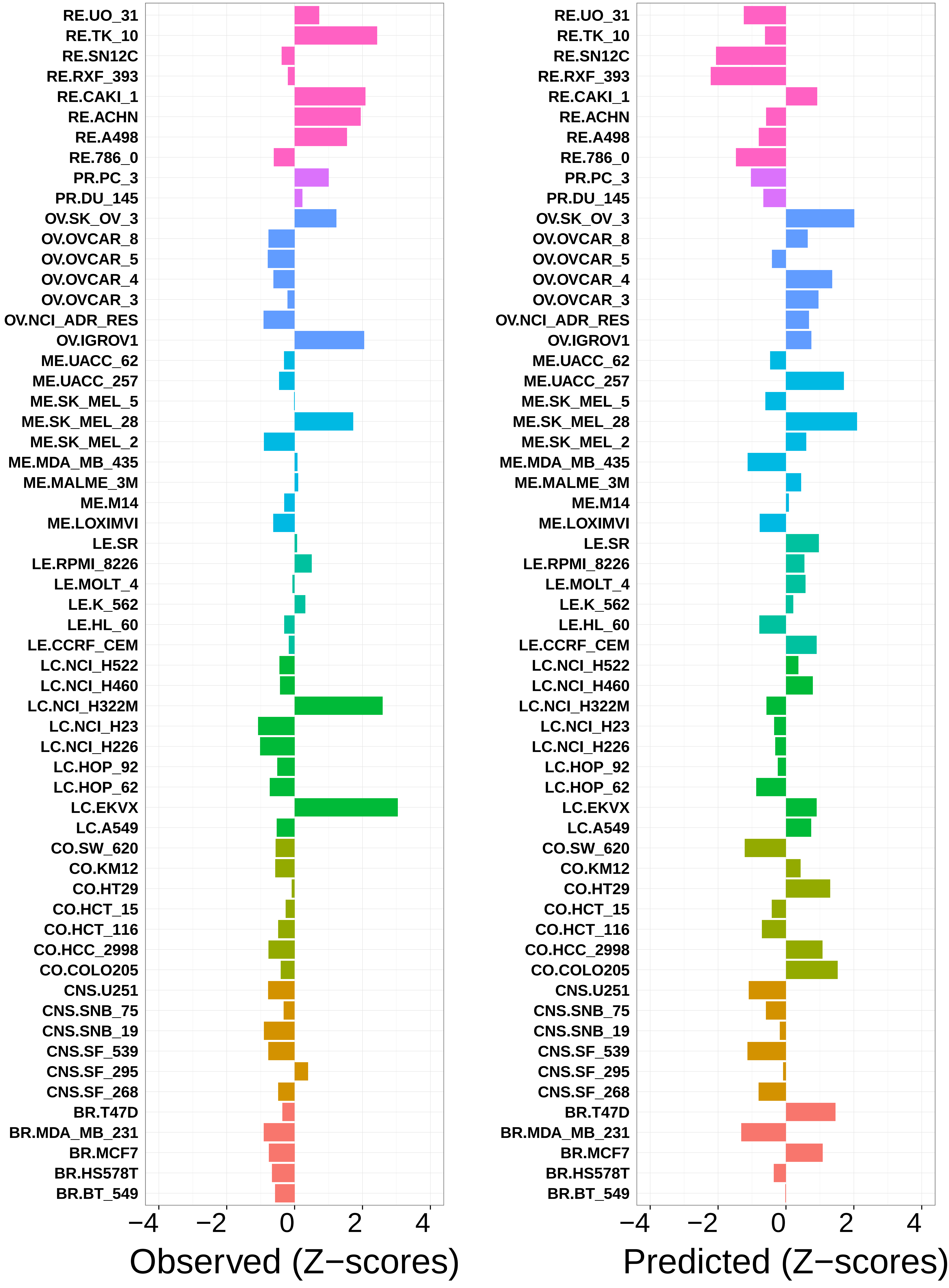

NSC 718781, (LOTO)

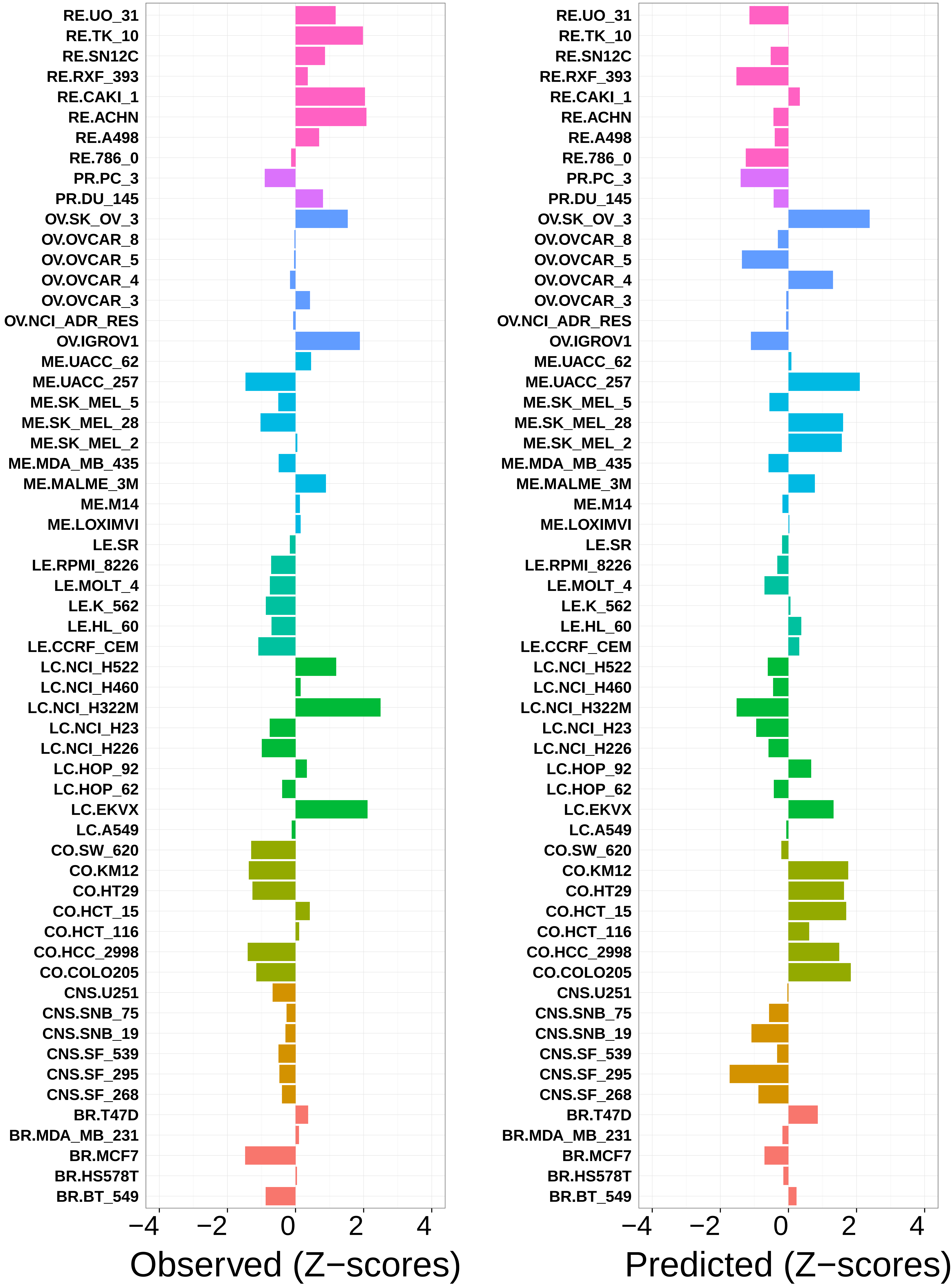

NSC 719239, (LOTO)

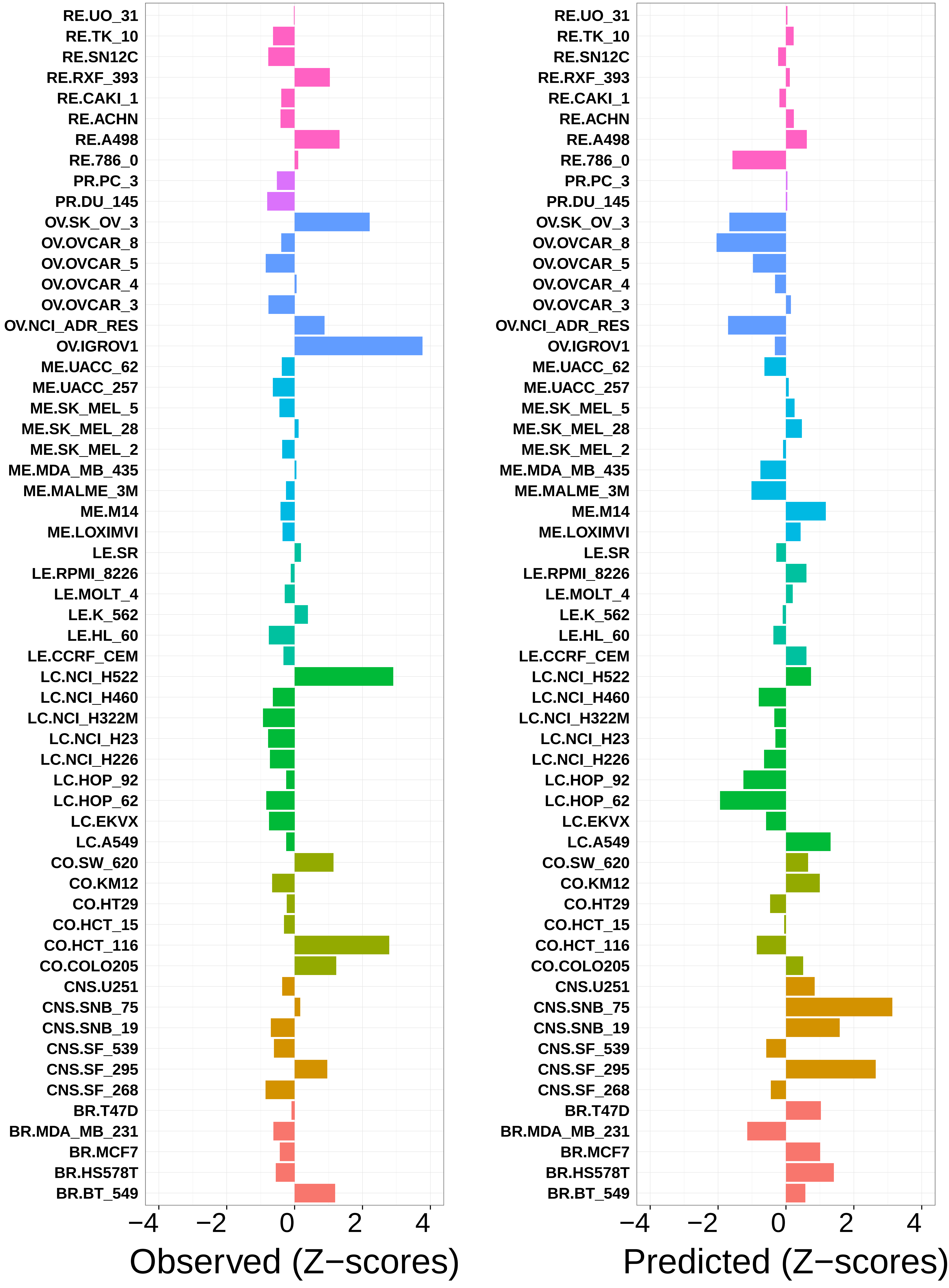

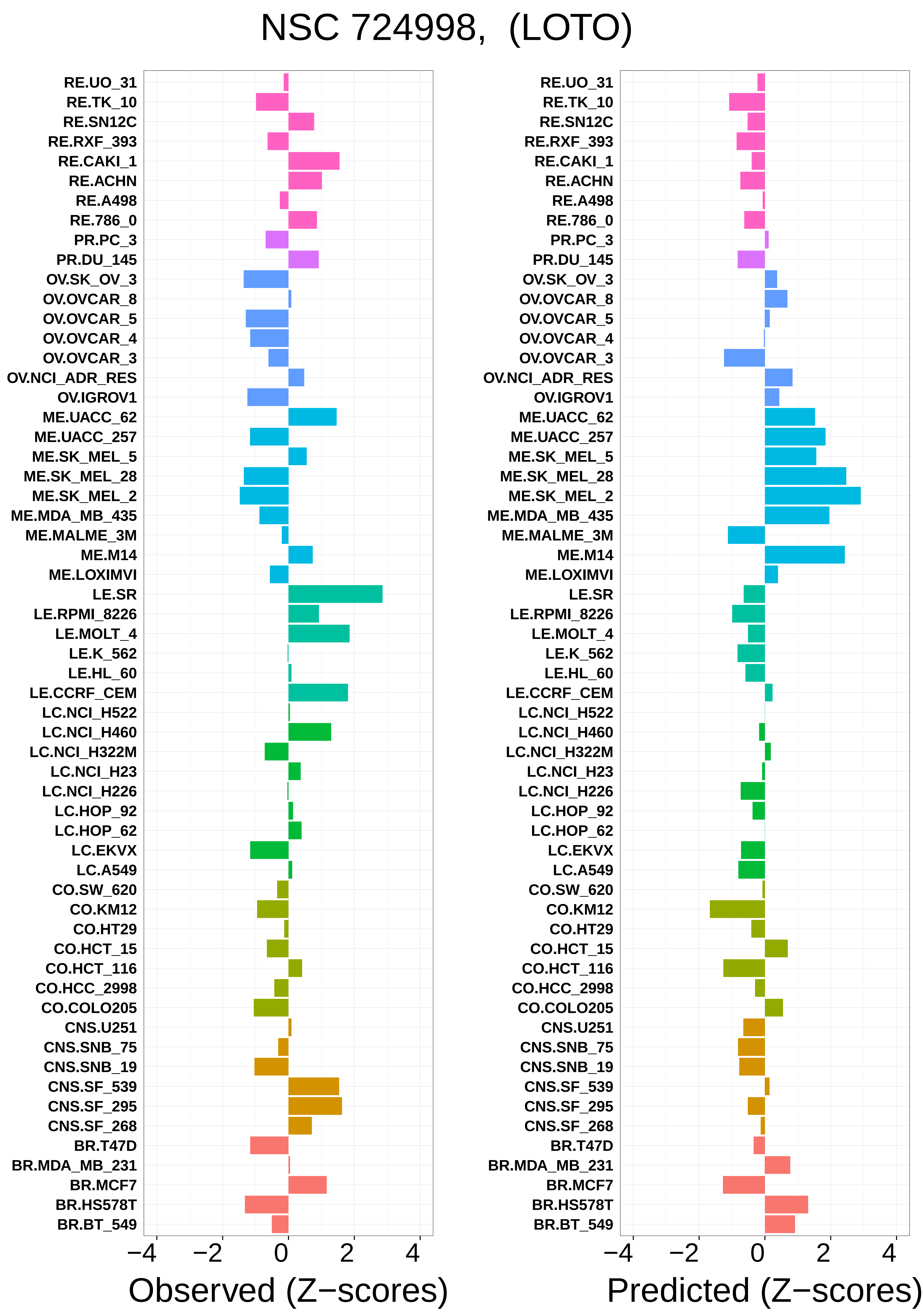
NSC 724998, (LOTO)
RE.UO_31
RE.TK_10
RE.SN12C
RE.RXF_393
RE.CAKI_1
RE.ACHN
RE.A498
RE.786_0
PR.PC_3
PR.DU_145
OV.SK_OV_3
OV.OVCAR_8
OV.OVCAR_5
OV.OVCAR_4
OV.OVCAR_3
OV.NCI_ADR_RES
OV.IGROV1
ME.UACC_62
ME.UACC_257
ME.SK_MEL_5
ME.SK_MEL_28
ME.SK_MEL_2
ME.MDA_MB_435
ME.MALME_3M
ME.M14
ME.LOXIMVI
LE.SR
LE.RPMI_8226
LE.MOLT_4
LE.K_562
LE.HL_60
LE.CCRF_CEM
LC.NCI_H522
LC.NCI_H460
LC.NCI_H322M
LC.NCI_H23
LC.NCI_H226
LC.HOP_92
LC.HOP_62
LC.EKVX
LC.A549
CO.SW_620
CO.KM12
CO.HT29
CO.HCT_15
CO.HCT_116
CO.HCC_2998
CO.COLO205
CNS.U251
CNS.SNB_75
CNS.SNB_19
CNS.SF_539
CNS.SF_295
CNS.SF_268
BR.T47D
BR.MDA_MB_231
BR.MCF7
BR.HS578T
BR.BT_549
−4
−2
0
2
4
Observed (Z−scores)
Predicted (Z−scores)

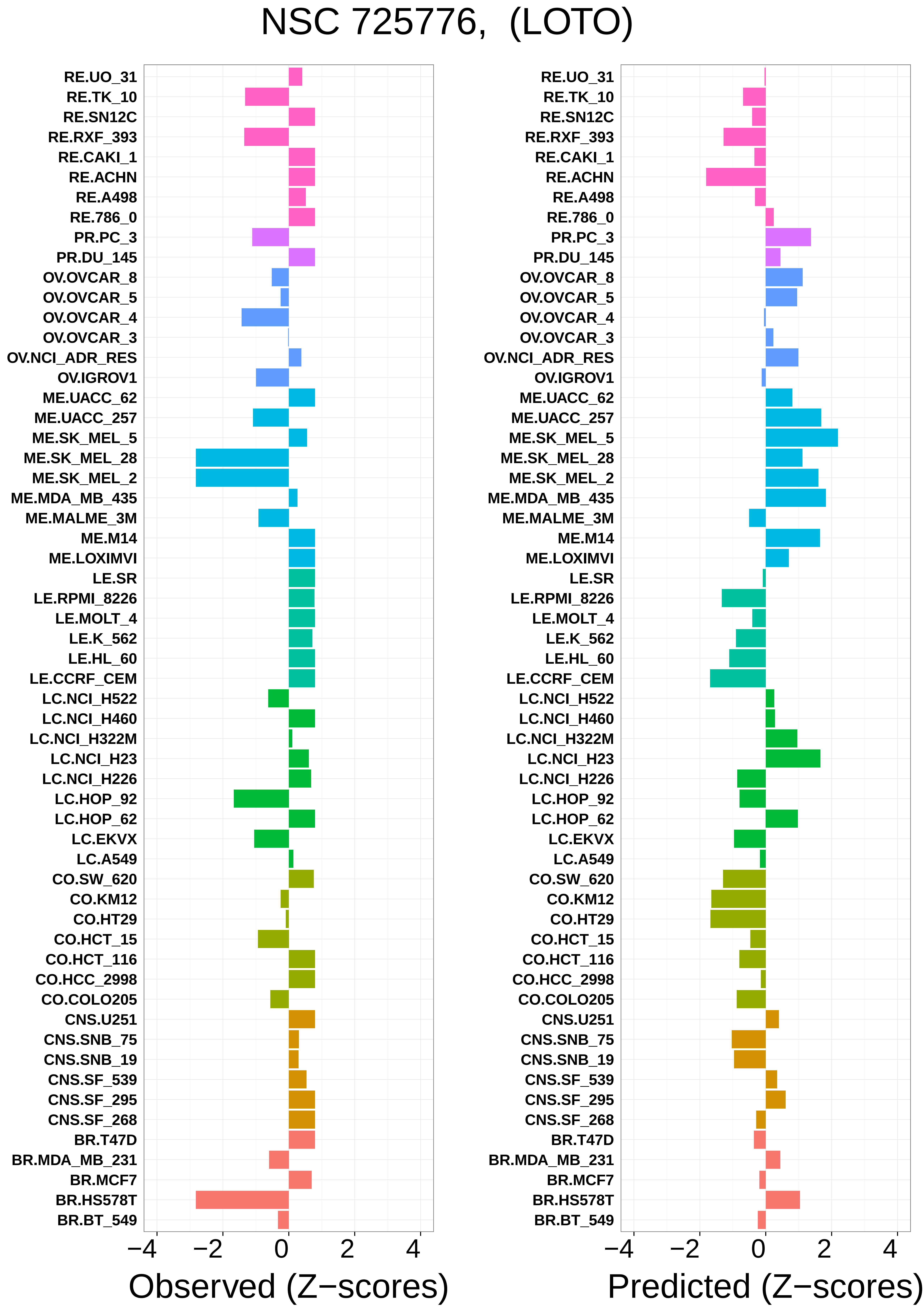
NSC 725776, (LOTO)
RE.UO_31
RE.TK_10
RE.SN12C
RE.RXF_393
RE.CAKI_1
RE.ACHN
RE.A498
RE.786_0
PR.PC_3
PR.DU_145
OV.OVCAR_8
OV.OVCAR_5
OV.OVCAR_4
OV.OVCAR_3
OV.NCI_ADR_RES
OV.IGROV1
ME.UACC_62
ME.UACC_257
ME.SK_MEL_5
ME.SK_MEL_28
ME.SK_MEL_2
ME.MDA_MB_435
ME.MALME_3M
ME.M14
ME.LOXIMVI
LE.SR
LE.RPMI_8226
LE.MOLT_4
LE.K_562
LE.HL_60
LE.CCRF_CEM
LC.NCI_H522
LC.NCI_H460
LC.NCI_H322M
LC.NCI_H23
LC.NCI_H226
LC.HOP_92
LC.HOP_62
LC.EKVX
LC.A549
CO.SW_620
CO.KM12
CO.HT29
CO.HCT_15
CO.HCT_116
CO.HCC_2998
CO.COLO205
CNS.U251
CNS.SNB_75
CNS.SNB_19
CNS.SF_539
CNS.SF_295
CNS.SF_268
BR.T47D
BR.MDA_MB_231
BR.MCF7
BR.HS578T
BR.BT_549
−4 −2 0 2 4
Observed (Z−scores)
Predicted (Z−scores)

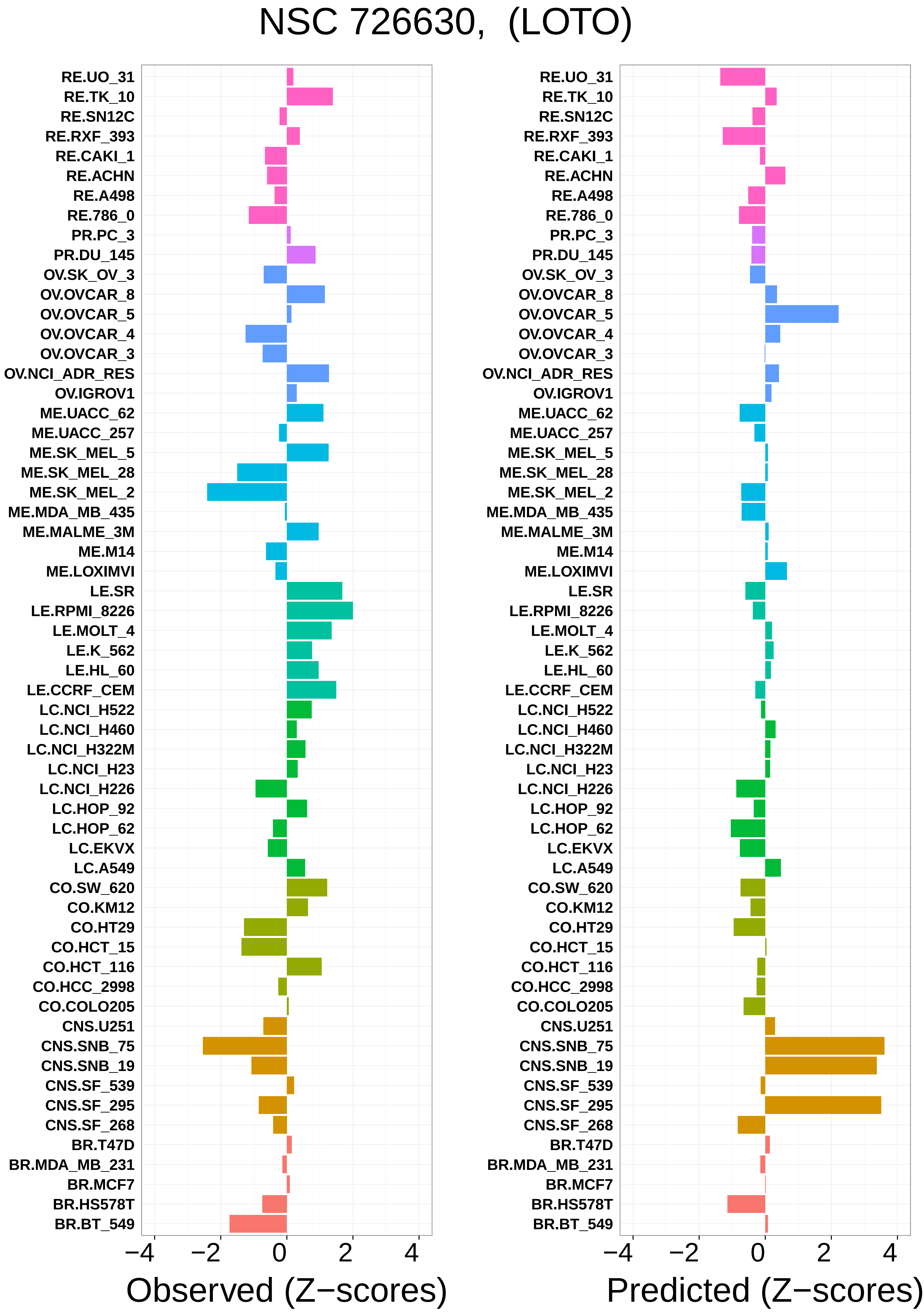

NSC 726630, (LOTO)
RE.UO_31
RE.TK_10
RE.SN12C
RE.RXF_393
RE.CAKI_1
RE.ACHN
RE.A498
RE.786_0
PR.PC_3
PR.DU_145
OV.SK_OV_3
OV.OVCAR_8
OV.OVCAR_5
OV.OVCAR_4
OV.OVCAR_3
OV.NCI_ADR_RES
OV.IGROV1
ME.UACC_62
ME.UACC_257
ME.SK_MEL_5
ME.SK_MEL_28
ME.SK_MEL_2
ME.MDA_MB_435
ME.MALME_3M
ME.M14
ME.LOXIMVI
LE.SR
LE.RPMI_8226
LE.MOLT_4
LE.K_562
LE.HL_60
LE.CCRF_CEM
LC.NCI_H522
LC.NCI_H460
LC.NCI_H322M
LC.NCI_H23
LC.NCI_H226
LC.HOP_92
LC.HOP_62
LC.EKVX
LC.A549
CO.SW_620
CO.KM12
CO.HT29
CO.HCT_15
CO.HCT_116
CO.HCC_2998
CO.COLO205
CNS.U251
CNS.SNB_75
CNS.SNB_19
CNS.SF_539
CNS.SF_295
CNS.SF_268
BR.T47D
BR.MDA_MB_231
BR.MCF7
BR.HS578T
BR.BT_549
−4 −2 0 2 4
Observed (Z−scores)
Predicted (Z−scores)

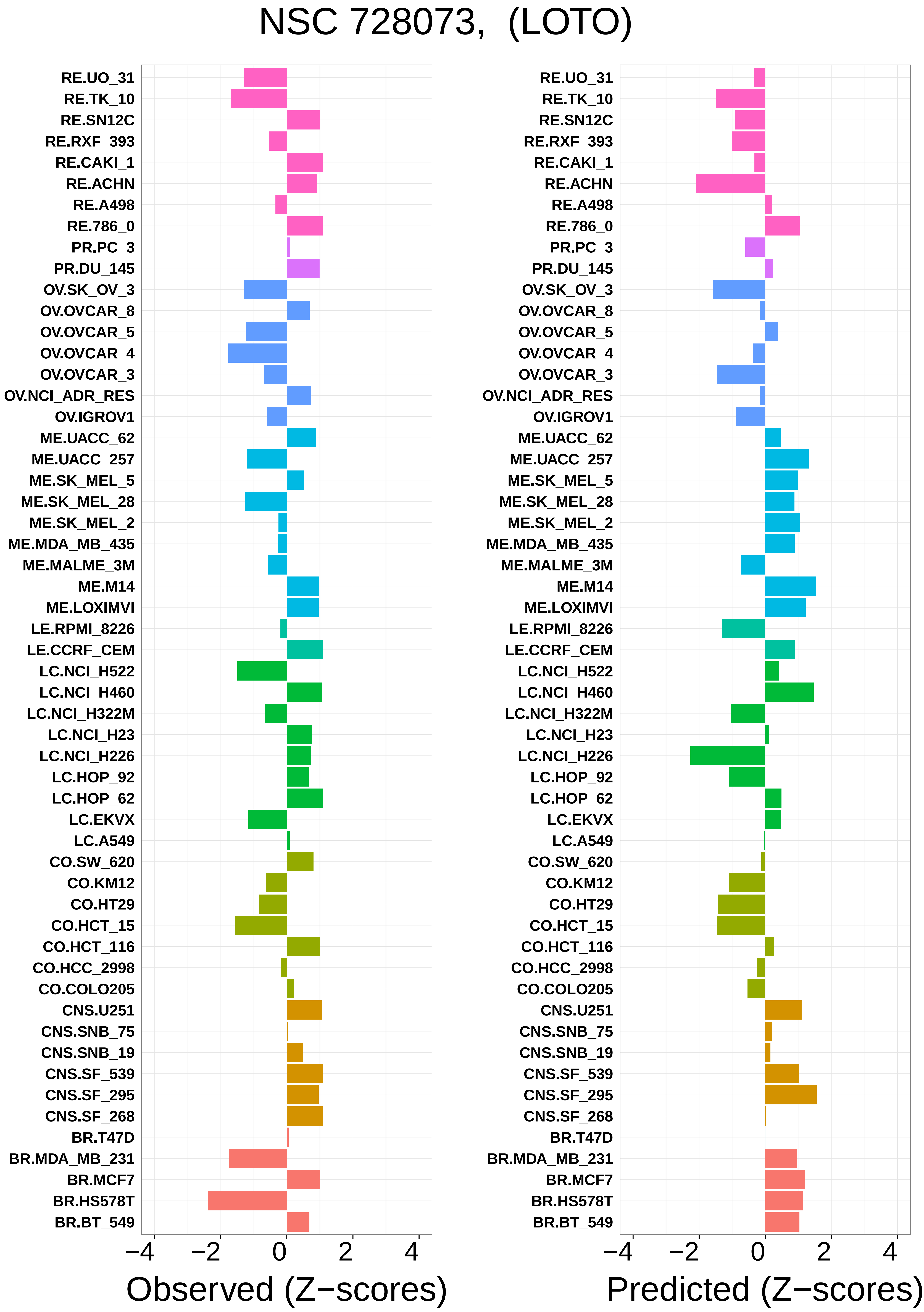

NSC 728073, (LOTO)
RE.UO_31
RE.TK_10
RE.SN12C
RE.RXF_393
RE.CAKI_1
RE.ACHN
RE.A498
RE.786_0
PR.PC_3
PR.DU_145
OV.SK_OV_3
OV.OVCAR_8
OV.OVCAR_5
OV.OVCAR_4
OV.OVCAR_3
OV.NCI_ADR_RES
OV.IGROV1
ME.UACC_62
ME.UACC_257
ME.SK_MEL_5
ME.SK_MEL_28
ME.SK_MEL_2
ME.MDA_MB_435
ME.MALME_3M
ME.M14
ME.LOXIMVI
LE.RPMI_8226
LE.CCRF_CEM
LC.NCI_H522
LC.NCI_H460
LC.NCI_H322M
LC.NCI_H23
LC.NCI_H226
LC.HOP_92
LC.HOP_62
LC.EKVX
LC.A549
CO.SW_620
CO.KM12
CO.HT29
CO.HCT_15
CO.HCT_116
CO.HCC_2998
CO.COLO205
CNS.U251
CNS.SNB_75
CNS.SNB_19
CNS.SF_539
CNS.SF_295
CNS.SF_268
BR.T47D
BR.MDA_MB_231
BR.MCF7
BR.HS578T
BR.BT_549
−4 −2 0 2 4
Observed (Z−scores)
Predicted (Z−scores)

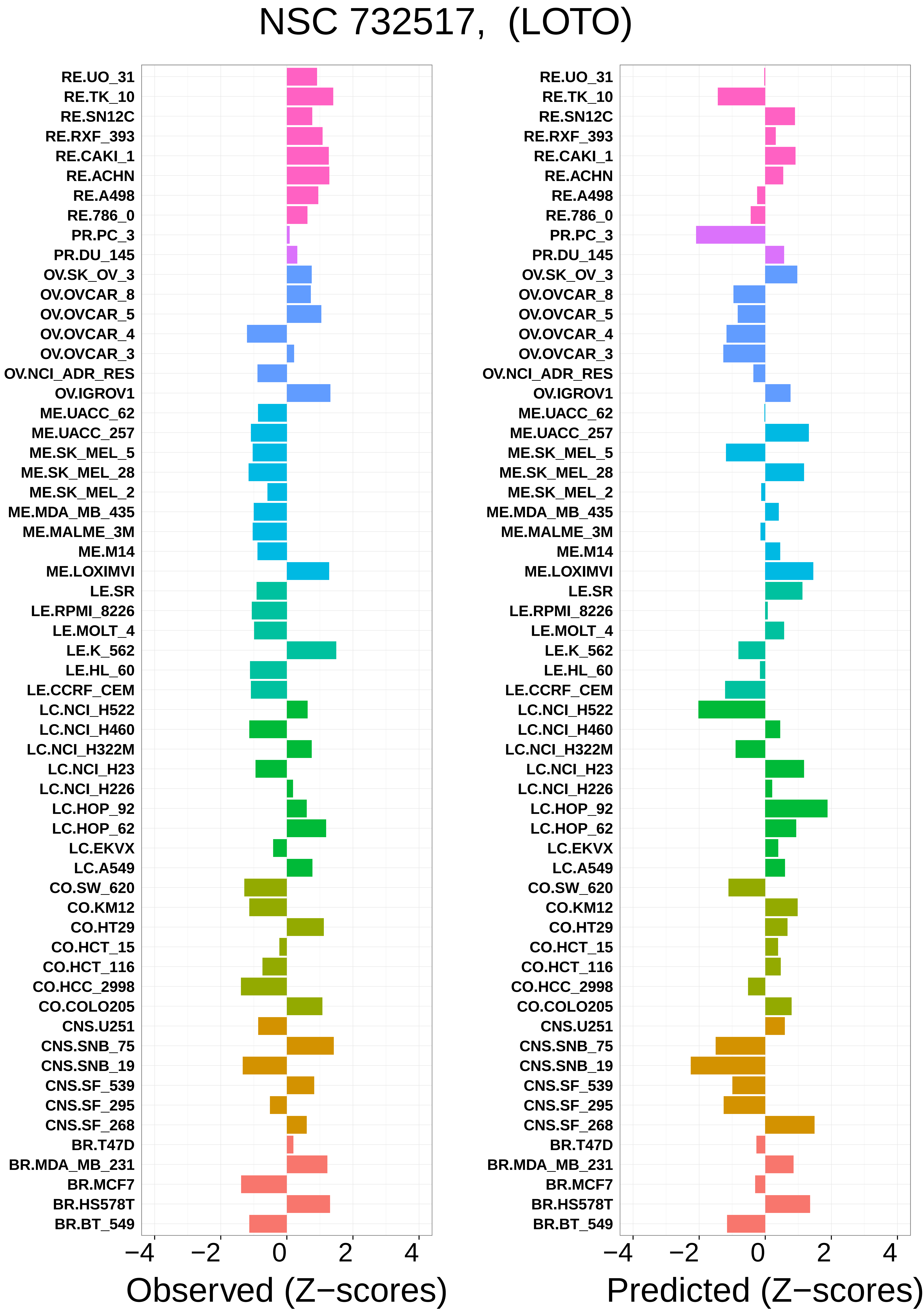
NSC 732517, (LOTO)
RE.UO_31
RE.TK_10
RE.SN12C
RE.RXF_393
RE.CAKI_1
RE.ACHN
RE.A498
RE.786_0
PR.PC_3
PR.DU_145
OV.SK_OV_3
OV.OVCAR_8
OV.OVCAR_5
OV.OVCAR_4
OV.OVCAR_3
OV.NCI_ADR_RES
OV.IGROV1
ME.UACC_62
ME.UACC_257
ME.SK_MEL_5
ME.SK_MEL_28
ME.SK_MEL_2
ME.MDA_MB_435
ME.MALME_3M
ME.M14
ME.LOXIMVI
LE.SR
LE.RPMI_8226
LE.MOLT_4
LE.K_562
LE.HL_60
LE.CCRF_CEM
LC.NCI_H522
LC.NCI_H460
LC.NCI_H322M
LC.NCI_H23
LC.NCI_H226
LC.HOP_92
LC.HOP_62
LC.EKVX
LC.A549
CO.SW_620
CO.KM12
CO.HT29
CO.HCT_15
CO.HCT_116
CO.HCC_2998
CO.COLO205
CNS.U251
CNS.SNB_75
CNS.SNB_19
CNS.SF_539
CNS.SF_295
CNS.SF_268
BR.T47D
BR.MDA_MB_231
BR.MCF7
BR.HS578T
BR.BT_549
−4
−2
0
2
4
Observed (Z−scores)
Predicted (Z−scores)

NSC 740, Methotrexate (LOTO)

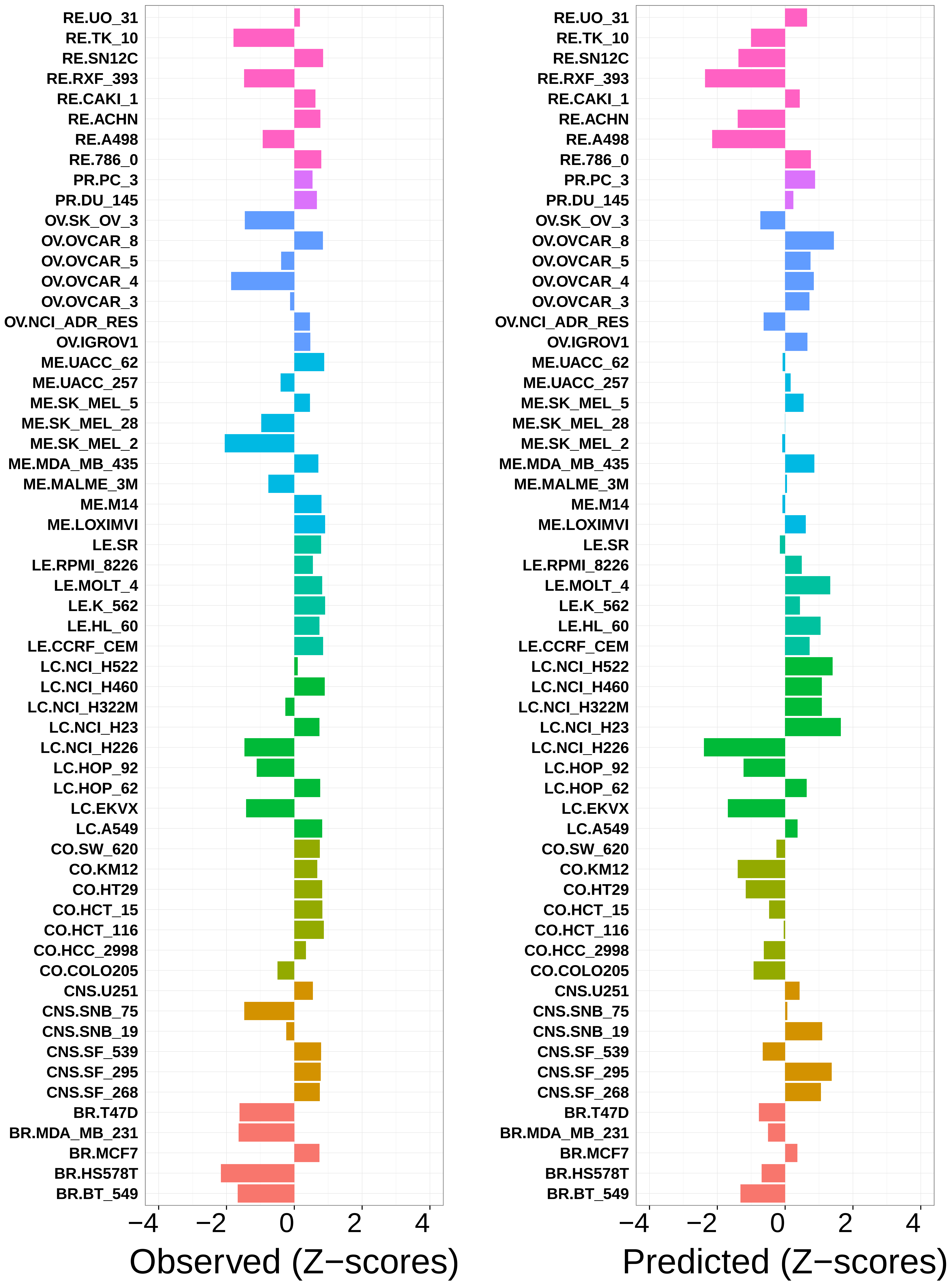

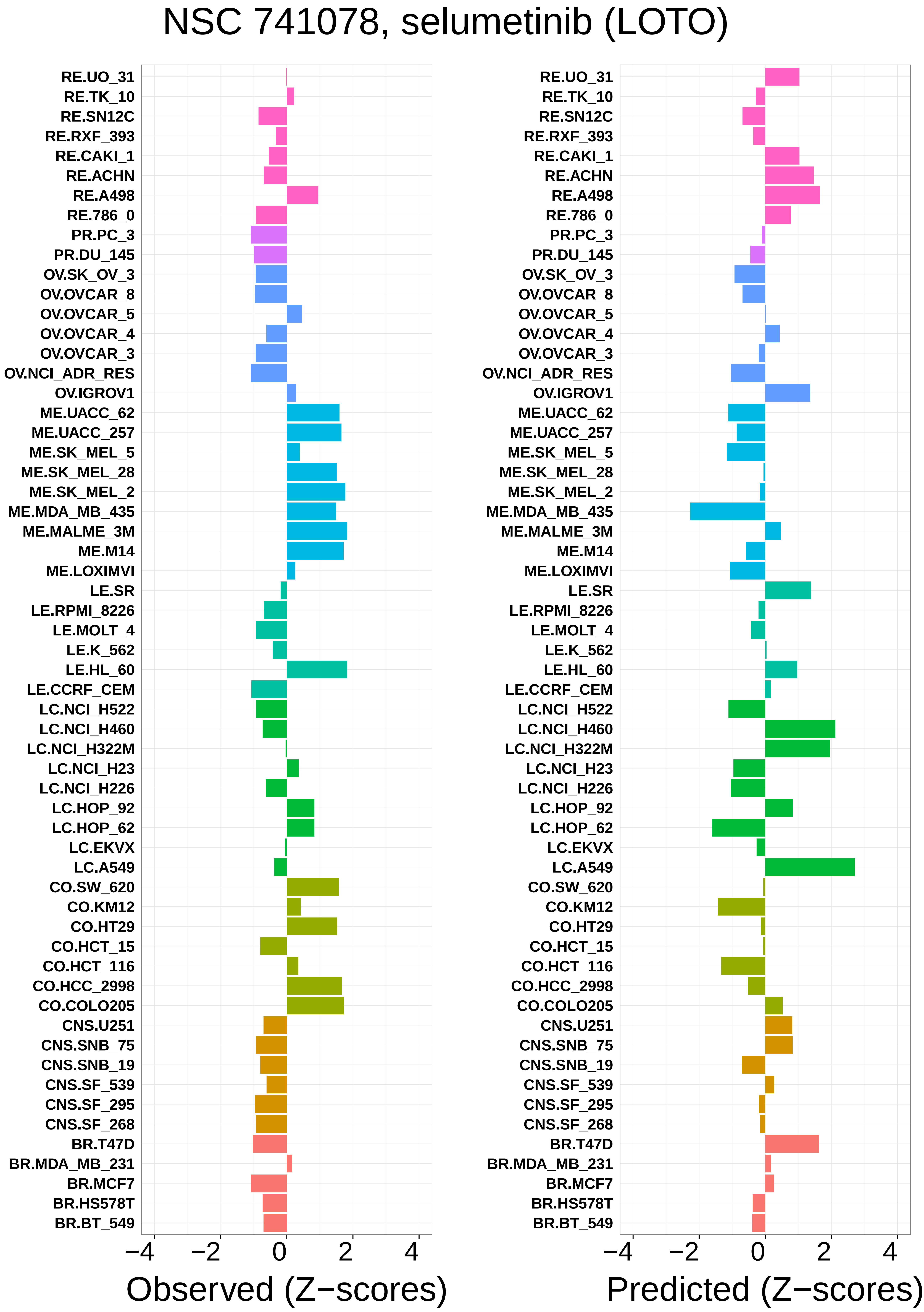
NSC 741078, selumetinib (LOTO)
RE.UO_31
RE.TK_10
RE.SN12C
RE.RXF_393
RE.CAKI_1
RE.ACHN
RE.A498
RE.786_0
PR.PC_3
PR.DU_145
OV.SK_OV_3
OV.OVCAR_8
OV.OVCAR_5
OV.OVCAR_4
OV.OVCAR_3
OV.NCI_ADR_RES
OV.IGROV1
ME.UACC_62
ME.UACC_257
ME.SK_MEL_5
ME.SK_MEL_28
ME.SK_MEL_2
ME.MDA_MB_435
ME.MALME_3M
ME.M14
ME.LOXIMVI
LE.SR
LE.RPMI_8226
LE.MOLT_4
LE.K_562
LE.HL_60
LE.CCRF_CEM
LC.NCI_H522
LC.NCI_H460
LC.NCI_H322M
LC.NCI_H23
LC.NCI_H226
LC.HOP_92
LC.HOP_62
LC.EKVX
LC.A549
CO.SW_620
CO.KM12
CO.HT29
CO.HCT_15
CO.HCT_116
CO.HCC_2998
CO.COLO205
CNS.U251
CNS.SNB_75
CNS.SNB_19
CNS.SF_539
CNS.SF_295
CNS.SF_268
BR.T47D
BR.MDA_MB_231
BR.MCF7
BR.HS578T
BR.BT_549
−4 −2 0 2 4
Observed (Z−scores)
Predicted (Z−scores)

NSC 745750, Tykerb (LOTO)

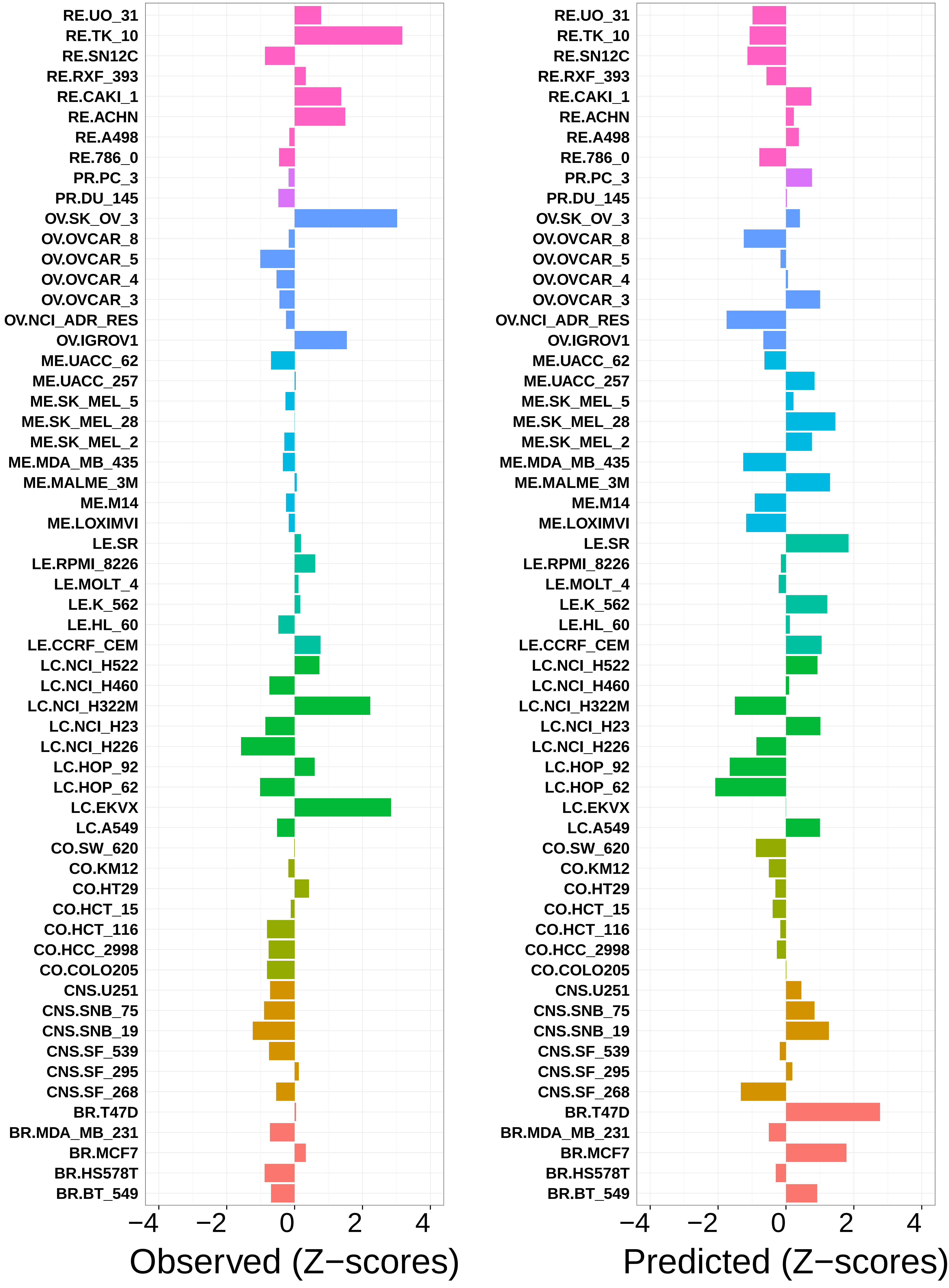

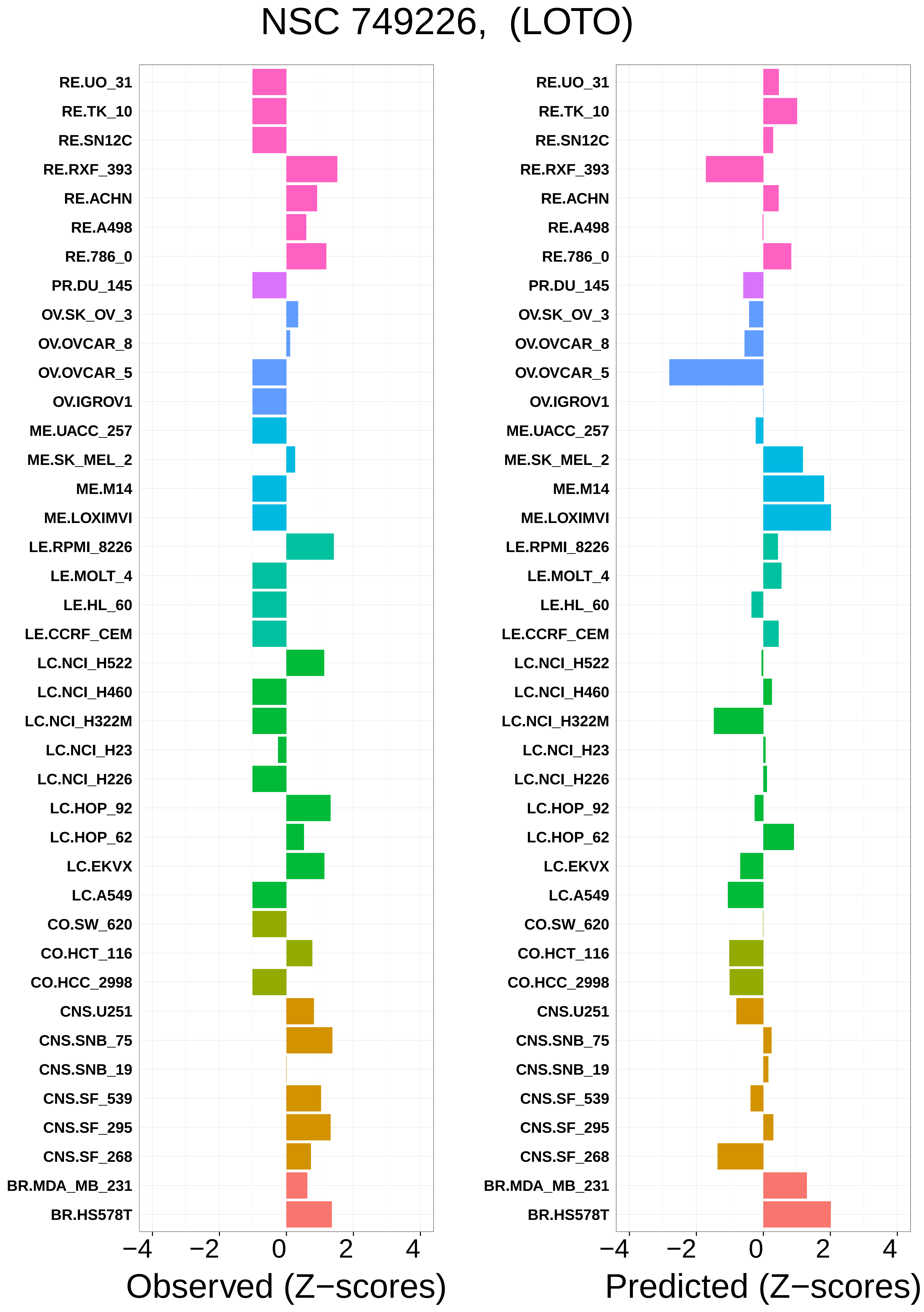
NSC 749226, (LOTO)
RE.UO_31
RE.TK_10
RE.SN12C
RE.RXF_393
RE.ACHN
RE.A498
RE.786_0
PR.DU_145
OV.SK_OV_3
OV.OVCAR_8
OV.OVCAR_5
OV.IGROV1
ME.UACC_257
ME.SK_MEL_2
ME.M14
ME.LOXIMVI
LE.RPMI_8226
LE.MOLT_4
LE.HL_60
LE.CCRF_CEM
LC.NCI_H522
LC.NCI_H460
LC.NCI_H322M
LC.NCI_H23
LC.NCI_H226
LC.HOP_92
LC.HOP_62
LC.EKVX
LC.A549
CO.SW_620
CO.HCT_116
CO.HCC_2998
CNS.U251
CNS.SNB_75
CNS.SNB_19
CNS.SF_539
CNS.SF_295
CNS.SF_268
BR.MDA_MB_231
BR.HS578T
−4 −2 0 2 4
Observed (Z−scores)
Predicted (Z−scores)

NSC 755, Mercaptopurine (LOTO)

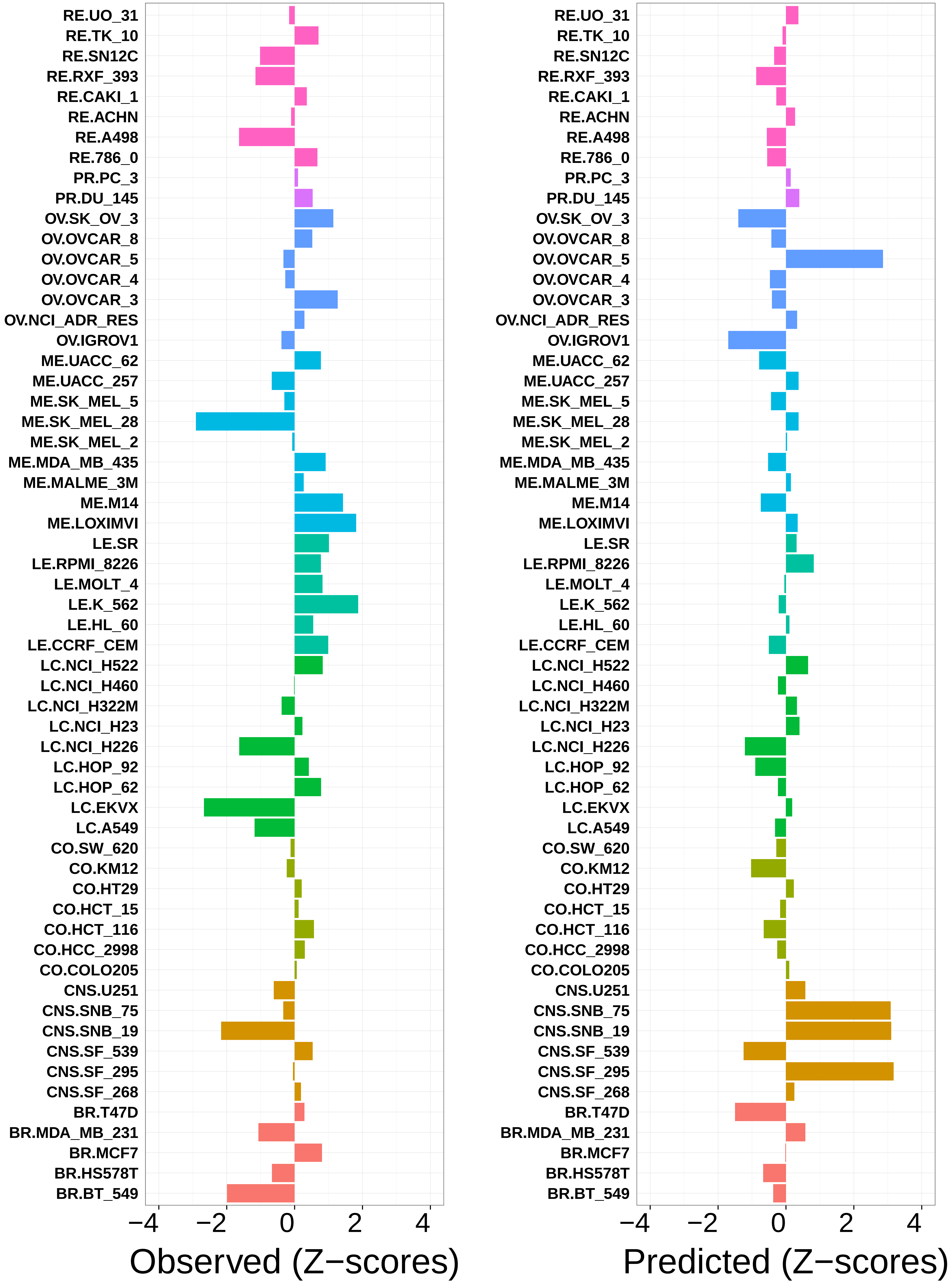

NSC 762, Mechlorethamine_Hydrochloride (LOTO)

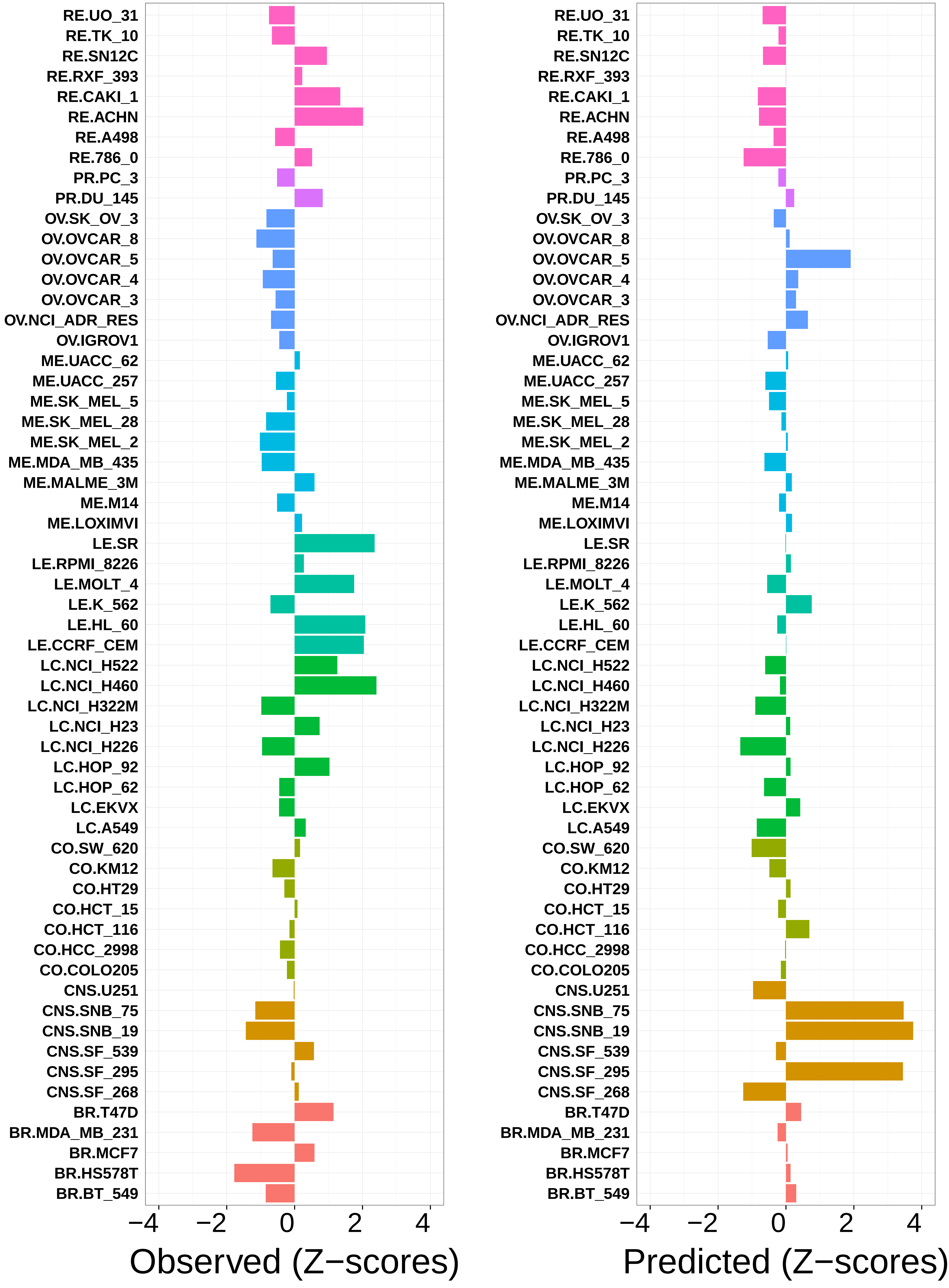

Supplement: Supplementary Data [file supp_btv529_barplots_all.pdf]
