# Supplementary material for: Improved large-scale prediction of growth inhibition patterns using the NCI60 cancer cell line panel
Source: Bioinformatics. 2015 Sep 8;32(1):85–95. doi: 10.1093/bioinformatics/btv529 (PMC4681992; doi:10.1093/bioinformatics/btv529)
Supplement: Supplementary Data [file supp_btv529_Drug_sensitivity_ICC_Supplementary_Figures_150722.pdf]

Supplementary Information

Improved large-scale prediction of growth inhibition patterns using the NCI60 cancer cell line panel

Isidro Cortes-Ciriano[1], Gerard JP van Westen[2], Guillaume Bouvier[1], Michael Nilges[1], John P. Overington[2], Andreas Bender[3*] and Thérèse E. Malliavin[1*]

(1) Institut Pasteur, Unité de Bioinformatique Structurale; CNRS UMR 3825; Département de Biologie Structurale et Chimie; 25, rue du Dr Roux, 75015 Paris, France.

(2) European Molecular Biology Laboratory European Bioinformatics Institute, Wellcome Trust Genome Campus, CB10 1SD, Hinxton, Cambridge, United Kingdom.

(3) Unilever Centre for Molecular Science Informatics, Department of Chemistry, University of Cambridge, Cambridge, United Kingdom.

Corresponding authors:

Thérèse E. Malliavin; E-mail: terez@pasteur.fr; Phone: +33 1 40 61 34 75

Andreas Bender; E-mail: ab454@cam.ac.uk; Phone: +44 (1223) 762 983

Index

Pages 3-25 Supplementary Figures

Pages 26-28 Suppplementary Text

Page 29 Bibliography

Supplementary Figures

Supplementary Figure S1.

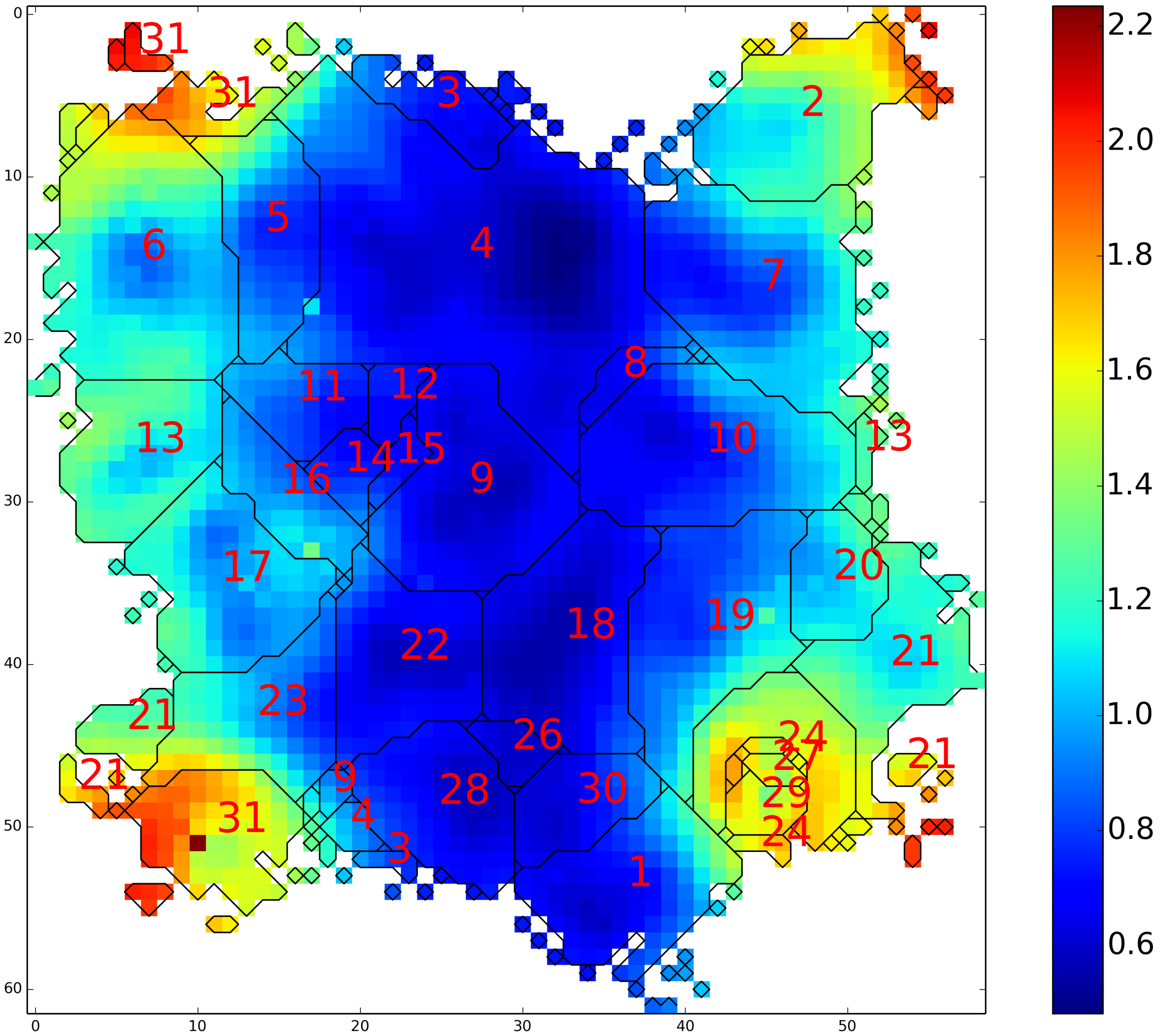

U-matrix for the SOM used to cluster the compounds. Black lines delimit the 31 clusters defined, whereas red labels indicate the cluster number. The similarity between each neuron and its 8 neighboring neurons defines the color code: blue corresponds to high similarity (homogeneous areas), and red corresponds to low similarity (heterogeneous areas). Therefore, clusters presenting blue and red neurons exhibit higher levels of intra-cluster chemical diversity.

Supplementary Figure S2.

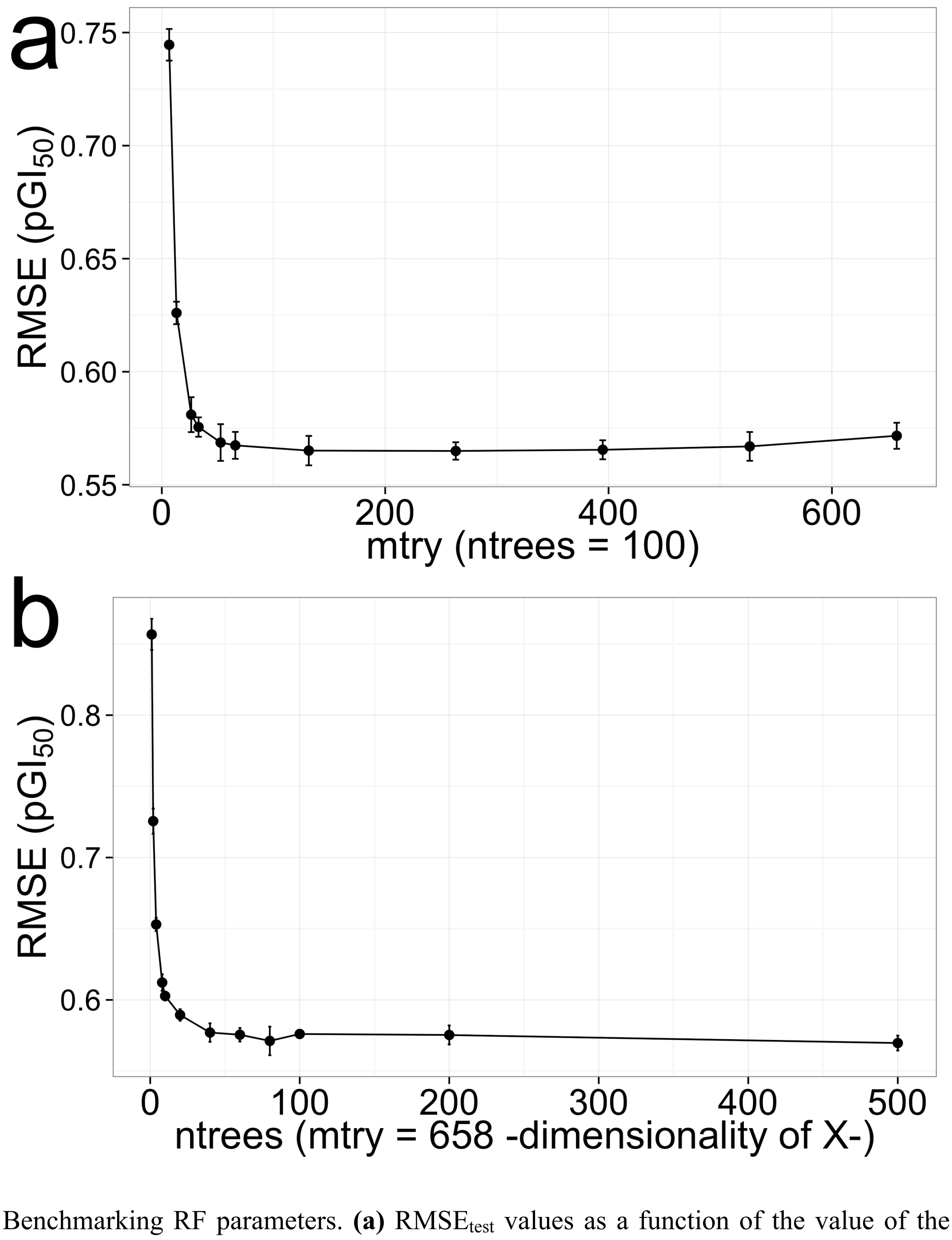

Benchmarking RF parameters. **(a)** $RMSE_{test}$ values as a function of the value of the parameter *mtry*. Converge is reached for *mtry* values of ~50 onwards. For this calculation, all RF models

were composed of 100 trees. **(b)** $RMSE_{test}$ values as a function of the number of trees (*ntrees*) in the forest. Converge is reached when the number of trees is 40 or higher. For this calculation, all RF models were trained with *mtry* values equal to the dimensionality of the input space, namely 658. All 10-fold CV PGM models used for the results reported in **(a)** and **(b)** were trained on the *uncorrelated bioactivities 0.5* data set using (i) Morgan fingerprints and (ii) the data set view "G.t.l Kin." as input features to the model. These results indicate that the parameter values used in this study, namely (i) *mtry*: dimensionality of the input space, and (ii) *ntrees*: 100, guarantee the convergence of model performance.

Supplementary Figure S3.

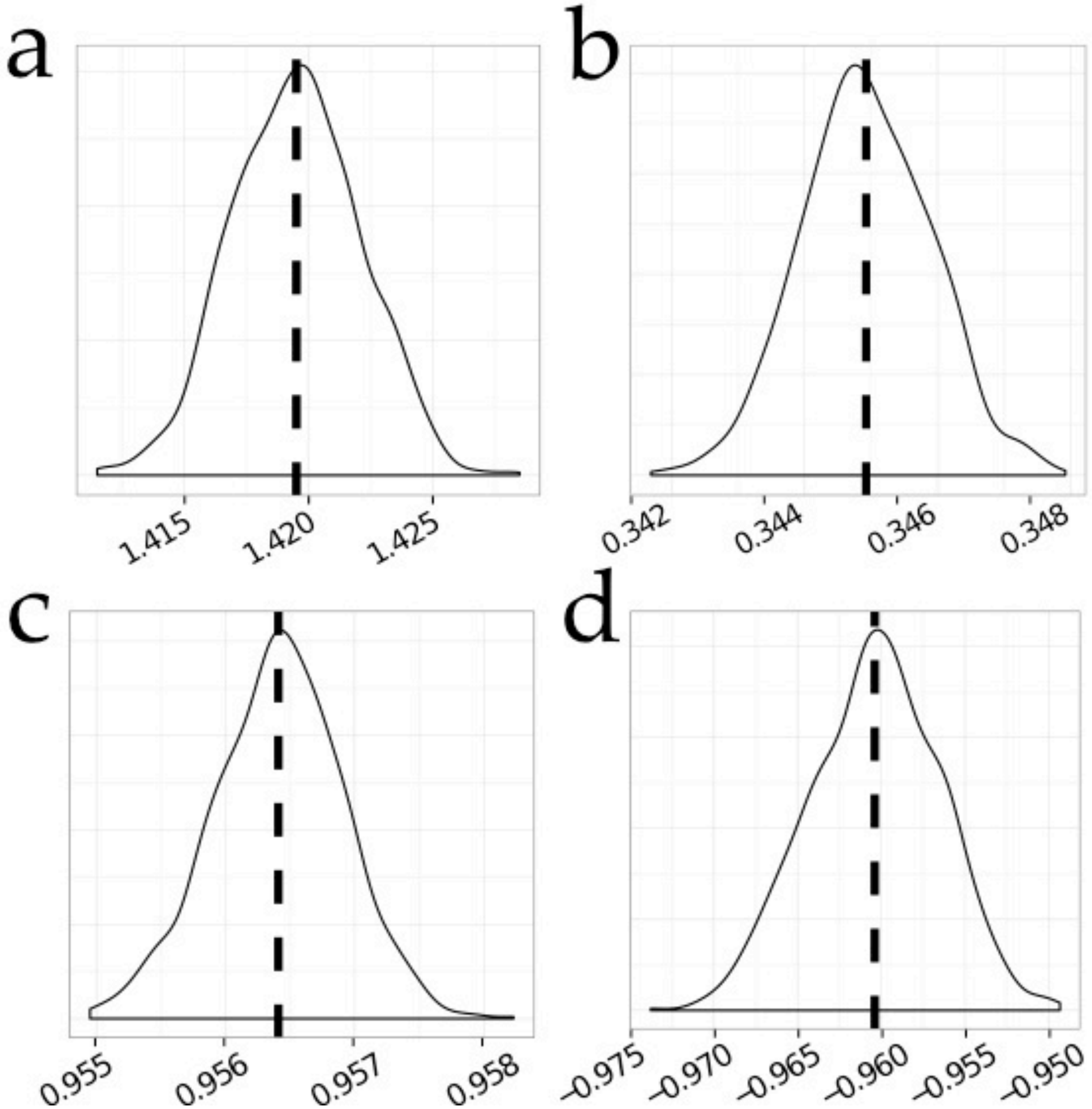

Distribution of respective maximum and minimum $RMSE_{test}$ (**a,b**) and $R^2_{0\ test}$ (**c,d**) values for the *complete* data set. Average maximum and minimum values of 1.42/0.35 and 0.96/-0.96, were obtained respectively for $RMSE_{test}$ / $R^2_{0\ test}$ with the simulated data. The performance of the 10-fold CV PGM models on the test set was in agreement with the uncertainty of the experimental measurements, as mean $RMSE_{test}$ and $R^2_{0\ test}$ values of 0.40 +/- 0.00 pGI_{50} unit and 0.83 +/- 0.00 (with n = 10 models) were obtained. These values are between the two extreme, maximum and minimum, theoretical $RMSE_{test}$ and $R^2_{0\ test}$ values.

Supplementary Figure S4.

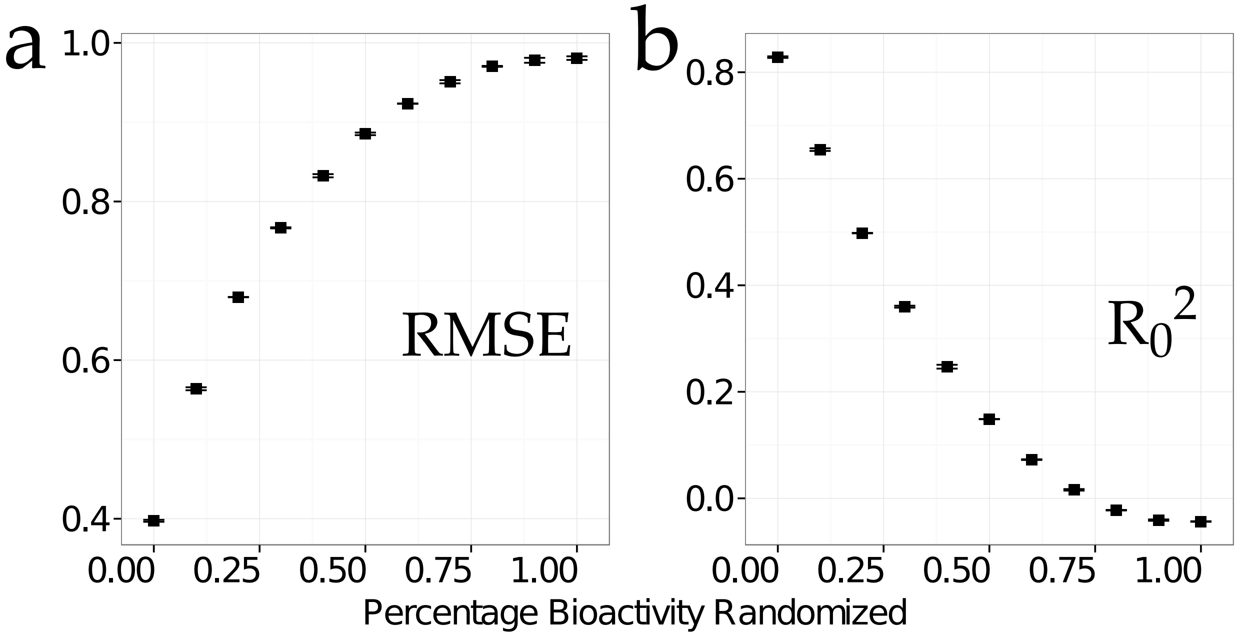

Y-scrambling validation. Mean (+/- std) $RMSE_{test}$ (**a**) and $R^2_{0\ test}$ (**b**) values were calculated for the observed against the predicted bioactivities on the test set calculated with models trained on pGI_{50} values increasingly randomized (*n*=3). $R^2_{0\ test}$ values become negative when 75% of the bioactivity values are randomized. These data suggest that the relationships established by the 10-fold CV PGM models between compound and cell line descriptors, and the pGI_{50} values did not arise from chance correlations.

Supplementary Figure S5.

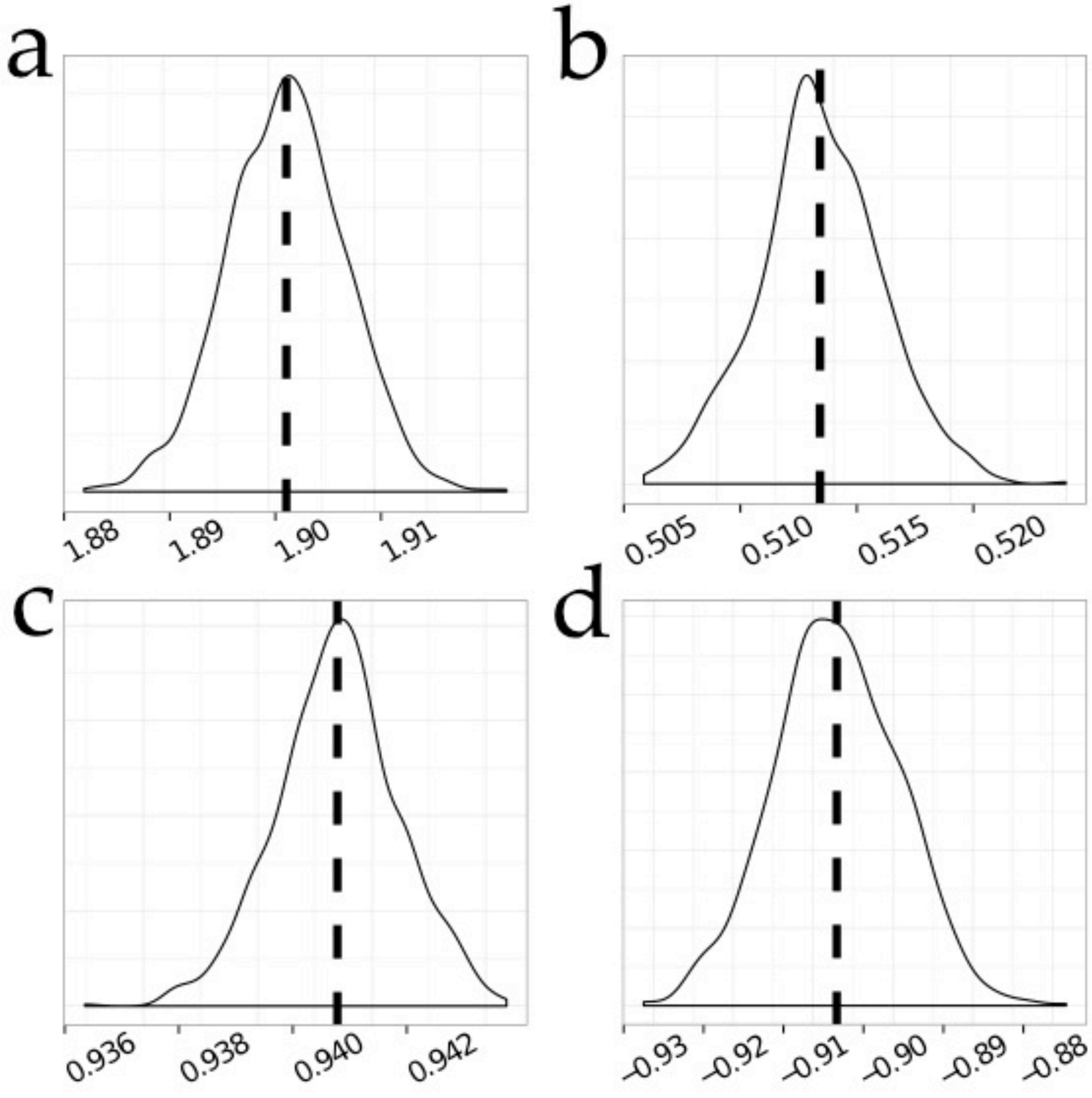

Distribution of respective maximum and minimum $RMSE_{test}$ (**a,b**) and $R^2_{0\ test}$ (**c,d**) values for the *uncorrelated bioactivities 0.5* data set. Average maximum and minimum values of 1.90/0.54 and 0.94/-0.90 were obtained respectively for $RMSE_{test}$/ $R^2_{0\ test}$ with the simulated data. The performance of 10-fold CV PGM models was in agreement with the uncertainty of the experimental measurements, as mean $RMSE_{test}$ and $R^2_{0\ test}$ values of 0.58 pGI_{50} unit and 0.79 were obtained. These values are between the two extreme, maximum and minimum, theoretical $RMSE_{test}$ and $R^2_{0\ test}$ values.

Supplementary Figure S6.

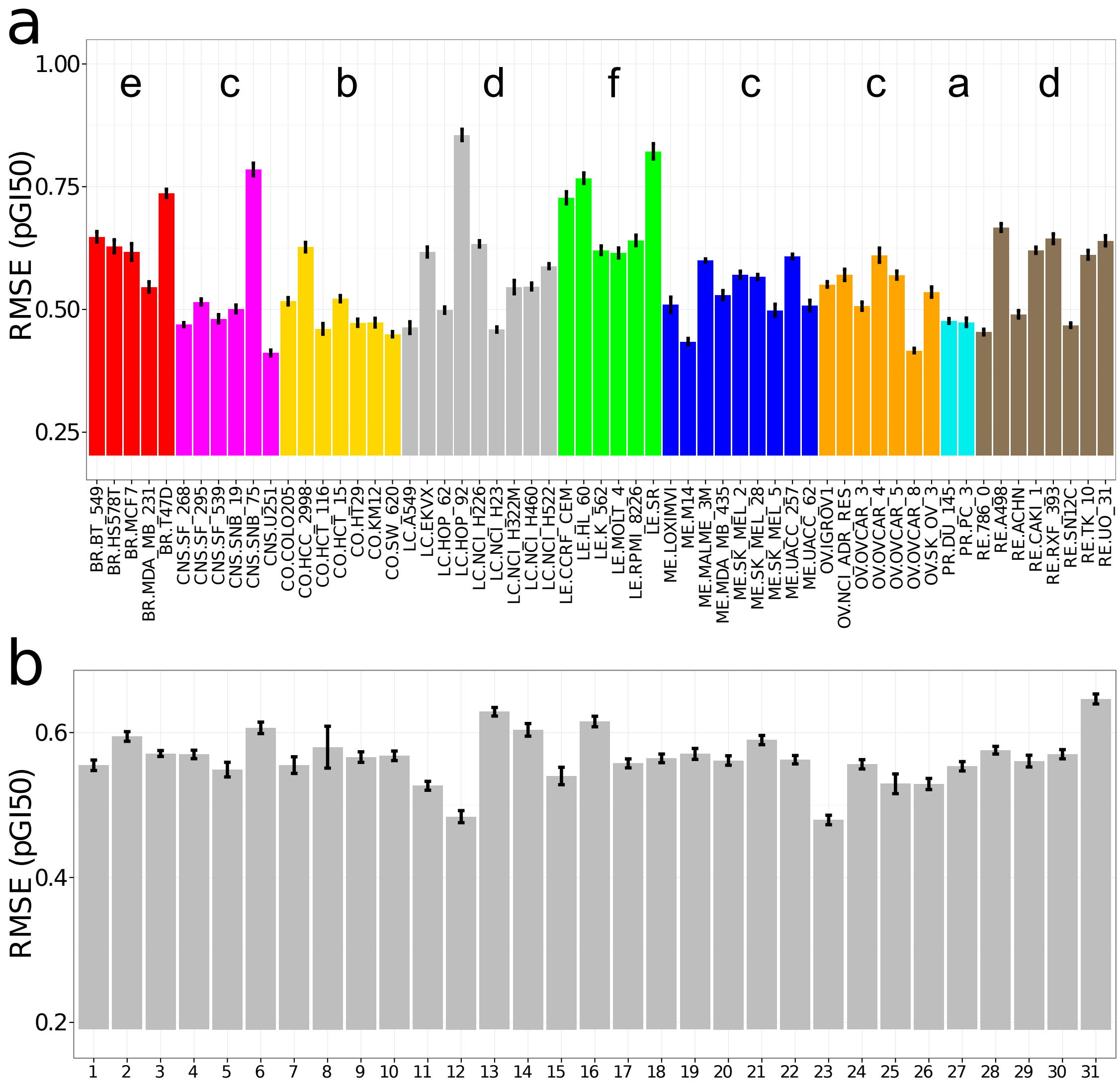

Interpolating compound bioactivities to novel cell lines, tissues, and chemical clusters. (a) Cell line-averaged $RMSE_{test}$ values ranged from 0.41 +/- 0.01 (U251) to 0.86 +/- 0.01 pGI_{50} unit (HOP-92). We found significant differences for tissue-averaged performance (Tukey's HSD, $P < 1 \times 10^{-16}$), with $RMSE_{test}$ values ranging from 0.48 +/- 0.01 (prostate) to 0.70 +/- 0.01 (leukemia) pGI_{50} unit. Cell lines originated from the same tissue are depicted in the same color (breast: red, central nervous system: magenta, colon: yellow, lung cancer: grey, leukemia: green, melanoma: blue, ovarian: orange, prostate: cyan, renal: brown). We did not observe significant differences in

tissue-averaged performance for tissues labeled with the same letter. (b) One-way ANOVA among the 31 chemical clusters ($P > 0.05$), with compound cluster-averaged $RMSE_{test}$ values in the 0.48 +/- 0.01 and 0.65 +/- 0.01 pGI_{50} unit range. This analysis illustrates that the models do not constantly favor specific chemical clusters, thus making it possible to interpolate compound bioactivities across the chemical space covered by the data at the same level of statistical significance. By contrast, interpolating on the cell line side depends significantly on the tissue source.

Supplementary Figure S7.

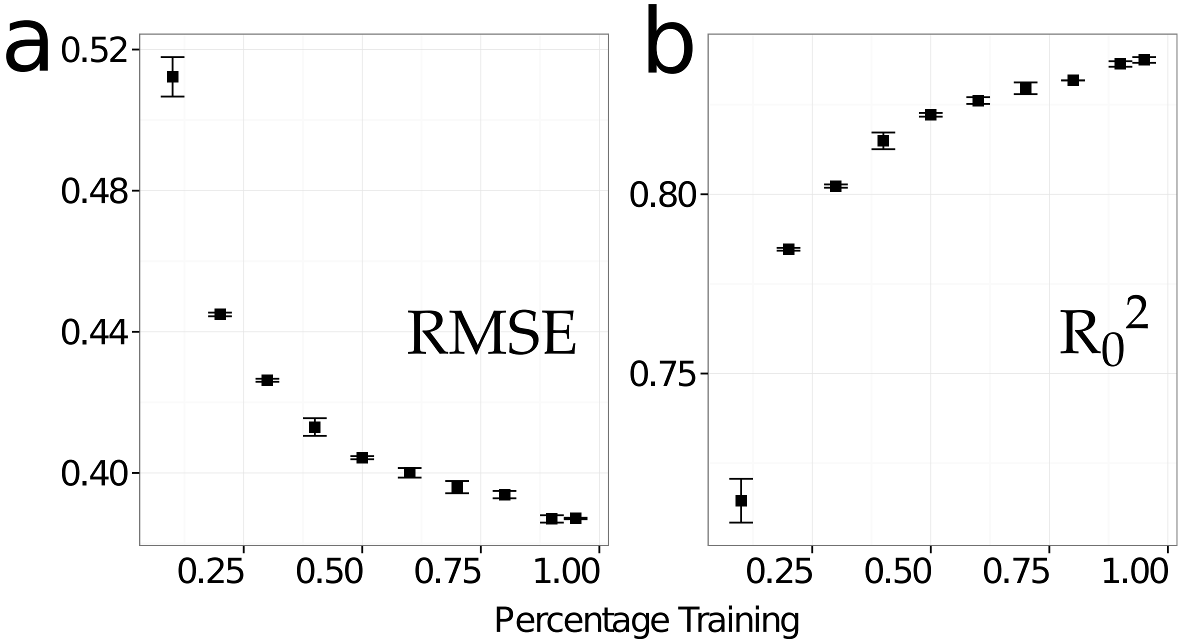

Learning curves. Mean (+/- std) $RMSE_{test}$ (**a**) and $R^2_{0\ test}$ (**b**) values were calculated for the observed against the predicted bioactivity values on the test set calculated with n=3 models obtained using training sets covering an increasingly higher fraction of the *complete* data set. Models trained on 5% of the data set exhibited a mean $RMSE_{test}$ value of 0.52 pGI_{50} unit, which decreased till 0.39 pGI_{50} unit when 95% of the data-points were included in the training set. These data suggest that 10-fold CV PGM models exhibit high interpolation capabilities. In practice, the compound-cell line interaction matrix could be completed with *in silico* predictions, with a $RMSE_{test}$ value of 0.39 pGI_{50} unit.

Supplementary Figure S8.

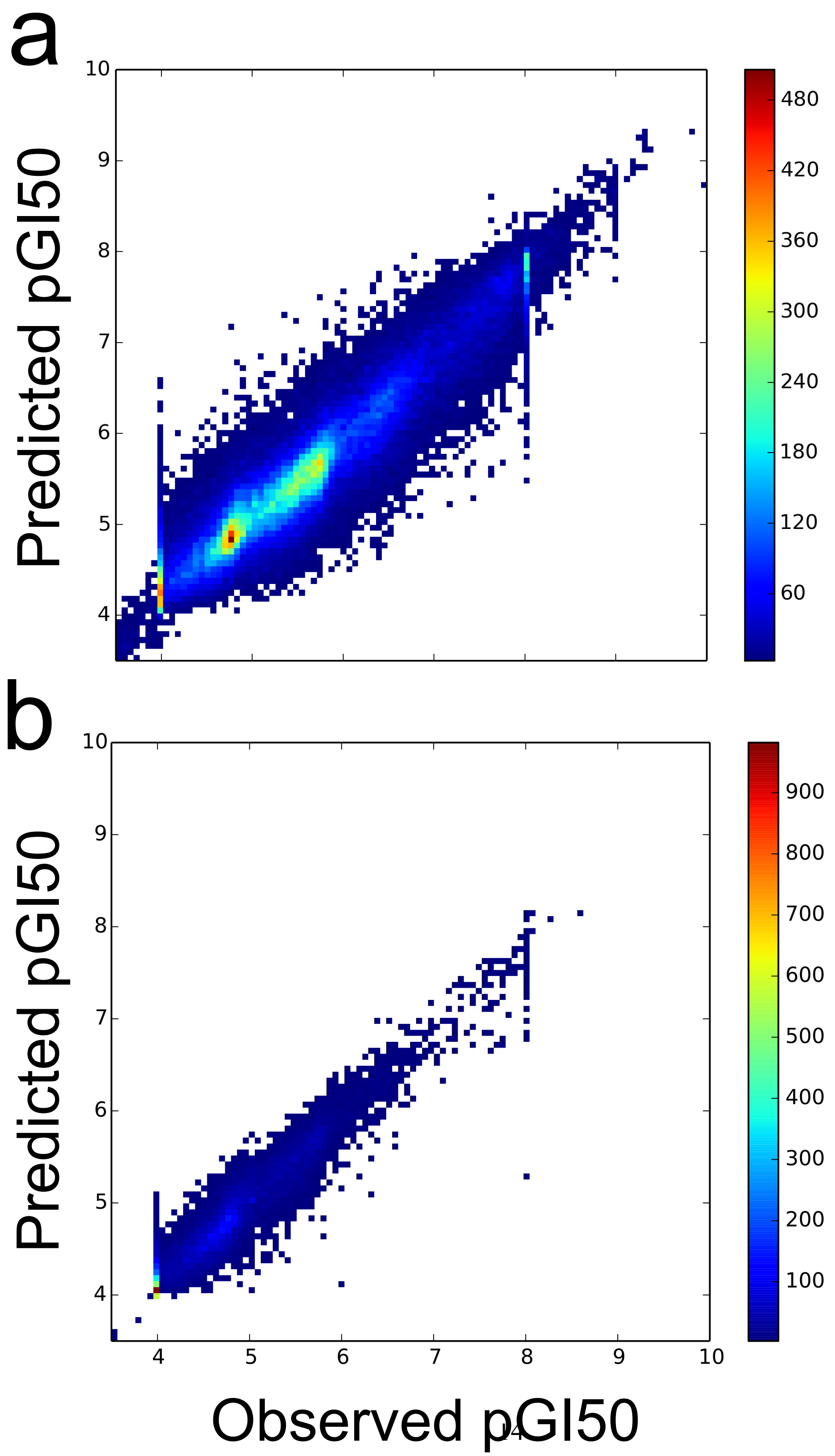

Correlation between observed and predicted pGI_{50} values. Density correlation plot corresponding to the observed against predicted pGI_{50} values on the test set for: (**a**) the LOTO model for melanoma ($RMSE_{test}$ and $R^2_{0\ test}$ values of 0.43 pGI_{50} unit and 0.80), and (**b**) the LOCO model for the melanoma cell line SK-MEL-5 ($RMSE_{test}$ and $R^2_{0\ test}$ values of 0.37 pGI_{50} unit and 0.87). The color bar indicates the density of points at each region of the plot. For the rest of LOCO and LOTO models comparable results were obtained (Table S11), with bioactivity values correctly predicted along the whole bioactivity range.

Supplementary Figure S9

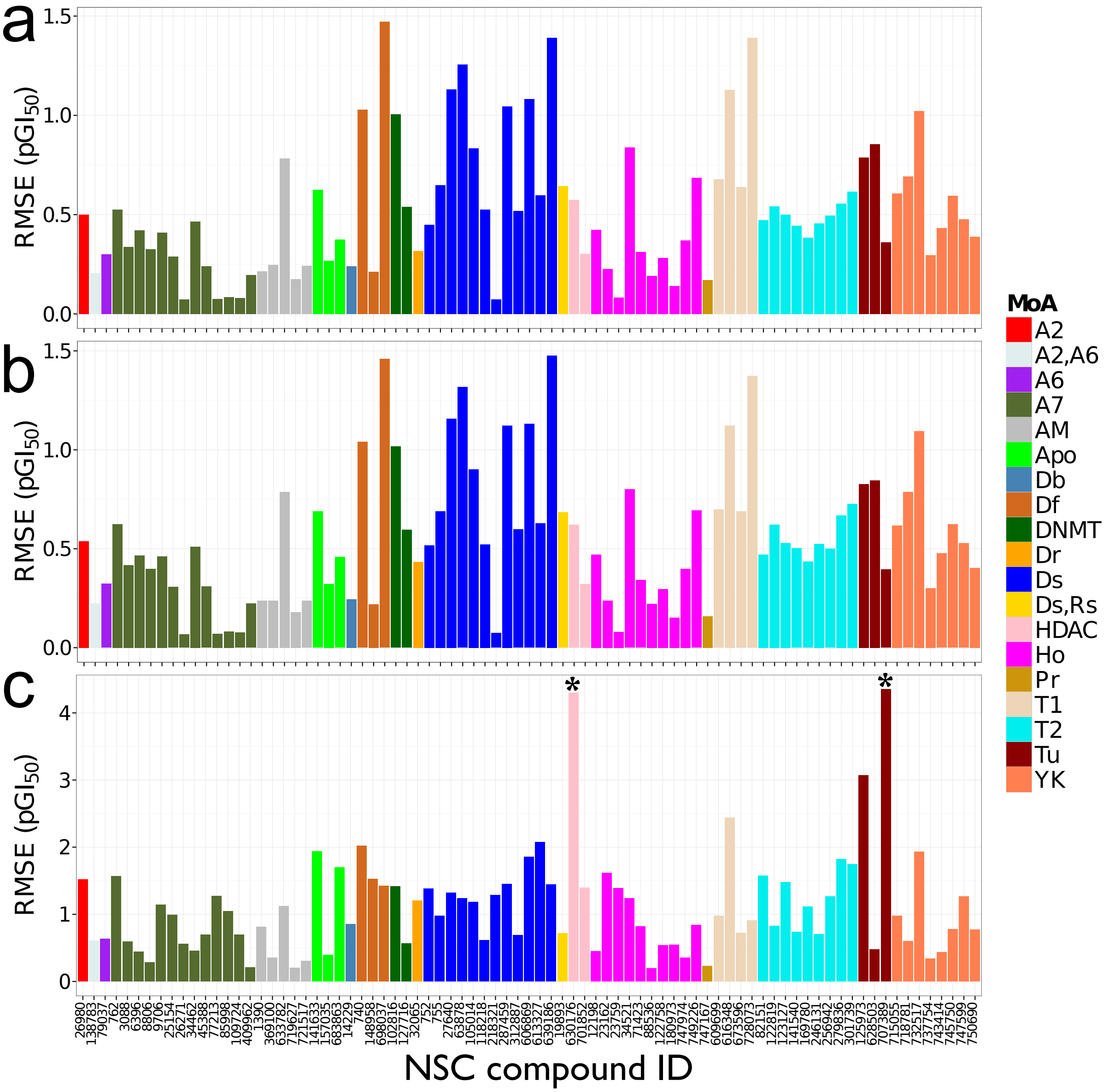

Correlation between observed and predicted pGI_{50} values for the 81 drugs present in the *complete* data set for the following model validation scenarios: (a) LOCO, (b) LOTO, and (c) LOCCO. The *x*-axis reports the drug NSC identifiers. Compounds discussed in the main text, namely NSC 630176 and NSC 707389, are marked with asterisks. Bars are colored according to drug mechanism of action (MoA). The abbreviations of the mechanisms of action are: A2:

alkylating at N-2 position of guanine; A7: alkylating at N-7 position of guanine; AM: antimetabolite; Ang: angiogenesis; Apo: apoptosis inducer; Db: DNA binder; Df: antifolates; DNMT: DNA methyltransferase inhibitor; Dr: ribonucleotide reductase inhibitor; Ds: DNA synthesis inhibitor; HDAC: Histone deacetylase; Ho: hormone; P90: hsp90 binder; PI3K: PI3kinase; PKC: Protein kinase C; ROS: reactive oxygen species; RSTK: serine/threonine kinase inhibitor; T1: topoisomerase 1 inhibitor; T2 : topoisomerase 2 inhibitor; Tu: tubulin-active antimitotic; YK: tyrosine kinase inhibitor.

Supplementary Figure S10

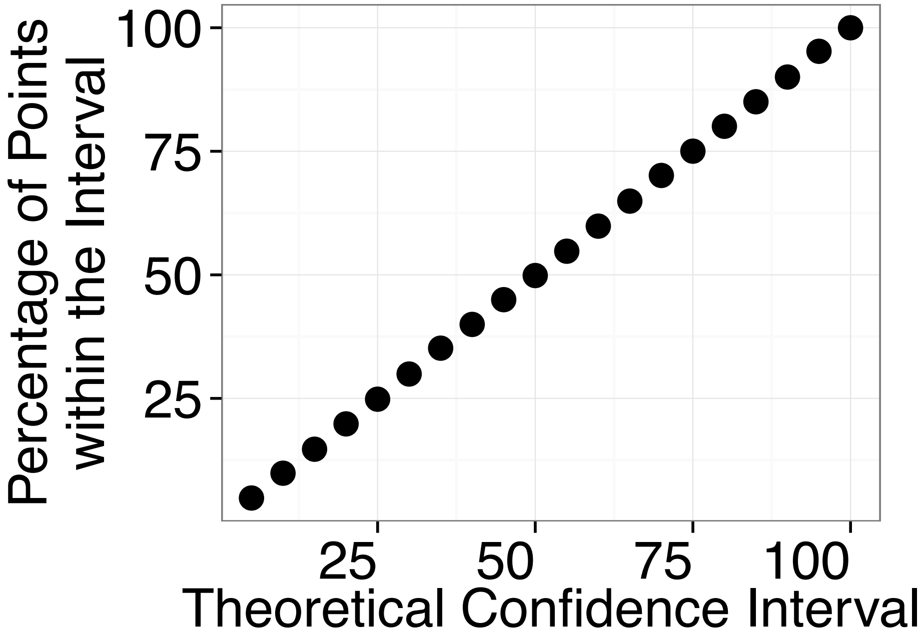

Validation of conformal prediction. For each confidence level (ε), represented in the *x*-axis, the number of data-points in the test set which true value lay within the predicted interval is calculated, *y*-axis. The high Spearman's r_s is likely due to the large size of the test set (188,366 data-points) and to the fact that the CI produced by conformal prediction are always valid (Norinder *et al.*, 2014). These data indicate that the modeling framework combining PGM models and conformal prediction is more information rich than what would be possible with only point prediction algorithms.

Supplementary Figure S11.

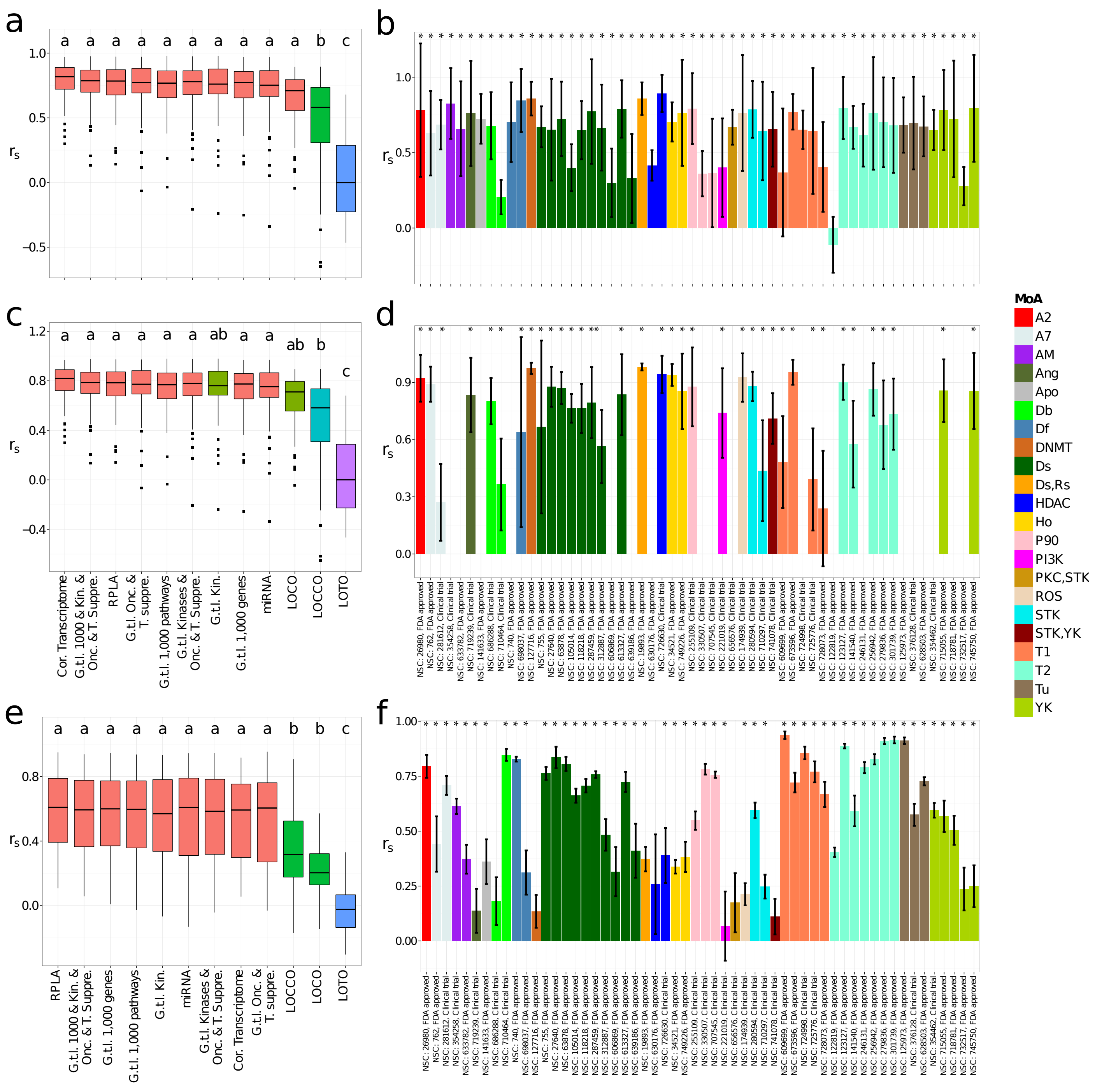

Consistency between the pathway-drug associations calculated with the experimental and the predicted bioactivity values. For each pathway, we fitted a linear model controlled by tissue source, where the average pathway expression was considered as predictor of drug sensitivity. Box plots report the distribution of Spearman's rs values between the β_p coefficients estimated with the experimental and the predicted values over the 56 drugs present in the *uncorrelated bioactivities 0.5* data set, using all pathway-drug associations (FDR < 20%), (**a**) or only significant associations (**c**), as estimated in the *uncorrelated bioactivities 0.5* data set. Bar plots representing the drug-averaged Spearman's r_s coefficients calculated with all (**b**) or with only significant (**d**) pathway-drug associations, averaged over the models labeled with "a" in (**a**). Missing bars in (**d**) correspond to drugs for which we did not find significant drug-pathway associations. (**e**) Data view-averaged Spearman's r_s coefficients for patterns of growth inhibition calculated with the experimental and the predicted values. (**f**) Bar plot reporting the drug-averaged Spearman's r_s coefficients for the patterns of growth inhibition calculated with the observed and the predicted bioactivities. Data views sharing a letter label and color in (**a,c,e**) perform at the same level of statistical significance. Significance for the Spearman's r_s in (**b,d,f)** is represented with an asterisk if two-sided *P* value < 0.05, for the Spearman's r_s coefficients calculated with the predictions generated with a model trained on the "G.t.l. 1,000 genes" data view. Bars in (**b,d,f)** are colored according to compound MoA.

Abbreviations of mechanisms of action: MoA: Mechanism of action; A2: alkylating at N-2 position of guanine; A7: alkylating at N-7 position of guanine; AM: antimetabolite; Ang: angiogenesis; Apo: apoptosis inducer; Db: DNA binder; Df: antifolates; DNMT: DNA methyltransferase inhibitor; Dr: ribonucleotide reductase inhibitor; Ds: DNA synthesis inhibitor; HDAC: Histone deacetylase; Ho: hormone; P90: hsp90 binder; PI3K: PI3kinase; PKC: Protein kinase C; ROS: reactive oxygen species; RSTK: serine/threonine kinase inhibitor; T1: topoisomerase 1 inhibitor; T2 : topoisomerase 2 inhibitor; Tu: tubulin-active antimitotic; YK: tyrosine kinase inhibitor.

Supplementary Figure S12.

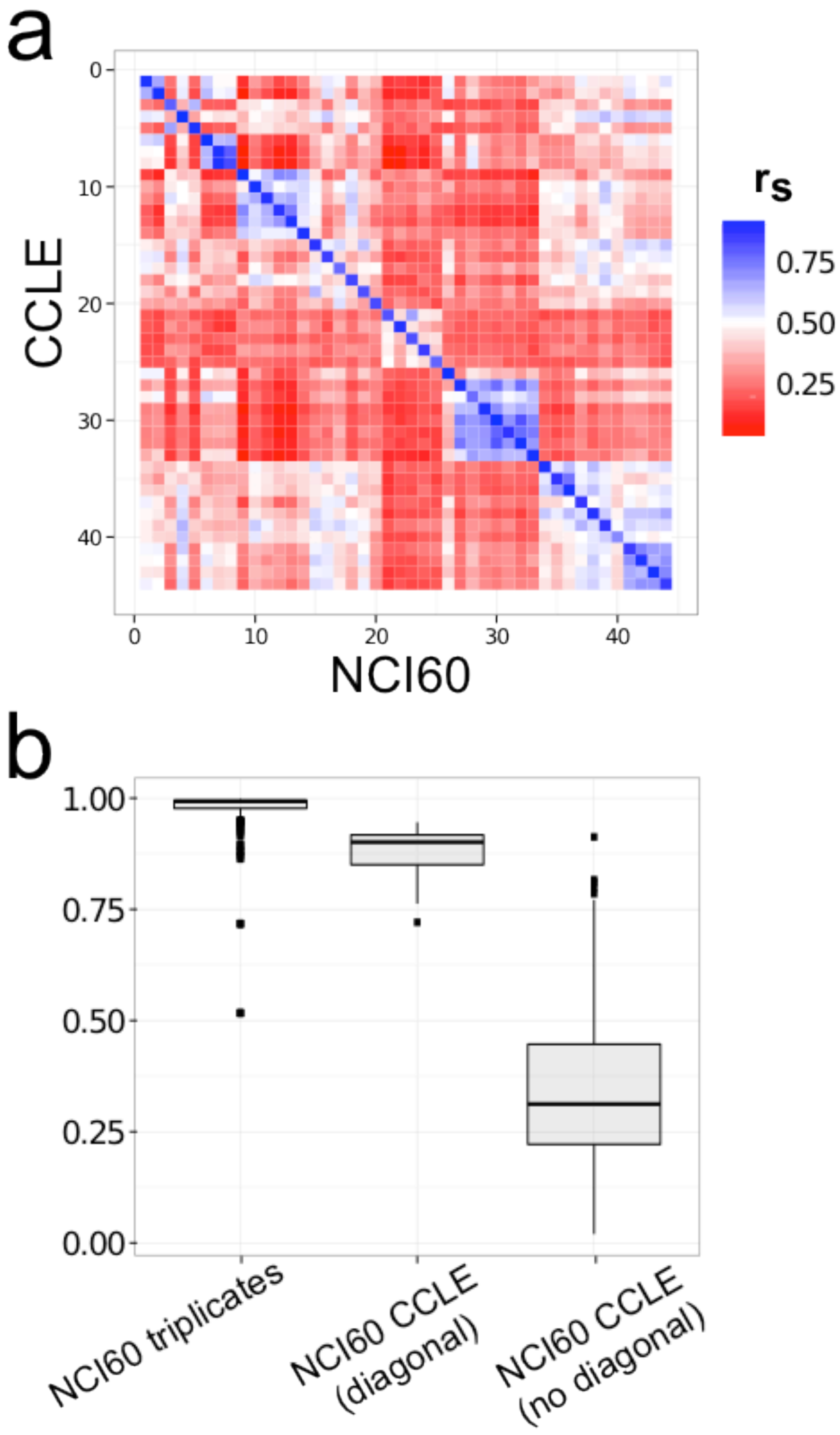

Correlation of gene expression profiles for the 44 cell lines present in both the NCI60 panel and the Cancer Cell Line Enclycopedia (CCLE). (**a**) Pairwise Spearman's r_s correlation of the 1,000 most varying genes between the DTP-NCI60 and the CCLE data sets. Both data sets share 44 cell lines. The correlation between the gene expression profiles of identical cell lines is higher than 0.8 in all cases (diagonal of the matrix), with a median Spearman's r_s value close to 0.875. (**b**) The fist box plot on the left reports the Spearman's r_s correlation, above 0.98, between the gene transcript levels calculated in triplicates for the NCI60 cell lines. The box plot in the middle corresponds to the correlation between the gene expression profiles of the cell lines found in both the CCLE and the NCI60 data set (diagonal of the matrix in (**a**)). The average Spearman's r_s correlation is close to 0.875. The third boxplot reports the Spearman's r_s correlation of different cell lines (the non-diagonal elements of the matrix in (**a**)). The high correlation between gene expression profiles for the cell lines present in both the CCLE and the NCI60 cell line panel, indicates that the PGM models reported in this study could be extended to the CCLE.

Supplementary Figure S13

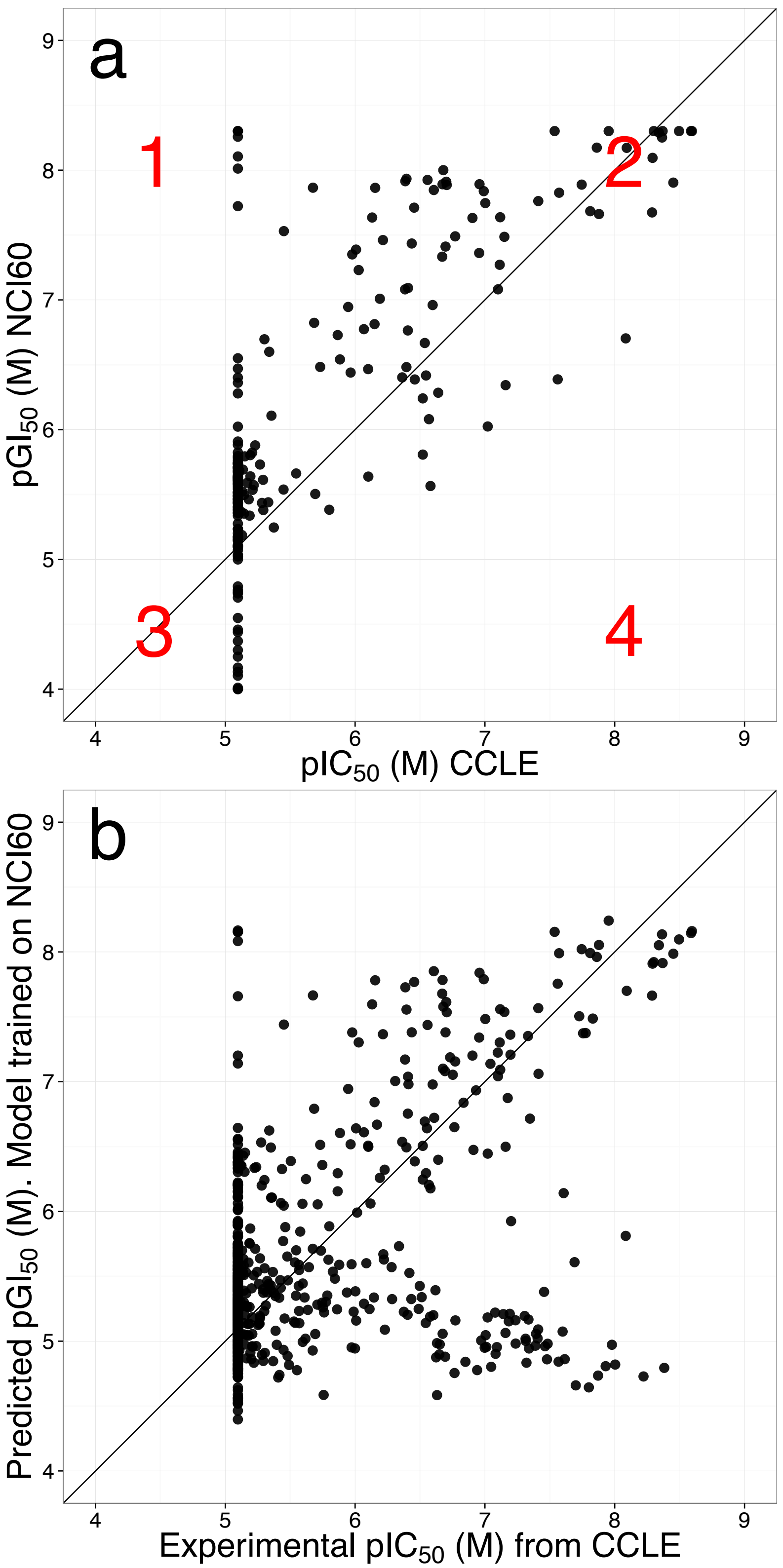

Correlation between *in vitro* drug sensitivity data from the NCI60 and CCLE. The subset of the NCI60 data used in our study and the CCLE share 44 cell lines and 8 drugs, namely: Erlotinib, Lapatinib, Nilotinib, Sorafenib, Paclitaxel, Irinotecan and Topotecan. We could retrieve bioactivity data from both data sets for a total of 208 compound-cell line pairs. (**a**) The RMSE value for (i) the pGI_{50} values from the NCI60 data set, against (ii) the pIC_{50} values from the CCLE is 0.87 $\log_{10}$ units. This low concordance was expected given the different assays used to screen the NCI60 and CCLE panels, namely sulforhodamine B (SRB) and CellTiter-Glo® Luminescent Cell Viability Assay from Promega, respectively. Therefore, three cases are possible when comparing data from the NCI60 and the CCLE data sets. In the first case, low compound concentration is sufficient to stop cell proliferation whereas high compound concentration is required to decrease cellular metabolic activity: this case is labeled with number 1 in red in the Figure. In the second case, cell proliferation and cellular metabolic activity are correlated and similar IC_{50} values are observed using both assays: this case is labeled with number 2 and 3 in red in the Figure. In the third case, low compound concentration is required to decrease cellular metabolic activity whereas high compound concentration is required to stop cell proliferation: this case is labeled with number 4 in red in the Figure, but does not correspond to a populated case for NCI60 and CCLE data sets. (**b**) Observed pIC_{50} values from the CCLE vs predicted values with a model trained on the NCI60 data set for the 8 drugs and 44 cell lines shared between the two data sets. Overall, low correlation is found between the experimental data and the predictions. The RMSE value between experimental and predicted bioactivities is 0.87 $\log_{10}$ units. This value is similar to the RMSE value obtained in (**a**), indicating that high predictive power cannot be attained given the low concordance of the sensitivity data from the CCLE and NCI60 data sets. This low level of concordance illustrates the statement of Haibe-Kains et al., 2013 about the inconvenience of validating a model trained on a given data set on data obtained with a different experimental setup.

Supplementary Figure S14

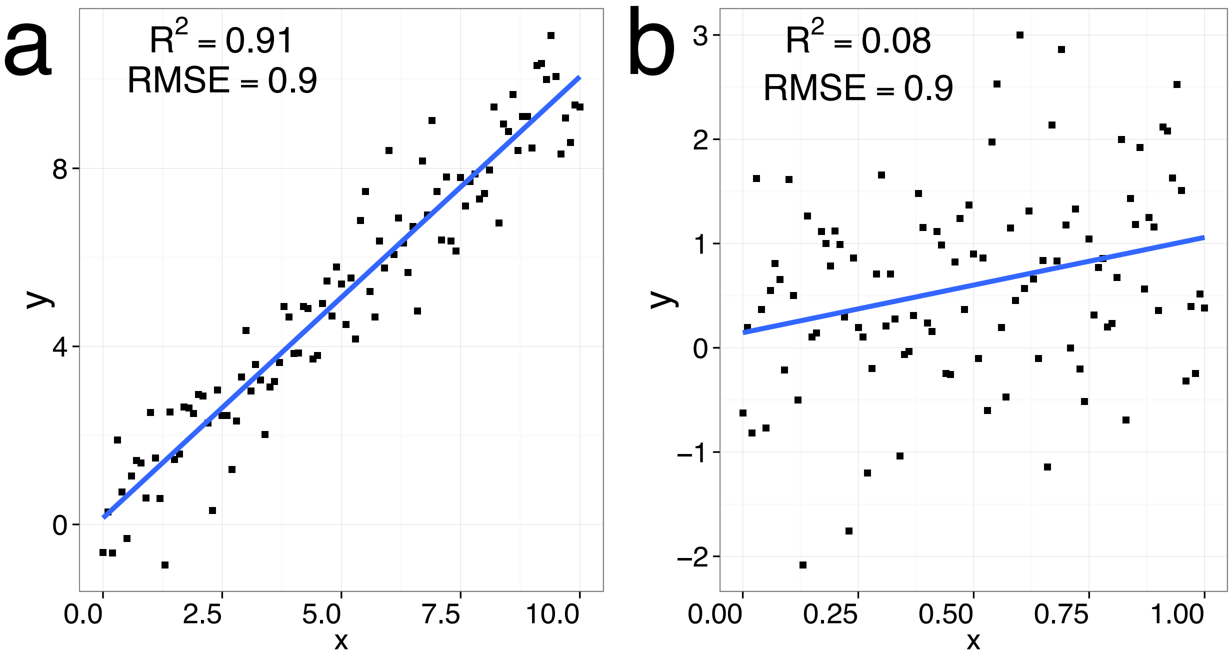

Toy example showing the influence of the range of the response variable (*e.g.* bioactivities) on R^2 values. (**a**) R^2 and RMSE values of 0.91 and 0.90, respectively, are obtained when the response values range from 0 to 10 (arbitrary units). (**b**) By contrast, the R^2 drops to 0.08 when the response value ranges from 0 to 1. Note that in both cases the RMSE values are the same, namely 0.90. To simulate y, random noise with mean 0 and standard deviation equal to 1 was added to x. The noise added was the exactly the same in both cases, namely (**a**) and (**b**). This example illustrates that low R^2 values obtained with LOTO, LOCO and, especially LOCCO models, do not necessarily imply that the predictions are inaccurate. LOCCO and Leave-One-Compound-Out are particularly prone to this situation, as, in many cases, the activities of a given compound across a cell line panel do not present a dynamic range of response. Thus, in these cases model predictive power should be based on RMSE values.

Supplementary Text

To compare our modeling approach to previous studies (Menden *et al.*, 2013; Ammad-ud-din *et al.*, 2014), we applied PGM to two additional datasets, namely the CCLE and GDS. The same metrics to evaluate model performance were used, namely RMSE and R^2. PGM models were trained on (i) Morgan fingerprints and (ii) the transcript levels for the genes displaying the highest variance across the cell line panel. Although this combination of descriptors has led to the most predictive models on the NCI60 panel, other combinations of descriptors might be more suitable for other data sets.

Preparation of the GDSC data set

MAS5-normalized gene transcript levels, measured with HT-HGU133A Affymetrix whole genome array, were downloaded from the GDSC website (http://www.cancerrxgene.org/) on February 16th 2015. Compound IC50 values were converted to log10 IC50 (µM) values in order to enable the comparison of our results with previous studies (Menden *et al.*, 2013; Ammad-ud-din *et al.*, 2014). In addition, we converted the IC50 values to pIC50 values, *i.e.* –log10 IC50 (M).

10-fold cross-validation was used to assess the interpolation power of the models, leading to $RMSE_{test}$ and R^2_{test} values of 0.75 +/- 0.01 and 0.74 +/- 0.01, respectively (Supplementary Table S15). To assess the extrapolation power on the cell line space, we used Leave-One-Tissue-Out (LOTO) validation ($RMSE_{test}$ =0.81+/-0.16 and R^2_{test} =0.72 +/-0.08), whereas Leave-One-Compound-Out validation was used to assess the predictive power on the compound space ($RMSE_{test}$ =1.40+/-0.80 and R^2_{test} =0.13 +/-0.11). We used Leave-One-Compound-Out instead of Leave-One-Compound-Cluster-Out validation given the low number of distinct compounds comprised in this data set, namely 139. All models were trained using: (i) 256-bit hashed Morgan fingerprints in count format using a maximum substructure radius of 2 bonds, and (ii) transcript levels for the 1,000 genes displaying the highest variance across the cell line panel. The results for (i) PGM models, and for (ii) the models reported in previous studies are given in Supplementary Table S15.

We note in particular that in Ammad-ud-din *et al.*, 2014 the extrapolation power of the models to

novel chemical structures was assessed by randomly dividing the compounds into 8 sets. A model was trained on all data-points comprising compounds from 7 sets. The trained model was then used to predict the bioactivities for the held-out data. This process was repeated 8 times, each time holding out the data from a different compound set. In this setting, which is similar to LOCCO except for the fact that compounds are not grouped based on a similarity, it is likely that the distribution of IC50 values for a given compound set spans a wide range of values, thus permitting to obtain high R^2 values for the observed against the predicted bioactivities (Supplementary Figure 14). By contrast, the range of IC50 values is likely to be much narrower for individual compounds across the cell line panel. Therefore, the R^2 values obtained with Leave-One-Compound-Out validation with PGM models are likely to be smaller than those obtained with LOCCO for the same accuracy in prediction, quantified with the RMSE value for the observed against the predicted bioactivities. Hence, it is important to note that although the R^2 values reported by Ammad-ud-din *et al.*, 2014 when assessing model extrapolation power on the chemical space, namely 0.52 +/- 0.37, might be higher in some cases than those obtained with Leave-One-Compound-Out validation, namely 0.13 +/- 0.11, this does not necessarily mean higher predictive power (Supplementary Figure 14). Therefore, the comparison between the two studies should be done in terms of RMSE values, which are 0.85 +/- 0.41 and 1.40 +/- 0.80 for Ammad-ud-din *et al.*, 2014 and our PGM models, respectively.

We note in particular that we did not apply the same validation as Ammad-ud-din *et al.*, 2014, namely partitioning the data set in 8 compound sets, as the composition of the 8 different sets was not reported by the authors.

Preparation of the CCLE data set

Gene transcript levels (Affymetrix U133+2 arrays), RMA-processed and normalized using quantile normalization, and compound IC50 values (μM) were downloaded from the CCLE website (https://www.broadinstitute.org/ccle/home) on February 16th 2015. IC50 values were converted to pIC50 values, *i.e.* –log10 IC50 (M), and to ln IC50 (μM).

The same learning strategies applied to the GDSC data set were applied here, namely: 10-fold cross-validation ($RMSE_{test}$ =1.02+/-0.05 and R^2_{test} =0.74 +/-0.03), LOTO ($RMSE_{test}$ =0.97+/-0.26

and R^2_{test} =0.75 +/-0.12) and Leave-One-Compound-Out ($RMSE_{test}$ =1.62+/-1.32 and R^2_{test} =0.18 +/-0.15). All models were trained using: (i) 256-bit hashed Morgan fingerprints in count format using a maximum substructure radius of 2 bonds, and (ii) the transcript levels for the 1,000 genes displaying the highest variance across the cell line panel. The results for these models and for previous studies are given in Supplementary Table S15.

Bibliography

Ammad-ud-din,M. *et al.* (2014) Integrative and Personalized QSAR Analysis in Cancer by Kernelized Bayesian Matrix Factorization. *J. Chem. Inf. Model.*, **54**, 2347–2359.

Menden,M.P. *et al.* (2013) Machine learning prediction of cancer cell sensitivity to drugs based on genomic and chemical properties. *PLoS One*, **8**, e61318.

Norinder,U. *et al.* (2014) Introducing Conformal Prediction in Predictive Modeling. A Transparent and Flexible Alternative To Applicability Domain Determination. *J. Chem. Inf. Model.*, **54**, 1596–1603.
